# Evolution of regulatory networks associated with traits under selection in cichlids

**DOI:** 10.1186/s13059-020-02208-8

**Published:** 2021-01-08

**Authors:** Tarang K. Mehta, Christopher Koch, Will Nash, Sara A. Knaack, Padhmanand Sudhakar, Marton Olbei, Sarah Bastkowski, Luca Penso-Dolfin, Tamas Korcsmaros, Wilfried Haerty, Sushmita Roy, Federica Di-Palma

**Affiliations:** 1 Earlham Institute (EI), Norwich, UK; 2 Department of Biostatistics and Medical Informatics, UW Madison, Madison, USA; 3 Wisconsin Institute for Discovery (WID), Madison, USA; 4 Quadram Institute, Norwich, UK; 5 Department of Computer Sciences, UW Madison, Madison, USA; 6 Norwich Medical School, University of East Anglia, Norwich, UK; 7 School of Biological Sciences, University of East Anglia, Norwich, UK

**Keywords:** Gene regulatory network, Co-expression, Cichlid, Opsin, Molecular evolution

## Abstract

**Background:**

Seminal studies of vertebrate protein evolution speculated that gene regulatory changes can drive anatomical innovations. However, very little is known about gene regulatory network (GRN) evolution associated with phenotypic effect across ecologically diverse species. Here we use a novel approach for comparative GRN analysis in vertebrate species to study GRN evolution in representative species of the most striking examples of adaptive radiations, the East African cichlids. We previously demonstrated how the explosive phenotypic diversification of East African cichlids can be attributed to diverse molecular mechanisms, including accelerated regulatory sequence evolution and gene expression divergence.

**Results:**

To investigate these mechanisms across species at a genome-wide scale, we develop a novel computational pipeline that predicts regulators for co-extant and ancestral co-expression modules along a phylogeny, and candidate regulatory regions associated with traits under selection in cichlids. As a case study, we apply our approach to a well-studied adaptive trait—the visual system—for which we report striking cases of network rewiring for visual opsin genes, identify discrete regulatory variants, and investigate their association with cichlid visual system evolution. In regulatory regions of visual opsin genes, in vitro assays confirm that transcription factor binding site mutations disrupt regulatory edges across species and segregate according to lake species phylogeny and ecology, suggesting GRN rewiring in radiating cichlids.

**Conclusions:**

Our approach reveals numerous novel potential candidate regulators and regulatory regions across cichlid genomes, including some novel and some previously reported associations to known adaptive evolutionary traits.

## Background

Seminal studies by King and Wilson [1] analyzing protein evolution in vertebrates speculated the importance of evolutionary changes in “regulatory processes” for morphological diversity [2, 3]. These ideas were soon expanded on by François Jacob [4], who suggested that the molecular “tinkering” of pre-existing systems is a hallmark of evolution where, for example, regulatory processes can either be transformed or combined for functional gain [4]. These theories underlie many studies on the divergence of regulatory processes associated with morphological evolution, and broadly focus on changes in gene regulatory networks (GRNs) that determine the expression patterns of genes [5, 6]. Such changes can be mutations within transcription factor binding sites (TFBSs) located in *cis-*regulatory elements (promoters and enhancers) of genes or *trans* regulatory changes that are due to changes in the level of a regulator [6]. Alterations of GRNs can lead to phenotypic divergence [7], and these GRN changes between species, irrespective of direct and indirect functional consequence, are defined as GRN “rewiring” events. This is characterized by regulatory interactions present in one or more species but absent in another species, and potentially replaced by a new interaction between the orthologous TF and a target gene. Several comparative studies of GRNs underlying mechanisms of adaptation and evolution have been carried out in unicellular prokaryotes, *E. coli* [8] and several non-vertebrate eukaryotes, including yeast [9, 10], plants [11], fruit fly [12], and echinoderms [12, 13]. While there are efforts to collate and integrate several genomic datasets for vertebrates, including human and mouse [14], comparative analysis of regulatory networks from these data alone remains a major computational challenge and very little is known about the phenotypic effect of genome-wide regulatory network rewiring events in non-model vertebrates [15].

In vertebrates, ray-finned fishes are the largest radiation of any group, and the East African cichlids represent arguably the most speciose modern examples of adaptive radiations. In the great lakes of East Africa (Tanganyika, Victoria, and Malawi) and within the last few million years [16, 17], one or a few ancestral lineages of cichlid fish have independently radiated to collectively give rise to over 1500 species. These species occupy a large diversity of ecological niches and differ dramatically in phenotypic traits, including skeletal morphology, dentition, color patterning, and a range of behavioral traits. We have previously demonstrated that a number of molecular mechanisms have shaped East African cichlid genomes, e.g., rapid evolution of regulatory elements and gene expression divergence [18], and the “evolutionary tinkering” of these systems [19] has provided the necessary substrate for diversification [18]. This, coupled with the recent origin of cichlid species and ongoing gene flow [20], suggests that evolutionary regulatory changes have an important functional role in controlling gene expression and, ultimately, phenotypic variation. However, very little is known about the genome-wide evolution of regulatory networks that may underlie several traits of cichlid phenotypic diversity. Here we developed a novel computational framework to characterize the evolution of regulatory networks and analyze the plausibility of whether the “tinkering” of regulatory systems could contribute towards phenotypic diversity in closely related cichlids.

## Results

### Gene co-expression is tissue-specific and highlights functional evolutionary trajectories

**Fig. 1:**
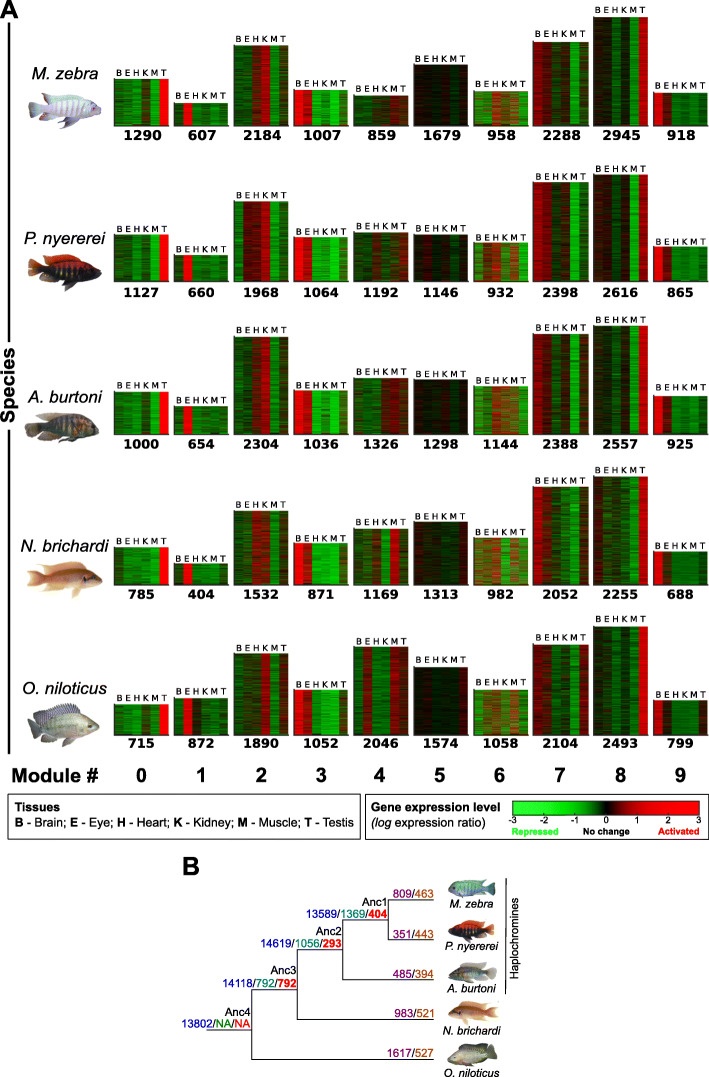
Evolution of gene expression in five cichlids. **a** Ten (0–9, heatmaps) co-expression modules identified by Arboretum [9] in six tissues of five cichlid species. Color bar denotes *log* expression ratio across each tissue, relative to the mean expression across all tissues—(red) activated, (green) repressed and (black) no change. Each heatmap shows the expression profile of genes assigned to that module in a given species and height is proportional to number of genes in the module (on *bottom*). **b** Number of state changes in module assignment of 1-to-1 orthologous genes along the five cichlid phylogeny [18]. Blue numbers: ancestral node genes assigned to modules; green numbers: state changes compared to the deepest common ancestor (Anc4); red numbers: state changes from last common ancestor (LCA); purple numbers: state changes in the one “focal” species compared to all other species; orange numbers: convergent state changes in each of the “focal” species and any of the other species

We applied the Arboretum [9] algorithm to RNA-seq data of six tissues in five species and identified 10 modules of 12,051–14,735 co-expressed genes (1205–1474 genes per module per species) represented across 18,799 orthogroups (Fig. 1a). Modules of co-expressed genes across the five species show varying expression levels in specific tissues, e.g., module 1 is eye specific, while module 3 is heart, kidney, and muscle specific (Fig. 1a). Consistent with the phylogeny and divergence times, there are more (13,171/18,799) orthologous genes that are conserved in module assignment (orthologous modules) in the three closely related haplochromines (*Pundamilia nyererei*, *Maylandia zebra*, and *Astatotilapia burtoni*) and *Neolamprologus brichardi*, than with *Oreochromis niloticus* (11,212/18,799 orthologous genes). Examples of modules where orthologs are not conserved in module assignment (non-orthologous modules) include modules 2, 4, and 6 (Additional file 1: Fig. S1a, blue off-diagonal elements) and are representative of gene expression divergence across the species. Between the haplochromines alone, 4179/18,799 orthologous genes are distributed in either one of two modules, e.g., 0 or 8 (Additional file 1: Fig. S1a, blue off-diagonal elements in haplochromines), indicative of gene expression divergence along the phylogeny.

The assignment of co-expressed gene modules by Arboretum [9] is inferred using a probabilistic framework starting from the last common ancestor (LCA) in the phylogeny. This allows us to model the evolutionary trajectory of orthologous genes and their co-expression along the species tree [9]. Orthologous genes of each species can be assigned to non-orthologous modules (Fig. S-R1a), indicative of co-expression divergence and potential transcriptional rewiring from the LCA; this is referred to as “state changes” in module assignment. In total, 7587/18,799 (40%) orthologous genes exhibit state changes in module assignment across branches. To ensure orthologous genes of all branches are included in subsequent analysis, we focused on state changes of 6844 1-to-1 orthologous genes to assess convergent and unique state changes along the phylogeny (Fig. 1b). We identified convergent state changes of 732 genes along all ancestral nodes versus Anc4 (Additional file 1: Fig. S2). This is made up of 772 genes in Anc3 and Anc2, 734 genes in Anc3 and Anc1, and 996 genes in Anc2 and Anc1 (Additional file 1: Fig. S2), including a few TFs (46 TFs—Anc3-2-1; 49 TFs—Anc3-2; 46 TFs—Anc3-1; 66 TFs—Anc2-1) such as *tbx20*, *nkx3-1*, and *hoxd10.* We identified unique state changes and expression divergence of 655 genes along ancestral nodes (Fig. 1b), including several cellular and developmental TFs (51 TFs—Anc4/3; 20 TFs—Anc3/2; 34 TFs—Anc2/1) such as *foxo1*, *hoxa11* and *lbx1*. Several of these state changed regulatory TFs are also enriched (fold enrichment 1.1–1.7; false discovery rate, FDR < 0.05) in gene promoters of relevant tissue-specific modules; for example, promoters of module 1 genes (eye-specific expression) are significantly enriched (fold enrichment 1.1–1.6; FDR < 0.05) for TF motifs involved in retina- and lens-related development/functions, e.g., CRX, PITX3, and OTX1 [21] (Additional file 1: Fig. S3, Additional file 2: Fig. S2). Further examination identifies that there are differences in the levels of TF motif enrichment across species genes, including that of retina/lens-related TFs, e.g., RAR*α/β/γ* and RXR*α/β/γ* [22] of module 1 gene promoters in all species except *N. brichardi* (Additional file 1: Fig. S3, Additional file 2: Fig. S2). Such differences in motif enrichment could be associated with changes in the level of TF expression, where state changes (Fig. 1b) reflect shifted domains of tissue expression and imply differential regulatory control of target genes across tissues and along the phylogeny. We tested this by taking (1) the *log* expression ratio (as used for Arboretum input), for all 337 expressed TFs in each species tissue; (2) the corresponding 2064 TF motif enrichment scores (−*log q*-value, FDR < 0.05) calculated across 12,051–14,735 promoters regions of all species genes in the 10 modules; and (3) calculating the cross-species Pearson correlation coefficient (*r*) between the motif enrichment score and expression value of each TF and in each tissue (Additional file 2: Fig. S3-S8) using the *n* = 5 species. We note different patterns of correlation between cross-species TF motif enrichment and tissue-specific expression; in total, 102–119/337 TFs had no correlation (0 ≤ *r* ≤ 0.01, *n* = 5) and included many TFs that had large shifts in motif enrichment and/or expression in several species, representative of several phylogenetic state changes, e.g., Kidney-Module2-FOXO1 (*r* = 0.01, *n* = 5) (Extended Data S-R1F). On the other hand, there is positive correlation ranging from small (0.1 ≤ *r* ≤ 0.3, *n* = 5) for 161–197 TFs, medium (0.3 < *r* ≤ 0.5) for 161–186 TFs, and large values (0.5 < *r* ≤ 1) for 226–262 TFs. The largely correlated TFs (0.5 < *r* ≤ 1) includes cases where there is comparable motif enrichment scores across species, as calculated by the variance distribution (see “Methods”), and either no shifts (no TF state changes), e.g., Brain-Module9-FOXA2 (*r* = 0.97, *n* = 5, *p* value < 0.05) or focused shifts (TF state change in one or subsets of species), e.g., Eye-Module2-CDX1 (*r* = 0.98, *n* = 5, *p* value < 0.05) in TF tissue expression (Additional file 1: Fig. S5, Additional file 2: Fig. S3-S8). Such patterns of focused shifts in expression are also observed in TFs of selected modules like, for example, module 1 which contains eye-expressed genes. We find that retinal TFs that are known to modulate opsin expression, e.g., CRX [23], have variable motif enrichment (fold enrichment 1.2–1.4) in eye-expressed genes, and are associated (*r* = 0.85, *n* = 5, *p* value < 0.1) with a concurrent change (increase in four species or decrease in *N. brichardi*) in TF eye expression along the phylogeny (Additional file 1: Fig. S6; see Additional file 1*text*). For most TFs (226–262/337 TFs) and tissues, motif enrichment is largely correlated (0.5 < *r* ≤ 1) with TF expression. After calculating the variance of each TF motif enrichment and categorizing the tails into either similar or dissimilar levels of TF motif enrichment (see “Methods”), we note that similar motif enrichment (across species) is associated with either expression conservation (across all species) or subtle expression changes (in one or subsets of species) and is more stable (in expression differences) than TFs with dissimilar/variable motif enrichment along the phylogeny (Additional file 2: Fig. S3-S8). Gene co-expression differences and convergence between species could therefore be driven by differences in TF motif levels in gene promoter regions.

### Fine scale nucleotide variation at TF binding sites drives regulatory divergence in cichlids through GRN rewiring

*Cis-*regulatory elements, including promoters and enhancers, are central to gene expression regulation, largely acting through the binding of TFs to multiple transcription factor binding sites (TFBSs). Therefore, mutations within TFBSs can alter target gene transcription without affecting the expression pattern of other genes co-regulated by the same TF, thus driving GRN evolution. In the five cichlid genomes however, there is no significant increase in evolutionary rate at promoter regions compared to fourfold degenerate sites (Additional file 1: Fig. S7). However, we identify a few outlier genes with significantly higher evolutionary rate at promoter regions at ancestral nodes (12–351 genes, Additional file 1: Fig. S7b) and within species (29–352 genes, Additional file 1: Fig. S7d), indicative of small-scale changes in promoter regions (see Additional file 1 text). Concurrently, of all the identified pairwise species variation (8 to 32 million variants), a large proportion (13–28%) overlap predicted TFBSs in promoter regions, and this is higher than (8–9%) of variants that are present in flanking gene promoter regions of the same length (Additional file 1: Table S2, Additional file 1: Fig. S8). GO enrichment analysis of co-expressed genes with variation in their regulatory regions, against a background of all genes in each genome, highlights associations with key molecular processes, e.g., signal transduction-promoter TFBSs (Additional file 1: Fig. S9).

To further investigate patterns of divergent regulatory programs that could be associated with discrete nucleotide variation at regulatory binding sites, we developed and applied a computational framework (see “Methods,” Additional file 1: Fig. S20) to comparatively study regulatory interactions/relationships across the five cichlids. This involved the reconstruction of species-specific GRNs through the integration of different genomic datasets (Additional file 1: Table S3). We focused on regulatory interactions/relationships of trans-acting factors (TFs) and DNA (gene promoter regions); this involved integrating an expression-based network with in silico predictions of TF binding to target gene (TG) promoters using our cichlid-specific and vertebrate-wide TF motif scanning pipeline (see “Methods,” Additional file 1: Fig. S20). We first used species- and module-specific gene expression levels to infer an expression-based network [24] (see “Methods,” Additional file 1: Fig. S20), generating 3180–4099 transcription factor-target gene (TF-TG) edges across the five species (FDR < 0.05, Additional file 1: Table S3). Next, based on our in silico TFBS motif prediction pipeline, we predicted TFBS motifs up to 20 kb upstream of a gene transcription start site (TSS), and using sliding window analysis of 100 nucleotides (nt), we retained TF motifs in the gene promoter region, defined as up to 5 kb upstream of a gene TSS (see “Methods,” Additional file 1: Fig. S22). Each statistically significant TFBS motif (FDR < 0.05) was associated to its proximal target gene (TG) and represented as two nodes and one TF-TG edge. Based on the integrated approach (see “Methods,” Additional file 1: Fig. S20), we predicted a total of 3,295,212–5,900,174 TF-TG edges (FDR < 0.05) across the five species that could be encoded into a matrix of 1,131,812 predicted TF-TG edges (FDR < 0.05), where each edge is present in at least two species. To ensure accurate analysis of GRN rewiring and to retain relevant TF-TG interactions, all collated edges were then further pruned to a total of 843,168 TF-TG edges (FDR < 0.05) where (1) the edge is present in at least two species; (2) edges are not absent in any species due to node loss or mis-annotation; and (3) edges are based on the presence of nodes in modules of co-expression genes (see “Methods”).

**Fig. 2:**
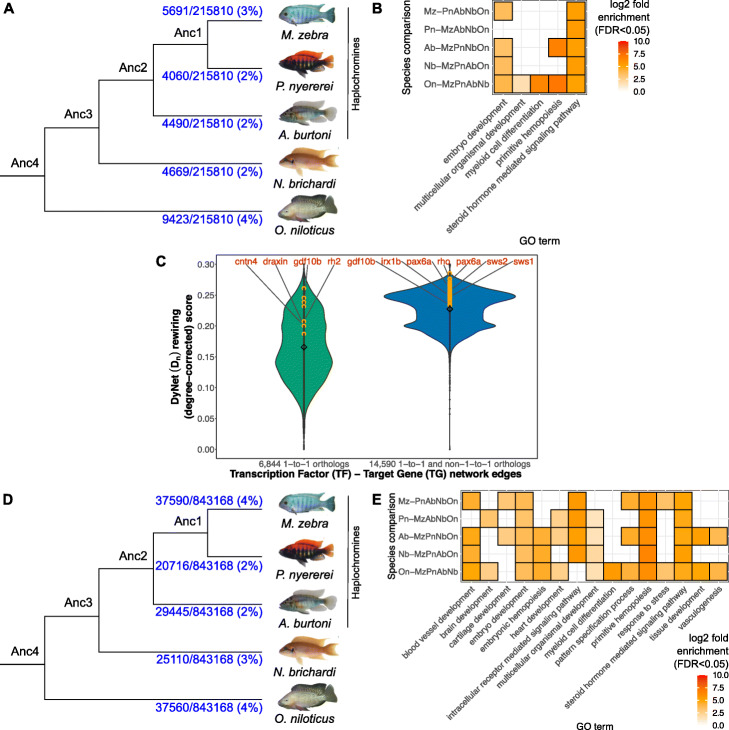
Network rewiring between species including TFs state changing co-expression module assignment and their targets (TGs). **a** Rewiring events linked to module assignment state changes of TFs in 215,810 TF-TG 1-to-1 edges (FDR < 0.05) of each species in the cichlid phylogeny compared to the other four species (see Additional file 1 for other FDR thresholds). **b** GO term enrichment of the 50–70 TFs that are rewired and state changed, and their associated TGs in one “focal” species vs the other four species (4060-9423/215,810 TF-TG 1-to-1 edges, **a**) against a background of all module genes, shown as grid heatmap of *log10* fold enrichment (legend on *right*, FDR < 0.05). **c** Violin plots of DyNet (*D*_*n*_) rewiring score (degree-corrected) from 6844 1-to-1 orthologs in 215,810 TF-TG network edges (green, left violin) and 14,590 1-to-1 and many-to-many orthologs in 843,168 TF-TG network edges (blue, right violin). Mean rewiring score shown within each plot (white diamond). Degree-corrected rewiring score shown for non-candidate genes (black dots through center) and candidate morphogenetic trait genes (orange dots) with rewiring scores higher than the mean, and selected candidate examples are demarcated within. **d** Rewiring events linked to module assignment state changes of TFs in 843,168 TF-TG all edges of each species in the cichlid phylogeny against the other four species. **e** GO term enrichment of 100–140 rewired and state changed TFs and their associated TGs in each focal species vs the other 4 species (20,716-37,590/843,168 edges TF-TG all edges, **d**) against a background of all module genes, shown as grid heatmap of *log10* fold enrichment (legend on *right*, FDR < 0.05)

We used three metrics to study large-scale TF-TG network rewiring between species that included: (1) state changes in module assignment; (2) DyNet [25] network rewiring scores; and (3) TF rate of edge gain and loss in networks. The first metric compares TF-TG edges of a single “focal” species versus the other species in the context of gene co-expression, while the second and third metric compute a likelihood score for the overall extent of edge changes (across all species) associated with single nodes of interest. We first focused on 6844 1-to-1 orthologous genes represented in 215,810 TF-TG interactions, termed “TF-TG 1-to-1 edges,” along the five cichlid tree. Using a background set of all module genes (18,799 orthogroups), the TF-TG 1-to-1 edges are associated with morphogenesis and cichlid traits under selection, e.g., eye and brain development (FDR < 0.05, Additional file 1: Fig. S10a). There are 379 TFs represented in the TF-TG 1-to-1 edges, and we focus on their interactions/relationships to determine whether TFs with (state) changes in module assignment have altered regulatory edges. In the first metric, rewiring is characterized as a unique TF-TG edge present in only one “focal” species, where the TF node is (1) state changed in module assignment and (2) present as a node in different TF-TG edges in any/all of the other species. Using this metric, 50–70 out of the 379 TFs (13–18%) are rewired (spanning 4060–9423/215,810 edges, FDR < 0.05, Fig. 2a; see Additional file 1 text) and change module assignment across the five species (in one focal vs all four other species). The gene nodes connected by the rewired edges are associated with signalling pathways and processes such as cell differentiation and embryonic development (FDR < 0.05, background of all module genes, Fig. 2b). Further examination of rewiring rates in the networks of 6844 1-to-1 orthologous genes (in 215,810 TF-TG interactions) using the DyNet [25] degree-corrected rewiring (*D*_*n*_) score (Fig. 2c, Additional file 3: Table S1) identifies rewired networks of nine teleost and cichlid trait genes associated with morphogenesis from previous studies (Fig. 2c, Additional file 3: Table S2). These genes have a few standard deviations higher degree-corrected rewiring (*D*_*n*_) score than the mean (0.17 ± 0.03 SD), and their rewiring scores are comparatively higher (Kolmogorov–Smirnov KS-test *p* value = 6 × 10^− 4^) than all 1-to-1 orthologs (Fig. 2c, left violin plot, orange dots; Additional file 3: Table S3; see Additional file 1 text). Examples of these rewired 1-to-1 genes include *gdf10b* associated with axonal outgrowth and fast evolving in cichlids [18] and the visual opsin gene, *rh2* (Fig. 2c, left violin plot; Additional file 3: Table S3 S-R3C). To enable a genome-wide study of network rewiring, we extend our analyses beyond the 6844 1-to-1 orthologs only, by including an additional 7746 many-to-many orthogroups (see “Methods”) resulting in a set of 843,168 “TF-TG all edges” across the five species. Using a background set of all module genes (18,799 orthogroups), the gene nodes in the 843,168 TF-TG all edges are associated with morphogenesis, e.g., retina development (FDR < 0.05, Fig. SR3aB). These edges include interactions of 783 TFs of which 13–18% (100–140 TFs) are predicted to be rewired (in 20,716-37,590/843,168 edges, FDR < 0.05, Fig. 2d) and change module assignment across the five species (in one focal vs all four other species), indicating their associated transcriptional programs (FDR < 0.05, background of all module genes) are also altered (Fig. 2e). By examining the network rewiring rates of 14,590 orthogroups (in 843,168 TF-TG interactions, Additional file 3: Table S4) using DyNet [25], we identify 60 candidate teleost and cichlid trait genes associated with phenotypic diversity from previous studies (Fig. 2c, *right* violin plot; Additional file 3: Table S5). These genes have a few standard deviations higher degree-corrected rewiring (*D*_*n*_) score than the mean (0.23 ± 0.007 SD) of all orthologs, and their rewiring score is comparatively higher (KS-test *p* value = 6 × 10^− 14^) (Fig. 2c, *right* violin plot, orange dots; Additional file 3: Table S4). These genes include those associated with craniofacial development, e.g., *dlx1a* and *nkx2-5* [21], telencephalon diversity, e.g., *foxg1* [26], tooth morphogenesis, e.g., *notch1* [27], and strikingly, most visual opsins, e.g., *rho*, *sws2*, and *sws1*, as well as genes associated with photoreceptor cell differentiation, *actr1b* [28], and eye development, *pax6a* [21] (Fig. 2c, *right* violin plot; Additional file 3: Table S5). We then focus on the gain and loss rates of 186/783 TFs with > 25 TF-TG edges along the five cichlid tree (see “Methods”). Out of the 186 TFs, 133 (72%) are predicted to have a higher rate of edge gain than loss, e.g., DLX5 and NEUROD2, possibly acting as recruited regulators of gene expression in each branch from their last common ancestor (LCA) (Additional file 3: Table S6), whereas 53/186 TFs (28%) have a higher loss of edges than gains, e.g., OLIG2 and NR2C2, implying loss of gene expression regulatory activity from their LCA (Additional file 3: Table S6). In general, TFs and their binding sites are evolving towards gaining, rather than losing regulatory edges from their LCA.

To further characterize the role of the observed changes in *cis*-regulatory elements and their potential association with cichlid traits, we extended our analyses to include several radiating cichlid species. We screened all predicted TFBS (see “Methods”) variants between *M. zebra* (a Lake Malawi species) and the other four cichlids, with their corresponding positions in 73 phenotypically distinct Lake Malawi species [20], to identify between-species variation at regulatory sites along the phylogeny (Additional file 1: Fig. S11). As expected, the majority of variation at regulatory sites is identified between *M. zebra* and distantly related Lake Malawi species clades, e.g., NKX2.1 TFBS in *sws1* gene promoter, whereas shared ancestral sites are found with mainly same/closely related Lake Malawi clades, e.g., EGR2 TFBS in *cntn4* gene promoter. Genes that are associated with traits under selection, e.g., visual systems [29] (*sws1)* and morphogenesis [18] (*cntn4*), harbor between species regulatory variants that segregate according to phylogeny and ecology of radiating lake species.

### *Cis*-regulatory changes lead to GRN alterations that segregate according to phylogeny and ecology of radiating cichlids

Through our comparative approach, we can examine the regulatory network topology of several genes that are important for cichlid diversification [30, 31] and represented by our six tissues. As a case study, we focus on the cichlid visual system; the evolution of cichlid GRNs and diverse palettes of co-expressed opsins can induce large shifts in adaptive spectral sensitivity of adult cichlids [29], and thus, we hypothesize that opsin expression diversity is the result of rapid adaptive GRN evolution in cichlids. Indeed, by focusing on species utilizing the same wavelength visual palette and opsin genes, we note that several visual opsin genes (*rh2b*, *sws1*, *sws2a*, and *rho*) have considerably rewired regulatory networks (Additional file 3: Table S6). Across the predicted transcriptional networks of cichlid visual opsins, there are several visual-system-associated regulators (TFs) of opsin genes (*sws2a*, *rh2b*, and *rho*) that are either common, e.g., STAT1A, CRX, and GATA2, or unique to each species, e.g., IRF1, MAFA, and GATA2A (Additional file 1: Fig. S12–14). These patterns of TF regulatory divergence could therefore contribute to differential opsin expression.

**Fig. 3:**
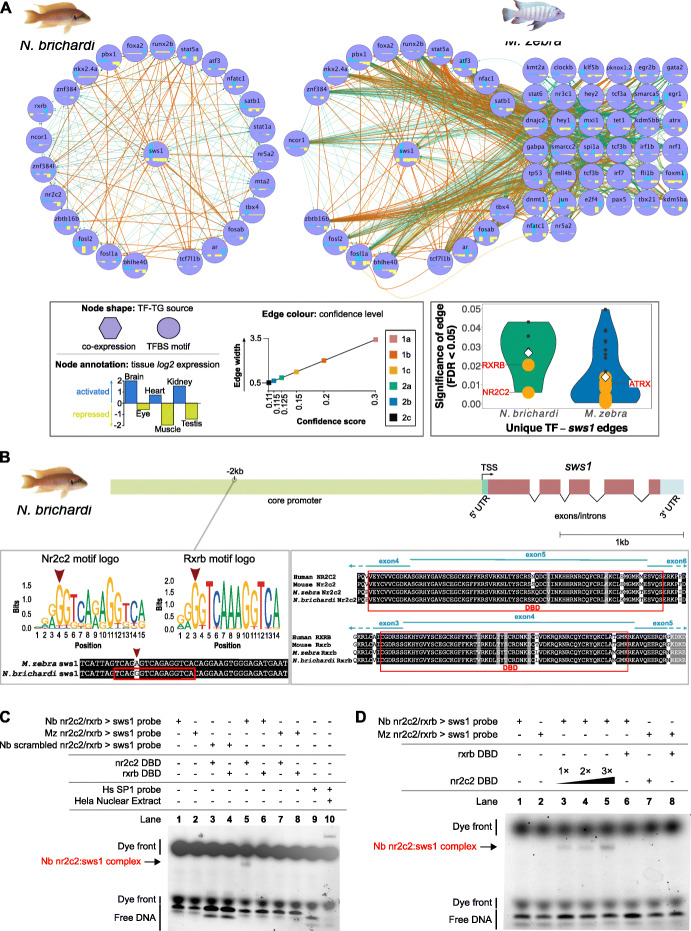
Evolution of the *sws1* opsin regulatory networks in *N. brichardi* and *M. zebra.* **a** Reconstructed regulatory networks of *sws1* opsin shown for *N. brichardi* (*left*) and *M. zebra* (*right)*: circular layout nodes are common regulators (unless missing); grid layout nodes are unique regulators in *M. zebra*. Node shape, annotation and edge color denoted in legend to *left bottom*. Violin plot of significance (FDR < 0.05) of unique TF-*sws1* edges in *N. brichardi* (*green violin*) and *M. zebra* (*blue violin*) to *bottom right*—mean edge significance score shown within each plot (white diamond); edges more than the mean (less significant) are shown as gray dots, and edges less than the mean (more significant) are shown as orange dots; selected example TFs are demarcated within*.* **b** On the *left*, NR2C2 and RXRB motif logos and motif prediction in negative orientation *N. brichardi sws1* gene promoter (red box) and variant in *M. zebra sws1* gene promoter (red arrow). On the *right*, NR2C2 and RXRB partial protein alignment showing DNA-binding domain (DBD) annotation in human, mouse, *M. zebra* and *N. brichardi.* **c** EMSA validation of NR2C2 and RXRB DBD binding to *N. brichardi* and *M. zebra sws1* gene promoter. Table denotes combinations of DNA probe and expressed DBD in EMSA reactions that include negative controls (lanes 1 to 4); *N. brichardi* protein: DNA-binding assay (lanes 5 and 6); *M. zebra* protein: DNA-binding assay (lanes 7 and 8); kit negative (lane 9) and binding positive control (lane 10). Protein:DNA complexes, dye front and free DNA are indicated by arrowhead and bracket within. **d** EMSA validation of increasing NR2C2 DBD concentrations and binding to predicted TFBS in *N. brichardi sws1* gene promoter

*Sws1* (ultraviolet) opsin is utilized as part of the short-wavelength sensitive palette in *N. brichardi* and *M. zebra*. While there are common regulators associated with retinal ganglion cell patterning in both species networks, e.g., SATB1 [32], there are also several unique regulators associated with nuclear receptor signalling, e.g., RXRB and NR2C2 [33], and retinal neuron synaptic activity, e.g., ATRX [34] (Fig. 3a). Overall, using a significance threshold of FDR < 0.05 for predicted TF-TG edges, there are more predicted unique TF regulators of *sws1* in *M. zebra* (38 TFs) as compared to *N. brichardi* (6 TFs) (Fig. 3a, *bottom right*). Furthermore, we identify that a candidate regulatory variant has likely broken the *M. zebra* NR2C2/RXRB shared motif that is otherwise predicted 2 kb upstream of the *N. brichardi sws1* TSS (Fig. 3b). Functional validation via EMSA confirms that NR2C2 and not RXRB binds to the predicted motif in the *N. brichardi sws1* promoter, forming a complex, and the variant has likely disrupted binding, and possibly regulation of *M. zebra sws1* (Fig. 3c, d). This is further supported by correlating expression values of these regulators and *sws1*, where NR2C2 is better associated with *sws1* than RXRB, particularly when focusing on eye tissue (Additional file 1: Fig. S16a *on right*; Additional file 1: Fig. S16b; see Additional file 1*text*).

**Fig. 4:**
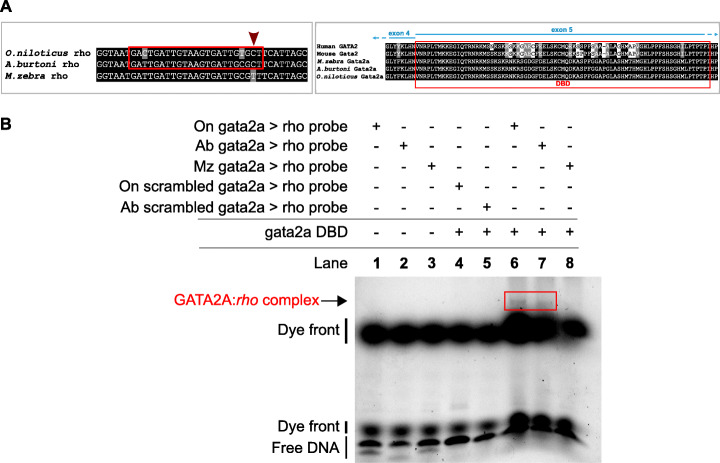
Evolution of the *rhodopsin* regulatory networks in *O. niloticus*, *A. burtoni* and *M. zebra.* **a** On the *left*, GATA2A motif prediction in reverse orientated *O. niloticus* and *A. burtoni rhodopsin* gene promoter (red box) and substitution demarcated in *M. zebra rhodopsin* gene promoter (red arrow). On the *right*, GATA2A partial protein alignment showing DNA-binding domain (DBD) annotation in human, mouse, *O. niloticus, A. burtoni* and *M. zebra.* **b** EMSA validation of GATA2A DBD binding to *O. niloticus* and *A. burtoni rhodopsin* gene promoter. Table denotes combinations of DNA probe and expressed DBD in EMSA reactions that include negative controls (lanes 1 to 5); *O. niloticus* (lane 6), *A. burtoni* (lane 7) and *M. zebra* (lane 8) protein: DNA-binding assays. GATA2A:*rho* complex formed in *O. niloticus* (lane 6) and *A. burtoni* (lane 7) as confirmed by band shift (red box) and no complex formed in *M. zebra* (lane 8)

In another example, *rhodopsin (rho)*, associated with dim-light vision, is predicted to be regulated by GATA2 in *O. niloticus*, *A. burtoni*, and *M. zebra* but not its duplicate gene, GATA2A only in *M. zebra* (Additional file 1: Fig. S14). We identify a candidate variant (red arrow, Fig. 4a) that has likely broken the *M. zebra* GATA2A motif that is otherwise predicted 1.8 kb and 1.9 kb upstream of the *O. niloticus* and *A. burtoni rho* TSS (Fig. 4a). Functional validation via EMSA confirms that GATA2A binds to the predicted motif in the *O. niloticus* and *A. burtoni rho* promoter, and the variant is likely to have disrupted binding, and possibly regulation of *M. zebra rho* (Fig. 4b). Species-specific expression correlations with the *rho* target gene are supportive of GATA2’s possible conserved role in all three species (*O. niloticus r* = 0.89; *A. burtoni r* = 0.39; *M. zebra r* = 0.28, *n* = 6 Additional file 1: Fig. S17c), while a more divergent role of GATA2A (*O. niloticus r* = 0.79 and *A. burtoni r* = 0.21, *n* = 6) and negative correlation in *M. zebra* (*r* = − 0.18, *n* = 6) is supportive (Additional file 1: Fig. S17c) of the EMSA validation (Fig. 4). This further supports the notion that discrete point mutations in TFBSs could be driving GRN evolution and rewiring events in traits that are under selection in radiating cichlids.

**Fig. 5:**
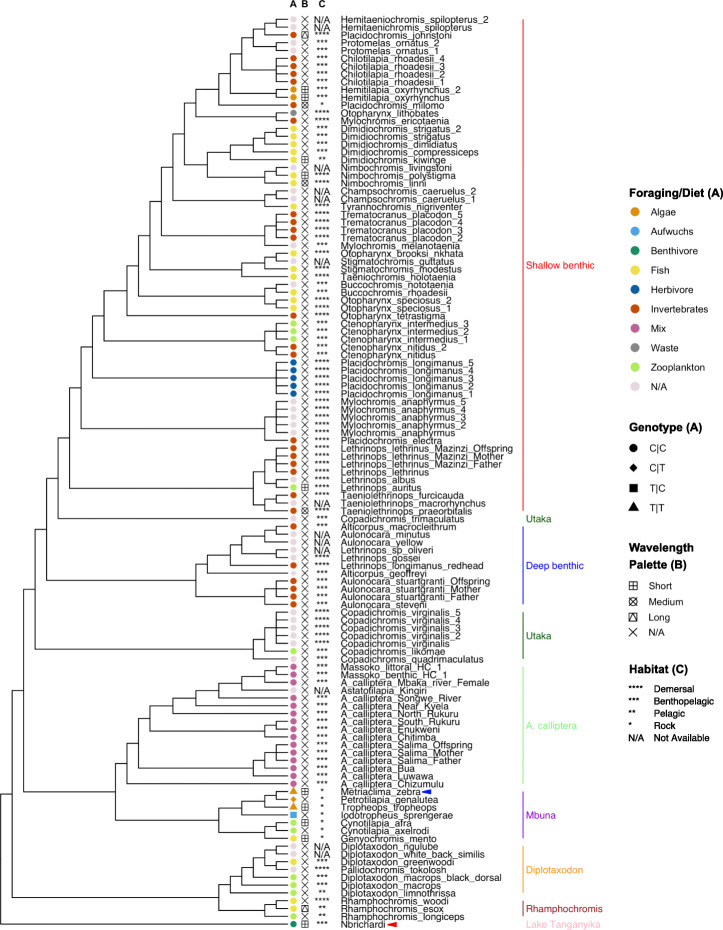
SNP genotypes overlapping NR2C2 TFBS in *M. zebra sws1* promoter and other Lake Malawi species. Lake Malawi phylogeny reproduced from published least controversial and all included species ASTRAL phylogeny [20], including *N. brichardi* as an outgroup. Phylogenetic branches labelled with species sample name (including *M. zebra* with blue arrow and *N. brichardi* with red arrow) and clade according to legends (*right*): **a** species foraging/diet habit (color) [35] and phased SNP genotype (shape) [20]; **b** adult opsin wavelength palette utilized [35] and **c** species habitat [35, 36]

Finally, we studied GRN rewiring as a result of between species TFBS variation in the context of phylogeny and ecology of lake species. Owing to the variability and importance of spectral tuning of visual systems to the foraging habits of all cichlid species, we focused on variants at regulatory sites of rewired visual opsin genes in the Lake Malawi species, *M. zebra*, as a reference to compare GRN rewiring (through TFBS variation) that could be associated with the ecology of sequenced Lake Malawi species [20]. If indeed the TFBSs are likely functional, we hypothesize that radiating species with similar foraging habits would share conserved regulatory genotypes, to possibly regulate and tune similar spectral sensitivities, whereas distally related species with dissimilar foraging habits would segregate at the corresponding regulatory site. For this, we started with 157,232 sites that (1) have identified variation between the five cichlid species and (2) are located in TFBSs of *M. zebra* candidate gene promoters. We identified 5710/157,232 sites with between species variation across 73 Lake Malawi species (Additional file 1: Fig. S11) that also exhibited flanking sequence conservation, representative of shared ancestral variation. The homozygous variant (T|T) that breaks binding of NR2C2 to *M. zebra sws1* promoter (Fig. 3 and Fig. 5*blue arrow*) is (1) conserved with the fellow algae eater, *Tropheops tropheops*, that also utilizes the same short-wavelength palette; (2) heterozygous segregating *(Petrotilapia genalutea*—C|T and *Iodotropheus sprengerae*—T|C) in closely related Mbuna species; and (3) homozygous segregated (C|C) in distantly related Mbuna species (*Cynotilapia afra*, *Corydoras axelrodi*, and *Genyochromis mento*) and most other Lake Malawi species of which some utilize the same short-wavelength palette and are algae eaters, e.g., *Hemitilapia oxyrhynchus* (Fig. 5). This suggests that in species closely related to *M. zebra*, and with a similar diet and more importantly, habitat, *sws1* may not be regulated by NR2C2, whereas in other species it could be, similar to *N. brichardi* (Fig. 3 and Fig. 5*red arrow*)*.* In another example, regulation of *rho* by GATA2, and not its duplicate, GATA2A (Fig. 4), could be sufficient for regulating dim-light vision response in rock dweller species (*M. zebra* and possibly *Petrotilapia genulatea*, *Tropheops tropheops* and *Iodotropheus sprengerae*), but both *gata2* copies could be required to regulate *rho* in many other Lake Malawi species (79% with C|C genotype that otherwise predicts the GATA2A TFBS in *rho* gene promoter), as well as *A. burtoni* and *O. niloticus* (Additional file 1: Fig. S14–15)*.* This highlights the potential differential usage of a duplicate TF in dim-light vision regulation. Phylogenetic independent contrast analysis [37] of the NR2C2-*sws1* (Additional file 1: Fig. S18a-f) and GATA2A-*rho* (Additional file 1: Fig. S19a-f) genotypes against visual traits and ecology of each of the 73 Lake Malawi species highlights very little change in correlation once the phylogeny is taken into account and a regression model fitted. Based on these examples of TFBS variants that segregate according to phylogeny and ecology of lake species, GRN rewiring through TFBS variation could be a key contributing mechanism of evolutionary innovation, especially visual systems, in East African cichlid radiations.

## Discussion

The evolutionary “tinkering” of regulatory systems through GRN divergence can facilitate the evolution of phenotypic diversity and rapid adaptation [19]. Various mechanisms underlie these events, including horizontal gene transfer and regulatory reorganization in bacteria [38]; gene duplication in fungi [39]; *cis-*regulatory expression divergence in flies [40]; variable gene co-expression in worms [41]; dynamic rewiring of TFs in plant leaf shape [11]; coding and non-coding evolution in stickleback fish [42]; alternative splicing [43], and differential rate of gene expression evolution shaped by various selective pressures [44, 45] in mammals. However, since very little is known about the combined effect of some of these mechanisms; in-depth analyses of regulatory network evolution can shed light on the key contributing mechanisms associated with phenotypic effect across ecologically diverse species in a phylogeny.

The three great lakes of East Africa (Tanganyika, Victoria, and Malawi) have independently experienced rapid radiations and explosive diversification of well over 1500 cichlid species. Alongside ecological opportunity [17], East African cichlid diversification has been shaped by complex evolutionary and genomic forces, including divergent selection acting upon regulatory regions [18] that is largely based on a canvas of low genetic diversity between species [20]. All of these findings imply the rapid evolution of regulatory networks underlying traits under selection; however, little is known about the genome-wide evolution of regulatory networks that may underlie several traits of cichlid phenotypic diversity [46]. Here we developed a novel approach to identify and compare gene regulatory modules and networks across six tissues of five East African cichlid species.

Along the phylogeny, our analyses identified gene co-expression modules with tissue-specific patterns and differential trajectories across six tissues of five cichlids. Between the haplochromine species alone, nearly a quarter of all orthologous genes are distributed in either one of two modules. Considering the smaller divergence time of the three haplochromines (~ 6 MYA) and the three haplochromines vs *O. niloticus* (~ 19 MYA) [47], this indicates gene expression divergence over different evolutionary timescales and co-expression of different clusters of genes across species. Given that the volumes and, hence, representation of region-specific cell types of selected organ, e.g., brain regions can be different, even between closely related cichlids [48], it is plausible that the observed expression differences between species are driven by changes in cell type abundances. However, given that expression data was generated from the organs of multiple similarly sized adult individuals and the identification of conserved tissue-specific patterns across all tissues and species, e.g., module 1 is eye specific (Fig. 1a), we suspect that the majority of observed co-expression differences are connected to gene regulatory differences. Indeed, these genes are predicted to be regulated by divergent suites of regulators, including TFs that are state changed in co-expression module assignment. This suggests that gene co-expression differences and convergence between species could be driven by differences in TF motif levels in gene promoter regions and could be associated with gene regulatory changes underpinning traits under selection in cichlids, such as the visual system [29]. In the five cichlids, transcriptional rewiring events and differential gene expression could therefore contribute to phenotypic diversity of the six studied tissues.

*Cis*-regulatory elements (including promoters and enhancers) are central to cichlid gene expression regulation [18], and in this study, we show that discrete nucleotide variation at binding sites drives regulatory edge divergence through GRN rewiring events. Comparative analysis of GRNs across species identifies that TFs and their binding sites are evolving towards gaining, rather than losing regulatory edges, and possibly regulatory activity of genes from their LCA. Comparative GRN analysis also identified striking cases of rapid network rewiring for genes known to be involved in traits under natural and/or sexual selection, such as the visual system, possibly shaping cichlid adaptation to a variety of ecological niches. While there are common regulators of the *sws1* visual opsin in two species (*N. brichardi* and *M. zebra*) sharing the same short-wavelength palette, the *sws1* networks of these two species have substantially diverged. Such tight TF-based regulation of *N. brichardi sws1* could induce rapid shifts in expression and spectral shift sensitivities between a larger peak ƛ_max_ of 417 nm in *N. brichardi* single cones [49] compared to 368 nm of *M. zebra* SWS1 [50]. Also, diverse regulation in *M. zebra* can increase *sws1* expression and, in turn, increase spectral sensitivity to UV light and the ability for *M. zebra* to detect/feed on UV-absorbing phytoplankton and algae, as previously shown for Lake Malawi cichlids [35]. In regulatory regions of *sws1*, in vitro assays confirm that variations in TFBSs (NR2C2) have driven network structure rewiring between the two species (*N. brichardi* and *M. zebra*) sharing the same visual palette. Since the modulation of cichlid visual sensitivity occurs through heterochronic shifts in opsin expression [51], our results are consistent with recent findings that visual tuning differences between cichlid species require regulatory mutations that are constrained by mutational dynamics [52].

Gene duplications have also been implicated in cichlid evolutionary divergence, including differences in duplicate TF gene expression [18]. However, due to incomplete lineage sorting (ILS) and variability in duplicates identified by three separate methods (gene trees, read-depth analyses and array comparative genomic hybridization) [18], we instead focus on particular examples of gene duplication associated with network rewiring of visual system genes. We predict that the dim-light vision gene, *rho*, is regulated by GATA2 and potentially common to regulating dim-light vision in *M. zebra, A. burtoni*, and *O. niloticus* but a duplicate TF, GATA2A, is predicted to be a unique regulator of *rho* in *A. burtoni* and *O. niloticus* only, owing to a variant in the GATA2A TFBS of the *M. zebra rho* gene promoter. Furthermore, *M. zebra* variants overlapping TFBSs in gene promoter regions, e.g., *sws1* (NR2C2) and *rho* (GATA2A) segregate according to phylogeny and ecology of Lake Malawi species [20], suggesting ecotype-associated network rewiring events could be linked to traits under selection in East African cichlid radiations. This is consistent with the adaptive potential of visual system evolution in cichlid species, where changes in spectral tuning of visual signals are likely to lead to dramatic species evolution and possibly speciation events [53]. Given that single regulatory mutations of *Tbx2a* can cause heterochronic shifts in opsin expression and visual tuning diversity between two distinct cichlid species [52], it is likely that the regulatory variation at opsin gene promoter TFBSs that we have predicted and experimentally validated, is a contributing mechanism of evolutionary innovation across many cichlid species. Furthermore, the identification (in predicted TFBSs) of segregating sites across several Lake Malawi species, with conservation of flanking regions, is indicative of shared ancestral variation and functional evolutionary constraint. The differences we identify at opsin gene promoter TFBSs and their implications in visual tuning could correspond to species variation of habitat choice, foraging habits, diet, and male nuptial coloration. Phylogenetic independent contrast analysis [37] shows that fitting the Lake Malawi phylogeny has little effect on the correlation between regulatory genotypes, visual traits, and ecology, suggesting possible covariance between these genotypes and traits. However, given the weak correlation (low adjusted *r*^2^ and *p* values), the impact of ecotype-associated network rewiring events requires further testing. This analysis would further benefit from (1) the addition of any missing data (wavelength palette, habitat, and/or foraging habit/diet) in the phylogeny; (2) the addition of further variables, e.g., average water depth measurements; (3) additional species data from lowly represented clades, e.g., Mbuna; and (4) further experimental testing, particularly in phenotypically divergent species pairs. Beyond the visual systems, we also identify network rewiring of genes associated with several cichlid adaptive traits like, for example, *runx2* associated with jaw morphology [54]; *ednrb1* in pigmentation and egg spots [18, 55]; and *egr1* implicated in behavioral phenotypes [56]. These also represent case studies that can be validated in species pairs that diverge for the trait of interest.

The regulatory networks generated here represent a rich scientific resource for the community, powering further molecular analysis of adaptive evolutionary traits in cichlids. As an example, further examination of the vast regulatory factors that we have predicted for the visual systems that could both up- and downregulate opsin expression diversity and could further shed light on preliminary studies of SWS1 [57], LWS, and RH2 [52] in other cichlid species*.* This could involve further functional validation to define a definitive link to trait variation by (1) high-throughput protein-DNA assays to confirm binding of hundreds of sites; (2) reporter and/or cell-based TF-perturbation assays to show that the regulatory variants indeed affect transcription; and (3) genome editing, e.g., CRISPR mutations of TFBS variants followed by phenotyping to observe trait effect. Nonetheless, this study is the first genome-wide exploration of GRN evolution in cichlids, and the computational framework (Additional file 1: Fig. S20) is largely applicable to other phylogenies to study the evolution of GRNs. In this study, we largely focus on *cis*-regulatory mechanisms of GRN rewiring. However, given the potential impact of other genetic mechanisms (protein coding changes, small RNAs, and posttranslational modifications) towards cichlid phenotypic diversity [18, 46], our framework can be extended by the inclusion of relevant datasets to allow for studies on the regulatory effect of other mechanisms, e.g., miRNAs, enhancers, and gene duplications on network topology during cichlid evolution. While many of the predicted TF-TG interactions/relationships could be false positives, our integrative approach ensured that we could apply rigorous filtering at each step, including stringent statistical significance measures, co-expression-based pruning, and all while accounting for gene node loss and mis-annotations in selected species (see “Methods”).

While it appears that cichlids utilize an array of regulatory mechanisms that are also shown to drive phenotypic diversity in other organisms [11, 39–42, 58], we provide experimental support of selected TF-TG rewiring events in regulatory regions of genes associated with adaptive traits in cichlids [18]. This is further supported by large-scale genotyping studies of the predicted sites in radiating cichlid species [20]. This potential link between GRN evolution and genes associated with adaptive trait variation in cichlids requires additional experimental verification and support by further studies on cichlid species that largely focus on large-scale genotyping [20]; whole-genome analysis and transgenesis assays [18]; behavioral and transcriptomic assays [59]; population studies and CRISPR mutant assays [60]; and transcriptomic/*cis-*regulatory assays [35, 49, 52, 57].

## Conclusions

We present a novel computational framework to study the evolution of regulatory networks in representative species of the rapid adaptive radiations of East African cichlids. Using six tissues from five species, our approach identified tissue-specific gene expression divergence between the five cichlid species that is likely associated with gene regulatory changes. As a case study, we focus on a well-studied trait—the visual system—for which we identified regulatory variation at TFBSs and demonstrate how the functional disruption of TFBSs abrogates binding of key regulators and, thus, can drive GRN evolution. Our approach revealed hundreds of novel potential regulatory regions and regulators of the five cichlid genomes, many of which have been previously associated with evolutionary traits. In conclusion, we show that regulatory network evolution can be driven by discrete changes at regulatory binding sites, and network rewiring events are likely to be a contributing source to evolutionary innovations in radiating cichlid species. This approach, with further functional validations, has the potential to identify novel genes linked to other evolutionary traits in cichlids and other evolutionary systems.

## Methods

### A comparative framework to study the evolution of tissue-specific regulatory networks in cichlids

We developed a comparative framework (Additional file 1: Fig. S20) to infer gene regulatory networks across five representative East African cichlid species—*O. niloticus* (On), *N. brichardi* (Nb), *A. burtoni* (Ab), *P. nyererei* (Pn), and *M. zebra* (Mz). Our framework comprises (1) identifying modules of co-expressed genes from multi-tissue/multi-species and single-tissue/multi-species data; (2) integrating several datasets (gene expression and *cis* regulatory regions) to reconstruct gene regulatory networks (GRNs) to find fine-grained tissue-specific network modules; (3) examining factors driving evolutionary innovation in cichlids, i.e. nucleotide divergence within regulatory binding sites and determining their mechanistic roles towards regulatory network and module divergence; and (4) using an integration of the reconstructed networks, co-expression modules, and enrichment of curated biological processes to interpret GRN evolution of genes in the context of cichlid adaptive traits.

### Inference of multi- and single-tissue transcriptional modules in five cichlids

We ran Arboretum [9], an algorithm for identifying modules of co-expressed genes on gene expression values of six tissues (brain, eye, heart, kidney, muscle, testis) from five cichlid species—*O. niloticus* (On), *N. brichardi* (Nb), *A. burtoni* (Ab), *P. nyererei* (Pn), and *M. zebra* (Mz) [18]. Tissues were isolated and RNA extracted from several adult individuals as described previously [18] and summarized here: *O. niloticus* tissues were isolated from Swansea stock individuals in the laboratory of Dr. Gideon Hulata (Volcani Center, Bet Dagan, Israel) and RNA extracted in the lab of Dr. Micha Ron (Volcani Center, Bet Dagan, Israel) using the mirVana™ miRNA Isolation Kit (Ambion); *N. brichardi* tissues were isolated from individuals inbred for ~ 10 generations in the laboratory of Prof. Walter Salzburger (University of Basel, Basel, Switzerland) and RNA extracted using TRIzol® (Invitrogen, USA); *A. burtoni* tissues were isolated from individuals inbred for ~ 60 generations in the laboratory of Dr. Hans Hoffman (University of Texas, Austin, TX, USA) and RNA extracted using TRIzol® (Invitrogen, USA); *P. nyererei* tissues were isolated from individuals inbred for ~ 5 generations in the lab of Prof. Ole Seehausen and RNA extracted using the QIAGEN RNeasy Plus Universal mini kit; *M. zebra* tissues were isolated from wild individuals in the laboratory of Dr. Karen Carleton (University of Maryland, College Park, MD, USA) and RNA extracted using the QIAGEN RNeasy Kit. In brief, the gene expression values used here were obtained from [18], and as described previously, this included (1) confirming RNA integrity on Agilent 2100 Bioanalyzer; (2) construction of RNA-seq libraries using a strand-specific dUTP protocol; (3) sequencing of RNA-seq libraries on HiSeq2000 (Illumina), yielding > 35 million 76 bp paired-end reads per tissue; (4) de novo transcriptome assembly using Trinity [61] and splice junction database from PASA gene models; (5) read alignment with TopHat2 [62]; and (6) calculating gene expression values (FPKM) with Cufflinks [63] using the protein-coding gene annotation as reference [18]. To ensure equality in *n-*fold change of expression, the gene expression values were log-transformed as: *log*(x + 1), where *x* is the raw expression value [18], and “*log*” is the natural logarithm, and then expression was normalized across each gene to have mean zero to be used as input for Arboretum [9]. The *log* expression ratio shown across modules is each gene expression relative to the mean expression across all tissues. Selection of the six tissues allowed us to study tissue-specific associated traits under natural and/or sexual selection in cichlids: brain (development, behavior and social interaction); eye (adaptive water depth/turbidity vision); heart (blood circulation and stress response); kidney (hematopoiesis and osmoregulation associated with water adaptation); muscle (size, shape, and movement associated with dimorphism and agility); and testis (sexual systems associated with behavior and dimorphism).

In total, 18,799 orthogroups, including 69,989 genes, and 34,220 1-to-1 orthologous genes (see “Cichlid gene trees”), and their associated expression data and gene tree information were inputted into Arboretum [9]. In total, this represents 59–68% of all protein-coding genes in the five cichlid genomes [18]. Certain annotated cichlid genes could not be included for a few reasons: (1) lack of tissue expression data for all five species; (2) no mapped reads for selected tissues; (3) Lack of co-expression with other genes; and (4) use of single development stage (adult). We selected the number of modules using a combination of strategies. First, we tried to identify the optimal number of multi-tissue modules *(k)* automatically from the data by scoring the Arboretum learned model based on the penalized log likelihood and silhouette index for *k* = 7–14 modules in increments of 1 (Additional file 1: Fig. S21a). This gave us *k* = 10 and 12 as the settings were local maxima for silhouette index. Second, we manually inspected the modules to see if increases of *k* yield patterns of expression that we have not seen before or generate recurring patterns (*k* = 12 is shown in Additional file 1: Fig. S21b). Based on our strategy, we found *k =* 10 modules to be optimal. Finally, we devised a metric for the top three random initializations, based on a silhouette index, orthology overlap, and cross-species cluster mean dissimilarity, selecting the optimal *k* stable to the initialization. Using a similar approach, this time for single tissues clustering, we found *k =* 5 modules to be optimal. The single-tissue modules were only initially used to assess tissue-specific gene expression divergence.

#### Handling ILS in arboretum

The Arboretum algorithm internally tries to reconcile a tree that is not obeying the species tree by adding additional duplication and loss events. An alternate approach is to use a different species trees each representing the different ILS types and estimating the parameters of each such tree. However, there are many different cases of ILS, as identified previously [18], and the number of gene trees in each category varied significantly. Estimating the conditional distributions for each branch in each ILS type would not be feasible as there are not enough example trees.

### Cichlid gene trees

By considering the gene tree of 18,799 orthologous groups (orthogroups), Arboretum [9] is able to generate module assignments reflecting many-to-many relationships between orthologs resulting from gene duplication and loss. To construct gene trees with different levels of duplication, we obtained the protein sequences of the longest transcripts from five cichlids as well as stickleback, spotted gar, and zebrafish as outgroups. Spotted gar was added as it predates the teleost-specific genome duplication event (3R) and zebrafish, as a model teleost to leverage known molecular interactions as an initial prediction of functional relationships in cichlids based on orthology. We applied OrthoMCL-1.4.0 [64] followed by TreeFix-1.1.10 [65] to learn the reconciled gene trees. We noticed that several of the trees exhibited incomplete lineage sorting (ILS) for the cichlid-specific subtree but disappeared once the tree was relearned using the cichlid only species. We therefore relearned gene trees for the cichlid only species—in total, we reconstructed 17,858 gene families of which 108 had gene duplication events. A fraction of these (29 gene families) also exhibited ILS. We also observed ILS for gene groups without gene duplications: of the 17,756 gene families that had no duplication, 810 exhibited ILS.

### Functional and transcription factor binding site (TFBS) enrichment in modules

We use the false discovery rate (FDR) corrected hypergeometric *p* value (*q-*value) test to assess enrichment of Gene Ontology (GO) terms and TFBSs (motifs) in a given gene set. In all cases, enrichment is tested using a set-based approach where a set of candidate genes is compared to a background (control set) of either all genes in species modules (18,799 orthogroups) or each genome (stated within figure legend for each test). We summarize the enrichment of terms/motifs with *q <* 0.05 statistical significance and conservation in all extant and ancestral species. GO terms for the five cichlids were from those published previously [18]. To study *cis-*regulatory elements likely driving tissue-specific expression patterns, we defined promoter regions for all genes in each of the five genomes. For this, we used the following published assemblies and associated gene annotations [18] for each species: *P. nyererei—*PunNye1.0, NCBI BioProject: ﻿PRJNA60367; BROADPN2 annotation; *M. zebra—*MetZeb1.1, NCBI BioProject: ﻿PRJNA60369﻿; BROADMZ2 annotation; *A. burtoni—*AstBur1.0, NCBI BioProject: ﻿﻿PRJNA60363; BROADAB2 annotation; *N. brichardi—*NeoBri1.0, NCBI BioProject: ﻿ ﻿PRJNA60365; BROADNB2 annotation; *O. niloticus*—Orenil1.1 (NCBI BioProject: ﻿﻿PRJNA59571; BROADON2 annotation. Gene promoter regions were defined as up to 5 kb upstream of the transcription start site (TSS) of each gene. This gene promoter region is based on analyzing the distribution of motifs in 100-nt window regions up to 20 kb upstream of each gene TSS, and observing a plateau of motifs (and distribution of CNEs) after ~ 5 kb in each species (Additional file 1: Fig. S22). Motif enrichment in *cis-*regulatory regions was carried out using TFBSs obtained by the method below, with a background (control set) of all motifs (FDR < 0.05) predicted within module gene promoters.

### Transcription factor (TF) motif scanning

TFBSs of known vertebrate transcription factors (TFs) were obtained from the JASPAR vertebrate core motif (2018 release) [66]. Binding peak information from ChIP-seq experiments of various human and mouse TFs were retrieved from GTRD v17.04 [14] and associated to protein-coding genes within a vicinity of 10 kb. Using core motif sequences available from JASPAR [66] or alternative databases like UniPROBE [67] and HOCOMOCO [68], sequences matching these motifs were identified within the TF binding peaks. In cases where the core motifs were not available for specific TFs with ChIP-seq data, they were predicted de novo from the sequences under peaks themselves using MEME [69] with default settings. The aforementioned steps provided a list of transcription factor-target gene (TF-TG) interactions with the exact coordinates of the corresponding binding site(s). Cichlid sites were extrapolated based on (1) gene-level orthology; (based on gene trees above), (2) minimum 70% sequence similarity [70, 71] between the vertebrate motif sequence and a sequence within the cichlid promoter, and (3) functional domain overlap as derived using *Interpro scan 5* [72] to both source organisms (human, mouse). Extrapolated sites from the promoters of each cichlid species were used to construct cichlid species-specific (CS) Position Specific Scoring Matrices (PSSMs) for each TF using the *info-gibbs* script from the RSAT tool suite [73]. In cases where the number of extrapolated sites per species was less than three, we aggregated the sites to construct generic cichlid-wide (CW) PSSMs. Using the PSSMs for each TF, we scanned up to 20 kb upstream of a genes TSS and conserved non-coding elements (CNEs) with FIMO [74] using either (1) an optimal calculated *p* value for each TF PSSM, calculated using the *matrix quality* script from the RSAT tool suite [73], with 1000 matrix permutations, or (2) FIMO [74] default *p* value (1e−4) for JASPAR [66] PSSMs and PSSMs for which an optimal *p* value could not be determined. Based on the distribution of motifs in 100-nt windows of up to 20 kb upstream of gene TSSs (Additional file 1: Fig. S22), we only retained motifs up to 5 kb upstream of a gene TSS as the gene promoter region (Additional file 1: Fig. S22). Statistically significant motifs were called using a *q-*value (FDR) < 0.05 and grouped in confidence levels and scores of (1a) overlap of mouse and human to cichlid extrapolated—0.3; (1b) mouse to cichlid extrapolated—0.2; (1c) human to cichlid extrapolated—0.15; (2a) FIMO [74] scans using extrapolated CS matrices—0.125; (2b) FIMO [74] scans using extrapolated CW matrices—0.110; and (2c) FIMO [74] scans using JASPAR [66] matrices—0.115. To assess whether motifs are predicted by chance, we also scanned randomized promoter sequences using the same PSSMs.

### Calculating tissue specificity index (tau)

As a measure for tissue specificity of gene expression, we calculated *τ* (Tau) [75] using log-transformed and normalized gene expression data (as inputted to run Arboretum):

$$ \tau =\frac{\sum_{i=1}^n\left(1-\hat{x_i}\right)}{n-1};\hat{x_i}=\frac{x_i}{\underset{1\le i\le n}{\max}\left({x}_i\right)} $$

Here, *n* is the number of tissues and *x*_*i*_ is the expression profile component normalized by the maximal component value [75]. The values of tau vary from 0 to 1: ubiquitous or broad expr (*τ* ≤ 0.5); intermediate expr (0.5 < *τ* < 0.9); and tissue-specific or narrow expr (*τ* ≥ 0.9) [75]. Amongst existing methods, *τ* has been shown to be a reliable method for calculating tissue specificity [76]. Testes normally express far more genes than any other tissue, generally displaying a tissue-specific pattern of expression. As tau was used to assess genome-wide expression levels across all tissues, but between species, testis expression data was included for each species to obtain a true representation of variation in transcriptional programs.

### Variation and evolutionary rate at coding and non-coding regions

We noticed several anomalous start site annotations of genes in *M. zebra*, *P. nyererei*, *A. burtoni*, and *N. brichardi* when compared to *O. niloticus.* Owing to these anomalies, we re-defined gene start sites to extract putative promoter regions. For each gene, we used the 1st exon (± 100 bp) of the longest protein-coding sequence in *O. niloticus* to identify, via BLAT-35 [77], corresponding orthologous start sites in the other four cichlid genomes. We filtered the output based on coherent overlap with original annotations [18] and orthogroups in cichlid gene trees. We re-annotated gene start sites (*M. zebra*—10,654/21,673; *P. nyererei*—10,030/20,611; *A. burtoni*—10,050/23,436; *N. brichardi*—8464/20119) based on BLAT orthology and end sites based on original annotations [18], which was otherwise used for annotating the remaining genes. Based on new annotations, for all 1:1 orthologs where gene expression data is available and there is no overlap of gene bodies, we extracted putative promoter regions, taken as up to 5 kb upstream of the transcription start site (TSS) as per methods above. Using *mafft-7.271* [78], we aligned 1:1 orthologous promoter, cds and protein sequences based on orthogrouping in gene trees (see “Cichlid gene trees”). We estimated the number of nonsynonymous substitutions per nonsynonymous site (d*N*) and synonymous substitutions per synonymous site (d*S*) in the 1:1 protein alignments using the *codeml* program in the PAML-4.9 package [79] for each branch and ancestral node in the species tree. Otherwise, we estimated evolutionary rate for each branch and ancestral node in the species tree at promoter regions and fourfold degenerate sites, using 1:1 promoter and cds alignments in *baseml* and *codeml* programs in the PAML-4.9 package [79], requiring that at least 10% of the alignment contains nucleotides and that at least 100 nucleotides are present for each species.

By using the published “*cichlid-5way.maf*” [18], we categorized pairwise substitutions for all species and intersected with annotated genomics regions (see Additional file 1: Table S2) using *bedtools-2.25.0* intersect [80].

### Reconstructing regulatory networks

To infer essential drivers of tissue-specific expression in cichlids, we constructed regulatory and functional interaction/association networks through the integration of several datasets and approaches (Additional file 1: Fig. S20). This approach was largely centered on the integration of expression-based and in silico TFBS motif prediction-based networks.

We first used species- and module-specific gene expression levels to infer an expression-based network. For this, we merged the cichlid gene expression data into a single 30 (five species, six tissues) dimensional dataset to learn cichlid-specific transcription factor (TF)-target gene (TG) interactions using the Per Gene Greedy (PGG) approach, a prior expression-based network inference method [24]. We projected the network into species-specific networks by considering edges that would not be present due to gene loss. We then integrated in silico-predicted TF-TG edges (see “Transcription factor (TF) motif scanning”) based on TFBS predictions in gene promoter regions. To ensure accurate analysis of GRN rewiring through an integrative approach, all collated edges were then pruned to ensure edges were (1) not absent in at least one species due to gene loss/poor annotation and (2) based on the presence of genes in co-expression modules.

To maintain a structured and connected network approach, we analyzed network topology using two methods; firstly, and to ensure suitable integration of co-expression data with all TF-TG predicted edges, one set of all gene nodes and their edges were constrained by Arboretum module assignments to correlate to their respective patterns of tissue-specific expression and co-expression module analysis. Secondly, since all included genes will not necessarily exhibit tissue-specific co-expression (and cluster accordingly) due to (1) differences in cell type abundance, (2) cell heterogeneity; and (3) small development stage differences, and as well as despite not being co-expressed, the fact that TFs are trans-acting factors able to regulate any gene, we also analyzed all network edges for selected candidate genes without constraining based on module assignment (co-expression). Accordingly, for candidate genes with rewired networks, we also analyzed network topology without constraining edges based on same module assignment (co-expression) and, instead, analyzed the Pearson correlation coefficient (r) between cross-species significant TF motif enrichment (FDR < 0.05), taken as −*log*(*q*-value), in all module genes and expression (zero-mean *log* expression ratio) in each tissue. Similar or dissimilar levels of TF motif enrichment were determined by calculating the variance over each TF motif enrichment, taken as −*log*(*q*-value) across the five species, and then by plotting the density distribution of the variance, categorizing TFs in each of the tails into similar or dissimilar fold enrichment (FE).

### Functional landscape of reconstructed regulatory networks

We use the FDR-corrected hypergeometric *p* value to assess enrichment of GO terms for genes in reconstructed networks. We used GO terms for the published five cichlids [18] and carried out enrichment analysis as previously done for Arboretum module genes (see “Methods” above).

### Regulatory rewiring analysis of gene sets

Regulatory rewiring of TF-TG interactions is based on predictions derived from TFBS scanning and TF-TG co-expression relationships inferred by the PGG method [24]. To ensure rewiring of TFs are correctly compared between species, and not based on gene loss/poor annotation, we only included edges for analysis where the TF had a 1-to-1 orthologous relationship in species where the TF-TG relationship or non-directed relationship exists. Also, we filtered out any TGs and their TF interaction/relationships if, based on orthologous gene *tblastx* [81], whether the gene was present in the genome but not annotated. Of the 18,799 orthogroups used for generating modules of co-expressed genes and network interactions, 4209 orthogroups had many-to-many genes actually present in the genome of at least one of the five species. These 4209 orthogroups were filtered out, retaining 843,168/1,131,812 predicted TF-TG edges across the five species; in summary, these represent edges that are (1) present in at least two species, (2) not absent in any species due to node loss or mis-annotation; and (3) based on the presence of nodes in modules of co-expression genes. The 843,168/1,131,812 predicted TF-TG edges across the five species were then used for network rewiring analysis.

Three metrics were used to study large-scale TF-TG network rewiring between species that included (1) state changes in module assignment, (2) DyNet [25] network rewiring scores and (3) TF rate of edge gain and loss in networks.

#### State changes in module assignment

In this metric, a rewired edge is where a unique TF-TG edge is present in only one “focal” species, but the TF ortholog is state changed in module assignment and is a node in other TF-TG edges in any of the other species.

#### DyNet network rewiring scores

The DyNet-2.0 package [25], implemented in Cytoscape-3.7.1 [82], was used for network visualization and calculation of a degree-corrected rewiring (*D*_*n*_) score of TF-TG interactions in each orthogroup. The *D*_*n*_ score for each orthogroup was ordered and the mean calculated; the significance of difference of each orthogroups rewiring score against all orthogroups was compared by calculating differences in the standard deviation and applying the non-parametric Kolmogorov–Smirnov test (KS-test*).*

#### TF rate of edge gain and loss in networks

Gain and loss rate analyses were similar to that performed previously [10]. This approach uses a continuous-time Markov process parameterized by TF-TG edge gain and loss rates and uses an expectation-maximization (EM)-based algorithm to estimate rates [83, 84]. The input network comprised target genes of 783 individual regulator genes mapped across the five cichlid species based on gene orthology. Each species regulator required a minimum of 25 edges as < 25 edges greatly hinder statistical analysis in this context. This resulted in a total of 345 regulators with 25 to 23,935 edges, with an average of 2609. Gain and loss rate was estimated for each regulator using the EM-based algorithm on the edge gain and loss pattern across the five cichlid phylogeny. Rates were inferred using published five cichlid branch lengths [18] that described neutral sequence evolution across the species. Stability analysis of rate estimations were carried out as follows: (1) gain and loss rate input values were scanned from 0 to 400 in intervals of 5 for each regulator matrix, and (2) from each scan, rates with the greatest likelihood were chosen as the recommended gain and loss rate (< 100), defining a final set of inferred rates for 186/345 regulators.

### Identification of segregating sites in TFBSs

Species pairwise variation was identified based on an *M. zebra* v1.1 assembly centered 8-way teleost *multiz* alignment [18]. Pairwise (single-nucleotide) variants were then overlapped with TFBS positions as determined by TF motif scanning using *bedtools-2.25.0* intersect [80]. Pairwise variants of *M. zebra* were overlapped with single-nucleotide polymorphisms (SNPs) in Lake Malawi species [20] using *bedtools-2.25.0* intersect [80]. Both sets of pairwise variants overlapping motifs and lake species SNPs were then filtered based on the presence of the same pairwise variant in orthologous promoter alignments. This ensured concordance of whole-genome alignment-derived variants with variation in orthologous promoter alignments and predicted motifs. At each step, reference and alternative allele complementation was accounted for to ensure correct overlap. This analysis was not to distinguish population differentiation due to genetic structure, but to instead map regulatory variants onto a number of radiating cichlid species to link to phylogenetic and ecological traits.

### Phylogenetic independent contrasts

Phylogenetic independent contrasts (PICs) were carried out to statistically test the effect of fitting the least controversial and all included 73 Lake Malawi species phylogeny [20] on the covariance of segregating TFBSs, visual (wavelength palette) and ecological traits (habitat and foraging habit/diet). This involved (1) categorically coding segregating TFBS genotypes (of NR2C2 > *sws1* and GATA2A > *rho*), visual trait and ecological measurements for each of the 73 Lake Malawi species (119 individuals), and (2) using the *ape* package (v5.4.1) in R (v4.0.2) to apply the PICs test [37] on all correlations with the TFBS genotypes (genotype vs wavelength palette, genotype vs habitat, and genotype vs foraging habit/diet). PICs assume a linear relationship and process of Brownian motion between traits, and thus, for each combination of data, a scatterplot was first generated. To test any change in the correlation (due to phylogenetic signal), the regression model was compared between relationships excluding and including the published Lake Malawi phylogeny [20].

### Expression of protein DNA-binding domains (DBDs)

DNA-binding domains (DBDs) of cichlid proteins (NR2C2 and RXRB) were predicted based on alignment and conservation to annotated human and mouse orthologs. *M. zebra* and *N. brichardi* individuals were sacrificed according to schedule 1 killing using overdose of MS-222 (tricaine) at The University of Hull, UK and University of Basel, Switzerland. Tissues were stored in RNA later using a 1:5 ratio. RNA was extracted from brain, liver, and testis tissues of adult *M. zebra* and *N. brichardi* using the RNeasy Plus Mini Kit (Qiagen), achieving RNA integrity (RIN) in the range of 8–10 (Agilent Bioanalyzer Total RNA Pico Assay). First-strand cDNA synthesis of DBD-specific regions was carried out using RevertAid H Minus Reverse Transcriptase (Thermo Scientific) and DBDs amplified (2-step RT-PCR) using Platinum Taq DNA Polymerase (Invitrogen) and the primers listed in Additional file 1: Table S1. Resulting cDNA was concentrated using Minelute PCR purification (Qiagen) and 700 ng used for in vitro transcription/translation using TnT T7 Quick for PCR DNA (Promega) and the Fluorotect GreenLys tRNA (Promega) labelling system. DBD expression was resolved by SDS-PAGE and detection using the fluorescein filter in the ChemiDoc Touch (Bio-Rad) system.

### Electrophoretic mobility shift assay (EMSA) validation of predicted TF-TG interactions

EMSA was carried out using double-stranded Cy5 fluorophore 5′-modified (IDT) DNA probes, in vitro expressed DBDs (see above) and the Gel Shift Assay Core System (Promega). Double-stranded DNA probes were generated by annealing sense and antisense oligonucleotides (see Additional file 1: Table S1) in annealing buffer (10 mM Tris pH 7.5, 1 mM EDTA, 50 mM NaCl) for 3 min at 96 °C, 1 min at 90 °C, 1 min at 85 °C, 3 min at 72 °C, 1 min at 65 °C, 1 min at 57 °C, 1 min at 50 °C, 3 min at 42 °C, and 3 min at 25 °C in a PCR thermocycler. Binding reactions were carried out in a final volume of 9 μl composed of Gel Shift Binding 5x Buffer (20% glycerol, 5 mM MgCl_2_, 2.5 mM EDTA, 2.5 mM DTT, 250 mM NaCl, 50 mM Tris-HCl (pH 7.5), 0.25 mg/ml poly (dI-dC)•poly (dI-dC)); 0.01 μM of Cy5-dsDNA probe covering the motif and flanking region (28 nt); and either 23 ng (RXRB, 10.42 kDa) or 27 ng (NR2C2, 10.73 kDa) of expressed DBD. For EMSA validation with increasing Nr2c2 DBD concentrations, 1× = 27 ng. For kit controls, 0.01 μM of human SP1 DNA probe was combined with 10,000 ng HeLa nuclear extract. Binding reactions were incubated at room temperature for 20 min. Protein-DNA complexes were resolved on 1 mm NuPAGE 4–12% Bis-Tris polyacrylamide gels (Invitrogen) in 0.5× TBE at 100 V for 60 min. Protein-DNA complexes were detected using the Cy5 filter on the ChemiDoc MP (Bio-Rad) system. Exposure settings were adjusted in Image Lab v6.0.1_build34 (Bio-Rad) with same high (5608), low (1152) and gamma (1.0) values set for all associated images.

## Supplementary information

**Additional file 1.** Supplementary analysis notes, figures and tables. This file includes supplementary notes, along with supplementary figs. S1-S22 and Tables S1-S3 referenced in the main text. This file also includes legends for figs and tables in Additional files 2 and 3.

**Additional file 2.** Extended data figure S1-S8. This file includes extended figures that support the findings of this study, including Fig. S1. GO enrichment of module genes (FDR < 0.05); Fig. S2. Motif enrichment of module genes (FDR < 0.05); Fig. S3. Brain heatmap pearson-correlation; Fig. S4. Eye heatmap pearson-correlation; Fig. S5. Heart heatmap pearson-correlation; Fig. S6. Kidney heatmap pearson-correlation; Fig. S7. Muscle heatmap pearson-correlation; and Fig. S8. Testis heatmap pearson-correlation.

**Additional file 3.** Large data Tables S1-S6. This file includes extended data tables that support the findings of this study.

**Additional file 4.** Review history.

## Data Availability

Cichlid PWMs that support the findings of this study are available in a figshare repository [85]. Datasets relevant to network reconstruction and their outputs are also available in figshare [86–88]. Original, uncropped gel images of EMSA experiments that support the findings of this study are available in figshare [89]. Datasets that are otherwise absent from this published article are available from the corresponding authors upon request. The source code to run motif prediction and network reconstruction from TFBS and TF-TG co-expression is freely available to all under the Creative Commons Attribution-ShareAlike licence (CC BY-SA) and under the standard GPL 3.0 licence from Github [90]. Otherwise, all other scripts relevant to this published article are available from the corresponding authors on request.
