# Supplementary material for: Evolution of regulatory networks associated with traits under selection in cichlids
Source: Genome Biol. 2021 Jan 8;22:25. doi: 10.1186/s13059-020-02208-8 (PMC7791837; doi:10.1186/s13059-020-02208-8)
Supplement: Supplementary file 2 — Additional file 2. Extended data figure S1-S8. This file includes extended figures that support the findings of this study, including Fig. S1. GO enrichment of module genes (FDR < 0.05); Fig. S2. Motif enrichment of module genes (FDR < 0.05); Fig. S3. Brain heatmap pearson-correlation; Fig. S4. Eye heatmap pearson-correlation; Fig. S5. Heart heatmap pearson-correlation; Fig. S6. Kidney heatmap pearson-correlation; Fig. S7. Muscle heatmap pearson-correlation; and Fig. S8. Testis heatmap pearson-correlation. [file 13059_2020_2208_MOESM2_ESM.pdf]

Additional file 2: Fig. S1
Gene Ontology (GO) enrichment of modules across all extant and ancestral species

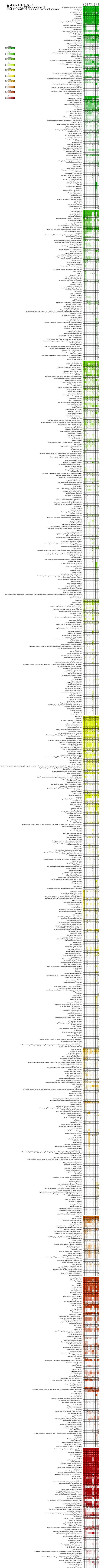

Additional file 2: Fig. S2

Transcription factor motif enrichment of module gene promoters across all extant and ancestral species.

Cluster0				
1.3	2.2	3.2	4.1	5.0

Cluster1				
1.3	2.2	3.2	4.1	5.0

Cluster2				
1.3	2.2	3.2	4.1	5.0

Cluster3				
1.3	2.2	3.2	4.1	5.0

Cluster4				
1.3	2.2	3.2	4.1	5.0

Cluster5				
1.3	2.2	3.2	4.1	5.0

	Meze	Puny	Anc1	Asbu	Anc2	Nebr	Anc3	Orni	Anc4
ALX1:1	2.9	2.3	1.9	1.3				2.4	
ALX3:1	2.6	4.0	4.2	3.9	3.7	1.6	2.1		2.5
ALX4:1	3.1	3.4	1.7	1.5	1.3			2.2	
ARID3B:1	1.7	2.9						1.8	
ARID5A:1	2.5	2.5	1.5	2.2	1.3			1.8	1.6
ARX:1	2.6	2.9	2.3	2.3	2.0				
ASCL1:1	1.8	3.1	2.4	2.3	1.7			1.9	
ASCL2:1	2.3	1.8	1.9	1.7				2.3	
ATOH1:1	3.4	3.0	3.3	2.7	2.6	2.6	2.6	3.4	3.4
BARHL1:1	2.3	2.3	2.8	3.0	2.6		2.0	1.7	2.8
BATF::JUN:1	2.5	3.5	2.1	1.9	1.4			2.3	1.5
BCL6:1	1.8	4.0	2.9	2.2	2.4			3.2	2.6
BCL6B:1	1.8	2.6	2.5	3.0	2.7		2.3	5.7	7.3
BHLHA15:1	3.6	3.4	4.4	4.6	4.3		3.4	2.8	2.6
BHLHE22:1	3.3	1.7	2.3	1.6	1.8	2.5	2.0	1.7	1.5
BHLHE23:1	2.5	1.7	1.8	2.0				1.9	
CDX1:1	2.1	2.5	2.6	2.1	1.8		1.5	1.4	2.1
CDX2:1	3.1	3.9	4.7	3.3	4.3		3.8	3.2	3.4
CEBPA:1	2.0	1.9	2.1	2.3	1.9			2.4	
CREB5:1	1.5	2.1	1.8	1.9	1.4			1.6	1.6
CRX:1	9.5	10.7	10.9	9.1	11.3	7.4	10.2	4.2	9.6
CTCF:1	1.6	2.1	1.8	1.7	1.6				1.6
DBP:1	2.6	3.6	2.0	3.4	1.8			1.7	
DDIT3::CEBPA:1	2.4	1.5	1.7	2.2	2.0			1.9	1.8
DLX1:1	1.4	1.5	1.5	1.6	1.5				
DLX4:1	2.3	2.6	1.8	2.7	1.5				
DLX6:1	1.3	2.0		2.2		2.0			

Cluster6

1.3	2.2	3.2	4.1	5.0

Cluster7

1.3	2.2	3.2	4.1	5.0

Cluster8

1.3	2.2	3.2	4.1	5.0

Cluster9

1.3	2.2	3.2	4.1	5.0

DMBX1:1	3.6	6.3	4.3	4.9	4.7	3.0	3.8	3.0	3.1
DMRT3:1	3.0	2.6	2.0	2.5	2.3		1.6	2.0	1.9
DUX:1	1.6	2.5	2.2	2.1	1.5			1.8	1.6
DUX4:1	1.9	2.5	2.1	2.0	1.9			2.4	1.3
DUXA:1	2.4	3.2	1.8	1.9				2.4	
E2F6:1	2.0	1.3	2.2	2.0	1.6		1.4	3.6	3.5
EMX1:1	2.0	2.4	1.9			2.1			1.3
EMX2:1	1.6	3.2	1.6	1.4					
EN1:1	1.6	2.1	1.8		1.6		1.5	1.6	1.7
EOMES:1	2.6	1.7	1.9	2.1	1.3			2.5	1.9
ESR2:1	3.2	3.4	3.8	3.6	3.5	2.1	3.0	2.6	2.8
ESRRA:1	2.1		2.4	1.9	1.4			1.8	1.4
ESRRB:1	1.3	3.0	1.7	1.4				1.3	
ESX1:1	1.8	2.8	2.2	2.1	1.9				1.7
EWSR1-FLI1:1	2.7	2.5	3.6	4.3	3.5		2.5	2.0	3.6
FIGLA:1	2.2	4.0	3.1	2.8	1.8			2.9	2.9
FOSL2::JUN(VAR.2):1	1.4	2.7	1.7	2.1	1.3			2.4	1.6
FOXA1:1	1.8	2.9	1.7	2.2				1.7	2.5
FOXA2:1	3.2	3.3	1.7	3.4	1.8			1.3	2.6
FOXB1:1	2.0	3.9	1.5	1.7					
FOXC1:1	3.2	4.6	2.6	2.3	1.6			1.5	1.6
FOXC2:1	2.4	2.8	2.0	1.8				2.0	2.0
FOXD1:1	1.6			1.4				1.8	1.5
FOXD2:1	1.6			1.4				1.8	1.5
FOXD3:1	2.5	1.9		1.4				2.1	
FOXF2:1	3.0	1.8	1.9	2.1	1.9		1.7		1.6
FOXG1:1	2.3		1.5	2.0				1.7	1.6
FOXH1:1	2.5	3.1	1.4	1.7	1.5			3.0	2.2
FOXI1:1	1.6			1.4				1.8	1.5
FOXJ2:1	2.4	1.4		2.3					1.4
FOXJ3:1	3.5	2.5	2.1	2.0	1.8		1.7		1.5

FOXK1:1	1.5	1.3		1.5				1.8	1.8
FOXK2:1	2.3	1.3		1.6					
FOXL1:1	1.6			1.4				1.8	1.5
FOXO1:1	3.7	2.6	2.1	1.6	1.4		1.4	4.0	1.6
FOXO3:1	1.7			1.6				1.7	1.6
FOXO4:1	1.6			1.4				1.8	1.5
FOXO6:1	1.6			1.4				1.8	1.5
FOXP1:1	3.4	2.8	2.6	2.7	2.2		1.8	2.2	2.2
FOXP2:1	3.6	2.9	2.2	2.1	1.7		1.4	1.7	1.9
FOXP3:1	1.6			1.4				1.8	1.5
FOXQ1:1	2.2	2.5	1.4	2.9					
GATA1:1	1.7	1.9	2.2	2.2	2.1		1.8	1.9	2.7
GATA1::TAL1:1	2.8	1.7		2.1				1.6	
GATA2:1	1.6			1.7					
GATA3:1	1.3							1.6	
GATA4:1	2.3	3.0	2.4	1.9	1.9			1.8	1.8
GATA5:1	1.3							1.6	1.7
GATA6:1	1.4							1.9	1.9
GBX1:1	1.7	1.8	2.0	1.3	1.9		1.4		1.7
GBX2:1	2.0	2.9	2.7	1.5	1.9				2.2
GFI1:1	4.3	5.0	4.7	4.7	4.2	3.0	3.4	2.8	2.3
GFI1B:1	3.0	4.6	2.7	3.7	2.3			4.1	2.3
GRHL2:1	2.0	1.6			1.4			3.3	2.8
GSC:1	7.6	9.0	12.9	12.4	13.2	9.7	13.5	4.4	7.3
GSC2:1	8.3	9.1	10.9	10.1	10.7	5.8	10.2	4.2	6.8
GSX1:1	2.7	3.6	2.0	1.6	1.7				
GSX2:1	3.2	3.1	2.7	1.9	2.6		1.7	2.4	2.1

HAND1::TCF3:1	1.5	2.9	2.6	1.7	1.6		1.4		2.0
HLF:1	3.5	3.6	2.4	3.8	2.3		1.3	1.8	
HNF1A:1	2.2	2.9	1.8						
HNF1B:1	2.7	4.0	2.4	1.4	1.5				
HNF4A:1	1.8	3.5	1.7	1.7				1.9	2.3
HNF4G:1	2.6	6.0	3.8	1.6	2.4	1.4	1.9	3.0	3.6
HOXA13:1	2.1	1.4	1.4	2.0	1.4			1.6	
HOXA2:1	1.6	2.0	1.9		1.8		1.5		1.7
HOXA5:1	2.7	2.3		1.7				1.7	1.6
HOXA9:1	2.1	1.5		2.5	1.7		1.4		
HOXB5:1	2.3	3.6	2.1	2.0	2.0	2.0	2.1		
HOXC9:1	1.8	1.9	1.4	2.0				1.8	
HOXD13:1	1.5	1.8	2.2	1.4	1.4				
HOXD3:1	3.6	3.5	2.7	2.3	2.1	1.9	1.7	1.8	
HOXD8:1	1.7	3.4	2.1	1.6	1.5				
HOXD9:1	1.8	2.4	3.1	2.7	2.1		1.5	1.5	2.1
HSF2:1	1.4		1.3	2.3	1.5				
ID4:1	2.5	4.0	2.3	2.2	1.6			2.2	1.6
INSM1:1	1.8	2.8	2.4					1.8	
IRF1:1	2.6	3.6	2.8	2.1	2.3		2.5	3.0	3.0
IRF7:1	1.3	2.5	1.7					1.7	1.6
ISX:1	3.2	1.4	2.6	2.3	2.3		1.8		2.4
JUN:1	2.6	3.6	3.1	2.3	2.2		2.0	3.0	2.1
JUND(VAR.2):1	2.9	5.1	3.0	4.9	2.5		2.0	2.3	1.8
KLF5:1	1.3	3.2	2.2	3.8	2.3		1.8	4.1	3.5
KLF9:1	2.0	3.5	3.3	2.5	2.2			3.2	3.6
LBX2:1	2.1	1.7	2.6	2.4	2.3		1.8		2.8

LEF1:1	3.7	6.2	5.9	3.3	3.7		2.7	3.7	3.5
LHX2:1	2.4	1.6	2.2	1.4	1.7				
LHX3:1	1.8	2.9	1.8	2.3			1.3		
LHX4:1	3.1	2.9	2.1		1.5				
LHX6:1	1.5	1.9		1.3					
LHX8:1	2.0	2.6	2.1	1.8	1.8				
LHX9:1	3.2	1.4	2.6	2.3	2.3		1.8		2.4
LIN54:1	2.1	1.8	1.8	1.8	1.4			2.1	2.1
LMX1A:1	2.1	3.2	2.1	1.8	1.8				
LMX1B:1	2.1	3.2	2.1	1.8	1.8				
MAFB:1	1.4	2.9	1.9		1.7			2.4	2.8
MAFF:1	2.3	3.2	2.2	1.8	1.7			3.1	2.9
MAFG:1	3.0	3.6	2.4	2.9	2.5		1.8	2.8	3.0
MAFK:1	2.0	1.9	1.8	2.5	1.9		1.5	2.9	3.0
MECOM:1	3.2	3.0	2.5	1.7	1.9			1.8	1.6
MEF2A:1	3.4	5.4	2.5	3.8					
MEF2B:1	1.5			1.6					
MEF2C:1	4.0	6.0	2.3	3.2	1.7		1.4	2.4	1.7
MEF2D:1	1.8	3.7	1.3	2.1				1.6	
MEIS3:1	1.4	2.7		1.4				1.5	2.2
MEOX1:1	2.6	4.0	4.1	2.0	3.3	1.5	3.2	1.3	2.1
MEOX2:1	1.8	3.6	2.3	2.0	1.7		1.6	1.4	1.5
MIX-A:1	2.3	2.2	2.1		1.5				
MIXL1:1	2.3	2.3	2.8	1.9	1.9				2.2
MNX1:1	1.6	2.4	2.6	1.8	2.0			1.8	
MSC:1	1.3		1.6	1.3				1.8	
MSX1:1	1.3	1.9	1.6	2.8	1.5			1.6	2.5
MYB:1	1.3	2.1		2.0				1.3	
MYOG:1	1.5	2.8	2.4				1.4	2.4	1.6
MZF1(VAR.2):1	1.7	5.7	3.1	3.2	2.3		1.7	3.0	2.4

NEUROD1:1	3.4	2.9	4.5	3.6	3.6		3.5	3.7	3.8
NEUROD2:1	2.8	2.6	2.4	2.9	2.1	1.6	2.1		1.4
NEUROG1:1	2.9		1.7	1.6	1.3				
NEUROG2:1	3.1	3.0	3.1	3.0	2.6		2.3	3.8	3.1
NFAT5:1	1.8	4.5	3.4	1.5	2.3		1.8	2.4	2.4
NFATC1:1	1.4	3.1	2.9	2.0	2.2			1.6	1.6
NFATC2:1	2.3	5.4	3.3	3.1	2.5	2.2	2.6	3.4	3.0
NFATC3:1	1.6	2.9	2.9	2.2	2.3			1.8	1.7
NFIL3:1	1.8	2.8		2.4				1.9	
NKX2-3:1	1.8	2.6	2.1	1.9	2.0			1.8	
NKX2-8:1	2.4	3.7	2.3	1.4	1.5			2.1	1.5
NKX3-1:1	1.9	2.2	1.7	1.7				2.0	1.6
NKX3-2:1	1.7	1.9		1.3				1.4	
NKX6-1:1	3.0	4.0	2.8		2.1		1.6		
NKX6-2:1	2.6	3.6	3.3	2.1	2.9	1.6	2.1	1.8	2.1
NOBOX:1	1.7	2.6	2.2	2.3	1.9		1.8		1.6
NOTO:1	3.0	2.7	2.3	1.7	1.9	1.4			
NR1H2::RXRA:1	2.4	3.2	2.6	3.9	2.2		1.4	2.7	2.1
NR1H3::RXRA:1	2.2	1.5	2.4	2.2	1.9			2.8	2.4
NR1H4:1	2.3	2.6	1.6	2.5	1.4			2.6	1.6
NR2C2:1	1.6	4.0	2.7	2.4	2.1		1.5	4.5	3.8
NR2E1:1	2.6	2.0	2.0	2.2	1.7			1.3	
NR2E3:1	1.8	3.5	2.7	2.9	2.6		1.6	2.2	2.8
NR2F1:1	4.1	2.1	3.5	3.3	3.5		2.7	3.2	3.1
NR2F2:1	2.7	2.5	2.5	2.1	1.9		1.5	4.4	2.5
NR2F6:1	3.0	2.6	3.0	2.4	3.0		1.6	3.0	2.9
NR2F6(VAR.2):1	2.6	3.5	3.3	3.8	3.6		3.5	4.4	4.4

NR4A1:1	3.7	3.2	3.3	3.3	3.7		2.6	3.3	3.4
NR4A2:1	2.0	3.3	2.8	2.9	3.1		2.1	3.5	3.5
NR5A2:1	1.3	1.4	1.9	1.8	1.4			2.5	2.1
OLIG1:1	3.2	2.4	2.2	2.3	1.9			1.4	1.4
OLIG2:1	3.0	3.0	2.4	2.5	1.9				
OLIG3:1	3.3	2.7	2.5	3.4	1.9	1.8		1.5	1.5
OTX1:1	5.6	4.2	6.5	6.3	5.6	4.2	6.6	2.0	4.0
OTX2:1	4.0	6.4	9.2	7.9	8.9	4.2	8.8	2.0	4.4
PAX3:1	2.1	3.3	2.2	1.6	1.7			1.6	
PAX4:1	2.3		1.8	1.6	1.6				
PAX6:1	1.3	1.9	2.1	1.7	1.4			2.5	1.7
PAX7:1	3.4	4.1	3.2	2.3	2.3		1.3	2.0	
PBX1:1	1.8	2.1		3.0	1.9				1.5
PBX2:1	2.4	1.9	1.5	3.0	1.4			2.8	2.3
PBX3:1	2.3	2.8	1.6	3.0	1.5			3.1	2.8
PDX1:1	1.5	2.8	1.8	1.5	1.5			1.8	1.7
PHOX2A:1	3.5	3.8	2.5	2.4	1.8		1.7		
PHOX2B:1	2.7	3.7	2.9	2.3	1.9		1.6		1.3
PITX1:1	8.2	7.5	8.6	6.5	5.7	2.4	5.3	3.2	4.5
PITX3:1	5.4	6.8	8.0	9.4	7.4	3.3	6.4	3.4	5.1
PKNOX1:1	1.4			1.8				2.0	1.4
POU1F1:1	3.1	2.3	1.7		1.4				
POU2F1:1	1.9	2.1		1.3					
POU2F2:1	2.7	3.8	2.3	2.0	1.7		1.7	1.7	1.7
POU2F3:1	2.6	3.5	2.1		1.8		1.7	1.4	
POU3F1:1	1.9	1.7		1.6				1.5	
POU3F2:1	3.2	2.0	1.8	2.5	1.7				
POU3F3:1	3.3	2.6	2.1	3.0	2.1		1.5		

POU3F4:1	1.4	1.8							
POU4F1:1	1.9	3.6	2.3	1.7	1.6				
POU4F2:1	2.1	3.7	2.6	1.7	1.8			1.7	
POU4F3:1	1.9	3.2	2.2	2.0	1.5				
POU5F1:1	2.5	3.1	1.9	1.8	1.7	2.3	1.4		1.6
POU5F1::SOX2:1	2.1	2.3	2.0	3.0	2.1		2.1	2.4	2.1
POU5F1B:1	2.0	1.9							
POU6F1:1	1.5	1.9	1.3						
POU6F2:1	4.4	3.7	3.8	2.2	3.7	1.8	2.6	1.7	1.8
PPARA::RXRA:1	1.7	2.9		1.6				2.2	
PPARG:1	2.3	2.2	3.3	2.2	2.6		1.8	1.8	2.2
PPARG::RXRA:1	2.3	3.2	2.4	3.1	1.4			2.1	1.7
PRDM1:1	2.9	1.8	1.4	2.0				3.3	1.6
PROP1:1	2.4	3.4	1.9	1.6			1.5		
PRRX1:1	3.2	1.4	2.6	2.3	2.3		1.8		2.4
PRRX2:1	1.8		1.6	2.7				1.5	2.2
RARA:1	3.2	3.9	2.7	3.4	3.0		1.9	2.3	2.6
RARA::RXRG:1	1.4	2.9	3.0	1.8	2.3		1.6	2.2	2.2
RARB:1	2.6	2.2	2.6	4.3	2.5		1.8	3.8	2.8
RARB(VAR.2):1	2.4	1.9	2.5	2.3	2.3		1.4	3.3	2.3
RARG:1	1.5	1.6	2.0	2.5	2.4		1.7	4.2	3.6
RARG(VAR.2):1	1.9	1.7		1.7	1.5			2.0	2.1
RAX:1	1.8	1.6	1.7	1.6	1.4				
RAX2:1	3.2	1.4	2.6	2.3	2.3		1.8		2.4
RBPJ:1	2.8	3.7	2.6	1.5	2.0		1.4		2.0
RFX5:1	1.5			1.4					
RHOX11:1	2.1	1.9		2.0				4.5	3.0
RHOXF1:1	5.1	7.5	8.1	9.5	8.5	4.6	8.8	3.7	6.4
RORA:1	2.9	1.9	3.1	3.0	3.4		1.6	2.7	2.3
RORA(VAR.2):1	4.3	4.2	2.6	2.5	2.4	2.0	1.9	3.0	2.7
RORB:1	2.4	2.2	2.3	1.9	1.9			1.7	1.6

RORC:1	4.6	4.6	4.7	2.9	3.9		2.6	3.0	3.6
RREB1:1	2.5	3.8	1.6	2.8	1.4		1.5	4.1	3.6
RUNX1:1	2.8	2.9	1.3					3.9	2.6
RUNX2:1	1.9			1.7				2.6	2.5
RUNX3:1	2.6	2.1	1.5					3.7	3.4
RXRA:1	3.3	2.7	3.0	3.3	2.6		1.8	1.9	1.9
RXRA::VDR:1	2.9	4.0	3.3	2.3	2.1		1.4	3.2	3.1
RXRB:1	3.2	2.9	3.4	3.8	2.9		1.9	2.4	2.1
RXRG:1	2.3	2.8	2.4	3.4	2.1		1.9	3.5	3.0
SCRT1:1	1.9	2.9	3.6	4.3	3.3	2.9	2.7	4.2	5.5
SCRT2:1	2.3	1.4	2.1	2.1	1.8			3.9	3.3
SHOX:1	3.2	1.4	2.6	2.3	2.3		1.8		2.4
SHOX2:1	3.2	1.4	2.6	2.3	2.3		1.8		2.4
SIX1:1	1.6	2.5	1.8	1.9				2.8	1.5
SMAD2::SMAD3::SMAD4:1	1.7	3.1	2.7	1.5	1.8		1.6	3.7	2.5
SMAD4:1	1.8		2.7	1.6	1.9		1.7	1.6	1.8
SNAI2:1	2.4	3.1	2.3	2.3	1.6			3.7	2.7
SOX10:1	2.8	3.2	2.0	1.4	1.4			1.8	1.6
SOX11:1	3.0	3.9	3.8	4.9	3.7		2.7	2.3	2.8
SOX13:1	2.3	3.0	1.9	2.9	1.6	1.6	1.5	1.8	
SOX15:1	3.1	2.8	2.0	1.4	1.5	2.1			
SOX17:1	2.4	2.8	1.6	2.6	1.6			1.8	
SOX2:1	1.7	2.4	1.4	2.1	1.3				
SOX3:1	2.8	4.2	4.9	3.4	4.3	3.0	3.0	2.2	2.8
SOX4:1	3.4	3.3	3.8	3.8	3.2		2.3	3.4	1.9
SOX6:1	3.2	3.9	3.4	2.8	2.9	3.1	2.7	2.2	2.2
SOX8:1	4.4	5.4	3.4	4.7	3.7		2.7	2.8	2.4
SOX9:1	2.4	3.8	2.5	2.6	2.6	1.8	1.9		

SP1:1	1.8	2.3	2.3	1.8	1.9			6.7	4.3
SP2:1	1.5	1.9	2.4	2.3	1.9		2.5	9.0	6.6
SREBF1:1	1.3	1.4		1.4					1.4
SREBF2:1	2.1	2.4	1.8	1.4	1.5			1.8	
SRF:1	1.4			1.8					
SRY:1	2.1	2.5	1.4	1.7					
STAT1:1	1.3	2.9	1.4	1.7	1.4			3.0	
STAT1::STAT2:1	1.6	2.8	2.1		1.5		1.4	2.0	2.1
STAT3:1	2.3	4.0	2.4	1.7	1.5			4.2	3.0
STAT4:1	2.7	2.9	2.8	2.3	2.9		1.5	4.1	3.0
STAT5A::STAT5B:1	1.4	1.8	2.6	2.2	1.9		1.4	2.7	2.0
TAL1::TCF3:1	2.3	1.9	1.9	2.6				3.4	2.8
TBP:1	2.3	2.5	2.1	1.9	2.0	1.8	2.1	1.5	2.1
TBR1:1	2.6	1.4	1.5	1.7				2.1	2.3
TBX1:1	2.6	3.0	2.5	3.1	2.6		1.6	2.8	3.4
TBX15:1	1.8	1.7	1.9	2.1	2.1		1.7	1.9	2.5
TBX2:1	2.0	1.9	1.5					2.6	2.6
TBX20:1	2.5	2.5	2.0	2.1				1.7	2.3
TBX21:1	2.0	1.9	1.7	1.8	1.5			2.7	2.0
TBX4:1	2.6	3.0	2.5	3.1	2.6		1.6	2.8	3.4
TBX5:1	1.5	2.8	2.2	3.3	2.2		1.6	2.3	2.1
TCF12:1	1.6	2.9	2.5	1.4	1.6		1.7	3.2	2.0
TCF3:1	2.3	3.5	1.8	2.5				2.6	2.4
TCF4:1	2.1	3.1	2.0	2.4	1.4			3.0	2.6
TCF7:1	3.3	4.6	4.2	3.8	3.1		1.9	2.9	2.2
TCF7L1:1	2.6	3.1	4.1	2.5	2.8		1.6	2.6	1.7
TCF7L2:1	2.6	3.0	2.9	2.7	2.2			2.2	1.9
TEF:1	2.0	4.5	2.0	3.4	1.6			2.3	

TFAP4:1	1.9		1.3					3.4	1.6
THAP1:1	1.8	1.8		1.9				1.8	1.5
TP53:1	1.5	2.5	2.5	2.2	2.1				1.4
TWIST1:1	2.2	2.6	3.0	2.3	2.4		2.7	4.4	4.0
TWIST2:1	3.2	3.0	2.6	3.8	2.2		1.7	2.4	1.6
UNCX:1	3.2	1.4	2.6	2.3	2.3		1.8		2.4
USF1:1	1.5	2.4	1.7	1.8	1.9				1.6
VAX2:1	1.8	3.7	1.8		1.3			1.8	1.5
VDR:1	2.5	4.0	1.8	3.9	1.8			2.3	1.6
ZBTB18:1	3.5	4.3	3.9	2.8	2.9		3.0	3.8	3.1
ZBTB7B:1	1.7	1.3	1.3	1.7	1.3				1.5
ZBTB7C:1	1.8	1.4	1.5	2.1					
ZIC4:1	1.6	1.4	2.2	3.3	1.8			1.5	
ZNF24:1	3.7	3.4	2.5	2.4	1.7		1.7	3.4	2.6
ZNF263:1	3.2	4.6	4.9	3.6	4.6		3.6	3.9	3.5
ZNF384:1	1.4	2.9	1.8		1.5		1.5	1.7	1.5
ZNF740:1	1.4	2.4	1.8	1.4	1.5			2.4	1.7
ZSCAN4:1	4.6	3.8	4.0	3.4	2.9		2.2	2.4	2.5
AR:1		1.9		1.4					
ARNT::HIF1A:1		1.4							
ATF4:1		1.4	1.8		1.3			2.0	
ATF7:1		1.5						2.0	
BACH1::MAFK:1		2.7		1.5				3.3	2.6
BACH2:1		3.4	2.2	2.2	1.6			4.5	3.3
BATF3:1		2.1	1.8	1.3					
BSX:1		1.9	1.9	2.3	1.6				2.2
CLOCK:1		1.5		1.4				1.7	
CREB1:1		2.1	2.2	2.4	1.7		1.5	2.5	2.2
CUX1:1		1.6							
DLX2:1		2.1	1.8	1.6			1.7		
DLX3:1		1.4		2.5					

EGR2:1		1.5						1.5
EGR3:1		1.5					1.4	1.4
EGR4:1		2.3		1.9			3.0	3.8
ESR1:1		1.4	1.7	2.2	1.8	1.6	1.7	2.5
ESRRG:1		1.5	1.4	2.0	1.3			
EVX1:1		1.8						
EVX2:1		1.9						1.3
FOS::JUN(VAR.2):1		1.6	1.4	1.6				
FOS::JUND:1		1.7					3.3	3.0
FOSL1::JUND:1		1.5					3.4	2.2
FOSL2:1		2.0					2.6	1.8
FOSL2::JUN:1		1.5					4.7	3.6
FOSL2::JUNB(VAR.2):1		1.4					1.4	1.3
FOSL2::JUND(VAR.2):1		3.3	2.1	2.1	1.8	1.5	2.3	1.9
GCM1:1		1.9	1.3		1.7			
GCM2:1		1.4		1.4				
GRHL1:1		1.7	1.4		1.5		2.5	2.3
HES7:1		1.5						
HLTF:1		1.5					1.4	
HMBOX1:1		1.7						
HMX1:1		2.3	1.7		1.4			
HMX2:1		1.7	1.6				1.8	1.6
HMX3:1		1.7	1.4					
HOXA10:1		2.1		1.8				
HOXB2:1		1.7	1.6					
HOXB3:1		1.8	1.7	1.6			1.8	
HOXC10:1		1.3	1.8	1.5	1.5		1.4	
HOXC13:1		1.3	1.3					
HSF1:1		1.7	1.9		1.4			
IRF2:1		2.6	2.2	1.7			1.9	2.2

IRF3:1		2.5							1.6
IRF4:1		1.7							
IRF8:1		3.0	2.3		1.8		1.8	3.0	1.6
IRF9:1		2.2	1.9					1.8	
ISL2:1		3.1	2.0	1.7	1.5				
JDP2:1		1.7						1.9	1.4
JDP2(VAR.2):1		2.1	1.6	1.8				2.0	2.2
JUN::JUNB:1		1.5						1.7	1.3
JUN::JUNB(VAR.2):1		1.4						2.2	
JUNB:1		1.9						2.7	1.9
JUND:1		1.3	1.4					4.6	3.5
KLF1:1		2.1		1.6					
KLF12:1		1.6		1.4					
KLF16:1		1.5	1.3					2.0	1.4
KLF4:1		1.9		3.0					
LBX1:1		1.4							
MAF::NFE2:1		3.4	1.8	1.7	1.4		1.5	3.0	3.1
MAX::MYC:1		1.9	1.4		1.3				
MEIS1:1		2.9		1.6					1.3
MEIS2:1		2.7		1.8				1.9	3.0
MITF:1		1.5							
MSX2:1		1.9	1.9	1.8	1.8		1.3	1.8	2.9
MSX3:1		1.9	1.9	1.8	1.8		1.3	1.8	2.9
MTF1:1		3.9						2.9	
MYF6:1		2.5	2.1	1.5	1.9		1.5	2.4	1.9
MYOD1:1		2.7	1.6					3.3	1.4
NFE2L2:1		2.2	1.3					3.2	2.4
NFIC:1		2.1						2.2	2.0
NFYA:1		1.3		1.8					
NHLH1:1		1.4							

NKX2-5:1		2.1	1.9	1.9	1.6				2.1
NKX2-5(VAR.2):1		3.6	1.6					1.9	
NR3C1:1		2.6							1.3
NR3C2:1		2.8		1.4	1.4				
NRL:1		2.4	2.2	1.7	2.0			2.6	3.6
ONECUT3:1		1.8		1.7				1.9	1.6
PAX9:1		1.5							
PKNOX2:1		1.6		2.3				2.6	1.6
PLAG1:1		1.9	1.9	2.9	1.9			1.3	1.8
RARA(VAR.2):1		2.2	1.4					1.8	
RARA::RXRA:1		2.5	1.7					3.5	2.4
RELB:1		1.5	1.5		1.4			2.0	1.4
RFX1:1		2.3	1.6	1.3				2.6	2.0
SIX2:1		1.7		1.9				1.9	
SIX3:1		2.1	1.4	1.7					
SOX1:1		1.5						1.8	
SOX21:1		3.6	1.9	2.2	1.4			2.6	
SPIC:1		1.4						3.2	2.3
SPZ1:1		2.2						1.4	1.8
SREBF1(VAR.2):1		2.1							
SREBF2(VAR.2):1		1.8							
STAT6:1		2.0		1.5				2.4	1.8
T:1		1.9						1.5	
TCF21:1		1.6						2.4	1.8
TEAD2:1		1.6						2.1	1.6
TEAD4:1		1.4						3.0	
TFAP2A:1		1.7		1.7					1.4
TFAP2A(VAR.2):1		1.7						1.6	
TFAP2A(VAR.3):1		1.3	1.8					1.6	1.6
TFAP2B:1		1.7		2.5				1.7	

TFAP2B(VAR.3):1		1.4	1.5					1.9	1.8
TFAP2C:1		1.4		1.5					
TFAP2C(VAR.3):1		1.4						2.3	2.3
TFCP2:1		1.4						1.7	
TFEB:1		1.6						1.6	
TFEC:1		2.0			1.4			1.6	1.8
TGIF2:1		1.6		1.6				2.5	1.6
TP73:1		1.5	1.3					1.5	
VAX1:1		1.9							
ZEB1:1		3.2		2.1				3.0	1.4
ZFX:1		1.9						1.6	1.6
ZNF143:1		1.7		1.6				2.4	
ZNF282:1		2.0		1.7				1.5	
ZNF410:1		2.0		1.4					
ZNF423:1		1.4		1.4					1.7
EGR1:1			1.5		1.4			2.6	1.8
EN2:1			2.0	2.1	1.9		1.5	1.8	2.2
FOSL1::JUN:1			1.4					3.5	3.2
GLIS3:1			1.8	2.0	1.7		1.5	2.2	2.1
HOXD11:1			1.6						
HSF4:1			1.4	1.4					
JUN(VAR.2):1			1.3				1.4	4.5	3.4
MGA:1			1.6	1.7				2.2	2.1
NFKB1:1			1.4	1.5					
VSX1:1			1.3						
VSX2:1			1.3						
ATF3:1				1.3					
CEBPE:1				1.3				1.4	
CREB3L1:1				1.6					
CTCFL:1				1.8					1.6

E2F8:1				2.1					2.2
HOXB13:1				1.5					
NFKB2:1				1.5				1.3	2.0
NFYB:1				1.4				1.3	1.7
SP3:1				1.5				1.9	2.2
TFAP2C(VAR.2):1				1.7					
TGIF1:1				1.7				2.8	2.1
ZIC3:1				2.7				1.5	
RFX3:1					1.4			1.4	
ATF1:1								1.9	2.1
CEBPB:1								1.5	
CEBPD:1								1.5	
CEBPG:1								1.5	
E2F2:1								1.5	
FOS:1								3.9	3.3
FOS::JUN:1								3.8	2.7
FOS::JUNB:1								2.3	
FOSB::JUN:1								1.8	
FOSB::JUNB:1								3.4	2.3
FOSB::JUNB(VAR.2):1								1.5	
FOSL1:1								4.2	3.4
FOSL1::JUNB:1								3.3	2.0
FOSL2::JUNB:1								3.5	2.4
FOSL2::JUND:1								2.7	1.8
GLI2:1								1.8	
HES2:1								1.5	1.4
HIC1:1								1.4	
HOXA11:1								1.5	
JUNB(VAR.2):1								1.8	1.4
KLF13:1								1.4	

NFE2:1								1.6	1.8
NFIX:1								1.4	
ONECUT1:1								2.0	
ONECUT2:1								1.6	
PAX1:1								1.5	1.3
PAX2:1								1.8	
PAX5:1								1.9	
REL:1								1.5	1.4
RELA:1								2.2	1.4
SMAD3:1								1.8	
SP4:1								2.2	1.7
SP8:1								1.9	
SPI1:1								1.8	
TBX19:1								1.9	1.5
TEAD1:1								2.4	
TEAD3:1								2.4	
TP63:1								1.4	
ZIC1:1								1.9	1.4
EBF1:1									1.4

ARNTL:2	1.8	1.7		2.2					
ASCL1:2	2.3	3.4	4.2	4.2	3.2		2.1		
ASCL2:2	5.4	7.0	5.6	5.6	4.5	1.4	2.4		2.4
ATF1:2	1.6	3.3	1.8	1.7	1.4				
ATF4:2	1.7	3.9	2.5	2.3	2.6	1.3	1.7		
BACH1::MAFK:2	3.0	5.0	4.4	2.8	4.7	1.4	2.9	1.6	2.3
BACH2:2	2.8	5.1	4.5	2.5	5.2	2.5	3.5		2.4
BATF::JUN:2	3.2	5.8	5.2	3.4	4.7	2.5	3.4		2.7
BCL6:2	2.8	5.1	4.1	4.1	4.1	2.3	3.0	1.4	2.5

BCL6B:2	4.2	4.6	5.1	3.4	5.1		3.3		1.9
BHLHE23:2	1.3	1.7							
CDX1:2	1.5	1.4	1.8	1.4		1.7			
CEBPA:2	2.9	3.8	4.8	3.3	3.0	3.0	2.8	3.2	2.9
CEBPB:2	2.8	3.8	3.8	3.9	3.5	3.0	4.2		2.4
CEBPD:2	3.0	4.1	3.9	4.1	3.6	3.2	4.5	1.3	2.6
CEBPE:2	3.0	3.9	3.8	3.6	3.3	3.2	3.9	1.4	2.4
CEBPG:2	3.8	4.4	3.5	3.4	3.9	2.8	3.7	1.8	2.4
CLOCK:2	1.5	1.8							
CREB3L2:2	2.9	4.1	2.2	2.3	2.4	1.6	2.2		
CTCF:2	1.6	2.4		1.7					
DMRT3:2	1.3	1.7			1.3			1.3	
E2F6:2	2.3	2.6	1.8						
E2F8:2	2.4	2.3	2.8	2.4	2.7		2.5		
EBF1:2	3.4		1.9	2.4	2.4		2.1		1.9
EGR1:2	1.6	1.4	1.8						
EGR2:2	1.8	3.7	2.4		1.5	1.6	1.9		1.9
EGR3:2	1.9	3.4	2.9		1.9				2.5
EGR4:2	1.6	2.5	1.8						
EHF:2	2.8	4.6	4.7	5.3	6.0	2.4	5.3	5.2	4.8
ELF3:2	3.1	6.0	5.0	6.1	5.4	2.8	4.8	7.0	5.3
ELF4:2	2.2	4.8	5.0	5.4	6.6	2.7	5.4	5.3	6.3
ELF5:2	5.7	7.0	6.7	9.5	7.1	3.4	6.1	5.3	6.4
ELK4:2	3.1	5.0	5.6	5.4	5.3	2.3	4.0	6.3	3.6
EOMES:2	5.6	5.0	5.3	5.5	5.2	2.3	3.5		1.9
ERF:2	5.0	11.2	5.6	4.8	5.0	2.1	3.8	3.9	3.4
ERG:2	3.2	6.8	2.9	3.8	3.0		2.0	2.4	1.7
ESR1:2	1.6	3.8	2.9	1.9	2.9				
ESR2:2	3.2	1.3	2.2	1.5	2.7				
ETS1:2	3.4	9.1	4.4	5.5	4.1	2.2	3.5	2.8	2.6

ETV2:2	6.7	15.8	8.1	9.8	8.8	3.4	7.2	3.8	4.8
ETV6:2	2.2	9.0	5.1	4.0	5.6	2.0	5.6	4.5	4.4
EWSR1-FLI1:2	2.9	4.4	3.0	2.3	3.1	1.3	2.5		1.5
FEV:2	1.8	3.7	2.0	1.6	1.7			1.5	
FIGLA:2	2.4	3.8	2.7	3.9	2.9		1.6	2.5	2.2
FLI1:2	2.4	6.6	3.0	3.3	2.7	1.6	2.0	2.6	2.0
FOS:2	4.4	7.6	4.8	2.9	3.0	2.5	2.8	1.4	3.1
FOS::JUN:2	5.7	7.3	5.0	4.1	4.7	2.7	3.3		1.9
FOS::JUNB:2	6.4	6.2	5.6	4.0	4.5	2.3	3.4		2.1
FOS::JUND:2	5.7	6.1	5.4	4.4	4.9	3.0	4.4	1.3	2.5
FOSB::JUNB:2	5.8	6.1	5.6	3.8	4.6	3.4	3.7		2.3
FOSL1:2	4.5	6.4	6.7	4.4	5.7	2.3	4.2	1.7	3.2
FOSL1::JUN:2	6.7	6.4	5.4	5.3	4.9	3.2	3.9		2.1
FOSL1::JUNB:2	6.3	7.1	4.6	4.1	3.5	2.7	3.1		2.4
FOSL1::JUND:2	5.2	7.3	4.9	4.2	4.2	2.5	3.3		1.8
FOSL1::JUND(VAR.2):2	1.7	1.6	2.2	2.5	1.8		1.5		
FOSL2:2	5.4	7.0	6.3	5.3	5.4	2.1	4.1	1.9	3.1
FOSL2::JUN:2	5.7	5.8	4.7	4.5	4.1	2.7	3.2		1.6
FOSL2::JUN(VAR.2):2	1.4	1.4	2.2		1.8				
FOSL2::JUNB:2	3.9	6.3	4.2	3.7	3.5	3.0	2.9		1.6
FOSL2::JUND:2	5.0	6.0	4.6	4.1	3.7	3.0	2.9		1.9
FOXA1:2	6.5	4.6	6.0	5.8	5.8	2.7	4.3		1.6
FOXA2:2	5.4	4.2	5.7	2.9	4.8	2.5	3.4	1.6	2.4
FOXB1:2	3.3	3.7	3.4	2.9	2.9		1.6		1.3
FOXC1:2	1.9	3.4	2.8	2.7	2.4		1.9		
FOXC2:2	4.0	4.1	3.7	2.8	2.8		1.9		1.8
FOXD1:2	4.4	4.2	4.2	4.2	4.0	2.3	1.9	1.3	2.0

FOXD2:2	4.4	4.2	4.2	4.2	4.0	2.3	1.9	1.3	2.0
FOXD3:2	1.8	2.5	2.6	2.6	3.1		1.5		1.3
FOXF2:2	2.2	3.4	2.0	2.2		1.9			
FOXG1:2	4.7	3.4	3.2	3.0	2.6	1.8	1.4		
FOXI1:2	4.4	4.2	4.2	4.2	4.0	2.3	1.9	1.3	2.0
FOXJ2:2	4.6	3.6	2.9	3.3	3.3	2.4			
FOXJ3:2	1.5	1.5	2.1	1.4		1.4			
FOXK1:2	3.3	3.8	4.1	3.4	3.5	2.1	1.9		1.8
FOXK2:2	3.5	5.1	3.9	3.9	3.0	1.9	1.8		1.6
FOXL1:2	4.4	4.2	4.2	4.2	4.0	2.3	1.9	1.3	2.0
FOXO1:2	3.8	6.1	5.7	3.3	4.8	3.0	3.4	2.6	3.7
FOXO3:2	4.9	4.7	4.8	4.6	4.9	2.3	2.2	1.4	2.3
FOXO4:2	4.4	4.2	4.2	4.2	4.0	2.3	1.9	1.3	2.0
FOXO6:2	4.4	4.2	4.2	4.2	4.0	2.3	1.9	1.3	2.0
FOXP1:2	5.0	5.6	4.7	3.9	4.7	3.1	2.9	1.9	3.1
FOXP2:2	2.9	5.1	3.0	2.2	3.1	3.0	2.7	1.3	1.6
FOXP3:2	4.4	4.2	4.2	4.2	4.0	2.3	1.9	1.3	2.0
FOXQ1:2	2.4	4.0	3.0	1.6	2.1	1.7		1.3	1.5
GABPA:2	2.0	3.8	2.5	4.0	2.7		1.8	1.9	1.8
GATA1:2	3.0	2.5	4.7	3.4	4.5	2.5	3.4		2.4
GATA1::TAL1:2	3.0	3.3	3.0	2.7	3.0				1.4
GATA2:2	3.8	3.8	5.9	3.4	5.4	3.4	4.5		2.3
GATA3:2	1.4	3.4	4.0	1.7	3.3		2.4		2.1
GATA4:2	4.0	4.9	5.5	3.6	5.6	2.8	3.9		2.4
GATA5:2	2.4	4.8	5.2	2.7	4.9	2.8	3.3		2.4
GATA6:2	3.3	4.6	5.9	3.1	5.1	3.4	3.7		2.8
GFI1B:2	2.6	4.7	3.7	2.1	1.6				

GLI2:2	1.4	1.6	1.6	3.5					
GRHL1:2	2.1	1.9	4.7	4.0	3.8	3.2	2.5		1.9
GRHL2:2	3.1	3.8	6.7	6.2	7.2	3.2	5.4	2.4	4.5
HIC1:2	1.6	2.6	1.8		1.6		1.3		
HLF:2	1.4		1.6	1.8	1.5				
HMX1:2	1.3								
HMX2:2	1.7	4.0	1.6	2.1	1.9		1.4		1.6
HNF1A:2	2.3	1.4	3.5	3.4	3.1		2.5	2.2	2.2
HNF1B:2	2.8		4.5	4.3	3.4		2.9	1.8	2.1
HNF4A:2	5.2	5.8	6.0	7.3	5.4	3.4	5.1	4.0	5.5
HNF4G:2	5.4	6.0	6.8	4.1	6.8	3.2	5.4	1.8	3.8
HOXA10:2	2.0	3.1	2.7	1.8	2.2	1.6	1.8		
HOXA11:2	1.9	2.0		2.5					
HOXA13:2	1.6	1.6	1.8		1.5	1.8			2.1
HOXC10:2	2.3	2.8	3.4	3.0	2.7	2.6	1.7		1.5
HOXC11:2	2.3	1.6	1.3	1.8					
HOXC12:2	2.4	2.0	1.5	2.0					
HOXD11:2	1.7	1.7		1.6					
HOXD12:2	2.0	2.4	1.9	2.5					1.3
HOXD13:2	1.3	2.8	2.8	3.0	3.0	3.4	2.7		2.5
HOXD9:2	1.7	2.1	1.6	2.2	1.7	2.0	1.6		
ID2:2	2.6	4.1		1.3					
IRF1:2	4.3	7.1	5.8	4.3	4.9	1.9	3.7	2.5	4.8
IRF2:2	4.2	4.9	3.2	3.6	2.8	1.7	1.8	2.7	3.3
IRF3:2	5.6	7.0	6.0	3.9	4.7	3.2	4.3	2.6	2.5
IRF4:2	5.0	6.7	6.6	4.2	4.9	1.4	4.5	1.3	3.6
IRF5:2	2.0	3.3	2.0		1.3			1.3	2.2

IRF7:2	3.2	5.0	3.9	4.6	3.0	2.3	2.9	1.3	3.1
IRF8:2	6.6	8.8	5.6	4.7	4.6	3.0	3.6	1.6	3.0
IRF9:2	5.7	6.0	3.9	3.4	2.5		1.6		2.1
ISL2:2	1.7		1.7		1.9		1.6		
JDP2:2	4.6	6.7	3.7	2.0	2.3	1.7	2.0		
JUN:2	1.4	2.5	3.0	3.0	3.3		1.6		1.7
JUN(VAR.2):2	3.8	7.1	5.0	3.5	4.0	2.7	2.8		2.4
JUN::JUNB:2	4.3	6.1	4.0	3.1	3.3	1.7	2.1		
JUN::JUNB(VAR.2):2	1.4	1.7	1.4						1.5
JUNB:2	5.0	7.6	5.5	4.2	4.3	2.3	2.9		2.0
JUND:2	5.1	5.6	5.5	3.5	4.8	2.3	3.6		2.7
KLF1:2	4.3	8.2	6.7	7.3	7.8	3.4	5.9	1.9	3.0
KLF12:2	3.3	5.0	6.0	4.5	6.6	3.4	4.8	2.4	4.4
KLF13:2	2.1	2.8	2.3	2.2	2.7		1.5		1.5
KLF14:2	2.5	4.1	4.2	3.8	4.1		2.0		2.4
KLF16:2	2.0	5.1	3.3	3.5	3.8		2.5	1.3	3.4
KLF4:2	3.3	4.7	3.8	2.8	3.7	1.5	2.8	1.3	2.4
KLF5:2	2.5	6.6	6.7	4.5	6.6	2.0	5.0		4.1
KLF9:2	5.0	7.0	6.7	6.2	6.6	3.7	6.1		3.5
LEF1:2	2.8	2.2	1.4	1.5					
MAF::NFE2:2	3.5	4.4	4.8	3.0	5.0	2.3	3.1		2.9
MAFB:2	3.8	8.9	6.7	6.0	7.5	1.5	5.0	1.9	4.1
MAFF:2	2.1	2.8	2.6	2.4	3.1	1.6	2.3		2.2
MAFG:2	1.5	2.0	1.6	1.6	1.7				
MAFK:2	2.0	4.2	3.9	2.7	4.1	2.1	3.6	1.6	2.7
MAX:2	2.0	3.3							
MAX::MYC:2	2.0	1.5		1.4					

MECOM:2	3.8	2.6	3.0	1.9	2.6	1.7	2.1		
MEIS1:2	2.7		1.6						
MEIS2:2	2.5	5.0	3.6	2.6	2.4				
MEIS3:2	1.8	2.7	2.2	2.1	1.9		1.4		
MGA:2	1.4	3.0	2.2	3.1	2.5	2.3	1.7		
MITF:2	2.2	3.6	2.8	2.4	2.8	1.4	2.1	1.3	
MLX:2	2.0	2.9		1.6					
MLXIP:2	2.3	3.7		1.4					
MLXIPL:2	2.4	2.7		2.7					
MNT:2	2.0	2.8							
MSC:2	3.5	4.4	5.0	3.3	3.5		1.9		2.3
MTF1:2	1.7	2.2	1.9	2.5	1.7	1.5	1.5		
MXI1:2	2.1	2.6							
MYC:2	1.6	1.9							
MYCN:2	2.7	2.8							
MYF6:2	3.2	7.8	4.9	2.6	4.1	2.0	2.8		2.4
MYOD1:2	3.4	4.3	4.8	5.0	3.1		1.4		1.8
MYOG:2	3.0	3.4	4.4	3.9	3.1	1.4	1.6		1.3
NEUROD1:2	1.7	2.5	2.1	2.1	1.4				
NFAT5:2	1.4	3.8	2.3	2.6	2.3	1.7	1.8		2.1
NFATC1:2	3.4	4.5	3.7	3.9	3.4	1.7	1.9	1.7	2.4
NFATC2:2	3.9	4.3	5.1	3.8	3.3	2.0	2.3		2.2
NFATC3:2	3.1	5.8	3.6	4.4	3.3	1.8	2.1		2.1
NFE2:2	2.8	6.4	3.6	2.0	2.0				
NFE2L2:2	4.1	3.1	4.7	3.1	4.6	1.9	3.3		2.3
NFIA:2	1.3	2.0	1.8	2.4	1.7		2.0		1.5
NFIC:2	1.7	1.4							
NFIC::TLX1:2	1.5								
NFIL3:2	1.5								
NFIX:2	1.9	3.1	2.2	1.9	2.4		2.1		

NFKB2:2	2.0	2.5							
NHLH1:2	2.1	3.7	2.7	2.8	2.1				
NKX3-1:2	1.3		1.5		1.7				
NKX6-1:2	1.6			2.8					
NKX6-2:2	1.3		1.8	2.2	1.7				1.4
NPAS2:2	1.6	2.5							
NR1H2::RXRA:2	2.8	2.9	2.6	3.6	2.8	2.3	3.3		2.3
NR1H3::RXRA:2	2.7	3.2	3.1	3.0	2.4		2.0		1.8
NR2C2:2	3.9	2.1	3.1	2.7	3.8		3.7		3.4
NR2E1:2	2.3	2.6	3.8	4.1	4.4	1.6	2.3	1.8	2.4
NR2F1:2	2.6	4.0	2.7	5.0	3.2	2.0	3.0		2.5
NR2F2:2	4.1	4.9	4.4	4.1	4.7	3.1	2.9	1.6	2.2
NR2F6:2	3.2	2.5	2.9	3.1	2.6		2.8		2.5
NR2F6(VAR.2):2	2.1	1.6	1.6	3.3	2.3		1.7		1.7
NR4A1:2	3.8	4.4	4.2	4.0	5.0	1.9	3.3		2.1
NR4A2:2	2.6	4.6	3.6	2.8	2.8	3.2	1.5		
NR5A2:2	1.6	3.3	2.5	1.8	1.9				
NRL:2	4.4	7.6	5.6	4.4	5.2	2.0	3.7	1.8	2.5
PBX1:2	1.9								
PDX1:2	1.6	2.8	1.6		1.8	1.5			
PKNOX1:2	2.2	4.8	2.3	3.4	1.7	1.3	1.5		
PKNOX2:2	3.2	4.2	2.8	3.0	3.3	2.3	1.8		
POU1F1:2	1.4	2.0	2.2	2.2	2.2		1.8		1.7
POU2F1:2	2.5	2.0	4.3	3.1	3.5		1.9		2.4
POU2F2:2	1.5	3.1	3.6	2.5	2.8		1.9		1.7
POU2F3:2	1.7	2.2	2.0	1.8	1.5				
POU3F2:2	1.3	1.8	1.8	2.9	1.8				1.6
POU3F3:2	2.0	1.5	2.8	3.0	2.7		1.4		1.4
POU3F4:2	2.1	2.4	2.8	3.2	2.4				2.0
POU4F1:2	2.5	1.6							

POU5F1:2	3.1	5.5	6.0	4.2	6.0		3.0		2.2
POU5F1::SOX2:2	3.2	3.5	3.0	2.4	2.2				1.6
POU5F1B:2	2.0	2.1	2.7	3.2	2.4				2.2
PPARA::RXRA:2	4.2	6.9	6.1	5.3	5.5	2.0	4.7	1.4	4.4
PPARG::RXRA:2	3.2	2.9	3.0	1.8	2.7	1.3	2.7	1.3	1.9
PRDM1:2	3.1	4.4	2.5	4.0	2.3	1.6	2.0	1.9	2.7
RARA:2	2.1	3.3	2.5	2.1	2.0		1.8		
RARA(VAR.2):2	1.6	1.5		1.5					
RARA::RXRG:2	1.9	1.5	1.3						
RARB:2	3.7	3.3	3.3	3.7	3.1	1.4	2.6		1.5
RARB(VAR.2):2	2.5	4.2	2.2	3.6	2.7	1.6	2.5	1.3	2.0
RARG:2	2.0	2.7	2.2	2.0					
RARG(VAR.2):2	2.0	2.2	3.1	2.9	3.2	1.7	2.0		2.0
RBPJ:2	3.1	5.8	3.3	3.3	3.5	2.3	2.9		1.6
REL:2	3.1	7.1	4.9	3.7	6.1	3.0	5.4		1.9
RELA:2	3.1	5.4	4.1	5.4	5.5	1.5	3.9		2.1
RELB:2	2.5	3.9	2.6	2.6	2.2				1.6
RHOX11:2	4.3	4.2	5.0	4.1	5.0		3.5		1.8
RORA:2	2.3	3.7	3.0	2.9	4.1		3.0		2.4
RORA(VAR.2):2	2.6	3.3	3.9	2.9	3.3		2.4		1.3
RORB:2	1.4	2.5	2.6		2.2	1.5			
RREB1:2	4.5	4.6	4.8	5.2	4.5	1.9	3.4	1.3	1.9
RUNX1:2	5.7	4.2	5.9	4.8	5.0	2.3	3.7		2.2
RUNX2:2	3.0	4.6	3.7	3.6	2.9	2.0	2.6		2.1
RUNX3:2	3.5	3.7	3.1	3.3	3.0	2.1	2.8		2.2
RXRA:2	4.2	3.1	3.6	2.9	3.2		3.4		3.0
RXRA::VDR:2	1.6		1.8	2.5	1.9	1.4	1.8		1.4
RXRB:2	3.5	2.7	3.9	3.9	3.3		3.5		2.2
RXRG:2	4.3	2.5	3.0	3.3	3.0		2.2		1.8
SCRT1:2	3.8	5.5	5.1	2.9	3.2		1.9		2.9

SCRT2:2	3.5	4.6	3.1	3.3	2.2		1.9		2.9
SIX1:2	2.6	3.7	1.3						2.3
SIX2:2	4.7	4.6	2.8	1.4	2.2	2.0	1.6		2.3
SMAD2::SMAD3::SMAD4:2	1.6	2.4							
SMAD4:2	1.7	1.4			1.6				2.4
SNAI2:2	1.5	2.4		1.5					
SOX1:2	1.5		1.6	1.9					1.8
SOX10:2	4.0	6.0	5.0	4.0	3.4	1.4	1.9		1.7
SOX15:2	1.9	4.4	2.9	2.0	2.0	1.6	1.8		1.8
SOX21:2	1.7	1.7		1.8					
SOX3:2	3.9	5.8	3.9	2.8	2.6		1.9	1.5	2.4
SOX6:2	3.7	5.8	2.8	3.2	2.2		2.1		1.9
SOX8:2	2.6	2.9	4.2	5.3	4.4		2.9	1.4	3.0
SP1:2	2.3	5.4	4.5	4.0	4.5	2.3	4.0		2.6
SP2:2	2.0	5.6	3.5	3.5	3.2	1.5	2.4		1.9
SP3:2	3.5	6.3	5.6	5.6	6.4	3.0	4.8	1.3	4.5
SP4:2	4.5	5.6	5.0	5.6	5.2	1.7	3.4	1.3	2.3
SP8:2	2.5	3.7	4.2	3.3	3.9	1.3	3.3		3.4
SPI1:2	4.6	12.1	6.7	9.8	7.1	3.2	6.4	3.7	8.2
SPIB:2	5.7	7.9	6.7	3.8	5.1		3.9	2.3	3.4
SPIC:2	6.7	9.7	5.9	5.6	5.2	3.4	4.0	1.8	3.6
SPZ1:2	2.6	3.5	4.0	2.7	4.9	2.4	3.6		2.4
SREBF1(VAR.2):2	1.4		1.9	3.0	1.6				
SRY:2	1.9	2.9	1.3		1.4		1.4		
STAT1:2	5.4	8.3	5.2	4.4	5.1	3.4	4.9	1.6	4.5
STAT1::STAT2:2	7.2	9.0	9.1	5.6	5.9	3.3	4.9	3.1	6.1
STAT3:2	3.9	6.3	6.0	3.6	5.7	2.4	2.9		2.5
STAT4:2	6.3	12.1	6.7	5.5	5.5	2.3	4.0		2.4
STAT5A::STAT5B:2	3.5	3.9	3.0	3.0	4.7	3.5	4.5	2.5	4.7
STAT6:2	4.7	5.6	5.2	4.1	4.4	1.9	3.6	1.4	2.7

T:2	1.9	2.8							
TAL1::TCF3:2	3.6	3.8	3.9	1.9	2.8		1.8		2.1
TBR1:2	3.5	4.9	5.3	4.9	4.7	2.1	2.7		1.5
TBX1:2	3.0	4.1	5.2	4.6	4.9	3.0	3.6		
TBX19:2	1.6	1.3				1.8			
TBX2:2	4.2	6.6	6.1	4.9	5.3	1.6	3.7		1.5
TBX20:2	3.4	4.2	4.0	4.5	4.1	2.1	2.3	1.3	2.3
TBX21:2	5.4	5.2	5.9	4.1	4.8	2.3	2.8	2.0	1.9
TBX4:2	3.0	4.1	5.2	4.6	4.9	3.0	3.6		
TBX5:2	2.9	4.5	3.8	3.7	3.7	1.7	2.1		1.6
TCF12:2	3.3	3.3	4.5	4.2	3.1	1.5	1.7		1.3
TCF21:2	5.8	5.5	5.5	3.4	4.0	2.0	1.7		
TCF3:2	1.8	3.9	2.3	2.9	2.4		2.6		1.4
TCF4:2	2.0	3.7	2.0	3.4	2.3		2.5		1.5
TCF7:2	3.9	6.3	4.9	5.3	5.5	2.0	4.0		2.4
TCF7L1:2	4.0	5.0	4.0	4.6	5.1	2.0	2.8	1.6	3.0
TCF7L2:2	3.5	4.0	3.2	1.8	2.6		1.6		
TEAD1:2	4.4	3.7	2.7	4.2	3.1	1.5	2.8		2.6
TEAD2:2	4.5	7.3	5.8	4.9	6.6	3.2	4.9		3.4
TEAD3:2	2.8	3.7	3.1	4.7	3.9	2.9	3.9		3.0
TEAD4:2	3.4	3.6	2.9	3.9	3.0	2.3	2.8		2.6
TEF:2	1.3	1.9	2.4	1.9	2.5		2.2		1.7
TFAP2B(VAR.2):2	1.6	1.4	2.7	3.5	2.0				
TFAP2C(VAR.2):2	1.4	1.5	2.0	4.1	1.4				1.3
TFAP4:2	5.2	6.2	4.2	5.0	2.8		1.6		1.5
TFCP2:2	2.8	2.0	2.8	3.3	3.3				
TFEB:2	2.1	3.8	2.9	2.7	2.1		1.7		
TFEC:2	2.7	5.9	3.5	2.9	3.2		1.7		
TGIF1:2	2.0	4.6	2.2	1.6	2.0	1.6	1.6		
TGIF2:2	2.4	4.8	2.6	3.2	2.1		1.7		

TP53:2	3.9	3.3	4.5	3.7	4.8		2.6		2.6
TP63:2	4.1	2.5	5.1	3.5	3.9		2.4	1.6	3.3
TP73:2	3.7	4.2	5.9	3.8	4.9	2.1	3.5		2.9
TWIST1:2	6.7	4.8	6.1	4.6	5.1	3.1	3.4		2.2
TWIST2:2	1.4	1.6							
USF1:2	3.7	4.4	3.9	2.9	4.0		2.8		1.6
USF2:2	3.3	3.9	3.8	1.7	3.6		1.5		
VAX1:2	1.3	1.5							
VAX2:2	1.7		1.7	1.5	1.9				
VDR:2	2.1	2.6	3.8	1.9	2.9		2.1		3.3
XBP1:2	1.8	3.4	1.4	2.0	1.3				
ZBTB18:2	3.1	7.5	5.7	6.2	5.0	1.3	3.8		3.3
ZBTB7A:2	1.6	3.8	3.3	4.5	3.3		2.2		2.0
ZBTB7C:2	1.4								
ZEB1:2	1.9	2.6	2.1	1.9	1.9		1.6	2.1	2.2
ZIC3:2	2.5	1.7	1.8	1.6	1.4				
ZNF24:2	1.9	1.7	3.6	3.5	2.6		1.3		
ZNF263:2	5.7	6.6	6.6	4.9	5.4	2.0	4.8		4.5
ZSCAN4:2	3.5	6.0	5.0	4.2	4.9	2.2	2.8		
ATF3:2		1.5							
BHLHA15:2		1.4							
BHLHE41:2		2.0							
CDX2:2		1.4				1.3			
CENPB:2		1.5							
CREB1:2		2.3	2.1		2.1				1.3
CREB3:2		2.3			1.4				
CREB3L1:2		2.9		2.6	1.9				
CTCFL:2		1.6	1.6	1.7					
DDIT3::CEBPA:2		1.9							1.4
ELF1:2		3.3	3.1	3.5	3.4	2.0	3.0	3.3	3.4

ELK1:2		1.5							
ELK3:2		3.0	1.9	1.3	1.9			2.1	
ESRRB:2		1.7							
ETV1:2		1.5		1.7	1.4				
ETV3:2		2.9		1.5	1.7				1.3
FOS::JUN(VAR.2):2		2.7	1.6	1.4	1.4				1.5
FOSB::JUN:2		1.6	2.0	2.0	1.8				1.4
FOSL2::JUND(VAR.2):2		1.6	2.4	1.8	1.9				1.4
FOXH1:2		3.4	1.8		1.5				
GCM2:2		2.0		3.0					
GFI1:2		3.1	2.3		1.9				
GLIS3:2		1.5	1.8		2.2	1.4			1.7
GMEB1:2		2.2	1.8	1.8	1.9				
HAND1::TCF3:2		1.8	1.4		1.4	1.6			
HES2:2		1.4							
HES5:2		1.6							
HEY1:2		1.7							
HIC2:2		1.9							
HLTF:2		2.0	3.5	3.4	3.5		2.4		2.2
HMX3:2		2.1							
HOXA9:2		1.4							
HOXB13:2		2.2		1.4		1.9			
HOXC13:2		2.1	2.6	3.1	2.2	1.8	2.2		2.1
HOXD3:2		1.4							
ID4:2		3.2	2.3	2.4	2.2		1.4		
JDP2(VAR.2):2		1.4			1.4				
JUND(VAR.2):2		2.6	1.3	2.5	2.4		1.8		2.4
LIN54:2		1.7	1.9						
MEF2C:2		1.5		1.3					
MYB:2		2.0	3.1	2.0	2.0		1.4		

MZF1(VAR.2):2		2.3							
NFKB1:2		1.9							
NKX2-5:2		1.3							
NKX2-8:2		1.9	1.5		1.6				
NR1H4:2		1.6		3.5					
NR2E3:2		1.5		1.5					
NR3C1:2		1.7		3.2	1.5	1.5			
NR3C2:2		2.0		3.1		1.6			
PAX6:2		3.4	2.8	3.4	2.3	1.3	1.6		
PBX2:2		2.0		1.3					
PBX3:2		2.0		2.5	1.4				
PITX1:2		1.4							
POU3F1:2		2.5	2.3	3.7	2.4		1.4		
PPARG:2		2.7	2.2		2.2				
RARA::RXRA:2		2.5	2.2	2.7	2.1		1.8		1.6
RFX1:2		1.3			1.4				
RORC:2		1.4			1.4				
SMAD3:2		1.4							
SOX11:2		1.5	1.5		1.4				
SOX13:2		2.0		2.7					
SOX4:2		2.3		1.6					
SPDEF:2		3.4	2.1	2.7	1.9				
SREBF2:2		1.6		2.2					
SREBF2(VAR.2):2		1.5	1.9	2.6	1.6				
TBP:2		2.2	1.7	1.5					
TBX15:2		2.0	2.3	2.6	2.2	2.4	1.5		
TFE3:2		3.3	2.1	3.2	2.1		1.8		
THAP1:2		2.2	1.3	1.7					
ZFX:2		2.7		1.5	1.3				
ZNF143:2		3.4				1.6			

ZNF384:2		1.8	2.0	3.0	2.2	1.4	1.7		3.0
ZNF740:2		2.5	2.0	1.4	1.6				
GSX2:2			2.2	2.5	2.3		1.6		1.3
HOXC9:2			1.4	4.0	1.9				1.5
HSF1:2			1.8		1.3				
HSF2:2			1.7	1.8	1.5		1.6		
HSF4:2			1.5	1.3					
NKX2-3:2			2.2	1.4	1.7				
NKX3-2:2			2.1	1.4	1.7				
POU4F2:2			1.6	1.4					
SRF:2			1.5		1.7		1.6		
TFAP2A:2			1.4	2.8	1.6				
ALX3:2				3.4	1.4				
AR:2				4.1	1.3				
ETV4:2				1.3					
GLIS1:2				1.6	1.5				
LHX3:2				1.6					
LHX4:2				1.8					
LMX1A:2				3.1	1.8		1.5		
LMX1B:2				3.1	1.8		1.5		
MEF2B:2				1.5					
MEF2D:2				2.0					
MEOX1:2				1.5					
MEOX2:2				1.7					
MIX-A:2				1.6					1.5
MNX1:2				1.9					
MYBL2:2				1.3					1.7
POU6F2:2				1.7					1.3
SOX17:2				3.3					1.3
TFAP2A(VAR.2):2				1.3	1.3				

ZBTB7B:2				1.7					
NKX2-5(VAR.2):2					1.6	1.7			1.9
CRX:2						1.5			
ESRRG:2						1.6			
FOSB::JUNB(VAR.2):2									1.4
OLIG1:2									1.6
PROX1:2									1.3
SOX2:2									1.4
SOX9:2									1.9

ALX1:3	3.8	3.9	4.0	3.4	3.6	2.9	4.3	2.9	3.8
ALX3:3	4.5	4.7	3.5	1.9	2.8	3.5	3.9	2.2	1.9
ALX4:3	4.6	3.9	4.1	3.1	3.2	2.0	3.1	3.5	3.5
AR:3	2.1		2.5	1.8	2.3	1.7	2.4	3.8	2.5
ARID3B:3	1.9	1.6	2.0		2.3	1.5	1.7		
ARNT::HIF1A:3	2.4	4.7	3.1	3.0	3.9	3.6	4.8	2.7	4.1
ARX:3	4.7	4.4	4.1	4.2	4.5	4.4	5.6	4.2	5.7
ASCL1:3	6.7	7.2	8.4	7.7	10.1	6.6	9.8	9.1	8.9
ASCL2:3	4.1	5.1	4.4	4.2	4.9	5.0	5.3	5.7	4.7
ATF1:3	1.4	2.4	2.0	1.3	2.3	3.0	2.3	2.4	
ATF3:3	3.3	2.0	1.9	2.0	2.2		1.9	4.5	2.2
ATF4:3	5.4	3.7	6.0	6.8	5.9	3.9	5.1	3.0	4.3
ATF7:3	2.9	5.4	2.7	2.9	2.3	3.0	2.7	3.4	2.9
ATOH1:3	3.8	5.4	5.8	4.5	5.3	4.4	6.2	3.2	4.1
BACH1::MAFK:3	5.5	8.1	7.1	4.4	7.6	4.9	7.5	9.7	10.2
BACH2:3	2.6	5.4	4.8	2.5	3.8	3.2	4.3	4.3	3.6
BARHL1:3	2.5	3.2	4.5	3.3	4.9	3.2	5.2	5.3	5.4
BARHL2:3	1.9	2.5			1.4	2.0	1.8	2.1	1.7
BARX1:3	2.4	2.4	2.7	1.9	2.7	4.8	3.1	3.7	3.2
BATF3:3	5.7	5.5	4.9	3.2	4.6	5.3	4.3	3.5	2.5
BATF::JUN:3	4.3	6.6	3.4	3.0	4.0	3.8	5.0	3.6	3.4
BCL6B:3	1.8	1.4	3.1		3.3	2.9	3.8	2.4	2.5

BHLHA15:3	6.0	7.6	5.0	4.0	5.0	4.6	6.0	6.4	5.0
BHLHE22:3	1.3	3.1	1.5	1.3		2.4		2.4	1.6
BHLHE23:3	1.9	3.6	2.2		1.4	2.3	1.9	1.9	1.6
BSX:3	4.5	6.7	6.4	4.3	6.6	7.5	7.8	5.2	7.0
CDX1:3	3.8	3.4	4.2	2.5	3.0	2.4	3.6	2.6	2.8
CDX2:3	4.1	4.7	3.8	3.8	3.3	2.8	3.1	2.9	3.1
CEBPB:3	1.3		1.7			1.3	1.4		1.6
CEBPE:3	1.5		1.6			1.3	1.5		1.8
CENPB:3	2.1		2.4		2.1	2.3	2.5	2.2	2.2
CREB1:3	3.6	4.6	5.2	3.2	4.2	5.8	4.3	3.6	2.9
CREB3:3	1.8	2.0	2.3	2.2	3.0	4.6	3.1		
CREB3L2:3	1.5	2.0			1.6	3.1	2.2		
CREB5:3	3.4	4.0	2.6	2.5	2.4	3.4	3.4	4.7	4.2
CREM:3	1.8	3.4	3.1		1.9		1.3	1.6	
CRX:3	4.1	3.7	3.7	5.0	4.0	4.2	4.8	2.9	2.6
CTCF:3	3.6	4.3	3.9	2.3	3.4	1.3	3.2	3.5	3.3
CTCFL:3	2.8	5.2	4.9	3.3	5.3	4.5	6.2	3.8	5.2
DBP:3	3.6	3.2	3.4	2.3	2.5	3.1	3.8	2.1	2.9
DDIT3::CEBPA:3	4.9	6.6	7.2	4.5	6.8	5.1	7.3	3.2	3.8
DLX1:3	4.3	7.4	5.0	5.2	4.5	4.5	3.5	4.1	3.4
DLX2:3	3.1	3.2	3.8	3.9	3.9	3.4	3.7	5.8	4.7
DLX3:3	2.9	3.6	4.4	2.9	4.4	1.5	5.1	3.7	5.1
DLX4:3	2.3	3.0	3.5	2.7	3.3	1.7	4.3	3.1	4.5
DLX6:3	3.3	1.9	3.0	2.0	2.9	1.9	3.4	3.1	3.5
DMBX1:3	2.4	1.9	3.7	2.2	2.3	2.2	2.8	5.2	4.5
DMRT3:3	2.6	2.3	3.2		2.2		1.9	2.1	1.8
DUX:3	2.3	4.3	5.1	3.6	4.7	3.6	4.7	3.1	4.6
DUX4:3	2.8	6.5	4.6	3.1	5.4	5.7	7.2	2.8	5.6
DUXA:3	3.8	4.1	4.5	2.4	5.4	5.2	5.9	2.5	4.6
E2F2:3	1.7	2.6	2.2	2.1	1.9	1.7	1.8		1.3

E2F3:3	2.5	2.7	2.5	1.7	2.2	2.0	2.0	1.5	1.3
E2F4:3	5.4	2.3	3.2	2.3	3.5	4.0	4.9	5.7	5.9
E2F6:3	9.8	10.5	12.5	11.5	14.0	13.4	15.8	16.3	14.7
E2F7:3	1.3			2.0					1.9
E2F8:3	1.6	3.2	3.6	2.2	2.3	1.6	2.7	2.7	3.5
EBF1:3	4.7	5.2	5.6	3.6	4.8	5.4	4.2	4.3	4.9
EGR1:3	7.4	5.1	6.1	4.1	6.5	9.2	6.6	7.2	7.2
EGR2:3	10.3	7.1	9.0	6.4	8.7	10.6	10.0	13.2	10.1
EGR3:3	9.7	8.0	14.0	7.6	13.1	11.5	12.1	11.2	12.3
EGR4:3	9.8	8.1	10.5	7.5	9.8	10.4	9.5	16.1	11.3
EMX1:3	4.4	3.5	3.7	4.7	3.8	4.0	3.5	2.4	2.1
EMX2:3	4.1	4.0	3.8	3.6	3.5	7.3	4.2	4.4	3.7
EN1:3	4.9	3.4	4.2	2.8	3.8	5.5	4.8	4.9	4.8
EN2:3	4.3	4.8	3.4	3.5	3.2	5.7	3.4	3.9	4.0
EOMES:3	3.6	3.1	4.0		3.5	6.3	3.6	2.9	3.5
ESR1:3	3.6	3.4	3.6	2.4	3.6	4.9	5.3	5.1	5.2
ESR2:3	4.7	4.5	5.8	2.1	5.4	5.6	6.3	9.4	6.6
ESRRA:3	4.6	5.4	4.1	5.0	4.7	3.1	4.3	5.7	4.4
ESRRB:3	3.3	2.5	2.8	3.4	3.8	4.4	4.4	4.6	3.5
ESRRG:3	4.7	5.7	5.7	5.6	5.8	4.6	5.8	6.3	5.3
ESX1:3	4.7	5.8	4.7	3.8	4.6	6.8	5.3	4.2	5.7
EVX1:3	3.6	4.2	3.3	2.6	2.6	4.2	2.7	3.4	2.8
EVX2:3	3.2	2.6	2.0	1.6	1.6	3.5	1.6	2.8	1.7
EWSR1-FLI1:3	10.6	13.1	12.3	8.6	12.9	15.0	15.9	16.5	14.9
FIGLA:3	3.5	6.4	4.8	2.2	4.4	4.1	3.4	2.4	3.1
FOS:3	5.3	7.7	7.8	4.1	7.7	5.2	7.9	4.5	4.4
FOS::JUN:3	3.1	4.0	5.0	3.2	5.4	3.8	5.2	3.8	3.8
FOS::JUN(VAR.2):3	2.7	4.1	3.6	3.4	4.4	3.0	4.2	4.3	5.2
FOS::JUNB:3	3.1	4.1	5.8	4.1	6.6	4.5	7.3	4.1	5.1
FOS::JUND:3	4.1	5.7	6.8	3.7	7.5	6.3	8.0	4.1	5.0

FOSB::JUN:3	3.7	5.7	3.2	3.0	2.6	3.8	3.6	4.4	3.5
FOSB::JUNB:3	3.0	3.8	5.0	2.7	5.4	3.8	5.2	3.8	4.7
FOSB::JUNB(VAR.2):3	1.9	4.1	2.3	1.8	2.1	2.6	2.4	3.1	3.2
FOSL1:3	3.8	5.1	5.0	2.4	4.7	4.5	5.9	3.4	3.4
FOSL1::JUN:3	4.1	5.7	6.1	4.0	6.9	5.7	6.8	3.6	3.9
FOSL1::JUN(VAR.2):3	2.5	4.4	3.0	2.3	2.7	2.3	3.0	4.5	4.1
FOSL1::JUNB:3	3.3	4.2	5.2	3.2	6.0	5.0	5.9	4.5	4.6
FOSL1::JUND:3	3.4	5.2	5.2	3.2	6.0	4.4	5.9	3.3	3.5
FOSL1::JUND(VAR.2):3	1.7	3.2	2.1	1.7	1.5		1.7	2.0	
FOSL2:3	5.2	5.4	5.1	3.4	4.7	5.8	6.2	5.8	4.7
FOSL2::JUN:3	4.2	5.7	6.7	4.0	7.1	4.6	6.8	3.6	4.0
FOSL2::JUN(VAR.2):3	3.8	5.9	4.9	3.7	4.7	5.0	6.6	5.3	5.8
FOSL2::JUNB:3	3.9	4.8	6.3	3.7	6.6	5.3	6.8	4.3	5.2
FOSL2::JUNB(VAR.2):3	3.6	4.7	3.5	3.0	2.9	3.6	4.1	5.2	4.1
FOSL2::JUND:3	3.2	4.1	5.0	3.3	5.7	5.2	6.0	3.6	5.1
FOSL2::JUND(VAR.2):3	3.0	5.1	3.9	3.3	3.0	4.7	4.4	3.6	3.1
FOXA1:3	4.5	4.2	6.8	4.6	6.2	2.3	4.8	4.1	3.4
FOXA2:3	3.3	3.8	4.8	2.1	3.6	1.6	3.3	3.8	3.6
FOXB1:3	1.5	3.3	4.1	2.0	3.6	3.8	4.1	2.2	2.3
FOXC1:3	2.5	4.8	6.1	3.1	5.4	4.6	4.9	3.3	3.6
FOXC2:3	2.2	4.3	5.7	2.6	4.7	4.0	4.3	3.8	2.8
FOXD1:3	1.9	3.5	3.3	1.9	3.0	2.7	3.5	1.6	2.7
FOXD2:3	2.0	3.5	3.3	2.0	3.0	2.7	3.5	1.6	2.7
FOXD3:3	2.7	3.9	3.5	1.7	3.1	4.1	4.2	4.4	2.1
FOXF2:3	4.1	4.6	5.9	4.6	4.4	2.4	5.0	3.6	4.3
FOXG1:3	1.9	2.4	4.6	3.1	4.5	1.5	4.2	1.8	3.2
FOXH1:3	3.9	3.7	5.0	3.9	4.4	4.5	4.7	5.0	5.2
FOXI1:3	2.0	3.5	3.3	2.0	3.0	2.7	3.5	1.6	2.7
FOXJ2:3	2.2	3.8	5.9	4.0	6.0	1.6	4.8	3.3	3.8
FOXJ3:3	2.4	3.3	5.4	1.6	3.1	3.2	4.2	4.4	5.0

FOXK1:3	2.2	4.3	4.4	2.1	3.2	3.0	3.2	1.8	2.6
FOXK2:3	3.3	4.4	5.5	2.5	4.6	3.8	4.0	2.1	3.7
FOXL1:3	2.0	3.5	3.3	2.0	3.0	2.7	3.5	1.6	2.7
FOXO1:3	4.6	5.9	6.5	5.3	5.4	3.9	5.9	4.0	4.6
FOXO3:3	2.3	4.5	4.5	2.0	3.8	3.0	4.3	2.8	3.4
FOXO4:3	2.0	3.5	3.3	2.0	3.0	2.7	3.5	1.6	2.7
FOXO6:3	2.0	3.5	3.3	2.0	3.0	2.7	3.5	1.6	2.7
FOXP1:3	3.1	6.4	3.6	2.6	3.0	4.7	4.6	3.0	4.0
FOXP2:3	3.1	5.5	5.7	2.4	4.3	4.0	3.5	3.4	3.7
FOXP3:3	2.0	3.5	3.3	2.0	3.0	2.7	3.5	1.6	2.7
FOXQ1:3	1.8		2.1	2.0	2.2	3.1	2.1		2.0
GATA1:3	2.1	4.7	3.8	1.8	3.8	4.1	3.9	4.4	4.3
GATA1::TAL1:3	2.4	5.2	4.3	1.8	3.5	3.5	4.0	3.9	4.6
GATA2:3	2.0	2.8	2.9	1.9	2.5		3.4	3.4	3.1
GATA3:3	1.9	3.5	3.6	1.6	2.7	2.0	2.8	3.1	3.2
GATA4:3	3.4	4.9	4.0	3.3	3.3	4.9	3.7	5.1	5.6
GATA5:3	1.8	2.9	3.2	1.4	2.9		2.2	1.6	2.0
GATA6:3	2.4	4.8	3.8	1.9	3.0	2.1	2.0	2.6	2.8
GBX1:3	6.4	5.6	6.2	5.2	5.3	7.7	6.7	6.0	6.8
GBX2:3	3.7	3.6	4.0	3.2	3.4	5.1	3.7	4.7	4.6
GCM1:3	3.4	4.4	3.9	4.2	4.0	4.1	3.9	4.3	4.4
GCM2:3	3.0	4.0	3.8	3.2	4.3	5.3	5.0	6.4	4.5
GFI1:3	5.6	5.8	4.4	3.6	4.2	4.1	5.0	8.0	5.7
GFI1B:3	5.8	7.6	6.7	4.2	6.2	5.6	7.2	9.2	5.9
GLI2:3	2.1	1.4	2.4		1.4	2.1	2.8	3.6	3.0
GLIS1:3	3.3	2.0	2.5	2.6	3.8	4.5	3.4	3.3	3.0
GLIS2:3	6.0	6.4	5.8	3.9	5.9	9.0	6.8	6.3	5.7
GLIS3:3	5.2	4.7	5.8	4.2	6.2	6.1	5.3	6.5	5.6
GMEB1:3	1.6	2.4	1.4				1.7	3.6	1.6
GRHL2:3	1.6			1.3			2.3	1.8	2.9

GSC:3	3.4	3.5	2.6	3.1	2.5	3.7	2.1	4.6	2.5
GSC2:3	3.3	2.1	2.7	3.2	3.0	4.2	3.6	4.7	2.7
GSX1:3	4.8	3.6	4.5	4.0	4.8	4.2	3.7	3.2	3.0
GSX2:3	5.6	5.0	5.1	5.2	5.8	4.1	4.6	2.8	2.9
HAND1::TCF3:3	2.2	2.4	2.6	2.6	3.3	2.7	2.8	2.6	1.8
HES1:3	3.9	4.6	4.5	1.9	3.3	5.2	2.4	4.7	2.5
HES2:3	2.6	2.7	2.4	2.4	2.3	5.0	2.4	1.5	1.3
HES5:3	1.4	3.2	2.0	2.1	2.0	2.6	2.5	2.4	1.6
HEY1:3	2.8	3.1	3.2	2.3	2.4	3.7	2.4	2.0	
HEY2:3	3.3	4.1	3.9	3.0	3.3	4.1	3.0	2.7	1.4
HIC1:3	6.1	8.2	7.1	4.8	6.8	7.0	7.6	6.0	7.3
HIC2:3	6.4	6.5	7.2	6.0	7.2	7.8	8.2	4.6	6.1
HIF1A:3	2.6	2.6	1.8	1.4	1.5	3.1	2.2	2.4	2.7
HINFP:3	4.7	1.8	4.4	2.1	4.1	3.5	5.2	4.3	4.0
HLF:3	3.6	1.9	3.1	1.9	2.3	3.4	4.2	3.2	3.8
HLTF:3	2.2	3.1	4.0		2.9	2.9	3.1	1.7	2.7
HMX1:3	2.7	4.4	5.9	4.1	5.0	3.8	4.4	5.2	5.0
HMX2:3	4.6	3.7	4.4	2.5	3.7	2.9	3.3	2.2	2.6
HMX3:3	3.5	2.2	4.1	2.5	3.9	3.1	4.5	3.8	4.1
HNF4G:3	1.5	3.0	3.2	2.7	4.0	2.3	3.7	2.4	2.7
HOXA10:3	4.2	3.6	5.6	4.4	5.2	4.9	5.9	3.9	4.1
HOXA11:3	1.4	2.2		1.6	1.7		1.9	1.3	1.3
HOXA13:3	2.7	3.3	3.2	1.3	2.6	3.3	3.6	3.1	3.2
HOXA2:3	4.7	4.9	4.0	4.2	4.3	4.6	4.6	4.8	4.7
HOXA5:3	2.9	3.0	3.2	1.8	3.0	1.7	2.7	1.4	1.7
HOXA9:3	2.6	3.8	6.4	4.9	6.1	3.9	6.8	3.4	4.4
HOXB13:3	3.2	4.2	3.1	1.6	2.7	2.8	3.4	2.0	3.3
HOXB2:3	4.0	2.5	2.8	3.7	2.5	6.9	3.3	3.7	3.2
HOXB3:3	3.6	2.3	4.5	3.6	4.5	6.8	6.0	4.1	5.3
HOXB5:3	3.7	3.8	3.7	3.8	4.2	5.0	5.3	4.0	4.2

HOXC10:3	1.6	3.8	1.9	1.5	1.6		2.8	2.2	1.8
HOXC11:3	2.2	3.5		1.6	1.6		1.9		
HOXC12:3	1.7	2.1		1.6				1.8	
HOXC9:3	1.5	3.6	3.4	1.9	2.0	1.7	2.9	3.9	3.2
HOXD13:3	3.6	4.2	4.5	2.6	4.5	4.5	3.3	2.5	2.1
HOXD3:3	4.6	5.4	4.5	3.7	5.7	6.3	7.0	4.6	5.7
HOXD8:3	4.0	2.3	2.4	2.2	2.2	2.5	2.6	3.5	3.3
HOXD9:3	4.0	3.4	4.0	2.5	4.1	5.7	6.8	5.3	5.1
HSF1:3	2.0	2.2	1.8		1.8		2.2	1.4	
HSF2:3	1.6	2.5	2.6	2.0	3.0	2.0	3.5	2.6	3.0
HSF4:3	2.3	1.8	2.5	1.9	2.7	1.6	2.7	3.0	2.4
ID4:3	3.7	8.8	4.9	2.4	5.4	5.6	6.5	5.0	4.9
INSM1:3	3.1	4.9	3.5	4.2	4.7	8.8	8.1	5.3	6.6
IRF1:3	5.4	6.7	5.5	4.2	6.0	4.0	5.5	4.5	4.1
IRF2:3	3.4	5.4	3.5	3.7	3.7	4.3	4.3		
IRF3:3	2.6	3.5	4.1	4.1	4.8	4.1	4.0	2.6	5.7
IRF5:3	2.4	3.2	4.1	2.4	5.0	2.1	3.6		2.2
IRF7:3	2.8	5.0	5.5	2.9	5.4	3.7	4.5	4.6	5.4
IRF9:3	3.9	6.5	8.9	4.2	7.1	3.6	6.5	1.8	4.0
ISL2:3	2.7	3.8	2.8	2.3	2.9	3.8	3.2	3.7	2.7
ISX:3	5.2	5.2	5.5	5.5	5.8	4.1	4.8	3.7	4.9
JDP2:3	2.4	5.3	4.7	3.7	6.1	3.6	6.0	2.1	3.1
JDP2(VAR.2):3	4.4	5.1	3.6	2.0	2.4	2.6	3.3	3.7	3.4
JUN:3	5.4	8.2	6.9	4.2	4.9	7.0	7.0	4.1	4.7
JUN(VAR.2):3	4.1	7.4	6.2	4.5	6.1	3.8	5.3	3.2	2.1
JUN::JUNB:3	3.8	6.7	5.1	3.2	5.3	3.7	5.4	4.9	4.3
JUN::JUNB(VAR.2):3	2.5	5.6	3.1	1.9	2.2	3.0	1.6	2.9	2.3
JUNB:3	3.5	5.0	5.0	2.1	4.7	4.3	5.8	2.9	3.0
JUNB(VAR.2):3	3.6	5.1	3.8	2.6	3.0	2.1	2.5	3.4	2.7
JUND:3	4.4	6.6	5.9	4.0	5.2	3.9	5.6	3.9	3.7

JUND(VAR.2):3	5.6	8.3	6.2	4.2	4.7	4.3	5.8	4.0	3.9
KLF1:3	6.4	7.1	6.0	4.6	5.5	8.4	6.0	6.4	5.9
KLF12:3	2.6	2.7	2.6	4.1	3.5	3.7	4.4	3.7	3.5
KLF13:3	2.0	2.8	3.3	3.0	4.0	4.3	3.8	2.3	2.6
KLF14:3	6.1	5.5	6.8	6.0	7.8	6.9	8.6	5.3	5.7
KLF16:3	11.1	10.5	11.8	6.8	9.0	8.8	7.6	7.3	7.5
KLF4:3	4.5	3.9	3.3	2.0	2.9	6.1	4.2	6.2	6.0
KLF5:3	8.8	11.7	12.9	11.3	14.0	14.6	16.0	13.4	14.3
KLF9:3	11.5	11.7	12.1	6.0	10.5	11.8	11.3	15.1	11.3
LBX1:3	5.5	4.6	5.0	5.2	4.9	4.6	4.1	3.2	3.3
LBX2:3	4.5	6.4	5.4	5.5	5.8	6.3	5.6	3.9	6.0
LEF1:3	3.2	4.2	5.5	3.7	5.0	2.7	3.8	3.6	4.2
LHX2:3	5.2	3.9	5.9	5.5	6.0	5.1	5.4	4.5	4.1
LHX3:3	1.9	2.4	3.2	1.7	3.1	2.8	3.2		1.7
LHX4:3	3.1	1.6	1.6	1.5	1.9		2.0	1.4	1.4
LHX6:3	3.1	1.8	3.0	2.2	2.5	3.1	2.6	2.4	1.8
LHX8:3	4.4	2.8	4.2	3.2	3.5	2.6	3.0	3.6	2.7
LHX9:3	5.2	5.2	5.5	5.5	5.8	4.1	4.8	3.7	4.9
LIN54:3	3.0	4.7	2.4	1.8	2.2	3.4	2.9	2.6	1.6
LMX1A:3	2.9	2.2	1.3	2.3	1.6	1.9	2.0		1.6
LMX1B:3	2.9	2.2	1.3	2.3	1.6	1.9	2.0		1.6
MAF::NFE2:3	4.1	4.3	6.3	3.2	6.5	3.4	6.7	6.5	7.2
MAFB:3	5.3	4.7	6.8	4.9	5.5	5.0	5.9	5.8	7.9
MAFF:3	2.6	3.4	3.2	2.4	3.8	4.5	4.9	2.6	2.9
MAFG:3	4.0	4.5	4.3	2.4	4.7	3.6	5.8	4.4	4.4
MAFK:3	2.3	2.0	2.1		2.1	5.6	3.3	2.3	3.0
MAX::MYC:3	1.4	3.4	2.1	3.1	2.4	2.2	3.9	3.4	3.5
MECOM:3	1.5	1.6	1.8	1.8	1.8	2.3		1.8	2.4
MEF2A:3	4.7	4.2	6.1	3.3	6.0	6.3	6.6	1.7	3.3
MEF2B:3	4.2	2.8	4.0	2.6	4.9	6.6	4.9	3.2	3.7

MEF2C:3	2.1	4.3	4.6	3.8	5.1	4.0	4.3	2.7	
MEF2D:3	4.4	3.0	4.9	2.1	5.5	5.2	5.2		2.6
MEIS1:3	1.7	2.5	1.4				1.5	1.7	
MEIS2:3	3.8	7.9	4.4	3.1	4.0	2.2	4.9	5.3	2.8
MEIS3:3	3.6	6.1	3.6	2.6	3.8	1.8	4.0	4.1	1.9
MEOX1:3	3.4	4.3	3.2	4.3	4.2	5.2	3.8	2.6	2.9
MEOX2:3	5.1	6.8	4.8	4.9	4.8	5.9	3.9	3.9	3.3
MGA:3	4.1	4.1	3.8	2.2	3.0	4.8	2.7	1.9	2.2
MIX-A:3	2.0	1.8		2.1	1.7	3.0	2.0	2.4	1.6
MIXL1:3	4.2	4.7	4.3	3.7	4.4	4.1	3.9	3.7	4.0
MNX1:3	4.5	3.1	3.5	4.0	3.7	4.2	4.8	3.7	4.1
MSC:3	3.0	6.5	4.0	4.7	5.2	4.8	5.8	3.7	3.7
MSX1:3	4.2	4.7	5.5	4.2	5.5	4.9	6.5	5.6	6.5
MSX2:3	3.6	5.2	5.5	3.8	5.7	6.5	5.7	5.2	5.8
MSX3:3	3.6	5.2	5.5	3.8	5.7	6.5	5.7	5.2	5.8
MTF1:3	4.9	5.2	5.6	3.8	4.9	4.5	5.2	3.8	4.1
MYB:3	1.5	4.6	3.5	2.4	3.1	2.8	3.1	1.4	2.2
MYBL1:3	1.8								
MYC:3	1.3	3.6	2.7	2.4	2.6	3.2	3.8	1.4	2.6
MYF6:3	2.4	3.8	2.4	3.2	3.0	3.3	2.7	3.1	1.8
MYOD1:3	7.3	9.2	7.8	4.9	8.2	6.2	8.6	6.8	6.2
MYOG:3	6.4	8.1	7.1	5.3	7.2	7.1	6.8	5.8	5.7
MZF1(VAR.2):3	12.8	12.4	13.5	8.2	12.9	11.4	14.4	16.2	14.9
NEUROD1:3	6.2	7.6	8.7	7.5	8.1	6.5	9.0	6.8	5.5
NEUROD2:3	1.8	2.3	2.6	2.5	2.8	2.5	2.3	2.1	2.5
NEUROG1:3	5.7	9.2	7.2	5.6	6.6	4.2	6.8	4.4	4.2
NEUROG2:3	4.2	2.7	5.0	4.0	4.0	4.5	4.7	3.9	3.7
NFAT5:3	3.9	5.0	4.4	3.0	4.1	2.7	4.2	4.3	4.2
NFATC1:3	3.1	4.3	3.6	2.3	3.8	4.2	3.8	1.9	3.1
NFATC2:3	2.2	2.3	1.9	1.6	2.2	2.1	1.7		1.3

NFATC3:3	4.6	4.6	4.7	2.8	4.5	4.6	4.5	2.2	3.4
NFE2:3	2.6	5.7	3.4	2.9	3.1	3.5	3.9	3.3	2.7
NFE2L2:3	4.8	7.9	8.5	5.2	7.2	5.1	9.2	7.6	10.2
NFIA:3	2.7	3.6	3.9	2.1	3.0	2.9	2.7	2.9	3.7
NFIC:3	2.6	3.4	4.8	3.2	4.5	4.6	5.1	3.0	4.4
NFIC::TLX1:3	4.4	5.0	4.3	4.7	4.3	4.3	4.9	3.3	4.5
NFIL3:3	3.1	3.1	5.1	2.5	2.8	2.7	4.2	2.2	3.3
NFIX:3	3.9	3.9	4.9	4.0	4.0	3.3	3.9	3.5	3.6
NFKB2:3	2.1	1.9	3.2	2.8	2.9	1.9	3.1	1.5	1.8
NFYA:3	2.6	3.2	2.7	1.8	3.8	3.6	3.2	3.3	2.3
NFYB:3	3.4	4.1	3.2	1.9	2.9	4.0	3.3	4.3	3.1
NHLH1:3	5.2	7.9	5.9	3.7	6.0	5.5	6.4	4.6	6.2
NKX2-3:3	3.2	5.5	5.1	4.3	5.1	3.9	4.2	5.5	4.9
NKX2-5:3	2.8	2.6	4.5	2.5	4.0	1.7	3.5	2.3	3.4
NKX2-5(VAR.2):3	2.6	4.4	5.5	4.1	6.5	6.5	5.9	6.5	6.6
NKX2-8:3	3.5	4.9	3.2	5.0	3.7	5.0	4.2	4.9	4.7
NKX3-1:3	2.3	3.0	2.8	2.0	2.5	2.9	2.9		2.5
NKX3-2:3	2.8	3.1	3.0	1.8	2.5	2.8	2.6	1.4	1.6
NKX6-1:3	3.6	4.4	6.3	5.1	6.1	4.7	5.8	3.2	4.0
NKX6-2:3	4.7	4.7	5.9	4.9	6.0	4.7	4.7	1.9	2.9
NOBOX:3	1.8	3.0	3.2	3.3	3.7	3.7	4.4	5.5	4.8
NOTO:3	3.8	2.6	4.2	3.0	3.8	3.0	3.5	3.8	3.0
NR1H2::RXRA:3	1.3	1.8	2.0	1.3	1.9	3.2	3.3		1.3
NR1H3::RXRA:3	3.4	2.8	3.1	1.6	2.9		3.2	3.4	2.8
NR1H4:3	1.6	2.7	3.1		2.8	2.2	3.6	3.2	2.4
NR2C2:3	4.2	6.0	4.7	3.4	4.0	5.1	4.4	4.1	4.6
NR2E1:3	2.0	3.0	3.5	1.8	2.9	3.0	3.2	2.4	2.5
NR2E3:3	1.8	2.3	2.2	2.2	2.3	1.5	1.5	2.3	1.7
NR2F1:3	3.5	5.0	5.7	3.8	5.0	3.5	6.1	4.0	4.9
NR2F2:3	3.4	3.8	5.3	4.1	6.2	3.4	6.8	3.5	5.3

NR2F6:3	1.8	3.7	2.7	2.4	2.8	2.5	3.5	1.5	2.5
NR2F6(VAR.2):3	2.0	2.2	2.2	2.0	2.3	1.9	2.5	1.4	1.4
NR3C1:3	1.5	2.0	3.5	2.2	2.5	2.1	2.5	4.2	2.5
NR3C2:3	1.9	1.9	4.5	3.3	3.6	2.8	3.2	4.3	3.4
NR4A1:3	2.1	3.3	4.8	3.0	4.9	4.6	6.2	4.1	4.7
NR4A2:3	2.7	3.5	5.7	2.1	5.5	3.2	7.8	3.4	5.6
NR4A2::RXRA:3	1.4		1.3		1.5	1.4	1.7	1.9	1.4
NR5A2:3	2.8	3.6	3.5	4.9	4.7	2.4	5.1	4.2	5.0
NRF1:3	8.1	8.1	8.9	8.1	9.0	13.2	8.8	8.8	9.9
NRL:3	6.2	6.0	7.3	4.2	6.3	5.2	6.1	4.2	6.0
OLIG1:3	2.0	5.2	3.2	1.3	2.2	2.6	2.7	2.8	3.0
OLIG2:3	2.1	5.9	4.0	2.2	3.4	3.7	3.6	1.6	2.6
OLIG3:3	1.6	5.1	3.2		2.9	4.1	3.1	1.8	2.1
ONECUT1:3	3.0	2.3	4.4	2.2	4.6	3.3	3.3	1.9	2.5
ONECUT2:3	2.1	3.7	4.5	2.0	4.3	2.5	3.4	4.2	4.0
ONECUT3:3	2.2	3.1	4.2	2.0	4.5	2.4	2.9	4.8	2.9
OTX1:3	2.1	2.6	2.0	3.0	2.2	2.1	2.6	4.1	3.0
OTX2:3	2.6	2.2	3.2	2.5	3.0	2.4	3.9	6.0	4.5
PAX1:3	5.5	5.0	4.4	2.5	3.9	4.7	5.1	4.2	3.7
PAX2:3	4.6	6.9	6.5	4.3	5.8	2.7	6.0	4.9	4.0
PAX4:3	4.7	4.0	4.7	4.2	4.7	2.9	4.2	2.8	2.9
PAX5:3	5.5	5.0	6.2	4.6	5.9	5.2	6.7	4.9	6.3
PAX6:3	5.6	4.6	7.9	4.5	6.7	7.3	6.7	3.5	4.4
PAX9:3	5.4	6.1	6.3	4.1	5.0	6.9	6.8	4.4	4.2
PBX1:3	1.3	2.2	3.9	3.7	4.1	3.6	5.1	4.9	5.5
PBX2:3	3.2	8.3	5.9	4.7	5.3	3.3	7.2	10.0	7.9
PBX3:3	8.5	11.9	9.9	5.4	8.9	7.1	9.7	10.8	8.4
PDX1:3	3.2	2.8	2.4	2.7	2.0	5.3	2.8	2.0	1.9
PHOX2A:3	2.3	2.4	2.1	2.7	2.6	1.8	2.6	4.0	3.8
PHOX2B:3	2.4	2.5	1.8	2.3	2.3	1.9	2.8	4.5	3.8

PITX1:3	4.9	5.3	5.5	4.5	5.5	6.3	7.1	5.4	5.2
PITX3:3	4.8	5.3	5.6	5.0	5.8	7.6	6.8	5.4	4.0
PKNOX1:3	3.1	5.2	5.1	4.4	5.0	2.6	5.0	2.8	3.9
PKNOX2:3	2.6	5.5	5.0	5.6	4.9	3.2	4.0	2.9	3.5
PLAG1:3	9.4	11.1	9.3	8.8	10.3	11.5	11.3	10.4	9.2
POU1F1:3	4.0	4.9	5.6	4.7	4.9	3.6	5.8	5.8	6.2
POU2F1:3	4.6	4.6	6.2	3.2	5.4	4.6	5.2	4.4	5.0
POU2F2:3	5.3	5.8	8.2	4.1	8.3	5.9	6.6	5.7	5.6
POU2F3:3	3.7	5.1	5.7	4.2	5.4	2.3	4.5	2.9	4.3
POU3F1:3	3.5	3.5	4.8	3.0	3.4	3.7	4.1	4.7	6.6
POU3F2:3	2.6	3.4	4.8	3.3	3.7	4.6	4.0	4.7	6.0
POU3F3:3	3.2	5.7	6.1	4.1	5.5	3.8	4.9	4.5	5.2
POU3F4:3	3.0	3.7	5.3	3.0	5.4	3.9	5.0	3.4	5.1
POU4F1:3	3.8	6.0	4.7	5.0	4.6	3.7	2.9	6.1	3.3
POU4F2:3	5.1	5.5	5.4	4.9	5.7	4.7	6.0	6.3	5.7
POU4F3:3	3.8	4.7	4.0	4.5	4.2	3.3	3.0	6.6	3.1
POU5F1:3	3.9	5.3	5.1	3.7	4.7	5.7	4.7	5.5	4.5
POU5F1::SOX2:3	5.0	7.3	6.8	3.0	5.4	3.9	5.5	5.5	4.8
POU5F1B:3	2.6	4.4	6.0	3.9	6.3	4.0	5.6	2.7	5.9
POU6F1:3	2.2	1.7	1.4	2.0		2.2	1.4		1.5
POU6F2:3	3.4	6.6	4.4	3.0	4.0	5.2	4.7	4.7	5.0
PPARA::RXRA:3	1.8	2.4	2.4	2.2	2.6	2.4	3.6	3.1	3.4
PPARG::RXRA:3	7.6	9.5	9.6	4.9	10.5	8.2	8.7	5.3	7.2
PRDM1:3	3.4	3.0	5.5	2.5	5.9	6.8	6.3	2.1	3.2
PROP1:3	3.0	2.1	3.5	2.8	3.7	2.5	4.1	3.0	3.3
PRRX1:3	5.2	5.2	5.5	5.5	5.8	4.1	4.8	3.7	4.9
PRRX2:3	4.0	5.6	5.3	3.9	5.5	4.5	6.3	4.2	5.6
RARA:3	3.2	3.1	5.8	3.6	5.1	2.8	6.2	2.7	4.4
RARA(VAR.2):3	3.0	3.6	5.2	2.2	5.0	4.3	6.1	4.1	4.7
RARA::RXRA:3	4.0	5.2	5.7	5.6	6.3	7.0	5.5	3.9	4.1

RARA::RXRG:3	5.2	3.8	5.9	4.8	6.6	4.6	7.2	2.9	5.6
RARB:3	5.3	4.1	5.9	2.5	6.3	3.2	6.2	2.8	4.0
RARB(VAR.2):3	2.5	2.7	5.7	2.6	4.9		4.6		3.2
RARG:3	1.8	4.3	3.2	1.4	4.2	3.8	4.9	2.7	4.1
RARG(VAR.2):3	3.3	5.3	6.1	4.7	6.1	2.6	4.6	3.7	4.0
RAX:3	3.5	4.0	5.5	5.0	5.9	4.1	4.9	4.4	5.5
RAX2:3	5.2	5.2	5.5	5.5	5.8	4.1	4.8	3.7	4.9
RBPJ:3	2.8	3.9	5.9	4.2	6.7	4.5	6.8	3.0	4.8
REL:3	1.4	1.4	1.5	2.3	2.1	1.5	1.6		
RELB:3	4.5	4.5	4.4	3.3	4.6	9.3	5.2	4.3	5.3
REST:3	7.4	9.0	7.4	6.0	5.8	7.1	7.6	9.7	6.5
RFX1:3	4.0	6.3	6.4	3.8	6.4	5.7	6.8	6.0	4.1
RFX2:3	5.2	6.8	7.2	4.1	6.5	7.0	7.6	8.0	5.7
RFX3:3	5.2	6.6	6.7	4.0	6.0	6.0	6.8	8.5	5.4
RFX4:3	4.6	7.4	6.7	2.8	6.3	5.9	8.1	7.7	5.1
RFX5:3	4.7	9.1	8.7	4.9	8.1	7.2	10.7	10.7	9.0
RHOX11:3	1.6	4.5	4.3	3.6	3.0	3.5	4.1	2.3	3.4
RHOXF1:3	3.5	2.7	3.2	3.1	3.9	3.5	4.2	2.9	3.1
RORA:3	2.6	5.1	4.2	2.7	4.0	4.1	5.6	4.6	5.7
RORB:3	2.1	3.3	4.9	4.2	5.3	2.1	5.4	7.5	6.2
RORC:3	2.5	2.9	3.7	3.4	4.0	2.4	5.8	5.3	5.1
RREB1:3	8.1	8.3	9.8	6.2	9.0	13.2	8.1	8.1	7.2
RUNX1:3	3.0	3.5	3.8	3.7	4.8	4.6	7.1	4.4	6.3
RUNX2:3	1.3	1.9				1.5	1.5	3.3	2.3
RUNX3:3	2.3	5.2	3.6	3.5	4.6	2.6	5.2	3.7	4.3
RXRB:3	1.5	4.8	3.1	1.7	2.3	2.1	2.5	1.6	1.5
SCRT1:3	1.3	1.6	1.5	1.6		2.3		1.9	1.7
SCRT2:3	2.6	3.9	5.2	3.4	4.7	3.2	4.5	3.4	5.1
SHOX:3	5.2	5.2	5.5	5.5	5.8	4.1	4.8	3.7	4.9
SHOX2:3	5.2	5.2	5.5	5.5	5.8	4.1	4.8	3.7	4.9

SIX1:3	4.3	4.4	5.2	3.5	5.8	4.8	4.7	2.5	2.9
SIX2:3	2.6	4.5	5.9	4.7	5.7	5.0	5.0	4.2	3.8
SMAD2::SMAD3::SMAD4:3	6.7	8.6	8.3	5.0	9.7	8.8	10.8	5.8	8.2
SMAD4:3	2.1	3.2	2.6	2.9	2.8	3.7	3.7	4.5	4.2
SNAI2:3	5.5	9.1	7.2	3.0	6.5	5.9	6.2	6.3	5.2
SOX1:3	3.0	3.4	4.4	1.9	4.0	4.0	4.5	4.0	3.3
SOX10:3	4.2	6.1	6.8	4.5	6.2	5.4	6.3	5.1	3.6
SOX11:3	2.4	2.6	3.2	1.8	3.3	4.2	3.2	1.3	2.8
SOX13:3	5.1	2.0	3.0	3.6	3.3	5.9	3.7	3.0	3.2
SOX15:3	4.0	4.9	3.9	3.2	3.3	3.9	3.1	3.7	1.7
SOX17:3	4.3	4.0	6.2	3.8	6.0	4.8	5.8	6.7	5.9
SOX2:3	4.8	3.9	5.3	3.1	4.0	7.6	4.8	5.3	4.7
SOX3:3	6.4	5.8	7.5	5.9	6.7	7.0	7.1	5.9	6.6
SOX4:3	2.1	2.8	3.2	1.8	3.6	4.4	3.6	3.7	4.4
SOX6:3	6.0	4.6	6.0	3.9	5.2	5.9	5.9	4.1	3.8
SOX8:3	2.3	3.3	4.8	3.0	5.9	5.0	5.7	1.6	3.0
SOX9:3	3.5	2.6	2.8	2.3	3.2	2.8	2.6	2.3	3.3
SP1:3	14.3	17.1	18.5	13.7	20.1	21.9	21.1	17.1	19.9
SP2:3	15.6	17.8	18.0	15.2	20.1	19.4	21.8	21.9	20.6
SP3:3	11.1	9.1	11.0	7.6	10.0	11.5	11.0	9.6	9.0
SP4:3	5.7	5.0	4.3	4.1	4.3	5.7	4.2	4.5	3.6
SP8:3	8.9	8.2	9.3	7.5	9.0	12.1	9.6	8.9	8.0
SPI1:3	1.8	4.0	2.7	1.3	2.8	4.5	3.0	1.3	2.2
SPIB:3	4.3	4.5	5.7	4.1	5.8	6.4	6.7	6.7	5.7
SPIC:3	2.3	3.5	3.3	2.9	3.6	4.7	4.8	3.6	4.1
SPZ1:3	3.7	4.8	5.7	3.4	4.5	4.1	5.2	7.9	4.9
SREBF1(VAR.2):3	1.6	2.2	1.7	1.6	1.8	2.5	2.5	2.0	1.8
SREBF2:3	4.5	8.8	7.7	4.8	8.7	6.9	10.6	10.0	9.3
SREBF2(VAR.2):3	1.5	1.9	1.8	1.5	1.9	2.5	1.9	1.9	1.4
SRF:3	1.4	1.3	3.3	1.7	1.9	2.3	2.2	1.5	2.3

STAT1::STAT2:3	2.8	4.1	5.5	5.0	5.6	4.8	5.2	4.3	5.6
STAT3:3	1.6	3.4	1.8	2.2	2.2	1.6	3.0	2.5	2.5
STAT6:3	1.4	3.1	4.0		3.7	2.5	4.1	5.1	5.7
T:3	1.5	1.9	1.9		2.1	3.7	2.9	2.3	3.4
TAL1::TCF3:3	2.7	6.0	5.8	4.4	5.3	4.6	4.9	4.3	3.6
TBP:3	4.8	3.1	4.4	2.9	3.9	3.2	4.1	4.1	4.2
TBR1:3	3.9	3.2	3.6		3.4	8.3	3.4	3.9	4.0
TBX1:3	3.0	5.3	4.4	2.0	3.6	6.7	3.9	3.7	4.4
TBX15:3	2.6	4.6	3.1		2.2	4.5	1.8	2.3	2.8
TBX19:3	1.8	4.4	4.1	1.9	4.0	5.6	4.2	2.5	3.0
TBX2:3	4.7	4.6	4.0		3.3	8.2	3.3	2.6	3.3
TBX20:3	4.5	2.8	4.4	2.0	4.1	5.1	4.0	3.6	3.0
TBX21:3	4.0	4.2	4.3	1.7	4.0	5.5	4.6	4.2	4.5
TBX4:3	3.0	5.3	4.4	2.0	3.6	6.7	3.9	3.7	4.4
TBX5:3	3.2	5.0	3.1		2.6	8.2	3.7	3.0	2.8
TCF12:3	6.4	7.3	6.8	5.3	6.7	6.3	7.1	5.2	5.7
TCF21:3	3.9	7.1	7.1	5.6	6.4	4.6	5.9	4.9	3.8
TCF3:3	2.4	7.8	4.0	2.1	4.8	5.5	5.0	3.9	3.8
TCF4:3	2.8	9.1	5.3	2.4	5.8	5.6	6.0	4.6	4.7
TCF7:3	2.8	4.9	6.6	3.4	6.5	2.1	6.3	5.2	5.7
TCF7L1:3	2.4	4.3	5.5	4.8	4.5	4.3	4.0	3.4	4.4
TCF7L2:3	4.1	6.1	6.9	5.0	6.2	2.6	5.3	6.8	6.2
TCFL5:3	2.3	2.0	2.4	3.1	2.5	2.9	2.6	5.9	4.3
TEAD1:3	3.5	3.1	3.2	2.1	3.1	2.8	4.2	2.4	3.0
TEAD2:3	2.4	1.4	2.3		2.2	2.2	2.1	2.3	2.4
TEAD3:3	1.6				1.7	1.5	1.8		
TEAD4:3	2.5	1.5	2.1	1.6	2.2	1.8	2.8	2.1	2.2
TEF:3	3.3	2.6	4.4	1.7	3.1	2.6	4.1	3.2	3.3
TFAP2A:3	1.6	2.3	3.6	1.9	3.4	2.5	4.0	4.2	3.5
TFAP2A(VAR.2):3	2.7	2.8	3.3	2.4	3.0	4.4	3.9	3.9	3.9

TFAP2A(VAR.3):3	1.5	3.4	3.7	3.1	4.5	2.8	4.0	3.4	3.3
TFAP2B:3	2.9	2.0	3.2	2.9	3.4	4.1	3.8	4.2	4.3
TFAP2B(VAR.3):3	2.6	4.5	5.2	5.2	7.0	4.6	7.0	3.3	4.1
TFAP2C:3	2.7	2.6	3.9	2.4	3.8	4.1	4.1	4.3	4.8
TFAP2C(VAR.2):3	2.0	2.4	3.2	1.3	2.7	2.0	3.2	2.8	2.8
TFAP2C(VAR.3):3	2.6	3.8	4.9	4.5	6.1	3.2	5.8	3.5	3.7
TFAP4:3	3.6	5.0	4.8	2.8	5.6	6.0	6.5	4.7	5.7
TFCP2:3	2.0		3.0		2.5	2.1	2.4	2.6	2.2
TFDP1:3	5.1	3.9	4.4	3.3	4.8	2.4	3.9	4.3	4.4
TFEB:3	1.3	1.9			1.6	2.4	1.8	1.6	2.3
TGIF1:3	3.0	4.1	3.8	3.7	3.9	3.4	4.8	3.8	4.0
TGIF2:3	3.1	4.2	4.3	4.3	4.1	4.4	4.4	3.4	4.4
THAP1:3	2.6	3.9	3.6	2.5	4.1	2.9	2.7	3.7	3.3
TWIST1:3	3.0	3.9	3.6	3.5	3.5	2.3	3.8	4.3	3.3
TWIST2:3	3.6	6.7	6.0	3.8	4.9	3.2	4.9	1.3	2.8
UNCX:3	5.2	5.2	5.5	5.5	5.8	4.1	4.8	3.7	4.9
USF1:3	2.1	4.5	5.0	3.9	4.8	1.8	4.3	4.3	4.7
VAX1:3	3.7	4.3	3.3	3.7	2.9	6.8	3.3	1.9	1.5
VAX2:3	2.3	2.1		2.5	1.6	3.8	2.0		
VSX1:3	5.6	4.3	5.5	5.0	4.8	5.5	4.8	3.1	2.5
VSX2:3	5.6	4.3	5.5	5.0	4.8	5.5	4.8	3.1	2.5
YY1:3	3.5	6.1	5.7	3.9	5.5	5.5	5.5	4.9	5.0
YY2:3	2.6	2.5				1.9			
ZBTB18:3	6.0	7.4	6.1	5.0	5.7	7.7	6.8	6.3	7.2
ZBTB33:3	2.1	1.5				1.9		2.4	
ZBTB7B:3	4.6	3.0	5.2	4.2	4.0	3.9	4.3	4.3	4.5
ZBTB7C:3	3.9	5.2	5.4	4.2	4.7	6.4	5.8	5.1	5.7
ZEB1:3	4.6	6.3	6.1	4.4	7.0	5.9	6.3	4.3	4.0
ZFX:3	3.0	1.9	3.6	2.6	3.4	3.1	3.7	2.8	2.5
ZIC1:3	4.8	6.6	6.1	5.3	6.5	3.0	6.0	5.8	5.7

ZIC3:3	4.4	8.0	5.5	3.3	5.4	4.9	4.9	5.4	4.1
ZIC4:3	5.4	7.4	5.9	4.5	6.1	3.1	5.9	5.8	6.1
ZNF143:3	5.1	3.1	4.7	4.4	5.3	5.2	7.4	5.6	5.0
ZNF24:3	5.7	7.3	4.6	3.0	4.6	6.7	6.2	6.3	4.3
ZNF263:3	14.3	16.6	15.4	10.1	18.4	16.2	17.1	12.3	11.7
ZNF282:3	4.4	3.9	6.2	4.5	7.0	4.1	7.7	5.4	7.6
ZNF384:3	4.6	4.9	5.4	2.4	5.4	2.6	6.4	5.2	5.3
ZNF410:3	2.7	3.4	3.4	2.8	3.5	3.4	4.7	6.0	4.1
ZNF423:3	2.3	4.9	3.6	2.5	2.3	1.7	3.5	2.7	2.7
ZNF740:3	11.2	10.8	15.4	11.2	14.8	16.2	15.6	17.0	13.8
ZSCAN4:3	5.3	10.0	8.6	4.5	9.8	9.8	10.7	15.3	10.5
ARID5A:3		1.6	2.7	1.6	2.1	2.0	1.7		
ARNTL:3		2.2	1.6		1.5	2.0			
BCL6:3		2.4	2.6		2.4	3.6	2.5		2.5
BHLHE40:3		1.7	2.3	1.6	2.6	2.8	2.0		
BHLHE41:3		1.6	1.4	1.6	1.3				
CEBPG:3		1.4							
CLOCK:3		1.9	2.7		2.7	3.4	2.7		
CREB3L1:3		2.6	2.4	1.9	2.8	6.4	3.9	1.8	1.7
ETV4:3		1.5							
ETV5:3		1.6							
GRHL1:3		1.5					1.9	1.3	2.2
HES7:3		2.2	1.4	1.9	1.4	3.2	1.4	3.1	1.6
HNF1A:3		3.2	2.0	1.6	2.2	3.3	3.0	2.3	1.9
HNF1B:3		2.6	2.3		2.8	3.4	2.5	1.8	1.5
HNF4A:3		1.4	1.3						1.9
HOXC13:3		1.7				1.4		2.2	
HOXD11:3		2.2					1.4		1.5
ID2:3		1.8	2.2		2.4	4.2	3.3		
IRF8:3		1.5	2.9	1.9	3.0	1.4	2.8		1.9

MAX:3		2.7	2.6	1.7	2.5	4.0	3.5		1.3
MITF:3		1.4	1.4	2.0			1.3	1.8	2.9
MLX:3		2.0	2.4	1.6	2.5	1.8	3.3		2.0
MLXIP:3		1.8	2.2		2.5	4.1	3.1		1.4
MLXIPL:3		1.9		1.4	2.2	2.0	2.8	1.3	1.9
MXI1:3		3.0	2.3	2.0	2.2	2.5	3.0		1.6
MYCN:3		2.4	2.5	2.7	2.5		2.8	1.4	1.8
NFKB1:3		2.5	2.7	1.7	2.2		2.3	3.0	2.5
NPAS2:3		2.1	2.1		2.0	3.2	3.7	1.8	1.8
NR1A4::RXRA:3		2.3	1.9	1.5	1.8	1.5	1.5	1.3	
PPARG:3		5.6	2.7		2.3	2.8	3.5	2.7	3.3
PROX1:3		2.1		1.4		1.6	1.8	2.0	
RELA:3		1.3						1.6	1.9
RXRA:3		2.4	1.8	2.3	1.6	1.3	2.1	1.4	1.4
RXRG:3		3.5	2.6	1.9	2.0	2.6	3.0		1.8
SIX3:3		2.8	2.2	3.1	2.7		3.1	2.2	1.8
SOX21:3		1.3	2.2	1.7	2.4	3.8	3.5		2.0
STAT1:3		1.7							
STAT4:3		1.8	2.2	1.6	2.2		2.8	3.4	3.1
STAT5A::STAT5B:3		1.6	1.9		2.3	1.6	2.2		2.0
TFE3:3		2.9	1.8	2.3	2.0	2.1	1.7		
TP53:3		1.6	1.3	1.3		1.6	2.5	2.8	3.3
USF2:3		1.9	1.7	1.9	1.7	3.6	1.7	1.3	
VENTX:3		2.0	1.8	1.4	1.9	2.6	2.4	2.1	2.7
ZBED1:3		2.4	2.3	1.3	2.5	1.4	3.3		2.1
CEBPD:3			1.5				1.3		1.4
CUX2:3			1.4	1.6	1.6				
RORA(VAR.2):3			2.2	2.6	3.3	3.9	5.4	1.6	2.8
RXRA::VDR:3			2.1	1.7	2.5	2.0	2.6	1.9	2.1
SRY:3			2.2	1.4	2.8	1.7	2.5	2.2	1.6

TFAP2B(VAR.2):3			1.4			1.6	1.5	2.7	1.5
TP63:3			1.7	2.3	2.0	1.5	2.4		
CUX1:3				1.4	1.5				
IRF4:3					1.3				
XBP1:3					1.3	2.9	1.7	1.9	2.0
CEBPA:3						1.9	1.7		1.8
TFEC:3						1.7	1.5		
TP73:3						1.4		1.5	1.4
VDR:3						1.7			
SMAD3:3							1.7		1.3
PAX3:3								1.8	1.6
PAX7:3								2.9	2.2
SPDEF:3									1.3

ELK1:4								3.7	
ELK3:4								3.0	
ETV1:4								2.9	
ETV3:4								1.4	
ETV4:4								2.8	
ETV5:4								3.7	

AR:5								2.1	
ARID5A:5								2.8	
ASCL1:5								1.7	
ASCL2:5								2.6	
BACH2:5								1.9	
BATF::JUN:5								4.2	
BCL6B:5								1.6	
BHLHA15:5								1.9	
CDX1:5								1.6	
CDX2:5								1.4	

CREB1:5								1.4	
CUX1:5								1.7	
CUX2:5								1.8	
DLX1:5								1.4	
DMRT3:5								1.5	
E2F6:5								1.6	
E2F7:5								1.3	
E2F8:5								1.6	
EBF1:5								2.7	
EMX1:5								1.7	
EOMES:5								1.8	
ESR2:5								1.5	
ESRRA:5								1.6	
ESRRB:5								2.1	
ESRRG:5								1.3	
EWSR1-FLI1:5								3.6	
FOS:5								2.4	
FOS::JUN:5								1.6	
FOS::JUNB:5								1.5	
FOS::JUND:5								1.8	
FOSB::JUNB:5								2.3	
FOSL1:5								1.8	
FOSL1::JUN:5								1.9	
FOSL1::JUNB:5								1.3	
FOSL1::JUND:5								2.1	
FOSL2:5								2.1	
FOSL2::JUN:5								1.5	
FOSL2::JUNB:5								2.3	
FOSL2::JUND:5								2.2	
FOXA1:5								1.7	

FOXA2:5								1.3	
FOXB1:5								1.5	
FOXC1:5								1.6	
FOXQ1:5								1.7	
GATA1:5								1.8	
GATA1::TAL1:5								1.4	
GATA2:5								1.4	
GATA4:5								1.8	
GFI1B:5								1.4	
GRHL2:5								3.0	
HAND1::TCF3:5								2.3	
HIC1:5								2.4	
HIC2:5								1.6	
HLTF:5								1.5	
HMX2:5								1.3	
HNF4G:5								2.3	
HOXA10:5								1.7	
HOXA13:5								1.5	
HOXA9:5								2.0	
HOXB13:5								1.6	
HOXC10:5								2.1	
HOXC9:5								1.6	
HOXD11:5								1.5	
HOXD12:5								1.4	
HOXD13:5								1.9	
HOXD9:5								1.3	
ID4:5								1.5	
IRF3:5								2.2	
IRF7:5								1.5	
IRF9:5								1.7	

JDP2:5							4.7	
JUN:5							3.1	
JUN(VAR.2):5							2.6	
JUN::JUNB:5							2.2	
JUNB:5							2.2	
JUND:5							2.3	
JUND(VAR.2):5							1.8	
KLF1:5							3.6	
KLF4:5							3.3	
KLF9:5							2.2	
LBX1:5							1.4	
LIN54:5							2.0	
MAFB:5							1.7	
MAX::MYC:5							1.8	
MECOM:5							1.8	
MEIS2:5							1.7	
MEOX2:5							1.5	
MSC:5							2.2	
MTF1:5							1.6	
MYB:5							1.8	
MYF6:5							2.0	
MYOD1:5							2.5	
MYOG:5							2.7	
NEUROD1:5							2.2	
NEUROG1:5							1.4	
NFATC1:5							2.0	
NFATC2:5							1.6	
NFATC3:5							2.2	
NFE2L2:5							1.8	
NFIA:5							2.1	

NFIC:5								1.9	
NFIX:5								2.4	
NHLH1:5								2.1	
NKX2-5(VAR.2):5								1.5	
NKX6-1:5								1.3	
NR1H2::RXRA:5								2.0	
NR1H3::RXRA:5								1.5	
NR1H4:5								2.2	
NR2E1:5								2.0	
NR2E3:5								1.7	
NR2F1:5								2.2	
NR2F2:5								1.8	
NR2F6:5								1.9	
NR2F6(VAR.2):5								1.6	
NR3C1:5								1.5	
NR4A1:5								1.4	
NR4A2::RXRA:5								2.0	
NR5A2:5								1.7	
NRL:5								1.6	
OLIG3:5								1.3	
PAX6:5								1.7	
PBX2:5								3.1	
PBX3:5								2.7	
PHOX2B:5								1.3	
PKNOX1:5								1.9	
PKNOX2:5								2.1	
PLAG1:5								1.6	
POU1F1:5								1.4	
POU2F1:5								2.4	
POU2F2:5								4.2	

POU2F3:5								4.2	
POU3F1:5								2.5	
POU3F2:5								1.9	
POU3F3:5								2.2	
POU3F4:5								2.3	
POU5F1:5								2.8	
POU5F1::SOX2:5								1.9	
POU5F1B:5								2.1	
PPARA::RXRA:5								3.3	
PPARG:5								2.0	
PPARG::RXRA:5								3.6	
PRDM1:5								1.9	
RARA:5								1.6	
RARA(VAR.2):5								2.0	
RARA::RXRA:5								2.2	
RARA::RXRG:5								1.8	
RARB:5								1.6	
RARB(VAR.2):5								2.1	
RARG:5								1.9	
RARG(VAR.2):5								2.0	
RBPJ:5								1.4	
RELB:5								1.4	
REST:5								1.8	
RORA:5								1.8	
RREB1:5								1.6	
RUNX1:5								1.8	
RXRA:5								2.4	
RXRB:5								2.6	
RXRG:5								2.9	
SCRT1:5								2.1	

SCRT2:5								2.0	
SIX3:5								1.8	
SMAD2::SMAD3::SMAD4:5								2.1	
SOX10:5								1.4	
SOX11:5								2.0	
SOX13:5								2.3	
SOX15:5								3.5	
SOX17:5								1.5	
SOX2:5								1.6	
SOX21:5								1.3	
SOX3:5								2.4	
SOX4:5								2.2	
SOX6:5								4.2	
SOX8:5								1.9	
SOX9:5								1.9	
SP3:5								1.8	
SP8:5								2.1	
SPI1:5								1.9	
SPIB:5								1.8	
SPZ1:5								1.6	
SREBF2:5								2.1	
SRF:5								1.5	
STAT1:5								2.8	
STAT3:5								1.6	
STAT5A::STAT5B:5								2.4	
STAT6:5								2.2	
T:5								1.6	
TAL1::TCF3:5								1.4	
TBP:5								1.8	
TBR1:5								2.3	

TBX1:5								2.1	
TBX15:5								1.6	
TBX19:5								1.8	
TBX2:5								2.6	
TBX20:5								2.2	
TBX21:5								1.9	
TBX4:5								2.1	
TBX5:5								1.6	
TCF12:5								3.5	
TCF21:5								2.5	
TCF3:5								1.5	
TCF4:5								1.5	
TCF7:5								2.0	
TCF7L1:5								2.0	
TCF7L2:5								1.5	
TEAD1:5								1.4	
TEAD4:5								1.7	
TFAP2B(VAR.2):5								1.4	
TFAP4:5								2.6	
TFCP2:5								1.6	
TFEC:5								1.6	
TGIF1:5								2.7	
TGIF2:5								2.4	
TP53:5								1.4	
TP63:5								2.0	
TP73:5								1.6	
TWIST1:5								2.1	
TWIST2:5								2.1	
USF1:5								1.4	
USF2:5								1.6	

VAX2:5								1.6	
VSX1:5								1.4	
VSX2:5								1.4	
ZBTB18:5								2.2	
ZBTB7B:5								1.8	
ZBTB7C:5								1.3	
ZEB1:5								1.8	
ZIC3:5								1.4	
ZNF143:5								1.5	
ZNF24:5								2.1	
ZNF263:5								4.7	
ZNF423:5								2.4	
ZSCAN4:5								4.2	

ALX3:6	1.5	2.4	2.2		1.6		1.7		
AR:6	4.5	5.2	3.2		2.1		2.6	1.5	2.2
ARNT::HIF1A:6	2.3	2.0	3.2		2.6		2.2		1.6
ASCL1:6	3.1	4.1	4.2		3.9		3.6	2.0	2.0
ASCL2:6	2.8	3.9	3.5		4.1		4.5	2.1	2.8
ATF7:6	3.0		2.6		2.2		2.0		
ATOH1:6	2.6	2.3	2.3		2.1		1.4		
BACH1::MAFK:6	2.6	5.2	6.1	1.6	4.9		3.9	3.2	1.9
BACH2:6	3.7	5.9	5.3	1.7	4.4		4.5	3.2	1.9
BATF3:6	2.2		2.3		2.6		2.0		
BATF::JUN:6	3.3	1.6	3.7		2.5		1.8		
BCL6:6	2.2	2.3	3.4	1.6	3.1		2.7		
BCL6B:6	1.7						2.0		
BHLHA15:6	2.3	3.4	2.7		2.3		1.9		1.9
BHLHE22:6	2.5	1.3	2.2		2.0		1.5		
BHLHE23:6	2.7	2.4	2.3		2.4		1.6		
BSX:6	1.6	1.9	1.8				1.5		
CDX1:6	2.2	2.3	2.7		2.1		2.0		

CDX2:6	2.1	2.4	4.6		3.7		2.9		
CEBPA:6	2.3	3.9	4.2		4.0		3.0		2.2
CEBPB:6	1.9		2.3		1.6				1.7
CEBPD:6	2.2		2.6		1.8				1.8
CEBPE:6	1.9		2.6		1.8				1.7
CEBPG:6	1.7		3.2		2.1		1.5		1.6
CLOCK:6	1.7	1.5	3.1		2.9		1.8		
CREB1:6	1.8	1.6	2.1		1.6		1.5		
CRX:6	2.6	2.8	3.3		2.8		1.8	1.3	
DBP:6	1.9	1.9	2.0		1.4		1.5		1.6
DDIT3::CEBPA:6	1.4		2.8		1.5				
DLX1:6	2.3		2.5		1.8		1.7	1.4	
DLX3:6	1.6	1.6	1.7						
DLX4:6	1.9	1.7	2.3						
DLX6:6	2.4	2.4	2.8		2.0	1.4	1.8	1.8	
DUX4:6	1.5	1.7	2.7		3.0		2.7	1.5	1.4
DUXA:6	1.3	2.4	1.6		1.5		1.8	1.7	1.5
E2F6:6	3.1	4.9	3.5		3.5		3.4	1.9	1.8
E2F7:6	1.4		1.6		1.6				
EGR2:6	1.9	2.9	2.7		1.5		1.4		
EMX1:6	1.6	2.5	2.7		1.8		2.0	1.9	
EMX2:6	1.4	1.8	2.9		1.9		2.2		
EN1:6	1.5	2.8	3.0		2.6		2.8		
ESR1:6	2.9	3.4	2.2						
ESR2:6	4.6	4.3	4.4	1.7	3.3		2.6		
ESRRA:6	1.5	3.1	2.6		2.5		3.0	1.5	1.5
ESRRB:6	2.0	1.8	3.4		2.4		2.6		1.7

ESRRG:6	1.7	2.0	2.8		2.2		2.4	1.5	1.7
ESX1:6	2.1		3.2		2.5		2.9	1.5	1.4
EVX1:6	2.3		2.9		1.8		2.1		
EWSR1-FLI1:6	3.1	3.5	3.0		2.6		2.7	1.8	
FIGLA:6	1.8	3.2	3.5		2.8		2.7	1.5	1.3
FOS:6	3.6	4.2	4.7		3.8		2.7	1.8	1.5
FOS::JUN:6	3.3	4.1	5.5		4.2	1.3	3.3		1.4
FOS::JUN(VAR.2):6	1.6				1.5		1.4		
FOS::JUNB:6	2.6	3.2	4.3		3.3		2.4	1.5	1.4
FOS::JUND:6	3.2	3.6	4.4		3.1		2.2		
FOSB::JUN:6	2.5		1.4		1.4				
FOSB::JUNB:6	3.8	4.3	4.8		3.9		3.0		
FOSB::JUNB(VAR.2):6	1.9		1.5		1.5				
FOSL1:6	3.0	2.4	3.5		2.9		2.4	1.5	
FOSL1::JUN:6	3.7	3.3	3.9		3.1		2.4	1.3	
FOSL1::JUN(VAR.2):6	1.4								
FOSL1::JUNB:6	3.7	3.4	5.1		3.3		2.6		
FOSL1::JUND:6	3.0	3.0	4.5		3.1		2.6		1.3
FOSL1::JUND(VAR.2):6	1.6								
FOSL2:6	3.3	3.9	3.5		2.8		1.9	1.4	1.4
FOSL2::JUN:6	3.5	3.6	4.5		3.1		2.6	1.5	
FOSL2::JUN(VAR.2):6	1.5								
FOSL2::JUNB:6	5.0	5.2	6.3		5.2		3.6	1.4	1.5
FOSL2::JUNB(VAR.2):6	2.1		2.0		1.8		1.4		
FOSL2::JUND:6	4.0	4.2	4.8		3.7		2.9		
FOSL2::JUND(VAR.2):6	2.5	1.4	1.9		2.0		1.5		
FOXD1:6	2.5	2.3	2.9		2.8		2.8	1.5	

FOXD2:6	2.5	2.3	2.9		2.8		2.8	1.5	
FOXF2:6	2.3	2.5	3.2		3.2		2.3		
FOXG1:6	1.8	1.6	2.0		1.5		2.6		
FOXH1:6	2.6	4.0	4.7		5.2		5.4		1.9
FOXI1:6	2.5	2.3	2.9		2.8		2.8	1.5	
FOXK1:6	1.5	1.8	2.5		2.7		2.6	1.7	
FOXK2:6	1.7	2.1	1.7		1.8		2.0		
FOXL1:6	2.5	2.3	2.9		2.8		2.8	1.5	
FOXO1:6	1.9	2.0	3.2	1.6	3.9		4.1	1.7	1.7
FOXO3:6	2.4	2.0	2.3		2.3		2.5		
FOXO4:6	2.5	2.3	2.9		2.8		2.8	1.5	
FOXO6:6	2.5	2.3	2.9		2.8		2.8	1.5	
FOXP1:6	2.2	1.8	2.3		2.1		2.2	1.9	
FOXP2:6	2.6	3.2	4.2		3.5		3.6	2.3	1.9
FOXP3:6	2.5	2.3	2.9		2.8		2.8	1.5	
FOXQ1:6	1.4		2.5		2.8		3.1		
GATA1:6	4.0	3.6	4.8	1.4	3.1		3.3	1.3	1.3
GATA1::TAL1:6	2.7	2.4	2.3						
GATA2:6	3.9	3.3	4.6		3.3		3.0	1.8	1.4
GATA3:6	3.1	2.5	3.5		2.6		2.9	1.9	
GATA4:6	3.9	2.8	4.5		2.8		2.8	1.5	
GATA5:6	3.7	2.7	4.3	1.5	2.6		2.9	3.0	
GATA6:6	4.5	3.6	5.3		3.4		2.7	2.0	1.4
GBX2:6	1.4	1.7	2.2		1.9		1.9	1.5	
GCM1:6	2.2	2.3	3.2		2.3		1.7		
GFI1:6	2.0	2.3	2.0		1.7		1.5		
GFI1B:6	3.3	2.9	4.0		3.3		2.8		

GRHL1:6	3.1		1.5						
GRHL2:6	3.8	1.5	1.7						
GSC:6	2.6	2.9	3.2		2.2		2.0	1.4	
GSC2:6	2.6	2.8	2.3		1.4		1.5	1.7	
GSX2:6	1.8	1.7	1.8		1.6		1.5		
HES2:6	1.9	1.8	2.6		2.0		1.8		
HEY1:6	1.5	1.7	2.4		1.9		1.3		
HIC2:6	1.8	2.0	1.6					1.5	
HLF:6	1.8	2.3	2.9		2.1		2.0	1.3	1.7
HNF4A:6	3.5	2.4	4.7		4.1		3.7	1.4	1.6
HNF4G:6	4.6	3.1	6.3	1.7	6.0		5.4	1.7	2.6
HOXA10:6	2.5	1.7	2.8		2.6		2.6		
HOXA13:6	2.0	1.3	2.9		2.3		2.1		
HOXA9:6	2.4	2.3	3.5		2.9		2.7		
HOXB13:6	2.2	1.9	3.5		2.3		1.4	1.4	
HOXB2:6	1.4	1.6	2.2		1.8		2.6	1.9	
HOXC9:6	1.7	2.2	2.5		2.0		2.1		
HOXD13:6	1.6	2.5	2.8		2.0		1.5		
HOXD3:6	1.5								
HOXD9:6	2.7	3.2	2.8		2.0			1.3	
HSF1:6	3.1	3.0	3.2	1.3	2.5		2.1		
HSF2:6	3.0	3.7	4.1		3.9		3.6	2.0	2.2
HSF4:6	2.0	1.8	2.7		2.6		2.1	1.7	
INSM1:6	3.0	2.9	6.3	1.7	4.7	1.9	4.4		2.8
IRF1:6	1.4	2.9	2.0		2.1		1.8		
IRF2:6	3.3	3.6	3.5		2.0		3.5	1.5	
IRF3:6	1.4	1.9	2.1		2.0		2.3	1.4	
IRF4:6	1.8		2.2		1.7		1.4		
IRF7:6	2.2	1.8	3.9		3.8		2.8	1.3	
IRF8:6	2.3		2.2		2.4		2.1		

ISX:6	1.4	2.2	2.2		1.7		1.7		
JDP2:6	3.3	3.9	3.9		3.2		2.2		
JUN:6	2.9	5.2	3.3		2.5		2.4	1.7	
JUN(VAR.2):6	3.5	3.0	5.3		3.9		3.3	2.0	1.4
JUN::JUNB:6	2.0	1.6	3.1		2.2		2.0		
JUN::JUNB(VAR.2):6	3.6		1.5		1.5		1.3		
JUNB:6	2.8	3.2	4.3		3.3		2.1	1.5	1.4
JUND:6	4.0	4.3	5.7		4.7		3.8	1.9	1.8
JUND(VAR.2):6	1.8		1.5		1.4				
KLF1:6	2.8	2.4	4.0		3.1		2.9	1.8	1.9
KLF12:6	3.1	1.6	2.4		1.9		1.7		
KLF13:6	1.5	1.4	2.5		2.0		1.8		
KLF16:6	2.0	2.2	1.8		1.3		1.5	1.4	
KLF4:6	3.4	4.1	3.8		2.7		2.1	1.6	
KLF5:6	4.6	5.2	3.7		2.3		2.1	1.5	
KLF9:6	3.9	5.2	5.9	1.6	4.0		2.7	1.8	
LBX1:6	1.4		2.1		2.3		2.4	1.4	
LEF1:6	3.3	4.2	5.3	1.7	4.9		3.8	1.5	1.8
LHX2:6	2.0	2.0	2.3		2.1		1.9		
LHX4:6	1.3	1.5	1.8		1.7		1.5		
LHX8:6	2.3	2.3	3.3		2.3		2.7		
LHX9:6	1.4	2.2	2.2		1.7		1.7		
LIN54:6	2.2		2.0		2.1		2.6	1.4	
LMX1A:6	1.6						1.5		
LMX1B:6	1.6						1.5		
MAF::NFE2:6	2.6	2.9	2.8	1.6	2.7		2.4	3.2	1.5
MAFF:6	2.7	3.5	4.5		2.8		1.9	1.8	1.3

MAFG:6	1.4	2.1	3.9		2.9		2.0	1.5	
MAFK:6	2.1	2.8	3.8		2.6		1.4	2.3	1.5
MAX:6	1.4		1.7		1.6				
MAX::MYC:6	1.5	3.5	3.5		2.8		2.1	1.4	
MECOM:6	3.0	1.5	2.9		2.8		2.2		
MEF2A:6	3.7	4.7	5.1		5.7		5.4	1.9	1.7
MEF2B:6	3.8	4.2	5.3		5.2		5.4	2.3	2.8
MEF2C:6	4.4	2.6	3.1		3.1		3.0	1.6	
MEF2D:6	2.4	2.3	3.6		4.1		4.5	3.0	1.8
MEIS2:6	3.7	1.5	3.0		1.8		1.8	1.9	
MEIS3:6	3.4	2.0	2.8		1.4				
MEOX2:6	1.7		1.5				2.0	1.7	
MITF:6	3.1	2.1	2.9		2.3		1.8		
MLXIP:6	1.5	1.4	1.6		1.4				
MNX1:6	3.2	2.3	2.6		2.2		2.4		
MSC:6	3.3	2.6	3.2		3.6		4.1	1.4	1.6
MSX1:6	1.6	1.4	1.7						
MTF1:6	2.6	2.3	3.3		3.1		3.1	1.4	2.8
MXI1:6	1.8		1.7						
MYB:6	2.3	1.7	2.7		2.2		1.6		
MYBL2:6	2.6		1.6		1.6		1.7		
MYC:6	2.1	1.5	1.5						
MYCN:6	2.3	2.8	2.1		1.6		1.4		
MYF6:6	2.0	1.8	2.9		2.2		2.5		
MYOD1:6	3.2	5.2	4.9	1.6	5.7	1.9	4.5	1.7	1.5
MYOG:6	3.3	5.2	4.6	1.5	5.2	1.8	4.4	1.5	
MZF1(VAR.2):6	4.0	3.5	4.3		3.7		2.8	1.9	1.8

NEUROD1:6	2.4	3.2	2.1		2.1	1.5	1.7	1.6	
NEUROD2:6	1.7	1.6	2.0						
NEUROG1:6	2.9	2.7	1.6						
NEUROG2:6	2.2	2.5	2.5		2.0		2.0		
NFAT5:6	3.7	3.0	6.2	1.5	5.2		4.3	1.7	2.0
NFATC1:6	2.2	2.4	3.4		3.3		2.6		1.4
NFATC2:6	1.9	1.9	2.4		1.8		1.8		
NFATC3:6	1.8	1.8	2.5		2.0				
NFE2:6	3.5	3.0	4.3		3.1		2.6	2.3	
NFE2L2:6	2.0	2.9	4.2	1.7	4.2		3.5	3.2	2.0
NFIA:6	3.3	3.3	4.3		3.3		2.9	2.0	1.9
NFIC:6	2.5	2.0	3.0	1.6	2.2		1.6		
NFIC::TLX1:6	2.4	3.9	4.6		3.2		3.0		
NFIL3:6	1.8	2.7	1.7		1.5			1.9	
NFIX:6	3.7	3.3	3.9	1.6	2.5		1.8		
NFKB1:6	1.6			1.6	1.5		1.8		
NHLH1:6	3.1	4.2	5.1		4.8		4.1	1.9	2.2
NKX2-3:6	2.3	3.3	3.3		2.0		2.0	1.3	1.7
NKX2-5:6	2.2	1.6	2.7		2.1		1.9		
NKX2-5(VAR.2):6	1.6	3.9	3.2		2.6		2.8		
NKX2-8:6	3.0	3.2	3.5		2.6		2.0	1.5	1.3
NKX3-2:6	1.4		1.4		1.4				
NKX6-1:6	1.3	1.4					1.5		
NKX6-2:6	1.7	1.8					1.6		
NOBOX:6	1.5		2.3		1.3		1.7		1.4
NOTO:6	2.2	3.8	3.1		2.2		1.6	1.5	
NPAS2:6	1.4	1.6	2.6		1.8		1.6		
NR1A4::RXRA:6	1.5	3.1	2.7		3.1		2.6		1.6
NR1H2::RXRA:6	2.3	2.6	4.3		3.4		2.5		
NR1H4:6	2.3	3.3	3.1		2.7		2.5		2.0
NR2C2:6	4.5	3.2	5.7		4.1		3.9	2.3	1.8

NR2E1:6	1.5	3.2	3.3		3.2		2.6		
NR2E3:6	1.4		2.0		1.9		2.1		
NR2F1:6	2.8	3.6	4.0		4.1		3.6		1.7
NR2F2:6	3.2	2.8	4.5		3.9		3.6	1.4	1.7
NR2F6:6	2.5	2.6	5.1		4.7		3.0	1.6	1.6
NR2F6(VAR.2):6	3.1	3.9	6.5	1.6	6.0		4.5	2.3	2.8
NR3C1:6	3.3	3.2	3.2		2.2		2.6	1.7	2.3
NR3C2:6	3.4	3.1	3.9	1.4	2.0		2.1	1.4	1.6
NR4A1:6	3.3	2.1	3.8		3.4		3.5	1.9	1.5
NR4A2:6	2.8	2.8	2.9		3.1		3.5	2.9	2.3
NR5A2:6	2.7	3.1	3.6		2.6		3.1	1.9	1.9
NRL:6	2.3	3.4	2.2		1.6				
OLIG1:6	2.3		1.6		1.4				
OLIG2:6	3.6	1.6	2.1		1.9		1.4		
OLIG3:6	3.2	2.8	2.6		1.9		1.4		
OTX1:6	2.0	2.4	1.8						
OTX2:6	2.6		1.8		1.3		1.5	1.9	
PAX4:6	1.8	3.2	3.0		2.6		2.3		
PBX2:6	3.5	3.6	4.7		4.7		3.6		1.6
PBX3:6	2.7	3.3	3.4		3.2		2.8		
PDX1:6	2.1	1.6	2.8		2.4		2.8	1.4	
PITX1:6	2.0		1.5						
PITX3:6	3.0	2.6	2.3					1.5	
PKNOX1:6	1.9	2.5	3.2		2.6		2.4		1.3
PKNOX2:6	2.3	2.9	3.1		3.3		2.8		
PLAG1:6	5.0	7.5	6.0		5.7		4.5	2.5	2.8

POU1F1:6	3.1	3.6	6.5		6.0		4.4	1.5	1.8
POU2F1:6	2.3	1.8	2.6		2.0		1.8		
POU2F2:6	1.6	3.3	2.7		2.7		1.8		
POU2F3:6	3.0	3.5	4.8		2.9		2.2		
POU3F1:6	2.1	2.1	2.9		2.4		1.8		
POU3F2:6	2.7	2.8	4.0		3.1		1.8		
POU3F3:6	1.5	1.5	3.3		3.0		2.2		
POU3F4:6	1.8	2.3	2.2		1.6		1.5		
POU5F1:6	2.3	3.5	3.0		3.1		2.7	1.9	
POU5F1::SOX2:6	1.3	1.4	2.3		2.2		2.7	1.7	2.0
POU5F1B:6	2.6	2.0	3.0		2.4		2.2		
PPARA::RXRA:6	3.0	1.4	3.3		2.9		2.4		
PPARG:6	1.6	1.3	1.9		1.5			1.9	1.6
PPARG::RXRA:6	4.7	4.5	5.9	1.5	6.0		4.4	1.6	1.6
PRDM1:6	3.7	3.5	4.0		3.0		2.2	2.0	1.7
PROX1:6	1.8	1.9	1.9						
PRRX1:6	1.4	2.2	2.2		1.7		1.7		
RARA:6	2.0	2.7	4.7		3.8		3.8	1.7	1.4
RARA(VAR.2):6	2.3	2.8	2.9		2.3		1.5	1.9	1.6
RARA::RXRA:6	2.0	2.8	3.0		2.5		1.9	1.9	
RARA::RXRG:6	2.6	4.8	5.7	1.6	4.7		3.5	2.3	2.8
RARB:6	4.0	3.3	6.3	1.6	5.7	1.4	3.8		1.8
RARB(VAR.2):6	1.8	2.9	2.5		2.1		2.3	1.4	
RARG:6	3.6	3.3	5.9	1.5	5.2		3.6	1.8	1.5
RARG(VAR.2):6	1.6	2.0	2.9		2.6		2.8	1.8	1.8
RAX2:6	1.4	2.2	2.2		1.7		1.7		
RBPJ:6	1.4	2.4	2.6		1.7		1.6	1.5	1.5
REL:6	2.2	2.6	2.6		2.1		2.0		
RELB:6	2.9	1.3	1.4		1.4		2.2		
RFX1:6	3.2	2.0	2.9		1.8		1.3		

RFX4:6	1.4	1.9	1.8		1.6				
RHOX11:6	2.0	2.7	1.8		1.3				
RHOXF1:6	1.5	3.3	3.5		2.2		1.8		
RORA:6	2.8	1.9	3.5		2.7		2.1	1.8	1.3
RORA(VAR.2):6	3.4	1.7	2.6		1.9		1.8		
RORB:6	3.3	2.2	2.2		1.9		1.5		
RORC:6	3.1	2.2	2.5		2.0		1.5	1.7	1.5
RREB1:6	4.5	2.7	4.0	1.4	3.7		3.7	3.2	1.6
RUNX1:6	2.1	2.9	2.9		1.6		2.0	1.4	
RXRA:6	2.0	2.5	4.7		2.9		2.2	1.5	
RXRA::VDR:6	2.0	2.2	3.1		3.1		2.4	1.9	2.3
RXRB:6	2.4	3.2	5.3		4.4		3.0	1.5	1.4
RXRG:6	2.2	3.0	5.9		4.3		3.0	1.4	
SCRT1:6	2.6	2.3	2.7		2.8		2.7	1.4	
SCRT2:6	3.5	4.7	4.5		3.6		3.7	2.5	1.9
SHOX:6	1.4	2.2	2.2		1.7		1.7		
SHOX2:6	1.4	2.2	2.2		1.7		1.7		
SIX1:6	2.2	2.0	4.6		4.7		4.5	2.9	2.0
SIX2:6	4.6	4.5	4.9	1.3	5.0		4.3	2.0	1.8
SIX3:6	1.8	1.5	2.5		2.9		2.8		
SMAD2::SMAD3::SMAD4:6	3.9	2.9	4.2	1.5	3.6		3.0	1.7	1.6
SNAI2:6	1.4	3.2	3.7		3.5		2.6	1.5	
SOX1:6	2.9	2.3	2.6		2.0		2.2	1.9	1.9
SOX10:6	2.2	2.6	3.4		3.9		4.2	1.7	1.8
SOX11:6	1.6	2.5	2.3		1.3		1.7	1.8	1.7
SOX13:6	1.7	1.3	2.9		3.0		2.2	1.8	1.7
SOX17:6	1.4	3.4	2.4		2.8		1.8		
SOX2:6	2.4	2.0	2.9		2.5		3.1	1.7	1.5
SOX21:6	2.3	2.6	2.0		1.8		2.4		1.7
SOX3:6	2.4	3.6	4.0		4.1		3.4	1.9	

SOX4:6	2.2	1.6	2.5		2.3		3.1		1.8
SP1:6	3.8	5.2	2.6	1.3	2.2		2.2		
SP2:6	4.6	4.8	4.0		2.7		2.6	1.3	
SP3:6	3.3	2.8	3.0		2.0		1.7	1.9	
SP4:6	1.9	1.6	2.5		2.3		2.3	1.5	
SP8:6	2.2	3.8	3.0		2.4		2.3	1.5	
SPIB:6	1.6	2.3	2.6		2.4		2.4		1.4
SPIC:6	1.8	1.5	2.1		2.2		2.1		
SPZ1:6	2.1	3.1	4.2		2.8		2.8		1.4
SREBF1:6	1.8	3.3	4.1		2.7		2.4		
SREBF2:6	3.7	2.5	4.4		3.9		3.4		1.4
SRF:6	2.7	3.5	2.2		2.0		2.2	3.2	1.8
STAT1:6	1.4	2.9	2.2		1.9		2.3	1.5	
STAT1::STAT2:6	2.3	2.0	1.6		2.2		2.1		
STAT4:6	2.2	2.3	2.2		2.0		3.0	1.5	1.5
STAT5A::STAT5B:6	2.3		1.8				1.5	1.9	
STAT6:6	3.3	2.8	2.9		2.4		1.5		
T:6	2.1	3.6	3.0		2.0		2.0		
TAL1::TCF3:6	2.6	3.7	2.6		2.3		2.3	1.5	
TBP:6	3.3	2.1	3.9		2.4		3.9	3.1	3.6
TBX19:6	2.4	2.3	2.9		1.9		1.6		
TBX5:6	1.4	1.7	2.9		2.2		1.8	1.5	1.4
TCF12:6	2.9	5.2	4.8	1.5	5.5	1.9	4.5	1.7	1.3
TCF21:6	2.8	2.9	2.2		2.4		2.7		
TCF3:6	1.5	2.3	3.6		2.8		1.8	1.4	
TCF7:6	2.4	3.2	4.5		3.8		3.4		1.8
TCF7L1:6	2.8	3.9	4.1		2.8		1.8		

TCF7L2:6	2.3	2.8	4.3	1.5	3.1		2.6	1.9	
TEAD1:6	2.3	1.9	2.8		1.9				
TEAD2:6	3.9	2.4	3.5		3.0		2.4	2.1	1.6
TEAD3:6	2.8	1.7	2.2		2.0		1.4		
TEAD4:6	3.2	2.2	2.9		2.6		1.8		
TFAP4:6	2.2	3.4	4.7		4.7		3.9	1.9	1.3
TFCP2:6	1.3								
TFE3:6	1.4	2.8	2.5		2.6		2.3	2.8	1.6
TFEB:6	3.0	2.7	2.4		2.6		3.1	1.7	1.3
TFEC:6	3.5	3.1	3.1		2.8		2.8	1.7	1.4
TGIF1:6	2.0	1.9	3.2		3.3		2.0		
TGIF2:6	1.8	1.8	3.0		3.0		2.8		1.4
THAP1:6	2.9	3.6	4.8		3.0		2.4		
TP53:6	1.7	2.5	1.9	1.5	1.5		1.4		
TP63:6	1.6		2.1	1.6	2.0		2.2		
TP73:6	3.4	3.3	3.4	1.6	3.1		3.4	1.7	1.6
TWIST1:6	2.3	4.2	2.7		2.7		2.2		1.5
UNCX:6	1.4	2.2	2.2		1.7		1.7		
USF1:6	1.4	3.3	1.6					1.5	
USF2:6	3.3	3.4	2.7		2.2		1.8	1.5	
VAX1:6	2.1		3.5		3.1		3.8	1.4	1.3
VAX2:6	2.4	2.4	3.2		2.8		3.2	1.5	
VDR:6	3.4	3.3	3.5		3.9		4.0	2.5	1.9
VSX1:6	1.7	2.2	3.2		3.0		3.0		
VSX2:6	1.7	2.2	3.2		3.0		3.0		
ZBTB18:6	2.9	2.6	3.0		2.7		3.0		1.7
ZFX:6	2.1	3.6	2.5				2.0		1.3
ZIC1:6	3.4	3.9	4.1		2.8		2.2		
ZIC3:6	2.4	3.6	2.5		1.8		1.9		
ZIC4:6	1.5	1.8	1.8		1.5		1.8		
ZNF263:6	3.3	5.4	4.5	1.6	4.7	1.8	4.0	1.8	1.8

ZNF282:6	2.4	2.5	2.3		2.2		2.2		
ZNF410:6	2.0				1.5		1.9		
ZNF423:6	1.8	2.8	3.5		1.9		1.8		
ZNF740:6	2.5	3.3	3.0		2.3		2.8	2.0	1.8
ZSCAN4:6	5.7	5.9	6.5	1.6	6.0		5.3	3.1	2.2
ALX1:6		1.5	1.6		1.4		2.0		
ALX4:6		2.0	1.6		2.0		2.0		
ARID3B:6		1.3	1.4				1.7		
ATF4:6		2.2							
BHLHE41:6		1.6	1.7						
CTCF:6		1.6	1.6		1.9		1.4		
CTCFL:6		1.7						1.3	1.5
DLX2:6		1.4							
DUX:6		2.3	2.1		2.2		1.9		
E2F2:6		1.7							
EBF1:6		1.5							
EGR1:6		2.4	2.3		1.5				
EGR3:6		2.1	2.6		1.8		1.4		
EGR4:6		2.8	2.1				1.5	1.3	
EOMES:6		2.3	2.9		2.7		2.4	1.9	1.5
FOXA1:6		2.2	2.5		2.2		2.4		
FOXC2:6		1.6	1.5		2.2		2.3		
FOXD3:6		2.2	2.1		1.7		2.5		
FOXJ3:6		3.1	2.6		2.2		2.0		
GBX1:6		1.5	1.8		1.5		1.7		
GLIS1:6		1.4							
GLIS2:6		1.8						1.4	
GSX1:6		1.9	2.2		1.9		2.1		
HES5:6		1.9	2.4		2.4		2.1		
HIC1:6		1.8	2.5		3.0		2.6		1.8
HMBOX1:6		1.5	1.3						

HMX1:6		2.2	2.4		1.4				
HOXB3:6		1.3	1.6		1.6		2.7	1.3	
HOXC13:6		2.3	1.8				1.5		
HOXD8:6		1.5	1.8		1.8		1.6		
ID4:6		2.2	3.0		2.6		1.7		
LBX2:6		1.5	1.6		1.3		1.6		
LHX6:6		2.2	2.9		2.2		2.1		
MAFB:6		2.5	1.9						
MEIS1:6		1.6	2.2		2.2		2.0		
MEOX1:6		1.5						1.4	
MGA:6		2.3	3.0		2.4		2.2	1.6	1.3
MIXL1:6		2.2	2.1		1.9		1.9		
MLXIPL:6		1.6	2.1		1.8				
MSX2:6		1.8	1.5						
MSX3:6		1.8	1.5						
NKX3-1:6		1.6							
NR1H3::RXRA:6		2.0	3.6		3.5		2.7		1.4
NR4A2::RXRA:6		3.6	3.3		3.3		2.5		1.7
ONECUT3:6		1.5	1.9		1.7				
PAX6:6		1.6	1.3						
POU4F2:6		2.3	2.7		1.6			1.6	
POU6F1:6		2.1	2.8		2.0		1.6		
POU6F2:6		1.5	1.8						
PRRX2:6		2.3	2.4		1.4		1.7		
RELA:6		2.6	2.2		1.4				
RFX2:6		1.5	1.5		1.3			1.7	
RFX3:6		1.7	1.8		1.6		1.4	1.4	1.4
RUNX2:6		1.4	1.6						
RUNX3:6		2.1	2.8		1.5				
SMAD4:6		1.7	1.4						

SOX6:6		2.0	1.8		2.3		2.0	1.9	
SREBF1(VAR.2):6		1.5	1.7		1.5				
TBR1:6		2.2	3.0		3.1		2.7	1.7	1.7
TBX15:6		1.7	2.6		2.5		2.6		1.6
TBX2:6		1.5	2.5		2.7		2.3		1.4
TBX20:6		1.7	3.0	1.5	2.8		2.4	1.3	
TBX21:6		2.3	2.4		2.3		1.6	1.5	1.4
TCF4:6		2.1	3.2		2.2		1.4	1.4	
TEF:6		2.0	1.9		1.5		1.5		
TFAP2A:6		3.4							
TFAP2A(VAR.3):6		1.5	2.3		1.5		1.8		
TFAP2B(VAR.2):6		4.2							
TFAP2B(VAR.3):6		1.5	2.2				1.6		
TFAP2C(VAR.2):6		3.1							
TFDP1:6		1.4							
TWIST2:6		1.9	2.5		2.3		2.3		
ZEB1:6		2.0	2.4		2.2		2.0		
ZNF143:6		1.5	1.4						
ZNF384:6		1.6	2.2		1.6		2.0		
ARID5A:6			1.5				1.3		
BHLHE40:6			1.5						
CREB3:6			1.9				1.7		
DMBX1:6			1.4		1.4				
DMRT3:6			1.6						
EVX2:6			2.1				1.5	1.4	
FOXA2:6			1.5		1.8		2.0		
GLI2:6			2.2		2.0		1.4		
HAND1::TCF3:6			1.4						
HEY2:6			1.3						
HLTF:6			2.6		2.9		2.8		

HMX2:6			1.6						
HMX3:6			1.8						
HNF1A:6			1.6				1.7	1.6	
HOXA2:6			1.3					1.3	
HOXC10:6			2.0						
HOXC12:6			1.6						
HOXD11:6			1.4						
ID2:6			1.7		1.5				
IRF5:6			1.5						
IRF9:6			1.5		1.5		2.1		
KLF14:6			1.6		1.4		1.9		
LHX3:6			1.6		1.6		1.5		
MIX-A:6			1.5		1.4				
MLX:6			1.4						
MYBL1:6			1.5		1.6		1.8		
NFYA:6			2.6		1.7		1.5		1.4
NFYB:6			2.3		2.0		1.7	1.8	
ONECUT2:6			1.5						
PAX1:6			1.4						
POU4F3:6			2.4		2.0		1.8	1.6	
RAX:6			1.6				1.7		
SMAD3:6			1.8		1.9		2.2		
SOX15:6			1.8		2.0		2.2	1.5	
SOX9:6			1.4						
SPI1:6			1.8						
SREBF2(VAR.2):6			1.4						
STAT3:6			1.6				1.7	1.5	
TBX1:6			2.5		2.4		2.4	1.4	1.6
TBX4:6			2.5		2.4		2.4	1.4	1.6
TFAP2C(VAR.3):6			2.0				1.7		

YY1:6			1.9						
ZNF24:6			1.8		1.6				
EN2:6					1.5		1.4		
HNF1B:6					1.4		1.7		
FOXJ2:6							2.0		
HOXB5:6							1.5		
PAX5:6							1.6		
ZBTB7C:6							1.6		
FOXB1:6								1.5	
HOXA5:6								1.6	1.4
POU4F1:6								1.4	
RFX5:6								1.8	1.4

ALX1:7	1.6		1.6	1.6	1.6		1.3		
ALX3:7	1.6		1.4			1.5			
ARNT::HIF1A:7	2.1	1.6	1.7	1.8	1.7	2.7	2.2	2.4	2.8
ARNTL:7	1.3					1.6	1.7	1.6	2.4
ASCL1:7	2.1	2.2	1.6		1.5	3.2	1.8	1.8	2.6
ASCL2:7	1.8	1.8	1.4		1.6	2.8	2.0		1.6
ATF3:7	1.6		1.7	1.8	2.5	1.7	3.1	3.7	3.4
ATF4:7	2.1		1.7	1.4	1.8	1.9	2.0		
ATF7:7	2.2	2.1	2.0	1.9	2.4	2.9	3.8	1.7	2.8
ATOH1:7	1.3		1.4	1.6	2.1	1.7	2.0		1.3
BACH1::MAFK:7	2.7		1.4			2.7	1.9		1.7
BACH2:7	2.5		1.9	1.7	1.7	2.3	1.5		
BARHL1:7	1.3		1.5						
BARX1:7	2.3		1.8		1.4	1.5	1.7	1.7	2.0
BATF3:7	2.2	2.7	3.2	3.5	3.8	1.6	4.1	2.3	3.2
BATF::JUN:7	1.4	1.7	2.7	1.9	2.6	2.4	2.4	1.9	2.7
BCL6:7	2.1		1.6	2.4	1.6		1.8		1.9
BHLHA15:7	2.1		1.7		1.5	1.9	1.8	1.3	
BHLHE40:7	2.1	1.4	1.6		1.5	2.0	1.9		2.2

BHLHE41:7	1.4					1.7	1.7		1.9
BSX:7	2.2								
CEBPA:7	2.1		1.6	1.5	1.5	1.9	1.4		
CENPB:7	2.4	2.0	2.0		1.6	2.1	1.9	1.9	1.7
CLOCK:7	1.4							1.4	1.4
CREB1:7	2.6	2.8	2.5	2.9	3.6	3.9	3.5	1.5	3.3
CREB3:7	1.6							1.5	
CREB3L1:7	1.9		3.0		2.7		3.6	3.2	3.4
CREB5:7	2.5	3.5	4.1	3.8	5.3	3.3	5.4	1.9	3.1
CTCF:7	2.4	1.7	2.4	1.4	1.8	2.7	2.1	2.2	2.4
CTCFL:7	2.2	4.0	3.1	2.1	3.2	3.1	4.3	2.7	4.6
DDIT3::CEBPA:7	1.4		1.8		2.0	2.2	2.5	1.5	1.6
DLX2:7	2.3		1.5		1.7		1.6	1.5	1.8
DLX3:7	2.6				1.5	1.5			
DLX4:7	2.1				1.5	2.3			
DLX6:7	2.6		1.5	1.5	1.9	1.7			
DUX:7	1.8		1.6	1.3	1.4		1.4		
E2F3:7	2.0	2.0		1.7	1.8		1.6		1.6
E2F4:7	1.6	1.6	2.3		2.1	1.9	3.0	1.5	3.0
E2F6:7	1.9	2.1	3.8	3.5	3.6	3.4	3.1	1.4	2.8
E2F7:7	1.8		1.6		2.2	3.0	2.5		
E2F8:7	2.7	1.4	1.7	1.5	2.0	2.5	3.0	1.5	2.4
EGR1:7	1.7	2.1	1.7	2.2	2.1	1.4	2.6	2.3	3.1
EGR2:7	2.0	2.0	2.1	2.1	2.1		2.2	1.5	1.6
EGR3:7	4.0	4.0	3.9	4.2	4.1	2.5	5.7	2.4	3.5
EGR4:7	4.2	2.2	4.4	4.2	4.3	2.2	4.8	2.0	2.9
ELK4:7	1.5	2.1	3.1	1.8	3.8	4.0	5.7	2.3	3.3
EN2:7	1.4		1.7		1.7		2.0		1.4
EOMES:7	2.5	1.6	2.1		2.4	1.8	2.3	1.4	2.1
ESX1:7	1.9								
ETV6:7	2.0	1.7	2.6		2.8	1.9	1.8	2.0	2.0

EWSR1-FLI1:7	3.6	4.7	6.9	3.5	4.9	3.9	4.8	2.1	4.6
FOS:7	1.7		1.9		1.8	2.8	1.7	1.9	1.8
FOS::JUN:7	1.6		1.7		1.5	1.8	1.8		2.0
FOS::JUN(VAR.2):7	3.6	3.1	2.9	2.1	3.2	2.0	2.9	1.5	2.2
FOS::JUNB:7	1.8	1.4	2.0		2.4	2.8	2.3	1.8	2.6
FOS::JUND:7	1.9		2.5		2.3	2.2	2.2	1.5	1.8
FOSB::JUN:7	1.9	2.0	2.0	1.4	2.2	2.2	2.6		1.8
FOSB::JUNB(VAR.2):7	2.1	2.2	2.2		2.2	2.5	3.1		2.8
FOSL1:7	1.8	1.4	1.7	1.5	2.1	2.6	2.3		1.5
FOSL1::JUN:7	1.4		2.1		2.2	1.7	1.8	1.4	1.7
FOSL1::JUN(VAR.2):7	1.7	1.8	1.9	1.5	2.1	2.1	3.2	1.3	2.8
FOSL1::JUND:7	1.7		1.9		1.8	2.4	1.8		1.9
FOSL1::JUND(VAR.2):7	2.5	2.2	2.0	1.5	2.4	3.1	3.2	2.2	2.7
FOSL2::JUN:7	1.3		1.8		1.9	1.8	2.0		1.9
FOSL2::JUN(VAR.2):7	1.9	3.2	3.0	2.7	3.7	3.4	4.4	1.4	3.2
FOSL2::JUNB(VAR.2):7	2.0	2.5	2.8	1.9	2.9	3.3	3.7	1.3	3.1
FOSL2::JUND:7	1.4		1.8		1.5	2.3	1.7		1.7
FOSL2::JUND(VAR.2):7	2.0	2.8	1.7	1.8	2.1	3.1	3.0	1.8	2.8
FOXB1:7	2.1		2.5		2.5	1.6	2.8	1.4	2.5
FOXC1:7	2.4		2.2		1.8	2.5	1.9		2.0
FOXC2:7	2.4		1.4		1.3	1.8			
FOXD3:7	2.5		1.6		1.5	1.9	1.4		1.3
FOXH1:7	2.2	2.0	2.4	2.5	2.3	2.0	2.1		1.6
FOXJ3:7	1.7		2.3	1.6	2.1	1.8	1.4		
FOXK1:7	1.5		2.1		1.6	2.2	2.5	2.4	2.7
FOXO1:7	1.4		1.4			1.5			
GATA1::TAL1:7	1.7		1.6	1.5	1.5	1.4	2.6	1.5	2.5
GCM1:7	1.6		1.4				1.6		1.6
GCM2:7	1.7		3.7		2.8	2.5	4.0	2.9	4.0

GLI2:7	2.0	1.5	1.7	1.4	1.8	2.8	1.3		
GLIS1:7	1.3	1.3	2.0		1.7	1.6	2.5	2.4	2.5
GLIS2:7	2.5	2.7	3.3	2.3	4.0	2.3	3.5	2.6	4.0
GLIS3:7	2.3	3.4	4.2	1.8	3.5	2.2	4.3	2.4	2.8
GSX2:7	1.3								
HAND1::TCF3:7	2.4	2.7	3.0	1.6	3.0	2.9	3.6	3.2	4.6
HES1:7	2.7		2.1	2.4	2.6	2.9	4.4	1.9	4.7
HES2:7	1.3	1.4	2.0	2.2	2.8	2.7	3.7	1.6	2.5
HES5:7	1.8		1.4	1.5	1.9	1.5	2.3	1.9	3.3
HES7:7	1.4			1.8	1.5		1.7	1.6	2.1
HEY2:7	1.8		1.6	1.8	2.1	3.3	3.7	2.1	3.4
HIC1:7	2.1	1.5	1.9		1.7		1.3		
HIC2:7	1.6	1.5	3.6	2.1	2.8	2.5	2.4		1.9
HIF1A:7	2.8		2.0	2.2	2.5	2.9	2.4	1.6	2.3
HINFP:7	4.1	5.0	9.3	5.4	7.4	3.6	5.7	2.3	4.0
HMX1:7	1.4		1.4	1.6	1.7	1.9	1.8		1.8
HMX3:7	1.8		1.4		1.6		1.5		
HNF4A:7	1.5	1.5			1.3	1.7		1.4	
HOXC13:7	1.5		1.4		1.8		1.5		1.4
HOXD13:7	1.4					2.7	2.0		1.4
INSM1:7	2.8		1.8	1.5	2.4	2.4	3.6	2.0	2.8
IRF1:7	1.6		1.7	1.3	1.5	1.8	1.6	1.8	1.4
IRF3:7	1.9	1.8	2.9	1.3	2.7	2.3	2.7	1.6	2.3
IRF4:7	2.0		1.6		2.0	1.8	2.3	1.8	1.9
IRF9:7	1.5		1.8	2.2	2.2		1.7		
ISL2:7	2.7	1.8	1.7		1.8			1.6	2.0
ISX:7	1.8		1.4						
JDP2:7	1.4		1.4		1.3	1.7	1.8	1.6	2.0
JDP2(VAR.2):7	2.4	2.7	3.0	3.8	4.1	3.5	5.3	2.2	3.3
JUN:7	2.1	2.1	1.7	1.8	2.3	1.4	1.9		1.5

JUN(VAR.2):7	1.5		1.9	1.7	1.7	3.4	2.1		1.7
JUN::JUNB:7	1.7		1.5		1.7	2.2	1.6		
JUN::JUNB(VAR.2):7	2.8	3.9	4.7	3.0	4.8	2.4	5.4	3.7	5.1
JUNB:7	1.9		2.2	2.1	2.3	3.7	2.8		2.1
JUNB(VAR.2):7	1.7	1.7	1.8	1.7	2.2	3.4	3.0		2.1
JUND:7	1.7	1.5	2.3	1.9	2.3	4.0	3.1		1.8
JUND(VAR.2):7	1.4		2.1	2.2	2.5	1.7	2.0	1.4	2.3
KLF1:7	2.5	2.5	3.3	2.2	3.0	1.8	3.0	1.8	2.3
KLF12:7	2.6	3.2	3.7	2.6	3.4	2.5	3.3	2.5	3.1
KLF13:7	3.1	2.7	4.4	3.8	4.4	2.3	3.2	2.3	2.5
KLF14:7	3.4	2.2	3.7	2.4	3.6	2.0	3.1	2.3	3.0
KLF16:7	3.2	3.7	4.5	3.5	4.4	3.6	3.8	3.1	4.0
KLF4:7	2.2	2.5	3.3	1.6	3.5	2.6	4.2	2.7	4.0
KLF5:7	2.3	5.0	5.4	2.8	4.1	3.7	4.1	2.5	3.7
KLF9:7	1.8	3.4	3.2	2.5	3.7	3.9	3.2	2.9	3.2
LBX1:7	1.4		1.5	1.4	1.9		1.5		
LBX2:7	2.1		1.5						
LEF1:7	2.7	1.8	1.9	1.5	1.6	3.1	1.8	2.5	2.7
LHX9:7	1.8		1.4						
MAF::NFE2:7	2.2	1.5	2.0		1.6	1.8			1.4
MAFB:7	2.4		1.8	1.3					
MEF2B:7	1.5								1.3
MEIS1:7	2.4	1.5	2.0	2.4	2.7	1.9	2.8	2.1	2.8
MEIS2:7	4.0	2.2	4.1	3.4	4.2	5.3	4.0	3.2	3.2
MEIS3:7	4.2	2.2	4.4	3.3	4.2	4.8	4.8	3.7	3.9
MGA:7	2.9	2.4	2.5	1.9	2.7	2.0	3.1	2.5	3.4
MIXL1:7	2.4		1.4				1.5		
MSC:7	1.6					2.2	1.6	1.6	1.6
MSX1:7	3.1		1.6		1.4				
MSX2:7	3.6		1.8		1.6		1.3	1.4	1.9

MSX3:7	3.6		1.8		1.6		1.3	1.4	1.9
MTF1:7	1.8	1.7	1.8	2.6	1.8		2.0	1.5	2.0
MYBL1:7	1.8		1.5		1.8		1.8		2.0
MYOG:7	1.3					1.5	1.7		1.7
MZF1(VAR.2):7	3.7	2.0	4.5	3.8	4.9	5.3	4.8	2.1	5.2
NEUROD1:7	2.6	1.6	2.6	1.7	3.7	3.0	3.3	2.4	3.2
NEUROG2:7	1.3				1.6	1.4			
NFAT5:7	2.8		2.8	2.2	2.2	1.8	1.9	2.2	1.8
NFATC1:7	1.9		1.5		1.7	1.6	2.0	1.8	2.1
NFATC2:7	1.7		1.4			1.9	1.4	2.5	1.9
NFATC3:7	2.0		1.4	1.4	2.1	1.5	2.3	2.2	2.7
NFIA:7	1.5	2.1	1.4		1.6		1.5	1.6	
NFIC:7	1.3	2.4	2.3	2.1	2.2	1.3	1.8	1.9	2.1
NFIC::TLX1:7	2.2	2.2	4.4	2.7	3.8	3.7	3.1		2.9
NFKB1:7	1.7	1.4	2.6		2.5	3.2	2.6	1.4	2.7
NFYA:7	2.3	1.9	1.9	2.0	1.8	1.5	2.7	2.0	3.0
NFYB:7	3.6	1.5	2.8	1.7	2.6	1.7	3.6		3.3
NHLH1:7	1.6	1.7	2.1	1.4	2.7	2.4	3.8	1.9	2.8
NKX2-3:7	2.0	1.7	1.4	1.7	1.4	1.6	1.9	1.4	1.5
NKX2-5:7	2.4								
NKX2-5(VAR.2):7	1.7	1.4	1.6	2.1	1.7	1.5	1.7		1.8
NKX2-8:7	2.0	1.4	1.6	1.8	2.0	2.2	2.7	1.9	1.8
NKX3-1:7	2.0	1.6	1.7		1.6	1.7	1.6		1.3
NKX3-2:7	3.1	2.5	1.7		1.7	1.7	2.2		2.2
NOBOX:7	3.6		1.6		1.6	1.3	1.4		
NPAS2:7	1.4		1.7	1.4	1.6	1.5	1.9	1.4	2.0
NR1A4::RXRA:7	1.5		2.0		1.6		1.7		1.7
NR1H2::RXRA:7	2.1	1.3			1.4	1.5			
NR2C2:7	2.1	2.1	3.5	2.9	3.9	2.9	3.7	2.4	2.5
NR2E1:7	1.5								1.5

NR2F1:7	1.4	1.9	1.3	1.4					1.5
NR2F6:7	2.3		2.1	2.1	1.9	2.9	1.7		1.5
NR4A1:7	1.4	2.0		1.6			1.3		1.7
NR4A2:7	1.5	1.8		1.4		1.6			1.4
NR4A2::RXRA:7	1.8	1.7	2.8		2.3		1.6		2.0
NR5A2:7	1.8	1.5	1.4			1.8	1.5		
NRF1:7	4.2	5.0	5.8	5.8	7.2	2.7	7.3	3.6	6.9
NRL:7	1.5		1.4	1.9		1.4	1.7	1.4	2.4
OLIG3:7	1.3					1.4			
ONECUT1:7	1.5		1.4	2.1	1.3	2.3	2.2		1.8
ONECUT2:7	2.7		1.5	2.1	2.1	1.5	2.1		1.5
ONECUT3:7	1.9		1.5		1.5	1.4	2.1		1.4
OTX2:7	1.4	2.2	2.3	2.1	2.9	2.5	3.0	1.9	2.9
PAX1:7	1.3	1.7	1.6	2.2	2.3	2.1	2.0		2.0
PAX2:7	2.7	1.8	4.2	2.6	3.8	2.6	5.5		2.8
PAX5:7	2.6	2.5	2.9	1.5	2.5	2.2	1.8	2.2	1.7
PAX6:7	1.5						1.6		1.6
PAX9:7	2.1	2.0	2.1	2.3	2.7	2.9	3.6	1.5	2.6
PBX1:7	2.0	1.9	2.8		2.3	2.3	2.6		
PBX2:7	2.2	1.6	2.9	2.1	3.0	3.5	4.2		2.8
PBX3:7	3.1	2.4	4.4	2.1	3.5	3.8	3.7		2.7
PITX1:7	1.3		1.6		1.7	1.5	1.8	2.1	2.0
PKNOX2:7	1.3		1.4		1.5		1.6	1.3	1.7
PLAG1:7	3.4	3.5	5.3	2.3	3.4	2.2	3.8	2.1	3.1
POU2F3:7	1.4		1.8	2.1	2.2	1.8	1.7		
POU3F1:7	1.3	1.6	1.4		1.5	2.5	1.6	1.4	
POU3F4:7	2.5		1.8		1.7	2.4	1.7		
POU4F1:7	2.0					2.2	1.5		
POU4F2:7	1.6		1.7		2.2	2.3	2.2		1.7
POU4F3:7	2.3		1.7		1.9	2.2	2.3		1.9

POU5F1:7	2.4					1.8	1.8	1.3	2.0
POU5F1B:7	2.0		1.6		1.9	2.4			
POU6F2:7	2.5		2.2	1.7	2.3	2.2	2.1	1.3	
PPARA::RXRA:7	1.8	1.7	2.2	2.2	2.3		2.0	1.5	1.5
PPARG::RXRA:7	2.1	1.7	3.2	2.8	2.6	2.2	1.6	1.4	2.1
PRDM1:7	1.6								
PRRX1:7	1.8		1.4						
PRRX2:7	2.6		1.3						
RARA::RXRA:7	1.6		1.9		1.6	2.4	2.3		2.0
RAX:7	1.9		1.5		1.3				
RAX2:7	1.8		1.4						
RBPJ:7	1.8	2.0	1.9	1.8	2.5	4.0	3.8	2.5	3.7
RELA:7	1.4		1.5			1.7	1.8		2.1
REST:7	1.9	2.0	3.9	2.5	3.5	2.8	3.3	2.3	2.8
RFX3:7	1.9					1.4			
RFX4:7	1.8					1.7			
RFX5:7	1.4					1.4			
RHOX11:7	1.4					1.7			
RORA:7	1.7		1.4	1.6	1.3	1.7	1.5		2.0
RORB:7	1.8				1.5	2.4	2.4	2.5	2.7
RORC:7	2.3	1.6	2.8	2.1	2.6	2.8	2.9	1.7	2.1
RREB1:7	1.4	2.0	2.8	1.7	2.2	1.8	2.1	1.4	2.2
RUNX1:7	2.2		2.2	1.5	2.1	2.1	2.4	1.5	2.3
RUNX2:7	2.7	1.6	1.7		1.6	2.4	1.8		1.6
RUNX3:7	4.2	1.6	2.1	1.6	2.2	3.4	3.5	1.4	2.7
RXRA:7	1.8		1.4	1.8	1.4	1.9		1.3	
RXRB:7	1.6	1.3	1.6	1.7	1.4	1.8			1.4
RXRG:7	2.1	1.9	1.6	1.9	1.6				
SHOX:7	1.8		1.4						
SHOX2:7	1.8		1.4						
SIX3:7	2.1		1.3		1.5	1.4			

SMAD2::SMAD3::SMAD4:7	3.0	2.2	4.4	3.8	4.0	3.5	3.8	1.7	4.0
SMAD3:7	1.7	2.7	3.0	1.8	2.7	1.4	1.7		1.5
SOX10:7	1.4	1.6	1.6	1.9	1.6	1.6	1.7		
SOX15:7	1.4		1.5		1.5	1.6			1.4
SOX17:7	1.7		1.3		1.5	1.7	2.2	1.4	2.2
SOX2:7	1.7	1.8	1.9	1.5	1.7		1.5	2.0	1.4
SOX3:7	2.5	2.8	2.7	2.8	2.1	1.6	2.0	1.5	1.9
SOX4:7	1.6					1.3			
SOX6:7	1.5		1.5		1.6	1.5	1.5	1.4	1.7
SP1:7	2.5	4.2	5.8	3.8	5.1	3.4	5.7	2.3	4.1
SP2:7	3.6	5.0	6.9	3.8	5.4	3.9	7.3	2.5	4.6
SP3:7	3.6	5.0	5.6	3.0	5.0	3.9	4.8	2.9	3.4
SP4:7	2.7	3.4	3.7	2.5	4.0	2.7	3.2		2.6
SP8:7	4.0	3.1	5.0	2.8	5.0	2.3	4.1	2.4	4.0
SPDEF:7	1.4		1.6		1.7	1.8	2.4	2.3	2.0
SPI1:7	2.2	1.3	2.8	2.4	3.0	2.9	4.1	3.1	4.4
SPIB:7	1.9	2.0	1.8	1.3	1.5	2.4	1.5	1.4	
SPIC:7	1.4	2.5	1.7			1.4		1.8	1.7
SREBF1(VAR.2):7	1.5		1.4		1.7	2.7	3.0	2.4	3.2
SREBF2:7	2.0	2.0	2.9	2.0	2.7	3.4	3.3		2.8
SREBF2(VAR.2):7	1.6		1.4		1.4	2.9	2.5	2.3	2.8
SRY:7	1.7								
T:7	1.4		1.8	2.0	2.1	1.7	2.7	1.9	3.0
TBR1:7	2.8		2.2		2.3	1.8	2.6	1.4	2.4
TBX1:7	2.6	1.4	2.1	1.5	2.6		2.5	1.5	3.1
TBX15:7	3.6	2.0	2.5	2.1	2.7		3.1	3.0	3.2
TBX19:7	1.4	1.7	2.1	1.5	2.1	2.2	2.6	1.4	2.3
TBX2:7	1.8		1.6		1.6	1.6	1.9		2.1
TBX20:7	1.8						1.4	1.5	2.5

TBX21:7	1.4						1.9		1.8
TBX4:7	2.6	1.4	2.1	1.5	2.6		2.5	1.5	3.1
TBX5:7	1.4	1.6	2.4	1.8	2.6	1.4	3.8	2.4	3.7
TCF12:7	1.3	1.4	1.3		1.4	2.0	1.7		1.5
TCF7:7	1.4					1.9			2.0
TCF7L1:7	2.3		1.8		1.6	1.9	2.1		1.8
TCF7L2:7	1.4					1.8	1.6	1.3	2.0
TCFL5:7	1.8		1.4	2.2	1.9	2.5	1.9	1.5	2.0
TEAD3:7	1.6	1.6	1.8	1.5	1.6		1.7		
TEF:7	1.5					1.4	1.8	2.3	1.9
TFAP2A(VAR.2):7	1.5	2.2	2.9	3.3	3.7	2.9	4.1	1.5	3.2
TFAP2B(VAR.3):7	2.1		1.5	1.7	1.7	3.3	1.6	1.4	
TFAP2C:7	1.3	1.6	1.9	2.4	2.5	2.3	2.9	1.5	2.4
TFAP2C(VAR.3):7	1.9	1.5	1.5	1.6	1.6	2.7	1.4		
THAP1:7	4.0	2.0	3.7	2.4	3.7	3.5	5.5	1.8	4.6
TP53:7	2.0		2.1	1.8	2.9	2.1	2.3	1.9	2.1
TP63:7	1.7	2.5	2.3	1.4	2.5	1.9	2.3		1.3
UNCX:7	1.8		1.4						
VDR:7	1.4		1.9		2.6	1.6	3.0		1.5
VENTX:7	2.0		1.4		1.9		1.8		1.5
XBP1:7	1.5		2.1		1.7		1.5	1.9	
YY1:7	2.1	1.5	2.7	1.6	2.8	1.9	3.1	2.9	3.6
YY2:7	3.6	2.9	4.1	3.0	4.1	2.2	5.4	3.1	4.0
ZBED1:7	2.1	4.6	2.9	2.6	3.6	2.5	3.0	1.9	2.8
ZBTB18:7	1.9		1.8		1.4	1.4			
ZBTB33:7	2.1	1.8	2.4	3.7	3.0		2.8	1.6	2.5
ZBTB7A:7	1.5	2.5	4.4	1.8	4.1	2.5	4.3	3.1	4.1
ZBTB7B:7	1.8	2.1	2.2	1.8	2.3	3.5	2.3	1.4	1.7
ZBTB7C:7	2.3	2.8	4.0	2.4	4.2	3.4	3.7	1.8	2.9
ZFX:7	3.1	1.4	3.3	2.4	3.7	1.9	3.1	2.4	3.3

ZIC1:7	2.7	3.0	4.4	2.8	4.2	3.6	5.5	2.3	4.3
ZIC3:7	3.5	4.2	6.8	3.8	5.8	3.4	5.7	3.1	4.5
ZIC4:7	2.8	3.4	5.2	2.9	4.8	3.1	5.4	2.7	4.7
ZNF143:7	2.6	2.7	4.4	2.2	3.7	3.1	4.4	2.3	3.0
ZNF24:7	2.0	1.3				2.3	1.4		1.7
ZNF263:7	3.9	3.5	4.1	2.3	2.7	3.1	2.8	1.9	2.7
ZNF282:7	2.2	1.4	3.6	2.0	3.5	2.1	3.1	1.4	2.7
ZNF384:7	3.7	1.5	2.0	1.5	1.8	2.1	1.4		
ZNF740:7	5.2	4.6	5.3	3.8	4.4	3.9	5.2	2.5	3.5
DMBX1:7		1.8	1.4	1.4	1.8	3.4	2.9	1.9	3.1
E2F2:7		1.4	1.7	1.7	2.2	1.7	2.3		2.4
ELF1:7		2.0	2.4		2.3	1.4	3.2		1.8
ELF3:7		1.4	2.8		2.1		1.8	1.9	2.3
ELF4:7		1.3	3.3		2.5		2.7	2.2	2.6
ESRRB:7		1.8	1.7		1.5		1.5	1.6	2.2
ESRRG:7		1.5							
ETV2:7		1.6	2.3		2.8	1.3	3.0	1.6	1.8
FOSL1::JUNB:7		1.3	2.1	1.4	2.4	2.0	2.1	1.4	2.1
FOXD1:7		1.3	1.7		1.5	1.5	1.5		1.4
FOXD2:7		1.3	1.7		1.5	1.5	1.5		1.4
FOXG1:7		1.3	2.0		1.7	1.8	2.0	1.3	2.2
FOXI1:7		1.3	1.7		1.5	1.5	1.5		1.4
FOXL1:7		1.3	1.7		1.5	1.5	1.5		1.4
FOXO3:7		1.5	1.5		1.4	1.9	1.8		1.4
FOXO4:7		1.3	1.7		1.5	1.5	1.5		1.4
FOXO6:7		1.3	1.7		1.5	1.5	1.5		1.4
FOXP3:7		1.3	1.7		1.5	1.5	1.5		1.4

GABPA:7		1.9	3.0		3.6	2.6	3.9	2.4	3.4
GRHL2:7		1.4	1.6	1.9	2.0	2.4	2.0		1.6
MECOM:7		1.3	2.0	1.9	2.2	1.5	3.3		2.8
NFIX:7		1.4							
NR2F2:7		2.1	1.4	1.8		1.5	1.5		1.8
POU3F2:7		1.8				1.7		1.3	
POU3F3:7		1.4				1.8			
PROX1:7		1.6							
RARA(VAR.2):7		1.7	1.7	1.6	1.9				1.6
RARA::RXRG:7		1.8				1.5		1.7	
RORA(VAR.2):7		1.6	1.9		2.4	1.5	2.6	1.3	1.5
SMAD4:7		1.4	2.2	1.5	3.0	2.0	2.6		2.0
SOX13:7		1.7	2.9		2.6		2.4		1.7
SPZ1:7		1.4	1.6		1.5		2.0	1.9	2.7
TEAD4:7		1.6	1.8	1.4	1.8		1.4		
TFAP2B:7		1.6	1.9	2.5	2.4	2.7	3.1	1.5	2.6
ZNF410:7		1.3	2.2		1.9		3.0		2.5
AR:7			1.5		1.6	1.4	1.4		
ATF1:7			1.5	1.5	1.7		1.7		1.8
CDX2:7			1.5		1.6	1.8		1.4	
CREM:7			1.5	1.9	1.7	1.6	2.4	1.7	2.4
CUX1:7			1.7	1.5	1.6	1.4	1.7		1.8
CUX2:7			1.8	2.1	2.3	1.6	2.0		1.8
EHF:7			1.7		1.6		1.8		1.8
ELF5:7			2.9	1.4	2.7	1.6	3.3	2.3	3.4
ELK3:7			2.2		2.9	2.8	2.9	1.6	2.0
ERF:7			1.5		1.7		1.8		1.5

ERG:7			2.2		2.5	1.9	3.0	1.3	2.4
ESR2:7			1.9	1.3	1.3	2.4	2.2		3.3
ETS1:7			1.3		1.6		2.3	1.7	2.1
ETV4:7			1.4		1.7	2.0	1.7		1.9
FEV:7			2.3		3.2	2.6	3.1	1.5	2.4
FLI1:7			2.3		3.0	1.9	3.2	1.4	2.5
FOSB::JUNB:7			2.0		1.9	2.1	1.9		1.4
FOSL2:7			1.3			1.8			
FOSL2::JUNB:7			1.5		1.4	1.8	1.5		
FOXA2:7			1.5		1.3			1.3	1.5
FOXJ2:7			1.6		1.4		1.6	1.4	1.8
FOXK2:7			1.3		1.3	1.9	1.8		2.0
FOXP1:7			1.7		1.8	1.5	1.6	1.3	1.8
GBX1:7			1.3						
GBX2:7			1.7		1.7		1.8		
GFI1:7			1.6	1.7	1.6	2.5	1.7	1.3	1.8
GFI1B:7			1.8	1.4	2.2	3.4	2.6		2.3
GSC2:7			1.4	1.4	1.5	2.2	1.5	1.7	1.5
HNF4G:7			1.5	1.4	1.5	1.8	1.3	2.1	
HOXB13:7			1.8	2.1	2.3	3.7	2.8		1.8
MAFG:7			1.8	1.3	1.6		1.7		2.0
MAFK:7			2.0		1.7		1.5		1.4
MAX:7			1.3			2.0	1.8	1.9	2.1
MAX::MYC:7			1.6		1.5				
MLXIP:7			1.5				1.9	1.4	1.8
MXI1:7			1.7		2.0	2.8	2.2	2.2	3.1
MYOD1:7			1.3		1.5	1.9	1.3		
NFKB2:7			2.0		2.3	1.6	2.4	1.3	3.3
NKX6-1:7			1.4						
NR1H3::RXRA:7			1.7		1.8	2.0	2.6	2.3	2.9

NR2F6(VAR.2):7			1.4		1.6	2.9	1.8		1.5
PITX3:7			1.9		1.7	2.2	2.8	2.7	3.3
POU5F1::SOX2:7			1.3	1.5	1.7	1.9	1.8		
POU6F1:7			1.3		1.4				
PPARG:7			1.8	1.7	1.5	1.6	1.3		
RARB:7			1.3						1.5
RARG:7			1.4		1.6		1.3		
REL:7			1.4					1.5	1.3
RELB:7			1.4				1.7		1.4
RXRA::VDR:7			1.3		1.5		1.3		
SIX1:7			1.7	1.3	1.9	2.1	2.8		2.1
SIX2:7			2.5	1.9	2.4		2.4		1.7
SNAI2:7			1.4						
SOX9:7			1.9	1.7	1.6				
STAT1::STAT2:7			1.3				1.3	1.5	
STAT5A::STAT5B:7			1.4						
TCF3:7			1.4		1.4				
TCF4:7			1.5		1.6		1.3		
TEAD1:7			1.3		1.7				
TEAD2:7			1.5	1.3			1.5		
TFAP2A:7			1.5		1.9	1.9	2.8	1.7	2.1
TFAP2B(VAR.2):7			2.0	1.5	2.6	2.2	3.1		1.8
TFAP2C(VAR.2):7			1.5		1.9	1.7	1.9		1.4
TFDP1:7			2.0	1.4	2.3	3.1	4.0	1.4	2.9
TFEB:7			1.9	1.5	1.7	1.7	2.5	2.3	3.6
TP73:7			1.7		1.5		1.4		
TWIST1:7			1.5		1.9		1.8	1.5	1.8
TWIST2:7			1.7		1.7	2.6	1.5		
VSX1:7			1.4		1.6				
VSX2:7			1.4		1.6				

ZEB1:7			1.8		1.4	2.4	1.6		1.5
ZNF423:7			1.4		1.9	2.6	1.9	1.9	2.4
ZSCAN4:7			1.9		1.7	2.1	1.9		1.7
ARX:7				1.6	1.3				
ESR1:7				1.6			1.3		
FOXP2:7				1.4		1.6			1.6
GMEB1:7				1.8					
HNF1B:7				1.4	1.5	1.4	1.4	1.4	1.8
HOXA13:7				1.8	1.6	1.7	2.4		2.1
MYBL2:7				1.6					
RARB(VAR.2):7				1.4			1.3	2.0	2.0
EMX2:7					1.4				
ESRRA:7					1.4				
ETV1:7					1.4	1.3	1.3		1.6
FOXQ1:7					1.6		1.9		1.8
GSX1:7					1.4				
HNF1A:7					1.3	1.4	1.8		1.4
HOXA5:7					1.3	1.7	1.6		1.5
IRF2:7					1.3	1.5	1.4		1.4
MEF2A:7					1.4	1.5	1.3		1.9
MITF:7					1.5	3.4	2.7	1.5	2.2
MYC:7					1.3	1.7	1.5	1.4	2.0
MYF6:7					1.5		1.8		
NR1H4:7					1.6	1.7	1.6		
SCRT2:7					1.5	1.4	1.5		
STAT6:7					1.6		1.4		1.5
TCF21:7					1.4	1.6	1.8	1.3	1.7
TFAP2A(VAR.3):7					1.5	3.4	1.6		
TGIF1:7					1.4		1.8	1.8	2.2
BHLHE23:7						1.4			

E2F1:7						1.5			
ELK1:7						1.7	1.4		1.8
ETV5:7						1.4			
FOXF2:7						2.0		1.7	
GATA1:7						1.4			
GATA2:7						1.6			
GATA6:7						1.4			1.4
GSC:7						1.6	1.4	1.4	1.7
HEY1:7						2.7	2.1	1.9	2.8
HMX2:7						1.4	1.5		
HOXA9:7						1.6		1.4	
HOXC9:7						1.8			
HSF1:7						1.7	1.3		
HSF4:7						1.6	1.4		
ID4:7						1.6			
IRF5:7						1.5	1.3	1.4	
LIN54:7						2.3			
MAFF:7						1.4			
MLX:7						2.2			
MLXIPL:7						1.3			
MYB:7						1.6			
NEUROG1:7						1.5	1.6		1.4
NFE2:7						1.9	1.9		1.8
NFE2L2:7						3.4			
OLIG1:7						1.7	1.5		
OTX1:7						1.5		1.9	
PAX7:7						1.6	1.4		
POU1F1:7						2.7			
POU2F1:7						1.7			
RFX1:7						1.5			

SOX8:7						1.5			
SREBF1:7						2.5	1.9		1.8
STAT4:7						1.9			1.5
TFAP4:7						1.7	1.6		1.6
TFE3:7						1.3	1.5	1.5	
TFEC:7						1.9	1.7	1.8	2.6
USF1:7						2.5	1.7	2.3	2.1
USF2:7						2.3	2.3	1.7	2.1
ARID3B:7							1.4		
BARHL2:7							1.8		2.1
HOXC12:7							1.3		1.6
ID2:7							1.7	1.4	1.8
IRF8:7							1.3	1.5	
MYCN:7							1.6		
NR2E3:7							1.3		1.9
PKNOX1:7							1.5	1.9	1.8
RHOXF1:7							1.9	1.9	2.5
SOX1:7							1.4		
SOX11:7							1.4		
STAT3:7							1.5	1.6	1.4
TGIF2:7							1.5	1.7	2.1
CREB3L2:7								1.5	
DBP:7								2.3	1.5
FOXA1:7								1.4	2.0
HLF:7								1.6	
HMBOX1:7								1.9	2.0
HOXA10:7								1.4	2.0
STAT1:7								1.3	1.3
CDX1:7									1.4
EBF1:7									1.5

HOXD9:7									1.8
LHX3:7									1.4
NFIL3:7									1.4
POU2F2:7									1.5
RARG(VAR.2):7									1.9
TFCP2:7									1.4
ELK1:8	2.0	3.9	7.4	7.4	8.7	3.1	9.6	2.1	5.8
ELK3:8	1.7	3.9	5.8	6.4	6.6	2.4	7.8	2.1	5.7
ETV1:8	1.5	2.8	5.9	6.4	7.8	3.1	8.4	1.6	5.4
ETV4:8	2.0	3.9	6.9	7.3	8.6	2.9	8.7	1.8	6.0
ETV5:8	5.2	5.9	11.4	12.9	13.7	6.4	14.2	5.5	10.1
ELF1:8			1.5		1.9	1.4	1.9		
ETV3:8			1.8		1.6		1.8		
FEV:8			2.5	2.7	3.1		3.0		2.8
GABPA:8			1.9	2.5	3.4	1.7	2.8		1.5
ELF4:8							1.8		
ALX1:9	6.3	10.4	8.4	10.0	9.7	8.8	9.4	11.8	8.0
ALX3:9	7.1	9.3	8.6	13.4	10.5	8.6	8.1	8.4	5.4
ALX4:9	7.0	8.3	7.9	8.7	9.0	6.6	8.9	9.5	5.8
AR:9	4.2	3.6	5.3	3.2	5.1	1.6	4.9	1.4	1.5
ARID3B:9	2.7	3.6	5.2	6.3	6.2	4.2	6.2	5.9	5.5
ARID5A:9	3.1	3.3	3.0	2.3	4.1	6.7	3.3	3.1	1.9
ARNT::HIF1A:9	2.2	4.0	3.3	4.6	4.6	6.1	5.1	3.2	3.5
ARNTL:9	2.7	2.3	2.6	2.6	3.1	2.0	3.3	3.0	2.0
ARX:9	7.1	4.7	6.3	6.9	6.1	7.0	5.5	9.3	4.9

ASCL1:9	11.0	13.1	12.1	14.9	13.0	10.7	14.1	19.5	13.5
ASCL2:9	7.7	8.3	9.9	10.8	9.7	9.6	11.4	11.7	9.4
ATF3:9	2.2	3.2	2.9	2.6	2.8	2.6	3.2	2.5	2.1
ATF4:9	2.9	3.4	3.5	4.2	3.4	2.6	4.2	5.5	3.2
ATF7:9	2.3		3.1	4.0	4.0		3.6		1.8
ATOH1:9	4.7	5.7	5.7	8.0	5.2	3.1	3.7	3.6	2.4
BACH1::MAFK:9	6.6	7.1	9.0	10.9	10.2	6.7	9.6	8.1	6.2
BACH2:9	3.2	5.3	6.0	8.5	6.6	6.2	7.2	6.5	4.7
BARHL1:9	3.9	6.1	6.6	11.1	8.2	9.0	8.0	6.2	4.8
BARHL2:9	1.7	2.9	2.8	5.6	3.2	4.8	4.4	4.1	3.5
BARX1:9	5.1	5.5	6.3	11.9	8.6	8.3	9.7	8.9	5.9
BATF::JUN:9	3.9	8.8	6.0	9.7	6.5	4.7	4.9	5.8	4.1
BCL6B:9	1.7	3.2	3.2	3.5	3.1	5.0	3.0	5.2	3.7
BHLHA15:9	3.0	5.6	3.6	8.3	6.2	5.6	6.3	7.9	7.0
BHLHE22:9	2.6	3.2	2.6	4.4	3.8	2.0	3.0	2.9	2.9
BHLHE23:9	2.0	2.0	2.9	4.3	3.6	2.0	3.4	3.6	3.3
BHLHE40:9	3.0	2.5	2.1	2.4	2.7	3.6	2.9	5.3	2.4
BHLHE41:9	2.2		2.0	2.1	2.0		2.1	3.1	
BSX:9	4.1	6.6	5.2	9.2	7.3	8.1	8.7	5.7	4.4
CDX1:9	4.5	5.4	7.7	8.0	9.0	7.4	7.8	6.7	6.1
CDX2:9	7.4	6.0	7.9	7.7	10.2	10.5	11.2	7.7	9.2
CEBPA:9	2.8	2.4	4.6	5.5	5.0	3.8	5.2	5.8	4.4
CLOCK:9	1.9	3.2	1.6	2.6	3.0	3.7	4.7	5.4	4.6
CREB1:9	2.5	1.5	2.1	4.7	3.9	2.0	3.7	1.9	2.7
CREB3L1:9	2.8	2.3	1.7	2.5	2.3	1.5	1.9	2.2	1.6
CRX:9	8.5	8.0	9.7	10.7	10.0	7.7	12.0	11.2	8.3

CTCF:9	2.2	2.3	3.2	4.4	4.1		3.3	3.3	3.0
CTCFL:9	3.7	2.9	5.0	4.4	5.8	3.7	5.1	2.7	3.7
CUX1:9	3.2	4.0	3.1	7.0	4.9	4.0	6.4	6.9	6.5
CUX2:9	2.8	3.1	2.5	4.0	3.4	3.7	5.3	6.0	4.8
DDIT3::CEBPA:9	8.5	6.0	6.1	5.4	6.7	3.9	5.6	6.5	4.5
DLX1:9	3.3	7.9	6.0	7.2	5.9	7.5	6.5	6.3	5.1
DLX2:9	4.7	4.2	6.3	7.9	7.4	10.1	9.5	6.8	5.7
DLX3:9	2.7	3.7	3.6	8.1	5.6	5.8	6.7	5.4	4.1
DLX4:9	2.5	5.0	2.8	7.5	5.1	5.2	5.8	5.7	3.7
DLX6:9	4.1	4.1	5.1	7.1	6.1	5.6	7.3	5.4	3.9
DMBX1:9	5.6	3.9	5.2	8.2	4.9	3.0	5.0	7.0	3.8
DMRT3:9	1.6	2.5	2.7	3.8	3.5	2.8	3.2	2.3	2.1
DUX:9	3.8	5.7	6.2	6.6	7.8	7.1	8.9	6.5	5.6
DUX4:9	6.2	4.1	6.6	8.2	6.1	4.4	5.8	7.6	6.2
DUXA:9	5.2	3.7	7.6	8.1	7.5	5.4	8.7	7.0	6.9
E2F3:9	1.3			3.4	2.3	3.0	2.6	5.7	4.5
E2F4:9	4.1	3.9	3.5	4.0	4.7	7.1	3.6	5.9	3.0
E2F6:9	8.2	12.4	14.5	16.5	19.5	9.7	21.1	18.9	15.5
E2F7:9	2.9	2.8	4.4	6.0	6.2	5.5	7.0	3.9	2.5
E2F8:9	3.9	5.1	4.4	3.7	4.6	3.2	4.7	7.4	4.4
EBF1:9	2.6	5.5	4.7	5.2	4.4	2.3	4.1	3.5	2.7
EGR1:9	8.6	5.3	6.9	5.8	6.0	3.1	5.4	5.2	3.3
EGR2:9	7.1	8.8	8.5	9.3	10.2	7.3	10.8	9.3	8.3
EGR3:9	9.3	8.1	9.1	10.6	12.3	11.5	11.7	9.6	5.3
EGR4:9	11.2	13.1	14.5	12.3	17.3	12.0	17.2	12.7	11.9
EMX1:9	4.9	5.6	4.1	6.8	6.2	7.5	4.9	5.5	4.3
EMX2:9	3.9	5.2	4.8	5.0	4.9	6.3	5.7	4.8	4.0
EN1:9	4.2	6.2	5.4	8.4	5.9	8.9	7.3	6.4	4.2
EN2:9	3.2	8.1	7.4	7.5	7.7	8.7	7.5	7.6	7.0

EOMES:9	4.7	8.3	10.1	11.8	9.2	7.2	9.8	9.2	5.5
ESR1:9	1.3	2.2	1.3	2.2	2.1	3.0	2.7	2.7	2.5
ESR2:9	3.7	4.9	6.0	6.9	7.7	6.7	6.1	6.7	3.7
ESRRA:9	4.9	6.8	7.4	5.2	7.7	6.9	8.0	9.5	6.5
ESRRB:9	3.6	3.1	5.2	4.5	6.3	6.7	5.9	5.3	4.9
ESRRG:9	5.7	5.2	7.4	5.7	8.2	6.1	7.2	8.6	6.5
ESX1:9	3.4	10.0	7.4	12.2	10.5	7.7	9.9	7.6	5.0
EVX1:9	5.6	8.1	4.6	8.2	7.3	6.1	6.6	8.1	6.2
EVX2:9	6.3	7.2	4.8	6.3	6.2	6.4	5.7	8.0	5.8
EWSR1-FLI1:9	16.1	13.5	16.2	21.2	22.4	15.0	21.2	18.4	13.9
FIGLA:9	3.0	3.9	4.4	5.1	5.1	3.1	6.7	4.3	4.5
FOS:9	3.9	6.8	5.3	11.4	7.1	3.0	5.8	4.5	4.2
FOS::JUN:9	2.6	4.1	4.4	9.0	4.4	3.5	4.9	6.0	3.8
FOS::JUN(VAR.2):9	2.1		1.9	3.2	2.1	1.8	1.4	1.5	
FOS::JUNB:9	2.0	3.8	3.9	7.3	4.2	2.9	3.6	3.3	2.1
FOS::JUND:9	4.4	5.2	5.7	11.9	6.1	3.2	5.4	5.1	3.3
FOSB::JUN:9	1.8		2.5	3.1	3.2	1.9	3.4		1.9
FOSB::JUNB:9	2.4	3.9	4.1	7.5	4.3	4.2	4.6	5.9	3.4
FOSB::JUNB(VAR.2):9	2.4		2.5	3.2	3.0		2.9		1.3
FOSL1:9	3.1	6.4	5.0	9.7	5.4	2.1	4.8	4.0	3.2
FOSL1::JUN:9	2.4	4.3	4.1	8.8	4.4	3.0	4.7	5.8	4.1
FOSL1::JUN(VAR.2):9	2.6	1.7	3.2	3.9	4.1	1.7	3.5		1.7
FOSL1::JUNB:9	2.2	3.5	4.0	7.3	4.4	2.9	4.2	4.5	2.4
FOSL1::JUND:9	2.8	4.0	4.3	8.3	4.4	3.0	5.1	6.4	4.0
FOSL2:9	3.4	7.6	6.8	8.7	8.7	4.3	8.6	5.1	6.1
FOSL2::JUN:9	2.6	4.2	4.8	9.6	4.9	2.2	5.1	6.1	4.1
FOSL2::JUN(VAR.2):9	1.8		2.0	3.2	2.9	1.8	3.0	2.1	1.6
FOSL2::JUNB:9	2.0	3.1	4.5	7.2	4.7	3.9	4.6	5.9	3.0
FOSL2::JUNB(VAR.2):9	1.6	1.8	3.1	4.3	4.0	1.4	3.1		
FOSL2::JUND:9	1.9	3.4	4.5	8.1	4.7	3.7	4.6	5.5	2.7
FOSL2::JUND(VAR.2):9	2.5	1.7	4.2	4.8	5.5	2.1	4.4	2.1	3.2

FOXA1:9	5.7	5.0	7.9	5.5	7.8	5.6	7.6	6.2	4.4
FOXA2:9	5.4	5.2	6.0	5.3	6.3	5.4	6.5	4.1	3.3
FOXB1:9	2.5	2.7	2.6	4.1	3.7	3.7	3.0	4.5	
FOXC1:9	2.1	2.5	1.4	3.3	2.7	3.9	3.2	5.4	2.7
FOXC2:9	2.4	3.6	2.7	4.0	3.7	6.4	5.2	5.4	4.0
FOXD1:9	2.5	1.9	2.7	2.4	2.9	1.4	2.1	2.6	1.4
FOXD2:9	2.5	2.0	2.8	2.4	3.0	1.4	2.1	2.6	1.4
FOXD3:9	4.9	5.3	5.4	6.5	4.4	4.9	4.3	7.4	4.0
FOXF2:9	4.2	1.9	5.2	2.7	5.4	3.6	4.2	5.7	2.9
FOXG1:9	3.0	2.1	2.9	4.2	3.7	2.4	4.0	1.9	2.0
FOXH1:9	4.4	4.6	6.8	8.3	7.8	6.6	8.5	7.6	6.3
FOXI1:9	2.5	2.0	2.8	2.4	3.0	1.4	2.1	2.6	1.4
FOXJ2:9	3.0	1.3	2.5	2.7	3.3	3.0	4.3	1.6	1.6
FOXJ3:9	4.0	3.3	4.2	4.6	5.5	3.9	4.3	3.5	2.1
FOXK1:9	3.3	3.6	3.6	4.6	4.5	2.9	3.0	2.2	
FOXK2:9	2.9	1.9	3.8	3.1	4.7	1.8	3.4	4.1	2.9
FOXL1:9	2.5	2.0	2.8	2.4	3.0	1.4	2.1	2.6	1.4
FOXO1:9	4.5	4.6	8.8	9.6	10.3	4.7	9.4	7.5	6.6
FOXO3:9	2.6	2.1	3.2	2.4	3.9	2.5	2.9	3.0	1.8
FOXO4:9	2.5	2.0	2.8	2.4	3.0	1.4	2.1	2.6	1.4
FOXO6:9	2.5	2.0	2.8	2.4	3.0	1.4	2.1	2.6	1.4
FOXP1:9	4.0	3.3	5.5	5.5	5.8	2.0	3.8	3.2	
FOXP2:9	3.2	2.7	4.8	7.0	6.1	4.4	5.5	5.8	4.3
FOXP3:9	2.5	2.0	2.8	2.4	3.0	1.4	2.1	2.6	1.4
FOXQ1:9	1.6	4.0	2.8	3.7	3.7	2.5	3.0	2.7	2.0
GATA1:9	4.4	6.1	5.5	7.5	7.1	2.6	6.4	6.6	4.9

GATA1::TAL1:9	5.4	9.8	10.1	9.7	13.3	6.0	11.5	9.4	7.2
GATA2:9	2.1	4.1	4.4	5.1	5.2	3.2	4.9	4.6	4.0
GATA3:9	3.1	3.7	3.6	6.1	5.1	3.9	4.1	3.3	1.6
GATA4:9	3.6	5.4	6.3	5.5	7.9	2.7	7.3	3.9	3.5
GATA5:9	3.6	4.9	5.6	5.8	5.6	3.2	4.1	3.2	2.5
GATA6:9	3.3	5.9	6.1	5.9	6.3	2.3	6.6	5.0	4.0
GBX1:9	2.9	6.5	5.8	6.3	6.1	6.4	5.7	7.5	3.6
GBX2:9	2.6	6.7	5.2	8.5	5.8	8.3	7.0	6.4	3.9
GCM1:9	1.6	3.2	2.5	3.3	2.8	3.7	3.8	4.9	3.0
GCM2:9	2.3			1.7			1.8	2.9	1.6
GFI1:9	9.0	5.7	10.4	12.3	11.3	7.4	11.3	11.5	7.9
GFI1B:9	8.5	9.5	12.1	12.9	14.8	9.5	14.0	12.9	10.0
GLI2:9	2.7	6.2	4.3	4.6	5.7	4.2	4.0	4.5	3.1
GLIS1:9	4.4	3.1	5.1	5.7	4.7	4.2	5.4	7.1	4.6
GLIS2:9	7.1	8.8	6.1	10.3	8.3	6.4	7.9	7.2	6.1
GLIS3:9	6.3	6.5	3.6	7.2	5.6	5.8	6.0	6.5	4.2
GRHL2:9	2.6	5.4	4.7	3.8	4.2	3.1	4.9	3.5	2.9
GSC:9	5.8	4.6	3.8	5.3	3.0	3.8	4.4	3.8	3.2
GSC2:9	8.5	6.1	5.7	5.8	4.9	5.7	6.0	5.8	3.9
GSX1:9	5.7	7.0	5.7	9.1	7.6	8.8	7.8	7.9	4.6
GSX2:9	4.4	8.0	6.7	10.4	9.5	9.3	11.0	10.4	6.8
HAND1::TCF3:9	5.4	5.7	4.3	7.1	4.6	5.2	5.6	7.3	4.6
HES1:9	6.2	9.6	9.7	8.2	11.5	5.7	11.7	6.7	8.9
HES2:9	1.6	2.3		3.1	2.6	4.3	3.0	4.6	3.1
HES5:9	3.0	4.0	2.1	3.1	3.3	3.2	3.6	4.1	3.5
HES7:9	3.7	3.6	2.9	3.7	3.9	4.0	5.8	3.7	4.9
HESX1:9	3.6	8.9	6.2	8.8	7.7	7.4	7.0	7.0	4.6
HEY1:9	3.0	3.2	4.0	4.8	5.5	3.7	4.9	3.8	4.1
HEY2:9	3.2	3.4	2.7	7.2	4.2	2.3	4.0	5.0	3.7
HIC1:9	5.6	4.9	6.3	11.1	7.9	6.1	6.9	8.9	6.4

HIC2:9	4.4	4.4	4.7	6.9	6.7	4.9	8.5	5.9	5.7
HIF1A:9	1.3	3.7	4.3	4.9	5.4	6.0	6.7	3.5	4.4
HINFP:9	2.4	6.2	2.0	3.2	2.0	3.2	2.7	3.6	2.9
HMX1:9	4.5	4.6	8.6	12.3	11.2	8.9	12.4	8.5	6.2
HMX2:9	2.6	4.3	5.6	6.3	7.0	5.9	8.0	6.3	3.3
HMX3:9	3.1	4.9	6.7	8.5	8.7	6.5	9.9	7.7	5.2
HNF1A:9	2.8	3.1	3.2	3.2	2.9	3.3	3.4	3.4	2.9
HNF1B:9	2.4	2.6	2.6	2.1	2.2	2.6	3.1	2.5	1.5
HNF4A:9	3.0		2.7	4.0	3.5	3.6	4.3	3.7	3.4
HNF4G:9	2.8	4.6	4.9	5.6	4.6	6.3	4.6	5.9	3.9
HOXA10:9	4.9	6.4	8.2	7.3	8.4	4.5	8.1	5.6	6.1
HOXA11:9	2.5			2.4	1.6	2.0	1.8	1.3	
HOXA13:9	3.9	6.5	8.7	7.6	8.3	3.8	6.9	7.9	5.7
HOXA2:9	4.5	6.3	5.6	5.6	6.3	7.4	7.2	10.0	6.6
HOXA5:9	2.1	4.1	4.4	6.3	5.7	4.1	5.4	5.2	3.2
HOXA9:9	8.8	9.5	10.2	10.1	10.2	4.2	8.4	6.4	3.7
HOXB13:9	7.6	6.8	10.1	8.0	10.1	5.2	9.9	9.8	7.0
HOXB2:9	2.5	4.7	3.1	5.4	4.0	5.7	4.1	4.9	3.4
HOXB3:9	4.0	9.3	5.3	8.8	6.4	7.3	5.7	7.6	5.5
HOXB5:9	5.5	7.6	6.3	7.6	8.6	7.5	6.1	9.0	4.5
HOXC10:9	3.6					1.6			
HOXC11:9	3.5	2.1	2.3	2.4	2.8	2.4	1.9		
HOXC12:9	1.8	2.6	2.7	4.6	4.0	3.0	3.0		
HOXC13:9	3.8	2.9	2.8	4.2	3.7	3.0	4.0	2.9	2.2
HOXC9:9	6.4	4.1	5.3	6.4	6.6	4.4	5.6	5.8	3.3
HOXD11:9	2.8	2.1	1.7	3.4	2.4	2.1	1.9		1.3
HOXD12:9	3.1		1.9	2.1	2.4	3.4	3.1		1.6
HOXD13:9	6.0	6.2	6.6	7.7	8.0	5.0	6.8	9.9	6.5

HOXD3:9	5.3	8.6	8.4	11.2	10.1	7.7	8.5	6.6	5.8
HOXD8:9	3.5	5.5	6.6	7.2	6.9	6.5	7.8	5.9	4.9
HOXD9:9	3.6	3.1	6.3	8.0	7.5	3.4	6.1	5.5	5.9
HSF4:9	1.5	2.3	1.5	3.4	1.6	3.1	1.5		
ID2:9	2.3	3.2	1.6	2.4	3.1	4.5	3.5	4.2	2.9
ID4:9	5.5	6.7	8.3	10.0	8.4	7.2	7.6	7.7	6.9
INSM1:9	4.1	9.2	4.7	5.0	4.9	3.2	3.6	6.6	3.6
IRF1:9	5.3	7.5	6.7	8.0	7.7	5.9	7.2	9.5	5.4
IRF2:9	3.3	4.1	5.0	6.7	6.1	3.8	4.0	4.2	3.8
IRF3:9	6.1	6.2	7.0	10.8	8.1	6.8	8.4	8.6	5.5
IRF4:9	2.0	5.3	3.4	4.0	4.1	3.2	3.8		2.4
IRF5:9	2.2	4.1	3.5	6.4	5.3	3.2	4.9	1.6	2.9
IRF7:9	5.9	5.9	6.6	8.2	8.4	5.4	6.6	7.4	5.9
IRF8:9	3.5	6.6	4.6	5.1	4.9	4.0	5.6	2.7	3.7
IRF9:9	3.6	7.1	4.1	6.2	5.2	5.4	6.9	5.7	6.0
ISL2:9	5.2	2.8	7.5	8.5	8.4	8.1	9.7	8.6	5.5
ISX:9	4.6	7.9	5.7	11.0	8.4	9.2	9.7	8.6	5.3
JDP2:9	4.6	3.9	6.0	9.1	6.9	5.9	5.3	5.8	4.9
JDP2(VAR.2):9	2.6	1.6	4.0	4.6	5.0	2.9	4.0	2.0	1.5
JUN:9	4.8	3.0	4.8	6.7	5.9	4.6	6.6	7.2	6.0
JUN(VAR.2):9	3.6	7.4	6.6	9.9	8.1	4.1	7.8	6.5	5.5
JUN::JUNB:9	3.1	3.4	4.2	9.4	5.1	3.7	6.5	7.7	6.0
JUN::JUNB(VAR.2):9	1.5		2.0	3.8	3.2	1.6	2.9		1.6
JUNB:9	3.2	6.5	6.0	8.9	6.9	3.5	6.5	5.1	5.1
JUNB(VAR.2):9	3.2	1.7	2.7	3.4	3.1		2.6		
JUND:9	5.8	6.6	6.3	10.4	7.1	4.5	6.5	5.4	4.8
JUND(VAR.2):9	4.9	3.0	6.2	7.0	7.0	3.7	6.6	6.0	4.6
KLF1:9	2.6	3.7	3.0	6.4	3.5	3.2	3.5	2.7	3.4
KLF14:9	3.9	4.5	2.8	3.2	3.0	2.6	3.6	4.8	3.5

KLF16:9	8.8	8.3	9.8	11.1	10.1	8.6	10.7	7.4	6.4
KLF4:9	2.7	3.2	3.9	5.8	5.1	3.6	4.4	7.5	4.9
KLF5:9	9.2	8.3	8.2	14.9	11.5	10.5	12.5	10.8	9.0
KLF9:9	10.4	13.1	10.1	12.4	11.9	11.0	12.4	11.6	9.2
LBX1:9	5.3	6.0	5.3	9.6	8.0	7.6	7.8	6.3	4.2
LBX2:9	4.7	7.9	5.6	10.2	7.5	8.3	9.0	8.6	5.3
LEF1:9	5.9	8.3	7.8	9.2	9.9	10.4	7.8	8.2	4.1
LHX2:9	4.4	9.8	5.5	7.2	7.1	8.1	7.6	8.7	5.3
LHX3:9	6.6	3.9	7.0	6.5	6.8	3.9	5.4	5.5	3.1
LHX4:9	6.4	7.0	9.3	6.7	8.8	5.8	7.5	7.6	6.4
LHX6:9	6.2	6.8	6.4	6.4	7.2	9.1	8.5	9.0	6.1
LHX8:9	5.7	6.6	5.2	4.8	4.9	10.7	5.8	7.5	4.3
LHX9:9	4.6	7.9	5.7	11.0	8.4	9.2	9.7	8.6	5.3
LIN54:9	6.4	6.4	7.9	7.6	7.6	4.6	4.7	5.4	3.6
LMX1A:9	7.1	8.6	10.2	7.2	10.1	8.1	8.4	6.8	6.0
LMX1B:9	7.1	8.6	10.2	7.2	10.1	8.1	8.4	6.8	6.0
MAF::NFE2:9	3.5	6.6	9.9	10.4	10.1	9.6	9.7	8.6	7.0
MAFB:9	5.5	7.1	6.2	8.2	8.1	9.3	7.3	9.5	6.2
MAFF:9	6.0	6.6	7.0	9.4	7.9	4.6	6.3	6.7	4.6
MAFG:9	3.0	5.8	3.1	6.1	3.1	3.6	3.0	4.2	4.1
MAFK:9	4.1	6.3	4.8	7.0	5.3	6.2	6.0	5.5	4.0
MAX:9	1.9	3.3	1.8	2.5	3.0	1.8	3.1	4.7	3.1
MAX::MYC:9	3.8	2.7	2.7	3.3	2.7	3.3	3.5	2.7	2.4
MECOM:9	4.1	5.4	6.7	8.7	8.1	5.0	7.7	6.8	5.0
MEF2A:9	4.3	3.6	4.3	2.8	4.4	2.9	3.9	5.1	2.7
MEF2B:9	2.3	3.2	3.0	3.1	3.6	4.8	3.3	2.1	2.0
MEF2C:9	4.4	4.4	6.2	5.5	6.2	5.2	5.9	7.7	7.1
MEF2D:9	5.3	4.6	5.5	3.7	6.2	4.5	5.0	5.4	3.4
MEIS1:9	7.1	6.9	7.7	8.7	6.2	6.8	7.4	6.2	5.1

MEIS2:9	8.5	5.8	8.9	12.4	10.1	6.4	9.1	10.3	8.1
MEIS3:9	9.4	6.0	9.0	11.1	9.4	6.4	8.0	9.0	6.9
MEOX1:9	4.5	8.0	7.0	8.6	8.1	5.5	6.1	9.3	4.5
MEOX2:9	4.5	7.6	6.6	6.8	7.9	6.2	5.4	4.9	3.2
MGA:9	4.5	5.6	9.1	10.7	9.9	3.2	7.2	10.4	7.8
MIX-A:9	6.2	9.5	11.0	9.6	10.2	7.4	9.9	8.9	7.1
MIXL1:9	7.6	9.1	7.5	14.1	9.3	10.0	8.5	11.0	6.5
MLX:9	1.5								
MLXIP:9	2.3	3.7	1.7	2.5	3.2	5.0	3.3	4.8	3.3
MNX1:9	6.2	5.9	7.8	8.5	8.2	8.0	9.5	8.5	5.8
MSC:9	4.4	5.1	5.0	6.3	4.8	3.6	5.5	8.3	5.7
MSX1:9	4.0	4.9	6.1	11.5	8.1	8.9	9.2	6.5	6.0
MSX2:9	4.3	3.9	5.3	10.7	6.8	7.5	8.3	7.5	5.1
MSX3:9	4.3	3.9	5.3	10.7	6.8	7.5	8.3	7.5	5.1
MTF1:9	4.5	6.8	6.5	5.5	6.7	4.1	6.6	5.3	3.2
MXI1:9	4.4	3.6	3.7	3.5	3.5	2.7	3.3	4.6	3.0
MYB:9	5.6	3.5	5.7	7.1	7.5	4.6	9.0	10.7	7.2
MYC:9	5.4	5.3	5.7	6.3	5.3	3.9	6.4	7.1	5.8
MYCN:9	2.4	1.8	1.6	2.9	2.1	2.6	2.7	2.0	1.4
MYF6:9	3.4	2.7	2.6	4.8	3.5	1.9	2.7	6.2	3.4
MYOD1:9	6.9	8.6	8.0	8.3	7.8	8.0	8.7	8.5	8.2
MYOG:9	9.7	10.9	11.0	10.2	10.6	8.1	10.5	11.5	10.0
MZF1(VAR.2):9	8.5	13.1	10.5	12.6	10.3	9.8	12.0	10.5	8.1
NEUROD1:9	7.1	8.6	8.8	11.8	10.2	7.5	11.6	11.3	9.9
NEUROD2:9	1.7	2.9	3.1	3.1	3.4	1.7	2.4	2.5	1.7
NEUROG1:9	3.7	5.1	4.6	5.8	5.0	1.9	2.4	4.2	3.7
NEUROG2:9	1.6	3.4	2.8	2.8	2.5	3.5	3.1	4.9	2.0
NFAT5:9	8.4	7.4	11.6	12.3	11.5	5.2	9.3	10.6	7.0
NFATC1:9	5.5	3.5	7.6	9.0	7.2	6.0	6.4	9.5	4.5
NFATC2:9	7.4	6.6	10.5	11.1	11.9	7.5	12.1	8.9	9.9

NFATC3:9	5.9	4.7	8.6	9.0	7.9	5.1	7.7	7.9	4.9
NFE2:9	2.6	4.2	5.0	6.3	5.5	4.1	5.2	4.2	3.7
NFE2L2:9	5.8	8.0	12.1	10.1	11.9	7.6	10.3	11.6	8.3
NFIA:9	4.7	3.0	3.2	3.5	3.4	5.4	4.9	8.6	4.5
NFIC:9	5.4	4.3	6.0	6.5	5.8	4.5	9.0	12.1	7.1
NFIC::TLX1:9	7.1	5.7	6.6	5.9	6.3	3.7	5.0	5.6	4.5
NFIL3:9	1.4		2.5	2.4	3.7	1.9	4.4	4.1	2.3
NFIX:9	5.2	7.3	4.7	4.8	5.0	6.1	6.3	8.6	4.2
NFKB1:9	4.3	5.2	5.5	4.4	4.7	5.4	5.6	7.0	4.4
NFYA:9	2.4	2.8	4.8	4.5	4.8	4.4	5.7	4.4	3.8
NFYB:9	3.9	3.6	5.8	6.9	6.5	4.1	8.2	5.8	6.1
NHLH1:9	5.3	8.3	7.7	11.0	6.3	8.6	8.2	10.9	8.3
NKX2-3:9	5.5	4.0	6.1	8.7	7.9	4.4	8.8	6.6	5.4
NKX2-5:9	5.0	3.3	5.0	6.3	7.0	6.1	8.3	9.2	4.2
NKX2-5(VAR.2):9	5.7	9.1	9.3	9.2	10.0	8.9	10.8	8.5	5.9
NKX2-8:9	9.2	4.8	10.1	9.0	11.9	5.2	11.3	11.0	8.9
NKX3-1:9	4.7	3.4	4.9	6.0	5.0	5.1	4.6	7.0	3.1
NKX3-2:9	4.2	3.6	4.6	7.2	5.6	5.6	5.8	7.4	4.3
NKX6-1:9	3.8	7.7	4.8	5.7	5.8	7.2	4.6	4.8	3.7
NKX6-2:9	4.4	8.9	6.4	9.4	9.3	7.2	7.9	6.4	5.6
NOBOX:9	1.6	4.6	3.3	5.1	4.5	8.7	5.5	4.8	3.9
NOTO:9	5.4	7.1	5.2	6.2	5.6	6.9	5.8	6.5	3.9
NPAS2:9	2.9	2.3	2.0	2.4	2.9	2.7	2.6	3.1	2.2
NR1A4::RXRA:9	3.3	5.2	2.4	5.3	3.1	2.3	3.1	2.8	2.9
NR1H2::RXRA:9	1.6	2.2	2.0	3.3	3.3	4.8	2.9	3.0	3.5
NR1H3::RXRA:9	2.4	4.3	2.0	4.2	1.8	2.3	1.7	5.0	1.7
NR1H4:9	3.8	6.7	3.3	5.6	4.7	4.5	3.0	3.6	3.5
NR2C2:9	9.2	9.8	9.9	12.1	10.7	9.8	10.3	9.3	6.3

NR2E1:9	3.8	3.2	3.8	6.1	4.2	5.8	5.4	3.9	3.1
NR2E3:9	4.9	4.4	3.1	3.9	3.3	2.1	3.4	2.6	1.6
NR2F1:9	2.2	1.8	2.9	1.6	2.9	2.4	2.7	4.6	2.4
NR2F2:9	3.3	3.6	4.3	4.3	5.1	4.4	3.7	3.6	2.2
NR2F6:9	1.8	2.5	3.3	6.7	5.2	4.1	5.3	4.9	3.2
NR2F6(VAR.2):9	3.3	2.3	2.5	3.3	2.1	4.0	2.3	2.3	2.0
NR3C1:9	2.2	3.1	4.7	3.0	4.6		5.1	3.3	2.5
NR3C2:9	2.7	3.5	5.0	4.8	4.3	2.3	6.2	4.9	2.8
NR4A1:9	2.8	3.1	3.6	5.9	3.6	3.5	2.2	2.7	
NR4A2:9	3.9	3.1	5.3	6.6	6.8	3.7	5.0	5.2	3.9
NR4A2::RXRA:9	1.6	3.5	1.7	3.9	1.9	2.7	1.7		
NR5A2:9	3.3	5.9	5.7	6.3	6.0	6.2	5.1	5.9	4.6
NRF1:9	9.0	7.8	8.4	8.3	8.6	6.7	9.2	6.9	6.2
NRL:9	8.0	8.5	10.2	10.3	11.8	8.8	11.0	12.8	6.9
OLIG1:9	2.2	3.2	2.5	5.5	3.5		2.5	3.2	2.0
OLIG2:9	3.1	2.6	3.3	4.3	3.0	1.8	2.9	2.8	2.5
OLIG3:9	3.6	3.1	3.6	4.4	3.2	1.4	2.8	3.4	3.2
ONECUT1:9	6.0	7.7	7.8	9.7	10.8	5.4	10.2	9.4	7.5
ONECUT2:9	5.0	6.3	6.2	7.2	8.1	6.2	9.0	6.7	5.5
ONECUT3:9	6.2	6.8	6.6	7.7	6.8	5.9	8.1	7.3	4.8
OTX1:9	7.3	3.2	4.5	5.4	3.5	3.8	4.7	3.1	2.5
OTX2:9	3.8	2.3	1.7	4.2	1.8	3.7	1.8	3.8	
PAX1:9	4.6	5.0	8.0	8.3	8.5	5.6	9.3	6.7	7.3
PAX2:9	2.2	1.5	1.7	5.2	2.1	1.8	2.1	2.1	
PAX3:9	3.9	4.9	5.3	8.3	6.7	4.8	5.5	2.8	2.5
PAX4:9	2.9	6.2	3.6	5.9	4.6	6.8	5.0	6.1	3.1
PAX5:9	2.5	3.7	2.6	4.1	2.7	3.3	3.4	4.1	1.9
PAX6:9	5.2	6.5	5.3	7.4	6.2	4.4	5.4	10.4	6.6
PAX7:9	3.8	6.2	5.8	7.4	6.9	6.0	6.6	5.0	4.1
PAX9:9	5.4	6.4	7.8	8.2	8.2	3.2	7.6	7.0	5.5
PBX1:9	5.9	6.5	7.2	7.3	8.6	10.2	8.7	9.4	6.3

PBX2:9	7.1	8.7	8.7	10.5	9.0	5.7	8.0	11.6	7.3
PBX3:9	12.8	15.8	20.0	21.4	22.5	12.0	21.3	17.4	14.2
PDX1:9	3.6	4.9	6.3	5.4	5.6	8.0	7.3	4.7	4.8
PHOX2A:9	2.3	3.7	5.6	4.8	6.4	3.7	6.6	6.6	4.1
PHOX2B:9	2.6	3.9	6.1	5.2	6.2	3.9	7.1	5.8	3.6
PITX1:9	4.4	4.4	4.6	6.6	4.2	2.7	4.1	5.6	4.3
PITX3:9	5.9	4.9	3.3	5.9	3.4	3.1	4.5	5.0	3.2
PKNOX1:9	4.3	3.7	4.7	5.7	5.1	3.5	3.2	7.0	3.5
PKNOX2:9	5.2	7.3	5.4	8.1	5.2	6.0	5.1	8.6	5.4
PLAG1:9	6.6	8.5	6.4	11.5	9.4	8.9	9.2	10.8	8.7
POU1F1:9	4.1	6.0	6.3	8.3	5.9	5.4	6.3	5.5	4.3
POU2F1:9	4.1	7.0	6.0	6.3	4.7	3.9	4.4	7.9	3.8
POU2F2:9	9.2	8.2	9.7	10.3	9.0	6.2	8.4	10.9	8.2
POU2F3:9	6.5	6.7	6.8	8.2	8.2	12.3	9.8	9.3	6.6
POU3F1:9	3.5	6.0	7.1	9.2	6.9	6.1	6.9	7.6	4.8
POU3F2:9	4.0	7.1	6.3	9.1	7.2	7.4	7.3	6.7	5.3
POU3F3:9	5.3	4.3	6.3	8.5	6.3	7.4	7.0	6.8	5.0
POU3F4:9	4.5	5.1	5.7	7.2	5.0	4.0	5.7	10.5	6.5
POU4F1:9	5.4	7.2	9.1	11.1	10.7	6.8	11.1	8.1	7.0
POU4F2:9	8.5	7.2	9.8	11.3	10.1	7.5	9.9	7.6	7.0
POU4F3:9	7.6	7.2	10.5	11.1	12.5	6.0	11.3	9.4	7.4
POU5F1:9	5.4	8.2	8.8	8.5	9.0	8.3	9.6	12.1	8.2
POU5F1::SOX2:9	6.3	7.4	7.3	9.0	9.0	6.6	9.9	10.0	7.0
POU5F1B:9	3.6	6.5	6.1	8.3	5.9	5.7	6.2	10.1	5.4
POU6F1:9	5.0	4.5	5.6	4.1	6.6	6.6	7.9	8.2	4.9
POU6F2:9	7.2	8.2	8.7	10.0	9.4	6.0	8.4	7.9	4.9
PPARA::RXRA:9	2.2	1.6	1.8	3.8	2.0	3.5	2.0	4.2	
PPARG:9	4.4	3.4	4.2	5.0	4.9	6.5	6.3	5.3	3.3
PPARG::RXRA:9	3.6	8.3	6.6	10.9	9.8	9.5	10.2	10.0	9.2

PRDM1:9	5.6	8.0	9.8	14.0	12.7	7.8	11.6	7.6	8.8
PROP1:9	3.3	3.5	4.4	3.4	5.2	3.0	6.0	3.8	3.7
PROX1:9	2.6	2.5	2.8	4.0	2.8	4.5	4.9	3.7	3.9
PRRX1:9	4.6	7.9	5.7	11.0	8.4	9.2	9.7	8.6	5.3
PRRX2:9	3.5	5.6	4.7	10.0	6.6	8.1	8.0	7.6	4.4
RARA:9	5.6	4.9	5.8	8.2	6.6	5.7	6.2	7.0	4.3
RARA(VAR.2):9	4.8	5.3	6.4	7.8	7.1	6.1	7.6	6.2	5.3
RARA::RXRA:9	6.6	10.2	7.0	8.5	7.6	8.9	7.8	7.9	5.7
RARA::RXRG:9	5.9	5.0	6.6	9.5	6.7	8.3	7.4	8.5	5.8
RARB:9	4.5	3.1	2.6	4.2	3.3	5.4	3.3	5.0	2.6
RARB(VAR.2):9	3.4	1.9	2.8	2.8	3.0	3.8	2.9	4.9	3.2
RARG:9	3.7	2.3	3.2	4.1	3.4	5.4	5.5	5.5	3.3
RARG(VAR.2):9	4.6	3.9	3.4	7.3	5.1	3.9	5.0	5.1	3.4
RAX:9	4.6	8.8	6.1	9.7	7.9	9.2	8.0	7.8	4.9
RAX2:9	4.6	7.9	5.7	11.0	8.4	9.2	9.7	8.6	5.3
RBPJ:9	5.1	4.1	6.7	7.3	7.6	4.8	7.2	6.8	5.5
REL:9	1.8	3.2	2.7	2.3	2.4	1.5	2.0	3.8	2.0
RELA:9	3.9	4.2	5.9	6.4	7.5	3.4	7.3	8.1	7.0
RELB:9	5.2	9.1	9.7	3.9	7.8	4.7	5.4	6.5	3.5
REST:9	6.7	5.3	6.0	7.3	7.6	8.3	9.9	11.8	8.9
RFX1:9	3.6	4.8	4.8	6.0	6.2	3.8	7.4	6.9	5.0
RFX2:9	3.3	5.5	3.4	4.9	2.8	3.1	3.4	3.5	1.5
RFX3:9	3.1	6.0	4.8	6.3	3.6	3.9	4.2	4.7	2.1
RFX4:9	3.4	5.1	4.8	6.4	4.2	3.8	4.7	6.3	3.7
RFX5:9	2.6	5.9	4.6	5.7	4.2	3.7	3.9	4.3	2.2
RHOX11:9	3.0	6.4	5.0	4.4	5.9	2.7	5.1	6.7	3.7
RHOXF1:9	3.8	4.7	4.7	6.3	3.2	2.1	3.0	2.3	2.8
RORA:9	6.2	6.1	6.3	5.8	5.5	7.8	5.2	3.1	2.7
RORA(VAR.2):9	2.7	2.9	2.3	2.9	3.0	3.1	2.0	3.1	2.0
RORB:9	5.0	5.4	5.4	6.0	5.0	3.5	4.2	4.0	3.7
RORC:9	3.4	4.6	3.3	3.7	3.4	2.8	3.0	2.5	2.4

RREB1:9	8.1	11.0	10.2	13.5	11.8	12.3	12.5	11.3	9.5
RUNX1:9	3.4	6.7	6.8	8.3	9.2	7.6	7.4	9.3	5.2
RUNX3:9	1.4		1.5	2.1	2.1	1.9	2.3	5.3	2.8
RXRA:9	2.6	2.6	4.0	4.6	5.7	5.5	5.5	3.8	3.7
RXRB:9	1.5	3.9	3.9	5.1	5.1	6.9	5.6	3.7	3.9
RXRG:9	2.7	2.9	4.2	4.8	5.5	6.6	6.5	6.2	6.0
SCRT1:9	2.0	3.0	3.9	5.4	4.3	5.2	4.7	4.5	3.5
SHOX:9	4.6	7.9	5.7	11.0	8.4	9.2	9.7	8.6	5.3
SHOX2:9	4.6	7.9	5.7	11.0	8.4	9.2	9.7	8.6	5.3
SIX1:9	4.1	4.7	6.2	4.5	5.1	4.5	4.7	7.9	4.1
SIX2:9	8.5	7.2	7.4	7.1	7.2	8.8	10.7	8.3	8.3
SMAD2::SMAD3::SMAD4:9	8.8	13.3	11.7	12.8	13.1	11.2	14.1	17.1	11.2
SMAD4:9	3.1	2.6	4.3	3.9	5.1	2.6	5.6	4.3	3.2
SNAI2:9	6.3	8.6	9.3	11.1	10.2	6.3	9.6	9.6	6.0
SOX1:9	5.3	3.7	4.2	4.8	5.6	6.1	5.6	5.2	4.1
SOX10:9	4.4	5.4	5.1	7.1	5.6	6.1	4.9	6.2	4.9
SOX11:9	6.1	8.5	6.8	10.1	8.4	5.0	8.1	7.6	6.0
SOX13:9	2.1	4.4	5.6	4.0	6.2	4.8	6.2	4.6	6.5
SOX15:9	6.1	5.0	6.7	7.6	8.4	3.9	7.6	7.4	5.7
SOX17:9	6.0	6.7	8.1	10.2	9.0	4.2	9.0	7.2	5.6
SOX2:9	3.6	4.5	5.2	6.7	5.9	5.0	5.5	4.8	4.5
SOX21:9	5.4	3.7	5.4	6.3	7.3	3.5	6.2	7.0	5.5
SOX3:9	6.8	9.8	9.5	8.3	9.6	5.1	9.5	11.6	7.3
SOX4:9	5.7	8.6	8.1	10.2	9.3	6.7	9.1	7.8	6.0
SOX6:9	5.2	6.9	7.4	8.4	8.4	5.4	7.9	5.4	5.7
SOX8:9	4.2	5.1	7.6	7.2	8.0	4.6	7.7	11.3	7.1
SOX9:9	3.6	5.3	6.4	5.4	6.2	5.8	6.7	6.4	3.9
SP1:9	14.5	12.9	13.9	18.6	15.7	10.4	17.6	17.0	11.4
SP2:9	11.5	9.7	9.9	16.6	11.9	12.0	12.4	12.2	9.2

SP3:9	7.4	8.4	6.9	10.3	8.2	6.8	9.4	5.4	4.3
SP4:9	4.9	4.0	3.6	5.9	4.5	2.6	5.3	4.8	4.4
SP8:9	6.3	5.5	8.1	11.1	8.9	6.6	9.3	7.8	5.6
SPI1:9	4.5	4.2	3.0	6.3	4.2	3.0	3.8	2.8	2.3
SPIB:9	7.1	7.9	10.9	13.4	13.3	12.3	13.9	11.6	10.1
SPIC:9	2.0	3.2	5.0	4.4	6.2	4.2	7.6	4.3	5.0
SPZ1:9	3.2	5.1	3.9	3.0	4.9	4.3	5.1	3.7	2.3
SREBF1:9	6.4	4.7	3.2	7.1	4.4	4.8	5.3	7.9	6.0
SREBF2:9	8.4	5.3	6.5	8.2	8.2	6.6	9.9	9.7	8.0
SREBF2(VAR.2):9	1.4			1.7					
SRF:9	3.0	3.2	2.4	2.8	2.7	2.3	3.4	2.5	3.3
SRY:9	4.4	4.6	5.7	6.2	7.3	3.4	6.6	4.6	3.7
STAT1:9	2.3	3.4	3.3	4.5	4.0	4.6	4.1	2.1	2.4
STAT1::STAT2:9	5.6	8.5	5.8	6.9	6.5	6.6	5.7	9.3	5.2
STAT3:9	5.7	4.1	4.9	6.8	6.4	5.3	4.9	2.7	2.3
STAT5A::STAT5B:9	1.9	2.4	1.9	4.4	2.7	4.2	2.4		
STAT6:9	4.6	3.9	4.7	7.9	6.2	3.5	5.8	4.2	4.0
T:9	3.8	7.3	5.9	7.3	6.2	3.0	5.6	2.2	3.3
TAL1::TCF3:9	3.2	4.6	4.1	7.1	3.6	3.3	3.4	5.4	3.8
TBP:9	2.6	3.5	2.6	2.1	3.3	4.3	4.7	4.2	4.0
TBR1:9	3.3	9.8	7.6	12.1	8.7	7.5	9.0	7.0	5.7
TBX1:9	4.1	6.7	6.9	8.3	7.7	3.7	6.1	5.7	2.7
TBX15:9	2.7	4.3	7.0	9.0	8.0	4.4	7.3	6.5	4.9
TBX19:9	3.3	6.0	4.8	7.0	5.0	1.9	5.2	4.2	5.1
TBX2:9	4.6	8.1	9.9	12.2	10.5	4.9	9.4	8.0	6.2
TBX20:9	3.2	8.3	8.9	9.8	8.4	8.2	7.9	9.8	7.0
TBX21:9	3.8	7.6	8.6	8.0	8.1	6.3	8.4	6.2	4.6
TBX4:9	4.1	6.7	6.9	8.3	7.7	3.7	6.1	5.7	2.7
TBX5:9	3.4	4.5	7.9	7.7	7.3	4.6	5.9	6.4	3.5
TCF12:9	8.4	10.1	10.3	9.9	10.3	7.4	10.3	11.4	10.1

TCF21:9	5.7	4.2	3.4	6.4	4.1	5.4	4.1	6.1	5.7
TCF3:9	5.3	5.8	6.6	6.9	6.3	3.3	6.8	6.4	6.1
TCF4:9	6.6	6.3	8.3	8.2	8.1	5.5	8.3	6.5	7.4
TCF7:9	5.3	7.5	7.5	7.3	8.6	9.7	8.3	6.9	3.5
TCF7L1:9	3.3	6.2	6.6	10.9	8.8	7.4	8.4	9.2	5.9
TCF7L2:9	6.0	7.3	10.1	11.6	10.8	9.5	12.4	10.2	6.6
TCFL5:9	8.0	6.6	6.0	3.2	5.4	2.1	4.3	3.7	3.2
TEAD1:9	2.8	3.0	3.3	4.0	3.0	4.6	4.3	4.2	3.8
TEAD4:9	1.3	3.4	3.1	3.5	2.7	2.3	4.1	3.5	3.0
TFAP2A:9	5.6	7.5	4.4	3.2	4.4	4.0	5.0	5.5	3.9
TFAP2A(VAR.2):9	6.3	7.2	6.7	6.5	6.8	8.6	7.9	6.4	5.4
TFAP2A(VAR.3):9	1.7	2.9	1.7			1.7		2.3	
TFAP2B:9	5.7	7.1	6.6	6.0	6.9	7.8	7.8	5.4	4.5
TFAP2B(VAR.2):9	3.9	3.7	2.5	2.4	2.5	2.1	3.6	6.5	3.9
TFAP2C:9	4.7	7.0	6.1	5.7	6.1	6.7	6.9	5.7	5.0
TFAP2C(VAR.2):9	5.2	4.9	4.3	4.1	4.1	3.4	4.8	6.5	4.2
TFAP2C(VAR.3):9	1.6	2.4	1.7	1.7		1.4	1.5	2.3	
TFAP4:9	3.8	8.4	7.0	5.6	6.7	5.4	8.3	5.8	4.7
TFCP2:9	1.9	1.4		2.0	2.3	2.2	2.7		1.7
TFDP1:9	5.8	2.5	3.3	5.9	4.4	5.4	4.7	6.5	4.1
TFE3:9	1.6					1.3	1.6	1.4	
TFEB:9	2.3	2.5	2.8	1.7	2.5	1.3	1.7		
TFEC:9	3.3	2.3	3.7	2.2	2.8	3.1	3.1	2.0	1.8
TGIF1:9	5.7	5.4	4.5	8.6	5.7	5.0	4.0	7.6	3.9
TGIF2:9	5.4	6.5	6.0	8.3	7.2	4.6	4.7	6.4	3.5
THAP1:9	5.9	4.5	5.0	7.0	6.7	5.3	6.3	7.2	5.2
TP53:9	3.9	5.5	3.9	3.2	4.2	4.2	3.1	2.5	1.7
TP63:9	3.1	2.2		1.5	1.5	2.9	1.7	2.9	1.9
TP73:9	2.8	3.2	1.6	2.0	2.1	2.8	1.4	3.6	1.9
TWIST1:9	3.2	8.1	5.9	7.0	6.7	4.2	7.5	10.5	7.6
TWIST2:9	2.7	4.2	3.2	6.1	3.5	2.0	2.6	5.1	3.1

UNCX:9	4.6	7.9	5.7	11.0	8.4	9.2	9.7	8.6	5.3
USF1:9	4.6	1.8	4.3	5.0	4.0	3.9	4.4	3.7	3.1
USF2:9	2.6	2.5	2.8	2.6	2.4		3.0	2.8	2.5
VAX1:9	4.6	4.7	5.3	5.0	5.5	8.1	6.3	6.1	4.8
VAX2:9	3.6	4.9	4.2	3.9	3.8	6.9	4.1	4.1	3.7
VENTX:9	1.7	2.5	2.1	2.9	3.0	5.0	3.3	4.1	2.1
VSX1:9	5.3	7.5	5.4	8.0	6.7	8.1	6.2	6.1	4.1
VSX2:9	5.3	7.5	5.4	8.0	6.7	8.1	6.2	6.1	4.1
YY1:9	5.9	8.0	9.1	7.9	8.0	5.5	7.5	5.8	6.1
YY2:9	2.1	1.3	1.4		1.4				1.4
ZBED1:9	2.8				1.5			2.5	
ZBTB18:9	4.6	6.9	6.3	8.4	7.9	5.3	9.0	11.2	8.7
ZBTB33:9	2.7		1.9	2.1	2.1		1.4	2.5	
ZBTB7B:9	2.4	3.2	2.9	4.9	3.4	3.6	3.9	4.2	3.1
ZBTB7C:9	1.6	2.8	2.2	4.3	4.2	3.6	4.6	5.5	3.4
ZEB1:9	8.6	7.6	10.9	9.5	10.2	7.1	9.9	9.1	7.1
ZFX:9	1.5	2.4		2.4		4.0	1.4	3.2	2.0
ZIC1:9	3.5	6.7	6.8	7.9	7.4	7.9	7.1	9.9	5.2
ZIC3:9	4.6	4.5	5.0	8.2	6.9	6.8	6.6	7.6	4.4
ZIC4:9	3.2	4.9	5.2	7.3	6.3	7.8	7.1	8.0	4.9
ZNF143:9	4.8	8.6	8.0	9.1	7.7	6.0	6.6	6.4	5.4
ZNF24:9	11.5	13.5	14.7	16.0	16.8	8.9	12.1	9.7	6.9
ZNF263:9	16.9	19.5	22.4	23.3	25.1	13.0	22.1	27.0	18.5
ZNF282:9	3.3	9.2	4.6	7.8	4.5	3.7	6.6	8.7	7.0
ZNF384:9	3.8	5.0	5.8	6.9	5.4	6.0	6.7	9.5	5.2
ZNF410:9	4.1	2.8	2.9	2.3	3.4	4.3	3.7	1.8	1.6
ZNF423:9	2.1	1.4	2.2	2.0	2.0	1.8	1.6	3.6	2.0
ZNF740:9	11.5	10.4	12.6	14.0	13.8	10.0	15.0	12.2	10.8
ZSCAN4:9	12.8	13.5	16.0	17.9	17.7	12.3	18.7	18.4	14.2

BCL6:9		1.7		1.8	1.4	2.0	1.6	2.6	1.3
CEBPB:9		1.5					1.5		
CEBPD:9		1.4					1.4		
CEBPE:9		1.7	1.5	1.7	1.6		1.9		
CEBPG:9		1.3					1.5		
CREB3L2:9		1.7		2.6	2.4		1.7	1.8	
CREB5:9		1.6	2.6	3.5	3.3	2.1	2.6		
E2F1:9		1.5				1.6		2.8	
GRHL1:9		1.5	2.0	2.9	1.8	2.7			
HLF:9		1.3	1.6	1.8	1.6		2.3	2.4	
HLTF:9		2.0		2.6		2.2	1.4	2.1	
KLF12:9		4.2	1.3	1.3	1.8	1.5			
KLF13:9		2.3	1.4			2.1	2.3	1.9	1.8
MYBL2:9		3.4	1.9	2.7	2.3	1.4	1.9		
NFKB2:9		4.5	2.4	1.6	2.1	3.8	2.4	4.8	3.3
RUNX2:9		1.9	1.7	2.0	2.6	1.5	2.2	3.7	2.1
SCRT2:9		3.9	1.5	3.0	2.1	3.8	2.5	1.9	
SIX3:9		2.5	2.3	4.8	2.9	1.5	3.0	5.0	2.6
STAT4:9		4.6	2.2	4.9	3.6	4.3	4.1	4.2	3.5
TEAD2:9		3.0	1.5	3.6	2.2	1.6	2.6	3.4	3.3
TEAD3:9		2.1	2.3	3.5	2.1	2.1	2.5	5.5	2.7
TFAP2B(VAR.3):9		1.4	1.3	1.4		1.5		2.1	
VDR:9		2.8	2.1	3.4				3.0	
XBP1:9		1.4		1.7	1.4		1.5	1.7	
DBP:9			1.4	1.7	1.8		2.0	1.9	
MITF:9			1.8		1.7		2.6		
MLXIPL:9			1.4		1.6	1.9	2.2	2.2	1.4
CENPB:9				1.5	1.6		1.6	1.7	1.7
E2F2:9				3.1	1.8	3.3	2.5	5.1	3.4
FOSL1::JUND(VAR.2):9				2.5				1.4	

GMEB1:9				1.4			1.5	1.6	
HMBOX1:9				1.3					
HSF2:9				2.7		2.1		1.7	1.6
SREBF1(VAR.2):9				1.4		1.5		1.9	
ATF1:9						2.0			
HSF1:9						1.6			1.6
SMAD3:9							1.9		
CREB3:9								2.2	
TEF:9								1.4	

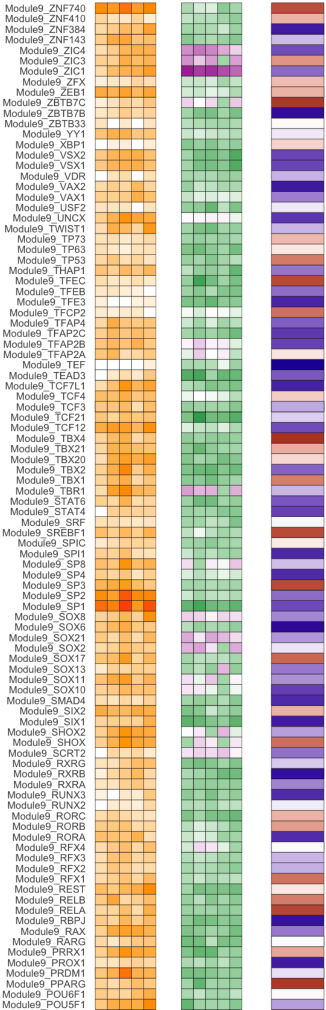

Additional file 2: Fig. S3

Heatmap matrices of enrichment, expression and Pearson correlation between the two for each motif, across brain tissue

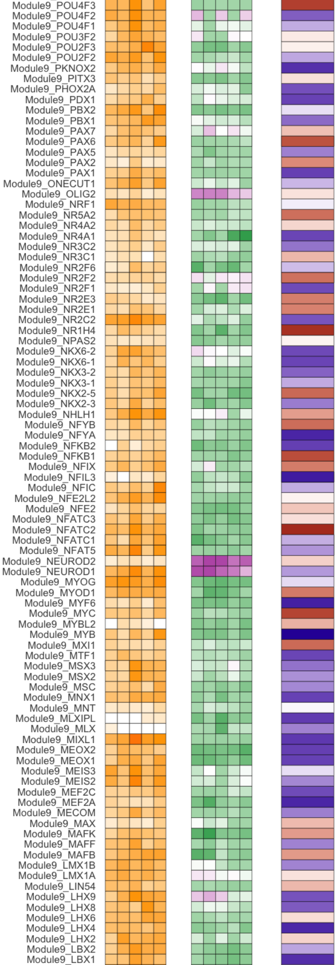
Module9_POU4F3
Module9_POU4F2
Module9_POU4F1
Module9_POU3F2
Module9_POU2F3
Module9_POU2F2
Module9_PKNOX2
Module9_PITX3
Module9_PHOX2A
Module9_PDX1
Module9_PBX2
Module9_PBX1
Module9_PAX7
Module9_PAX6
Module9_PAX5
Module9_PAX2
Module9_PAX1
Module9_ONECUT1
Module9_OLIG2
Module9_NRF1
Module9_NR5A2
Module9_NR4A2
Module9_NR4A1
Module9_NR3C2
Module9_NR3C1
Module9_NR2F6
Module9_NR2F2
Module9_NR2F1
Module9_NR2E3
Module9_NR2E1
Module9_NR2C2
Module9_NR1H4
Module9_NPAS2
Module9_NKX6-2
Module9_NKX6-1
Module9_NKX3-2
Module9_NKX3-1
Module9_NKX2-5
Module9_NKX2-3
Module9_NHLH1
Module9_NFYB
Module9_NFYA
Module9_NFKB2
Module9_NFKB1
Module9_NFIX
Module9_NFIL3
Module9_NFIC
Module9_NFE2L2
Module9_NFE2
Module9_NFATC3
Module9_NFATC2
Module9_NFATC1
Module9_NFAT5
Module9_NEUROD2
Module9_NEUROD1
Module9_MYOG
Module9_MYOD1
Module9_MYF6
Module9_MYC
Module9_MYBL2
Module9_MYB
Module9_MXI1
Module9_MTF1
Module9_MSX3
Module9_MSX2
Module9_MSC
Module9_MNX1
Module9_MNT
Module9_MLXIPL
Module9_MLX
Module9_MIXL1
Module9_MEOX2
Module9_MEOX1
Module9_MEIS3
Module9_MEIS2
Module9_MEF2C
Module9_MEF2A
Module9_MECOM
Module9_MAX
Module9_MAFK
Module9_MAFF
Module9_MAFB
Module9_LMX1B
Module9_LMX1A
Module9_LIN54
Module9_LHX9
Module9_LHX8
Module9_LHX6
Module9_LHX4
Module9_LHX2
Module9_LBX2
Module9_LBX1

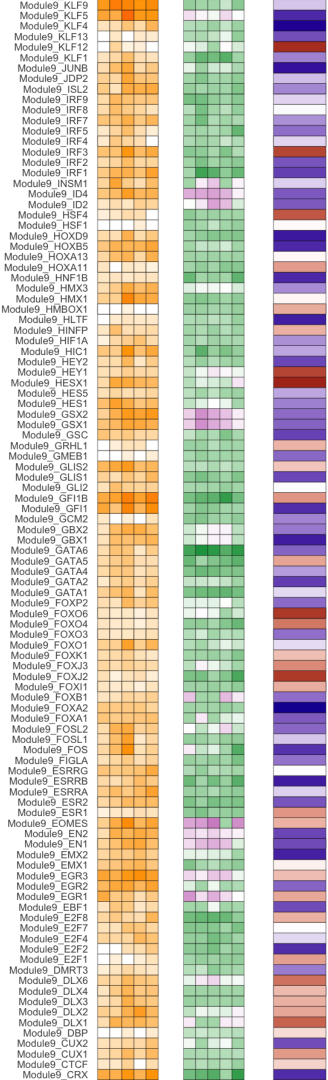
Module9_KLF9
Module9_KLF5
Module9_KLF4
Module9_KLF13
Module9_KLF12
Module9_KLF1
Module9_JUNB
Module9_JDP2
Module9_ISL2
Module9_IRF9
Module9_IRF8
Module9_IRF7
Module9_IRF5
Module9_IRF4
Module9_IRF3
Module9_IRF2
Module9_IRF1
Module9_INSM1
Module9_ID4
Module9_ID2
Module9_HSF4
Module9_HSF1
Module9_HOXD9
Module9_HOXB5
Module9_HOXA13
Module9_HOXA11
Module9_HNF1B
Module9_HMX3
Module9_HMX1
Module9_HMBOX1
Module9_HLTF
Module9_HINFP
Module9_HIF1A
Module9_HIC1
Module9_HEY2
Module9_HEY1
Module9_HESX1
Module9_HES5
Module9_HES1
Module9_GSX2
Module9_GSX1
Module9_GSC
Module9_GRHL1
Module9_GMEB1
Module9_GLIS2
Module9_GLIS1
Module9_GLI2
Module9_GFI1B
Module9_GFI1
Module9_GCM2
Module9_GBX2
Module9_GBX1
Module9_GATA6
Module9_GATA5
Module9_GATA4
Module9_GATA2
Module9_GATA1
Module9_FOXP2
Module9_FOXO6
Module9_FOXO4
Module9_FOXO3
Module9_FOXO1
Module9_FOXK1
Module9_FOXJ3
Module9_FOXJ2
Module9_FOXI1
Module9_FOXB1
Module9_FOXA2
Module9_FOXA1
Module9_FOSL2
Module9_FOSL1
Module9_FOS
Module9_FIGLA
Module9_ESRRG
Module9_ESRRB
Module9_ESRRA
Module9_ESR2
Module9_ESR1
Module9_EOMES
Module9_EN2
Module9_EN1
Module9_EMX2
Module9_EMX1
Module9_EGR3
Module9_EGR2
Module9_EGR1
Module9_EBF1
Module9_E2F8
Module9_E2F7
Module9_E2F4
Module9_E2F2
Module9_E2F1
Module9_DMRT3
Module9_DLX6
Module9_DLX4
Module9_DLX3
Module9_DLX2
Module9_DLX1
Module9_DBP
Module9_CUX2
Module9_CUX1
Module9_CTCF
Module9_CRX

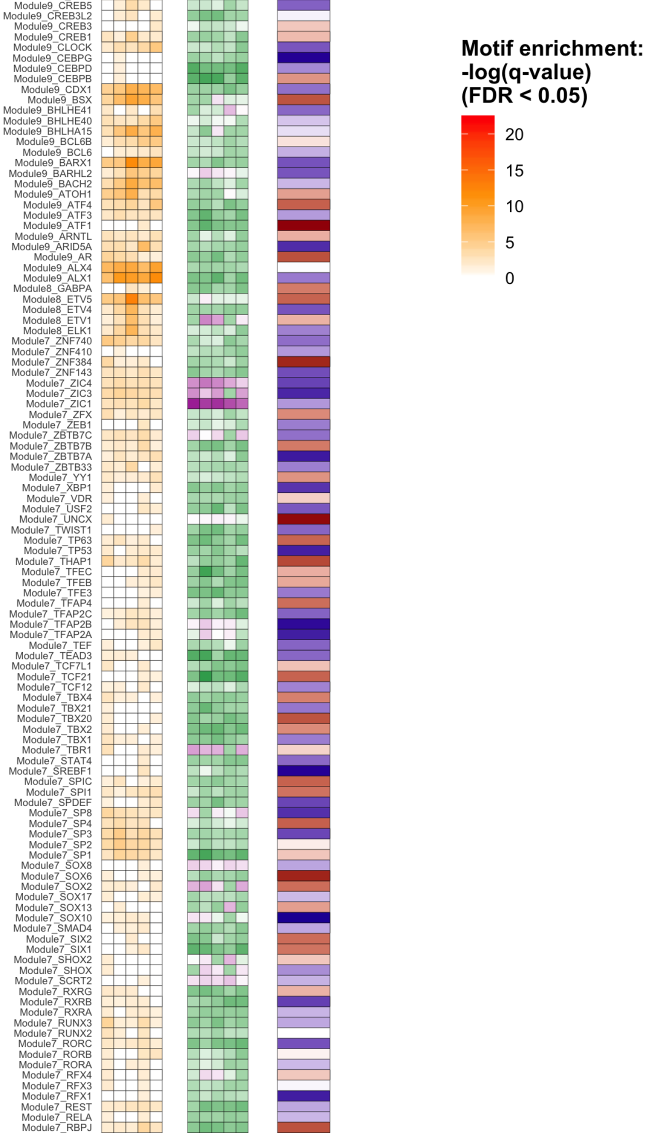
Module9_CREB5
Module9_CREB3L2
Module9_CREB3
Module9_CREB1
Module9_CLOCK
Module9_CEBPG
Module9_CEBPD
Module9_CEBPB
Module9_CDX1
Module9_BSX
Module9_BHLHE41
Module9_BHLHE40
Module9_BHLHA15
Module9_BCL6B
Module9_BCL6
Module9_BARX1
Module9_BARHL2
Module9_BACH2
Module9_ATOH1
Module9_ATF4
Module9_ATF3
Module9_ATF1
Module9_ARNTL
Module9_ARID5A
Module9_AR
Module9_ALX4
Module9_ALX1
Module8_GABPA
Module8_ETV5
Module8_ETV4
Module8_ETV1
Module8_ELK1
Module7_ZNF740
Module7_ZNF410
Module7_ZNF384
Module7_ZNF143
Module7_ZIC4
Module7_ZIC3
Module7_ZIC1
Module7_ZFX
Module7_ZEB1
Module7_ZBTB7C
Module7_ZBTB7B
Module7_ZBTB7A
Module7_ZBTB33
Module7_YY1
Module7_XBP1
Module7_VDR
Module7_USF2
Module7_UNCX
Module7_TWIST1
Module7_TP63
Module7_TP53
Module7_THAP1
Module7_TFEC
Module7_TFEB
Module7_TFE3
Module7_TFAP4
Module7_TFAP2C
Module7_TFAP2B
Module7_TFAP2A
Module7_TEF
Module7_TEAD3
Module7_TCF7L1
Module7_TCF21
Module7_TCF12
Module7_TBX4
Module7_TBX21
Module7_TBX20
Module7_TBX2
Module7_TBX1
Module7_TBR1
Module7_STAT4
Module7_SREBF1
Module7_SPIC
Module7_SPI1
Module7_SPDEF
Module7_SP8
Module7_SP4
Module7_SP3
Module7_SP2
Module7_SP1
Module7_SOX8
Module7_SOX6
Module7_SOX2
Module7_SOX17
Module7_SOX13
Module7_SOX10
Module7_SMAD4
Module7_SIX2
Module7_SIX1
Module7_SHOX2
Module7_SHOX
Module7_SCRT2
Module7_RXRG
Module7_RXRB
Module7_RXRA
Module7_RUNX3
Module7_RUNX2
Module7_RORC
Module7_RORB
Module7_RORA
Module7_RFX4
Module7_RFX3
Module7_RFX1
Module7_REST
Module7_RELA
Module7_RBPJ
Motif enrichment:
-log(q-value)
(FDR < 0.05)
20
15
10
5
0

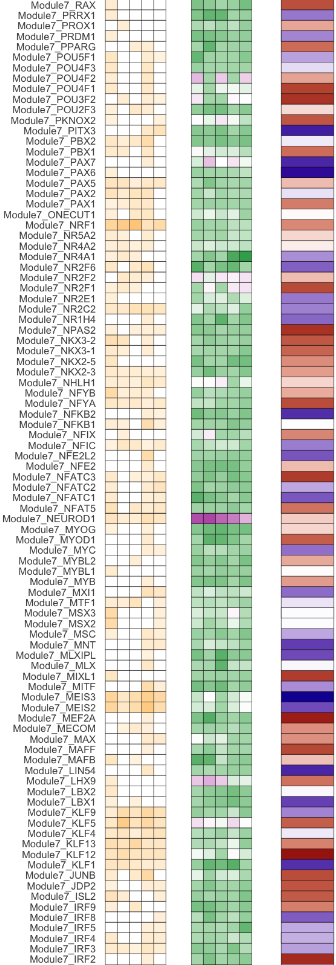
Module7_RAX
Module7_PRRX1
Module7_PROX1
Module7_PRDM1
Module7_PPARG
Module7_POU5F1
Module7_POU4F3
Module7_POU4F2
Module7_POU4F1
Module7_POU3F2
Module7_POU2F3
Module7_PKNOX2
Module7_PITX3
Module7_PBX2
Module7_PBX1
Module7_PAX7
Module7_PAX6
Module7_PAX5
Module7_PAX2
Module7_PAX1
Module7_ONECUT1
Module7_NRF1
Module7_NR5A2
Module7_NR4A2
Module7_NR4A1
Module7_NR2F6
Module7_NR2F2
Module7_NR2F1
Module7_NR2E1
Module7_NR2C2
Module7_NR1H4
Module7_NPAS2
Module7_NKX3-2
Module7_NKX3-1
Module7_NKX2-5
Module7_NKX2-3
Module7_NHLH1
Module7_NFYB
Module7_NFYA
Module7_NFKB2
Module7_NFKB1
Module7_NFIX
Module7_NFIC
Module7_NFE2L2
Module7_NFE2
Module7_NFATC3
Module7_NFATC2
Module7_NFATC1
Module7_NFAT5
Module7_NEUROD1
Module7_MYOG
Module7_MYOD1
Module7_MYC
Module7_MYBL2
Module7_MYBL1
Module7_MYB
Module7_MXI1
Module7_MTF1
Module7_MSX3
Module7_MSX2
Module7_MSC
Module7_MNT
Module7_MLXIPL
Module7_MLX
Module7_MIXL1
Module7_MITF
Module7_MEIS3
Module7_MEIS2
Module7_MEF2A
Module7_MECOM
Module7_MAX
Module7_MAFF
Module7_MAFB
Module7_LIN54
Module7_LHX9
Module7_LBX2
Module7_LBX1
Module7_KLF9
Module7_KLF5
Module7_KLF4
Module7_KLF13
Module7_KLF12
Module7_KLF1
Module7_JUNB
Module7_JDP2
Module7_ISL2
Module7_IRF9
Module7_IRF8
Module7_IRF5
Module7_IRF4
Module7_IRF3
Module7_IRF2

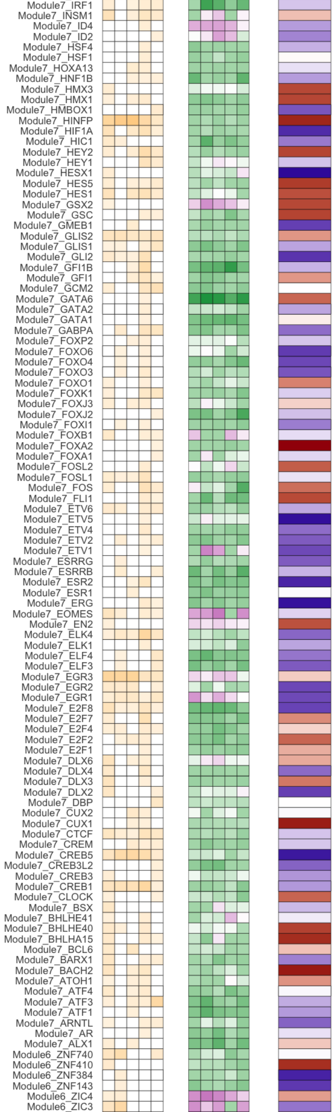
Module7_IRF1
Module7_INSM1
Module7_ID4
Module7_ID2
Module7_HSF4
Module7_HSF1
Module7_HOXA13
Module7_HNF1B
Module7_HMX3
Module7_HMX1
Module7_HMBOX1
Module7_HINFP
Module7_HIF1A
Module7_HIC1
Module7_HEY2
Module7_HEY1
Module7_HESX1
Module7_HES5
Module7_HES1
Module7_GSX2
Module7_GSC
Module7_GMEB1
Module7_GLIS2
Module7_GLIS1
Module7_GLI2
Module7_GFI1B
Module7_GFI1
Module7_GCM2
Module7_GATA6
Module7_GATA2
Module7_GATA1
Module7_GABPA
Module7_FOXP2
Module7_FOXO6
Module7_FOXO4
Module7_FOXO3
Module7_FOXO1
Module7_FOXK1
Module7_FOXJ3
Module7_FOXJ2
Module7_FOXI1
Module7_FOXB1
Module7_FOXA2
Module7_FOXA1
Module7_FOSL2
Module7_FOSL1
Module7_FOS
Module7_FLI1
Module7_ETV6
Module7_ETV5
Module7_ETV4
Module7_ETV2
Module7_ETV1
Module7_ESRRG
Module7_ESRRB
Module7_ESR2
Module7_ESR1
Module7_ERG
Module7_EOMES
Module7_EN2
Module7_ELK4
Module7_ELK1
Module7_ELF4
Module7_ELF3
Module7_EGR3
Module7_EGR2
Module7_EGR1
Module7_E2F8
Module7_E2F7
Module7_E2F4
Module7_E2F2
Module7_E2F1
Module7_DLX6
Module7_DLX4
Module7_DLX3
Module7_DLX2
Module7_DBP
Module7_CUX2
Module7_CUX1
Module7_CTCF
Module7_CREM
Module7_CREB5
Module7_CREB3L2
Module7_CREB3
Module7_CREB1
Module7_CLOCK
Module7_BSX
Module7_BHLHE41
Module7_BHLHE40
Module7_BHLHA15
Module7_BCL6
Module7_BARX1
Module7_BACH2
Module7_ATOH1
Module7_ATF4
Module7_ATF3
Module7_ATF1
Module7_ARNTL
Module7_AR
Module7_ALX1
Module6_ZNF740
Module6_ZNF410
Module6_ZNF384
Module6_ZNF143
Module6_ZIC4
Module6_ZIC3

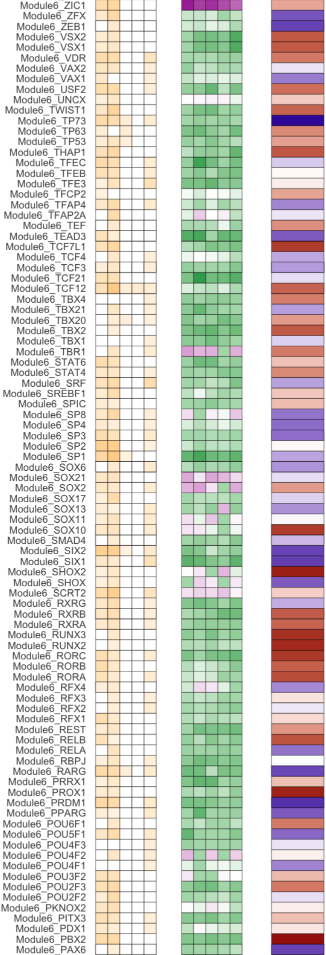
Module6_ZIC1
Module6_ZFX
Module6_ZEB1
Module6_VSX2
Module6_VSX1
Module6_VDR
Module6_VAX2
Module6_VAX1
Module6_USF2
Module6_UNCX
Module6_TWIST1
Module6_TP73
Module6_TP63
Module6_TP53
Module6_THAP1
Module6_TFEC
Module6_TFEB
Module6_TFE3
Module6_TFCP2
Module6_TFAP4
Module6_TFAP2A
Module6_TEF
Module6_TEAD3
Module6_TCF7L1
Module6_TCF4
Module6_TCF3
Module6_TCF21
Module6_TCF12
Module6_TBX4
Module6_TBX21
Module6_TBX20
Module6_TBX2
Module6_TBX1
Module6_TBR1
Module6_STAT6
Module6_STAT4
Module6_SRF
Module6_SREBF1
Module6_SPIC
Module6_SP8
Module6_SP4
Module6_SP3
Module6_SP2
Module6_SP1
Module6_SOX6
Module6_SOX21
Module6_SOX2
Module6_SOX17
Module6_SOX13
Module6_SOX11
Module6_SOX10
Module6_SMAD4
Module6_SIX2
Module6_SIX1
Module6_SHOX2
Module6_SHOX
Module6_SCRT2
Module6_RXRG
Module6_RXRB
Module6_RXRA
Module6_RUNX3
Module6_RUNX2
Module6_RORC
Module6_RORB
Module6_RORA
Module6_RFX4
Module6_RFX3
Module6_RFX2
Module6_RFX1
Module6_REST
Module6_RELB
Module6_RELA
Module6_RBPJ
Module6_RARG
Module6_PRRX1
Module6_PROX1
Module6_PRDM1
Module6_PPARG
Module6_POU6F1
Module6_POU5F1
Module6_POU4F3
Module6_POU4F2
Module6_POU4F1
Module6_POU3F2
Module6_POU2F3
Module6_POU2F2
Module6_PKNOX2
Module6_PITX3
Module6_PDX1
Module6_PBX2
Module6_PAX6

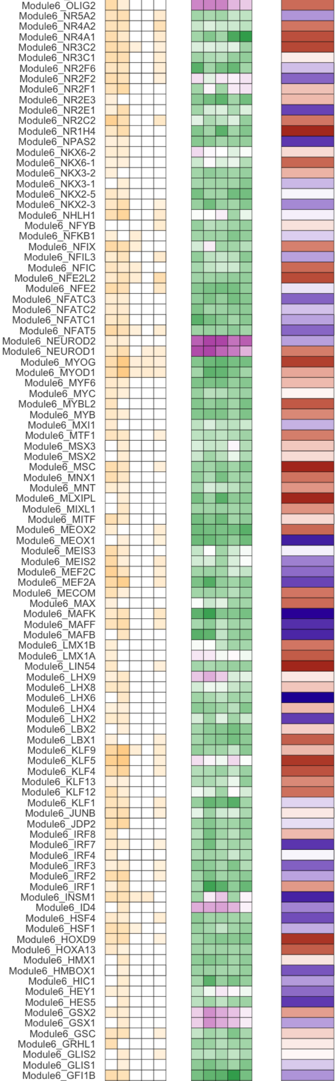
Module6_OLIG2
Module6_NR5A2
Module6_NR4A2
Module6_NR4A1
Module6_NR3C2
Module6_NR3C1
Module6_NR2F6
Module6_NR2F2
Module6_NR2F1
Module6_NR2E3
Module6_NR2E1
Module6_NR2C2
Module6_NR1H4
Module6_NPAS2
Module6_NKX6-2
Module6_NKX6-1
Module6_NKX3-2
Module6_NKX3-1
Module6_NKX2-5
Module6_NKX2-3
Module6_NHLH1
Module6_NFYB
Module6_NFKB1
Module6_NFIX
Module6_NFIL3
Module6_NFIC
Module6_NFE2L2
Module6_NFE2
Module6_NFATC3
Module6_NFATC2
Module6_NFATC1
Module6_NFAT5
Module6_NEUROD2
Module6_NEUROD1
Module6_MYOG
Module6_MYOD1
Module6_MYF6
Module6_MYC
Module6_MYBL2
Module6_MYB
Module6_MXI1
Module6_MTF1
Module6_MSX3
Module6_MSX2
Module6_MSC
Module6_MNX1
Module6_MNT
Module6_MLXIPL
Module6_MIXL1
Module6_MITF
Module6_MEOX2
Module6_MEOX1
Module6_MEIS3
Module6_MEIS2
Module6_MEF2C
Module6_MEF2A
Module6_MECOM
Module6_MAX
Module6_MAFK
Module6_MAFF
Module6_MAFB
Module6_LMX1B
Module6_LMX1A
Module6_LIN54
Module6_LHX9
Module6_LHX8
Module6_LHX6
Module6_LHX4
Module6_LHX2
Module6_LBX2
Module6_LBX1
Module6_KLF9
Module6_KLF5
Module6_KLF4
Module6_KLF13
Module6_KLF12
Module6_KLF1
Module6_JUNB
Module6_JDP2
Module6_IRF8
Module6_IRF7
Module6_IRF4
Module6_IRF3
Module6_IRF2
Module6_IRF1
Module6_INSM1
Module6_ID4
Module6_HSF4
Module6_HSF1
Module6_HOXD9
Module6_HOXA13
Module6_HMX1
Module6_HMBOX1
Module6_HIC1
Module6_HEY1
Module6_HES5
Module6_GSX2
Module6_GSX1
Module6_GSC
Module6_GRHL1
Module6_GLIS2
Module6_GLIS1
Module6_GFI1B

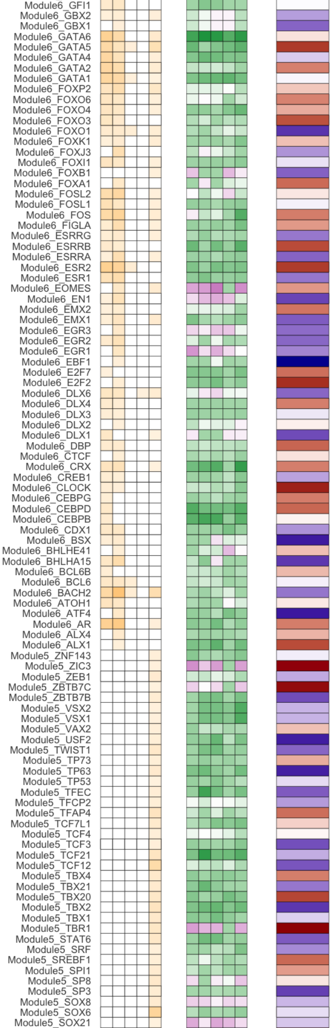
Module6_GFI1
Module6_GBX2
Module6_GBX1
Module6_GATA6
Module6_GATA5
Module6_GATA4
Module6_GATA2
Module6_GATA1
Module6_FOXP2
Module6_FOXO6
Module6_FOXO4
Module6_FOXO3
Module6_FOXO1
Module6_FOXK1
Module6_FOXJ3
Module6_FOXI1
Module6_FOXB1
Module6_FOXA1
Module6_FOSL2
Module6_FOSL1
Module6_FOS
Module6_FIGLA
Module6_ESRRG
Module6_ESRRB
Module6_ESRRA
Module6_ESR2
Module6_ESR1
Module6_EOMES
Module6_EN1
Module6_EMX2
Module6_EMX1
Module6_EGR3
Module6_EGR2
Module6_EGR1
Module6_EBF1
Module6_E2F7
Module6_E2F2
Module6_DLX6
Module6_DLX4
Module6_DLX3
Module6_DLX2
Module6_DLX1
Module6_DBP
Module6_CTCF
Module6_CRX
Module6_CREB1
Module6_CLOCK
Module6_CEBPG
Module6_CEBPD
Module6_CEBPB
Module6_CDX1
Module6_BSX
Module6_BHLHE41
Module6_BHLHA15
Module6_BCL6B
Module6_BCL6
Module6_BACH2
Module6_ATOH1
Module6_ATF4
Module6_AR
Module6_ALX4
Module6_ALX1
Module5_ZNF143
Module5_ZIC3
Module5_ZEB1
Module5_ZBTB7C
Module5_ZBTB7B
Module5_VSX2
Module5_VSX1
Module5_VAX2
Module5_USF2
Module5_TWIST1
Module5_TP73
Module5_TP63
Module5_TP53
Module5_TFEC
Module5_TFCP2
Module5_TFAP4
Module5_TCF7L1
Module5_TCF4
Module5_TCF3
Module5_TCF21
Module5_TCF12
Module5_TBX4
Module5_TBX21
Module5_TBX20
Module5_TBX2
Module5_TBX1
Module5_TBR1
Module5_STAT6
Module5_SRF
Module5_SREBF1
Module5_SPI1
Module5_SP8
Module5_SP3
Module5_SOX8
Module5_SOX6
Module5_SOX21

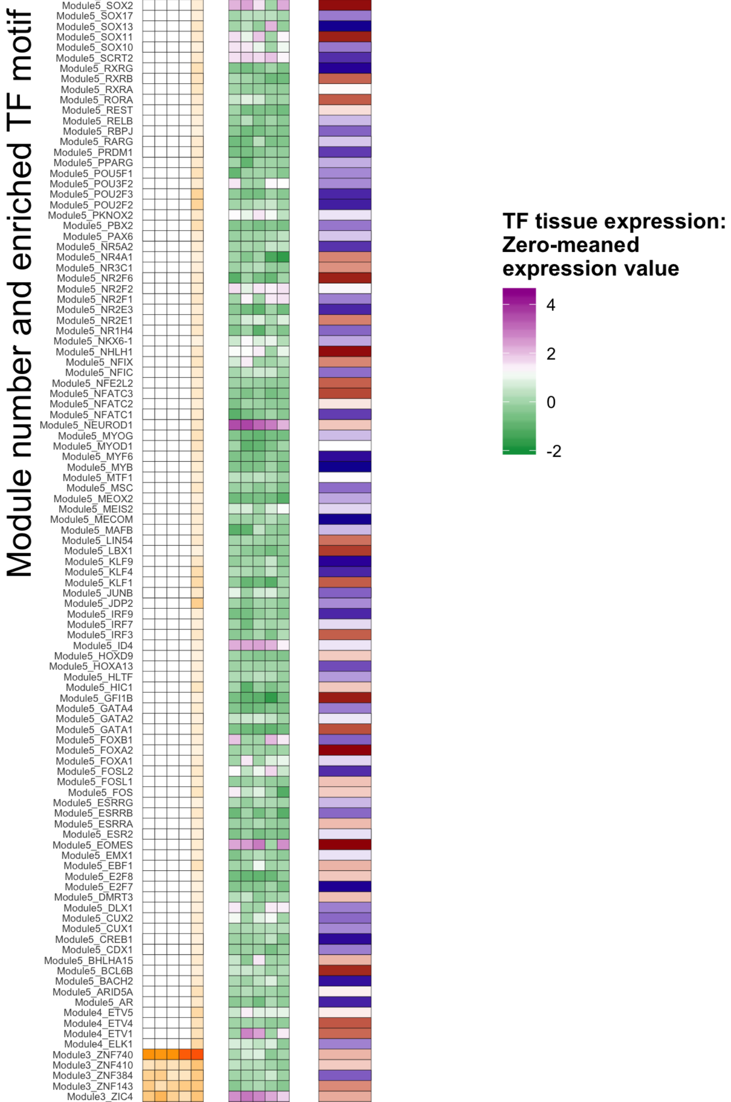
Module number and enriched TF motif
Module5_SOX2
Module5_SOX17
Module5_SOX13
Module5_SOX11
Module5_SOX10
Module5_SCRT2
Module5_RXRG
Module5_RXRB
Module5_RXRA
Module5_RORA
Module5_REST
Module5_RELB
Module5_RBPJ
Module5_RARG
Module5_PRDM1
Module5_PPARG
Module5_POU5F1
Module5_POU3F2
Module5_POU2F3
Module5_POU2F2
Module5_PKNOX2
Module5_PBX2
Module5_PAX6
Module5_NR5A2
Module5_NR4A1
Module5_NR3C1
Module5_NR2F6
Module5_NR2F2
Module5_NR2F1
Module5_NR2E3
Module5_NR2E1
Module5_NR1H4
Module5_NKX6-1
Module5_NHLH1
Module5_NFIX
Module5_NFIC
Module5_NFE2L2
Module5_NFATC3
Module5_NFATC2
Module5_NFATC1
Module5_NEUROD1
Module5_MYOG
Module5_MYOD1
Module5_MYF6
Module5_MYB
Module5_MTF1
Module5_MSC
Module5_MEOX2
Module5_MEIS2
Module5_MECOM
Module5_MAFB
Module5_LIN54
Module5_LBX1
Module5_KLF9
Module5_KLF4
Module5_KLF1
Module5_JUNB
Module5_JDP2
Module5_IRF9
Module5_IRF7
Module5_IRF3
Module5_ID4
Module5_HOXD9
Module5_HOXA13
Module5_HLTF
Module5_HIC1
Module5_GFI1B
Module5_GATA4
Module5_GATA2
Module5_GATA1
Module5_FOXB1
Module5_FOXA2
Module5_FOXA1
Module5_FOSL2
Module5_FOSL1
Module5_FOS
Module5_ESRRG
Module5_ESRRB
Module5_ESRRA
Module5_ESR2
Module5_EOMES
Module5_EMX1
Module5_EBF1
Module5_E2F8
Module5_E2F7
Module5_DMRT3
Module5_DLX1
Module5_CUX2
Module5_CUX1
Module5_CREB1
Module5_CDX1
Module5_BHLHA15
Module5_BCL6B
Module5_BACH2
Module5_ARID5A
Module5_AR
Module4_ETV5
Module4_ETV4
Module4_ETV1
Module4_ELK1
Module3_ZNF740
Module3_ZNF410
Module3_ZNF384
Module3_ZNF143
Module3_ZIC4
TF tissue expression:
Zero-meaned
expression value
4
2
0
-2

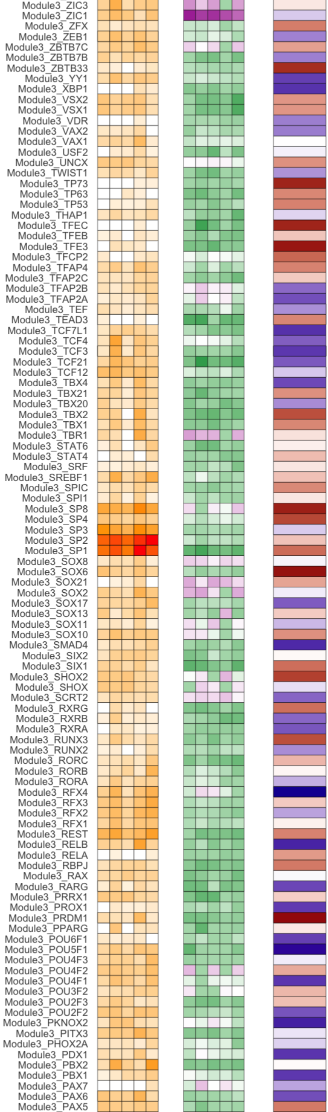
Module3_ZIC3
Module3_ZIC1
Module3_ZFX
Module3_ZEB1
Module3_ZBTB7C
Module3_ZBTB7B
Module3_ZBTB33
Module3_YY1
Module3_XBP1
Module3_VSX2
Module3_VSX1
Module3_VDR
Module3_VAX2
Module3_VAX1
Module3_USF2
Module3_UNCX
Module3_TWIST1
Module3_TP73
Module3_TP63
Module3_TP53
Module3_THAP1
Module3_TFEC
Module3_TFEB
Module3_TFE3
Module3_TFCP2
Module3_TFAP4
Module3_TFAP2C
Module3_TFAP2B
Module3_TFAP2A
Module3_TEF
Module3_TEAD3
Module3_TCF7L1
Module3_TCF4
Module3_TCF3
Module3_TCF21
Module3_TCF12
Module3_TBX4
Module3_TBX21
Module3_TBX20
Module3_TBX2
Module3_TBX1
Module3_TBR1
Module3_STAT6
Module3_STAT4
Module3_SRF
Module3_SREBF1
Module3_SPIC
Module3_SPI1
Module3_SP8
Module3_SP4
Module3_SP3
Module3_SP2
Module3_SP1
Module3_SOX8
Module3_SOX6
Module3_SOX21
Module3_SOX2
Module3_SOX17
Module3_SOX13
Module3_SOX11
Module3_SOX10
Module3_SMAD4
Module3_SIX2
Module3_SIX1
Module3_SHOX2
Module3_SHOX
Module3_SCRT2
Module3_RXRG
Module3_RXRB
Module3_RXRA
Module3_RUNX3
Module3_RUNX2
Module3_RORC
Module3_RORB
Module3_RORA
Module3_RFX4
Module3_RFX3
Module3_RFX2
Module3_RFX1
Module3_REST
Module3_RELB
Module3_RELA
Module3_RBPJ
Module3_RAX
Module3_RARG
Module3_PRRX1
Module3_PROX1
Module3_PRDM1
Module3_PPARG
Module3_POU6F1
Module3_POU5F1
Module3_POU4F3
Module3_POU4F2
Module3_POU4F1
Module3_POU3F2
Module3_POU2F3
Module3_POU2F2
Module3_PKNOX2
Module3_PITX3
Module3_PHOX2A
Module3_PDX1
Module3_PBX2
Module3_PBX1
Module3_PAX7
Module3_PAX6
Module3_PAX5

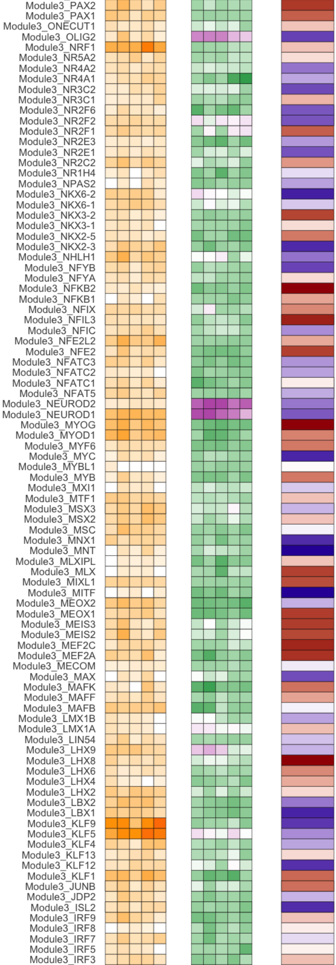
Module3_PAX2
Module3_PAX1
Module3_ONECUT1
Module3_OLIG2
Module3_NRF1
Module3_NR5A2
Module3_NR4A2
Module3_NR4A1
Module3_NR3C2
Module3_NR3C1
Module3_NR2F6
Module3_NR2F2
Module3_NR2F1
Module3_NR2E3
Module3_NR2E1
Module3_NR2C2
Module3_NR1H4
Module3_NPAS2
Module3_NKX6-2
Module3_NKX6-1
Module3_NKX3-2
Module3_NKX3-1
Module3_NKX2-5
Module3_NKX2-3
Module3_NHLH1
Module3_NFYB
Module3_NFYA
Module3_NFKB2
Module3_NFKB1
Module3_NFIX
Module3_NFIL3
Module3_NFIC
Module3_NFE2L2
Module3_NFE2
Module3_NFATC3
Module3_NFATC2
Module3_NFATC1
Module3_NFAT5
Module3_NEUROD2
Module3_NEUROD1
Module3_MYOG
Module3_MYOD1
Module3_MYF6
Module3_MYC
Module3_MYBL1
Module3_MYB
Module3_MXI1
Module3_MTF1
Module3_MSX3
Module3_MSX2
Module3_MSC
Module3_MNX1
Module3_MNT
Module3_MLXIPL
Module3_MLX
Module3_MIXL1
Module3_MITF
Module3_MEOX2
Module3_MEOX1
Module3_MEIS3
Module3_MEIS2
Module3_MEF2C
Module3_MEF2A
Module3_MECOM
Module3_MAX
Module3_MAFK
Module3_MAFF
Module3_MAFB
Module3_LMX1B
Module3_LMX1A
Module3_LIN54
Module3_LHX9
Module3_LHX8
Module3_LHX6
Module3_LHX4
Module3_LHX2
Module3_LBX2
Module3_LBX1
Module3_KLF9
Module3_KLF5
Module3_KLF4
Module3_KLF13
Module3_KLF12
Module3_KLF1
Module3_JUNB
Module3_JDP2
Module3_ISL2
Module3_IRF9
Module3_IRF8
Module3_IRF7
Module3_IRF5
Module3_IRF3

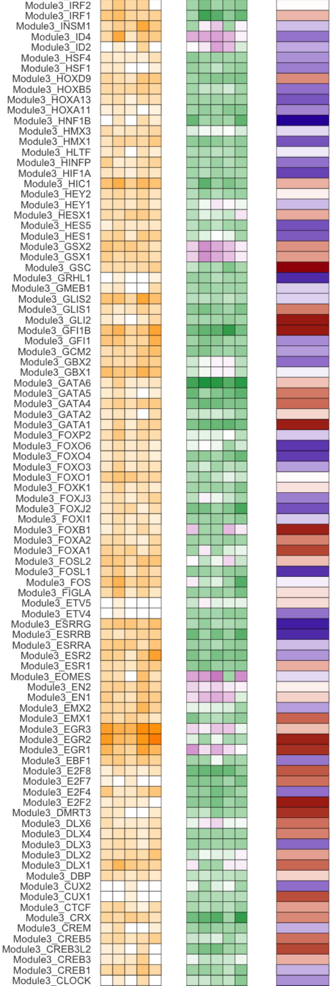
Module3_IRF2
Module3_IRF1
Module3_INSM1
Module3_ID4
Module3_ID2
Module3_HSF4
Module3_HSF1
Module3_HOXD9
Module3_HOXB5
Module3_HOXA13
Module3_HOXA11
Module3_HNF1B
Module3_HMX3
Module3_HMX1
Module3_HLTF
Module3_HINFP
Module3_HIF1A
Module3_HIC1
Module3_HEY2
Module3_HEY1
Module3_HESX1
Module3_HES5
Module3_HES1
Module3_GSX2
Module3_GSX1
Module3_GSC
Module3_GRHL1
Module3_GMEB1
Module3_GLIS2
Module3_GLIS1
Module3_GLI2
Module3_GFI1B
Module3_GFI1
Module3_GCM2
Module3_GBX2
Module3_GBX1
Module3_GATA6
Module3_GATA5
Module3_GATA4
Module3_GATA2
Module3_GATA1
Module3_FOXP2
Module3_FOXO6
Module3_FOXO4
Module3_FOXO3
Module3_FOXO1
Module3_FOXK1
Module3_FOXJ3
Module3_FOXJ2
Module3_FOXI1
Module3_FOXB1
Module3_FOXA2
Module3_FOXA1
Module3_FOSL2
Module3_FOSL1
Module3_FOS
Module3_FIGLA
Module3_ETV5
Module3_ETV4
Module3_ESRRG
Module3_ESRRB
Module3_ESRRA
Module3_ESR2
Module3_ESR1
Module3_EOMES
Module3_EN2
Module3_EN1
Module3_EMX2
Module3_EMX1
Module3_EGR3
Module3_EGR2
Module3_EGR1
Module3_EBF1
Module3_E2F8
Module3_E2F7
Module3_E2F4
Module3_E2F2
Module3_DMRT3
Module3_DLX6
Module3_DLX4
Module3_DLX3
Module3_DLX2
Module3_DLX1
Module3_DBP
Module3_CUX2
Module3_CUX1
Module3_CTCF
Module3_CRX
Module3_CREM
Module3_CREB5
Module3_CREB3L2
Module3_CREB3
Module3_CREB1
Module3_CLOCK

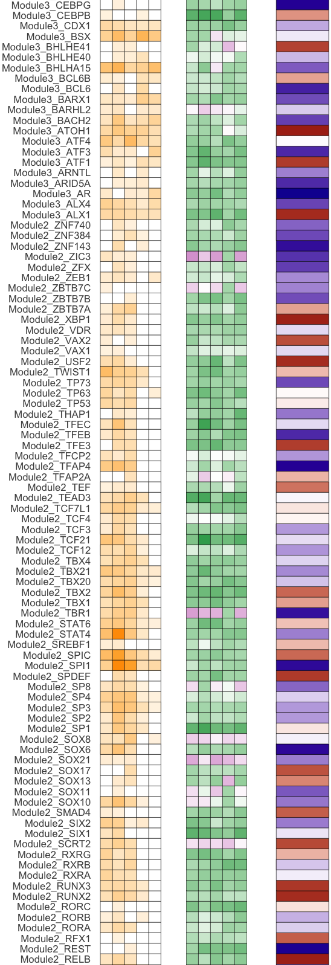
Module3_CEBPG
Module3_CEBPB
Module3_CDX1
Module3_BSX
Module3_BHLHE41
Module3_BHLHE40
Module3_BHLHA15
Module3_BCL6B
Module3_BCL6
Module3_BARX1
Module3_BARHL2
Module3_BACH2
Module3_ATOH1
Module3_ATF4
Module3_ATF3
Module3_ATF1
Module3_ARNTL
Module3_ARID5A
Module3_AR
Module3_ALX4
Module3_ALX1
Module2_ZNF740
Module2_ZNF384
Module2_ZNF143
Module2_ZIC3
Module2_ZFX
Module2_ZEB1
Module2_ZBTB7C
Module2_ZBTB7B
Module2_ZBTB7A
Module2_XBP1
Module2_VDR
Module2_VAX2
Module2_VAX1
Module2_USF2
Module2_TWIST1
Module2_TP73
Module2_TP63
Module2_TP53
Module2_THAP1
Module2_TFEC
Module2_TFEB
Module2_TFE3
Module2_TFCP2
Module2_TFAP4
Module2_TFAP2A
Module2_TEF
Module2_TEAD3
Module2_TCF7L1
Module2_TCF4
Module2_TCF3
Module2_TCF21
Module2_TCF12
Module2_TBX4
Module2_TBX21
Module2_TBX20
Module2_TBX2
Module2_TBX1
Module2_TBR1
Module2_STAT6
Module2_STAT4
Module2_SREBF1
Module2_SPIC
Module2_SPI1
Module2_SPDEF
Module2_SP8
Module2_SP4
Module2_SP3
Module2_SP2
Module2_SP1
Module2_SOX8
Module2_SOX6
Module2_SOX21
Module2_SOX17
Module2_SOX13
Module2_SOX11
Module2_SOX10
Module2_SMAD4
Module2_SIX2
Module2_SIX1
Module2_SCRT2
Module2_RXRG
Module2_RXRB
Module2_RXRA
Module2_RUNX3
Module2_RUNX2
Module2_RORC
Module2_RORB
Module2_RORA
Module2_RFX1
Module2_REST
Module2_RELB

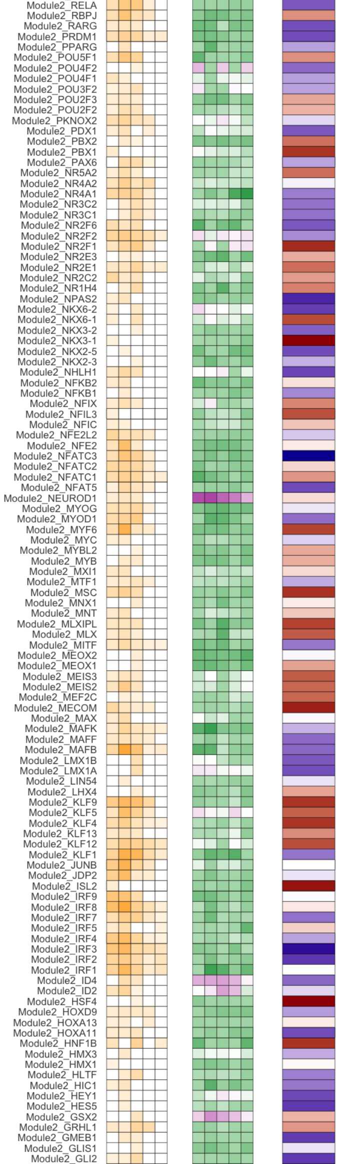
Module2_RELA
Module2_RBPJ
Module2_RARG
Module2_PRDM1
Module2_PPARG
Module2_POU5F1
Module2_POU4F2
Module2_POU4F1
Module2_POU3F2
Module2_POU2F3
Module2_POU2F2
Module2_PKNOX2
Module2_PDX1
Module2_PBX2
Module2_PBX1
Module2_PAX6
Module2_NR5A2
Module2_NR4A2
Module2_NR4A1
Module2_NR3C2
Module2_NR3C1
Module2_NR2F6
Module2_NR2F2
Module2_NR2F1
Module2_NR2E3
Module2_NR2E1
Module2_NR2C2
Module2_NR1H4
Module2_NPAS2
Module2_NKX6-2
Module2_NKX6-1
Module2_NKX3-2
Module2_NKX3-1
Module2_NKX2-5
Module2_NKX2-3
Module2_NHLH1
Module2_NFKB2
Module2_NFKB1
Module2_NFIX
Module2_NFIL3
Module2_NFIC
Module2_NFE2L2
Module2_NFE2
Module2_NFATC3
Module2_NFATC2
Module2_NFATC1
Module2_NFAT5
Module2_NEUROD1
Module2_MYOG
Module2_MYOD1
Module2_MYF6
Module2_MYC
Module2_MYBL2
Module2_MYB
Module2_MXI1
Module2_MTF1
Module2_MSC
Module2_MNX1
Module2_MNT
Module2_MLXIPL
Module2_MLX
Module2_MITF
Module2_MEOX2
Module2_MEOX1
Module2_MEIS3
Module2_MEIS2
Module2_MEF2C
Module2_MECOM
Module2_MAX
Module2_MAFK
Module2_MAFF
Module2_MAFB
Module2_LMX1B
Module2_LMX1A
Module2_LIN54
Module2_LHX4
Module2_KLF9
Module2_KLF5
Module2_KLF4
Module2_KLF13
Module2_KLF12
Module2_KLF1
Module2_JUNB
Module2_JDP2
Module2_ISL2
Module2_IRF9
Module2_IRF8
Module2_IRF7
Module2_IRF5
Module2_IRF4
Module2_IRF3
Module2_IRF2
Module2_IRF1
Module2_ID4
Module2_ID2
Module2_HSF4
Module2_HOXD9
Module2_HOXA13
Module2_HOXA11
Module2_HNF1B
Module2_HMX3
Module2_HMX1
Module2_HLTF
Module2_HIC1
Module2_HEY1
Module2_HES5
Module2_GSX2
Module2_GRHL1
Module2_GMEB1
Module2_GLIS1
Module2_GLI2

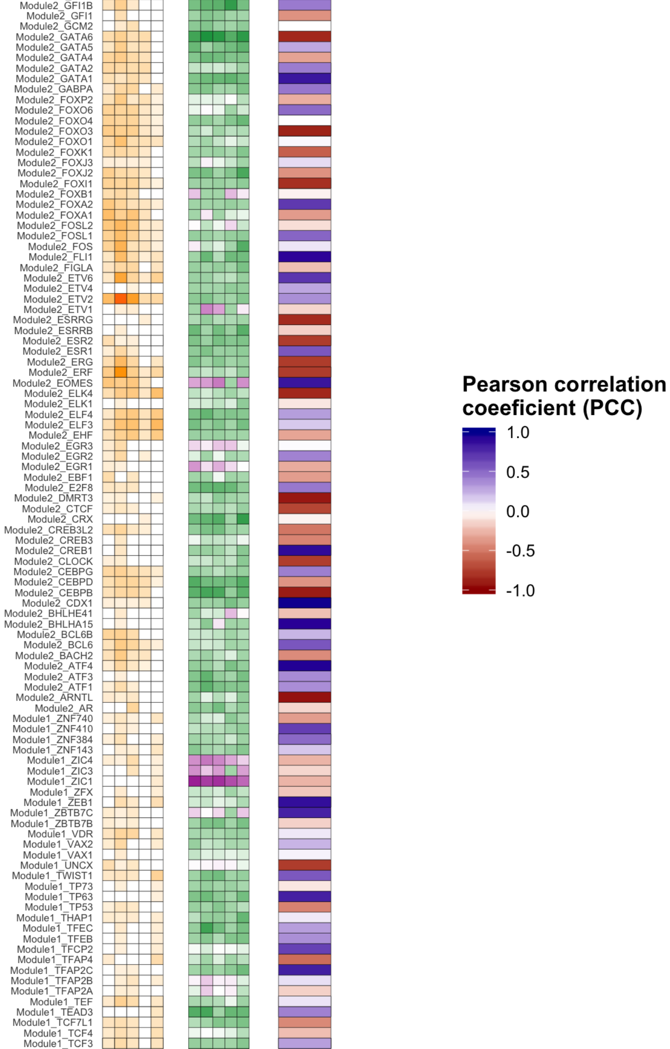
Module2_GFI1B
Module2_GFI1
Module2_GCM2
Module2_GATA6
Module2_GATA5
Module2_GATA4
Module2_GATA2
Module2_GATA1
Module2_GABPA
Module2_FOXP2
Module2_FOXO6
Module2_FOXO4
Module2_FOXO3
Module2_FOXO1
Module2_FOXK1
Module2_FOXJ3
Module2_FOXJ2
Module2_FOXI1
Module2_FOXB1
Module2_FOXA2
Module2_FOXA1
Module2_FOSL2
Module2_FOSL1
Module2_FOS
Module2_FLI1
Module2_FIGLA
Module2_ETV6
Module2_ETV4
Module2_ETV2
Module2_ETV1
Module2_ESRRG
Module2_ESRRB
Module2_ESR2
Module2_ESR1
Module2_ERG
Module2_ERF
Module2_EOMES
Module2_ELK4
Module2_ELK1
Module2_ELF4
Module2_ELF3
Module2_EHF
Module2_EGR3
Module2_EGR2
Module2_EGR1
Module2_EBF1
Module2_E2F8
Module2_DMRT3
Module2_CTCF
Module2_CRX
Module2_CREB3L2
Module2_CREB3
Module2_CREB1
Module2_CLOCK
Module2_CEBPG
Module2_CEBPD
Module2_CEBPB
Module2_CDX1
Module2_BHLHE41
Module2_BHLHA15
Module2_BCL6B
Module2_BCL6
Module2_BACH2
Module2_ATF4
Module2_ATF3
Module2_ATF1
Module2_ARNTL
Module2_AR
Module1_ZNF740
Module1_ZNF410
Module1_ZNF384
Module1_ZNF143
Module1_ZIC4
Module1_ZIC3
Module1_ZIC1
Module1_ZFX
Module1_ZEB1
Module1_ZBTB7C
Module1_ZBTB7B
Module1_VDR
Module1_VAX2
Module1_VAX1
Module1_UNCX
Module1_TWIST1
Module1_TP73
Module1_TP63
Module1_TP53
Module1_THAP1
Module1_TFEC
Module1_TFEB
Module1_TFCP2
Module1_TFAP4
Module1_TFAP2C
Module1_TFAP2B
Module1_TFAP2A
Module1_TEF
Module1_TEAD3
Module1_TCF7L1
Module1_TCF4
Module1_TCF3
Pearson correlation coeeficient (PCC)
1.0
0.5
0.0
-0.5
-1.0

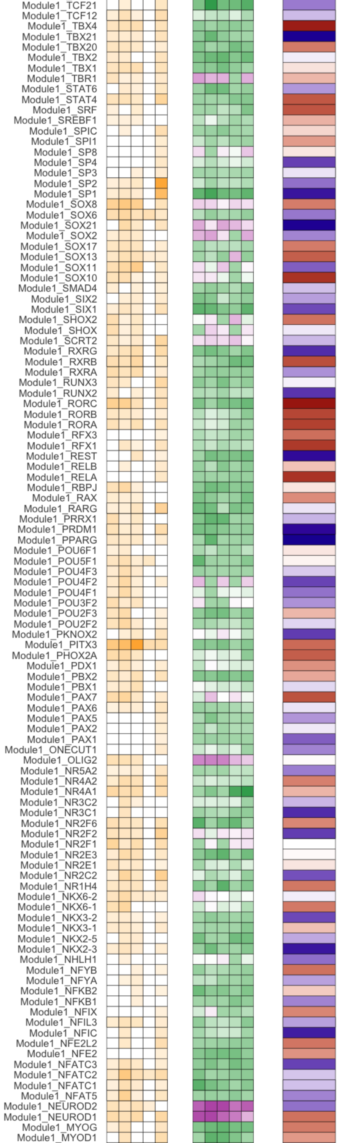
Module1_TCF21
Module1_TCF12
Module1_TBX4
Module1_TBX21
Module1_TBX20
Module1_TBX2
Module1_TBX1
Module1_TBR1
Module1_STAT6
Module1_STAT4
Module1_SRF
Module1_SREBF1
Module1_SPIC
Module1_SPI1
Module1_SP8
Module1_SP4
Module1_SP3
Module1_SP2
Module1_SP1
Module1_SOX8
Module1_SOX6
Module1_SOX21
Module1_SOX2
Module1_SOX17
Module1_SOX13
Module1_SOX11
Module1_SOX10
Module1_SMAD4
Module1_SIX2
Module1_SIX1
Module1_SHOX2
Module1_SHOX
Module1_SCRT2
Module1_RXRG
Module1_RXRB
Module1_RXRA
Module1_RUNX3
Module1_RUNX2
Module1_RORC
Module1_RORB
Module1_RORA
Module1_RFX3
Module1_RFX1
Module1_REST
Module1_RELB
Module1_RELA
Module1_RBPJ
Module1_RAX
Module1_RARG
Module1_PRRX1
Module1_PRDM1
Module1_PPARG
Module1_POU6F1
Module1_POU5F1
Module1_POU4F3
Module1_POU4F2
Module1_POU4F1
Module1_POU3F2
Module1_POU2F3
Module1_POU2F2
Module1_PKNOX2
Module1_PITX3
Module1_PHOX2A
Module1_PDX1
Module1_PBX2
Module1_PBX1
Module1_PAX7
Module1_PAX6
Module1_PAX5
Module1_PAX2
Module1_PAX1
Module1_ONECUT1
Module1_OLIG2
Module1_NR5A2
Module1_NR4A2
Module1_NR4A1
Module1_NR3C2
Module1_NR3C1
Module1_NR2F6
Module1_NR2F2
Module1_NR2F1
Module1_NR2E3
Module1_NR2E1
Module1_NR2C2
Module1_NR1H4
Module1_NKX6-2
Module1_NKX6-1
Module1_NKX3-2
Module1_NKX3-1
Module1_NKX2-5
Module1_NKX2-3
Module1_NHLH1
Module1_NFYB
Module1_NFYA
Module1_NFKB2
Module1_NFKB1
Module1_NFIX
Module1_NFIL3
Module1_NFIC
Module1_NFE2L2
Module1_NFE2
Module1_NFATC3
Module1_NFATC2
Module1_NFATC1
Module1_NFAT5
Module1_NEUROD2
Module1_NEUROD1
Module1_MYOG
Module1_MYOD1

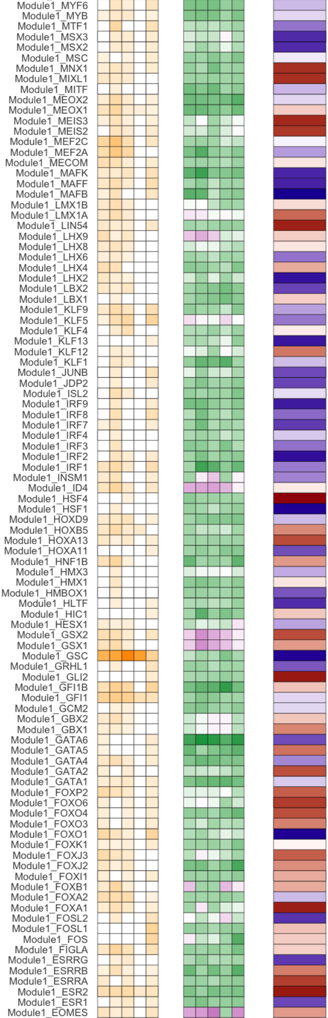
Module1_MYF6
Module1_MYB
Module1_MTF1
Module1_MSX3
Module1_MSX2
Module1_MSC
Module1_MNX1
Module1_MIXL1
Module1_MITF
Module1_MEOX2
Module1_MEOX1
Module1_MEIS3
Module1_MEIS2
Module1_MEF2C
Module1_MEF2A
Module1_MECOM
Module1_MAFK
Module1_MAFF
Module1_MAFB
Module1_LMX1B
Module1_LMX1A
Module1_LIN54
Module1_LHX9
Module1_LHX8
Module1_LHX6
Module1_LHX4
Module1_LHX2
Module1_LBX2
Module1_LBX1
Module1_KLF9
Module1_KLF5
Module1_KLF4
Module1_KLF13
Module1_KLF12
Module1_KLF1
Module1_JUNB
Module1_JDP2
Module1_ISL2
Module1_IRF9
Module1_IRF8
Module1_IRF7
Module1_IRF4
Module1_IRF3
Module1_IRF2
Module1_IRF1
Module1_INSM1
Module1_ID4
Module1_HSF4
Module1_HSF1
Module1_HOXD9
Module1_HOXB5
Module1_HOXA13
Module1_HOXA11
Module1_HNF1B
Module1_HMX3
Module1_HMX1
Module1_HMBOX1
Module1_HLTF
Module1_HIC1
Module1_HESX1
Module1_GSX2
Module1_GSX1
Module1_GSC
Module1_GRHL1
Module1_GLI2
Module1_GFI1B
Module1_GFI1
Module1_GCM2
Module1_GBX2
Module1_GBX1
Module1_GATA6
Module1_GATA5
Module1_GATA4
Module1_GATA2
Module1_GATA1
Module1_FOXP2
Module1_FOXO6
Module1_FOXO4
Module1_FOXO3
Module1_FOXO1
Module1_FOXK1
Module1_FOXJ3
Module1_FOXJ2
Module1_FOXI1
Module1_FOXB1
Module1_FOXA2
Module1_FOXA1
Module1_FOSL2
Module1_FOSL1
Module1_FOS
Module1_FIGLA
Module1_ESRRG
Module1_ESRRB
Module1_ESRRA
Module1_ESR2
Module1_ESR1
Module1_EOMES

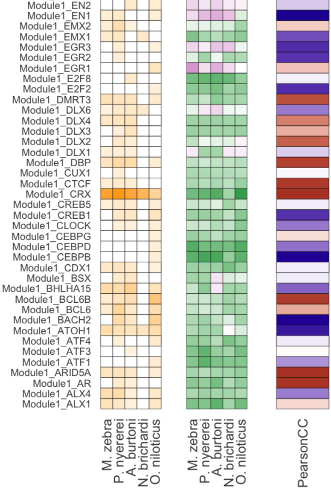
Module1_EN2
Module1_EN1
Module1_EMX2
Module1_EMX1
Module1_EGR3
Module1_EGR2
Module1_EGR1
Module1_E2F8
Module1_E2F2
Module1_DMRT3
Module1_DLX6
Module1_DLX4
Module1_DLX3
Module1_DLX2
Module1_DLX1
Module1_DBP
Module1_CUX1
Module1_CTCF
Module1_CRX
Module1_CREB5
Module1_CREB1
Module1_CLOCK
Module1_CEBPG
Module1_CEBPD
Module1_CEBPB
Module1_CDX1
Module1_BSX
Module1_BHLHA15
Module1_BCL6B
Module1_BCL6
Module1_BACH2
Module1_ATOH1
Module1_ATF4
Module1_ATF3
Module1_ATF1
Module1_ARID5A
Module1_AR
Module1_ALX4
Module1_ALX1
M. zebra
P. nyererei
A. burtoni
N. brichardi
O. niloticus
M. zebra
P. nyererei
A. burtoni
N. brichardi
O. niloticus
PearsonCC

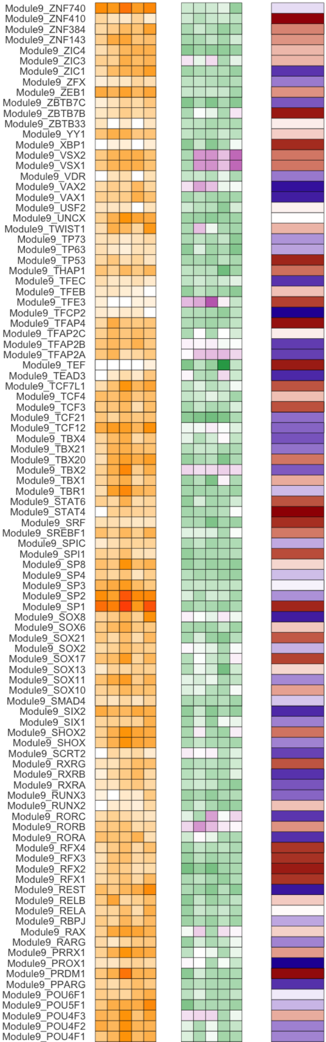

Additional file 2: Fig. S4
Heatmap matrices of enrichment, expression and Pearson correlation between the two for each motif, across eye tissue

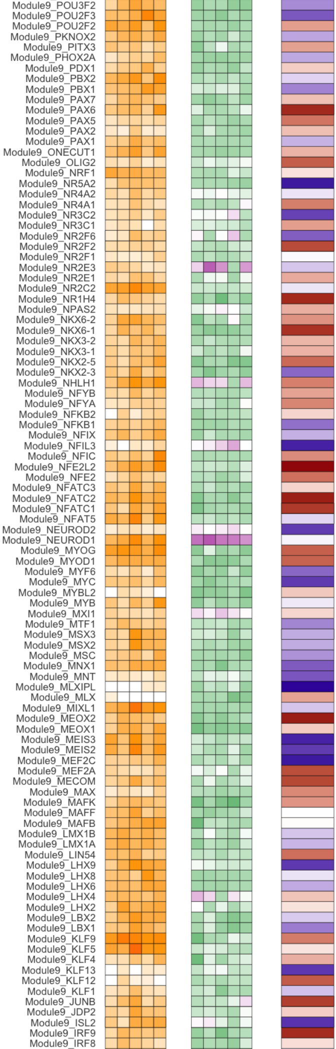
Module9_POU3F2
Module9_POU2F3
Module9_POU2F2
Module9_PKNOX2
Module9_PITX3
Module9_PHOX2A
Module9_PDX1
Module9_PBX2
Module9_PBX1
Module9_PAX7
Module9_PAX6
Module9_PAX5
Module9_PAX2
Module9_PAX1
Module9_ONECUT1
Module9_OLIG2
Module9_NRF1
Module9_NR5A2
Module9_NR4A2
Module9_NR4A1
Module9_NR3C2
Module9_NR3C1
Module9_NR2F6
Module9_NR2F2
Module9_NR2F1
Module9_NR2E3
Module9_NR2E1
Module9_NR2C2
Module9_NR1H4
Module9_NPAS2
Module9_NKX6-2
Module9_NKX6-1
Module9_NKX3-2
Module9_NKX3-1
Module9_NKX2-5
Module9_NKX2-3
Module9_NHLH1
Module9_NFYB
Module9_NFYA
Module9_NFKB2
Module9_NFKB1
Module9_NFIX
Module9_NFIL3
Module9_NFIC
Module9_NFE2L2
Module9_NFE2
Module9_NFATC3
Module9_NFATC2
Module9_NFATC1
Module9_NFAT5
Module9_NEUROD2
Module9_NEUROD1
Module9_MYOG
Module9_MYOD1
Module9_MYF6
Module9_MYC
Module9_MYBL2
Module9_MYB
Module9_MXI1
Module9_MTF1
Module9_MSX3
Module9_MSX2
Module9_MSC
Module9_MNX1
Module9_MNT
Module9_MLXIPL
Module9_MLX
Module9_MIXL1
Module9_MEOX2
Module9_MEOX1
Module9_MEIS3
Module9_MEIS2
Module9_MEF2C
Module9_MEF2A
Module9_MECOM
Module9_MAX
Module9_MAFK
Module9_MAFF
Module9_MAFB
Module9_LMX1B
Module9_LMX1A
Module9_LIN54
Module9_LHX9
Module9_LHX8
Module9_LHX6
Module9_LHX4
Module9_LHX2
Module9_LBX2
Module9_LBX1
Module9_KLF9
Module9_KLF5
Module9_KLF4
Module9_KLF13
Module9_KLF12
Module9_KLF1
Module9_JUNB
Module9_JDP2
Module9_ISL2
Module9_IRF9
Module9_IRF8

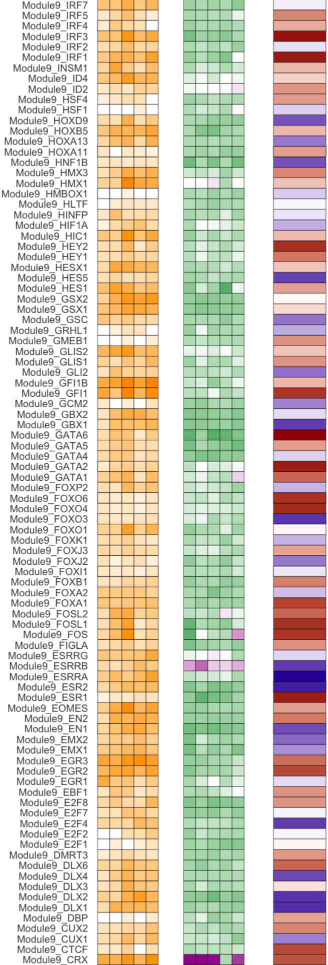
Module9_IRF7
Module9_IRF5
Module9_IRF4
Module9_IRF3
Module9_IRF2
Module9_IRF1
Module9_INSM1
Module9_ID4
Module9_ID2
Module9_HSF4
Module9_HSF1
Module9_HOXD9
Module9_HOXB5
Module9_HOXA13
Module9_HOXA11
Module9_HNF1B
Module9_HMX3
Module9_HMX1
Module9_HMBOX1
Module9_HLTF
Module9_HINFP
Module9_HIF1A
Module9_HIC1
Module9_HEY2
Module9_HEY1
Module9_HESX1
Module9_HES5
Module9_HES1
Module9_GSX2
Module9_GSX1
Module9_GSC
Module9_GRHL1
Module9_GMEB1
Module9_GLIS2
Module9_GLIS1
Module9_GLI2
Module9_GFI1B
Module9_GFI1
Module9_GCM2
Module9_GBX2
Module9_GBX1
Module9_GATA6
Module9_GATA5
Module9_GATA4
Module9_GATA2
Module9_GATA1
Module9_FOXP2
Module9_FOXO6
Module9_FOXO4
Module9_FOXO3
Module9_FOXO1
Module9_FOXK1
Module9_FOXJ3
Module9_FOXJ2
Module9_FOXI1
Module9_FOXB1
Module9_FOXA2
Module9_FOXA1
Module9_FOSL2
Module9_FOSL1
Module9_FOS
Module9_FIGLA
Module9_ESRRG
Module9_ESRRB
Module9_ESRRA
Module9_ESR2
Module9_ESR1
Module9_EOMES
Module9_EN2
Module9_EN1
Module9_EMX2
Module9_EMX1
Module9_EGR3
Module9_EGR2
Module9_EGR1
Module9_EBF1
Module9_E2F8
Module9_E2F7
Module9_E2F4
Module9_E2F2
Module9_E2F1
Module9_DMRT3
Module9_DLX6
Module9_DLX4
Module9_DLX3
Module9_DLX2
Module9_DLX1
Module9_DBP
Module9_CUX2
Module9_CUX1
Module9_CTCF
Module9_CRX

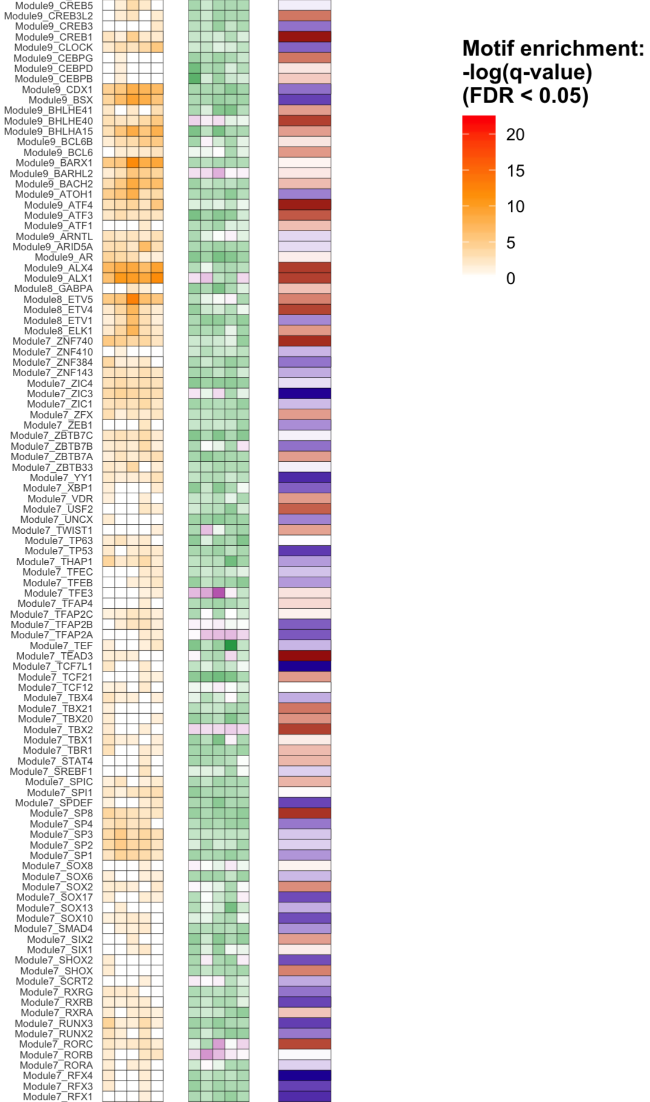
Module9_CREB5
Module9_CREB3L2
Module9_CREB3
Module9_CREB1
Module9_CLOCK
Module9_CEBPG
Module9_CEBPD
Module9_CEBPB
Module9_CDX1
Module9_BSX
Module9_BHLHE41
Module9_BHLHE40
Module9_BHLHA15
Module9_BCL6B
Module9_BCL6
Module9_BARX1
Module9_BARHL2
Module9_BACH2
Module9_ATOH1
Module9_ATF4
Module9_ATF3
Module9_ATF1
Module9_ARNTL
Module9_ARID5A
Module9_AR
Module9_ALX4
Module9_ALX1
Module8_GABPA
Module8_ETV5
Module8_ETV4
Module8_ETV1
Module8_ELK1
Module7_ZNF740
Module7_ZNF410
Module7_ZNF384
Module7_ZNF143
Module7_ZIC4
Module7_ZIC3
Module7_ZIC1
Module7_ZFX
Module7_ZEB1
Module7_ZBTB7C
Module7_ZBTB7B
Module7_ZBTB7A
Module7_ZBTB33
Module7_YY1
Module7_XBP1
Module7_VDR
Module7_USF2
Module7_UNCX
Module7_TWIST1
Module7_TP63
Module7_TP53
Module7_THAP1
Module7_TFEC
Module7_TFEB
Module7_TFE3
Module7_TFAP4
Module7_TFAP2C
Module7_TFAP2B
Module7_TFAP2A
Module7_TEF
Module7_TEAD3
Module7_TCF7L1
Module7_TCF21
Module7_TCF12
Module7_TBX4
Module7_TBX21
Module7_TBX20
Module7_TBX2
Module7_TBX1
Module7_TBR1
Module7_STAT4
Module7_SREBF1
Module7_SPIC
Module7_SPI1
Module7_SPDEF
Module7_SP8
Module7_SP4
Module7_SP3
Module7_SP2
Module7_SP1
Module7_SOX8
Module7_SOX6
Module7_SOX2
Module7_SOX17
Module7_SOX13
Module7_SOX10
Module7_SMAD4
Module7_SIX2
Module7_SIX1
Module7_SHOX2
Module7_SHOX
Module7_SCRT2
Module7_RXRG
Module7_RXRB
Module7_RXRA
Module7_RUNX3
Module7_RUNX2
Module7_RORC
Module7_RORB
Module7_RORA
Module7_RFX4
Module7_RFX3
Module7_RFX1
Motif enrichment:
-log(q-value)
(FDR < 0.05)
20
15
10
5
0

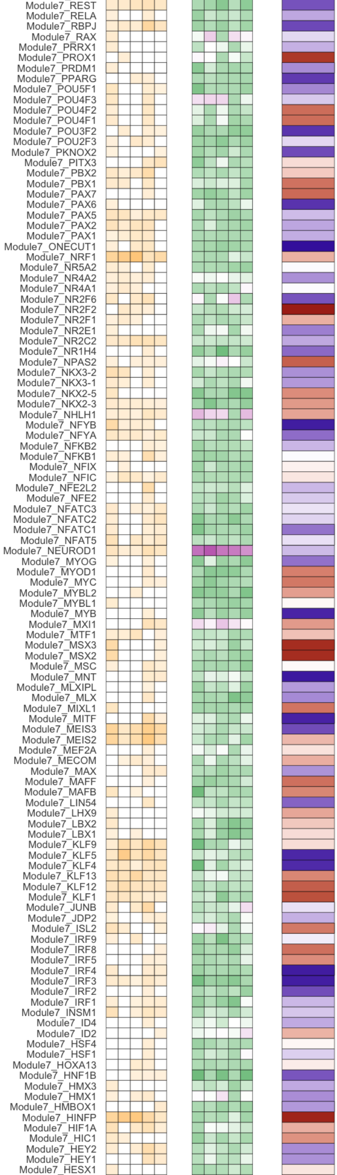
Module7_REST
Module7_RELA
Module7_RBPJ
Module7_RAX
Module7_PRRX1
Module7_PROX1
Module7_PRDM1
Module7_PPARG
Module7_POU5F1
Module7_POU4F3
Module7_POU4F2
Module7_POU4F1
Module7_POU3F2
Module7_POU2F3
Module7_PKNOX2
Module7_PITX3
Module7_PBX2
Module7_PBX1
Module7_PAX7
Module7_PAX6
Module7_PAX5
Module7_PAX2
Module7_PAX1
Module7_ONECUT1
Module7_NRF1
Module7_NR5A2
Module7_NR4A2
Module7_NR4A1
Module7_NR2F6
Module7_NR2F2
Module7_NR2F1
Module7_NR2E1
Module7_NR2C2
Module7_NR1H4
Module7_NPAS2
Module7_NKX3-2
Module7_NKX3-1
Module7_NKX2-5
Module7_NKX2-3
Module7_NHLH1
Module7_NFYB
Module7_NFYA
Module7_NFKB2
Module7_NFKB1
Module7_NFIX
Module7_NFIC
Module7_NFE2L2
Module7_NFE2
Module7_NFATC3
Module7_NFATC2
Module7_NFATC1
Module7_NFAT5
Module7_NEUROD1
Module7_MYOG
Module7_MYOD1
Module7_MYC
Module7_MYBL2
Module7_MYBL1
Module7_MYB
Module7_MXI1
Module7_MTF1
Module7_MSX3
Module7_MSX2
Module7_MSC
Module7_MNT
Module7_MLXIPL
Module7_MLX
Module7_MIXL1
Module7_MITF
Module7_MEIS3
Module7_MEIS2
Module7_MEF2A
Module7_MECOM
Module7_MAX
Module7_MAFF
Module7_MAFB
Module7_LIN54
Module7_LHX9
Module7_LBX2
Module7_LBX1
Module7_KLF9
Module7_KLF5
Module7_KLF4
Module7_KLF13
Module7_KLF12
Module7_KLF1
Module7_JUNB
Module7_JDP2
Module7_ISL2
Module7_IRF9
Module7_IRF8
Module7_IRF5
Module7_IRF4
Module7_IRF3
Module7_IRF2
Module7_IRF1
Module7_INSM1
Module7_ID4
Module7_ID2
Module7_HSF4
Module7_HSF1
Module7_HOXA13
Module7_HNF1B
Module7_HMX3
Module7_HMX1
Module7_HMBOX1
Module7_HINFP
Module7_HIF1A
Module7_HIC1
Module7_HEY2
Module7_HEY1
Module7_HESX1

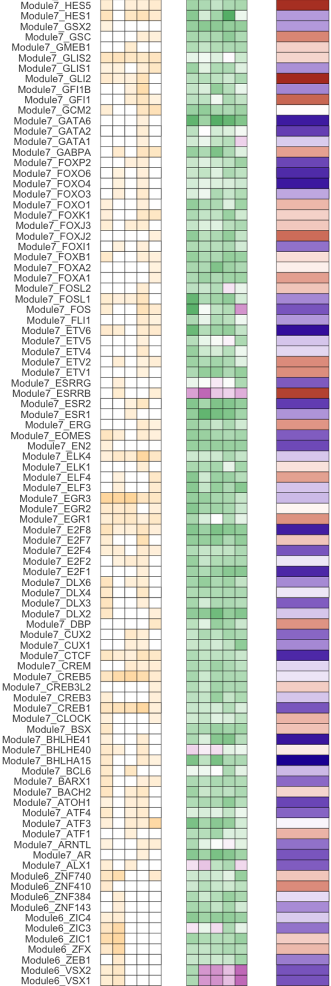
Module7_HES5
Module7_HES1
Module7_GSX2
Module7_GSC
Module7_GMEB1
Module7_GLIS2
Module7_GLIS1
Module7_GLI2
Module7_GFI1B
Module7_GFI1
Module7_GCM2
Module7_GATA6
Module7_GATA2
Module7_GATA1
Module7_GABPA
Module7_FOXP2
Module7_FOXO6
Module7_FOXO4
Module7_FOXO3
Module7_FOXO1
Module7_FOXK1
Module7_FOXJ3
Module7_FOXJ2
Module7_FOXI1
Module7_FOXB1
Module7_FOXA2
Module7_FOXA1
Module7_FOSL2
Module7_FOSL1
Module7_FOS
Module7_FLI1
Module7_ETV6
Module7_ETV5
Module7_ETV4
Module7_ETV2
Module7_ETV1
Module7_ESRRG
Module7_ESRRB
Module7_ESR2
Module7_ESR1
Module7_ERG
Module7_EOMES
Module7_EN2
Module7_ELK4
Module7_ELK1
Module7_ELF4
Module7_ELF3
Module7_EGR3
Module7_EGR2
Module7_EGR1
Module7_E2F8
Module7_E2F7
Module7_E2F4
Module7_E2F2
Module7_E2F1
Module7_DLX6
Module7_DLX4
Module7_DLX3
Module7_DLX2
Module7_DBP
Module7_CUX2
Module7_CUX1
Module7_CTCF
Module7_CREM
Module7_CREB5
Module7_CREB3L2
Module7_CREB3
Module7_CREB1
Module7_CLOCK
Module7_BSX
Module7_BHLHE41
Module7_BHLHE40
Module7_BHLHA15
Module7_BCL6
Module7_BARX1
Module7_BACH2
Module7_ATOH1
Module7_ATF4
Module7_ATF3
Module7_ATF1
Module7_ARNTL
Module7_AR
Module7_ALX1
Module6_ZNF740
Module6_ZNF410
Module6_ZNF384
Module6_ZNF143
Module6_ZIC4
Module6_ZIC3
Module6_ZIC1
Module6_ZFX
Module6_ZEB1
Module6_VSX2
Module6_VSX1

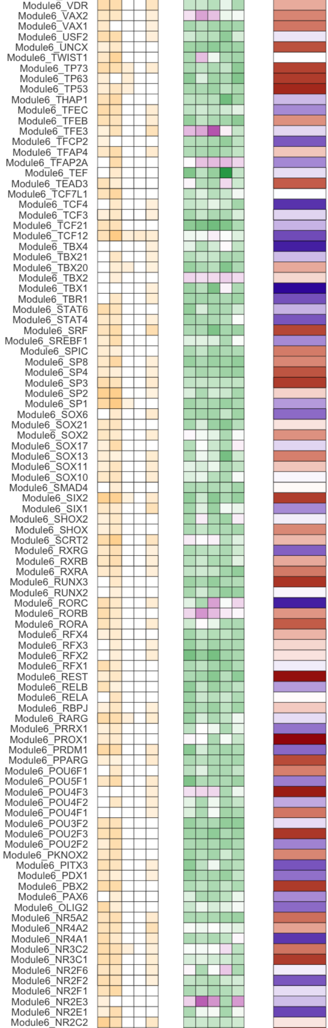
Module6_VDR
Module6_VAX2
Module6_VAX1
Module6_USF2
Module6_UNCX
Module6_TWIST1
Module6_TP73
Module6_TP63
Module6_TP53
Module6_THAP1
Module6_TFEC
Module6_TFEB
Module6_TFE3
Module6_TFCP2
Module6_TFAP4
Module6_TFAP2A
Module6_TEF
Module6_TEAD3
Module6_TCF7L1
Module6_TCF4
Module6_TCF3
Module6_TCF21
Module6_TCF12
Module6_TBX4
Module6_TBX21
Module6_TBX20
Module6_TBX2
Module6_TBX1
Module6_TBR1
Module6_STAT6
Module6_STAT4
Module6_SRF
Module6_SREBF1
Module6_SPIC
Module6_SP8
Module6_SP4
Module6_SP3
Module6_SP2
Module6_SP1
Module6_SOX6
Module6_SOX21
Module6_SOX2
Module6_SOX17
Module6_SOX13
Module6_SOX11
Module6_SOX10
Module6_SMAD4
Module6_SIX2
Module6_SIX1
Module6_SHOX2
Module6_SHOX
Module6_SCRT2
Module6_RXRG
Module6_RXRB
Module6_RXRA
Module6_RUNX3
Module6_RUNX2
Module6_RORC
Module6_RORB
Module6_RORA
Module6_RFX4
Module6_RFX3
Module6_RFX2
Module6_RFX1
Module6_REST
Module6_RELB
Module6_RELA
Module6_RBPJ
Module6_RARG
Module6_PRRX1
Module6_PROX1
Module6_PRDM1
Module6_PPARG
Module6_POU6F1
Module6_POU5F1
Module6_POU4F3
Module6_POU4F2
Module6_POU4F1
Module6_POU3F2
Module6_POU2F3
Module6_POU2F2
Module6_PKNOX2
Module6_PITX3
Module6_PDX1
Module6_PBX2
Module6_PAX6
Module6_OLIG2
Module6_NR5A2
Module6_NR4A2
Module6_NR4A1
Module6_NR3C2
Module6_NR3C1
Module6_NR2F6
Module6_NR2F2
Module6_NR2F1
Module6_NR2E3
Module6_NR2E1
Module6_NR2C2

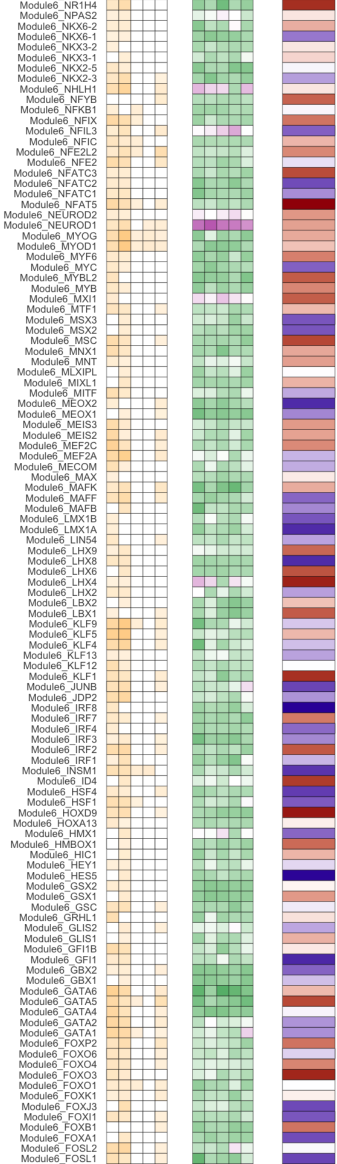
Module6_NR1H4
Module6_NPAS2
Module6_NKX6-2
Module6_NKX6-1
Module6_NKX3-2
Module6_NKX3-1
Module6_NKX2-5
Module6_NKX2-3
Module6_NHLH1
Module6_NFYB
Module6_NFKB1
Module6_NFIX
Module6_NFIL3
Module6_NFIC
Module6_NFE2L2
Module6_NFE2
Module6_NFATC3
Module6_NFATC2
Module6_NFATC1
Module6_NFAT5
Module6_NEUROD2
Module6_NEUROD1
Module6_MYOG
Module6_MYOD1
Module6_MYF6
Module6_MYC
Module6_MYBL2
Module6_MYB
Module6_MXI1
Module6_MTF1
Module6_MSX3
Module6_MSX2
Module6_MSC
Module6_MNX1
Module6_MNT
Module6_MLXIPL
Module6_MIXL1
Module6_MITF
Module6_MEOX2
Module6_MEOX1
Module6_MEIS3
Module6_MEIS2
Module6_MEF2C
Module6_MEF2A
Module6_MECOM
Module6_MAX
Module6_MAFK
Module6_MAFF
Module6_MAFB
Module6_LMX1B
Module6_LMX1A
Module6_LIN54
Module6_LHX9
Module6_LHX8
Module6_LHX6
Module6_LHX4
Module6_LHX2
Module6_LBX2
Module6_LBX1
Module6_KLF9
Module6_KLF5
Module6_KLF4
Module6_KLF13
Module6_KLF12
Module6_KLF1
Module6_JUNB
Module6_JDP2
Module6_IRF8
Module6_IRF7
Module6_IRF4
Module6_IRF3
Module6_IRF2
Module6_IRF1
Module6_INSM1
Module6_ID4
Module6_HSF4
Module6_HSF1
Module6_HOXD9
Module6_HOXA13
Module6_HMX1
Module6_HMBOX1
Module6_HIC1
Module6_HEY1
Module6_HES5
Module6_GSX2
Module6_GSX1
Module6_GSC
Module6_GRHL1
Module6_GLIS2
Module6_GLIS1
Module6_GFI1B
Module6_GFI1
Module6_GBX2
Module6_GBX1
Module6_GATA6
Module6_GATA5
Module6_GATA4
Module6_GATA2
Module6_GATA1
Module6_FOXP2
Module6_FOXO6
Module6_FOXO4
Module6_FOXO3
Module6_FOXO1
Module6_FOXK1
Module6_FOXJ3
Module6_FOXI1
Module6_FOXB1
Module6_FOXA1
Module6_FOSL2
Module6_FOSL1

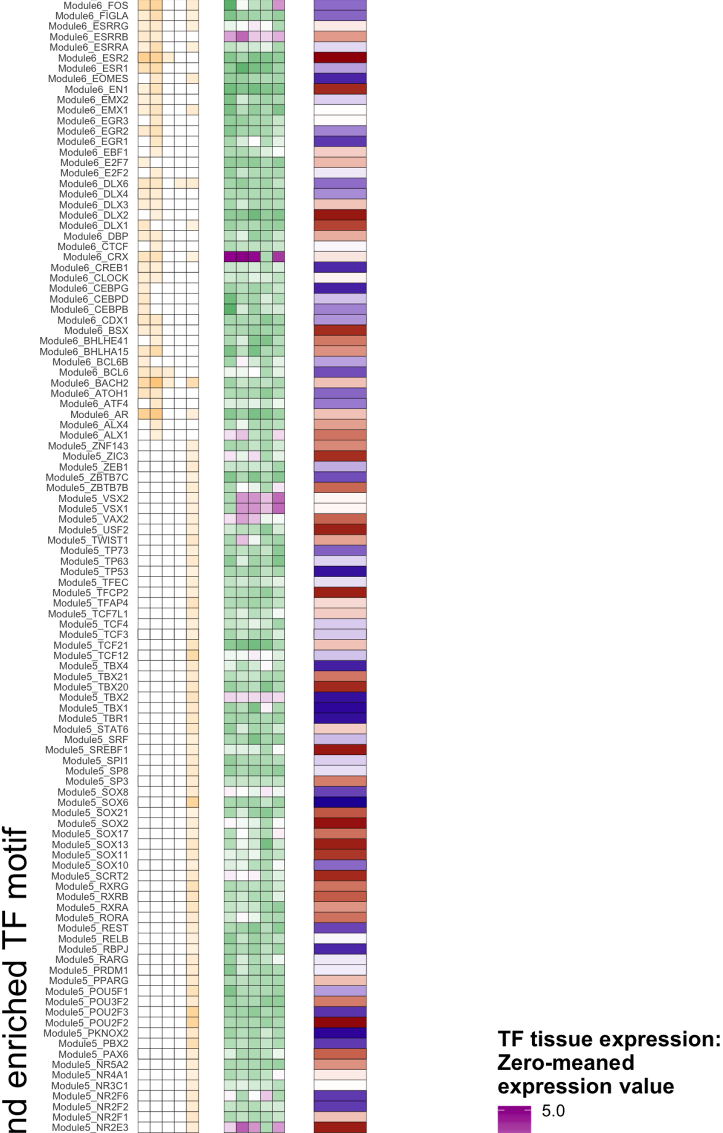

Module6_FOS
Module6_FIGLA
Module6_ESRRG
Module6_ESRRB
Module6_ESRRA
Module6_ESR2
Module6_ESR1
Module6_EOMES
Module6_EN1
Module6_EMX2
Module6_EMX1
Module6_EGR3
Module6_EGR2
Module6_EGR1
Module6_EBF1
Module6_E2F7
Module6_E2F2
Module6_DLX6
Module6_DLX4
Module6_DLX3
Module6_DLX2
Module6_DLX1
Module6_DBP
Module6_CTCF
Module6_CRX
Module6_CREB1
Module6_CLOCK
Module6_CEBPG
Module6_CEBPD
Module6_CEBPB
Module6_CDX1
Module6_BSX
Module6_BHLHE41
Module6_BHLHA15
Module6_BCL6B
Module6_BCL6
Module6_BACH2
Module6_ATOH1
Module6_ATF4
Module6_AR
Module6_ALX4
Module6_ALX1
Module5_ZNF143
Module5_ZIC3
Module5_ZEB1
Module5_ZBTB7C
Module5_ZBTB7B
Module5_VSX2
Module5_VSX1
Module5_VAX2
Module5_USF2
Module5_TWIST1
Module5_TP73
Module5_TP63
Module5_TP53
Module5_TFEC
Module5_TFCP2
Module5_TFAP4
Module5_TCF7L1
Module5_TCF4
Module5_TCF3
Module5_TCF21
Module5_TCF12
Module5_TBX4
Module5_TBX21
Module5_TBX20
Module5_TBX2
Module5_TBX1
Module5_TBR1
Module5_STAT6
Module5_SRF
Module5_SREBF1
Module5_SPI1
Module5_SP8
Module5_SP3
Module5_SOX8
Module5_SOX6
Module5_SOX21
Module5_SOX2
Module5_SOX17
Module5_SOX13
Module5_SOX11
Module5_SOX10
Module5_SCRT2
Module5_RXRG
Module5_RXRB
Module5_RXRA
Module5_RORA
Module5_REST
Module5_RELB
Module5_RBPJ
Module5_RARG
Module5_PRDM1
Module5_PPARG
Module5_POU5F1
Module5_POU3F2
Module5_POU2F3
Module5_POU2F2
Module5_PKNOX2
Module5_PBX2
Module5_PAX6
Module5_NR5A2
Module5_NR4A1
Module5_NR3C1
Module5_NR2F6
Module5_NR2F2
Module5_NR2F1
Module5_NR2E3
nd enriched TF motif
TF tissue expression:
Zero-meaned
expression value
5.0

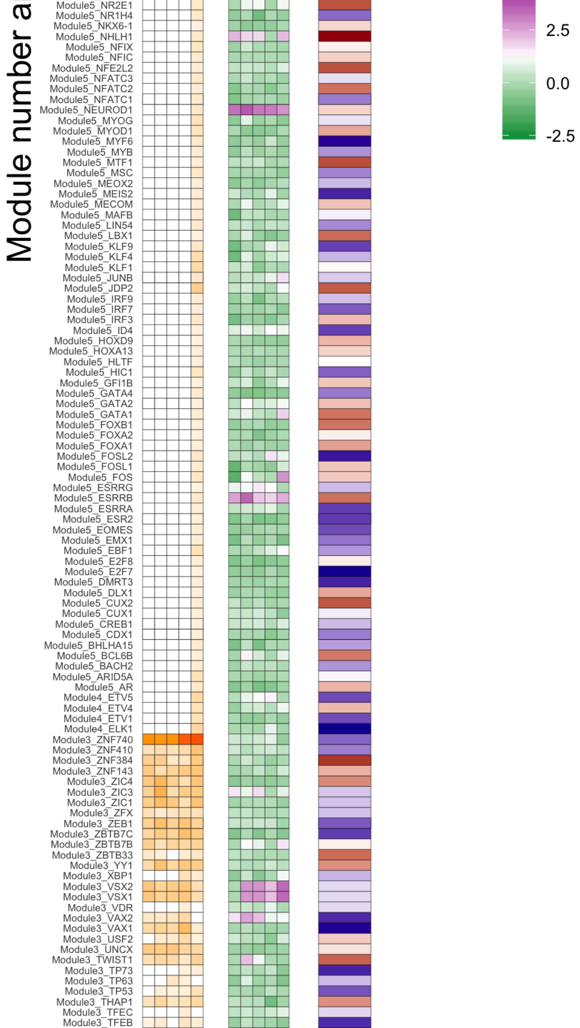
Module number a
Module5_NR2E1
Module5_NR1H4
Module5_NKX6-1
Module5_NHLH1
Module5_NFIX
Module5_NFIC
Module5_NFE2L2
Module5_NFATC3
Module5_NFATC2
Module5_NFATC1
Module5_NEUROD1
Module5_MYOG
Module5_MYOD1
Module5_MYF6
Module5_MYB
Module5_MTF1
Module5_MSC
Module5_MEOX2
Module5_MEIS2
Module5_MECOM
Module5_MAFB
Module5_LIN54
Module5_LBX1
Module5_KLF9
Module5_KLF4
Module5_KLF1
Module5_JUNB
Module5_JDP2
Module5_IRF9
Module5_IRF7
Module5_IRF3
Module5_ID4
Module5_HOXD9
Module5_HOXA13
Module5_HLTF
Module5_HIC1
Module5_GFI1B
Module5_GATA4
Module5_GATA2
Module5_GATA1
Module5_FOXB1
Module5_FOXA2
Module5_FOXA1
Module5_FOSL2
Module5_FOSL1
Module5_FOS
Module5_ESRRG
Module5_ESRRB
Module5_ESRRA
Module5_ESR2
Module5_EOMES
Module5_EMX1
Module5_EBF1
Module5_E2F8
Module5_E2F7
Module5_DMRT3
Module5_DLX1
Module5_CUX2
Module5_CUX1
Module5_CREB1
Module5_CDX1
Module5_BHLHA15
Module5_BCL6B
Module5_BACH2
Module5_ARID5A
Module5_AR
Module4_ETV5
Module4_ETV4
Module4_ETV1
Module4_ELK1
Module3_ZNF740
Module3_ZNF410
Module3_ZNF384
Module3_ZNF143
Module3_ZIC4
Module3_ZIC3
Module3_ZIC1
Module3_ZFX
Module3_ZEB1
Module3_ZBTB7C
Module3_ZBTB7B
Module3_ZBTB33
Module3_YY1
Module3_XBP1
Module3_VSX2
Module3_VSX1
Module3_VDR
Module3_VAX2
Module3_VAX1
Module3_USF2
Module3_UNCX
Module3_TWIST1
Module3_TP73
Module3_TP63
Module3_TP53
Module3_THAP1
Module3_TFEC
Module3_TFEB
2.5
0.0
-2.5

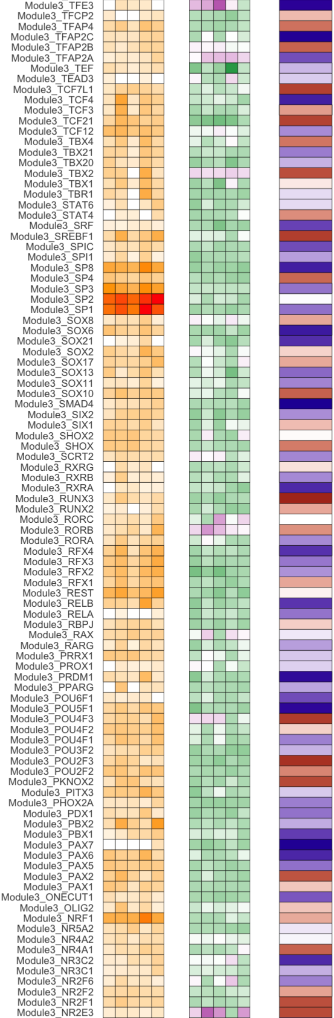
Module3_TFE3
Module3_TFCP2
Module3_TFAP4
Module3_TFAP2C
Module3_TFAP2B
Module3_TFAP2A
Module3_TEF
Module3_TEAD3
Module3_TCF7L1
Module3_TCF4
Module3_TCF3
Module3_TCF21
Module3_TCF12
Module3_TBX4
Module3_TBX21
Module3_TBX20
Module3_TBX2
Module3_TBX1
Module3_TBR1
Module3_STAT6
Module3_STAT4
Module3_SRF
Module3_SREBF1
Module3_SPIC
Module3_SPI1
Module3_SP8
Module3_SP4
Module3_SP3
Module3_SP2
Module3_SP1
Module3_SOX8
Module3_SOX6
Module3_SOX21
Module3_SOX2
Module3_SOX17
Module3_SOX13
Module3_SOX11
Module3_SOX10
Module3_SMAD4
Module3_SIX2
Module3_SIX1
Module3_SHOX2
Module3_SHOX
Module3_SCRT2
Module3_RXRG
Module3_RXRB
Module3_RXRA
Module3_RUNX3
Module3_RUNX2
Module3_RORC
Module3_RORB
Module3_RORA
Module3_RFX4
Module3_RFX3
Module3_RFX2
Module3_RFX1
Module3_REST
Module3_RELB
Module3_RELA
Module3_RBPJ
Module3_RAX
Module3_RARG
Module3_PRRX1
Module3_PROX1
Module3_PRDM1
Module3_PPARG
Module3_POU6F1
Module3_POU5F1
Module3_POU4F3
Module3_POU4F2
Module3_POU4F1
Module3_POU3F2
Module3_POU2F3
Module3_POU2F2
Module3_PKNOX2
Module3_PITX3
Module3_PHOX2A
Module3_PDX1
Module3_PBX2
Module3_PBX1
Module3_PAX7
Module3_PAX6
Module3_PAX5
Module3_PAX2
Module3_PAX1
Module3_ONECUT1
Module3_OLIG2
Module3_NRF1
Module3_NR5A2
Module3_NR4A2
Module3_NR4A1
Module3_NR3C2
Module3_NR3C1
Module3_NR2F6
Module3_NR2F2
Module3_NR2F1
Module3_NR2E3

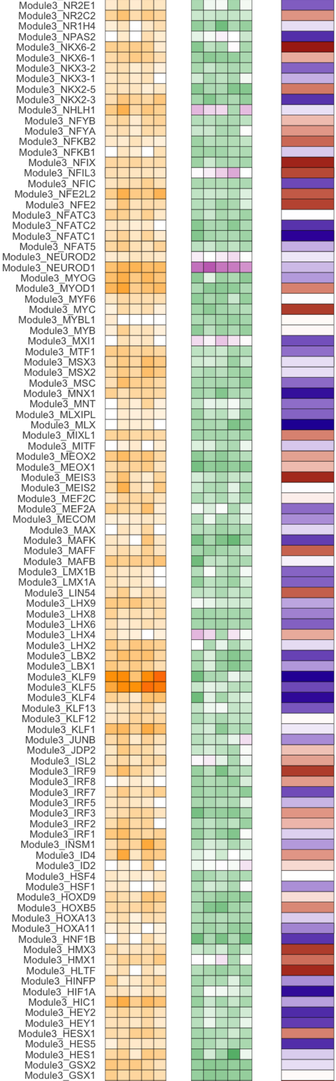
Module3_NR2E1
Module3_NR2C2
Module3_NR1H4
Module3_NPAS2
Module3_NKX6-2
Module3_NKX6-1
Module3_NKX3-2
Module3_NKX3-1
Module3_NKX2-5
Module3_NKX2-3
Module3_NHLH1
Module3_NFYB
Module3_NFYA
Module3_NFKB2
Module3_NFKB1
Module3_NFIX
Module3_NFIL3
Module3_NFIC
Module3_NFE2L2
Module3_NFE2
Module3_NFATC3
Module3_NFATC2
Module3_NFATC1
Module3_NFAT5
Module3_NEUROD2
Module3_NEUROD1
Module3_MYOG
Module3_MYOD1
Module3_MYF6
Module3_MYC
Module3_MYBL1
Module3_MYB
Module3_MXI1
Module3_MTF1
Module3_MSX3
Module3_MSX2
Module3_MSC
Module3_MNX1
Module3_MNT
Module3_MLXIPL
Module3_MLX
Module3_MIXL1
Module3_MITF
Module3_MEOX2
Module3_MEOX1
Module3_MEIS3
Module3_MEIS2
Module3_MEF2C
Module3_MEF2A
Module3_MECOM
Module3_MAX
Module3_MAFK
Module3_MAFF
Module3_MAFB
Module3_LMX1B
Module3_LMX1A
Module3_LIN54
Module3_LHX9
Module3_LHX8
Module3_LHX6
Module3_LHX4
Module3_LHX2
Module3_LBX2
Module3_LBX1
Module3_KLF9
Module3_KLF5
Module3_KLF4
Module3_KLF13
Module3_KLF12
Module3_KLF1
Module3_JUNB
Module3_JDP2
Module3_ISL2
Module3_IRF9
Module3_IRF8
Module3_IRF7
Module3_IRF5
Module3_IRF3
Module3_IRF2
Module3_IRF1
Module3_INSM1
Module3_ID4
Module3_ID2
Module3_HSF4
Module3_HSF1
Module3_HOXD9
Module3_HOXB5
Module3_HOXA13
Module3_HOXA11
Module3_HNF1B
Module3_HMX3
Module3_HMX1
Module3_HLTF
Module3_HINFP
Module3_HIF1A
Module3_HIC1
Module3_HEY2
Module3_HEY1
Module3_HESX1
Module3_HES5
Module3_HES1
Module3_GSX2
Module3_GSX1

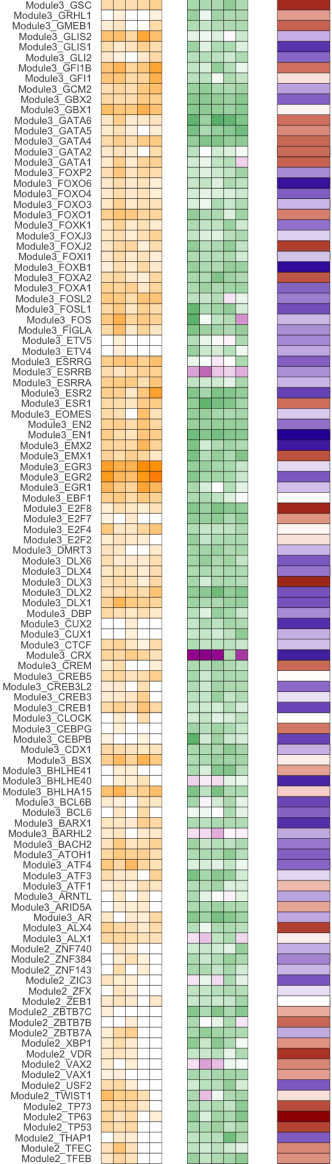
Module3_GSC
Module3_GRHL1
Module3_GMEB1
Module3_GLIS2
Module3_GLIS1
Module3_GLI2
Module3_GFI1B
Module3_GFI1
Module3_GCM2
Module3_GBX2
Module3_GBX1
Module3_GATA6
Module3_GATA5
Module3_GATA4
Module3_GATA2
Module3_GATA1
Module3_FOXP2
Module3_FOXO6
Module3_FOXO4
Module3_FOXO3
Module3_FOXO1
Module3_FOXK1
Module3_FOXJ3
Module3_FOXJ2
Module3_FOXI1
Module3_FOXB1
Module3_FOXA2
Module3_FOXA1
Module3_FOSL2
Module3_FOSL1
Module3_FOS
Module3_FIGLA
Module3_ETV5
Module3_ETV4
Module3_ESRRG
Module3_ESRRB
Module3_ESRRA
Module3_ESR2
Module3_ESR1
Module3_EOMES
Module3_EN2
Module3_EN1
Module3_EMX2
Module3_EMX1
Module3_EGR3
Module3_EGR2
Module3_EGR1
Module3_EBF1
Module3_E2F8
Module3_E2F7
Module3_E2F4
Module3_E2F2
Module3_DMRT3
Module3_DLX6
Module3_DLX4
Module3_DLX3
Module3_DLX2
Module3_DLX1
Module3_DBP
Module3_CUX2
Module3_CUX1
Module3_CTCF
Module3_CRX
Module3_CREM
Module3_CREB5
Module3_CREB3L2
Module3_CREB3
Module3_CREB1
Module3_CLOCK
Module3_CEBPG
Module3_CEBPB
Module3_CDX1
Module3_BSX
Module3_BHLHE41
Module3_BHLHE40
Module3_BHLHA15
Module3_BCL6B
Module3_BCL6
Module3_BARX1
Module3_BARHL2
Module3_BACH2
Module3_ATOH1
Module3_ATF4
Module3_ATF3
Module3_ATF1
Module3_ARNTL
Module3_ARID5A
Module3_AR
Module3_ALX4
Module3_ALX1
Module2_ZNF740
Module2_ZNF384
Module2_ZNF143
Module2_ZIC3
Module2_ZFX
Module2_ZEB1
Module2_ZBTB7C
Module2_ZBTB7B
Module2_ZBTB7A
Module2_XBP1
Module2_VDR
Module2_VAX2
Module2_VAX1
Module2_USF2
Module2_TWIST1
Module2_TP73
Module2_TP63
Module2_TP53
Module2_THAP1
Module2_TFEC
Module2_TFEB

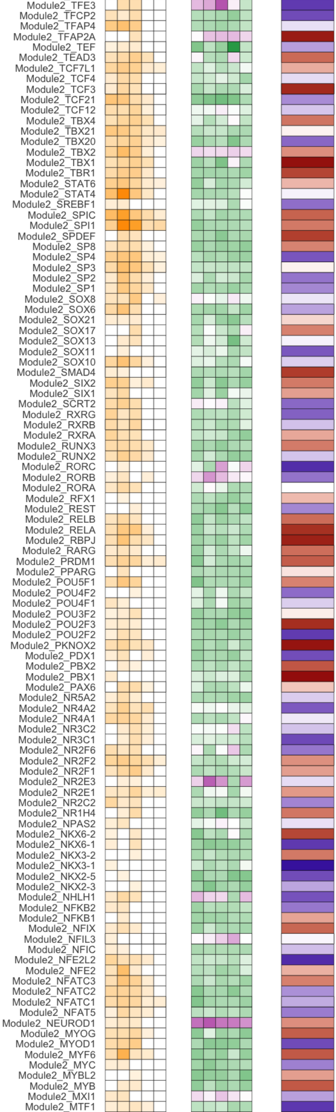
Module2_TFE3
Module2_TFCP2
Module2_TFAP4
Module2_TFAP2A
Module2_TEF
Module2_TEAD3
Module2_TCF7L1
Module2_TCF4
Module2_TCF3
Module2_TCF21
Module2_TCF12
Module2_TBX4
Module2_TBX21
Module2_TBX20
Module2_TBX2
Module2_TBX1
Module2_TBR1
Module2_STAT6
Module2_STAT4
Module2_SREBF1
Module2_SPIC
Module2_SPI1
Module2_SPDEF
Module2_SP8
Module2_SP4
Module2_SP3
Module2_SP2
Module2_SP1
Module2_SOX8
Module2_SOX6
Module2_SOX21
Module2_SOX17
Module2_SOX13
Module2_SOX11
Module2_SOX10
Module2_SMAD4
Module2_SIX2
Module2_SIX1
Module2_SCRT2
Module2_RXRG
Module2_RXRB
Module2_RXRA
Module2_RUNX3
Module2_RUNX2
Module2_RORC
Module2_RORB
Module2_RORA
Module2_RFX1
Module2_REST
Module2_RELB
Module2_RELA
Module2_RBPJ
Module2_RARG
Module2_PRDM1
Module2_PPARG
Module2_POU5F1
Module2_POU4F2
Module2_POU4F1
Module2_POU3F2
Module2_POU2F3
Module2_POU2F2
Module2_PKNOX2
Module2_PDX1
Module2_PBX2
Module2_PBX1
Module2_PAX6
Module2_NR5A2
Module2_NR4A2
Module2_NR4A1
Module2_NR3C2
Module2_NR3C1
Module2_NR2F6
Module2_NR2F2
Module2_NR2F1
Module2_NR2E3
Module2_NR2E1
Module2_NR2C2
Module2_NR1H4
Module2_NPAS2
Module2_NKX6-2
Module2_NKX6-1
Module2_NKX3-2
Module2_NKX3-1
Module2_NKX2-5
Module2_NKX2-3
Module2_NHLH1
Module2_NFKB2
Module2_NFKB1
Module2_NFIX
Module2_NFIL3
Module2_NFIC
Module2_NFE2L2
Module2_NFE2
Module2_NFATC3
Module2_NFATC2
Module2_NFATC1
Module2_NFAT5
Module2_NEUROD1
Module2_MYOG
Module2_MYOD1
Module2_MYF6
Module2_MYC
Module2_MYBL2
Module2_MYB
Module2_MXI1
Module2_MTF1

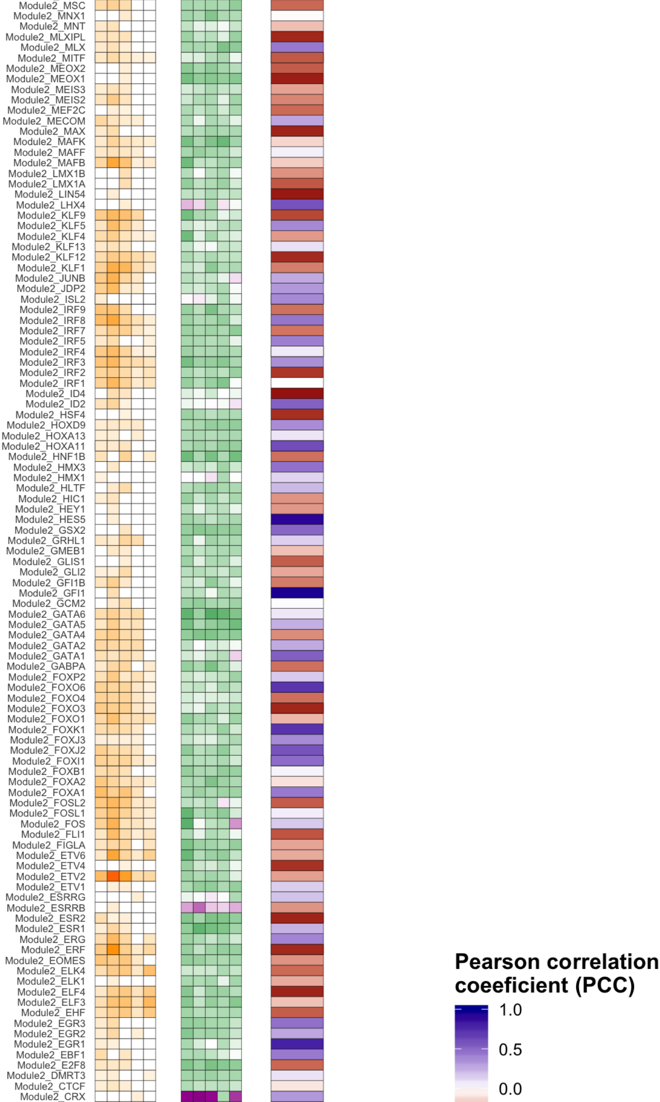
Module2_MSC
Module2_MNX1
Module2_MNT
Module2_MLXIPL
Module2_MLX
Module2_MITF
Module2_MEOX2
Module2_MEOX1
Module2_MEIS3
Module2_MEIS2
Module2_MEF2C
Module2_MECOM
Module2_MAX
Module2_MAFK
Module2_MAFF
Module2_MAFB
Module2_LMX1B
Module2_LMX1A
Module2_LIN54
Module2_LHX4
Module2_KLF9
Module2_KLF5
Module2_KLF4
Module2_KLF13
Module2_KLF12
Module2_KLF1
Module2_JUNB
Module2_JDP2
Module2_ISL2
Module2_IRF9
Module2_IRF8
Module2_IRF7
Module2_IRF5
Module2_IRF4
Module2_IRF3
Module2_IRF2
Module2_IRF1
Module2_ID4
Module2_ID2
Module2_HSF4
Module2_HOXD9
Module2_HOXA13
Module2_HOXA11
Module2_HNF1B
Module2_HMX3
Module2_HMX1
Module2_HLTF
Module2_HIC1
Module2_HEY1
Module2_HES5
Module2_GSX2
Module2_GRHL1
Module2_GMEB1
Module2_GLIS1
Module2_GLI2
Module2_GFI1B
Module2_GFI1
Module2_GCM2
Module2_GATA6
Module2_GATA5
Module2_GATA4
Module2_GATA2
Module2_GATA1
Module2_GABPA
Module2_FOXP2
Module2_FOXO6
Module2_FOXO4
Module2_FOXO3
Module2_FOXO1
Module2_FOXK1
Module2_FOXJ3
Module2_FOXJ2
Module2_FOXI1
Module2_FOXB1
Module2_FOXA2
Module2_FOXA1
Module2_FOSL2
Module2_FOSL1
Module2_FOS
Module2_FLI1
Module2_FIGLA
Module2_ETV6
Module2_ETV4
Module2_ETV2
Module2_ETV1
Module2_ESRRG
Module2_ESRRB
Module2_ESR2
Module2_ESR1
Module2_ERG
Module2_ERF
Module2_EOMES
Module2_ELK4
Module2_ELK1
Module2_ELF4
Module2_ELF3
Module2_EHF
Module2_EGR3
Module2_EGR2
Module2_EGR1
Module2_EBF1
Module2_E2F8
Module2_DMRT3
Module2_CTCF
Module2_CRX
Pearson correlation
coeeficient (PCC)
1.0
0.5
0.0

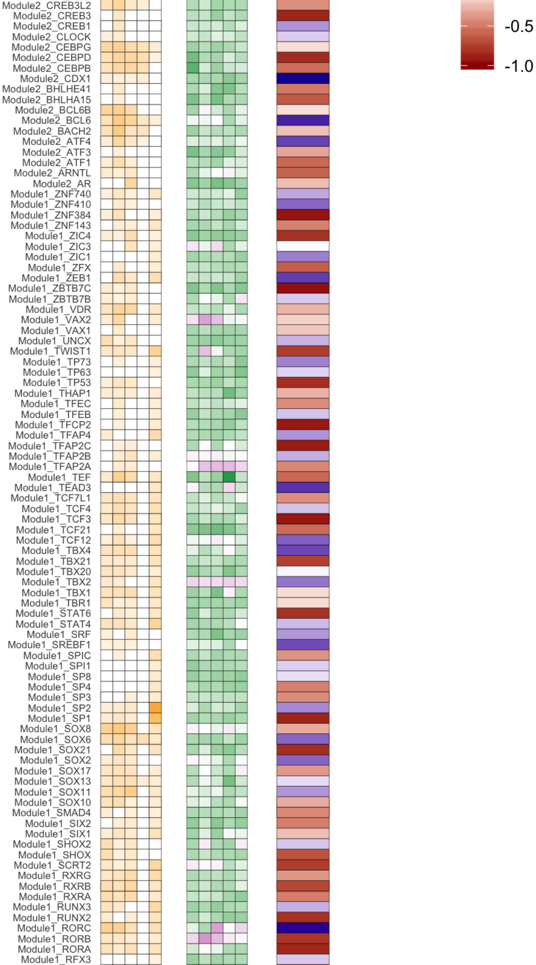

Module2_CREB3L2
Module2_CREB3
Module2_CREB1
Module2_CLOCK
Module2_CEBPG
Module2_CEBPD
Module2_CEBPB
Module2_CDX1
Module2_BHLHE41
Module2_BHLHA15
Module2_BCL6B
Module2_BCL6
Module2_BACH2
Module2_ATF4
Module2_ATF3
Module2_ATF1
Module2_ARNTL
Module2_AR
Module1_ZNF740
Module1_ZNF410
Module1_ZNF384
Module1_ZNF143
Module1_ZIC4
Module1_ZIC3
Module1_ZIC1
Module1_ZFX
Module1_ZEB1
Module1_ZBTB7C
Module1_ZBTB7B
Module1_VDR
Module1_VAX2
Module1_VAX1
Module1_UNCX
Module1_TWIST1
Module1_TP73
Module1_TP63
Module1_TP53
Module1_THAP1
Module1_TFEC
Module1_TFEB
Module1_TFCP2
Module1_TFAP4
Module1_TFAP2C
Module1_TFAP2B
Module1_TFAP2A
Module1_TEF
Module1_TEAD3
Module1_TCF7L1
Module1_TCF4
Module1_TCF3
Module1_TCF21
Module1_TCF12
Module1_TBX4
Module1_TBX21
Module1_TBX20
Module1_TBX2
Module1_TBX1
Module1_TBR1
Module1_STAT6
Module1_STAT4
Module1_SRF
Module1_SREBF1
Module1_SPIC
Module1_SPI1
Module1_SP8
Module1_SP4
Module1_SP3
Module1_SP2
Module1_SP1
Module1_SOX8
Module1_SOX6
Module1_SOX21
Module1_SOX2
Module1_SOX17
Module1_SOX13
Module1_SOX11
Module1_SOX10
Module1_SMAD4
Module1_SIX2
Module1_SIX1
Module1_SHOX2
Module1_SHOX
Module1_SCRT2
Module1_RXRG
Module1_RXRB
Module1_RXRA
Module1_RUNX3
Module1_RUNX2
Module1_RORC
Module1_RORB
Module1_RORA
Module1_RFX3
-0.5
-1.0

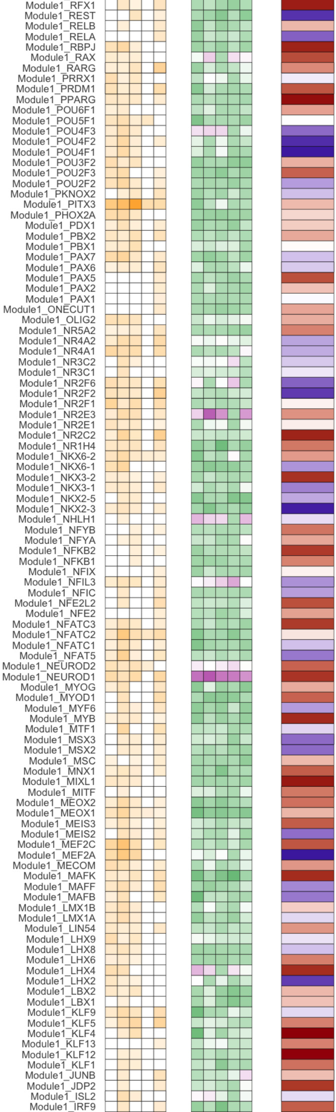
Module1_RFX1
Module1_REST
Module1_RELB
Module1_RELA
Module1_RBPJ
Module1_RAX
Module1_RARG
Module1_PRRX1
Module1_PRDM1
Module1_PPARG
Module1_POU6F1
Module1_POU5F1
Module1_POU4F3
Module1_POU4F2
Module1_POU4F1
Module1_POU3F2
Module1_POU2F3
Module1_POU2F2
Module1_PKNOX2
Module1_PITX3
Module1_PHOX2A
Module1_PDX1
Module1_PBX2
Module1_PBX1
Module1_PAX7
Module1_PAX6
Module1_PAX5
Module1_PAX2
Module1_PAX1
Module1_ONECUT1
Module1_OLIG2
Module1_NR5A2
Module1_NR4A2
Module1_NR4A1
Module1_NR3C2
Module1_NR3C1
Module1_NR2F6
Module1_NR2F2
Module1_NR2F1
Module1_NR2E3
Module1_NR2E1
Module1_NR2C2
Module1_NR1H4
Module1_NKX6-2
Module1_NKX6-1
Module1_NKX3-2
Module1_NKX3-1
Module1_NKX2-5
Module1_NKX2-3
Module1_NHLH1
Module1_NFYB
Module1_NFYA
Module1_NFKB2
Module1_NFKB1
Module1_NFIX
Module1_NFIL3
Module1_NFIC
Module1_NFE2L2
Module1_NFE2
Module1_NFATC3
Module1_NFATC2
Module1_NFATC1
Module1_NFAT5
Module1_NEUROD2
Module1_NEUROD1
Module1_MYOG
Module1_MYOD1
Module1_MYF6
Module1_MYB
Module1_MTF1
Module1_MSX3
Module1_MSX2
Module1_MSC
Module1_MNX1
Module1_MIXL1
Module1_MITF
Module1_MEOX2
Module1_MEOX1
Module1_MEIS3
Module1_MEIS2
Module1_MEF2C
Module1_MEF2A
Module1_MECOM
Module1_MAFK
Module1_MAFF
Module1_MAFB
Module1_LMX1B
Module1_LMX1A
Module1_LIN54
Module1_LHX9
Module1_LHX8
Module1_LHX6
Module1_LHX4
Module1_LHX2
Module1_LBX2
Module1_LBX1
Module1_KLF9
Module1_KLF5
Module1_KLF4
Module1_KLF13
Module1_KLF12
Module1_KLF1
Module1_JUNB
Module1_JDP2
Module1_ISL2
Module1_IRF9

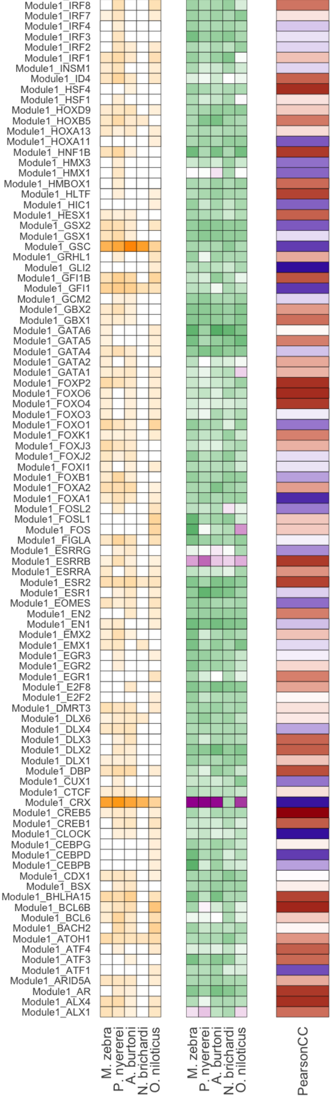
Module1_IRF8
Module1_IRF7
Module1_IRF4
Module1_IRF3
Module1_IRF2
Module1_IRF1
Module1_INSM1
Module1_ID4
Module1_HSF4
Module1_HSF1
Module1_HOXD9
Module1_HOXB5
Module1_HOXA13
Module1_HOXA11
Module1_HNF1B
Module1_HMX3
Module1_HMX1
Module1_HMBOX1
Module1_HLTF
Module1_HIC1
Module1_HESX1
Module1_GSX2
Module1_GSX1
Module1_GSC
Module1_GRHL1
Module1_GLI2
Module1_GFI1B
Module1_GFI1
Module1_GCM2
Module1_GBX2
Module1_GBX1
Module1_GATA6
Module1_GATA5
Module1_GATA4
Module1_GATA2
Module1_GATA1
Module1_FOXP2
Module1_FOXO6
Module1_FOXO4
Module1_FOXO3
Module1_FOXO1
Module1_FOXK1
Module1_FOXJ3
Module1_FOXJ2
Module1_FOXI1
Module1_FOXB1
Module1_FOXA2
Module1_FOXA1
Module1_FOSL2
Module1_FOSL1
Module1_FOS
Module1_FIGLA
Module1_ESRRG
Module1_ESRRB
Module1_ESRRA
Module1_ESR2
Module1_ESR1
Module1_EOMES
Module1_EN2
Module1_EN1
Module1_EMX2
Module1_EMX1
Module1_EGR3
Module1_EGR2
Module1_EGR1
Module1_E2F8
Module1_E2F2
Module1_DMRT3
Module1_DLX6
Module1_DLX4
Module1_DLX3
Module1_DLX2
Module1_DLX1
Module1_DBP
Module1_CUX1
Module1_CTCF
Module1_CRX
Module1_CREB5
Module1_CREB1
Module1_CLOCK
Module1_CEBPG
Module1_CEBPD
Module1_CEBPB
Module1_CDX1
Module1_BSX
Module1_BHLHA15
Module1_BCL6B
Module1_BCL6
Module1_BACH2
Module1_ATOH1
Module1_ATF4
Module1_ATF3
Module1_ATF1
Module1_ARID5A
Module1_AR
Module1_ALX4
Module1_ALX1
M. zebra
P. nyererei
A. burtoni
N. brichardi
O. niloticus
M. zebra
P. nyererei
A. burtoni
N. brichardi
O. niloticus
PearsonCC

Additional file 2: Fig. S5

Heatmap matrices of enrichment, expression and Pearson correlation between the two for each motif, across heart tissue

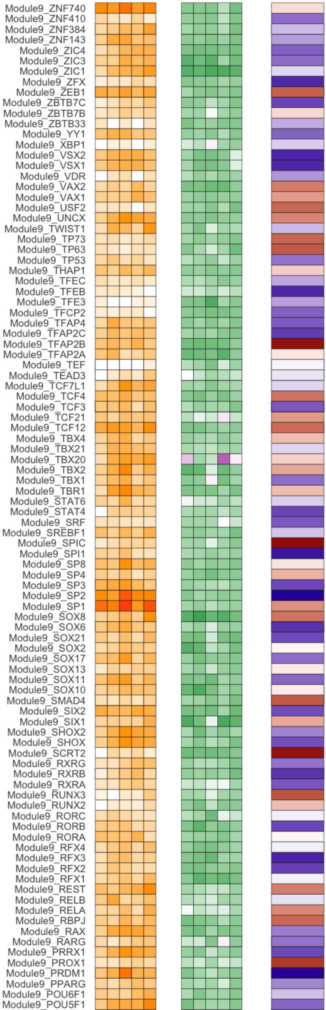

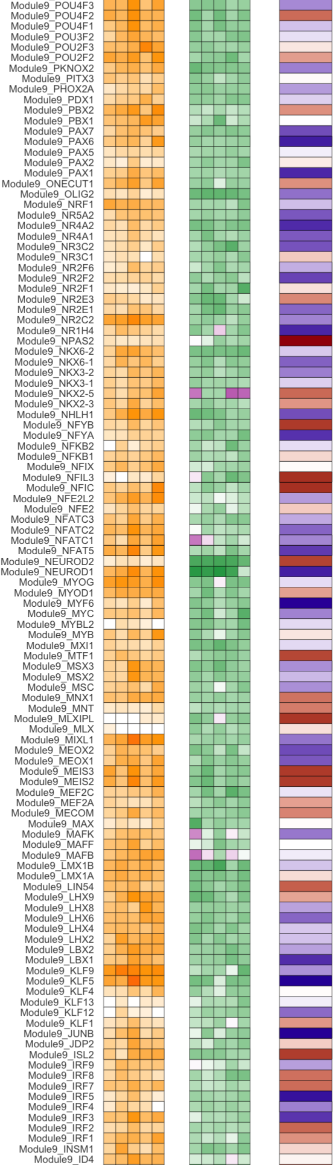

Module9_POU4F3
Module9_POU4F2
Module9_POU4F1
Module9_POU3F2
Module9_POU2F3
Module9_POU2F2
Module9_PKNOX2
Module9_PITX3
Module9_PHOX2A
Module9_PDX1
Module9_PBX2
Module9_PBX1
Module9_PAX7
Module9_PAX6
Module9_PAX5
Module9_PAX2
Module9_PAX1
Module9_ONECUT1
Module9_OLIG2
Module9_NRF1
Module9_NR5A2
Module9_NR4A2
Module9_NR4A1
Module9_NR3C2
Module9_NR3C1
Module9_NR2F6
Module9_NR2F2
Module9_NR2F1
Module9_NR2E3
Module9_NR2E1
Module9_NR2C2
Module9_NR1H4
Module9_NPAS2
Module9_NKX6-2
Module9_NKX6-1
Module9_NKX3-2
Module9_NKX3-1
Module9_NKX2-5
Module9_NKX2-3
Module9_NHLH1
Module9_NFYB
Module9_NFYA
Module9_NFKB2
Module9_NFKB1
Module9_NFIX
Module9_NFIL3
Module9_NFIC
Module9_NFE2L2
Module9_NFE2
Module9_NFATC3
Module9_NFATC2
Module9_NFATC1
Module9_NFAT5
Module9_NEUROD2
Module9_NEUROD1
Module9_MYOG
Module9_MYOD1
Module9_MYF6
Module9_MYC
Module9_MYBL2
Module9_MYB
Module9_MXI1
Module9_MTF1
Module9_MSX3
Module9_MSX2
Module9_MSC
Module9_MNX1
Module9_MNT
Module9_MLXIPL
Module9_MLX
Module9_MIXL1
Module9_MEOX2
Module9_MEOX1
Module9_MEIS3
Module9_MEIS2
Module9_MEF2C
Module9_MEF2A
Module9_MECOM
Module9_MAX
Module9_MAFK
Module9_MAFF
Module9_MAFB
Module9_LMX1B
Module9_LMX1A
Module9_LIN54
Module9_LHX9
Module9_LHX8
Module9_LHX6
Module9_LHX4
Module9_LHX2
Module9_LBX2
Module9_LBX1
Module9_KLF9
Module9_KLF5
Module9_KLF4
Module9_KLF13
Module9_KLF12
Module9_KLF1
Module9_JUNB
Module9_JDP2
Module9_ISL2
Module9_IRF9
Module9_IRF8
Module9_IRF7
Module9_IRF5
Module9_IRF4
Module9_IRF3
Module9_IRF2
Module9_IRF1
Module9_INSM1
Module9_ID4

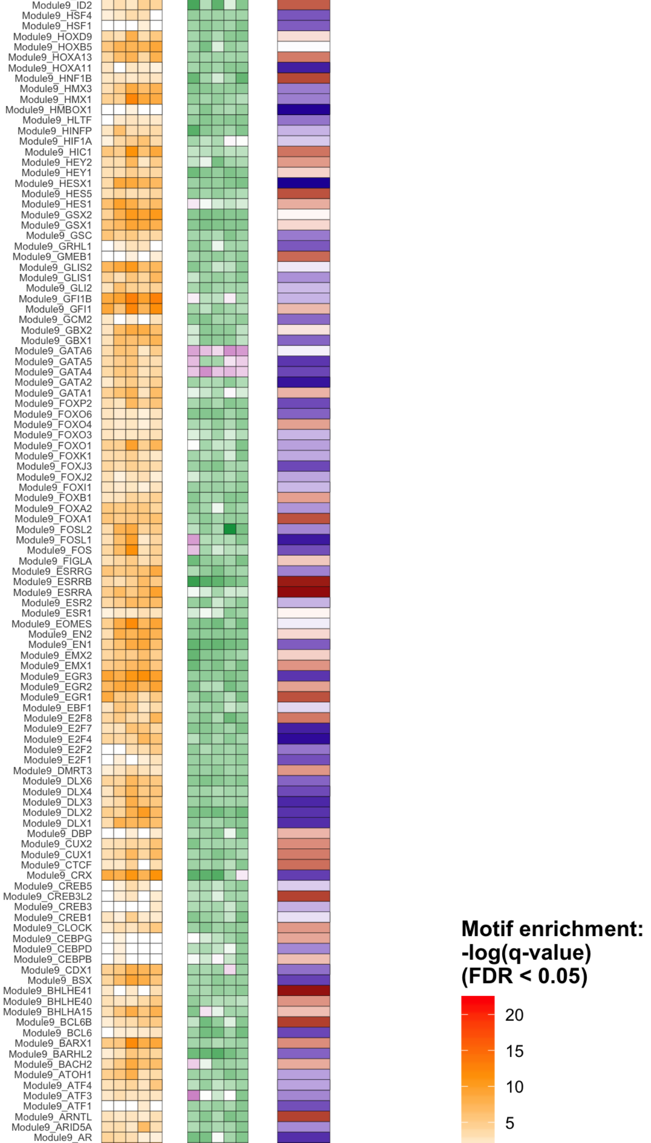
Module9_ID2
Module9_HSF4
Module9_HSF1
Module9_HOXD9
Module9_HOXB5
Module9_HOXA13
Module9_HOXA11
Module9_HNF1B
Module9_HMX3
Module9_HMX1
Module9_HMBOX1
Module9_HLTF
Module9_HINFP
Module9_HIF1A
Module9_HIC1
Module9_HEY2
Module9_HEY1
Module9_HESX1
Module9_HES5
Module9_HES1
Module9_GSX2
Module9_GSX1
Module9_GSC
Module9_GRHL1
Module9_GMEB1
Module9_GLIS2
Module9_GLIS1
Module9_GLI2
Module9_GFI1B
Module9_GFI1
Module9_GCM2
Module9_GBX2
Module9_GBX1
Module9_GATA6
Module9_GATA5
Module9_GATA4
Module9_GATA2
Module9_GATA1
Module9_FOXP2
Module9_FOXO6
Module9_FOXO4
Module9_FOXO3
Module9_FOXO1
Module9_FOXK1
Module9_FOXJ3
Module9_FOXJ2
Module9_FOXI1
Module9_FOXB1
Module9_FOXA2
Module9_FOXA1
Module9_FOSL2
Module9_FOSL1
Module9_FOS
Module9_FIGLA
Module9_ESRRG
Module9_ESRRB
Module9_ESRRA
Module9_ESR2
Module9_ESR1
Module9_EOMES
Module9_EN2
Module9_EN1
Module9_EMX2
Module9_EMX1
Module9_EGR3
Module9_EGR2
Module9_EGR1
Module9_EBF1
Module9_E2F8
Module9_E2F7
Module9_E2F4
Module9_E2F2
Module9_E2F1
Module9_DMRT3
Module9_DLX6
Module9_DLX4
Module9_DLX3
Module9_DLX2
Module9_DLX1
Module9_DBP
Module9_CUX2
Module9_CUX1
Module9_CTCF
Module9_CRX
Module9_CREB5
Module9_CREB3L2
Module9_CREB3
Module9_CREB1
Module9_CLOCK
Module9_CEBPG
Module9_CEBPD
Module9_CEBPB
Module9_CDX1
Module9_BSX
Module9_BHLHE41
Module9_BHLHE40
Module9_BHLHA15
Module9_BCL6B
Module9_BCL6
Module9_BARX1
Module9_BARHL2
Module9_BACH2
Module9_ATOH1
Module9_ATF4
Module9_ATF3
Module9_ATF1
Module9_ARNTL
Module9_ARID5A
Module9_AR
Motif enrichment:
-log(q-value)
(FDR < 0.05)
20
15
10
5

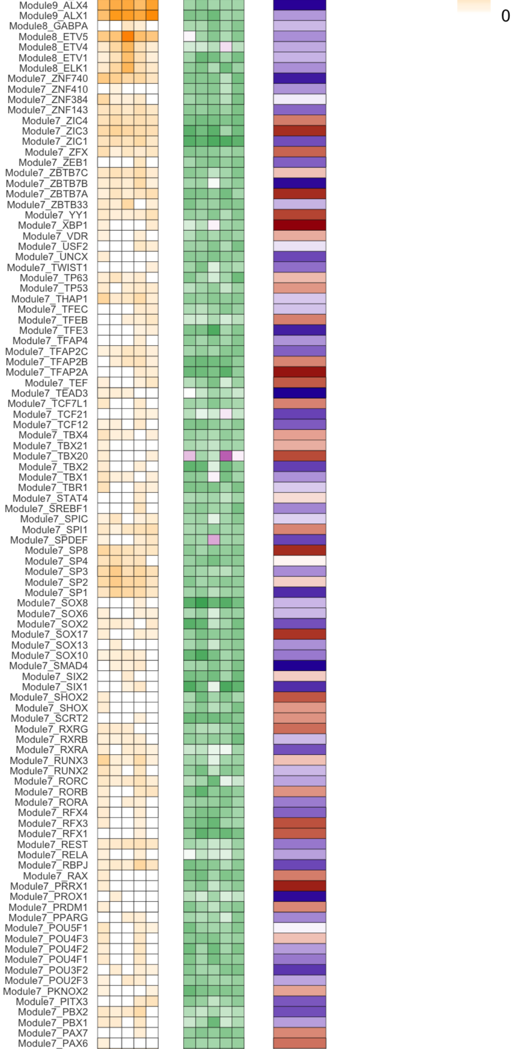
Module9_ALX4
Module9_ALX1
Module8_GABPA
Module8_ETV5
Module8_ETV4
Module8_ETV1
Module8_ELK1
Module7_ZNF740
Module7_ZNF410
Module7_ZNF384
Module7_ZNF143
Module7_ZIC4
Module7_ZIC3
Module7_ZIC1
Module7_ZFX
Module7_ZEB1
Module7_ZBTB7C
Module7_ZBTB7B
Module7_ZBTB7A
Module7_ZBTB33
Module7_YY1
Module7_XBP1
Module7_VDR
Module7_USF2
Module7_UNCX
Module7_TWIST1
Module7_TP63
Module7_TP53
Module7_THAP1
Module7_TFEC
Module7_TFEB
Module7_TFE3
Module7_TFAP4
Module7_TFAP2C
Module7_TFAP2B
Module7_TFAP2A
Module7_TEF
Module7_TEAD3
Module7_TCF7L1
Module7_TCF21
Module7_TCF12
Module7_TBX4
Module7_TBX21
Module7_TBX20
Module7_TBX2
Module7_TBX1
Module7_TBR1
Module7_STAT4
Module7_SREBF1
Module7_SPIC
Module7_SPI1
Module7_SPDEF
Module7_SP8
Module7_SP4
Module7_SP3
Module7_SP2
Module7_SP1
Module7_SOX8
Module7_SOX6
Module7_SOX2
Module7_SOX17
Module7_SOX13
Module7_SOX10
Module7_SMAD4
Module7_SIX2
Module7_SIX1
Module7_SHOX2
Module7_SHOX
Module7_SCRT2
Module7_RXRG
Module7_RXRB
Module7_RXRA
Module7_RUNX3
Module7_RUNX2
Module7_RORC
Module7_RORB
Module7_RORA
Module7_RFX4
Module7_RFX3
Module7_RFX1
Module7_REST
Module7_RELA
Module7_RBPJ
Module7_RAX
Module7_PRRX1
Module7_PROX1
Module7_PRDM1
Module7_PPARG
Module7_POU5F1
Module7_POU4F3
Module7_POU4F2
Module7_POU4F1
Module7_POU3F2
Module7_POU2F3
Module7_PKNOX2
Module7_PITX3
Module7_PBX2
Module7_PBX1
Module7_PAX7
Module7_PAX6
0

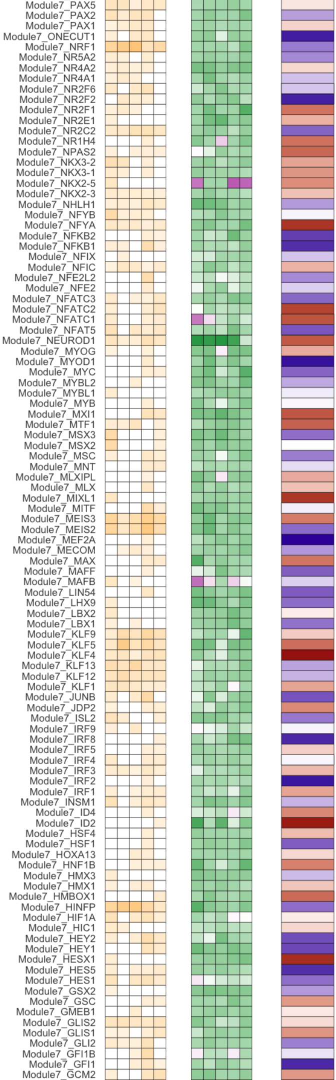
Module7_PAX5
Module7_PAX2
Module7_PAX1
Module7_ONECUT1
Module7_NRF1
Module7_NR5A2
Module7_NR4A2
Module7_NR4A1
Module7_NR2F6
Module7_NR2F2
Module7_NR2F1
Module7_NR2E1
Module7_NR2C2
Module7_NR1H4
Module7_NPAS2
Module7_NKX3-2
Module7_NKX3-1
Module7_NKX2-5
Module7_NKX2-3
Module7_NHLH1
Module7_NFYB
Module7_NFYA
Module7_NFKB2
Module7_NFKB1
Module7_NFIX
Module7_NFIC
Module7_NFE2L2
Module7_NFE2
Module7_NFATC3
Module7_NFATC2
Module7_NFATC1
Module7_NFAT5
Module7_NEUROD1
Module7_MYOG
Module7_MYOD1
Module7_MYC
Module7_MYBL2
Module7_MYBL1
Module7_MYB
Module7_MXI1
Module7_MTF1
Module7_MSX3
Module7_MSX2
Module7_MSC
Module7_MNT
Module7_MLXIPL
Module7_MLX
Module7_MIXL1
Module7_MITF
Module7_MEIS3
Module7_MEIS2
Module7_MEF2A
Module7_MECOM
Module7_MAX
Module7_MAFF
Module7_MAFB
Module7_LIN54
Module7_LHX9
Module7_LBX2
Module7_LBX1
Module7_KLF9
Module7_KLF5
Module7_KLF4
Module7_KLF13
Module7_KLF12
Module7_KLF1
Module7_JUNB
Module7_JDP2
Module7_ISL2
Module7_IRF9
Module7_IRF8
Module7_IRF5
Module7_IRF4
Module7_IRF3
Module7_IRF2
Module7_IRF1
Module7_INSM1
Module7_ID4
Module7_ID2
Module7_HSF4
Module7_HSF1
Module7_HOXA13
Module7_HNF1B
Module7_HMX3
Module7_HMX1
Module7_HMBOX1
Module7_HINFP
Module7_HIF1A
Module7_HIC1
Module7_HEY2
Module7_HEY1
Module7_HESX1
Module7_HES5
Module7_HES1
Module7_GSX2
Module7_GSC
Module7_GMEB1
Module7_GLIS2
Module7_GLIS1
Module7_GLI2
Module7_GFI1B
Module7_GFI1
Module7_GCM2

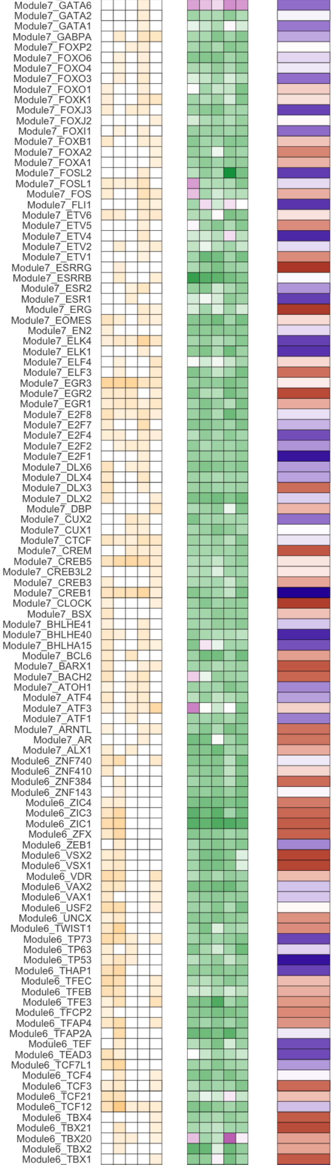
Module7_GATA6
Module7_GATA2
Module7_GATA1
Module7_GABPA
Module7_FOXP2
Module7_FOXO6
Module7_FOXO4
Module7_FOXO3
Module7_FOXO1
Module7_FOXK1
Module7_FOXJ3
Module7_FOXJ2
Module7_FOXI1
Module7_FOXB1
Module7_FOXA2
Module7_FOXA1
Module7_FOSL2
Module7_FOSL1
Module7_FOS
Module7_FLI1
Module7_ETV6
Module7_ETV5
Module7_ETV4
Module7_ETV2
Module7_ETV1
Module7_ESRRG
Module7_ESRRB
Module7_ESR2
Module7_ESR1
Module7_ERG
Module7_EOMES
Module7_EN2
Module7_ELK4
Module7_ELK1
Module7_ELF4
Module7_ELF3
Module7_EGR3
Module7_EGR2
Module7_EGR1
Module7_E2F8
Module7_E2F7
Module7_E2F4
Module7_E2F2
Module7_E2F1
Module7_DLX6
Module7_DLX4
Module7_DLX3
Module7_DLX2
Module7_DBP
Module7_CUX2
Module7_CUX1
Module7_CTCF
Module7_CREM
Module7_CREB5
Module7_CREB3L2
Module7_CREB3
Module7_CREB1
Module7_CLOCK
Module7_BSX
Module7_BHLHE41
Module7_BHLHE40
Module7_BHLHA15
Module7_BCL6
Module7_BARX1
Module7_BACH2
Module7_ATOH1
Module7_ATF4
Module7_ATF3
Module7_ATF1
Module7_ARNTL
Module7_AR
Module7_ALX1
Module6_ZNF740
Module6_ZNF410
Module6_ZNF384
Module6_ZNF143
Module6_ZIC4
Module6_ZIC3
Module6_ZIC1
Module6_ZFX
Module6_ZEB1
Module6_VSX2
Module6_VSX1
Module6_VDR
Module6_VAX2
Module6_VAX1
Module6_USF2
Module6_UNCX
Module6_TWIST1
Module6_TP73
Module6_TP63
Module6_TP53
Module6_THAP1
Module6_TFEC
Module6_TFEB
Module6_TFE3
Module6_TFCP2
Module6_TFAP4
Module6_TFAP2A
Module6_TEF
Module6_TEAD3
Module6_TCF7L1
Module6_TCF4
Module6_TCF3
Module6_TCF21
Module6_TCF12
Module6_TBX4
Module6_TBX21
Module6_TBX20
Module6_TBX2
Module6_TBX1

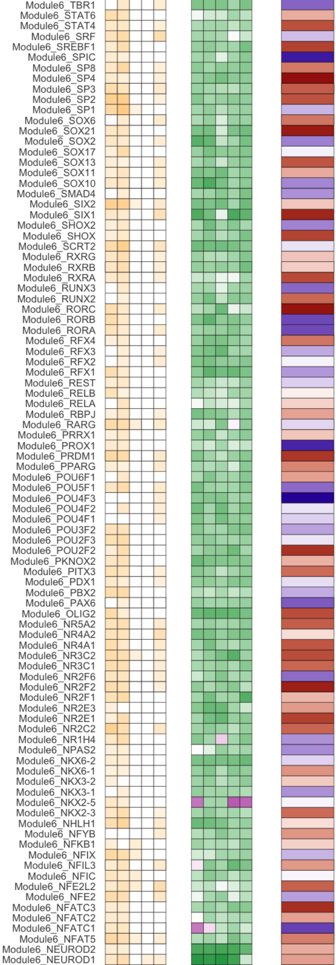
Module6_TBR1
Module6_STAT6
Module6_STAT4
Module6_SRF
Module6_SREBF1
Module6_SPIC
Module6_SP8
Module6_SP4
Module6_SP3
Module6_SP2
Module6_SP1
Module6_SOX6
Module6_SOX21
Module6_SOX2
Module6_SOX17
Module6_SOX13
Module6_SOX11
Module6_SOX10
Module6_SMAD4
Module6_SIX2
Module6_SIX1
Module6_SHOX2
Module6_SHOX
Module6_SCRT2
Module6_RXRG
Module6_RXRB
Module6_RXRA
Module6_RUNX3
Module6_RUNX2
Module6_RORC
Module6_RORB
Module6_RORA
Module6_RFX4
Module6_RFX3
Module6_RFX2
Module6_RFX1
Module6_REST
Module6_RELB
Module6_RELA
Module6_RBPJ
Module6_RARG
Module6_PRRX1
Module6_PROX1
Module6_PRDM1
Module6_PPARG
Module6_POU6F1
Module6_POU5F1
Module6_POU4F3
Module6_POU4F2
Module6_POU4F1
Module6_POU3F2
Module6_POU2F3
Module6_POU2F2
Module6_PKNOX2
Module6_PITX3
Module6_PDX1
Module6_PBX2
Module6_PAX6
Module6_OLIG2
Module6_NR5A2
Module6_NR4A2
Module6_NR4A1
Module6_NR3C2
Module6_NR3C1
Module6_NR2F6
Module6_NR2F2
Module6_NR2F1
Module6_NR2E3
Module6_NR2E1
Module6_NR2C2
Module6_NR1H4
Module6_NPAS2
Module6_NKX6-2
Module6_NKX6-1
Module6_NKX3-2
Module6_NKX3-1
Module6_NKX2-5
Module6_NKX2-3
Module6_NHLH1
Module6_NFYB
Module6_NFKB1
Module6_NFIX
Module6_NFIL3
Module6_NFIC
Module6_NFE2L2
Module6_NFE2
Module6_NFATC3
Module6_NFATC2
Module6_NFATC1
Module6_NFAT5
Module6_NEUROD2
Module6_NEUROD1

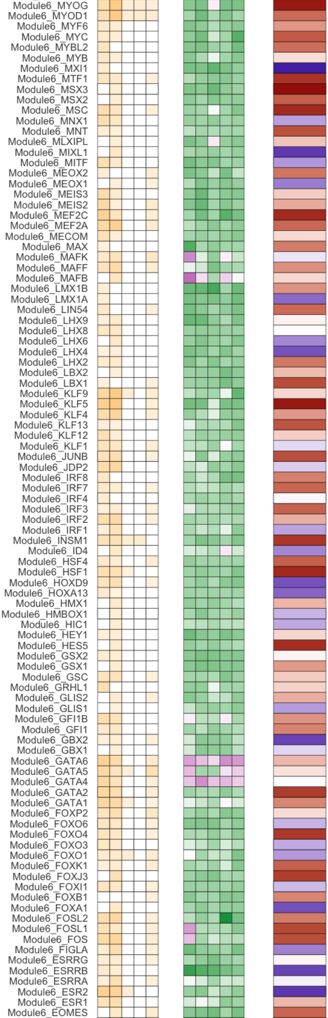
Module6_MYOG
Module6_MYOD1
Module6_MYF6
Module6_MYC
Module6_MYBL2
Module6_MYB
Module6_MXI1
Module6_MTF1
Module6_MSX3
Module6_MSX2
Module6_MSC
Module6_MNX1
Module6_MNT
Module6_MLXIPL
Module6_MIXL1
Module6_MITF
Module6_MEOX2
Module6_MEOX1
Module6_MEIS3
Module6_MEIS2
Module6_MEF2C
Module6_MEF2A
Module6_MECOM
Module6_MAX
Module6_MAFK
Module6_MAFF
Module6_MAFB
Module6_LMX1B
Module6_LMX1A
Module6_LIN54
Module6_LHX9
Module6_LHX8
Module6_LHX6
Module6_LHX4
Module6_LHX2
Module6_LBX2
Module6_LBX1
Module6_KLF9
Module6_KLF5
Module6_KLF4
Module6_KLF13
Module6_KLF12
Module6_KLF1
Module6_JUNB
Module6_JDP2
Module6_IRF8
Module6_IRF7
Module6_IRF4
Module6_IRF3
Module6_IRF2
Module6_IRF1
Module6_INSM1
Module6_ID4
Module6_HSF4
Module6_HSF1
Module6_HOXD9
Module6_HOXA13
Module6_HMX1
Module6_HMBOX1
Module6_HIC1
Module6_HEY1
Module6_HES5
Module6_GSX2
Module6_GSX1
Module6_GSC
Module6_GRHL1
Module6_GLIS2
Module6_GLIS1
Module6_GFI1B
Module6_GFI1
Module6_GBX2
Module6_GBX1
Module6_GATA6
Module6_GATA5
Module6_GATA4
Module6_GATA2
Module6_GATA1
Module6_FOXP2
Module6_FOXO6
Module6_FOXO4
Module6_FOXO3
Module6_FOXO1
Module6_FOXK1
Module6_FOXJ3
Module6_FOXI1
Module6_FOXB1
Module6_FOXA1
Module6_FOSL2
Module6_FOSL1
Module6_FOS
Module6_FIGLA
Module6_ESRRG
Module6_ESRRB
Module6_ESRRA
Module6_ESR2
Module6_ESR1
Module6_EOMES

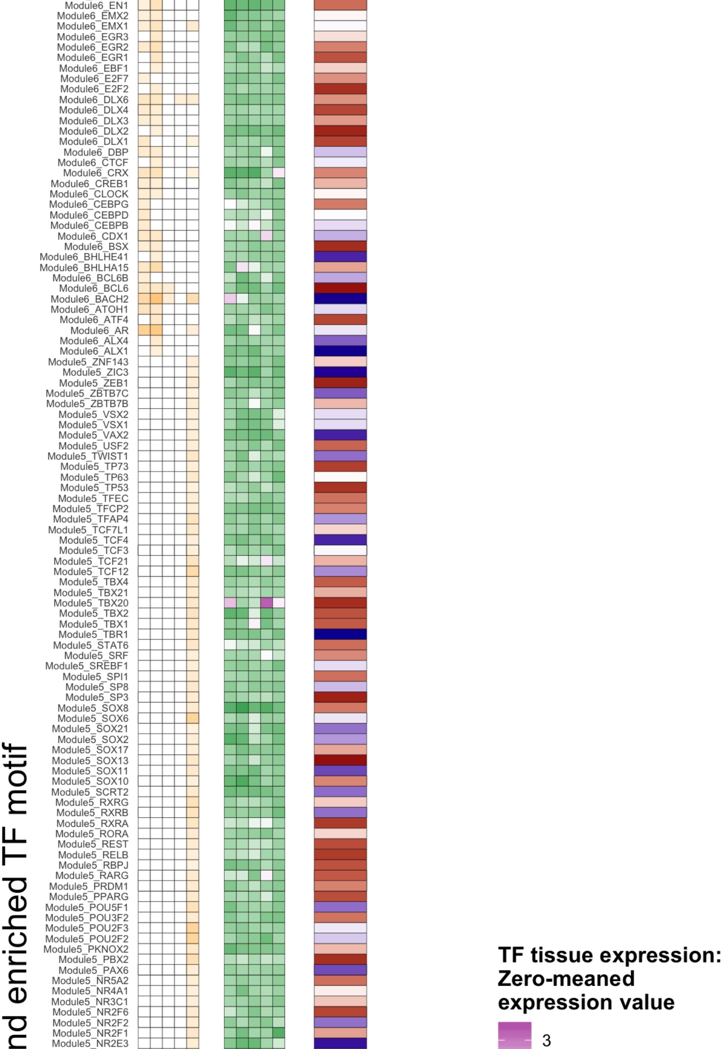
Module6_EN1
Module6_EMX2
Module6_EMX1
Module6_EGR3
Module6_EGR2
Module6_EGR1
Module6_EBF1
Module6_E2F7
Module6_E2F2
Module6_DLX6
Module6_DLX4
Module6_DLX3
Module6_DLX2
Module6_DLX1
Module6_DBP
Module6_CTCF
Module6_CRX
Module6_CREB1
Module6_CLOCK
Module6_CEBPG
Module6_CEBPD
Module6_CEBPB
Module6_CDX1
Module6_BSX
Module6_BHLHE41
Module6_BHLHA15
Module6_BCL6B
Module6_BCL6
Module6_BACH2
Module6_ATOH1
Module6_ATF4
Module6_AR
Module6_ALX4
Module6_ALX1
Module5_ZNF143
Module5_ZIC3
Module5_ZEB1
Module5_ZBTB7C
Module5_ZBTB7B
Module5_VSX2
Module5_VSX1
Module5_VAX2
Module5_USF2
Module5_TWIST1
Module5_TP73
Module5_TP63
Module5_TP53
Module5_TFEC
Module5_TFCP2
Module5_TFAP4
Module5_TCF7L1
Module5_TCF4
Module5_TCF3
Module5_TCF21
Module5_TCF12
Module5_TBX4
Module5_TBX21
Module5_TBX20
Module5_TBX2
Module5_TBX1
Module5_TBR1
Module5_STAT6
Module5_SRF
Module5_SREBF1
Module5_SPI1
Module5_SP8
Module5_SP3
Module5_SOX8
Module5_SOX6
Module5_SOX21
Module5_SOX2
Module5_SOX17
Module5_SOX13
Module5_SOX11
Module5_SOX10
Module5_SCRT2
Module5_RXRG
Module5_RXRB
Module5_RXRA
Module5_RORA
Module5_REST
Module5_RELB
Module5_RBPJ
Module5_RARG
Module5_PRDM1
Module5_PPARG
Module5_POU5F1
Module5_POU3F2
Module5_POU2F3
Module5_POU2F2
Module5_PKNOX2
Module5_PBX2
Module5_PAX6
Module5_NR5A2
Module5_NR4A1
Module5_NR3C1
Module5_NR2F6
Module5_NR2F2
Module5_NR2F1
Module5_NR2E3
nd enriched TF motif
TF tissue expression:
Zero-meaned
expression value
3

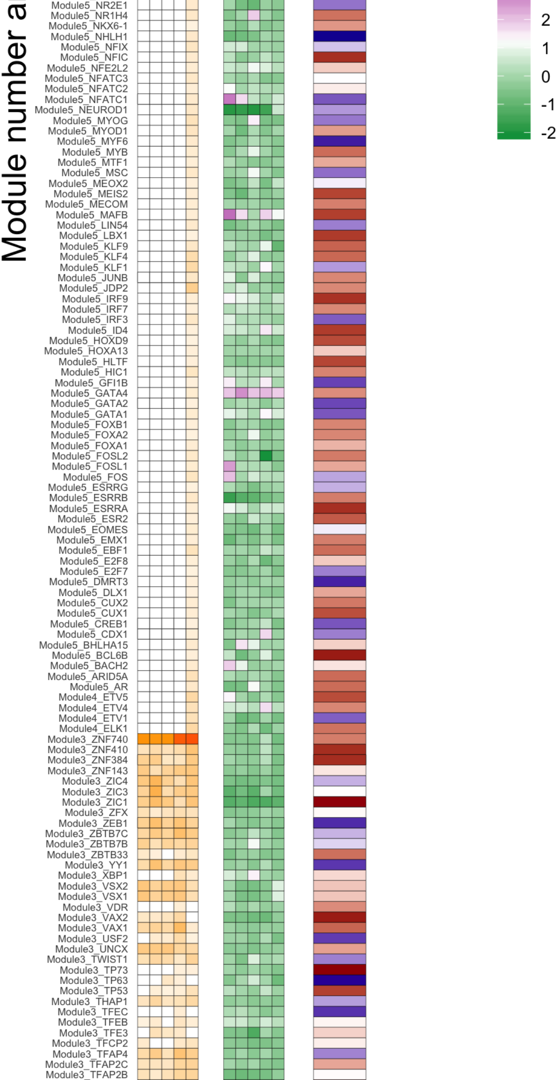
Module number a
Module5_NR2E1
Module5_NR1H4
Module5_NKX6-1
Module5_NHLH1
Module5_NFIX
Module5_NFIC
Module5_NFE2L2
Module5_NFATC3
Module5_NFATC2
Module5_NFATC1
Module5_NEUROD1
Module5_MYOG
Module5_MYOD1
Module5_MYF6
Module5_MYB
Module5_MTF1
Module5_MSC
Module5_MEOX2
Module5_MEIS2
Module5_MECOM
Module5_MAFB
Module5_LIN54
Module5_LBX1
Module5_KLF9
Module5_KLF4
Module5_KLF1
Module5_JUNB
Module5_JDP2
Module5_IRF9
Module5_IRF7
Module5_IRF3
Module5_ID4
Module5_HOXD9
Module5_HOXA13
Module5_HLTF
Module5_HIC1
Module5_GFI1B
Module5_GATA4
Module5_GATA2
Module5_GATA1
Module5_FOXB1
Module5_FOXA2
Module5_FOXA1
Module5_FOSL2
Module5_FOSL1
Module5_FOS
Module5_ESRRG
Module5_ESRRB
Module5_ESRRA
Module5_ESR2
Module5_EOMES
Module5_EMX1
Module5_EBF1
Module5_E2F8
Module5_E2F7
Module5_DMRT3
Module5_DLX1
Module5_CUX2
Module5_CUX1
Module5_CREB1
Module5_CDX1
Module5_BHLHA15
Module5_BCL6B
Module5_BACH2
Module5_ARID5A
Module5_AR
Module4_ETV5
Module4_ETV4
Module4_ETV1
Module4_ELK1
Module3_ZNF740
Module3_ZNF410
Module3_ZNF384
Module3_ZNF143
Module3_ZIC4
Module3_ZIC3
Module3_ZIC1
Module3_ZFX
Module3_ZEB1
Module3_ZBTB7C
Module3_ZBTB7B
Module3_ZBTB33
Module3_YY1
Module3_XBP1
Module3_VSX2
Module3_VSX1
Module3_VDR
Module3_VAX2
Module3_VAX1
Module3_USF2
Module3_UNCX
Module3_TWIST1
Module3_TP73
Module3_TP63
Module3_TP53
Module3_THAP1
Module3_TFEC
Module3_TFEB
Module3_TFE3
Module3_TFCP2
Module3_TFAP4
Module3_TFAP2C
Module3_TFAP2B
2
1
0
-1
-2

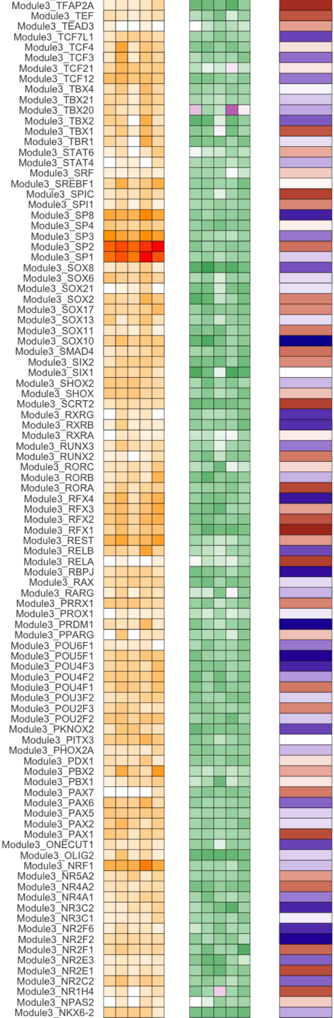
Module3_TFAP2A
Module3_TEF
Module3_TEAD3
Module3_TCF7L1
Module3_TCF4
Module3_TCF3
Module3_TCF21
Module3_TCF12
Module3_TBX4
Module3_TBX21
Module3_TBX20
Module3_TBX2
Module3_TBX1
Module3_TBR1
Module3_STAT6
Module3_STAT4
Module3_SRF
Module3_SREBF1
Module3_SPIC
Module3_SPI1
Module3_SP8
Module3_SP4
Module3_SP3
Module3_SP2
Module3_SP1
Module3_SOX8
Module3_SOX6
Module3_SOX21
Module3_SOX2
Module3_SOX17
Module3_SOX13
Module3_SOX11
Module3_SOX10
Module3_SMAD4
Module3_SIX2
Module3_SIX1
Module3_SHOX2
Module3_SHOX
Module3_SCRT2
Module3_RXRG
Module3_RXRB
Module3_RXRA
Module3_RUNX3
Module3_RUNX2
Module3_RORC
Module3_RORB
Module3_RORA
Module3_RFX4
Module3_RFX3
Module3_RFX2
Module3_RFX1
Module3_REST
Module3_RELB
Module3_RELA
Module3_RBPJ
Module3_RAX
Module3_RARG
Module3_PRRX1
Module3_PROX1
Module3_PRDM1
Module3_PPARG
Module3_POU6F1
Module3_POU5F1
Module3_POU4F3
Module3_POU4F2
Module3_POU4F1
Module3_POU3F2
Module3_POU2F3
Module3_POU2F2
Module3_PKNOX2
Module3_PITX3
Module3_PHOX2A
Module3_PDX1
Module3_PBX2
Module3_PBX1
Module3_PAX7
Module3_PAX6
Module3_PAX5
Module3_PAX2
Module3_PAX1
Module3_ONECUT1
Module3_OLIG2
Module3_NRF1
Module3_NR5A2
Module3_NR4A2
Module3_NR4A1
Module3_NR3C2
Module3_NR3C1
Module3_NR2F6
Module3_NR2F2
Module3_NR2F1
Module3_NR2E3
Module3_NR2E1
Module3_NR2C2
Module3_NR1H4
Module3_NPAS2
Module3_NKX6-2

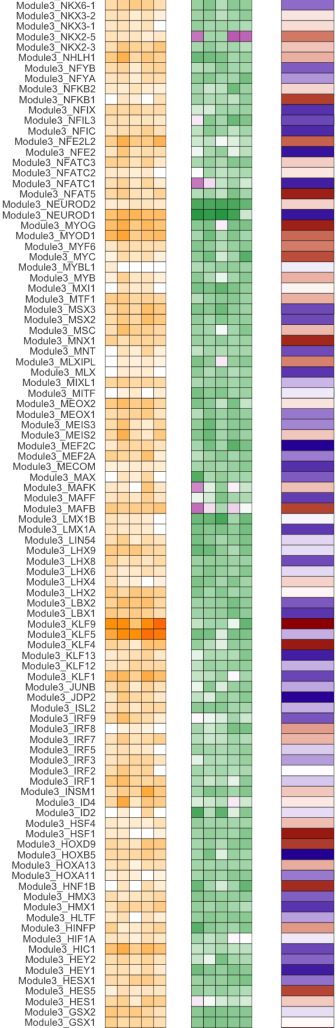

Module3_NKX6-1
Module3_NKX3-2
Module3_NKX3-1
Module3_NKX2-5
Module3_NKX2-3
Module3_NHLH1
Module3_NFYB
Module3_NFYA
Module3_NFKB2
Module3_NFKB1
Module3_NFIX
Module3_NFIL3
Module3_NFIC
Module3_NFE2L2
Module3_NFE2
Module3_NFATC3
Module3_NFATC2
Module3_NFATC1
Module3_NFAT5
Module3_NEUROD2
Module3_NEUROD1
Module3_MYOG
Module3_MYOD1
Module3_MYF6
Module3_MYC
Module3_MYBL1
Module3_MYB
Module3_MXI1
Module3_MTF1
Module3_MSX3
Module3_MSX2
Module3_MSC
Module3_MNX1
Module3_MNT
Module3_MLXIPL
Module3_MLX
Module3_MIXL1
Module3_MITF
Module3_MEOX2
Module3_MEOX1
Module3_MEIS3
Module3_MEIS2
Module3_MEF2C
Module3_MEF2A
Module3_MECOM
Module3_MAX
Module3_MAFK
Module3_MAFF
Module3_MAFB
Module3_LMX1B
Module3_LMX1A
Module3_LIN54
Module3_LHX9
Module3_LHX8
Module3_LHX6
Module3_LHX4
Module3_LHX2
Module3_LBX2
Module3_LBX1
Module3_KLF9
Module3_KLF5
Module3_KLF4
Module3_KLF13
Module3_KLF12
Module3_KLF1
Module3_JUNB
Module3_JDP2
Module3_ISL2
Module3_IRF9
Module3_IRF8
Module3_IRF7
Module3_IRF5
Module3_IRF3
Module3_IRF2
Module3_IRF1
Module3_INSM1
Module3_ID4
Module3_ID2
Module3_HSF4
Module3_HSF1
Module3_HOXD9
Module3_HOXB5
Module3_HOXA13
Module3_HOXA11
Module3_HNF1B
Module3_HMX3
Module3_HMX1
Module3_HLTF
Module3_HINFP
Module3_HIF1A
Module3_HIC1
Module3_HEY2
Module3_HEY1
Module3_HESX1
Module3_HES5
Module3_HES1
Module3_GSX2
Module3_GSX1

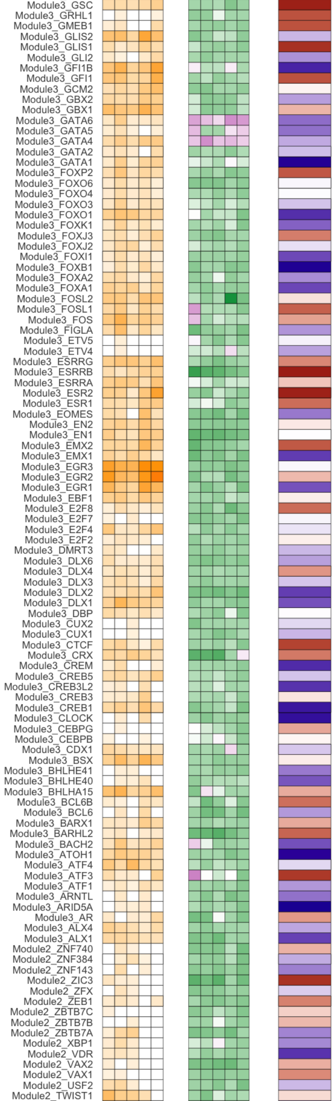

Module3_GSC
Module3_GRHL1
Module3_GMEB1
Module3_GLIS2
Module3_GLIS1
Module3_GLI2
Module3_GFI1B
Module3_GFI1
Module3_GCM2
Module3_GBX2
Module3_GBX1
Module3_GATA6
Module3_GATA5
Module3_GATA4
Module3_GATA2
Module3_GATA1
Module3_FOXP2
Module3_FOXO6
Module3_FOXO4
Module3_FOXO3
Module3_FOXO1
Module3_FOXK1
Module3_FOXJ3
Module3_FOXJ2
Module3_FOXI1
Module3_FOXB1
Module3_FOXA2
Module3_FOXA1
Module3_FOSL2
Module3_FOSL1
Module3_FOS
Module3_FIGLA
Module3_ETV5
Module3_ETV4
Module3_ESRRG
Module3_ESRRB
Module3_ESRRA
Module3_ESR2
Module3_ESR1
Module3_EOMES
Module3_EN2
Module3_EN1
Module3_EMX2
Module3_EMX1
Module3_EGR3
Module3_EGR2
Module3_EGR1
Module3_EBF1
Module3_E2F8
Module3_E2F7
Module3_E2F4
Module3_E2F2
Module3_DMRT3
Module3_DLX6
Module3_DLX4
Module3_DLX3
Module3_DLX2
Module3_DLX1
Module3_DBP
Module3_CUX2
Module3_CUX1
Module3_CTCF
Module3_CRX
Module3_CREM
Module3_CREB5
Module3_CREB3L2
Module3_CREB3
Module3_CREB1
Module3_CLOCK
Module3_CEBPG
Module3_CEBPB
Module3_CDX1
Module3_BSX
Module3_BHLHE41
Module3_BHLHE40
Module3_BHLHA15
Module3_BCL6B
Module3_BCL6
Module3_BARX1
Module3_BARHL2
Module3_BACH2
Module3_ATOH1
Module3_ATF4
Module3_ATF3
Module3_ATF1
Module3_ARNTL
Module3_ARID5A
Module3_AR
Module3_ALX4
Module3_ALX1
Module2_ZNF740
Module2_ZNF384
Module2_ZNF143
Module2_ZIC3
Module2_ZFX
Module2_ZEB1
Module2_ZBTB7C
Module2_ZBTB7B
Module2_ZBTB7A
Module2_XBP1
Module2_VDR
Module2_VAX2
Module2_VAX1
Module2_USF2
Module2_TWIST1

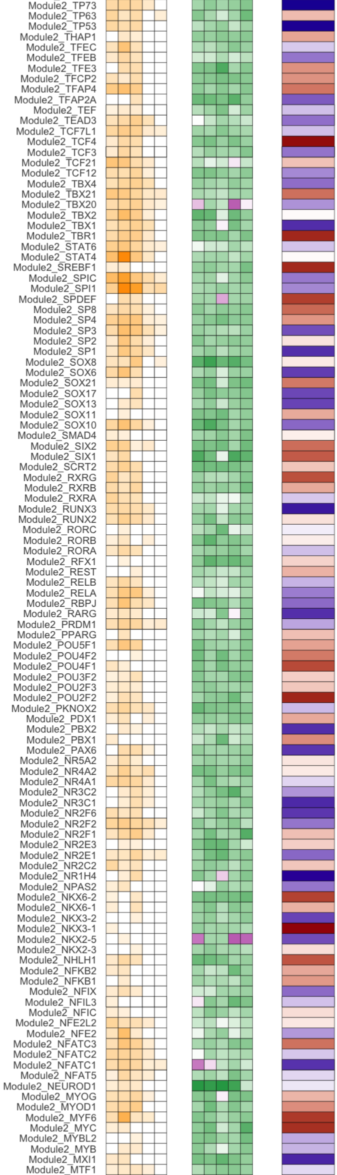

Module2_TP73
Module2_TP63
Module2_TP53
Module2_THAP1
Module2_TFEC
Module2_TFEB
Module2_TFE3
Module2_TFCP2
Module2_TFAP4
Module2_TFAP2A
Module2_TEF
Module2_TEAD3
Module2_TCF7L1
Module2_TCF4
Module2_TCF3
Module2_TCF21
Module2_TCF12
Module2_TBX4
Module2_TBX21
Module2_TBX20
Module2_TBX2
Module2_TBX1
Module2_TBR1
Module2_STAT6
Module2_STAT4
Module2_SREBF1
Module2_SPIC
Module2_SPI1
Module2_SPDEF
Module2_SP8
Module2_SP4
Module2_SP3
Module2_SP2
Module2_SP1
Module2_SOX8
Module2_SOX6
Module2_SOX21
Module2_SOX17
Module2_SOX13
Module2_SOX11
Module2_SOX10
Module2_SMAD4
Module2_SIX2
Module2_SIX1
Module2_SCRT2
Module2_RXRG
Module2_RXRB
Module2_RXRA
Module2_RUNX3
Module2_RUNX2
Module2_RORC
Module2_RORB
Module2_RORA
Module2_RFX1
Module2_REST
Module2_RELB
Module2_RELA
Module2_RBPJ
Module2_RARG
Module2_PRDM1
Module2_PPARG
Module2_POU5F1
Module2_POU4F2
Module2_POU4F1
Module2_POU3F2
Module2_POU2F3
Module2_POU2F2
Module2_PKNOX2
Module2_PDX1
Module2_PBX2
Module2_PBX1
Module2_PAX6
Module2_NR5A2
Module2_NR4A2
Module2_NR4A1
Module2_NR3C2
Module2_NR3C1
Module2_NR2F6
Module2_NR2F2
Module2_NR2F1
Module2_NR2E3
Module2_NR2E1
Module2_NR2C2
Module2_NR1H4
Module2_NPAS2
Module2_NKX6-2
Module2_NKX6-1
Module2_NKX3-2
Module2_NKX3-1
Module2_NKX2-5
Module2_NKX2-3
Module2_NHLH1
Module2_NFKB2
Module2_NFKB1
Module2_NFIX
Module2_NFIL3
Module2_NFIC
Module2_NFE2L2
Module2_NFE2
Module2_NFATC3
Module2_NFATC2
Module2_NFATC1
Module2_NFAT5
Module2_NEUROD1
Module2_MYOG
Module2_MYOD1
Module2_MYF6
Module2_MYC
Module2_MYBL2
Module2_MYB
Module2_MXI1
Module2_MTF1

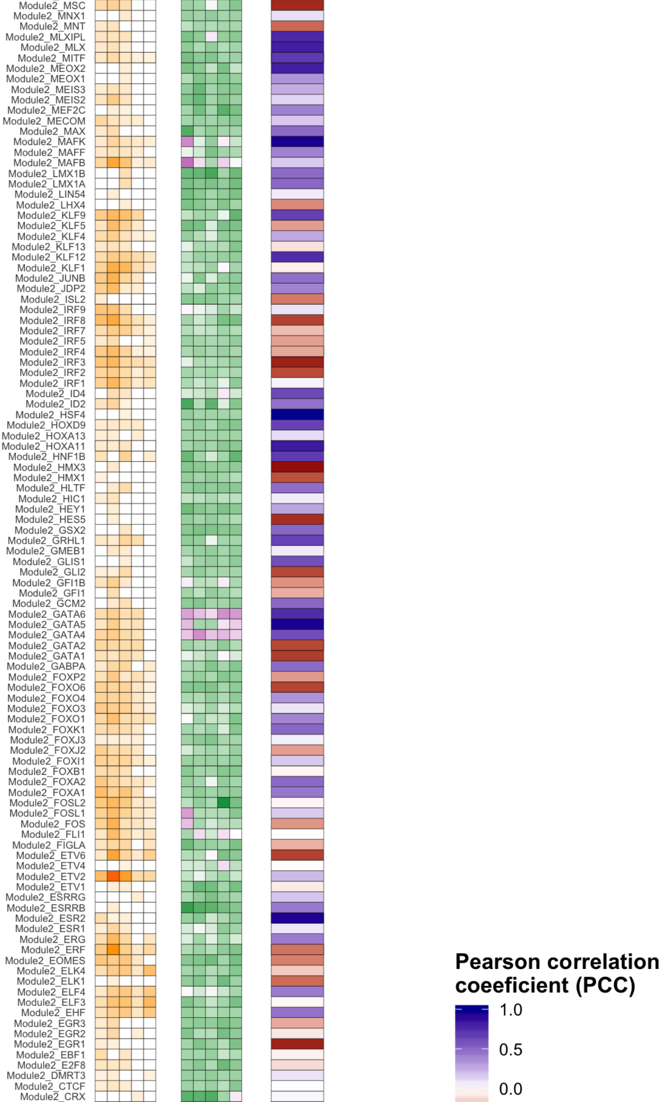
Module2_MSC
Module2_MNX1
Module2_MNT
Module2_MLXIPL
Module2_MLX
Module2_MITF
Module2_MEOX2
Module2_MEOX1
Module2_MEIS3
Module2_MEIS2
Module2_MEF2C
Module2_MECOM
Module2_MAX
Module2_MAFK
Module2_MAFF
Module2_MAFB
Module2_LMX1B
Module2_LMX1A
Module2_LIN54
Module2_LHX4
Module2_KLF9
Module2_KLF5
Module2_KLF4
Module2_KLF13
Module2_KLF12
Module2_KLF1
Module2_JUNB
Module2_JDP2
Module2_ISL2
Module2_IRF9
Module2_IRF8
Module2_IRF7
Module2_IRF5
Module2_IRF4
Module2_IRF3
Module2_IRF2
Module2_IRF1
Module2_ID4
Module2_ID2
Module2_HSF4
Module2_HOXD9
Module2_HOXA13
Module2_HOXA11
Module2_HNF1B
Module2_HMX3
Module2_HMX1
Module2_HLTF
Module2_HIC1
Module2_HEY1
Module2_HES5
Module2_GSX2
Module2_GRHL1
Module2_GMEB1
Module2_GLIS1
Module2_GLI2
Module2_GFI1B
Module2_GFI1
Module2_GCM2
Module2_GATA6
Module2_GATA5
Module2_GATA4
Module2_GATA2
Module2_GATA1
Module2_GABPA
Module2_FOXP2
Module2_FOXO6
Module2_FOXO4
Module2_FOXO3
Module2_FOXO1
Module2_FOXK1
Module2_FOXJ3
Module2_FOXJ2
Module2_FOXI1
Module2_FOXB1
Module2_FOXA2
Module2_FOXA1
Module2_FOSL2
Module2_FOSL1
Module2_FOS
Module2_FLI1
Module2_FIGLA
Module2_ETV6
Module2_ETV4
Module2_ETV2
Module2_ETV1
Module2_ESRRG
Module2_ESRRB
Module2_ESR2
Module2_ESR1
Module2_ERG
Module2_ERF
Module2_EOMES
Module2_ELK4
Module2_ELK1
Module2_ELF4
Module2_ELF3
Module2_EHF
Module2_EGR3
Module2_EGR2
Module2_EGR1
Module2_EBF1
Module2_E2F8
Module2_DMRT3
Module2_CTCF
Module2_CRX
Pearson correlation coeeficient (PCC)
1.0
0.5
0.0

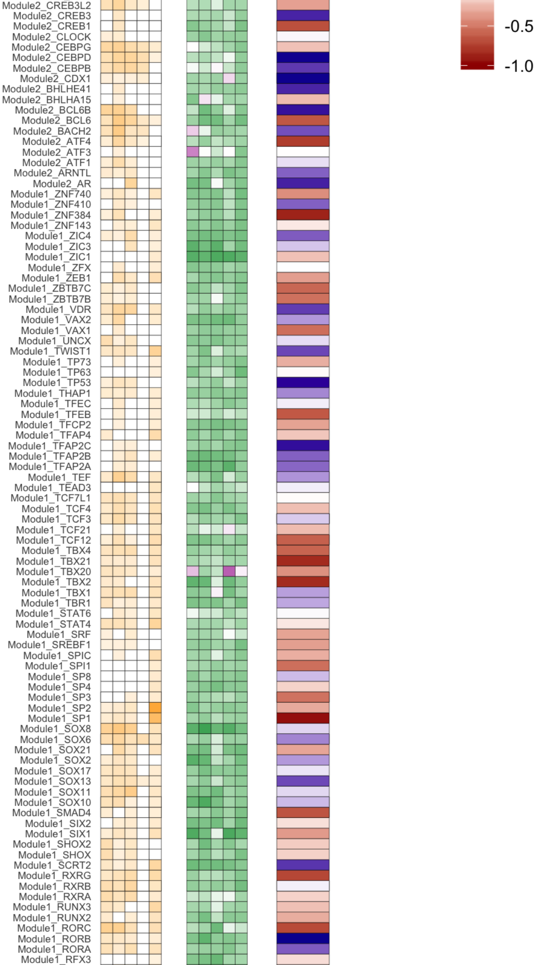

Module2_CREB3L2
Module2_CREB3
Module2_CREB1
Module2_CLOCK
Module2_CEBPG
Module2_CEBPD
Module2_CEBPB
Module2_CDX1
Module2_BHLHE41
Module2_BHLHA15
Module2_BCL6B
Module2_BCL6
Module2_BACH2
Module2_ATF4
Module2_ATF3
Module2_ATF1
Module2_ARNTL
Module2_AR
Module1_ZNF740
Module1_ZNF410
Module1_ZNF384
Module1_ZNF143
Module1_ZIC4
Module1_ZIC3
Module1_ZIC1
Module1_ZFX
Module1_ZEB1
Module1_ZBTB7C
Module1_ZBTB7B
Module1_VDR
Module1_VAX2
Module1_VAX1
Module1_UNCX
Module1_TWIST1
Module1_TP73
Module1_TP63
Module1_TP53
Module1_THAP1
Module1_TFEC
Module1_TFEB
Module1_TFCP2
Module1_TFAP4
Module1_TFAP2C
Module1_TFAP2B
Module1_TFAP2A
Module1_TEF
Module1_TEAD3
Module1_TCF7L1
Module1_TCF4
Module1_TCF3
Module1_TCF21
Module1_TCF12
Module1_TBX4
Module1_TBX21
Module1_TBX20
Module1_TBX2
Module1_TBX1
Module1_TBR1
Module1_STAT6
Module1_STAT4
Module1_SRF
Module1_SREBF1
Module1_SPIC
Module1_SPI1
Module1_SP8
Module1_SP4
Module1_SP3
Module1_SP2
Module1_SP1
Module1_SOX8
Module1_SOX6
Module1_SOX21
Module1_SOX2
Module1_SOX17
Module1_SOX13
Module1_SOX11
Module1_SOX10
Module1_SMAD4
Module1_SIX2
Module1_SIX1
Module1_SHOX2
Module1_SHOX
Module1_SCRT2
Module1_RXRG
Module1_RXRB
Module1_RXRA
Module1_RUNX3
Module1_RUNX2
Module1_RORC
Module1_RORB
Module1_RORA
Module1_RFX3
-0.5
-1.0

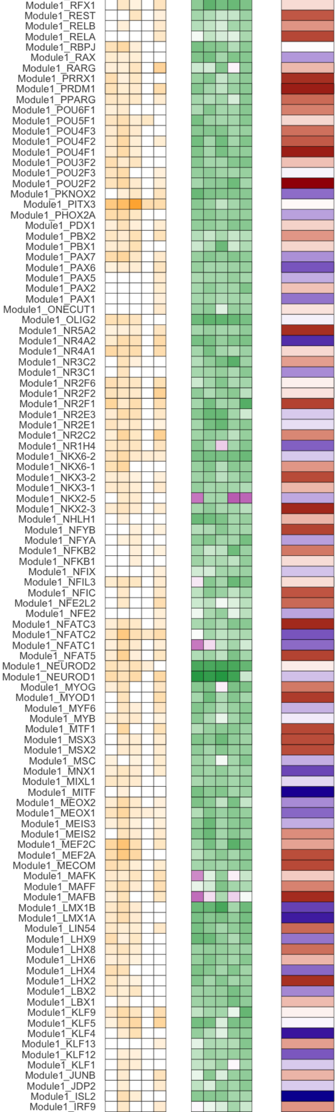
Module1_RFX1
Module1_REST
Module1_RELB
Module1_RELA
Module1_RBPJ
Module1_RAX
Module1_RARG
Module1_PRRX1
Module1_PRDM1
Module1_PPARG
Module1_POU6F1
Module1_POU5F1
Module1_POU4F3
Module1_POU4F2
Module1_POU4F1
Module1_POU3F2
Module1_POU2F3
Module1_POU2F2
Module1_PKNOX2
Module1_PITX3
Module1_PHOX2A
Module1_PDX1
Module1_PBX2
Module1_PBX1
Module1_PAX7
Module1_PAX6
Module1_PAX5
Module1_PAX2
Module1_PAX1
Module1_ONECUT1
Module1_OLIG2
Module1_NR5A2
Module1_NR4A2
Module1_NR4A1
Module1_NR3C2
Module1_NR3C1
Module1_NR2F6
Module1_NR2F2
Module1_NR2F1
Module1_NR2E3
Module1_NR2E1
Module1_NR2C2
Module1_NR1H4
Module1_NKX6-2
Module1_NKX6-1
Module1_NKX3-2
Module1_NKX3-1
Module1_NKX2-5
Module1_NKX2-3
Module1_NHLH1
Module1_NFYB
Module1_NFYA
Module1_NFKB2
Module1_NFKB1
Module1_NFIX
Module1_NFIL3
Module1_NFIC
Module1_NFE2L2
Module1_NFE2
Module1_NFATC3
Module1_NFATC2
Module1_NFATC1
Module1_NFAT5
Module1_NEUROD2
Module1_NEUROD1
Module1_MYOG
Module1_MYOD1
Module1_MYF6
Module1_MYB
Module1_MTF1
Module1_MSX3
Module1_MSX2
Module1_MSC
Module1_MNX1
Module1_MIXL1
Module1_MITF
Module1_MEOX2
Module1_MEOX1
Module1_MEIS3
Module1_MEIS2
Module1_MEF2C
Module1_MEF2A
Module1_MECOM
Module1_MAFK
Module1_MAFF
Module1_MAFB
Module1_LMX1B
Module1_LMX1A
Module1_LIN54
Module1_LHX9
Module1_LHX8
Module1_LHX6
Module1_LHX4
Module1_LHX2
Module1_LBX2
Module1_LBX1
Module1_KLF9
Module1_KLF5
Module1_KLF4
Module1_KLF13
Module1_KLF12
Module1_KLF1
Module1_JUNB
Module1_JDP2
Module1_ISL2
Module1_IRF9

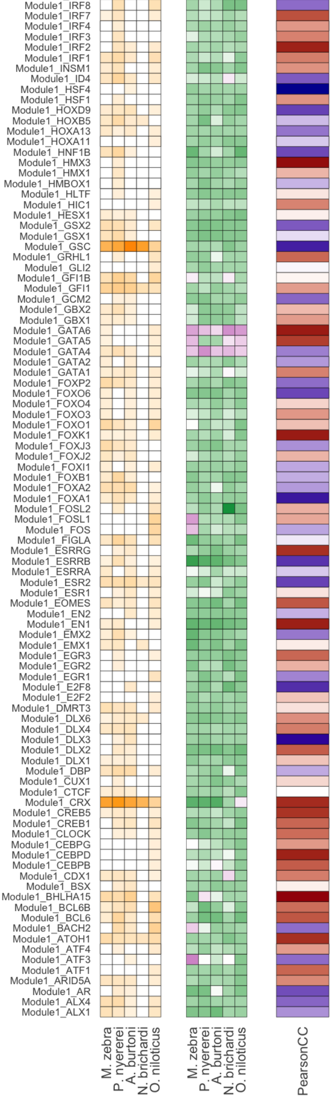
Module1_IRF8
Module1_IRF7
Module1_IRF4
Module1_IRF3
Module1_IRF2
Module1_IRF1
Module1_INSM1
Module1_ID4
Module1_HSF4
Module1_HSF1
Module1_HOXD9
Module1_HOXB5
Module1_HOXA13
Module1_HOXA11
Module1_HNF1B
Module1_HMX3
Module1_HMX1
Module1_HMBOX1
Module1_HLTF
Module1_HIC1
Module1_HESX1
Module1_GSX2
Module1_GSX1
Module1_GSC
Module1_GRHL1
Module1_GLI2
Module1_GFI1B
Module1_GFI1
Module1_GCM2
Module1_GBX2
Module1_GBX1
Module1_GATA6
Module1_GATA5
Module1_GATA4
Module1_GATA2
Module1_GATA1
Module1_FOXP2
Module1_FOXO6
Module1_FOXO4
Module1_FOXO3
Module1_FOXO1
Module1_FOXK1
Module1_FOXJ3
Module1_FOXJ2
Module1_FOXI1
Module1_FOXB1
Module1_FOXA2
Module1_FOXA1
Module1_FOSL2
Module1_FOSL1
Module1_FOS
Module1_FIGLA
Module1_ESRRG
Module1_ESRRB
Module1_ESRRA
Module1_ESR2
Module1_ESR1
Module1_EOMES
Module1_EN2
Module1_EN1
Module1_EMX2
Module1_EMX1
Module1_EGR3
Module1_EGR2
Module1_EGR1
Module1_E2F8
Module1_E2F2
Module1_DMRT3
Module1_DLX6
Module1_DLX4
Module1_DLX3
Module1_DLX2
Module1_DLX1
Module1_DBP
Module1_CUX1
Module1_CTCF
Module1_CRX
Module1_CREB5
Module1_CREB1
Module1_CLOCK
Module1_CEBPG
Module1_CEBPD
Module1_CEBPB
Module1_CDX1
Module1_BSX
Module1_BHLHA15
Module1_BCL6B
Module1_BCL6
Module1_BACH2
Module1_ATOH1
Module1_ATF4
Module1_ATF3
Module1_ATF1
Module1_ARID5A
Module1_AR
Module1_ALX4
Module1_ALX1
M. zebra
P. nyererei
A. burtoni
N. brichardi
O. niloticus
M. zebra
P. nyererei
A. burtoni
N. brichardi
O. niloticus
PearsonCC

Additional file 2: Fig. S6

Heatmap matrices of enrichment, expression and Pearson correlation between the two for each motif, across kidney tissue

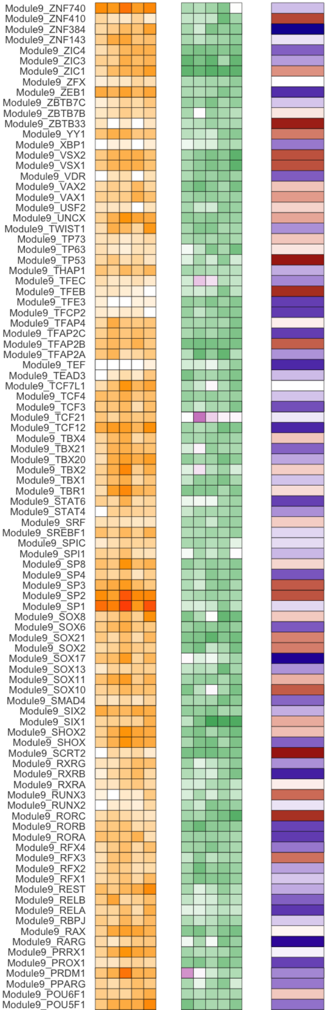

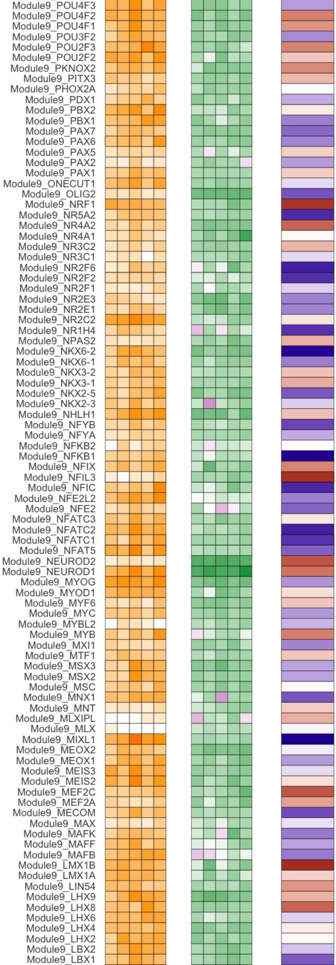
Module9_POU4F3
Module9_POU4F2
Module9_POU4F1
Module9_POU3F2
Module9_POU2F3
Module9_POU2F2
Module9_PKNOX2
Module9_PITX3
Module9_PHOX2A
Module9_PDX1
Module9_PBX2
Module9_PBX1
Module9_PAX7
Module9_PAX6
Module9_PAX5
Module9_PAX2
Module9_PAX1
Module9_ONECUT1
Module9_OLIG2
Module9_NRF1
Module9_NR5A2
Module9_NR4A2
Module9_NR4A1
Module9_NR3C2
Module9_NR3C1
Module9_NR2F6
Module9_NR2F2
Module9_NR2F1
Module9_NR2E3
Module9_NR2E1
Module9_NR2C2
Module9_NR1H4
Module9_NPAS2
Module9_NKX6-2
Module9_NKX6-1
Module9_NKX3-2
Module9_NKX3-1
Module9_NKX2-5
Module9_NKX2-3
Module9_NHLH1
Module9_NFYB
Module9_NFYA
Module9_NFKB2
Module9_NFKB1
Module9_NFIX
Module9_NFIL3
Module9_NFIC
Module9_NFE2L2
Module9_NFE2
Module9_NFATC3
Module9_NFATC2
Module9_NFATC1
Module9_NFAT5
Module9_NEUROD2
Module9_NEUROD1
Module9_MYOG
Module9_MYOD1
Module9_MYF6
Module9_MYC
Module9_MYBL2
Module9_MYB
Module9_MXI1
Module9_MTF1
Module9_MSX3
Module9_MSX2
Module9_MSC
Module9_MNX1
Module9_MNT
Module9_MLXIPL
Module9_MLX
Module9_MIXL1
Module9_MEOX2
Module9_MEOX1
Module9_MEIS3
Module9_MEIS2
Module9_MEF2C
Module9_MEF2A
Module9_MECOM
Module9_MAX
Module9_MAFK
Module9_MAFF
Module9_MAFB
Module9_LMX1B
Module9_LMX1A
Module9_LIN54
Module9_LHX9
Module9_LHX8
Module9_LHX6
Module9_LHX4
Module9_LHX2
Module9_LBX2
Module9_LBX1

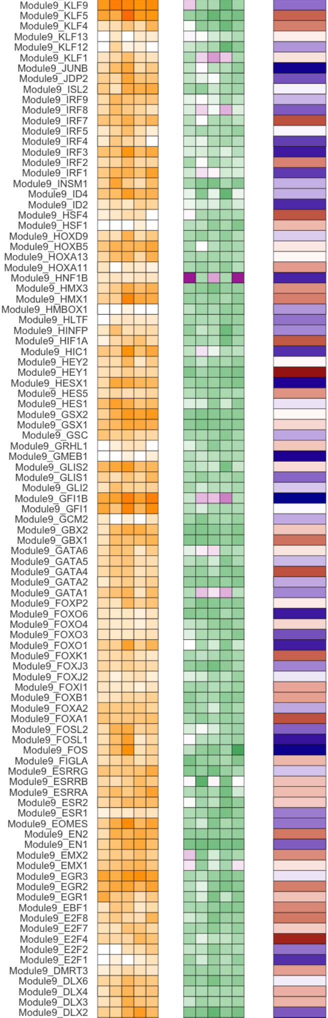

Module9_KLF9
Module9_KLF5
Module9_KLF4
Module9_KLF13
Module9_KLF12
Module9_KLF1
Module9_JUNB
Module9_JDP2
Module9_ISL2
Module9_IRF9
Module9_IRF8
Module9_IRF7
Module9_IRF5
Module9_IRF4
Module9_IRF3
Module9_IRF2
Module9_IRF1
Module9_INSM1
Module9_ID4
Module9_ID2
Module9_HSF4
Module9_HSF1
Module9_HOXD9
Module9_HOXB5
Module9_HOXA13
Module9_HOXA11
Module9_HNF1B
Module9_HMX3
Module9_HMX1
Module9_HMBOX1
Module9_HLTF
Module9_HINFP
Module9_HIF1A
Module9_HIC1
Module9_HEY2
Module9_HEY1
Module9_HESX1
Module9_HES5
Module9_HES1
Module9_GSX2
Module9_GSX1
Module9_GSC
Module9_GRHL1
Module9_GMEB1
Module9_GLIS2
Module9_GLIS1
Module9_GLI2
Module9_GFI1B
Module9_GFI1
Module9_GCM2
Module9_GBX2
Module9_GBX1
Module9_GATA6
Module9_GATA5
Module9_GATA4
Module9_GATA2
Module9_GATA1
Module9_FOXP2
Module9_FOXO6
Module9_FOXO4
Module9_FOXO3
Module9_FOXO1
Module9_FOXK1
Module9_FOXJ3
Module9_FOXJ2
Module9_FOXI1
Module9_FOXB1
Module9_FOXA2
Module9_FOXA1
Module9_FOSL2
Module9_FOSL1
Module9_FOS
Module9_FIGLA
Module9_ESRRG
Module9_ESRRB
Module9_ESRRA
Module9_ESR2
Module9_ESR1
Module9_EOMES
Module9_EN2
Module9_EN1
Module9_EMX2
Module9_EMX1
Module9_EGR3
Module9_EGR2
Module9_EGR1
Module9_EBF1
Module9_E2F8
Module9_E2F7
Module9_E2F4
Module9_E2F2
Module9_E2F1
Module9_DMRT3
Module9_DLX6
Module9_DLX4
Module9_DLX3
Module9_DLX2

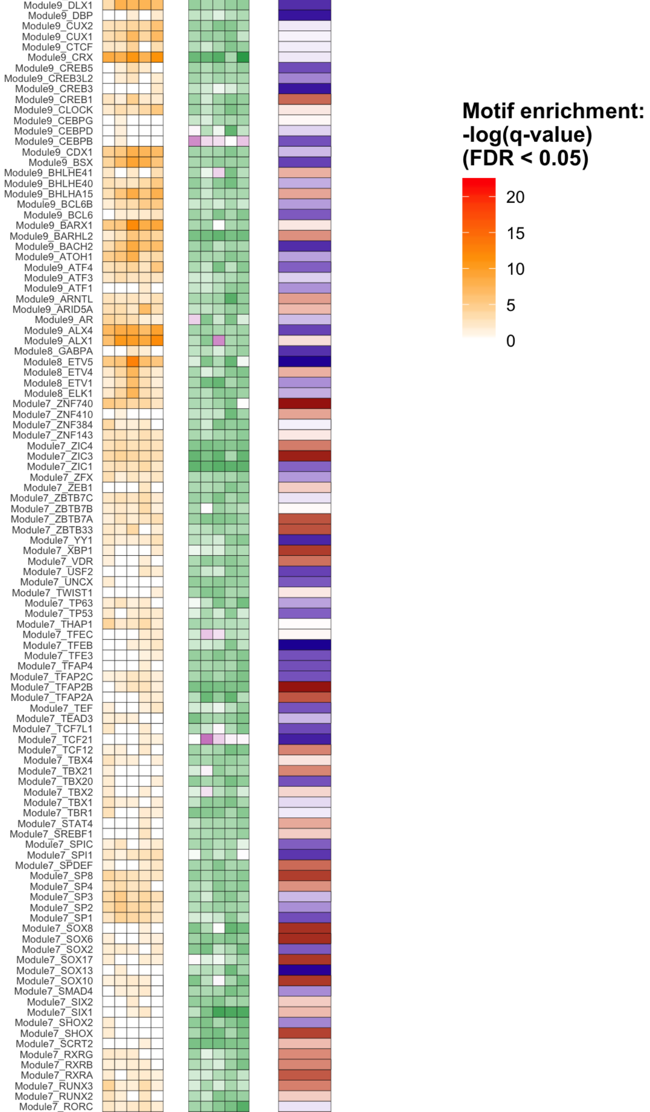
Module9_DLX1
Module9_DBP
Module9_CUX2
Module9_CUX1
Module9_CTCF
Module9_CRX
Module9_CREB5
Module9_CREB3L2
Module9_CREB3
Module9_CREB1
Module9_CLOCK
Module9_CEBPG
Module9_CEBPD
Module9_CEBPB
Module9_CDX1
Module9_BSX
Module9_BHLHE41
Module9_BHLHE40
Module9_BHLHA15
Module9_BCL6B
Module9_BCL6
Module9_BARX1
Module9_BARHL2
Module9_BACH2
Module9_ATOH1
Module9_ATF4
Module9_ATF3
Module9_ATF1
Module9_ARNTL
Module9_ARID5A
Module9_AR
Module9_ALX4
Module9_ALX1
Module8_GABPA
Module8_ETV5
Module8_ETV4
Module8_ETV1
Module8_ELK1
Module7_ZNF740
Module7_ZNF410
Module7_ZNF384
Module7_ZNF143
Module7_ZIC4
Module7_ZIC3
Module7_ZIC1
Module7_ZFX
Module7_ZEB1
Module7_ZBTB7C
Module7_ZBTB7B
Module7_ZBTB7A
Module7_ZBTB33
Module7_YY1
Module7_XBP1
Module7_VDR
Module7_USF2
Module7_UNCX
Module7_TWIST1
Module7_TP63
Module7_TP53
Module7_THAP1
Module7_TFEC
Module7_TFEB
Module7_TFE3
Module7_TFAP4
Module7_TFAP2C
Module7_TFAP2B
Module7_TFAP2A
Module7_TEF
Module7_TEAD3
Module7_TCF7L1
Module7_TCF21
Module7_TCF12
Module7_TBX4
Module7_TBX21
Module7_TBX20
Module7_TBX2
Module7_TBX1
Module7_TBR1
Module7_STAT4
Module7_SREBF1
Module7_SPIC
Module7_SPI1
Module7_SPDEF
Module7_SP8
Module7_SP4
Module7_SP3
Module7_SP2
Module7_SP1
Module7_SOX8
Module7_SOX6
Module7_SOX2
Module7_SOX17
Module7_SOX13
Module7_SOX10
Module7_SMAD4
Module7_SIX2
Module7_SIX1
Module7_SHOX2
Module7_SHOX
Module7_SCRT2
Module7_RXRG
Module7_RXRB
Module7_RXRA
Module7_RUNX3
Module7_RUNX2
Module7_RORC
Motif enrichment:
-log(q-value)
(FDR < 0.05)
20
15
10
5
0

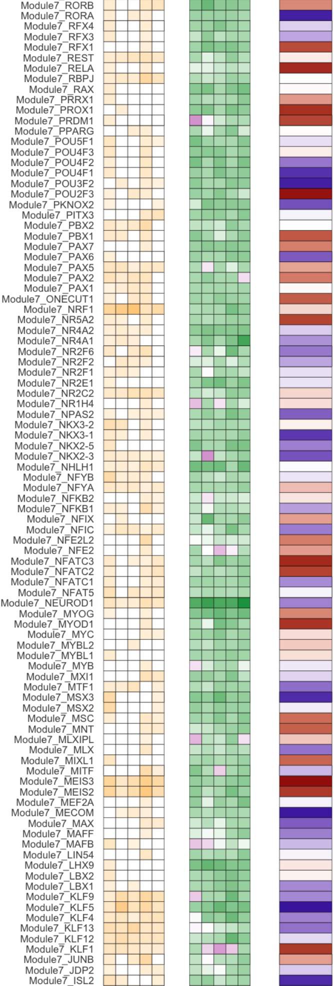
Module7_RORB
Module7_RORA
Module7_RFX4
Module7_RFX3
Module7_RFX1
Module7_REST
Module7_RELA
Module7_RBPJ
Module7_RAX
Module7_PRRX1
Module7_PROX1
Module7_PRDM1
Module7_PPARG
Module7_POU5F1
Module7_POU4F3
Module7_POU4F2
Module7_POU4F1
Module7_POU3F2
Module7_POU2F3
Module7_PKNOX2
Module7_PITX3
Module7_PBX2
Module7_PBX1
Module7_PAX7
Module7_PAX6
Module7_PAX5
Module7_PAX2
Module7_PAX1
Module7_ONECUT1
Module7_NRF1
Module7_NR5A2
Module7_NR4A2
Module7_NR4A1
Module7_NR2F6
Module7_NR2F2
Module7_NR2F1
Module7_NR2E1
Module7_NR2C2
Module7_NR1H4
Module7_NPAS2
Module7_NKX3-2
Module7_NKX3-1
Module7_NKX2-5
Module7_NKX2-3
Module7_NHLH1
Module7_NFYB
Module7_NFYA
Module7_NFKB2
Module7_NFKB1
Module7_NFIX
Module7_NFIC
Module7_NFE2L2
Module7_NFE2
Module7_NFATC3
Module7_NFATC2
Module7_NFATC1
Module7_NFAT5
Module7_NEUROD1
Module7_MYOG
Module7_MYOD1
Module7_MYC
Module7_MYBL2
Module7_MYBL1
Module7_MYB
Module7_MXI1
Module7_MTF1
Module7_MSX3
Module7_MSX2
Module7_MSC
Module7_MNT
Module7_MLXIPL
Module7_MLX
Module7_MIXL1
Module7_MITF
Module7_MEIS3
Module7_MEIS2
Module7_MEF2A
Module7_MECOM
Module7_MAX
Module7_MAFF
Module7_MAFB
Module7_LIN54
Module7_LHX9
Module7_LBX2
Module7_LBX1
Module7_KLF9
Module7_KLF5
Module7_KLF4
Module7_KLF13
Module7_KLF12
Module7_KLF1
Module7_JUNB
Module7_JDP2
Module7_ISL2

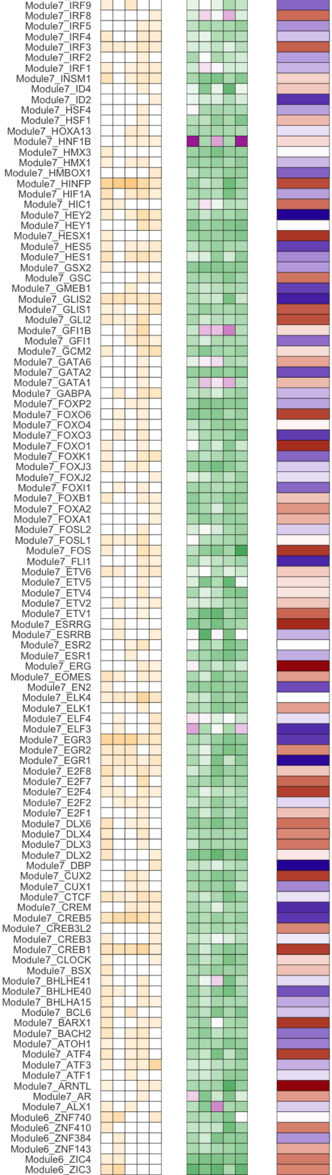
Module7_IRF9
Module7_IRF8
Module7_IRF5
Module7_IRF4
Module7_IRF3
Module7_IRF2
Module7_IRF1
Module7_INSM1
Module7_ID4
Module7_ID2
Module7_HSF4
Module7_HSF1
Module7_HOXA13
Module7_HNF1B
Module7_HMX3
Module7_HMX1
Module7_HMBOX1
Module7_HINFP
Module7_HIF1A
Module7_HIC1
Module7_HEY2
Module7_HEY1
Module7_HESX1
Module7_HES5
Module7_HES1
Module7_GSX2
Module7_GSC
Module7_GMEB1
Module7_GLIS2
Module7_GLIS1
Module7_GLI2
Module7_GFI1B
Module7_GFI1
Module7_GCM2
Module7_GATA6
Module7_GATA2
Module7_GATA1
Module7_GABPA
Module7_FOXP2
Module7_FOXO6
Module7_FOXO4
Module7_FOXO3
Module7_FOXO1
Module7_FOXK1
Module7_FOXJ3
Module7_FOXJ2
Module7_FOXI1
Module7_FOXB1
Module7_FOXA2
Module7_FOXA1
Module7_FOSL2
Module7_FOSL1
Module7_FOS
Module7_FLI1
Module7_ETV6
Module7_ETV5
Module7_ETV4
Module7_ETV2
Module7_ETV1
Module7_ESRRG
Module7_ESRRB
Module7_ESR2
Module7_ESR1
Module7_ERG
Module7_EOMES
Module7_EN2
Module7_ELK4
Module7_ELK1
Module7_ELF4
Module7_ELF3
Module7_EGR3
Module7_EGR2
Module7_EGR1
Module7_E2F8
Module7_E2F7
Module7_E2F4
Module7_E2F2
Module7_E2F1
Module7_DLX6
Module7_DLX4
Module7_DLX3
Module7_DLX2
Module7_DBP
Module7_CUX2
Module7_CUX1
Module7_CTCF
Module7_CREM
Module7_CREB5
Module7_CREB3L2
Module7_CREB3
Module7_CREB1
Module7_CLOCK
Module7_BSX
Module7_BHLHE41
Module7_BHLHE40
Module7_BHLHA15
Module7_BCL6
Module7_BARX1
Module7_BACH2
Module7_ATOH1
Module7_ATF4
Module7_ATF3
Module7_ATF1
Module7_ARNTL
Module7_AR
Module7_ALX1
Module6_ZNF740
Module6_ZNF410
Module6_ZNF384
Module6_ZNF143
Module6_ZIC4
Module6_ZIC3

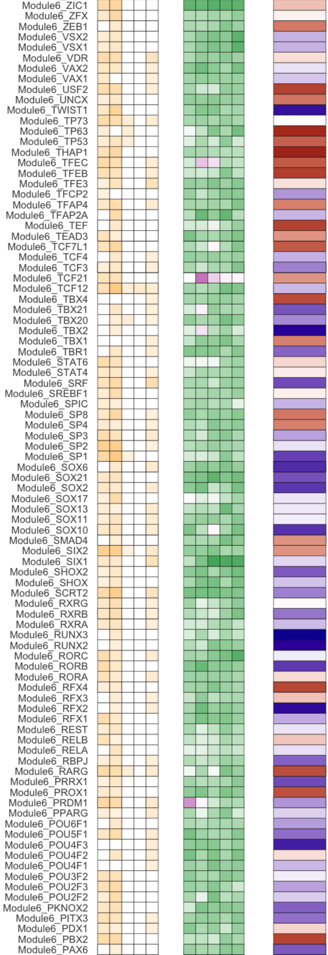
Module6_ZIC1
Module6_ZFX
Module6_ZEB1
Module6_VSX2
Module6_VSX1
Module6_VDR
Module6_VAX2
Module6_VAX1
Module6_USF2
Module6_UNCX
Module6_TWIST1
Module6_TP73
Module6_TP63
Module6_TP53
Module6_THAP1
Module6_TFEC
Module6_TFEB
Module6_TFE3
Module6_TFCP2
Module6_TFAP4
Module6_TFAP2A
Module6_TEF
Module6_TEAD3
Module6_TCF7L1
Module6_TCF4
Module6_TCF3
Module6_TCF21
Module6_TCF12
Module6_TBX4
Module6_TBX21
Module6_TBX20
Module6_TBX2
Module6_TBX1
Module6_TBR1
Module6_STAT6
Module6_STAT4
Module6_SRF
Module6_SREBF1
Module6_SPIC
Module6_SP8
Module6_SP4
Module6_SP3
Module6_SP2
Module6_SP1
Module6_SOX6
Module6_SOX21
Module6_SOX2
Module6_SOX17
Module6_SOX13
Module6_SOX11
Module6_SOX10
Module6_SMAD4
Module6_SIX2
Module6_SIX1
Module6_SHOX2
Module6_SHOX
Module6_SCRT2
Module6_RXRG
Module6_RXRB
Module6_RXRA
Module6_RUNX3
Module6_RUNX2
Module6_RORC
Module6_RORB
Module6_RORA
Module6_RFX4
Module6_RFX3
Module6_RFX2
Module6_RFX1
Module6_REST
Module6_RELB
Module6_RELA
Module6_RBPJ
Module6_RARG
Module6_PRRX1
Module6_PROX1
Module6_PRDM1
Module6_PPARG
Module6_POU6F1
Module6_POU5F1
Module6_POU4F3
Module6_POU4F2
Module6_POU4F1
Module6_POU3F2
Module6_POU2F3
Module6_POU2F2
Module6_PKNOX2
Module6_PITX3
Module6_PDX1
Module6_PBX2
Module6_PAX6

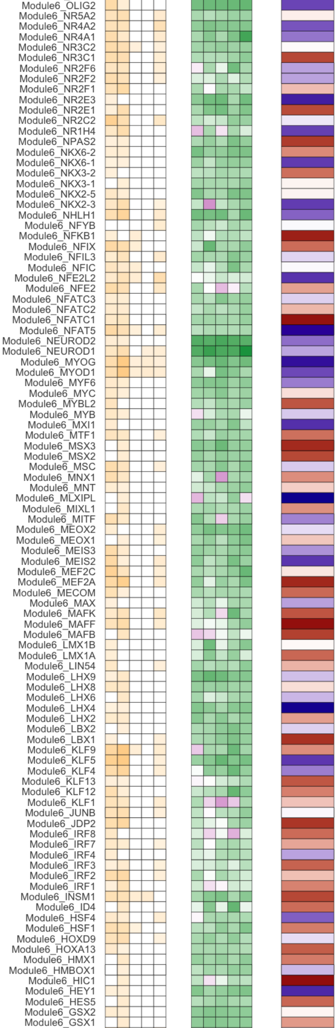
Module6_OLIG2
Module6_NR5A2
Module6_NR4A2
Module6_NR4A1
Module6_NR3C2
Module6_NR3C1
Module6_NR2F6
Module6_NR2F2
Module6_NR2F1
Module6_NR2E3
Module6_NR2E1
Module6_NR2C2
Module6_NR1H4
Module6_NPAS2
Module6_NKX6-2
Module6_NKX6-1
Module6_NKX3-2
Module6_NKX3-1
Module6_NKX2-5
Module6_NKX2-3
Module6_NHLH1
Module6_NFYB
Module6_NFKB1
Module6_NFIX
Module6_NFIL3
Module6_NFIC
Module6_NFE2L2
Module6_NFE2
Module6_NFATC3
Module6_NFATC2
Module6_NFATC1
Module6_NFAT5
Module6_NEUROD2
Module6_NEUROD1
Module6_MYOG
Module6_MYOD1
Module6_MYF6
Module6_MYC
Module6_MYBL2
Module6_MYB
Module6_MXI1
Module6_MTF1
Module6_MSX3
Module6_MSX2
Module6_MSC
Module6_MNX1
Module6_MNT
Module6_MLXIPL
Module6_MIXL1
Module6_MITF
Module6_MEOX2
Module6_MEOX1
Module6_MEIS3
Module6_MEIS2
Module6_MEF2C
Module6_MEF2A
Module6_MECOM
Module6_MAX
Module6_MAFK
Module6_MAFF
Module6_MAFB
Module6_LMX1B
Module6_LMX1A
Module6_LIN54
Module6_LHX9
Module6_LHX8
Module6_LHX6
Module6_LHX4
Module6_LHX2
Module6_LBX2
Module6_LBX1
Module6_KLF9
Module6_KLF5
Module6_KLF4
Module6_KLF13
Module6_KLF12
Module6_KLF1
Module6_JUNB
Module6_JDP2
Module6_IRF8
Module6_IRF7
Module6_IRF4
Module6_IRF3
Module6_IRF2
Module6_IRF1
Module6_INSM1
Module6_ID4
Module6_HSF4
Module6_HSF1
Module6_HOXD9
Module6_HOXA13
Module6_HMX1
Module6_HMBOX1
Module6_HIC1
Module6_HEY1
Module6_HES5
Module6_GSX2
Module6_GSX1

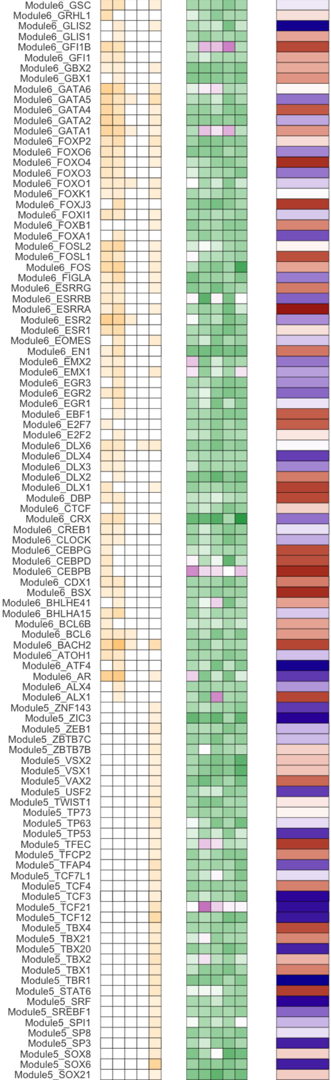
Module6_GSC
Module6_GRHL1
Module6_GLIS2
Module6_GLIS1
Module6_GFI1B
Module6_GFI1
Module6_GBX2
Module6_GBX1
Module6_GATA6
Module6_GATA5
Module6_GATA4
Module6_GATA2
Module6_GATA1
Module6_FOXP2
Module6_FOXO6
Module6_FOXO4
Module6_FOXO3
Module6_FOXO1
Module6_FOXK1
Module6_FOXJ3
Module6_FOXI1
Module6_FOXB1
Module6_FOXA1
Module6_FOSL2
Module6_FOSL1
Module6_FOS
Module6_FIGLA
Module6_ESRRG
Module6_ESRRB
Module6_ESRRA
Module6_ESR2
Module6_ESR1
Module6_EOMES
Module6_EN1
Module6_EMX2
Module6_EMX1
Module6_EGR3
Module6_EGR2
Module6_EGR1
Module6_EBF1
Module6_E2F7
Module6_E2F2
Module6_DLX6
Module6_DLX4
Module6_DLX3
Module6_DLX2
Module6_DLX1
Module6_DBP
Module6_CTCF
Module6_CRX
Module6_CREB1
Module6_CLOCK
Module6_CEBPG
Module6_CEBPD
Module6_CEBPB
Module6_CDX1
Module6_BSX
Module6_BHLHE41
Module6_BHLHA15
Module6_BCL6B
Module6_BCL6
Module6_BACH2
Module6_ATOH1
Module6_ATF4
Module6_AR
Module6_ALX4
Module6_ALX1
Module5_ZNF143
Module5_ZIC3
Module5_ZEB1
Module5_ZBTB7C
Module5_ZBTB7B
Module5_VSX2
Module5_VSX1
Module5_VAX2
Module5_USF2
Module5_TWIST1
Module5_TP73
Module5_TP63
Module5_TP53
Module5_TFEC
Module5_TFCP2
Module5_TFAP4
Module5_TCF7L1
Module5_TCF4
Module5_TCF3
Module5_TCF21
Module5_TCF12
Module5_TBX4
Module5_TBX21
Module5_TBX20
Module5_TBX2
Module5_TBX1
Module5_TBR1
Module5_STAT6
Module5_SRF
Module5_SREBF1
Module5_SPI1
Module5_SP8
Module5_SP3
Module5_SOX8
Module5_SOX6
Module5_SOX21

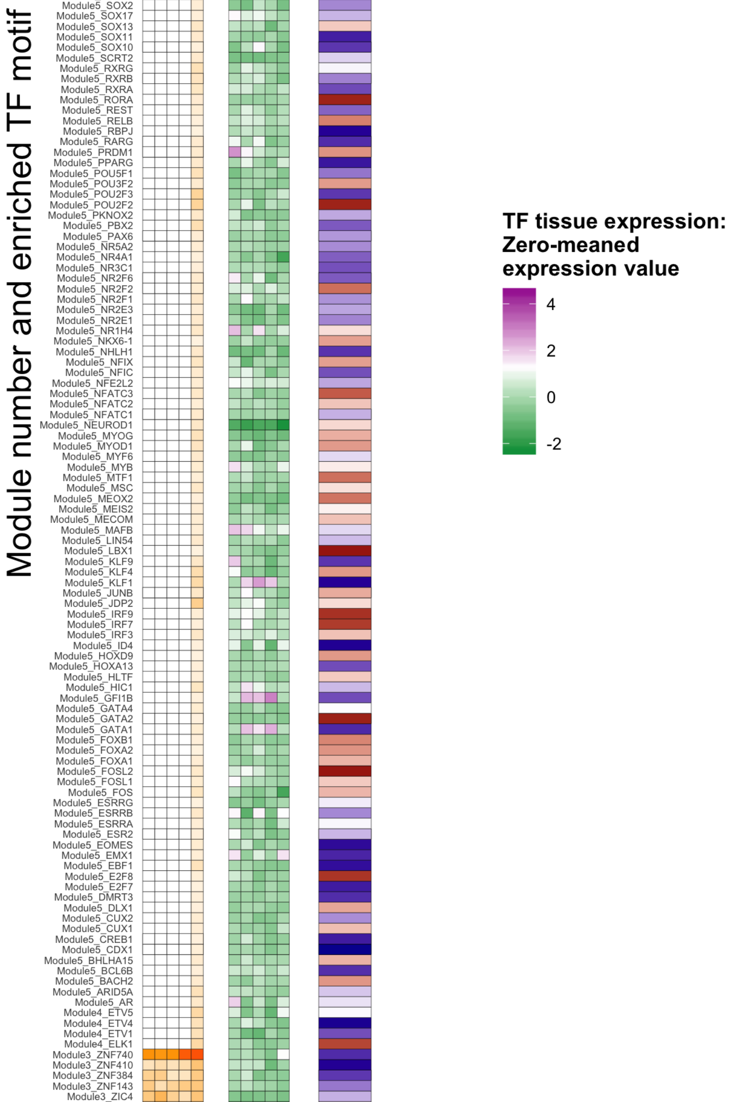
Module number and enriched TF motif
Module5_SOX2
Module5_SOX17
Module5_SOX13
Module5_SOX11
Module5_SOX10
Module5_SCRT2
Module5_RXRG
Module5_RXRB
Module5_RXRA
Module5_RORA
Module5_REST
Module5_RELB
Module5_RBPJ
Module5_RARG
Module5_PRDM1
Module5_PPARG
Module5_POU5F1
Module5_POU3F2
Module5_POU2F3
Module5_POU2F2
Module5_PKNOX2
Module5_PBX2
Module5_PAX6
Module5_NR5A2
Module5_NR4A1
Module5_NR3C1
Module5_NR2F6
Module5_NR2F2
Module5_NR2F1
Module5_NR2E3
Module5_NR2E1
Module5_NR1H4
Module5_NKX6-1
Module5_NHLH1
Module5_NFIX
Module5_NFIC
Module5_NFE2L2
Module5_NFATC3
Module5_NFATC2
Module5_NFATC1
Module5_NEUROD1
Module5_MYOG
Module5_MYOD1
Module5_MYF6
Module5_MYB
Module5_MTF1
Module5_MSC
Module5_MEOX2
Module5_MEIS2
Module5_MECOM
Module5_MAFB
Module5_LIN54
Module5_LBX1
Module5_KLF9
Module5_KLF4
Module5_KLF1
Module5_JUNB
Module5_JDP2
Module5_IRF9
Module5_IRF7
Module5_IRF3
Module5_ID4
Module5_HOXD9
Module5_HOXA13
Module5_HLTF
Module5_HIC1
Module5_GFI1B
Module5_GATA4
Module5_GATA2
Module5_GATA1
Module5_FOXB1
Module5_FOXA2
Module5_FOXA1
Module5_FOSL2
Module5_FOSL1
Module5_FOS
Module5_ESRRG
Module5_ESRRB
Module5_ESRRA
Module5_ESR2
Module5_EOMES
Module5_EMX1
Module5_EBF1
Module5_E2F8
Module5_E2F7
Module5_DMRT3
Module5_DLX1
Module5_CUX2
Module5_CUX1
Module5_CREB1
Module5_CDX1
Module5_BHLHA15
Module5_BCL6B
Module5_BACH2
Module5_ARID5A
Module5_AR
Module4_ETV5
Module4_ETV4
Module4_ETV1
Module4_ELK1
Module3_ZNF740
Module3_ZNF410
Module3_ZNF384
Module3_ZNF143
Module3_ZIC4
TF tissue expression: Zero-meaned expression value
4
2
0
-2

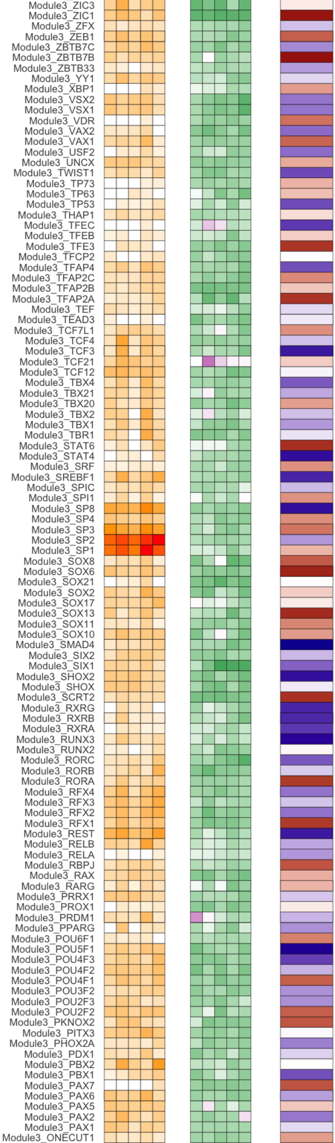
Module3_ZIC3
Module3_ZIC1
Module3_ZFX
Module3_ZEB1
Module3_ZBTB7C
Module3_ZBTB7B
Module3_ZBTB33
Module3_YY1
Module3_XBP1
Module3_VSX2
Module3_VSX1
Module3_VDR
Module3_VAX2
Module3_VAX1
Module3_USF2
Module3_UNCX
Module3_TWIST1
Module3_TP73
Module3_TP63
Module3_TP53
Module3_THAP1
Module3_TFEC
Module3_TFEB
Module3_TFE3
Module3_TFCP2
Module3_TFAP4
Module3_TFAP2C
Module3_TFAP2B
Module3_TFAP2A
Module3_TEF
Module3_TEAD3
Module3_TCF7L1
Module3_TCF4
Module3_TCF3
Module3_TCF21
Module3_TCF12
Module3_TBX4
Module3_TBX21
Module3_TBX20
Module3_TBX2
Module3_TBX1
Module3_TBR1
Module3_STAT6
Module3_STAT4
Module3_SRF
Module3_SREBF1
Module3_SPIC
Module3_SPI1
Module3_SP8
Module3_SP4
Module3_SP3
Module3_SP2
Module3_SP1
Module3_SOX8
Module3_SOX6
Module3_SOX21
Module3_SOX2
Module3_SOX17
Module3_SOX13
Module3_SOX11
Module3_SOX10
Module3_SMAD4
Module3_SIX2
Module3_SIX1
Module3_SHOX2
Module3_SHOX
Module3_SCRT2
Module3_RXRG
Module3_RXRB
Module3_RXRA
Module3_RUNX3
Module3_RUNX2
Module3_RORC
Module3_RORB
Module3_RORA
Module3_RFX4
Module3_RFX3
Module3_RFX2
Module3_RFX1
Module3_REST
Module3_RELB
Module3_RELA
Module3_RBPJ
Module3_RAX
Module3_RARG
Module3_PRRX1
Module3_PROX1
Module3_PRDM1
Module3_PPARG
Module3_POU6F1
Module3_POU5F1
Module3_POU4F3
Module3_POU4F2
Module3_POU4F1
Module3_POU3F2
Module3_POU2F3
Module3_POU2F2
Module3_PKNOX2
Module3_PITX3
Module3_PHOX2A
Module3_PDX1
Module3_PBX2
Module3_PBX1
Module3_PAX7
Module3_PAX6
Module3_PAX5
Module3_PAX2
Module3_PAX1
Module3_ONECUT1

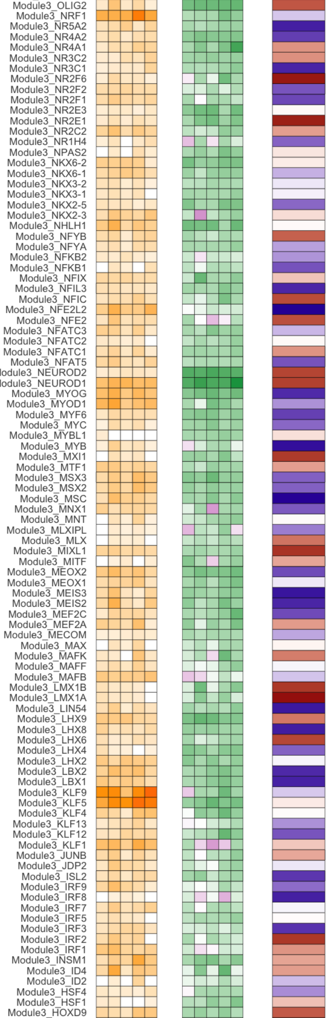
Module3_OLIG2
Module3_NRF1
Module3_NR5A2
Module3_NR4A2
Module3_NR4A1
Module3_NR3C2
Module3_NR3C1
Module3_NR2F6
Module3_NR2F2
Module3_NR2F1
Module3_NR2E3
Module3_NR2E1
Module3_NR2C2
Module3_NR1H4
Module3_NPAS2
Module3_NKX6-2
Module3_NKX6-1
Module3_NKX3-2
Module3_NKX3-1
Module3_NKX2-5
Module3_NKX2-3
Module3_NHLH1
Module3_NFYB
Module3_NFYA
Module3_NFKB2
Module3_NFKB1
Module3_NFIX
Module3_NFIL3
Module3_NFIC
Module3_NFE2L2
Module3_NFE2
Module3_NFATC3
Module3_NFATC2
Module3_NFATC1
Module3_NFAT5
Module3_NEUROD2
Module3_NEUROD1
Module3_MYOG
Module3_MYOD1
Module3_MYF6
Module3_MYC
Module3_MYBL1
Module3_MYB
Module3_MXI1
Module3_MTF1
Module3_MSX3
Module3_MSX2
Module3_MSC
Module3_MNX1
Module3_MNT
Module3_MLXIPL
Module3_MLX
Module3_MIXL1
Module3_MITF
Module3_MEOX2
Module3_MEOX1
Module3_MEIS3
Module3_MEIS2
Module3_MEF2C
Module3_MEF2A
Module3_MECOM
Module3_MAX
Module3_MAFK
Module3_MAFF
Module3_MAFB
Module3_LMX1B
Module3_LMX1A
Module3_LIN54
Module3_LHX9
Module3_LHX8
Module3_LHX6
Module3_LHX4
Module3_LHX2
Module3_LBX2
Module3_LBX1
Module3_KLF9
Module3_KLF5
Module3_KLF4
Module3_KLF13
Module3_KLF12
Module3_KLF1
Module3_JUNB
Module3_JDP2
Module3_ISL2
Module3_IRF9
Module3_IRF8
Module3_IRF7
Module3_IRF5
Module3_IRF3
Module3_IRF2
Module3_IRF1
Module3_INSM1
Module3_ID4
Module3_ID2
Module3_HSF4
Module3_HSF1
Module3_HOXD9

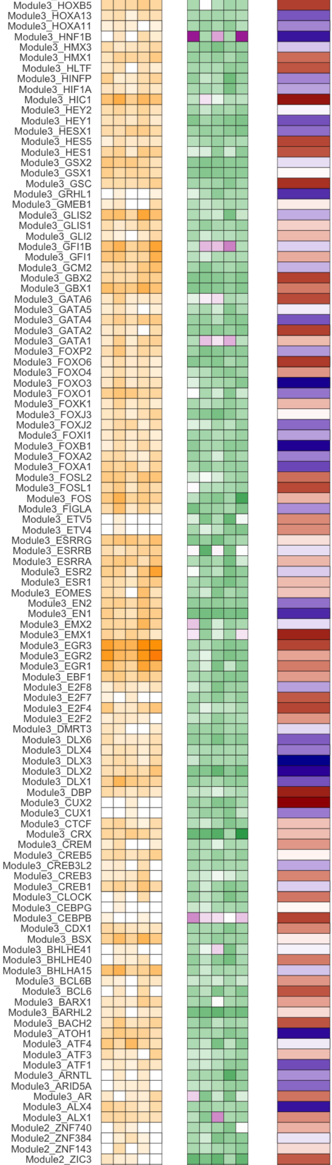
Module3_HOXB5
Module3_HOXA13
Module3_HOXA11
Module3_HNF1B
Module3_HMX3
Module3_HMX1
Module3_HLTF
Module3_HINFP
Module3_HIF1A
Module3_HIC1
Module3_HEY2
Module3_HEY1
Module3_HESX1
Module3_HES5
Module3_HES1
Module3_GSX2
Module3_GSX1
Module3_GSC
Module3_GRHL1
Module3_GMEB1
Module3_GLIS2
Module3_GLIS1
Module3_GLI2
Module3_GFI1B
Module3_GFI1
Module3_GCM2
Module3_GBX2
Module3_GBX1
Module3_GATA6
Module3_GATA5
Module3_GATA4
Module3_GATA2
Module3_GATA1
Module3_FOXP2
Module3_FOXO6
Module3_FOXO4
Module3_FOXO3
Module3_FOXO1
Module3_FOXK1
Module3_FOXJ3
Module3_FOXJ2
Module3_FOXI1
Module3_FOXB1
Module3_FOXA2
Module3_FOXA1
Module3_FOSL2
Module3_FOSL1
Module3_FOS
Module3_FIGLA
Module3_ETV5
Module3_ETV4
Module3_ESRRG
Module3_ESRRB
Module3_ESRRA
Module3_ESR2
Module3_ESR1
Module3_EOMES
Module3_EN2
Module3_EN1
Module3_EMX2
Module3_EMX1
Module3_EGR3
Module3_EGR2
Module3_EGR1
Module3_EBF1
Module3_E2F8
Module3_E2F7
Module3_E2F4
Module3_E2F2
Module3_DMRT3
Module3_DLX6
Module3_DLX4
Module3_DLX3
Module3_DLX2
Module3_DLX1
Module3_DBP
Module3_CUX2
Module3_CUX1
Module3_CTCF
Module3_CRX
Module3_CREM
Module3_CREB5
Module3_CREB3L2
Module3_CREB3
Module3_CREB1
Module3_CLOCK
Module3_CEBPG
Module3_CEBPB
Module3_CDX1
Module3_BSX
Module3_BHLHE41
Module3_BHLHE40
Module3_BHLHA15
Module3_BCL6B
Module3_BCL6
Module3_BARX1
Module3_BARHL2
Module3_BACH2
Module3_ATOH1
Module3_ATF4
Module3_ATF3
Module3_ATF1
Module3_ARNTL
Module3_ARID5A
Module3_AR
Module3_ALX4
Module3_ALX1
Module2_ZNF740
Module2_ZNF384
Module2_ZNF143
Module2_ZIC3

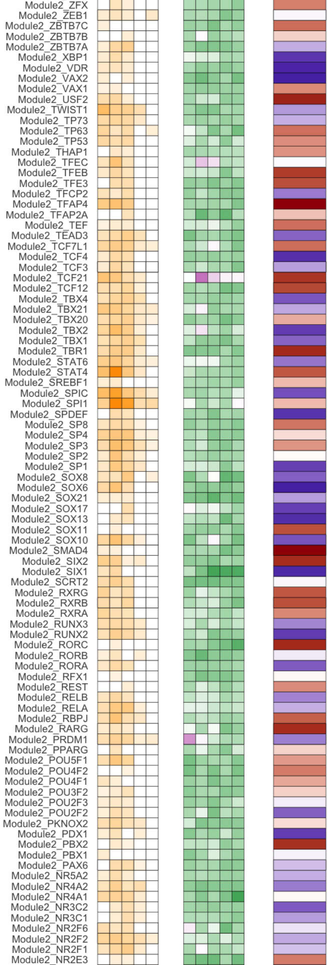
Module2_ZFX
Module2_ZEB1
Module2_ZBTB7C
Module2_ZBTB7B
Module2_ZBTB7A
Module2_XBP1
Module2_VDR
Module2_VAX2
Module2_VAX1
Module2_USF2
Module2_TWIST1
Module2_TP73
Module2_TP63
Module2_TP53
Module2_THAP1
Module2_TFEC
Module2_TFEB
Module2_TFE3
Module2_TFCP2
Module2_TFAP4
Module2_TFAP2A
Module2_TEF
Module2_TEAD3
Module2_TCF7L1
Module2_TCF4
Module2_TCF3
Module2_TCF21
Module2_TCF12
Module2_TBX4
Module2_TBX21
Module2_TBX20
Module2_TBX2
Module2_TBX1
Module2_TBR1
Module2_STAT6
Module2_STAT4
Module2_SREBF1
Module2_SPIC
Module2_SPI1
Module2_SPDEF
Module2_SP8
Module2_SP4
Module2_SP3
Module2_SP2
Module2_SP1
Module2_SOX8
Module2_SOX6
Module2_SOX21
Module2_SOX17
Module2_SOX13
Module2_SOX11
Module2_SOX10
Module2_SMAD4
Module2_SIX2
Module2_SIX1
Module2_SCRT2
Module2_RXRG
Module2_RXRB
Module2_RXRA
Module2_RUNX3
Module2_RUNX2
Module2_RORC
Module2_RORB
Module2_RORA
Module2_RFX1
Module2_REST
Module2_RELB
Module2_RELA
Module2_RBPJ
Module2_RARG
Module2_PRDM1
Module2_PPARG
Module2_POU5F1
Module2_POU4F2
Module2_POU4F1
Module2_POU3F2
Module2_POU2F3
Module2_POU2F2
Module2_PKNOX2
Module2_PDX1
Module2_PBX2
Module2_PBX1
Module2_PAX6
Module2_NR5A2
Module2_NR4A2
Module2_NR4A1
Module2_NR3C2
Module2_NR3C1
Module2_NR2F6
Module2_NR2F2
Module2_NR2F1
Module2_NR2E3

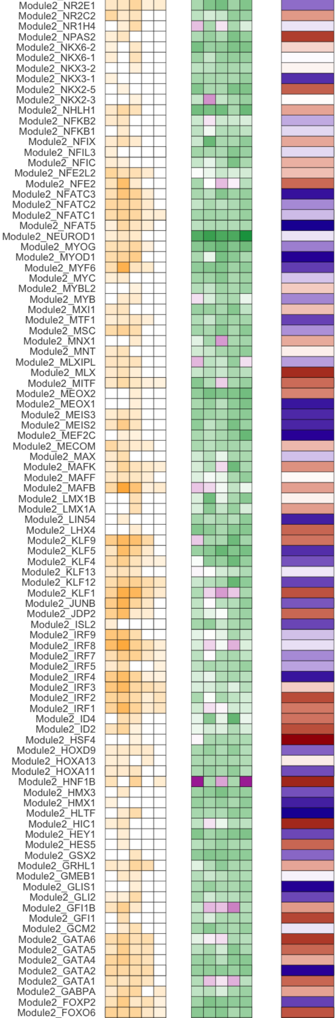
Module2_NR2E1
Module2_NR2C2
Module2_NR1H4
Module2_NPAS2
Module2_NKX6-2
Module2_NKX6-1
Module2_NKX3-2
Module2_NKX3-1
Module2_NKX2-5
Module2_NKX2-3
Module2_NHLH1
Module2_NFKB2
Module2_NFKB1
Module2_NFIX
Module2_NFIL3
Module2_NFIC
Module2_NFE2L2
Module2_NFE2
Module2_NFATC3
Module2_NFATC2
Module2_NFATC1
Module2_NFAT5
Module2_NEUROD1
Module2_MYOG
Module2_MYOD1
Module2_MYF6
Module2_MYC
Module2_MYBL2
Module2_MYB
Module2_MXI1
Module2_MTF1
Module2_MSC
Module2_MNX1
Module2_MNT
Module2_MLXIPL
Module2_MLX
Module2_MITF
Module2_MEOX2
Module2_MEOX1
Module2_MEIS3
Module2_MEIS2
Module2_MEF2C
Module2_MECOM
Module2_MAX
Module2_MAFK
Module2_MAFF
Module2_MAFB
Module2_LMX1B
Module2_LMX1A
Module2_LIN54
Module2_LHX4
Module2_KLF9
Module2_KLF5
Module2_KLF4
Module2_KLF13
Module2_KLF12
Module2_KLF1
Module2_JUNB
Module2_JDP2
Module2_ISL2
Module2_IRF9
Module2_IRF8
Module2_IRF7
Module2_IRF5
Module2_IRF4
Module2_IRF3
Module2_IRF2
Module2_IRF1
Module2_ID4
Module2_ID2
Module2_HSF4
Module2_HOXD9
Module2_HOXA13
Module2_HOXA11
Module2_HNF1B
Module2_HMX3
Module2_HMX1
Module2_HLTF
Module2_HIC1
Module2_HEY1
Module2_HES5
Module2_GSX2
Module2_GRHL1
Module2_GMEB1
Module2_GLIS1
Module2_GLI2
Module2_GFI1B
Module2_GFI1
Module2_GCM2
Module2_GATA6
Module2_GATA5
Module2_GATA4
Module2_GATA2
Module2_GATA1
Module2_GABPA
Module2_FOXP2
Module2_FOXO6

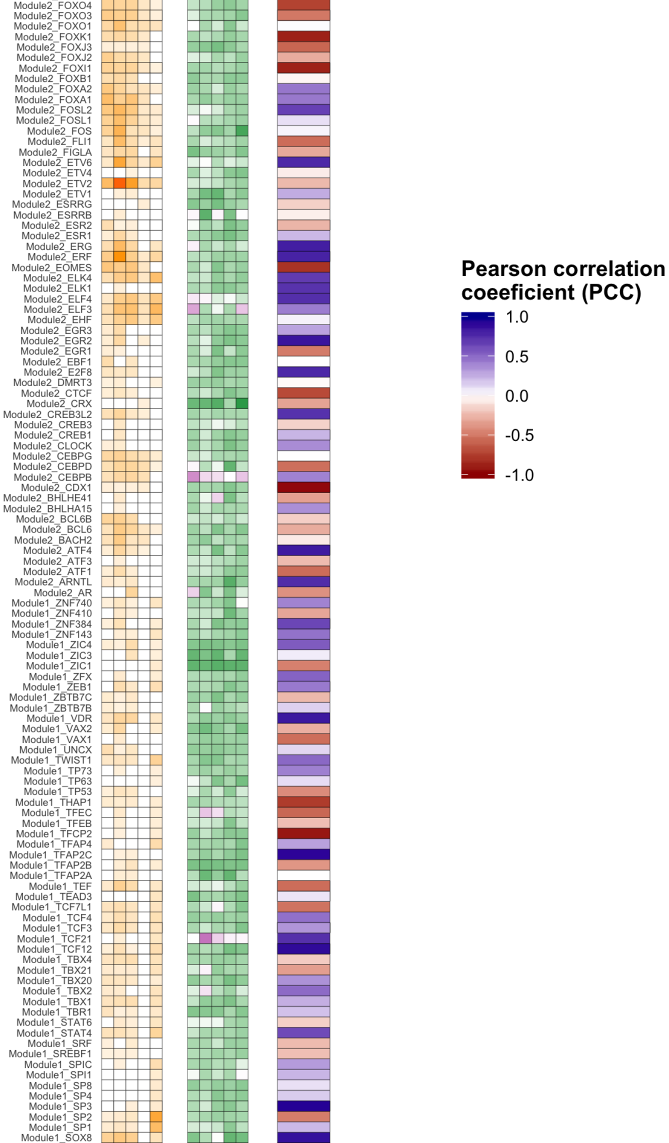
Module2_FOXO4
Module2_FOXO3
Module2_FOXO1
Module2_FOXK1
Module2_FOXJ3
Module2_FOXJ2
Module2_FOXI1
Module2_FOXB1
Module2_FOXA2
Module2_FOXA1
Module2_FOSL2
Module2_FOSL1
Module2_FOS
Module2_FLI1
Module2_FIGLA
Module2_ETV6
Module2_ETV4
Module2_ETV2
Module2_ETV1
Module2_ESRRG
Module2_ESRRB
Module2_ESR2
Module2_ESR1
Module2_ERG
Module2_ERF
Module2_EOMES
Module2_ELK4
Module2_ELK1
Module2_ELF4
Module2_ELF3
Module2_EHF
Module2_EGR3
Module2_EGR2
Module2_EGR1
Module2_EBF1
Module2_E2F8
Module2_DMRT3
Module2_CTCF
Module2_CRX
Module2_CREB3L2
Module2_CREB3
Module2_CREB1
Module2_CLOCK
Module2_CEBPG
Module2_CEBPD
Module2_CEBPB
Module2_CDX1
Module2_BHLHE41
Module2_BHLHA15
Module2_BCL6B
Module2_BCL6
Module2_BACH2
Module2_ATF4
Module2_ATF3
Module2_ATF1
Module2_ARNTL
Module2_AR
Module1_ZNF740
Module1_ZNF410
Module1_ZNF384
Module1_ZNF143
Module1_ZIC4
Module1_ZIC3
Module1_ZIC1
Module1_ZFX
Module1_ZEB1
Module1_ZBTB7C
Module1_ZBTB7B
Module1_VDR
Module1_VAX2
Module1_VAX1
Module1_UNCX
Module1_TWIST1
Module1_TP73
Module1_TP63
Module1_TP53
Module1_THAP1
Module1_TFEC
Module1_TFEB
Module1_TFCP2
Module1_TFAP4
Module1_TFAP2C
Module1_TFAP2B
Module1_TFAP2A
Module1_TEF
Module1_TEAD3
Module1_TCF7L1
Module1_TCF4
Module1_TCF3
Module1_TCF21
Module1_TCF12
Module1_TBX4
Module1_TBX21
Module1_TBX20
Module1_TBX2
Module1_TBX1
Module1_TBR1
Module1_STAT6
Module1_STAT4
Module1_SRF
Module1_SREBF1
Module1_SPIC
Module1_SPI1
Module1_SP8
Module1_SP4
Module1_SP3
Module1_SP2
Module1_SP1
Module1_SOX8
Pearson correlation coeeficient (PCC)
1.0
0.5
0.0
-0.5
-1.0

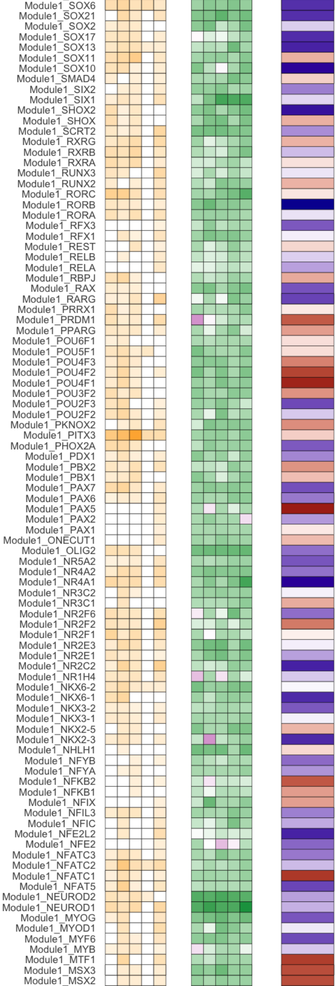
Module1_SOX6
Module1_SOX21
Module1_SOX2
Module1_SOX17
Module1_SOX13
Module1_SOX11
Module1_SOX10
Module1_SMAD4
Module1_SIX2
Module1_SIX1
Module1_SHOX2
Module1_SHOX
Module1_SCRT2
Module1_RXRG
Module1_RXRB
Module1_RXRA
Module1_RUNX3
Module1_RUNX2
Module1_RORC
Module1_RORB
Module1_RORA
Module1_RFX3
Module1_RFX1
Module1_REST
Module1_RELB
Module1_RELA
Module1_RBPJ
Module1_RAX
Module1_RARG
Module1_PRRX1
Module1_PRDM1
Module1_PPARG
Module1_POU6F1
Module1_POU5F1
Module1_POU4F3
Module1_POU4F2
Module1_POU4F1
Module1_POU3F2
Module1_POU2F3
Module1_POU2F2
Module1_PKNOX2
Module1_PITX3
Module1_PHOX2A
Module1_PDX1
Module1_PBX2
Module1_PBX1
Module1_PAX7
Module1_PAX6
Module1_PAX5
Module1_PAX2
Module1_PAX1
Module1_ONECUT1
Module1_OLIG2
Module1_NR5A2
Module1_NR4A2
Module1_NR4A1
Module1_NR3C2
Module1_NR3C1
Module1_NR2F6
Module1_NR2F2
Module1_NR2F1
Module1_NR2E3
Module1_NR2E1
Module1_NR2C2
Module1_NR1H4
Module1_NKX6-2
Module1_NKX6-1
Module1_NKX3-2
Module1_NKX3-1
Module1_NKX2-5
Module1_NKX2-3
Module1_NHLH1
Module1_NFYB
Module1_NFYA
Module1_NFKB2
Module1_NFKB1
Module1_NFIX
Module1_NFIL3
Module1_NFIC
Module1_NFE2L2
Module1_NFE2
Module1_NFATC3
Module1_NFATC2
Module1_NFATC1
Module1_NFAT5
Module1_NEUROD2
Module1_NEUROD1
Module1_MYOG
Module1_MYOD1
Module1_MYF6
Module1_MYB
Module1_MTF1
Module1_MSX3
Module1_MSX2

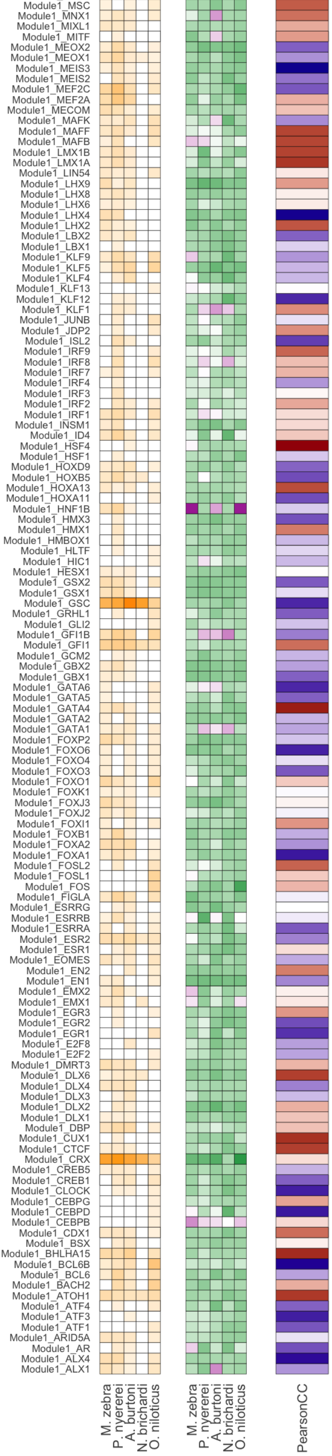
Module1_MSC
Module1_MNX1
Module1_MIXL1
Module1_MITF
Module1_MEOX2
Module1_MEOX1
Module1_MEIS3
Module1_MEIS2
Module1_MEF2C
Module1_MEF2A
Module1_MECOM
Module1_MAFK
Module1_MAFF
Module1_MAFB
Module1_LMX1B
Module1_LMX1A
Module1_LIN54
Module1_LHX9
Module1_LHX8
Module1_LHX6
Module1_LHX4
Module1_LHX2
Module1_LBX2
Module1_LBX1
Module1_KLF9
Module1_KLF5
Module1_KLF4
Module1_KLF13
Module1_KLF12
Module1_KLF1
Module1_JUNB
Module1_JDP2
Module1_ISL2
Module1_IRF9
Module1_IRF8
Module1_IRF7
Module1_IRF4
Module1_IRF3
Module1_IRF2
Module1_IRF1
Module1_INSM1
Module1_ID4
Module1_HSF4
Module1_HSF1
Module1_HOXD9
Module1_HOXB5
Module1_HOXA13
Module1_HOXA11
Module1_HNF1B
Module1_HMX3
Module1_HMX1
Module1_HMBOX1
Module1_HLTF
Module1_HIC1
Module1_HESX1
Module1_GSX2
Module1_GSX1
Module1_GSC
Module1_GRHL1
Module1_GLI2
Module1_GFI1B
Module1_GFI1
Module1_GCM2
Module1_GBX2
Module1_GBX1
Module1_GATA6
Module1_GATA5
Module1_GATA4
Module1_GATA2
Module1_GATA1
Module1_FOXP2
Module1_FOXO6
Module1_FOXO4
Module1_FOXO3
Module1_FOXO1
Module1_FOXK1
Module1_FOXJ3
Module1_FOXJ2
Module1_FOXI1
Module1_FOXB1
Module1_FOXA2
Module1_FOXA1
Module1_FOSL2
Module1_FOSL1
Module1_FOS
Module1_FIGLA
Module1_ESRRG
Module1_ESRRB
Module1_ESRRA
Module1_ESR2
Module1_ESR1
Module1_EOMES
Module1_EN2
Module1_EN1
Module1_EMX2
Module1_EMX1
Module1_EGR3
Module1_EGR2
Module1_EGR1
Module1_E2F8
Module1_E2F2
Module1_DMRT3
Module1_DLX6
Module1_DLX4
Module1_DLX3
Module1_DLX2
Module1_DLX1
Module1_DBP
Module1_CUX1
Module1_CTCF
Module1_CRX
Module1_CREB5
Module1_CREB1
Module1_CLOCK
Module1_CEBPG
Module1_CEBPD
Module1_CEBPB
Module1_CDX1
Module1_BSX
Module1_BHLHA15
Module1_BCL6B
Module1_BCL6
Module1_BACH2
Module1_ATOH1
Module1_ATF4
Module1_ATF3
Module1_ATF1
Module1_ARID5A
Module1_AR
Module1_ALX4
Module1_ALX1
M. zebra
P. nyererei
A. burtoni
N. brichardi
O. niloticus
M. zebra
P. nyererei
A. burtoni
N. brichardi
O. niloticus
PearsonCC

Additional file 2: Fig. S7

Heatmap matrices of enrichment, expression and Pearson correlation between the two for each motif, across muscle tissue

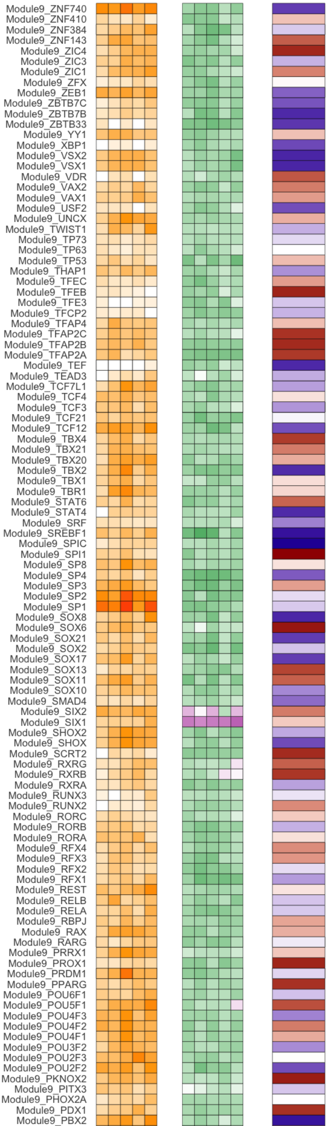

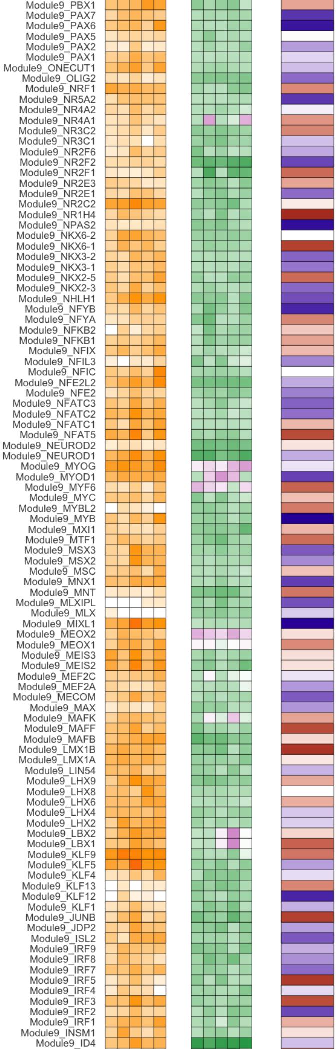
Module9_PBX1
Module9_PAX7
Module9_PAX6
Module9_PAX5
Module9_PAX2
Module9_PAX1
Module9_ONECUT1
Module9_OLIG2
Module9_NRF1
Module9_NR5A2
Module9_NR4A2
Module9_NR4A1
Module9_NR3C2
Module9_NR3C1
Module9_NR2F6
Module9_NR2F2
Module9_NR2F1
Module9_NR2E3
Module9_NR2E1
Module9_NR2C2
Module9_NR1H4
Module9_NPAS2
Module9_NKX6-2
Module9_NKX6-1
Module9_NKX3-2
Module9_NKX3-1
Module9_NKX2-5
Module9_NKX2-3
Module9_NHLH1
Module9_NFYB
Module9_NFYA
Module9_NFKB2
Module9_NFKB1
Module9_NFIX
Module9_NFIL3
Module9_NFIC
Module9_NFE2L2
Module9_NFE2
Module9_NFATC3
Module9_NFATC2
Module9_NFATC1
Module9_NFAT5
Module9_NEUROD2
Module9_NEUROD1
Module9_MYOG
Module9_MYOD1
Module9_MYF6
Module9_MYC
Module9_MYBL2
Module9_MYB
Module9_MXI1
Module9_MTF1
Module9_MSX3
Module9_MSX2
Module9_MSC
Module9_MNX1
Module9_MNT
Module9_MLXIPL
Module9_MLX
Module9_MIXL1
Module9_MEOX2
Module9_MEOX1
Module9_MEIS3
Module9_MEIS2
Module9_MEF2C
Module9_MEF2A
Module9_MECOM
Module9_MAX
Module9_MAFK
Module9_MAFF
Module9_MAFB
Module9_LMX1B
Module9_LMX1A
Module9_LIN54
Module9_LHX9
Module9_LHX8
Module9_LHX6
Module9_LHX4
Module9_LHX2
Module9_LBX2
Module9_LBX1
Module9_KLF9
Module9_KLF5
Module9_KLF4
Module9_KLF13
Module9_KLF12
Module9_KLF1
Module9_JUNB
Module9_JDP2
Module9_ISL2
Module9_IRF9
Module9_IRF8
Module9_IRF7
Module9_IRF5
Module9_IRF4
Module9_IRF3
Module9_IRF2
Module9_IRF1
Module9_INSM1
Module9_ID4

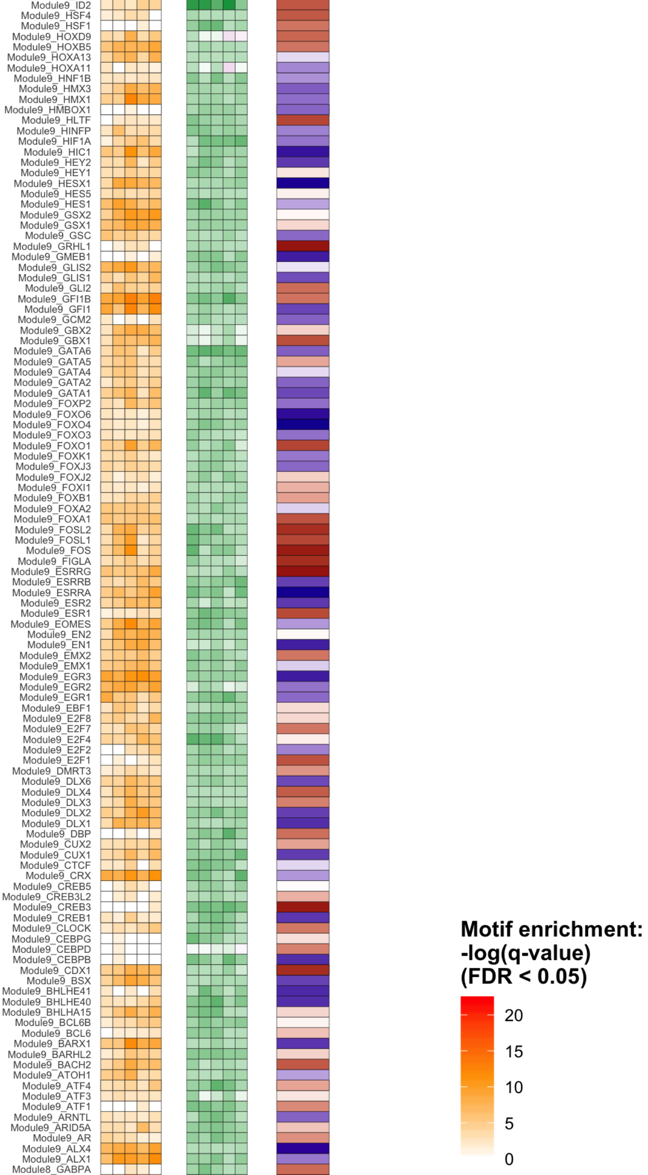

Module9_ID2
Module9_HSF4
Module9_HSF1
Module9_HOXD9
Module9_HOXB5
Module9_HOXA13
Module9_HOXA11
Module9_HNF1B
Module9_HMX3
Module9_HMX1
Module9_HMBOX1
Module9_HLTF
Module9_HINFP
Module9_HIF1A
Module9_HIC1
Module9_HEY2
Module9_HEY1
Module9_HESX1
Module9_HES5
Module9_HES1
Module9_GSX2
Module9_GSX1
Module9_GSC
Module9_GRHL1
Module9_GMEB1
Module9_GLIS2
Module9_GLIS1
Module9_GLI2
Module9_GFI1B
Module9_GFI1
Module9_GCM2
Module9_GBX2
Module9_GBX1
Module9_GATA6
Module9_GATA5
Module9_GATA4
Module9_GATA2
Module9_GATA1
Module9_FOXP2
Module9_FOXO6
Module9_FOXO4
Module9_FOXO3
Module9_FOXO1
Module9_FOXK1
Module9_FOXJ3
Module9_FOXJ2
Module9_FOXI1
Module9_FOXB1
Module9_FOXA2
Module9_FOXA1
Module9_FOSL2
Module9_FOSL1
Module9_FOS
Module9_FIGLA
Module9_ESRRG
Module9_ESRRB
Module9_ESRRA
Module9_ESR2
Module9_ESR1
Module9_EOMES
Module9_EN2
Module9_EN1
Module9_EMX2
Module9_EMX1
Module9_EGR3
Module9_EGR2
Module9_EGR1
Module9_EBF1
Module9_E2F8
Module9_E2F7
Module9_E2F4
Module9_E2F2
Module9_E2F1
Module9_DMRT3
Module9_DLX6
Module9_DLX4
Module9_DLX3
Module9_DLX2
Module9_DLX1
Module9_DBP
Module9_CUX2
Module9_CUX1
Module9_CTCF
Module9_CRX
Module9_CREB5
Module9_CREB3L2
Module9_CREB3
Module9_CREB1
Module9_CLOCK
Module9_CEBPG
Module9_CEBPD
Module9_CEBPB
Module9_CDX1
Module9_BSX
Module9_BHLHE41
Module9_BHLHE40
Module9_BHLHA15
Module9_BCL6B
Module9_BCL6
Module9_BARX1
Module9_BARHL2
Module9_BACH2
Module9_ATOH1
Module9_ATF4
Module9_ATF3
Module9_ATF1
Module9_ARNTL
Module9_ARID5A
Module9_AR
Module9_ALX4
Module9_ALX1
Module8_GABPA
Motif enrichment:
-log(q-value)
(FDR < 0.05)
20
15
10
5
0

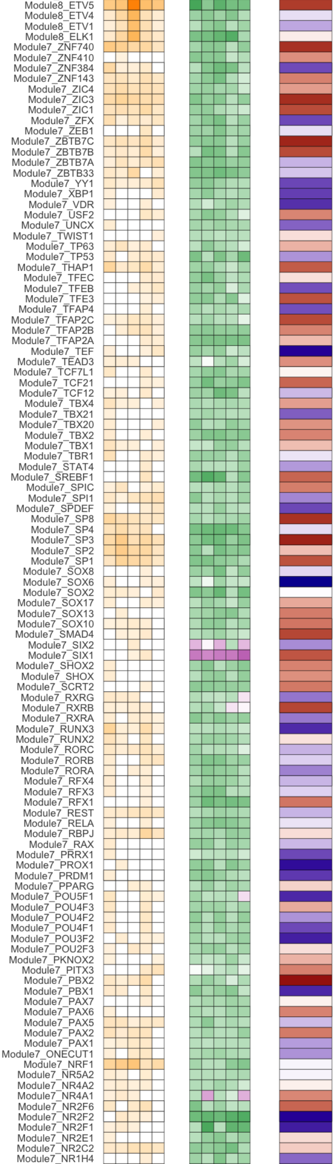
Module8_ETV5
Module8_ETV4
Module8_ETV1
Module8_ELK1
Module7_ZNF740
Module7_ZNF410
Module7_ZNF384
Module7_ZNF143
Module7_ZIC4
Module7_ZIC3
Module7_ZIC1
Module7_ZFX
Module7_ZEB1
Module7_ZBTB7C
Module7_ZBTB7B
Module7_ZBTB7A
Module7_ZBTB33
Module7_YY1
Module7_XBP1
Module7_VDR
Module7_USF2
Module7_UNCX
Module7_TWIST1
Module7_TP63
Module7_TP53
Module7_THAP1
Module7_TFEC
Module7_TFEB
Module7_TFE3
Module7_TFAP4
Module7_TFAP2C
Module7_TFAP2B
Module7_TFAP2A
Module7_TEF
Module7_TEAD3
Module7_TCF7L1
Module7_TCF21
Module7_TCF12
Module7_TBX4
Module7_TBX21
Module7_TBX20
Module7_TBX2
Module7_TBX1
Module7_TBR1
Module7_STAT4
Module7_SREBF1
Module7_SPIC
Module7_SPI1
Module7_SPDEF
Module7_SP8
Module7_SP4
Module7_SP3
Module7_SP2
Module7_SP1
Module7_SOX8
Module7_SOX6
Module7_SOX2
Module7_SOX17
Module7_SOX13
Module7_SOX10
Module7_SMAD4
Module7_SIX2
Module7_SIX1
Module7_SHOX2
Module7_SHOX
Module7_SCRT2
Module7_RXRG
Module7_RXRB
Module7_RXRA
Module7_RUNX3
Module7_RUNX2
Module7_RORC
Module7_RORB
Module7_RORA
Module7_RFX4
Module7_RFX3
Module7_RFX1
Module7_REST
Module7_RELA
Module7_RBPJ
Module7_RAX
Module7_PRRX1
Module7_PROX1
Module7_PRDM1
Module7_PPARG
Module7_POU5F1
Module7_POU4F3
Module7_POU4F2
Module7_POU4F1
Module7_POU3F2
Module7_POU2F3
Module7_PKNOX2
Module7_PITX3
Module7_PBX2
Module7_PBX1
Module7_PAX7
Module7_PAX6
Module7_PAX5
Module7_PAX2
Module7_PAX1
Module7_ONECUT1
Module7_NRF1
Module7_NR5A2
Module7_NR4A2
Module7_NR4A1
Module7_NR2F6
Module7_NR2F2
Module7_NR2F1
Module7_NR2E1
Module7_NR2C2
Module7_NR1H4

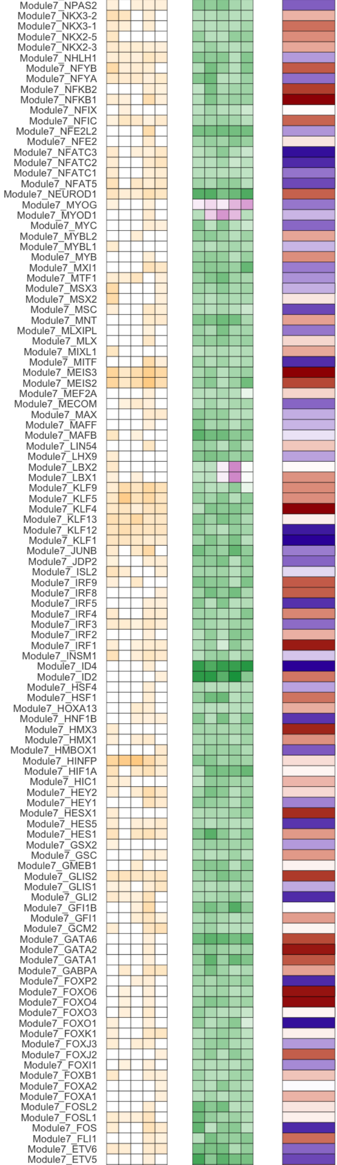
Module7_NPAS2
Module7_NKX3-2
Module7_NKX3-1
Module7_NKX2-5
Module7_NKX2-3
Module7_NHLH1
Module7_NFYB
Module7_NFYA
Module7_NFKB2
Module7_NFKB1
Module7_NFIX
Module7_NFIC
Module7_NFE2L2
Module7_NFE2
Module7_NFATC3
Module7_NFATC2
Module7_NFATC1
Module7_NFAT5
Module7_NEUROD1
Module7_MYOG
Module7_MYOD1
Module7_MYC
Module7_MYBL2
Module7_MYBL1
Module7_MYB
Module7_MXI1
Module7_MTF1
Module7_MSX3
Module7_MSX2
Module7_MSC
Module7_MNT
Module7_MLXIPL
Module7_MLX
Module7_MIXL1
Module7_MITF
Module7_MEIS3
Module7_MEIS2
Module7_MEF2A
Module7_MECOM
Module7_MAX
Module7_MAFF
Module7_MAFB
Module7_LIN54
Module7_LHX9
Module7_LBX2
Module7_LBX1
Module7_KLF9
Module7_KLF5
Module7_KLF4
Module7_KLF13
Module7_KLF12
Module7_KLF1
Module7_JUNB
Module7_JDP2
Module7_ISL2
Module7_IRF9
Module7_IRF8
Module7_IRF5
Module7_IRF4
Module7_IRF3
Module7_IRF2
Module7_IRF1
Module7_INSM1
Module7_ID4
Module7_ID2
Module7_HSF4
Module7_HSF1
Module7_HOXA13
Module7_HNF1B
Module7_HMX3
Module7_HMX1
Module7_HMBOX1
Module7_HINFP
Module7_HIF1A
Module7_HIC1
Module7_HEY2
Module7_HEY1
Module7_HESX1
Module7_HES5
Module7_HES1
Module7_GSX2
Module7_GSC
Module7_GMEB1
Module7_GLIS2
Module7_GLIS1
Module7_GLI2
Module7_GFI1B
Module7_GFI1
Module7_GCM2
Module7_GATA6
Module7_GATA2
Module7_GATA1
Module7_GABPA
Module7_FOXP2
Module7_FOXO6
Module7_FOXO4
Module7_FOXO3
Module7_FOXO1
Module7_FOXK1
Module7_FOXJ3
Module7_FOXJ2
Module7_FOXI1
Module7_FOXB1
Module7_FOXA2
Module7_FOXA1
Module7_FOSL2
Module7_FOSL1
Module7_FOS
Module7_FLI1
Module7_ETV6
Module7_ETV5

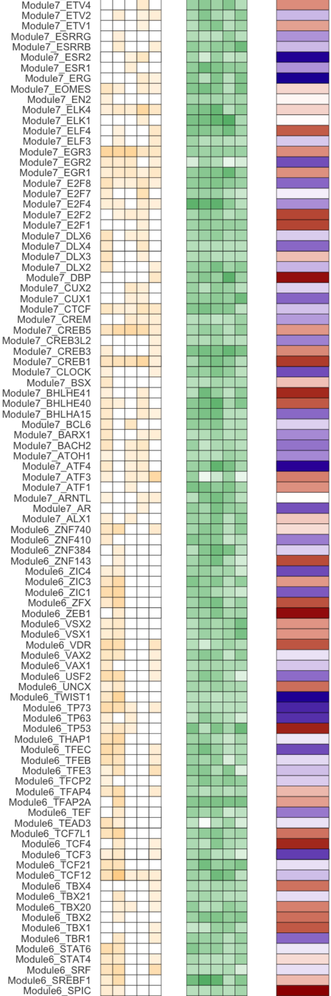
Module7_ETV4
Module7_ETV2
Module7_ETV1
Module7_ESRRG
Module7_ESRRB
Module7_ESR2
Module7_ESR1
Module7_ERG
Module7_EOMES
Module7_EN2
Module7_ELK4
Module7_ELK1
Module7_ELF4
Module7_ELF3
Module7_EGR3
Module7_EGR2
Module7_EGR1
Module7_E2F8
Module7_E2F7
Module7_E2F4
Module7_E2F2
Module7_E2F1
Module7_DLX6
Module7_DLX4
Module7_DLX3
Module7_DLX2
Module7_DBP
Module7_CUX2
Module7_CUX1
Module7_CTCF
Module7_CREM
Module7_CREB5
Module7_CREB3L2
Module7_CREB3
Module7_CREB1
Module7_CLOCK
Module7_BSX
Module7_BHLHE41
Module7_BHLHE40
Module7_BHLHA15
Module7_BCL6
Module7_BARX1
Module7_BACH2
Module7_ATOH1
Module7_ATF4
Module7_ATF3
Module7_ATF1
Module7_ARNTL
Module7_AR
Module7_ALX1
Module6_ZNF740
Module6_ZNF410
Module6_ZNF384
Module6_ZNF143
Module6_ZIC4
Module6_ZIC3
Module6_ZIC1
Module6_ZFX
Module6_ZEB1
Module6_VSX2
Module6_VSX1
Module6_VDR
Module6_VAX2
Module6_VAX1
Module6_USF2
Module6_UNCX
Module6_TWIST1
Module6_TP73
Module6_TP63
Module6_TP53
Module6_THAP1
Module6_TFEC
Module6_TFEB
Module6_TFE3
Module6_TFCP2
Module6_TFAP4
Module6_TFAP2A
Module6_TEF
Module6_TEAD3
Module6_TCF7L1
Module6_TCF4
Module6_TCF3
Module6_TCF21
Module6_TCF12
Module6_TBX4
Module6_TBX21
Module6_TBX20
Module6_TBX2
Module6_TBX1
Module6_TBR1
Module6_STAT6
Module6_STAT4
Module6_SRF
Module6_SREBF1
Module6_SPIC

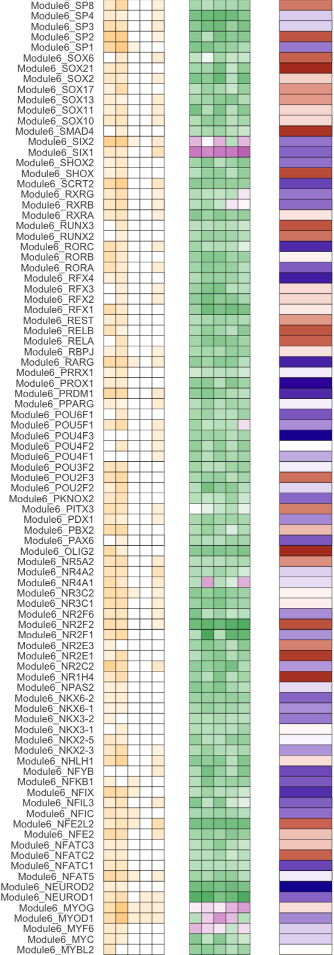
Module6_SP8
Module6_SP4
Module6_SP3
Module6_SP2
Module6_SP1
Module6_SOX6
Module6_SOX21
Module6_SOX2
Module6_SOX17
Module6_SOX13
Module6_SOX11
Module6_SOX10
Module6_SMAD4
Module6_SIX2
Module6_SIX1
Module6_SHOX2
Module6_SHOX
Module6_SCRT2
Module6_RXRG
Module6_RXRB
Module6_RXRA
Module6_RUNX3
Module6_RUNX2
Module6_RORC
Module6_RORB
Module6_RORA
Module6_RFX4
Module6_RFX3
Module6_RFX2
Module6_RFX1
Module6_REST
Module6_RELB
Module6_RELA
Module6_RBPJ
Module6_RARG
Module6_PRRX1
Module6_PROX1
Module6_PRDM1
Module6_PPARG
Module6_POU6F1
Module6_POU5F1
Module6_POU4F3
Module6_POU4F2
Module6_POU4F1
Module6_POU3F2
Module6_POU2F3
Module6_POU2F2
Module6_PKNOX2
Module6_PITX3
Module6_PDX1
Module6_PBX2
Module6_PAX6
Module6_OLIG2
Module6_NR5A2
Module6_NR4A2
Module6_NR4A1
Module6_NR3C2
Module6_NR3C1
Module6_NR2F6
Module6_NR2F2
Module6_NR2F1
Module6_NR2E3
Module6_NR2E1
Module6_NR2C2
Module6_NR1H4
Module6_NPAS2
Module6_NKX6-2
Module6_NKX6-1
Module6_NKX3-2
Module6_NKX3-1
Module6_NKX2-5
Module6_NKX2-3
Module6_NHLH1
Module6_NFYB
Module6_NFKB1
Module6_NFIX
Module6_NFIL3
Module6_NFIC
Module6_NFE2L2
Module6_NFE2
Module6_NFATC3
Module6_NFATC2
Module6_NFATC1
Module6_NFAT5
Module6_NEUROD2
Module6_NEUROD1
Module6_MYOG
Module6_MYOD1
Module6_MYF6
Module6_MYC
Module6_MYBL2

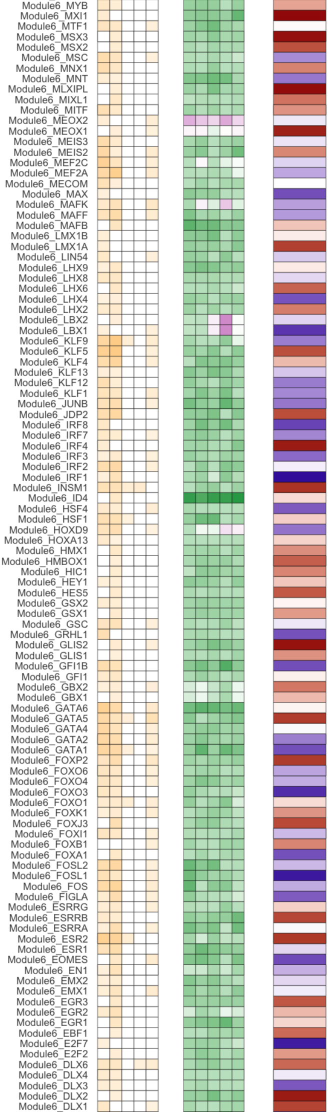
Module6_MYB
Module6_MXI1
Module6_MTF1
Module6_MSX3
Module6_MSX2
Module6_MSC
Module6_MNX1
Module6_MNT
Module6_MLXIPL
Module6_MIXL1
Module6_MITF
Module6_MEOX2
Module6_MEOX1
Module6_MEIS3
Module6_MEIS2
Module6_MEF2C
Module6_MEF2A
Module6_MECOM
Module6_MAX
Module6_MAFK
Module6_MAFF
Module6_MAFB
Module6_LMX1B
Module6_LMX1A
Module6_LIN54
Module6_LHX9
Module6_LHX8
Module6_LHX6
Module6_LHX4
Module6_LHX2
Module6_LBX2
Module6_LBX1
Module6_KLF9
Module6_KLF5
Module6_KLF4
Module6_KLF13
Module6_KLF12
Module6_KLF1
Module6_JUNB
Module6_JDP2
Module6_IRF8
Module6_IRF7
Module6_IRF4
Module6_IRF3
Module6_IRF2
Module6_IRF1
Module6_INSM1
Module6_ID4
Module6_HSF4
Module6_HSF1
Module6_HOXD9
Module6_HOXA13
Module6_HMX1
Module6_HMBOX1
Module6_HIC1
Module6_HEY1
Module6_HES5
Module6_GSX2
Module6_GSX1
Module6_GSC
Module6_GRHL1
Module6_GLIS2
Module6_GLIS1
Module6_GFI1B
Module6_GFI1
Module6_GBX2
Module6_GBX1
Module6_GATA6
Module6_GATA5
Module6_GATA4
Module6_GATA2
Module6_GATA1
Module6_FOXP2
Module6_FOXO6
Module6_FOXO4
Module6_FOXO3
Module6_FOXO1
Module6_FOXK1
Module6_FOXJ3
Module6_FOXI1
Module6_FOXB1
Module6_FOXA1
Module6_FOSL2
Module6_FOSL1
Module6_FOS
Module6_FIGLA
Module6_ESRRG
Module6_ESRRB
Module6_ESRRA
Module6_ESR2
Module6_ESR1
Module6_EOMES
Module6_EN1
Module6_EMX2
Module6_EMX1
Module6_EGR3
Module6_EGR2
Module6_EGR1
Module6_EBF1
Module6_E2F7
Module6_E2F2
Module6_DLX6
Module6_DLX4
Module6_DLX3
Module6_DLX2
Module6_DLX1

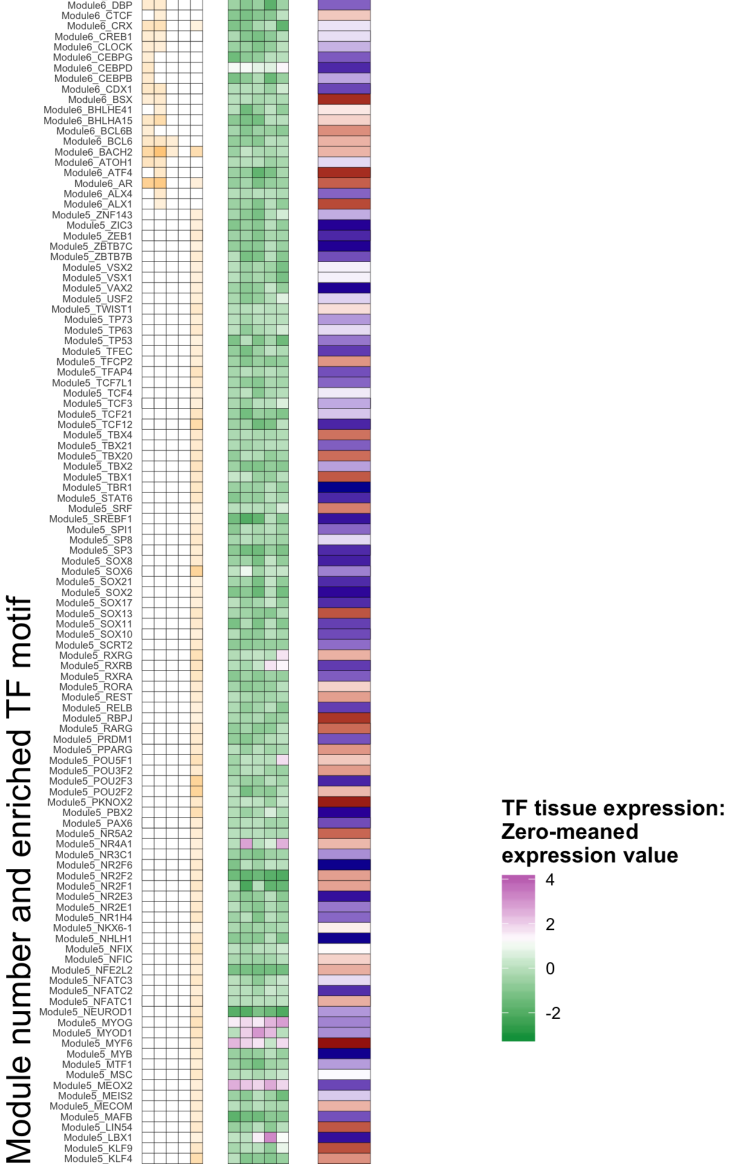
Module number and enriched TF motif
Module6_DBP
Module6_CTCF
Module6_CRX
Module6_CREB1
Module6_CLOCK
Module6_CEBPG
Module6_CEBPD
Module6_CEBPB
Module6_CDX1
Module6_BSX
Module6_BHLHE41
Module6_BHLHA15
Module6_BCL6B
Module6_BCL6
Module6_BACH2
Module6_ATOH1
Module6_ATF4
Module6_AR
Module6_ALX4
Module6_ALX1
Module5_ZNF143
Module5_ZIC3
Module5_ZEB1
Module5_ZBTB7C
Module5_ZBTB7B
Module5_VSX2
Module5_VSX1
Module5_VAX2
Module5_USF2
Module5_TWIST1
Module5_TP73
Module5_TP63
Module5_TP53
Module5_TFEC
Module5_TFCP2
Module5_TFAP4
Module5_TCF7L1
Module5_TCF4
Module5_TCF3
Module5_TCF21
Module5_TCF12
Module5_TBX4
Module5_TBX21
Module5_TBX20
Module5_TBX2
Module5_TBX1
Module5_TBR1
Module5_STAT6
Module5_SRF
Module5_SREBF1
Module5_SPI1
Module5_SP8
Module5_SP3
Module5_SOX8
Module5_SOX6
Module5_SOX21
Module5_SOX2
Module5_SOX17
Module5_SOX13
Module5_SOX11
Module5_SOX10
Module5_SCRT2
Module5_RXRG
Module5_RXRB
Module5_RXRA
Module5_RORA
Module5_REST
Module5_RELB
Module5_RBPJ
Module5_RARG
Module5_PRDM1
Module5_PPARG
Module5_POU5F1
Module5_POU3F2
Module5_POU2F3
Module5_POU2F2
Module5_PKNOX2
Module5_PBX2
Module5_PAX6
Module5_NR5A2
Module5_NR4A1
Module5_NR3C1
Module5_NR2F6
Module5_NR2F2
Module5_NR2F1
Module5_NR2E3
Module5_NR2E1
Module5_NR1H4
Module5_NKX6-1
Module5_NHLH1
Module5_NFIX
Module5_NFIC
Module5_NFE2L2
Module5_NFATC3
Module5_NFATC2
Module5_NFATC1
Module5_NEUROD1
Module5_MYOG
Module5_MYOD1
Module5_MYF6
Module5_MYB
Module5_MTF1
Module5_MSC
Module5_MEOX2
Module5_MEIS2
Module5_MECOM
Module5_MAFB
Module5_LIN54
Module5_LBX1
Module5_KLF9
Module5_KLF4
TF tissue expression:
Zero-meaned
expression value
4
2
0
-2

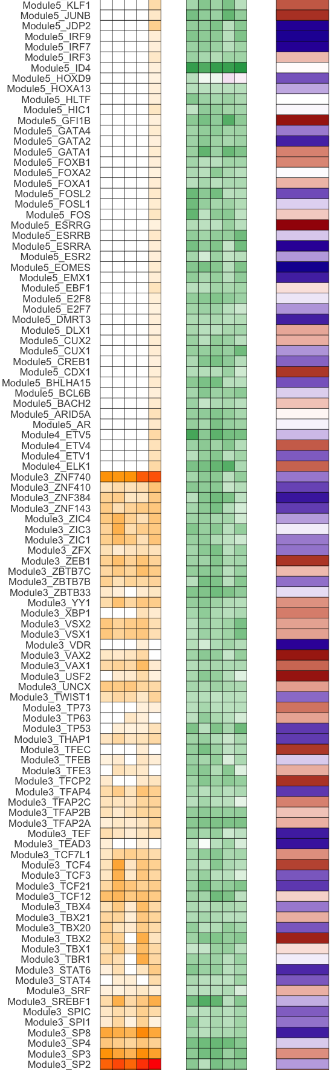

Module5_KLF1
Module5_JUNB
Module5_JDP2
Module5_IRF9
Module5_IRF7
Module5_IRF3
Module5_ID4
Module5_HOXD9
Module5_HOXA13
Module5_HLTF
Module5_HIC1
Module5_GFI1B
Module5_GATA4
Module5_GATA2
Module5_GATA1
Module5_FOXB1
Module5_FOXA2
Module5_FOXA1
Module5_FOSL2
Module5_FOSL1
Module5_FOS
Module5_ESRRG
Module5_ESRRB
Module5_ESRRA
Module5_ESR2
Module5_EOMES
Module5_EMX1
Module5_EBF1
Module5_E2F8
Module5_E2F7
Module5_DMRT3
Module5_DLX1
Module5_CUX2
Module5_CUX1
Module5_CREB1
Module5_CDX1
Module5_BHLHA15
Module5_BCL6B
Module5_BACH2
Module5_ARID5A
Module5_AR
Module4_ETV5
Module4_ETV4
Module4_ETV1
Module4_ELK1
Module3_ZNF740
Module3_ZNF410
Module3_ZNF384
Module3_ZNF143
Module3_ZIC4
Module3_ZIC3
Module3_ZIC1
Module3_ZFX
Module3_ZEB1
Module3_ZBTB7C
Module3_ZBTB7B
Module3_ZBTB33
Module3_YY1
Module3_XBP1
Module3_VSX2
Module3_VSX1
Module3_VDR
Module3_VAX2
Module3_VAX1
Module3_USF2
Module3_UNCX
Module3_TWIST1
Module3_TP73
Module3_TP63
Module3_TP53
Module3_THAP1
Module3_TFEC
Module3_TFEB
Module3_TFE3
Module3_TFCP2
Module3_TFAP4
Module3_TFAP2C
Module3_TFAP2B
Module3_TFAP2A
Module3_TEF
Module3_TEAD3
Module3_TCF7L1
Module3_TCF4
Module3_TCF3
Module3_TCF21
Module3_TCF12
Module3_TBX4
Module3_TBX21
Module3_TBX20
Module3_TBX2
Module3_TBX1
Module3_TBR1
Module3_STAT6
Module3_STAT4
Module3_SRF
Module3_SREBF1
Module3_SPIC
Module3_SPI1
Module3_SP8
Module3_SP4
Module3_SP3
Module3_SP2

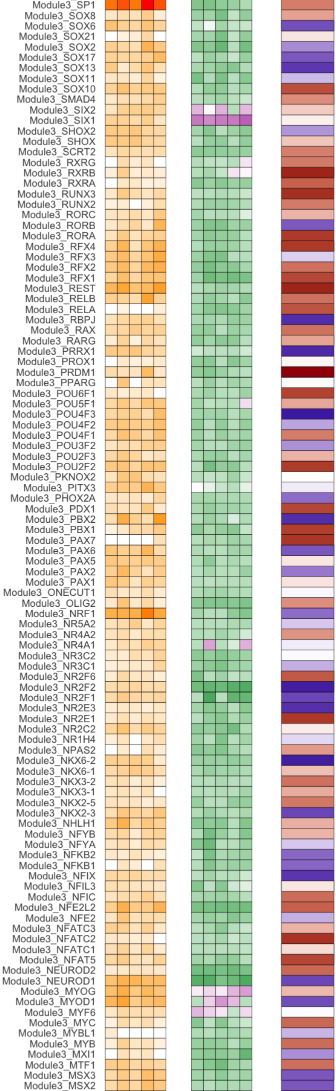
Module3_SP1
Module3_SOX8
Module3_SOX6
Module3_SOX21
Module3_SOX2
Module3_SOX17
Module3_SOX13
Module3_SOX11
Module3_SOX10
Module3_SMAD4
Module3_SIX2
Module3_SIX1
Module3_SHOX2
Module3_SHOX
Module3_SCRT2
Module3_RXRG
Module3_RXRB
Module3_RXRA
Module3_RUNX3
Module3_RUNX2
Module3_RORC
Module3_RORB
Module3_RORA
Module3_RFX4
Module3_RFX3
Module3_RFX2
Module3_RFX1
Module3_REST
Module3_RELB
Module3_RELA
Module3_RBPJ
Module3_RAX
Module3_RARG
Module3_PRRX1
Module3_PROX1
Module3_PRDM1
Module3_PPARG
Module3_POU6F1
Module3_POU5F1
Module3_POU4F3
Module3_POU4F2
Module3_POU4F1
Module3_POU3F2
Module3_POU2F3
Module3_POU2F2
Module3_PKNOX2
Module3_PITX3
Module3_PHOX2A
Module3_PDX1
Module3_PBX2
Module3_PBX1
Module3_PAX7
Module3_PAX6
Module3_PAX5
Module3_PAX2
Module3_PAX1
Module3_ONECUT1
Module3_OLIG2
Module3_NRF1
Module3_NR5A2
Module3_NR4A2
Module3_NR4A1
Module3_NR3C2
Module3_NR3C1
Module3_NR2F6
Module3_NR2F2
Module3_NR2F1
Module3_NR2E3
Module3_NR2E1
Module3_NR2C2
Module3_NR1H4
Module3_NPAS2
Module3_NKX6-2
Module3_NKX6-1
Module3_NKX3-2
Module3_NKX3-1
Module3_NKX2-5
Module3_NKX2-3
Module3_NHLH1
Module3_NFYB
Module3_NFYA
Module3_NFKB2
Module3_NFKB1
Module3_NFIX
Module3_NFIL3
Module3_NFIC
Module3_NFE2L2
Module3_NFE2
Module3_NFATC3
Module3_NFATC2
Module3_NFATC1
Module3_NFAT5
Module3_NEUROD2
Module3_NEUROD1
Module3_MYOG
Module3_MYOD1
Module3_MYF6
Module3_MYC
Module3_MYBL1
Module3_MYB
Module3_MXI1
Module3_MTF1
Module3_MSX3
Module3_MSX2

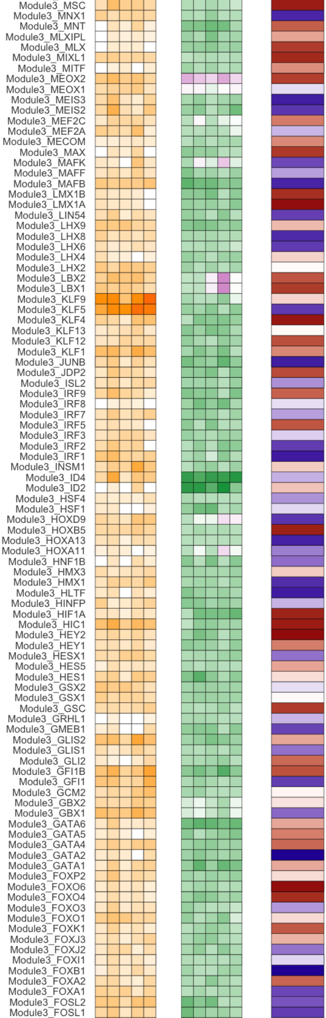
Module3_MSC
Module3_MNX1
Module3_MNT
Module3_MLXIPL
Module3_MLX
Module3_MIXL1
Module3_MITF
Module3_MEOX2
Module3_MEOX1
Module3_MEIS3
Module3_MEIS2
Module3_MEF2C
Module3_MEF2A
Module3_MECOM
Module3_MAX
Module3_MAFK
Module3_MAFF
Module3_MAFB
Module3_LMX1B
Module3_LMX1A
Module3_LIN54
Module3_LHX9
Module3_LHX8
Module3_LHX6
Module3_LHX4
Module3_LHX2
Module3_LBX2
Module3_LBX1
Module3_KLF9
Module3_KLF5
Module3_KLF4
Module3_KLF13
Module3_KLF12
Module3_KLF1
Module3_JUNB
Module3_JDP2
Module3_ISL2
Module3_IRF9
Module3_IRF8
Module3_IRF7
Module3_IRF5
Module3_IRF3
Module3_IRF2
Module3_IRF1
Module3_INSM1
Module3_ID4
Module3_ID2
Module3_HSF4
Module3_HSF1
Module3_HOXD9
Module3_HOXB5
Module3_HOXA13
Module3_HOXA11
Module3_HNF1B
Module3_HMX3
Module3_HMX1
Module3_HLTF
Module3_HINFP
Module3_HIF1A
Module3_HIC1
Module3_HEY2
Module3_HEY1
Module3_HESX1
Module3_HES5
Module3_HES1
Module3_GSX2
Module3_GSX1
Module3_GSC
Module3_GRHL1
Module3_GMEB1
Module3_GLIS2
Module3_GLIS1
Module3_GLI2
Module3_GFI1B
Module3_GFI1
Module3_GCM2
Module3_GBX2
Module3_GBX1
Module3_GATA6
Module3_GATA5
Module3_GATA4
Module3_GATA2
Module3_GATA1
Module3_FOXP2
Module3_FOXO6
Module3_FOXO4
Module3_FOXO3
Module3_FOXO1
Module3_FOXK1
Module3_FOXJ3
Module3_FOXJ2
Module3_FOXI1
Module3_FOXB1
Module3_FOXA2
Module3_FOXA1
Module3_FOSL2
Module3_FOSL1

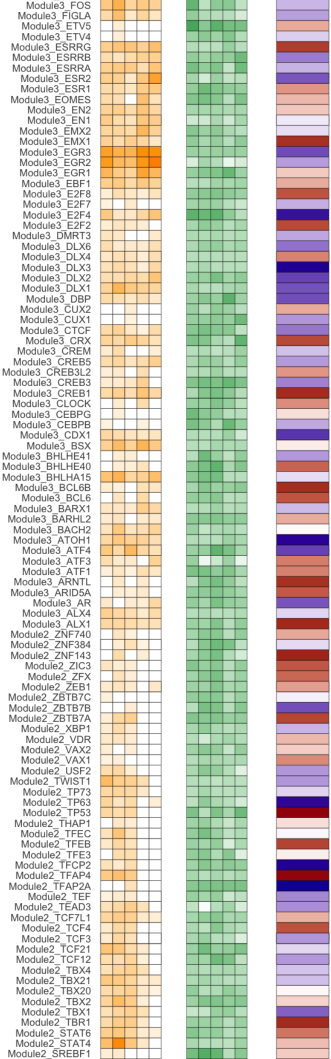
Module3_FOS
Module3_FIGLA
Module3_ETV5
Module3_ETV4
Module3_ESRRG
Module3_ESRRB
Module3_ESRRA
Module3_ESR2
Module3_ESR1
Module3_EOMES
Module3_EN2
Module3_EN1
Module3_EMX2
Module3_EMX1
Module3_EGR3
Module3_EGR2
Module3_EGR1
Module3_EBF1
Module3_E2F8
Module3_E2F7
Module3_E2F4
Module3_E2F2
Module3_DMRT3
Module3_DLX6
Module3_DLX4
Module3_DLX3
Module3_DLX2
Module3_DLX1
Module3_DBP
Module3_CUX2
Module3_CUX1
Module3_CTCF
Module3_CRX
Module3_CREM
Module3_CREB5
Module3_CREB3L2
Module3_CREB3
Module3_CREB1
Module3_CLOCK
Module3_CEBPG
Module3_CEBPB
Module3_CDX1
Module3_BSX
Module3_BHLHE41
Module3_BHLHE40
Module3_BHLHA15
Module3_BCL6B
Module3_BCL6
Module3_BARX1
Module3_BARHL2
Module3_BACH2
Module3_ATOH1
Module3_ATF4
Module3_ATF3
Module3_ATF1
Module3_ARNTL
Module3_ARID5A
Module3_AR
Module3_ALX4
Module3_ALX1
Module2_ZNF740
Module2_ZNF384
Module2_ZNF143
Module2_ZIC3
Module2_ZFX
Module2_ZEB1
Module2_ZBTB7C
Module2_ZBTB7B
Module2_ZBTB7A
Module2_XBP1
Module2_VDR
Module2_VAX2
Module2_VAX1
Module2_USF2
Module2_TWIST1
Module2_TP73
Module2_TP63
Module2_TP53
Module2_THAP1
Module2_TFEC
Module2_TFEB
Module2_TFE3
Module2_TFCP2
Module2_TFAP4
Module2_TFAP2A
Module2_TEF
Module2_TEAD3
Module2_TCF7L1
Module2_TCF4
Module2_TCF3
Module2_TCF21
Module2_TCF12
Module2_TBX4
Module2_TBX21
Module2_TBX20
Module2_TBX2
Module2_TBX1
Module2_TBR1
Module2_STAT6
Module2_STAT4
Module2_SREBF1

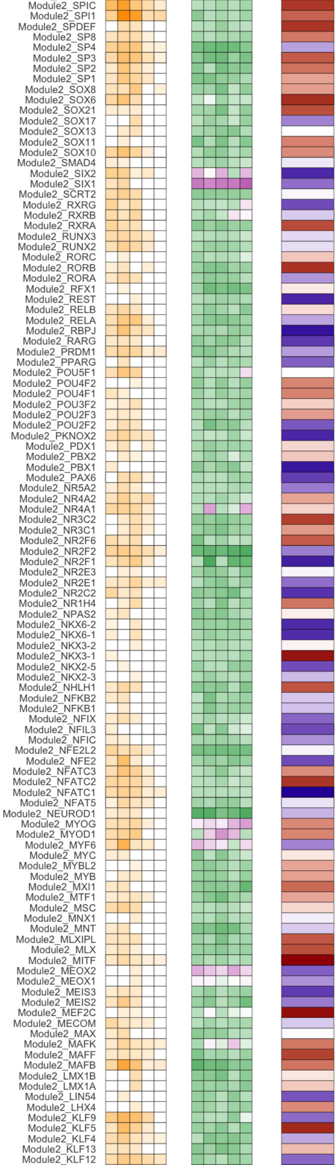
Module2_SPIC
Module2_SPI1
Module2_SPDEF
Module2_SP8
Module2_SP4
Module2_SP3
Module2_SP2
Module2_SP1
Module2_SOX8
Module2_SOX6
Module2_SOX21
Module2_SOX17
Module2_SOX13
Module2_SOX11
Module2_SOX10
Module2_SMAD4
Module2_SIX2
Module2_SIX1
Module2_SCRT2
Module2_RXRG
Module2_RXRB
Module2_RXRA
Module2_RUNX3
Module2_RUNX2
Module2_RORC
Module2_RORB
Module2_RORA
Module2_RFX1
Module2_REST
Module2_RELB
Module2_RELA
Module2_RBPJ
Module2_RARG
Module2_PRDM1
Module2_PPARG
Module2_POU5F1
Module2_POU4F2
Module2_POU4F1
Module2_POU3F2
Module2_POU2F3
Module2_POU2F2
Module2_PKNOX2
Module2_PDX1
Module2_PBX2
Module2_PBX1
Module2_PAX6
Module2_NR5A2
Module2_NR4A2
Module2_NR4A1
Module2_NR3C2
Module2_NR3C1
Module2_NR2F6
Module2_NR2F2
Module2_NR2F1
Module2_NR2E3
Module2_NR2E1
Module2_NR2C2
Module2_NR1H4
Module2_NPAS2
Module2_NKX6-2
Module2_NKX6-1
Module2_NKX3-2
Module2_NKX3-1
Module2_NKX2-5
Module2_NKX2-3
Module2_NHLH1
Module2_NFKB2
Module2_NFKB1
Module2_NFIX
Module2_NFIL3
Module2_NFIC
Module2_NFE2L2
Module2_NFE2
Module2_NFATC3
Module2_NFATC2
Module2_NFATC1
Module2_NFAT5
Module2_NEUROD1
Module2_MYOG
Module2_MYOD1
Module2_MYF6
Module2_MYC
Module2_MYBL2
Module2_MYB
Module2_MXI1
Module2_MTF1
Module2_MSC
Module2_MNX1
Module2_MNT
Module2_MLXIPL
Module2_MLX
Module2_MITF
Module2_MEOX2
Module2_MEOX1
Module2_MEIS3
Module2_MEIS2
Module2_MEF2C
Module2_MECOM
Module2_MAX
Module2_MAFK
Module2_MAFF
Module2_MAFB
Module2_LMX1B
Module2_LMX1A
Module2_LIN54
Module2_LHX4
Module2_KLF9
Module2_KLF5
Module2_KLF4
Module2_KLF13
Module2_KLF12

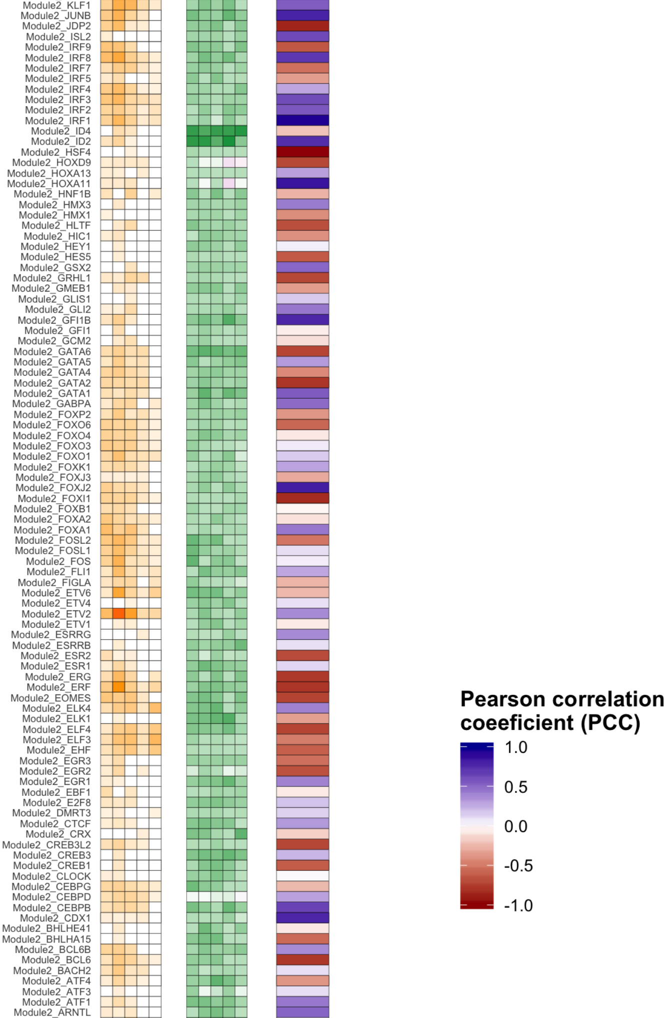
Module2_KLF1
Module2_JUNB
Module2_JDP2
Module2_ISL2
Module2_IRF9
Module2_IRF8
Module2_IRF7
Module2_IRF5
Module2_IRF4
Module2_IRF3
Module2_IRF2
Module2_IRF1
Module2_ID4
Module2_ID2
Module2_HSF4
Module2_HOXD9
Module2_HOXA13
Module2_HOXA11
Module2_HNF1B
Module2_HMX3
Module2_HMX1
Module2_HLTF
Module2_HIC1
Module2_HEY1
Module2_HES5
Module2_GSX2
Module2_GRHL1
Module2_GMEB1
Module2_GLIS1
Module2_GLI2
Module2_GFI1B
Module2_GFI1
Module2_GCM2
Module2_GATA6
Module2_GATA5
Module2_GATA4
Module2_GATA2
Module2_GATA1
Module2_GABPA
Module2_FOXP2
Module2_FOXO6
Module2_FOXO4
Module2_FOXO3
Module2_FOXO1
Module2_FOXK1
Module2_FOXJ3
Module2_FOXJ2
Module2_FOXI1
Module2_FOXB1
Module2_FOXA2
Module2_FOXA1
Module2_FOSL2
Module2_FOSL1
Module2_FOS
Module2_FLI1
Module2_FIGLA
Module2_ETV6
Module2_ETV4
Module2_ETV2
Module2_ETV1
Module2_ESRRG
Module2_ESRRB
Module2_ESR2
Module2_ESR1
Module2_ERG
Module2_ERF
Module2_EOMES
Module2_ELK4
Module2_ELK1
Module2_ELF4
Module2_ELF3
Module2_EHF
Module2_EGR3
Module2_EGR2
Module2_EGR1
Module2_EBF1
Module2_E2F8
Module2_DMRT3
Module2_CTCF
Module2_CRX
Module2_CREB3L2
Module2_CREB3
Module2_CREB1
Module2_CLOCK
Module2_CEBPG
Module2_CEBPD
Module2_CEBPB
Module2_CDX1
Module2_BHLHE41
Module2_BHLHA15
Module2_BCL6B
Module2_BCL6
Module2_BACH2
Module2_ATF4
Module2_ATF3
Module2_ATF1
Module2_ARNTL
Pearson correlation coeeficient (PCC)
1.0
0.5
0.0
-0.5
-1.0

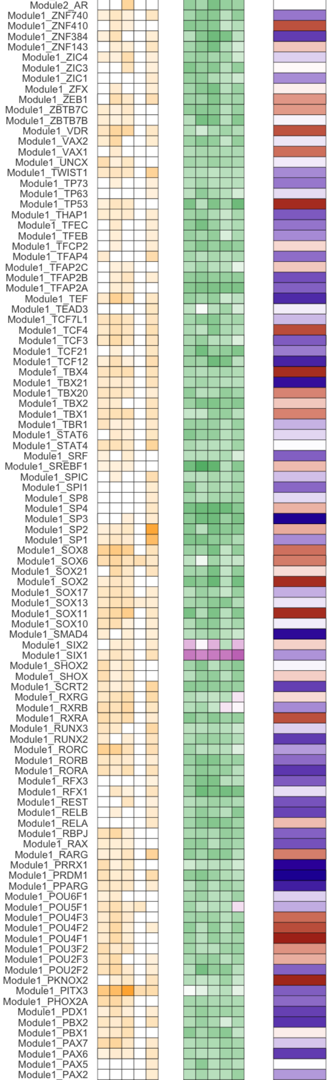
Module2_AR
Module1_ZNF740
Module1_ZNF410
Module1_ZNF384
Module1_ZNF143
Module1_ZIC4
Module1_ZIC3
Module1_ZIC1
Module1_ZFX
Module1_ZEB1
Module1_ZBTB7C
Module1_ZBTB7B
Module1_VDR
Module1_VAX2
Module1_VAX1
Module1_UNCX
Module1_TWIST1
Module1_TP73
Module1_TP63
Module1_TP53
Module1_THAP1
Module1_TFEC
Module1_TFEB
Module1_TFCP2
Module1_TFAP4
Module1_TFAP2C
Module1_TFAP2B
Module1_TFAP2A
Module1_TEF
Module1_TEAD3
Module1_TCF7L1
Module1_TCF4
Module1_TCF3
Module1_TCF21
Module1_TCF12
Module1_TBX4
Module1_TBX21
Module1_TBX20
Module1_TBX2
Module1_TBX1
Module1_TBR1
Module1_STAT6
Module1_STAT4
Module1_SRF
Module1_SREBF1
Module1_SPIC
Module1_SPI1
Module1_SP8
Module1_SP4
Module1_SP3
Module1_SP2
Module1_SP1
Module1_SOX8
Module1_SOX6
Module1_SOX21
Module1_SOX2
Module1_SOX17
Module1_SOX13
Module1_SOX11
Module1_SOX10
Module1_SMAD4
Module1_SIX2
Module1_SIX1
Module1_SHOX2
Module1_SHOX
Module1_SCRT2
Module1_RXRG
Module1_RXRB
Module1_RXRA
Module1_RUNX3
Module1_RUNX2
Module1_RORC
Module1_RORB
Module1_RORA
Module1_RFX3
Module1_RFX1
Module1_REST
Module1_RELB
Module1_RELA
Module1_RBPJ
Module1_RAX
Module1_RARG
Module1_PRRX1
Module1_PRDM1
Module1_PPARG
Module1_POU6F1
Module1_POU5F1
Module1_POU4F3
Module1_POU4F2
Module1_POU4F1
Module1_POU3F2
Module1_POU2F3
Module1_POU2F2
Module1_PKNOX2
Module1_PITX3
Module1_PHOX2A
Module1_PDX1
Module1_PBX2
Module1_PBX1
Module1_PAX7
Module1_PAX6
Module1_PAX5
Module1_PAX2

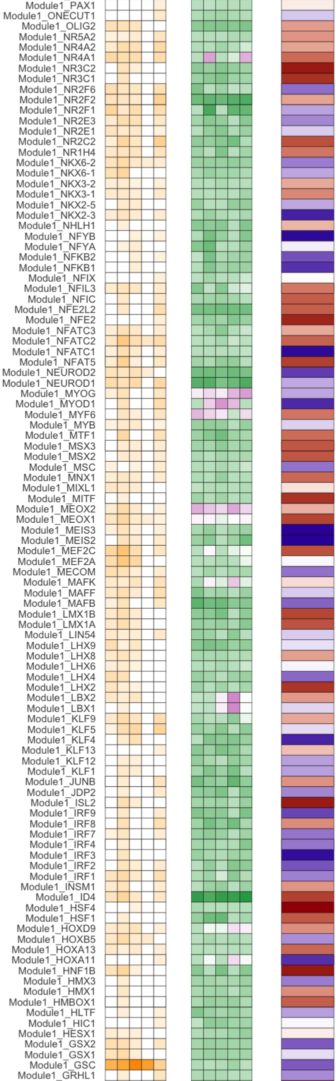
Module1_PAX1
Module1_ONECUT1
Module1_OLIG2
Module1_NR5A2
Module1_NR4A2
Module1_NR4A1
Module1_NR3C2
Module1_NR3C1
Module1_NR2F6
Module1_NR2F2
Module1_NR2F1
Module1_NR2E3
Module1_NR2E1
Module1_NR2C2
Module1_NR1H4
Module1_NKX6-2
Module1_NKX6-1
Module1_NKX3-2
Module1_NKX3-1
Module1_NKX2-5
Module1_NKX2-3
Module1_NHLH1
Module1_NFYB
Module1_NFYA
Module1_NFKB2
Module1_NFKB1
Module1_NFIX
Module1_NFIL3
Module1_NFIC
Module1_NFE2L2
Module1_NFE2
Module1_NFATC3
Module1_NFATC2
Module1_NFATC1
Module1_NFAT5
Module1_NEUROD2
Module1_NEUROD1
Module1_MYOG
Module1_MYOD1
Module1_MYF6
Module1_MYB
Module1_MTF1
Module1_MSX3
Module1_MSX2
Module1_MSC
Module1_MNX1
Module1_MIXL1
Module1_MITF
Module1_MEOX2
Module1_MEOX1
Module1_MEIS3
Module1_MEIS2
Module1_MEF2C
Module1_MEF2A
Module1_MECOM
Module1_MAFK
Module1_MAFF
Module1_MAFB
Module1_LMX1B
Module1_LMX1A
Module1_LIN54
Module1_LHX9
Module1_LHX8
Module1_LHX6
Module1_LHX4
Module1_LHX2
Module1_LBX2
Module1_LBX1
Module1_KLF9
Module1_KLF5
Module1_KLF4
Module1_KLF13
Module1_KLF12
Module1_KLF1
Module1_JUNB
Module1_JDP2
Module1_ISL2
Module1_IRF9
Module1_IRF8
Module1_IRF7
Module1_IRF4
Module1_IRF3
Module1_IRF2
Module1_IRF1
Module1_INSM1
Module1_ID4
Module1_HSF4
Module1_HSF1
Module1_HOXD9
Module1_HOXB5
Module1_HOXA13
Module1_HOXA11
Module1_HNF1B
Module1_HMX3
Module1_HMX1
Module1_HMBOX1
Module1_HLTF
Module1_HIC1
Module1_HESX1
Module1_GSX2
Module1_GSX1
Module1_GSC
Module1_GRHL1

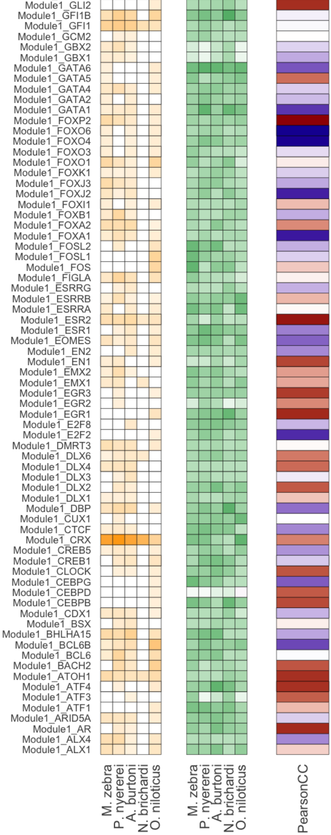
Module1_GLI2
Module1_GFI1B
Module1_GFI1
Module1_GCM2
Module1_GBX2
Module1_GBX1
Module1_GATA6
Module1_GATA5
Module1_GATA4
Module1_GATA2
Module1_GATA1
Module1_FOXP2
Module1_FOXO6
Module1_FOXO4
Module1_FOXO3
Module1_FOXO1
Module1_FOXK1
Module1_FOXJ3
Module1_FOXJ2
Module1_FOXI1
Module1_FOXB1
Module1_FOXA2
Module1_FOXA1
Module1_FOSL2
Module1_FOSL1
Module1_FOS
Module1_FIGLA
Module1_ESRRG
Module1_ESRRB
Module1_ESRRA
Module1_ESR2
Module1_ESR1
Module1_EOMES
Module1_EN2
Module1_EN1
Module1_EMX2
Module1_EMX1
Module1_EGR3
Module1_EGR2
Module1_EGR1
Module1_E2F8
Module1_E2F2
Module1_DMRT3
Module1_DLX6
Module1_DLX4
Module1_DLX3
Module1_DLX2
Module1_DLX1
Module1_DBP
Module1_CUX1
Module1_CTCF
Module1_CRX
Module1_CREB5
Module1_CREB1
Module1_CLOCK
Module1_CEBPG
Module1_CEBPD
Module1_CEBPB
Module1_CDX1
Module1_BSX
Module1_BHLHA15
Module1_BCL6B
Module1_BCL6
Module1_BACH2
Module1_ATOH1
Module1_ATF4
Module1_ATF3
Module1_ATF1
Module1_ARID5A
Module1_AR
Module1_ALX4
Module1_ALX1
M. zebra
P. nyererei
A. burtoni
N. brichardi
O. niloticus
M. zebra
P. nyererei
A. burtoni
N. brichardi
O. niloticus
PearsonCC

Additional file 2: Fig. S8

Heatmap matrices of enrichment, expression and Pearson correlation between the two for each motif, across testis tissue

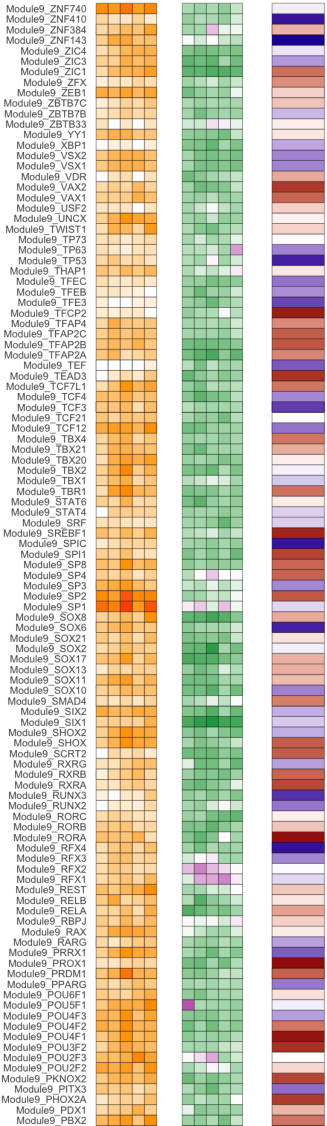

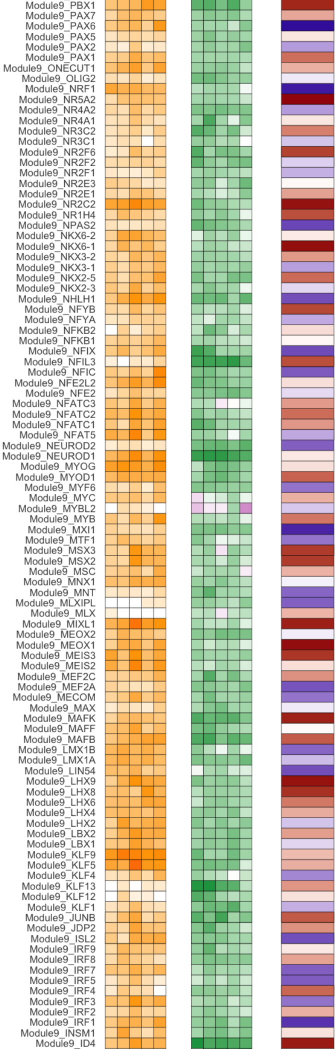
Module9_PBX1
Module9_PAX7
Module9_PAX6
Module9_PAX5
Module9_PAX2
Module9_PAX1
Module9_ONECUT1
Module9_OLIG2
Module9_NRF1
Module9_NR5A2
Module9_NR4A2
Module9_NR4A1
Module9_NR3C2
Module9_NR3C1
Module9_NR2F6
Module9_NR2F2
Module9_NR2F1
Module9_NR2E3
Module9_NR2E1
Module9_NR2C2
Module9_NR1H4
Module9_NPAS2
Module9_NKX6-2
Module9_NKX6-1
Module9_NKX3-2
Module9_NKX3-1
Module9_NKX2-5
Module9_NKX2-3
Module9_NHLH1
Module9_NFYB
Module9_NFYA
Module9_NFKB2
Module9_NFKB1
Module9_NFIX
Module9_NFIL3
Module9_NFIC
Module9_NFE2L2
Module9_NFE2
Module9_NFATC3
Module9_NFATC2
Module9_NFATC1
Module9_NFAT5
Module9_NEUROD2
Module9_NEUROD1
Module9_MYOG
Module9_MYOD1
Module9_MYF6
Module9_MYC
Module9_MYBL2
Module9_MYB
Module9_MXI1
Module9_MTF1
Module9_MSX3
Module9_MSX2
Module9_MSC
Module9_MNX1
Module9_MNT
Module9_MLXIPL
Module9_MLX
Module9_MIXL1
Module9_MEOX2
Module9_MEOX1
Module9_MEIS3
Module9_MEIS2
Module9_MEF2C
Module9_MEF2A
Module9_MECOM
Module9_MAX
Module9_MAFK
Module9_MAFF
Module9_MAFB
Module9_LMX1B
Module9_LMX1A
Module9_LIN54
Module9_LHX9
Module9_LHX8
Module9_LHX6
Module9_LHX4
Module9_LHX2
Module9_LBX2
Module9_LBX1
Module9_KLF9
Module9_KLF5
Module9_KLF4
Module9_KLF13
Module9_KLF12
Module9_KLF1
Module9_JUNB
Module9_JDP2
Module9_ISL2
Module9_IRF9
Module9_IRF8
Module9_IRF7
Module9_IRF5
Module9_IRF4
Module9_IRF3
Module9_IRF2
Module9_IRF1
Module9_INSM1
Module9_ID4

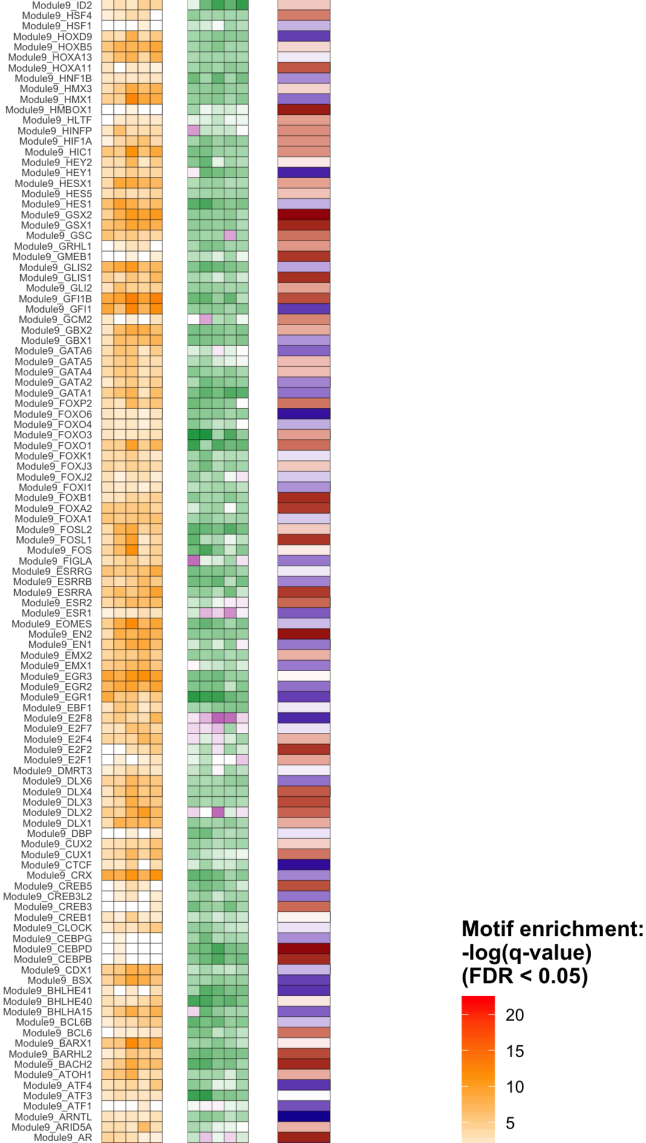
Module9_ID2
Module9_HSF4
Module9_HSF1
Module9_HOXD9
Module9_HOXB5
Module9_HOXA13
Module9_HOXA11
Module9_HNF1B
Module9_HMX3
Module9_HMX1
Module9_HMBOX1
Module9_HLTF
Module9_HINFP
Module9_HIF1A
Module9_HIC1
Module9_HEY2
Module9_HEY1
Module9_HESX1
Module9_HES5
Module9_HES1
Module9_GSX2
Module9_GSX1
Module9_GSC
Module9_GRHL1
Module9_GMEB1
Module9_GLIS2
Module9_GLIS1
Module9_GLI2
Module9_GFI1B
Module9_GFI1
Module9_GCM2
Module9_GBX2
Module9_GBX1
Module9_GATA6
Module9_GATA5
Module9_GATA4
Module9_GATA2
Module9_GATA1
Module9_FOXP2
Module9_FOXO6
Module9_FOXO4
Module9_FOXO3
Module9_FOXO1
Module9_FOXK1
Module9_FOXJ3
Module9_FOXJ2
Module9_FOXI1
Module9_FOXB1
Module9_FOXA2
Module9_FOXA1
Module9_FOSL2
Module9_FOSL1
Module9_FOS
Module9_FIGLA
Module9_ESRRG
Module9_ESRRB
Module9_ESRRA
Module9_ESR2
Module9_ESR1
Module9_EOMES
Module9_EN2
Module9_EN1
Module9_EMX2
Module9_EMX1
Module9_EGR3
Module9_EGR2
Module9_EGR1
Module9_EBF1
Module9_E2F8
Module9_E2F7
Module9_E2F4
Module9_E2F2
Module9_E2F1
Module9_DMRT3
Module9_DLX6
Module9_DLX4
Module9_DLX3
Module9_DLX2
Module9_DLX1
Module9_DBP
Module9_CUX2
Module9_CUX1
Module9_CTCF
Module9_CRX
Module9_CREB5
Module9_CREB3L2
Module9_CREB3
Module9_CREB1
Module9_CLOCK
Module9_CEBPG
Module9_CEBPD
Module9_CEBPB
Module9_CDX1
Module9_BSX
Module9_BHLHE41
Module9_BHLHE40
Module9_BHLHA15
Module9_BCL6B
Module9_BCL6
Module9_BARX1
Module9_BARHL2
Module9_BACH2
Module9_ATOH1
Module9_ATF4
Module9_ATF3
Module9_ATF1
Module9_ARNTL
Module9_ARID5A
Module9_AR
Motif enrichment:
-log(q-value)
(FDR < 0.05)
20
15
10
5

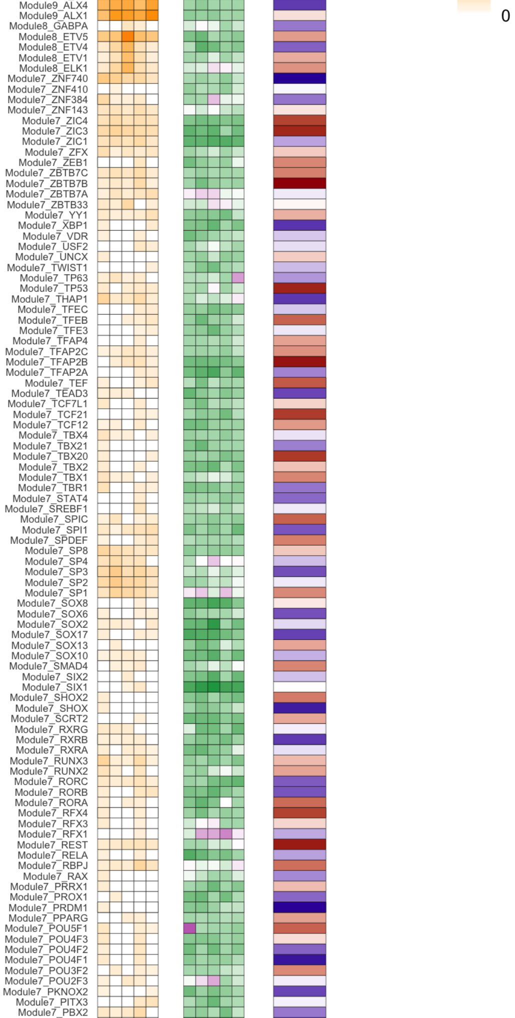
0
Module9_ALX4
Module9_ALX1
Module8_GABPA
Module8_ETV5
Module8_ETV4
Module8_ETV1
Module8_ELK1
Module7_ZNF740
Module7_ZNF410
Module7_ZNF384
Module7_ZNF143
Module7_ZIC4
Module7_ZIC3
Module7_ZIC1
Module7_ZFX
Module7_ZEB1
Module7_ZBTB7C
Module7_ZBTB7B
Module7_ZBTB7A
Module7_ZBTB33
Module7_YY1
Module7_XBP1
Module7_VDR
Module7_USF2
Module7_UNCX
Module7_TWIST1
Module7_TP63
Module7_TP53
Module7_THAP1
Module7_TFEC
Module7_TFEB
Module7_TFE3
Module7_TFAP4
Module7_TFAP2C
Module7_TFAP2B
Module7_TFAP2A
Module7_TEF
Module7_TEAD3
Module7_TCF7L1
Module7_TCF21
Module7_TCF12
Module7_TBX4
Module7_TBX21
Module7_TBX20
Module7_TBX2
Module7_TBX1
Module7_TBR1
Module7_STAT4
Module7_SREBF1
Module7_SPIC
Module7_SPI1
Module7_SPDEF
Module7_SP8
Module7_SP4
Module7_SP3
Module7_SP2
Module7_SP1
Module7_SOX8
Module7_SOX6
Module7_SOX2
Module7_SOX17
Module7_SOX13
Module7_SOX10
Module7_SMAD4
Module7_SIX2
Module7_SIX1
Module7_SHOX2
Module7_SHOX
Module7_SCRT2
Module7_RXRG
Module7_RXRB
Module7_RXRA
Module7_RUNX3
Module7_RUNX2
Module7_RORC
Module7_RORB
Module7_RORA
Module7_RFX4
Module7_RFX3
Module7_RFX1
Module7_REST
Module7_RELA
Module7_RBPJ
Module7_RAX
Module7_PRRX1
Module7_PROX1
Module7_PRDM1
Module7_PPARG
Module7_POU5F1
Module7_POU4F3
Module7_POU4F2
Module7_POU4F1
Module7_POU3F2
Module7_POU2F3
Module7_PKNOX2
Module7_PITX3
Module7_PBX2

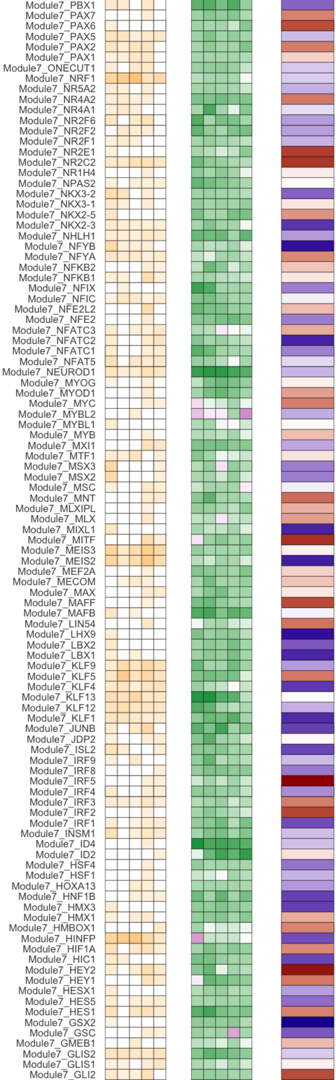
Module7_PBX1
Module7_PAX7
Module7_PAX6
Module7_PAX5
Module7_PAX2
Module7_PAX1
Module7_ONECUT1
Module7_NRF1
Module7_NR5A2
Module7_NR4A2
Module7_NR4A1
Module7_NR2F6
Module7_NR2F2
Module7_NR2F1
Module7_NR2E1
Module7_NR2C2
Module7_NR1H4
Module7_NPAS2
Module7_NKX3-2
Module7_NKX3-1
Module7_NKX2-5
Module7_NKX2-3
Module7_NHLH1
Module7_NFYB
Module7_NFYA
Module7_NFKB2
Module7_NFKB1
Module7_NFIX
Module7_NFIC
Module7_NFE2L2
Module7_NFE2
Module7_NFATC3
Module7_NFATC2
Module7_NFATC1
Module7_NFAT5
Module7_NEUROD1
Module7_MYOG
Module7_MYOD1
Module7_MYC
Module7_MYBL2
Module7_MYBL1
Module7_MYB
Module7_MXI1
Module7_MTF1
Module7_MSX3
Module7_MSX2
Module7_MSC
Module7_MNT
Module7_MLXIPL
Module7_MLX
Module7_MIXL1
Module7_MITF
Module7_MEIS3
Module7_MEIS2
Module7_MEF2A
Module7_MECOM
Module7_MAX
Module7_MAFF
Module7_MAFB
Module7_LIN54
Module7_LHX9
Module7_LBX2
Module7_LBX1
Module7_KLF9
Module7_KLF5
Module7_KLF4
Module7_KLF13
Module7_KLF12
Module7_KLF1
Module7_JUNB
Module7_JDP2
Module7_ISL2
Module7_IRF9
Module7_IRF8
Module7_IRF5
Module7_IRF4
Module7_IRF3
Module7_IRF2
Module7_IRF1
Module7_INSM1
Module7_ID4
Module7_ID2
Module7_HSF4
Module7_HSF1
Module7_HOXA13
Module7_HNF1B
Module7_HMX3
Module7_HMX1
Module7_HMBOX1
Module7_HINFP
Module7_HIF1A
Module7_HIC1
Module7_HEY2
Module7_HEY1
Module7_HESX1
Module7_HES5
Module7_HES1
Module7_GSX2
Module7_GSC
Module7_GMEB1
Module7_GLIS2
Module7_GLIS1
Module7_GLI2

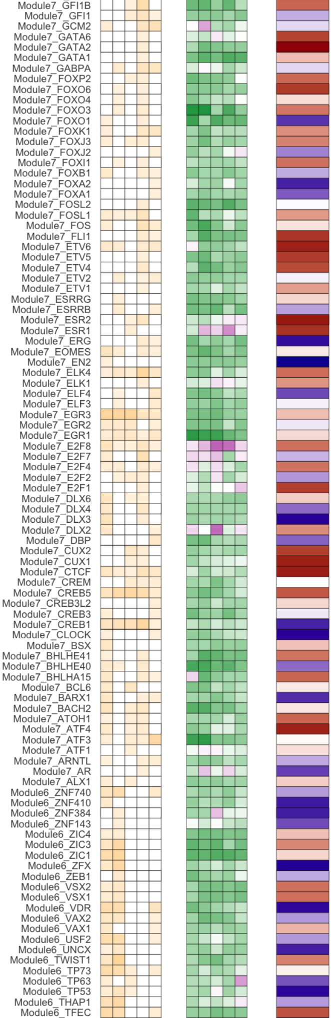
Module7_GFI1B
Module7_GFI1
Module7_GCM2
Module7_GATA6
Module7_GATA2
Module7_GATA1
Module7_GABPA
Module7_FOXP2
Module7_FOXO6
Module7_FOXO4
Module7_FOXO3
Module7_FOXO1
Module7_FOXK1
Module7_FOXJ3
Module7_FOXJ2
Module7_FOXI1
Module7_FOXB1
Module7_FOXA2
Module7_FOXA1
Module7_FOSL2
Module7_FOSL1
Module7_FOS
Module7_FLI1
Module7_ETV6
Module7_ETV5
Module7_ETV4
Module7_ETV2
Module7_ETV1
Module7_ESRRG
Module7_ESRRB
Module7_ESR2
Module7_ESR1
Module7_ERG
Module7_EOMES
Module7_EN2
Module7_ELK4
Module7_ELK1
Module7_ELF4
Module7_ELF3
Module7_EGR3
Module7_EGR2
Module7_EGR1
Module7_E2F8
Module7_E2F7
Module7_E2F4
Module7_E2F2
Module7_E2F1
Module7_DLX6
Module7_DLX4
Module7_DLX3
Module7_DLX2
Module7_DBP
Module7_CUX2
Module7_CUX1
Module7_CTCF
Module7_CREM
Module7_CREB5
Module7_CREB3L2
Module7_CREB3
Module7_CREB1
Module7_CLOCK
Module7_BSX
Module7_BHLHE41
Module7_BHLHE40
Module7_BHLHA15
Module7_BCL6
Module7_BARX1
Module7_BACH2
Module7_ATOH1
Module7_ATF4
Module7_ATF3
Module7_ATF1
Module7_ARNTL
Module7_AR
Module7_ALX1
Module6_ZNF740
Module6_ZNF410
Module6_ZNF384
Module6_ZNF143
Module6_ZIC4
Module6_ZIC3
Module6_ZIC1
Module6_ZFX
Module6_ZEB1
Module6_VSX2
Module6_VSX1
Module6_VDR
Module6_VAX2
Module6_VAX1
Module6_USF2
Module6_UNCX
Module6_TWIST1
Module6_TP73
Module6_TP63
Module6_TP53
Module6_THAP1
Module6_TFEC

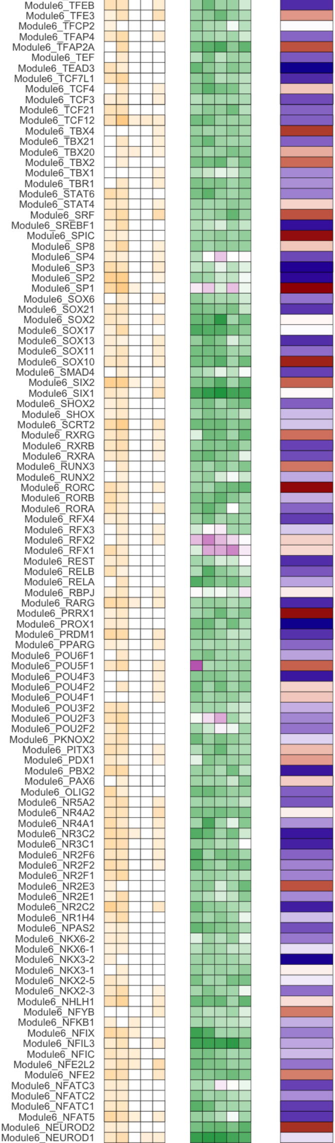
Module6_TFEB
Module6_TFE3
Module6_TFCP2
Module6_TFAP4
Module6_TFAP2A
Module6_TEF
Module6_TEAD3
Module6_TCF7L1
Module6_TCF4
Module6_TCF3
Module6_TCF21
Module6_TCF12
Module6_TBX4
Module6_TBX21
Module6_TBX20
Module6_TBX2
Module6_TBX1
Module6_TBR1
Module6_STAT6
Module6_STAT4
Module6_SRF
Module6_SREBF1
Module6_SPIC
Module6_SP8
Module6_SP4
Module6_SP3
Module6_SP2
Module6_SP1
Module6_SOX6
Module6_SOX21
Module6_SOX2
Module6_SOX17
Module6_SOX13
Module6_SOX11
Module6_SOX10
Module6_SMAD4
Module6_SIX2
Module6_SIX1
Module6_SHOX2
Module6_SHOX
Module6_SCRT2
Module6_RXRG
Module6_RXRB
Module6_RXRA
Module6_RUNX3
Module6_RUNX2
Module6_RORC
Module6_RORB
Module6_RORA
Module6_RFX4
Module6_RFX3
Module6_RFX2
Module6_RFX1
Module6_REST
Module6_RELB
Module6_RELA
Module6_RBPJ
Module6_RARG
Module6_PRRX1
Module6_PROX1
Module6_PRDM1
Module6_PPARG
Module6_POU6F1
Module6_POU5F1
Module6_POU4F3
Module6_POU4F2
Module6_POU4F1
Module6_POU3F2
Module6_POU2F3
Module6_POU2F2
Module6_PKNOX2
Module6_PITX3
Module6_PDX1
Module6_PBX2
Module6_PAX6
Module6_OLIG2
Module6_NR5A2
Module6_NR4A2
Module6_NR4A1
Module6_NR3C2
Module6_NR3C1
Module6_NR2F6
Module6_NR2F2
Module6_NR2F1
Module6_NR2E3
Module6_NR2E1
Module6_NR2C2
Module6_NR1H4
Module6_NPAS2
Module6_NKX6-2
Module6_NKX6-1
Module6_NKX3-2
Module6_NKX3-1
Module6_NKX2-5
Module6_NKX2-3
Module6_NHLH1
Module6_NFYB
Module6_NFKB1
Module6_NFIX
Module6_NFIL3
Module6_NFIC
Module6_NFE2L2
Module6_NFE2
Module6_NFATC3
Module6_NFATC2
Module6_NFATC1
Module6_NFAT5
Module6_NEUROD2
Module6_NEUROD1

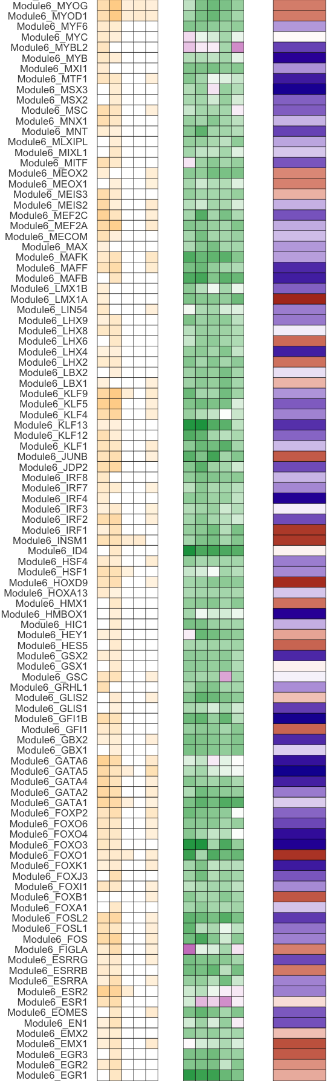
Module6_MYOG
Module6_MYOD1
Module6_MYF6
Module6_MYC
Module6_MYBL2
Module6_MYB
Module6_MXI1
Module6_MTF1
Module6_MSX3
Module6_MSX2
Module6_MSC
Module6_MNX1
Module6_MNT
Module6_MLXIPL
Module6_MIXL1
Module6_MITF
Module6_MEOX2
Module6_MEOX1
Module6_MEIS3
Module6_MEIS2
Module6_MEF2C
Module6_MEF2A
Module6_MECOM
Module6_MAX
Module6_MAFK
Module6_MAFF
Module6_MAFB
Module6_LMX1B
Module6_LMX1A
Module6_LIN54
Module6_LHX9
Module6_LHX8
Module6_LHX6
Module6_LHX4
Module6_LHX2
Module6_LBX2
Module6_LBX1
Module6_KLF9
Module6_KLF5
Module6_KLF4
Module6_KLF13
Module6_KLF12
Module6_KLF1
Module6_JUNB
Module6_JDP2
Module6_IRF8
Module6_IRF7
Module6_IRF4
Module6_IRF3
Module6_IRF2
Module6_IRF1
Module6_INSM1
Module6_ID4
Module6_HSF4
Module6_HSF1
Module6_HOXD9
Module6_HOXA13
Module6_HMX1
Module6_HMBOX1
Module6_HIC1
Module6_HEY1
Module6_HES5
Module6_GSX2
Module6_GSX1
Module6_GSC
Module6_GRHL1
Module6_GLIS2
Module6_GLIS1
Module6_GFI1B
Module6_GFI1
Module6_GBX2
Module6_GBX1
Module6_GATA6
Module6_GATA5
Module6_GATA4
Module6_GATA2
Module6_GATA1
Module6_FOXP2
Module6_FOXO6
Module6_FOXO4
Module6_FOXO3
Module6_FOXO1
Module6_FOXK1
Module6_FOXJ3
Module6_FOXI1
Module6_FOXB1
Module6_FOXA1
Module6_FOSL2
Module6_FOSL1
Module6_FOS
Module6_FIGLA
Module6_ESRRG
Module6_ESRRB
Module6_ESRRA
Module6_ESR2
Module6_ESR1
Module6_EOMES
Module6_EN1
Module6_EMX2
Module6_EMX1
Module6_EGR3
Module6_EGR2
Module6_EGR1

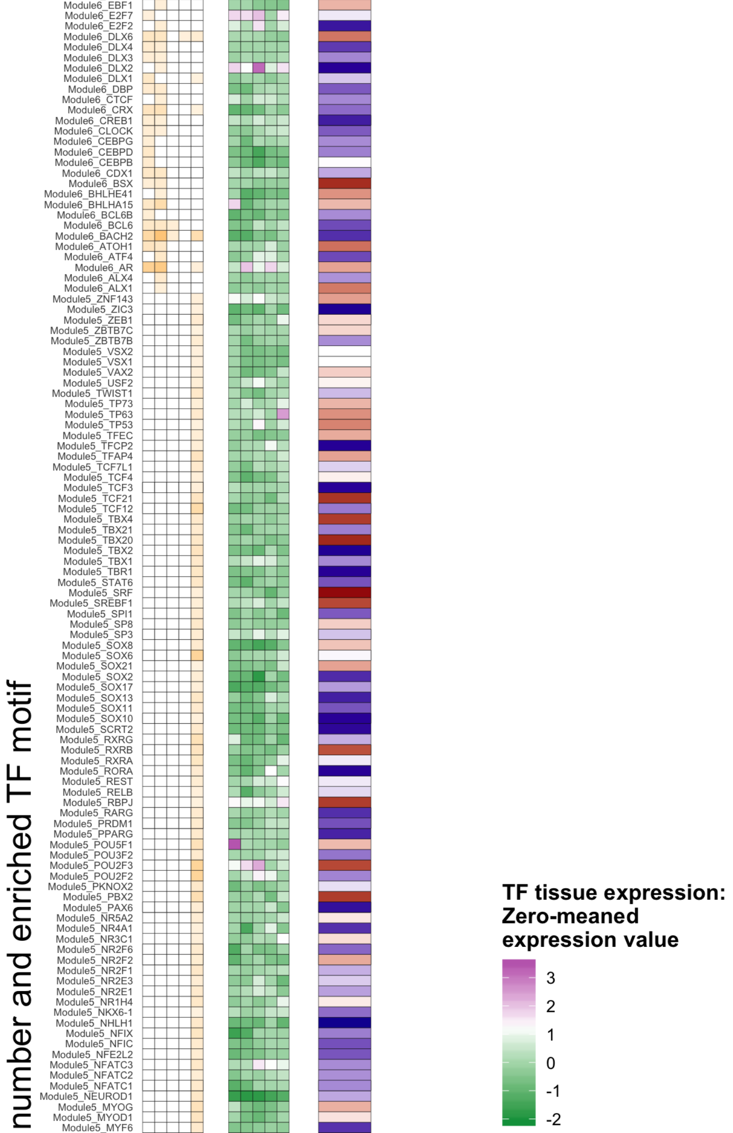

Module6_EBF1
Module6_E2F7
Module6_E2F2
Module6_DLX6
Module6_DLX4
Module6_DLX3
Module6_DLX2
Module6_DLX1
Module6_DBP
Module6_CTCF
Module6_CRX
Module6_CREB1
Module6_CLOCK
Module6_CEBPG
Module6_CEBPD
Module6_CEBPB
Module6_CDX1
Module6_BSX
Module6_BHLHE41
Module6_BHLHA15
Module6_BCL6B
Module6_BCL6
Module6_BACH2
Module6_ATOH1
Module6_ATF4
Module6_AR
Module6_ALX4
Module6_ALX1
Module5_ZNF143
Module5_ZIC3
Module5_ZEB1
Module5_ZBTB7C
Module5_ZBTB7B
Module5_VSX2
Module5_VSX1
Module5_VAX2
Module5_USF2
Module5_TWIST1
Module5_TP73
Module5_TP63
Module5_TP53
Module5_TFEC
Module5_TFCP2
Module5_TFAP4
Module5_TCF7L1
Module5_TCF4
Module5_TCF3
Module5_TCF21
Module5_TCF12
Module5_TBX4
Module5_TBX21
Module5_TBX20
Module5_TBX2
Module5_TBX1
Module5_TBR1
Module5_STAT6
Module5_SRF
Module5_SREBF1
Module5_SPI1
Module5_SP8
Module5_SP3
Module5_SOX8
Module5_SOX6
Module5_SOX21
Module5_SOX2
Module5_SOX17
Module5_SOX13
Module5_SOX11
Module5_SOX10
Module5_SCRT2
Module5_RXRG
Module5_RXRB
Module5_RXRA
Module5_RORA
Module5_REST
Module5_RELB
Module5_RBPJ
Module5_RARG
Module5_PRDM1
Module5_PPARG
Module5_POU5F1
Module5_POU3F2
Module5_POU2F3
Module5_POU2F2
Module5_PKNOX2
Module5_PBX2
Module5_PAX6
Module5_NR5A2
Module5_NR4A1
Module5_NR3C1
Module5_NR2F6
Module5_NR2F2
Module5_NR2F1
Module5_NR2E3
Module5_NR2E1
Module5_NR1H4
Module5_NKX6-1
Module5_NHLH1
Module5_NFIX
Module5_NFIC
Module5_NFE2L2
Module5_NFATC3
Module5_NFATC2
Module5_NFATC1
Module5_NEUROD1
Module5_MYOG
Module5_MYOD1
Module5_MYF6
number and enriched TF motif
TF tissue expression:
Zero-meaned
expression value
3
2
1
0
-1
-2

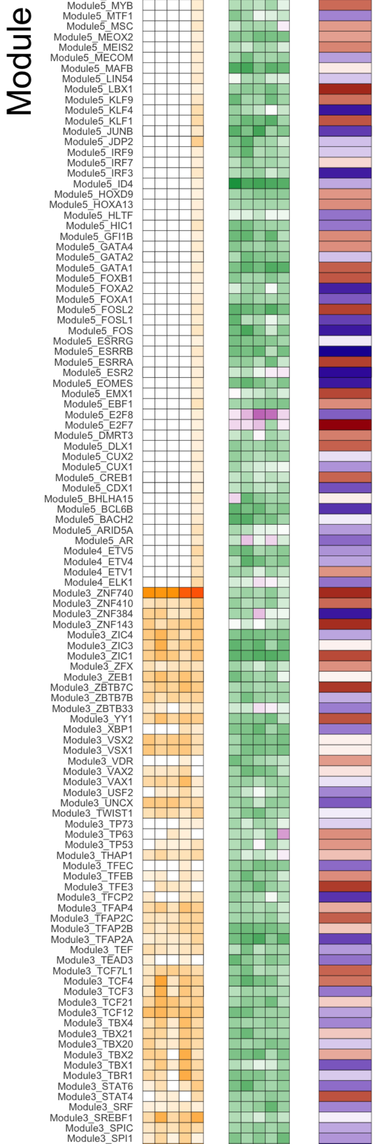
Module
Module5_MYB
Module5_MTF1
Module5_MSC
Module5_MEOX2
Module5_MEIS2
Module5_MECOM
Module5_MAFB
Module5_LIN54
Module5_LBX1
Module5_KLF9
Module5_KLF4
Module5_KLF1
Module5_JUNB
Module5_JDP2
Module5_IRF9
Module5_IRF7
Module5_IRF3
Module5_ID4
Module5_HOXD9
Module5_HOXA13
Module5_HLTF
Module5_HIC1
Module5_GFI1B
Module5_GATA4
Module5_GATA2
Module5_GATA1
Module5_FOXB1
Module5_FOXA2
Module5_FOXA1
Module5_FOSL2
Module5_FOSL1
Module5_FOS
Module5_ESRRG
Module5_ESRRB
Module5_ESRRA
Module5_ESR2
Module5_EOMES
Module5_EMX1
Module5_EBF1
Module5_E2F8
Module5_E2F7
Module5_DMRT3
Module5_DLX1
Module5_CUX2
Module5_CUX1
Module5_CREB1
Module5_CDX1
Module5_BHLHA15
Module5_BCL6B
Module5_BACH2
Module5_ARID5A
Module5_AR
Module4_ETV5
Module4_ETV4
Module4_ETV1
Module4_ELK1
Module3_ZNF740
Module3_ZNF410
Module3_ZNF384
Module3_ZNF143
Module3_ZIC4
Module3_ZIC3
Module3_ZIC1
Module3_ZFX
Module3_ZEB1
Module3_ZBTB7C
Module3_ZBTB7B
Module3_ZBTB33
Module3_YY1
Module3_XBP1
Module3_VSX2
Module3_VSX1
Module3_VDR
Module3_VAX2
Module3_VAX1
Module3_USF2
Module3_UNCX
Module3_TWIST1
Module3_TP73
Module3_TP63
Module3_TP53
Module3_THAP1
Module3_TFEC
Module3_TFEB
Module3_TFE3
Module3_TFCP2
Module3_TFAP4
Module3_TFAP2C
Module3_TFAP2B
Module3_TFAP2A
Module3_TEF
Module3_TEAD3
Module3_TCF7L1
Module3_TCF4
Module3_TCF3
Module3_TCF21
Module3_TCF12
Module3_TBX4
Module3_TBX21
Module3_TBX20
Module3_TBX2
Module3_TBX1
Module3_TBR1
Module3_STAT6
Module3_STAT4
Module3_SRF
Module3_SREBF1
Module3_SPIC
Module3_SPI1

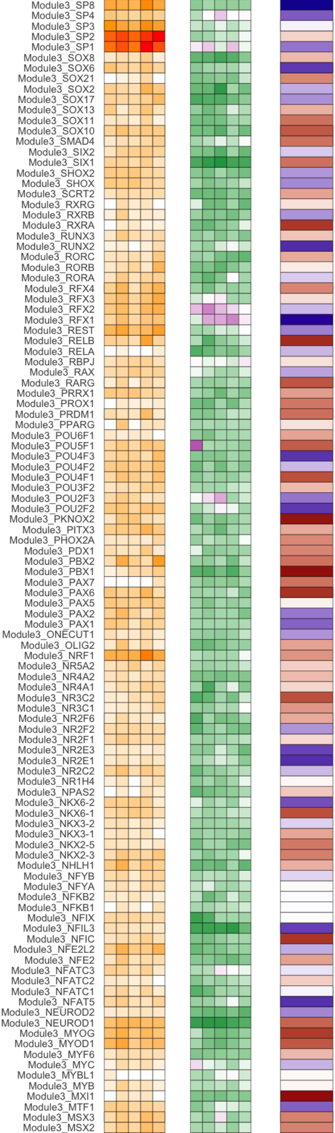

Module3_SP8
Module3_SP4
Module3_SP3
Module3_SP2
Module3_SP1
Module3_SOX8
Module3_SOX6
Module3_SOX21
Module3_SOX2
Module3_SOX17
Module3_SOX13
Module3_SOX11
Module3_SOX10
Module3_SMAD4
Module3_SIX2
Module3_SIX1
Module3_SHOX2
Module3_SHOX
Module3_SCRT2
Module3_RXRG
Module3_RXRB
Module3_RXRA
Module3_RUNX3
Module3_RUNX2
Module3_RORC
Module3_RORB
Module3_RORA
Module3_RFX4
Module3_RFX3
Module3_RFX2
Module3_RFX1
Module3_REST
Module3_RELB
Module3_RELA
Module3_RBPJ
Module3_RAX
Module3_RARG
Module3_PRRX1
Module3_PROX1
Module3_PRDM1
Module3_PPARG
Module3_POU6F1
Module3_POU5F1
Module3_POU4F3
Module3_POU4F2
Module3_POU4F1
Module3_POU3F2
Module3_POU2F3
Module3_POU2F2
Module3_PKNOX2
Module3_PITX3
Module3_PHOX2A
Module3_PDX1
Module3_PBX2
Module3_PBX1
Module3_PAX7
Module3_PAX6
Module3_PAX5
Module3_PAX2
Module3_PAX1
Module3_ONECUT1
Module3_OLIG2
Module3_NRF1
Module3_NR5A2
Module3_NR4A2
Module3_NR4A1
Module3_NR3C2
Module3_NR3C1
Module3_NR2F6
Module3_NR2F2
Module3_NR2F1
Module3_NR2E3
Module3_NR2E1
Module3_NR2C2
Module3_NR1H4
Module3_NPAS2
Module3_NKX6-2
Module3_NKX6-1
Module3_NKX3-2
Module3_NKX3-1
Module3_NKX2-5
Module3_NKX2-3
Module3_NHLH1
Module3_NFYB
Module3_NFYA
Module3_NFKB2
Module3_NFKB1
Module3_NFIX
Module3_NFIL3
Module3_NFIC
Module3_NFE2L2
Module3_NFE2
Module3_NFATC3
Module3_NFATC2
Module3_NFATC1
Module3_NFAT5
Module3_NEUROD2
Module3_NEUROD1
Module3_MYOG
Module3_MYOD1
Module3_MYF6
Module3_MYC
Module3_MYBL1
Module3_MYB
Module3_MXI1
Module3_MTF1
Module3_MSX3
Module3_MSX2

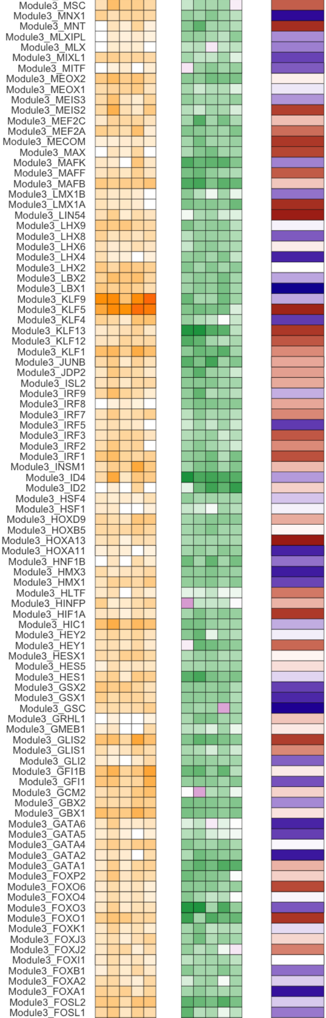
Module3_MSC
Module3_MNX1
Module3_MNT
Module3_MLXIPL
Module3_MLX
Module3_MIXL1
Module3_MITF
Module3_MEOX2
Module3_MEOX1
Module3_MEIS3
Module3_MEIS2
Module3_MEF2C
Module3_MEF2A
Module3_MECOM
Module3_MAX
Module3_MAFK
Module3_MAFF
Module3_MAFB
Module3_LMX1B
Module3_LMX1A
Module3_LIN54
Module3_LHX9
Module3_LHX8
Module3_LHX6
Module3_LHX4
Module3_LHX2
Module3_LBX2
Module3_LBX1
Module3_KLF9
Module3_KLF5
Module3_KLF4
Module3_KLF13
Module3_KLF12
Module3_KLF1
Module3_JUNB
Module3_JDP2
Module3_ISL2
Module3_IRF9
Module3_IRF8
Module3_IRF7
Module3_IRF5
Module3_IRF3
Module3_IRF2
Module3_IRF1
Module3_INSM1
Module3_ID4
Module3_ID2
Module3_HSF4
Module3_HSF1
Module3_HOXD9
Module3_HOXB5
Module3_HOXA13
Module3_HOXA11
Module3_HNF1B
Module3_HMX3
Module3_HMX1
Module3_HLTF
Module3_HINFP
Module3_HIF1A
Module3_HIC1
Module3_HEY2
Module3_HEY1
Module3_HESX1
Module3_HES5
Module3_HES1
Module3_GSX2
Module3_GSX1
Module3_GSC
Module3_GRHL1
Module3_GMEB1
Module3_GLIS2
Module3_GLIS1
Module3_GLI2
Module3_GFI1B
Module3_GFI1
Module3_GCM2
Module3_GBX2
Module3_GBX1
Module3_GATA6
Module3_GATA5
Module3_GATA4
Module3_GATA2
Module3_GATA1
Module3_FOXP2
Module3_FOXO6
Module3_FOXO4
Module3_FOXO3
Module3_FOXO1
Module3_FOXK1
Module3_FOXJ3
Module3_FOXJ2
Module3_FOXI1
Module3_FOXB1
Module3_FOXA2
Module3_FOXA1
Module3_FOSL2
Module3_FOSL1

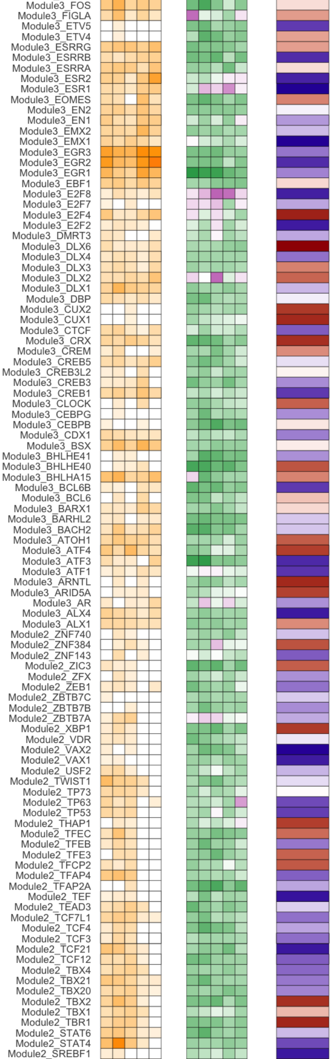
Module3_FOS
Module3_FIGLA
Module3_ETV5
Module3_ETV4
Module3_ESRRG
Module3_ESRRB
Module3_ESRRA
Module3_ESR2
Module3_ESR1
Module3_EOMES
Module3_EN2
Module3_EN1
Module3_EMX2
Module3_EMX1
Module3_EGR3
Module3_EGR2
Module3_EGR1
Module3_EBF1
Module3_E2F8
Module3_E2F7
Module3_E2F4
Module3_E2F2
Module3_DMRT3
Module3_DLX6
Module3_DLX4
Module3_DLX3
Module3_DLX2
Module3_DLX1
Module3_DBP
Module3_CUX2
Module3_CUX1
Module3_CTCF
Module3_CRX
Module3_CREM
Module3_CREB5
Module3_CREB3L2
Module3_CREB3
Module3_CREB1
Module3_CLOCK
Module3_CEBPG
Module3_CEBPB
Module3_CDX1
Module3_BSX
Module3_BHLHE41
Module3_BHLHE40
Module3_BHLHA15
Module3_BCL6B
Module3_BCL6
Module3_BARX1
Module3_BARHL2
Module3_BACH2
Module3_ATOH1
Module3_ATF4
Module3_ATF3
Module3_ATF1
Module3_ARNTL
Module3_ARID5A
Module3_AR
Module3_ALX4
Module3_ALX1
Module2_ZNF740
Module2_ZNF384
Module2_ZNF143
Module2_ZIC3
Module2_ZFX
Module2_ZEB1
Module2_ZBTB7C
Module2_ZBTB7B
Module2_ZBTB7A
Module2_XBP1
Module2_VDR
Module2_VAX2
Module2_VAX1
Module2_USF2
Module2_TWIST1
Module2_TP73
Module2_TP63
Module2_TP53
Module2_THAP1
Module2_TFEC
Module2_TFEB
Module2_TFE3
Module2_TFCP2
Module2_TFAP4
Module2_TFAP2A
Module2_TEF
Module2_TEAD3
Module2_TCF7L1
Module2_TCF4
Module2_TCF3
Module2_TCF21
Module2_TCF12
Module2_TBX4
Module2_TBX21
Module2_TBX20
Module2_TBX2
Module2_TBX1
Module2_TBR1
Module2_STAT6
Module2_STAT4
Module2_SREBF1

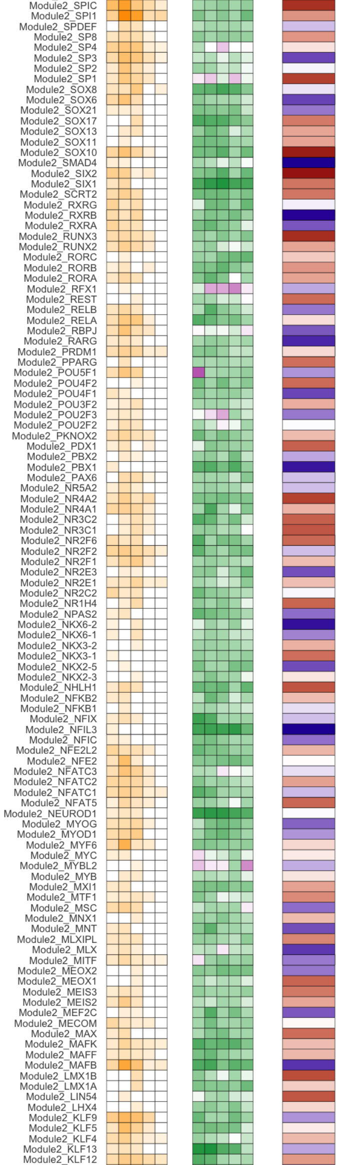
Module2_SPIC
Module2_SPI1
Module2_SPDEF
Module2_SP8
Module2_SP4
Module2_SP3
Module2_SP2
Module2_SP1
Module2_SOX8
Module2_SOX6
Module2_SOX21
Module2_SOX17
Module2_SOX13
Module2_SOX11
Module2_SOX10
Module2_SMAD4
Module2_SIX2
Module2_SIX1
Module2_SCRT2
Module2_RXRG
Module2_RXRB
Module2_RXRA
Module2_RUNX3
Module2_RUNX2
Module2_RORC
Module2_RORB
Module2_RORA
Module2_RFX1
Module2_REST
Module2_RELB
Module2_RELA
Module2_RBPJ
Module2_RARG
Module2_PRDM1
Module2_PPARG
Module2_POU5F1
Module2_POU4F2
Module2_POU4F1
Module2_POU3F2
Module2_POU2F3
Module2_POU2F2
Module2_PKNOX2
Module2_PDX1
Module2_PBX2
Module2_PBX1
Module2_PAX6
Module2_NR5A2
Module2_NR4A2
Module2_NR4A1
Module2_NR3C2
Module2_NR3C1
Module2_NR2F6
Module2_NR2F2
Module2_NR2F1
Module2_NR2E3
Module2_NR2E1
Module2_NR2C2
Module2_NR1H4
Module2_NPAS2
Module2_NKX6-2
Module2_NKX6-1
Module2_NKX3-2
Module2_NKX3-1
Module2_NKX2-5
Module2_NKX2-3
Module2_NHLH1
Module2_NFKB2
Module2_NFKB1
Module2_NFIX
Module2_NFIL3
Module2_NFIC
Module2_NFE2L2
Module2_NFE2
Module2_NFATC3
Module2_NFATC2
Module2_NFATC1
Module2_NFAT5
Module2_NEUROD1
Module2_MYOG
Module2_MYOD1
Module2_MYF6
Module2_MYC
Module2_MYBL2
Module2_MYB
Module2_MXI1
Module2_MTF1
Module2_MSC
Module2_MNX1
Module2_MNT
Module2_MLXIPL
Module2_MLX
Module2_MITF
Module2_MEOX2
Module2_MEOX1
Module2_MEIS3
Module2_MEIS2
Module2_MEF2C
Module2_MECOM
Module2_MAX
Module2_MAFK
Module2_MAFF
Module2_MAFB
Module2_LMX1B
Module2_LMX1A
Module2_LIN54
Module2_LHX4
Module2_KLF9
Module2_KLF5
Module2_KLF4
Module2_KLF13
Module2_KLF12

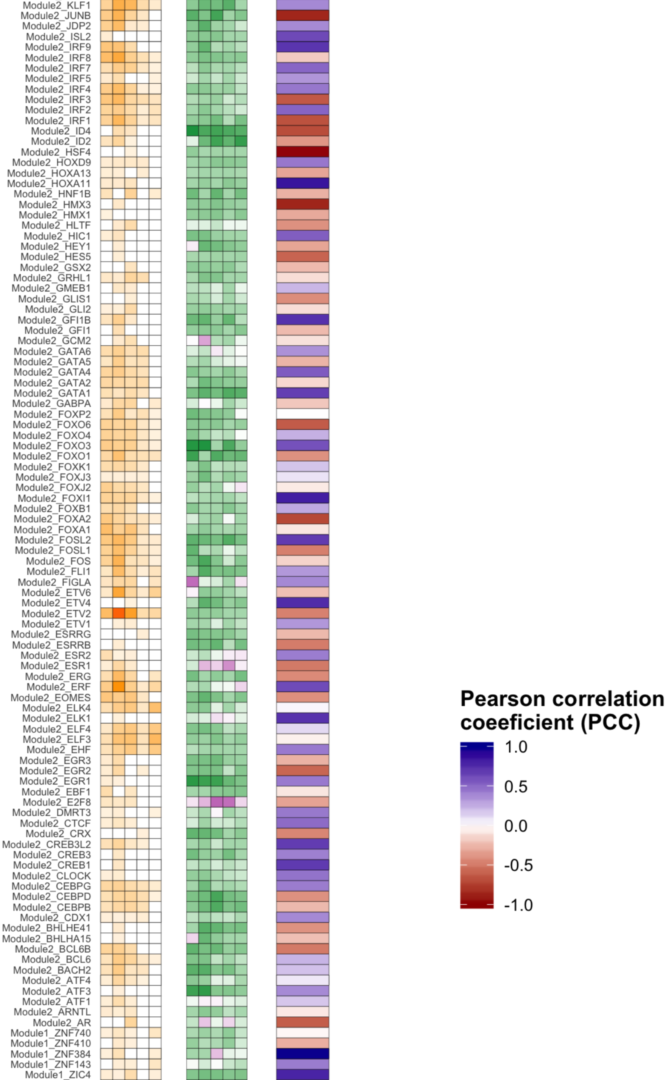
Module2_KLF1
Module2_JUNB
Module2_JDP2
Module2_ISL2
Module2_IRF9
Module2_IRF8
Module2_IRF7
Module2_IRF5
Module2_IRF4
Module2_IRF3
Module2_IRF2
Module2_IRF1
Module2_ID4
Module2_ID2
Module2_HSF4
Module2_HOXD9
Module2_HOXA13
Module2_HOXA11
Module2_HNF1B
Module2_HMX3
Module2_HMX1
Module2_HLTF
Module2_HIC1
Module2_HEY1
Module2_HES5
Module2_GSX2
Module2_GRHL1
Module2_GMEB1
Module2_GLIS1
Module2_GLI2
Module2_GFI1B
Module2_GFI1
Module2_GCM2
Module2_GATA6
Module2_GATA5
Module2_GATA4
Module2_GATA2
Module2_GATA1
Module2_GABPA
Module2_FOXP2
Module2_FOXO6
Module2_FOXO4
Module2_FOXO3
Module2_FOXO1
Module2_FOXK1
Module2_FOXJ3
Module2_FOXJ2
Module2_FOXI1
Module2_FOXB1
Module2_FOXA2
Module2_FOXA1
Module2_FOSL2
Module2_FOSL1
Module2_FOS
Module2_FLI1
Module2_FIGLA
Module2_ETV6
Module2_ETV4
Module2_ETV2
Module2_ETV1
Module2_ESRRG
Module2_ESRRB
Module2_ESR2
Module2_ESR1
Module2_ERG
Module2_ERF
Module2_EOMES
Module2_ELK4
Module2_ELK1
Module2_ELF4
Module2_ELF3
Module2_EHF
Module2_EGR3
Module2_EGR2
Module2_EGR1
Module2_EBF1
Module2_E2F8
Module2_DMRT3
Module2_CTCF
Module2_CRX
Module2_CREB3L2
Module2_CREB3
Module2_CREB1
Module2_CLOCK
Module2_CEBPG
Module2_CEBPD
Module2_CEBPB
Module2_CDX1
Module2_BHLHE41
Module2_BHLHA15
Module2_BCL6B
Module2_BCL6
Module2_BACH2
Module2_ATF4
Module2_ATF3
Module2_ATF1
Module2_ARNTL
Module2_AR
Module1_ZNF740
Module1_ZNF410
Module1_ZNF384
Module1_ZNF143
Module1_ZIC4
Pearson correlation
coeeficient (PCC)
1.0
0.5
0.0
-0.5
-1.0

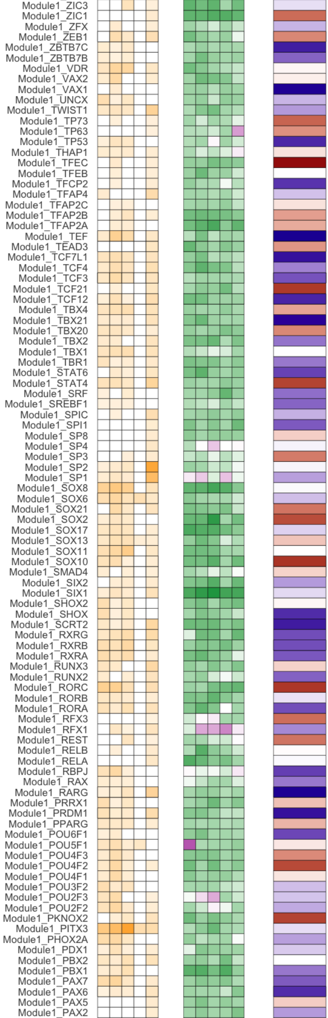
Module1_ZIC3
Module1_ZIC1
Module1_ZFX
Module1_ZEB1
Module1_ZBTB7C
Module1_ZBTB7B
Module1_VDR
Module1_VAX2
Module1_VAX1
Module1_UNCX
Module1_TWIST1
Module1_TP73
Module1_TP63
Module1_TP53
Module1_THAP1
Module1_TFEC
Module1_TFEB
Module1_TFCP2
Module1_TFAP4
Module1_TFAP2C
Module1_TFAP2B
Module1_TFAP2A
Module1_TEF
Module1_TEAD3
Module1_TCF7L1
Module1_TCF4
Module1_TCF3
Module1_TCF21
Module1_TCF12
Module1_TBX4
Module1_TBX21
Module1_TBX20
Module1_TBX2
Module1_TBX1
Module1_TBR1
Module1_STAT6
Module1_STAT4
Module1_SRF
Module1_SREBF1
Module1_SPIC
Module1_SPI1
Module1_SP8
Module1_SP4
Module1_SP3
Module1_SP2
Module1_SP1
Module1_SOX8
Module1_SOX6
Module1_SOX21
Module1_SOX2
Module1_SOX17
Module1_SOX13
Module1_SOX11
Module1_SOX10
Module1_SMAD4
Module1_SIX2
Module1_SIX1
Module1_SHOX2
Module1_SHOX
Module1_SCRT2
Module1_RXRG
Module1_RXRB
Module1_RXRA
Module1_RUNX3
Module1_RUNX2
Module1_RORC
Module1_RORB
Module1_RORA
Module1_RFX3
Module1_RFX1
Module1_REST
Module1_RELB
Module1_RELA
Module1_RBPJ
Module1_RAX
Module1_RARG
Module1_PRRX1
Module1_PRDM1
Module1_PPARG
Module1_POU6F1
Module1_POU5F1
Module1_POU4F3
Module1_POU4F2
Module1_POU4F1
Module1_POU3F2
Module1_POU2F3
Module1_POU2F2
Module1_PKNOX2
Module1_PITX3
Module1_PHOX2A
Module1_PDX1
Module1_PBX2
Module1_PBX1
Module1_PAX7
Module1_PAX6
Module1_PAX5
Module1_PAX2

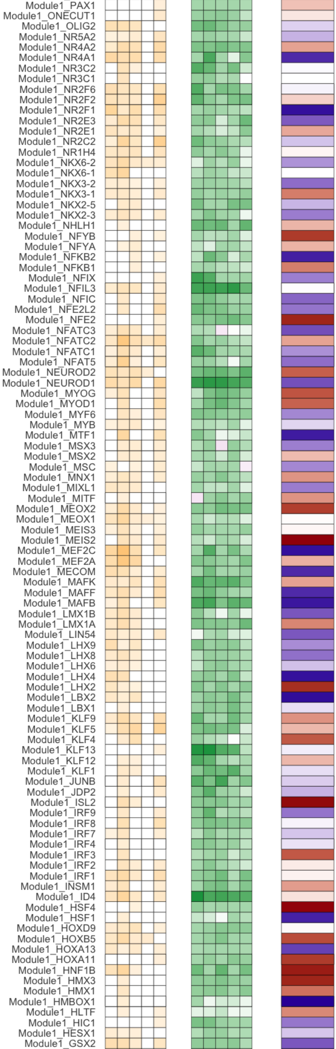
Module1_PAX1
Module1_ONECUT1
Module1_OLIG2
Module1_NR5A2
Module1_NR4A2
Module1_NR4A1
Module1_NR3C2
Module1_NR3C1
Module1_NR2F6
Module1_NR2F2
Module1_NR2F1
Module1_NR2E3
Module1_NR2E1
Module1_NR2C2
Module1_NR1H4
Module1_NKX6-2
Module1_NKX6-1
Module1_NKX3-2
Module1_NKX3-1
Module1_NKX2-5
Module1_NKX2-3
Module1_NHLH1
Module1_NFYB
Module1_NFYA
Module1_NFKB2
Module1_NFKB1
Module1_NFIX
Module1_NFIL3
Module1_NFIC
Module1_NFE2L2
Module1_NFE2
Module1_NFATC3
Module1_NFATC2
Module1_NFATC1
Module1_NFAT5
Module1_NEUROD2
Module1_NEUROD1
Module1_MYOG
Module1_MYOD1
Module1_MYF6
Module1_MYB
Module1_MTF1
Module1_MSX3
Module1_MSX2
Module1_MSC
Module1_MNX1
Module1_MIXL1
Module1_MITF
Module1_MEOX2
Module1_MEOX1
Module1_MEIS3
Module1_MEIS2
Module1_MEF2C
Module1_MEF2A
Module1_MECOM
Module1_MAFK
Module1_MAFF
Module1_MAFB
Module1_LMX1B
Module1_LMX1A
Module1_LIN54
Module1_LHX9
Module1_LHX8
Module1_LHX6
Module1_LHX4
Module1_LHX2
Module1_LBX2
Module1_LBX1
Module1_KLF9
Module1_KLF5
Module1_KLF4
Module1_KLF13
Module1_KLF12
Module1_KLF1
Module1_JUNB
Module1_JDP2
Module1_ISL2
Module1_IRF9
Module1_IRF8
Module1_IRF7
Module1_IRF4
Module1_IRF3
Module1_IRF2
Module1_IRF1
Module1_INSM1
Module1_ID4
Module1_HSF4
Module1_HSF1
Module1_HOXD9
Module1_HOXB5
Module1_HOXA13
Module1_HOXA11
Module1_HNF1B
Module1_HMX3
Module1_HMX1
Module1_HMBOX1
Module1_HLTF
Module1_HIC1
Module1_HESX1
Module1_GSX2

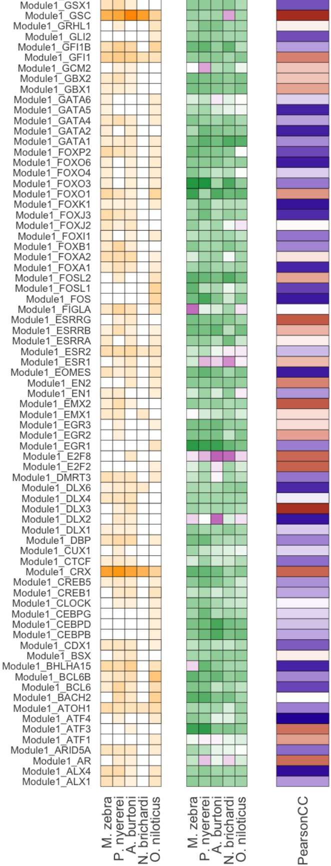
Module1_GSX1
Module1_GSC
Module1_GRHL1
Module1_GLI2
Module1_GFI1B
Module1_GFI1
Module1_GCM2
Module1_GBX2
Module1_GBX1
Module1_GATA6
Module1_GATA5
Module1_GATA4
Module1_GATA2
Module1_GATA1
Module1_FOXP2
Module1_FOXO6
Module1_FOXO4
Module1_FOXO3
Module1_FOXO1
Module1_FOXK1
Module1_FOXJ3
Module1_FOXJ2
Module1_FOXI1
Module1_FOXB1
Module1_FOXA2
Module1_FOXA1
Module1_FOSL2
Module1_FOSL1
Module1_FOS
Module1_FIGLA
Module1_ESRRG
Module1_ESRRB
Module1_ESRRA
Module1_ESR2
Module1_ESR1
Module1_EOMES
Module1_EN2
Module1_EN1
Module1_EMX2
Module1_EMX1
Module1_EGR3
Module1_EGR2
Module1_EGR1
Module1_E2F8
Module1_E2F2
Module1_DMRT3
Module1_DLX6
Module1_DLX4
Module1_DLX3
Module1_DLX2
Module1_DLX1
Module1_DBP
Module1_CUX1
Module1_CTCF
Module1_CRX
Module1_CREB5
Module1_CREB1
Module1_CLOCK
Module1_CEBPG
Module1_CEBPD
Module1_CEBPB
Module1_CDX1
Module1_BSX
Module1_BHLHA15
Module1_BCL6B
Module1_BCL6
Module1_BACH2
Module1_ATOH1
Module1_ATF4
Module1_ATF3
Module1_ATF1
Module1_ARID5A
Module1_AR
Module1_ALX4
Module1_ALX1
M. zebra
P. nyererei
A. burtoni
N. brichardi
O. niloticus
M. zebra
P. nyererei
A. burtoni
N. brichardi
O. niloticus
PearsonCC
