# Supplementary material for: Evolution of regulatory networks associated with traits under selection in cichlids
Source: Genome Biol. 2021 Jan 8;22:25. doi: 10.1186/s13059-020-02208-8 (PMC7791837; doi:10.1186/s13059-020-02208-8)
Supplement: Supplementary file 1 — Additional file 1. Supplementary analysis notes, figures and tables. This file includes supplementary notes, along with supplementary figs. S1-S22 and Tables S1-S3 referenced in the main text. This file also includes legends for figs and tables in Additional files 2 and 3. [file 13059_2020_2208_MOESM1_ESM.pdf]

<u>Additional file 1</u>

Results

Gene co-expression is tissue-specific and highlights functional evolutionary trajectories

The functional landscape of modules can be related to tissue-specific co-expression (Additional file 1: Fig. S1b, Additional file 2: Fig. S1); for example, module 3 with strong brain induction (Fig. 1a) is significantly enriched for neural processes, reflecting core co-regulated networks of genes associated with signal transduction and synaptic activity (FDR <0.05, Additional file 1: Fig. S1b, Additional file 2: Fig. S1). Modules with variable tissue co-expression e.g. module 2 (Fig. 1a) have divergent functional enrichment across species, suggesting that proteolysis and ribosomal activity (FDR <0.05, Additional file 1: Fig. S1b, Additional file 2: Fig. S1) in kidney and heart physiological function is potentially different among the five cichlid species.

Orthologous genes of each species can be assigned to non-orthologous modules (Fig S-R1a), indicative of potential co-expression divergence and transcriptional rewiring from the LCA (referred to as 'state changes' in module assignment). To ensure orthologous genes of all branches are included in subsequent analysis, we focused on state changes of 6,844 1-to-1 orthologous genes to assess the level of convergent and unique state changes along the phylogeny (Fig. 1b). We identified convergent state changes of 732 genes along all ancestral nodes versus Anc4 (Additional file 1: Fig. S2a). This is made up of 772 genes in Anc3 and Anc2, 734 genes in Anc3 and Anc1, and 996 genes in Anc2 and Anc1 (Additional file 1: Fig. S2a), including a few TFs (46 TFs - Anc3-2-1; 49 TFs - Anc3-2; 46 TFs - Anc3-1; 66 TFs - Anc2-1) such as *tbx20, nkx3-1* and *hoxd10.* When mapping convergent state changes on species branches (focal species vs any other species), we note that there are 463 genes in *M. zebra*, 443 in *P. nyererei*, 394 in *A. burtoni*, 521 in *N. brichardi*, and 527 in

O. niloticus (orange numbers, Fig. 1b). In each species (except *P. nyererei*), there are more state changes and expression divergence of genes, than convergent state changes with any of the other species (purple vs orange numbers, Fig. 1b). All convergent state changes between two, three and four species is further summarised in the venn diagram (Additional file 1: Fig. S2b); convergence of genes is highest in 1) *N. brichardi* and *O. niloticus* (229 genes) when comparing two species versus the rest; 2) the haplochromines (358 genes) when comparing three species versus the rest; and 3) the three haplochromines and *N. brichardi* (983 genes) when comparing four species and the remaining single species. The fewest convergent state changes are generally observed when comparing evolutionary distant species e.g. *M. zebra* and *A. burtoni* (41 genes) versus the rest (Additional file 1: Fig. S2b). Overall, convergent state changes are generally higher between phylogenetically closer species but in general, lower than unique state changes between species (Fig. 1b; Fig. S-R1c-d).

We identified unique state changes and expression divergence of 655 genes along ancestral nodes (Fig. 1b), including several cellular and developmental TFs (51 TFs - Anc4/3; 20 TFs - Anc3/2; 34 TFs - Anc2/1) such as *foxo1, hoxa11* and *lbx1*. These state changed regulatory TFs are also enriched in module gene promoters according to tissue-specific function like, for example, promoters of module 1 genes (eye-specific expression) are significantly enriched (False Discovery Rate, FDR <0.05) for TF motifs involved in retina- and lens-related development/functions e.g. CRX, PITX3 and OTX1 [1] and module 9 genes (brain-specific expression) are significantly enriched (FDR<0.05) for TF motifs involved in brain development/functions e.g. EGR1 [2] and NEUROD2 (Additional file 1: Fig. S3, Additional file 2: Fig. S2). We observe variability in motif enrichment of TFs across species genes e.g. RAR*α/β/γ* and RXR*α/β/γ* [3] of module 1 gene promoters in all species except *N. brichardi* (Additional file 1: Fig. S3, Additional file 2: Fig. S2).

Using a measure of gene expression tissue-specificity, *tau* [4], we show that genes with no state change in module assignment (green bars) have an even, narrow to mid-intermediate breadth of expression whereas state changed genes (red bars) have a narrow to broad expression breadth (Additional file 1: Fig. S6). This is representative of orthologs clustering in non-orthologous modules (state changes) and such variability could be associated with variability at gene regulatory regions e.g. gene promoter. The observed variability of motif enrichment Additional file 1: Fig. S3, Additional file 2: Fig. S2), linked to TF expression changes (state-changing) reflects a shifted domain of tissue expression, implying differences in the regulatory control of target genes along the phylogeny. We test this by computing the Pearson correlation coefficient (r) between the cross-species TF motif enrichment and tissue-specific expression across species (see *Main Text*) using the n=5 species. Our analysis identified several cases of TFs whose expression change/stability was correlated with motif enrichment change. For several TFs that are functionally associated with tissues [5,6], we note a gradual increase in expression along the phylogeny, positively correlated with an increase in motif enrichment e.g. Brain-Cluster2-NFATC3 (r=0.99, n=5, p-value<0.05), Testis-Cluster3-LBX1 (r=0.98, n=5, p-value<0.05), Kidney-Cluster3-DLX3 (r=1.00, n=5, p-value<0.05), Heart-Cluster1-ISL2 (r=0.99, n=5, p-value<0.05) (Additional file 1: Fig. S5). In other TFs, positive correlation was due to a focused shift in expression where most species have similar fold enrichment e.g. Brain-Cluster2-CDX1 (r=0.98, n=5, p-value<0.05) and similar tissue-specific expression profiles (stable within a subset of species), whereas in the divergent species e.g. *N. brichardi*, the expression profile is negatively shifted along with a different motif enrichment (Additional file 1: Fig. S5, Additional file 2: Fig. S3-S8). There are several cases of this in highly correlated TFs across tissues, examples of which include Brain-Cluster6-EBF1 (r=1.0), Eye-Cluster5-E2F7 (r=0.97, n=5, p-value<0.05), Heart-Cluster5-TBR1 (r=0.99, n=5, p-value<0.05), Kidney-Cluster5-CDX1 (r=1.0, n=5, p-value<0.05), Muscle-Cluster5-TBR1 (r=1.0, n=5, p-value<0.05) and Testis-Cluster7-EN2 (r=0.98, n=5, p-value<0.05) (Additional file 1: Fig. S5, Additional file 2: Fig. S3-S8).

Overall, we generally note that TFs with a similar motif fold enrichment across all taxa and similar expression across most (but not all) species (state-change in one species) are most positively correlated e.g. Brain-Cluster2-CDX1 (Additional file 1: Fig. S5, Additional file 2: Fig. S3). Similarly, TFs with comparable motif fold enrichment and expression (no state-change) across all species are amongst the most positively correlated e.g. Brain Module9-ZBTB7B, Module9-TEF, Module9-SOX6, Module9-RBPJ, Module9-NFIL3 (Additional file 2: Fig. S3). On the other hand, TFs with subsets of similar motif enrichment in more than one species have slightly reduced, but positively correlated with an expression change in the same species subsets e.g. Eye-Cluster7-RFX4 ($r=0.95$, $n=5$, p-value <0.05) (Additional file 1: Fig. S5, Additional file 2: Fig. S3). At the other end, TFs with no correlation tend to have variability in motif fold enrichment and/or expression (state-change) across all species e.g. Brain Module9-SOX2, Module9-RFX4, Module9-RARG, Module-IRF8, Module9-HSF1. These patterns are present across all tissues (Additional file 2: Fig. S3-S8), and therefore shows that there is a reduction in correlation when there are large shifts in motif enrichment and/or expression in several species (several phylogenetic state-changes), but otherwise positively correlated when there are no shifts (no TF state-changes) or subtle shifts (TF state-change in one or subsets of species).

For selected TFs and tissues, the levels of motif enrichment in gene promoters and TF expression are therefore correlated; similar levels of motif enrichment are largely associated with expression conservation (across all species) and subtle expression changes (in one or subsets of species), and therefore more stable (in expression differences) than TFs with variable motif enrichment along the phylogeny. This highlights differential gene regulatory programmes in the five cichlids, that we later confirm to be subtle differences in TFBSs when studying network rewiring events.

Owing to the variability in motif enrichment of retina/lens related TFs of module 1 gene promoters (Additional file 1: Fig. S3, see *Main Text*), we test the Pearson correlation coefficient (r) between the cross-species TF motif enrichment and eye expression across species. A change in TF motif enrichment between species is representative of either a gain or loss (decay) of TFBSs in eye-expressed (module 1) genes. For module 1 (eye-expressed) gene promoter motifs, we show that some TFs have a near positive correlation (+1) of TF motif enrichment and eye expression e.g. RORC (r=0.92, n=5, p-value<0.05), GLI2 (r=0.88,n=5, p-value<0.1), CLOCK (r=0.86, n=5, p-value<0.1) and CRX (r=0.85, n=5, p-value<0.1) across species (Additional file 1: Fig. S6 *top row*, Additional file 2: Fig. S4). Some of these TFs for example have important functions in modulating opsin expression e.g. CRX [7] and Hedgehog signalling [8] in retinal axon guidance [9] e.g. GLI2. Across these TFs, there are examples in one species (RORC and CRX) or multiple species (CLOCK), where there is an increased enrichment of motifs in module 1 gene promoters (gain of TFBSs) compared to the other species, that are positively correlated with a concurrent increase in TF eye expression (Additional file 1: Fig. S6). Therefore, a gain of retinal TF motifs in eye expressed genes is positively correlated with increased expression in the eye. In CRX, a TF known to modulate opsin expression in zebrafish [7] and exhibiting TFBS turnover in cichlid opsin genes [10], there is a significant increase in eye expression (0 > 4+) upon doubling the level of motif enrichment in *M. zebra, P. nyererei, A. burtoni* and *O. niloticus* as compared to *N. brichardi* in general (Additional file 1: Fig. S6). This is due to loss (or decay) of retinal motifs associated with decreased eye expression in *N. brichardi* (compared to the ancestral species, *O. niloticus*). However, a similar level of motif enrichment in the haplochromines (*M. zebra, P. nyererei* and *A. burtoni*), that is slightly higher than motif enrichment in *O. niloticus*, is associated with a concurrent higher level of eye expression than *O. niloticus* (Additional file 1: Fig. S6). Along the phylogeny, a similar pattern is observed in all TFs with r>0.7 (Additional file 1: Fig. S6), indicating that variable motif enrichment in eye-expressed genes is associated with a concurrent change (increase/decrease) in TF eye expression along the phylogeny.

In summary, these correlative patterns of TF expression changes (state-changing module assignment) and TFBS variation, indicative of motif gain and loss, suggest shifted domains of expression in tissues of species along the phylogeny, implying regulatory control by different suites of regulators. This highlights differential gene regulatory programmes, that could be associated with regulatory network changes underpinning traits under selection in cichlids, such as the visual system [11].

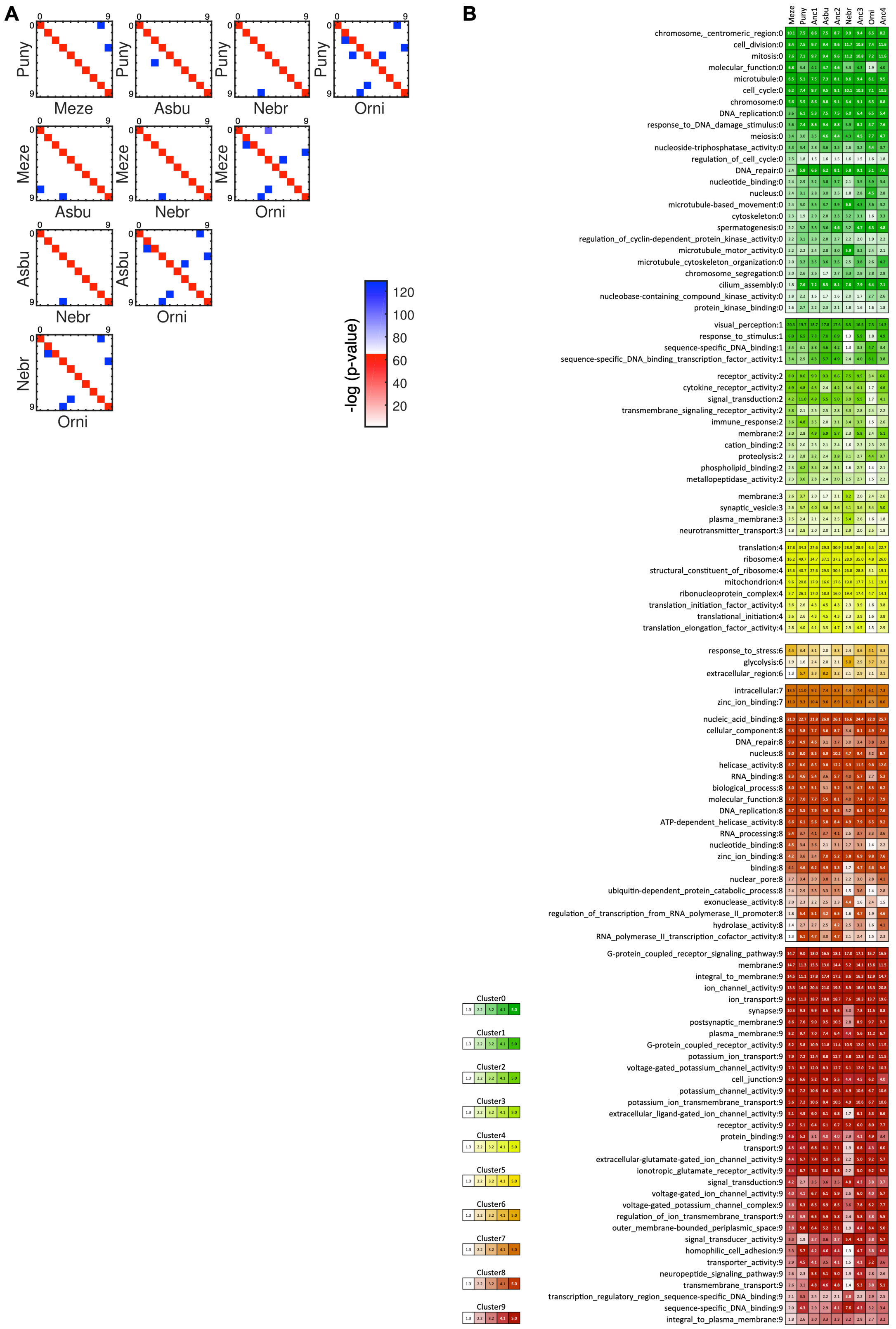

Fig. S1 – (a) Overlap of module genes between cichlid species. Shown is the degree overlap of orthologous genes between every module (0-9) pair (rows and columns in each matrix) and in every extant species pair. Diagonal elements (red): overlap between modules of the same ID; off-diagonal elements (blue): overlap between modules of different IDs. Red

and blue intensity is proportional to −log (*P*-value) of the hypergeometric distribution (*right,* color scales). **(b) Conserved Gene Ontology (GO) enrichment of modules across all extant and ancestral species.** Conserved enriched terms of significance FDR-corrected *P*-value (*q*-value <0.05) in modules (rows and ':n' module number) are shown for extant and ancestral species (columns) and colored according to module and gradient, -log(*q*-value) in each grid position (see legend, *left*). Set-based hypergeometric test of enrichment carried out using a background of all module genes. Module 5 does not have any conserved enriched terms of significance (FDR<0.05) and instead, all enriched terms for each module are found in Additional file 2: Fig. S1.

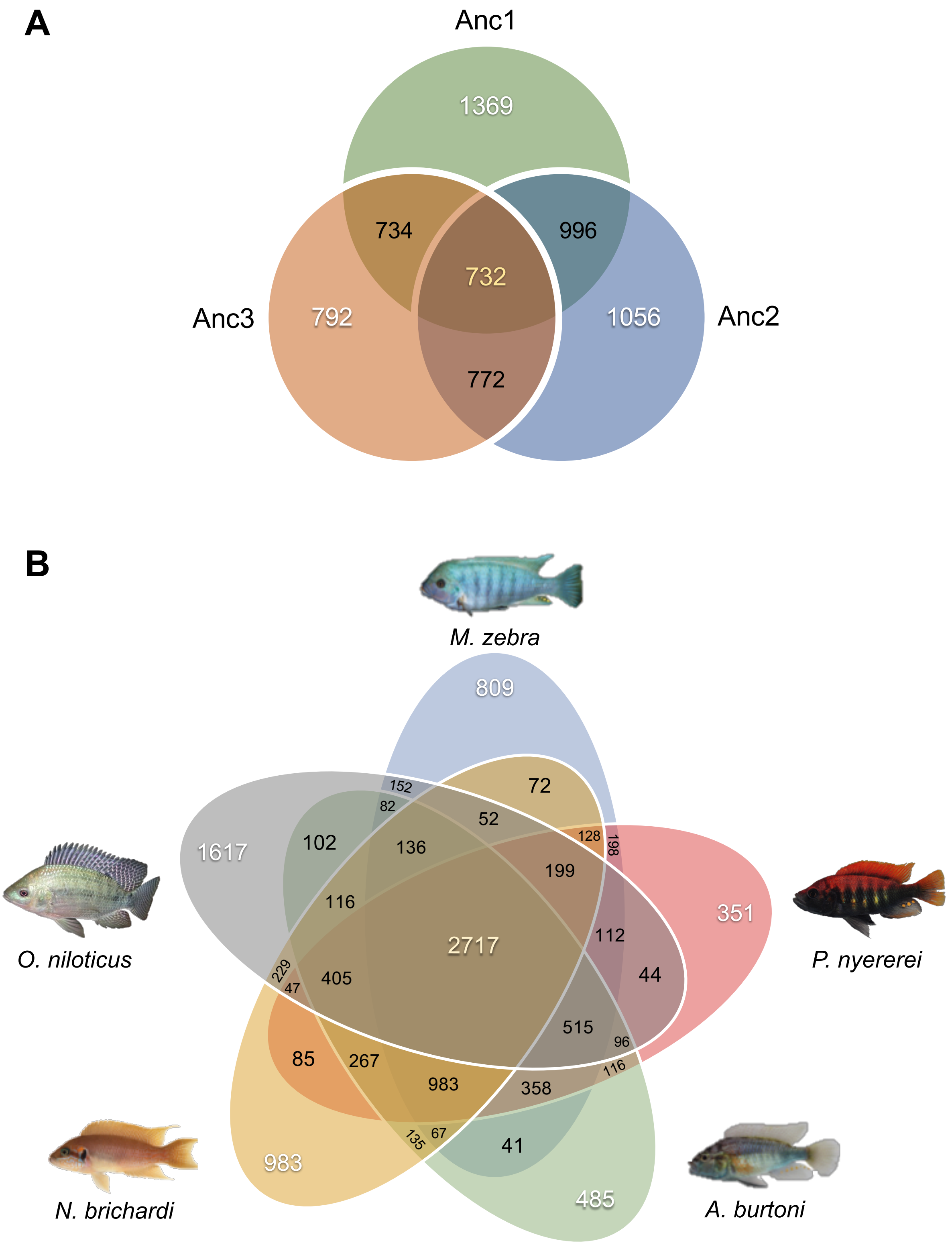

Fig. S2 – (a) Unique and convergent state changes of 1-to-1 orthologous genes at three ancestral nodes compared to Anc4. Venn diagram of unique state changes (white numbers), and convergent state changes (black and yellow number/s) of 1-to-1 orthologous genes between three ancestral nodes (Anc3, 2 and 1) versus Anc4 (as per phylogeny in Fig. 1b). **(b) Unique and convergent state changes of 1-to-1 orthologous genes at each species branch.** Venn diagram of unique state changes (white numbers), and convergent

state changes (black numbers) of 1-to-1 orthologous genes between all combinations of two, three, and four species versus the other remaining species. Central number (in yellow) represents 1-to-1 orthologous genes assigned to the same module in all five species.

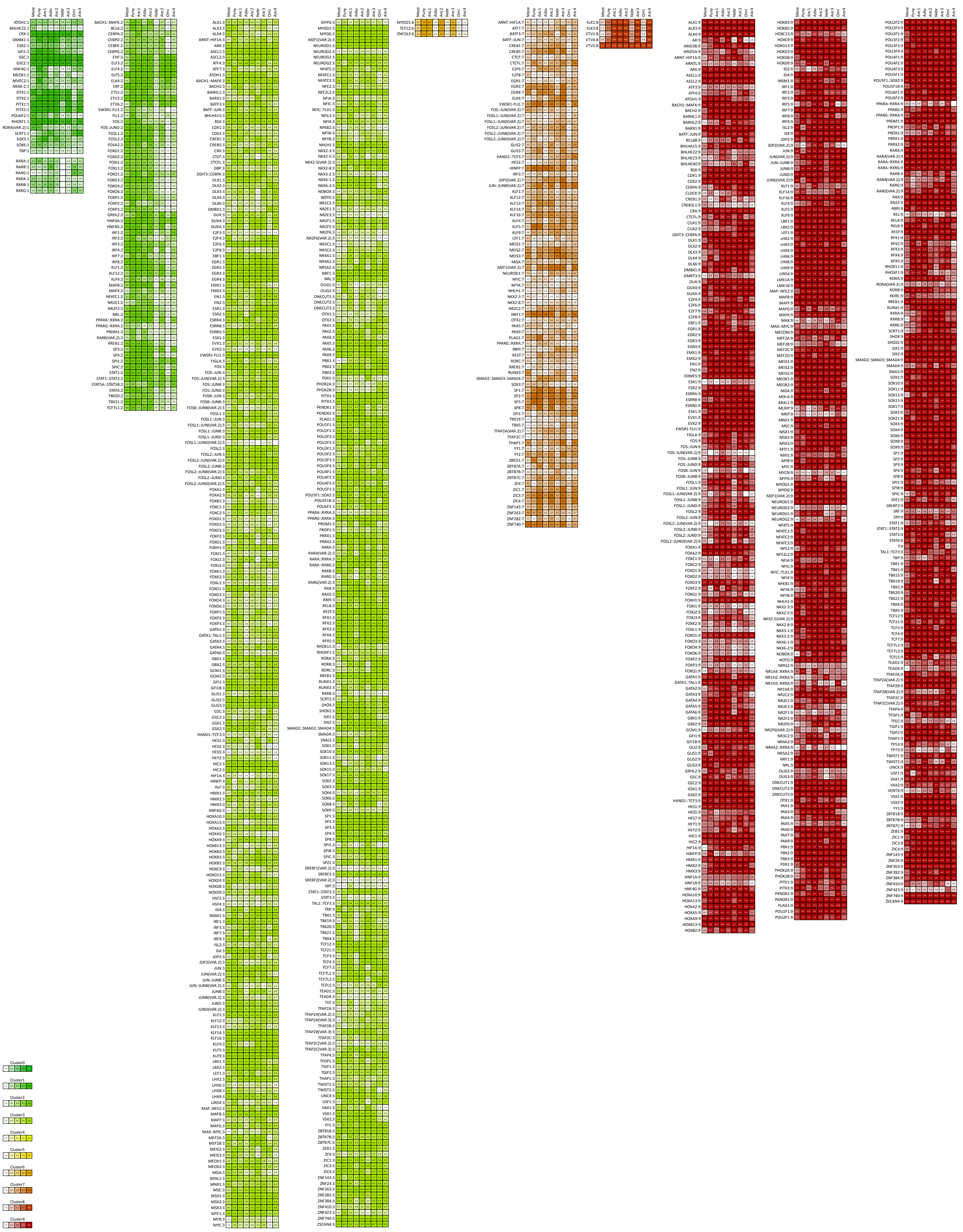

Fig. S3 – Conserved transcription factor motif enrichment of module gene promoters across all extant and ancestral species. Conserved enriched motifs of significance FDR-corrected *P*-value (*q*-value <0.05) in modules (rows and ‘:n’ module number) are shown for extant and ancestral species (columns) and colored according to module and gradient, -log(*q*-value) in each grid position (see legend, *left*). All enriched motifs shown are only for conserved across extant and ancestral species modules with the exception of RAR and RXR in module 1 that are shown for the purpose of functional validations. Set-based

hypergeometric test of enrichment carried out using a background of all module genes. Selected modules (0, 4 and 5) do not have any conserved enriched motifs of significance (FDR<0.05) and instead, all enriched motifs for each module are found in Additional file 2: Fig. S2.

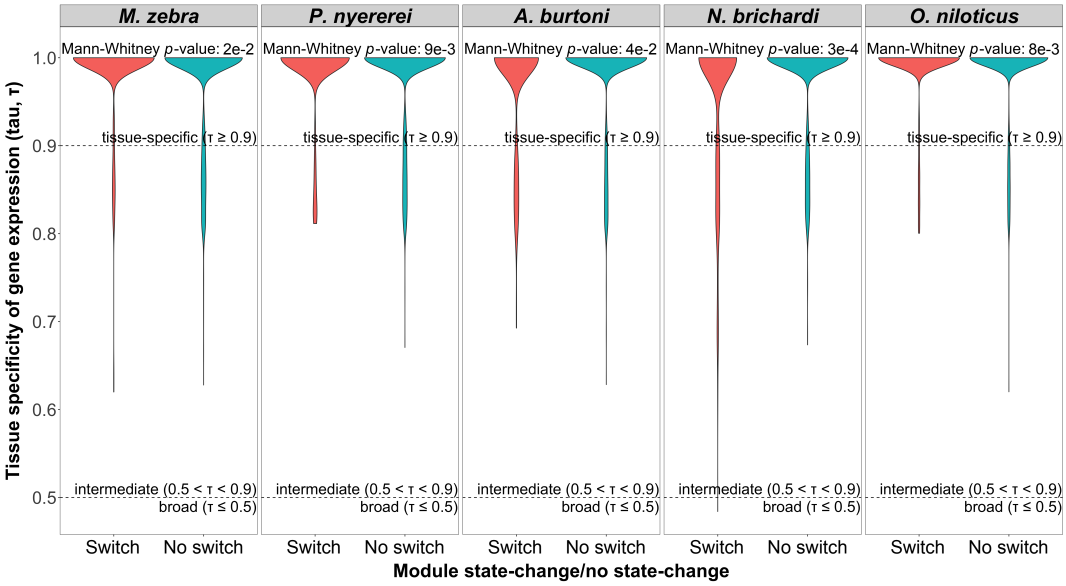

Fig. S4 – Module genes breadth of expression. Calculated as Tau (demarcated in plots), shown for each of five species module genes that are switch/state changed (*left*, red violin bars) and no switch/non state changed (*right*, green violin bars). *P* values describing difference between state changed and non-state changed genes breadth of expression calculated using Mann-Whitney test.

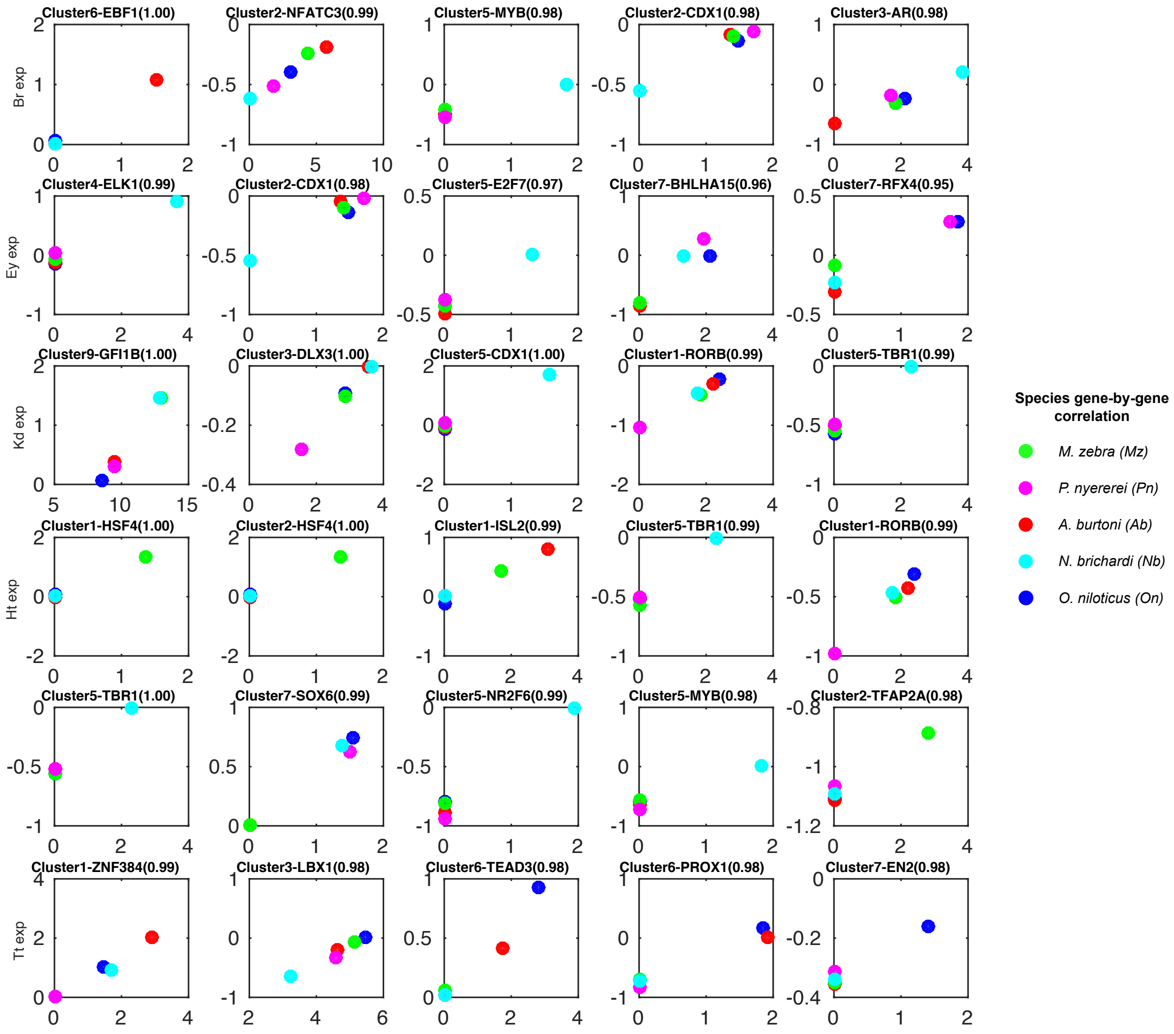

Fig. S5 – Top five TF motif enrichment and tissue expression correlations across the five cichlid species. Scatter plots relating to the -log(*q*-value) of fold enrichment (x-axis) in different module gene promoters and the expression of the transcription factor in six tissues (y-axis). The expression is log zero-mean where the mean of the gene is computed for each species. The title of each scatter plot indicates the module of enrichment (Cluster), TF symbol, and Pearson correlation coefficient (r) for all points on the plot. Each dot colour corresponds to a particular species as per legend.

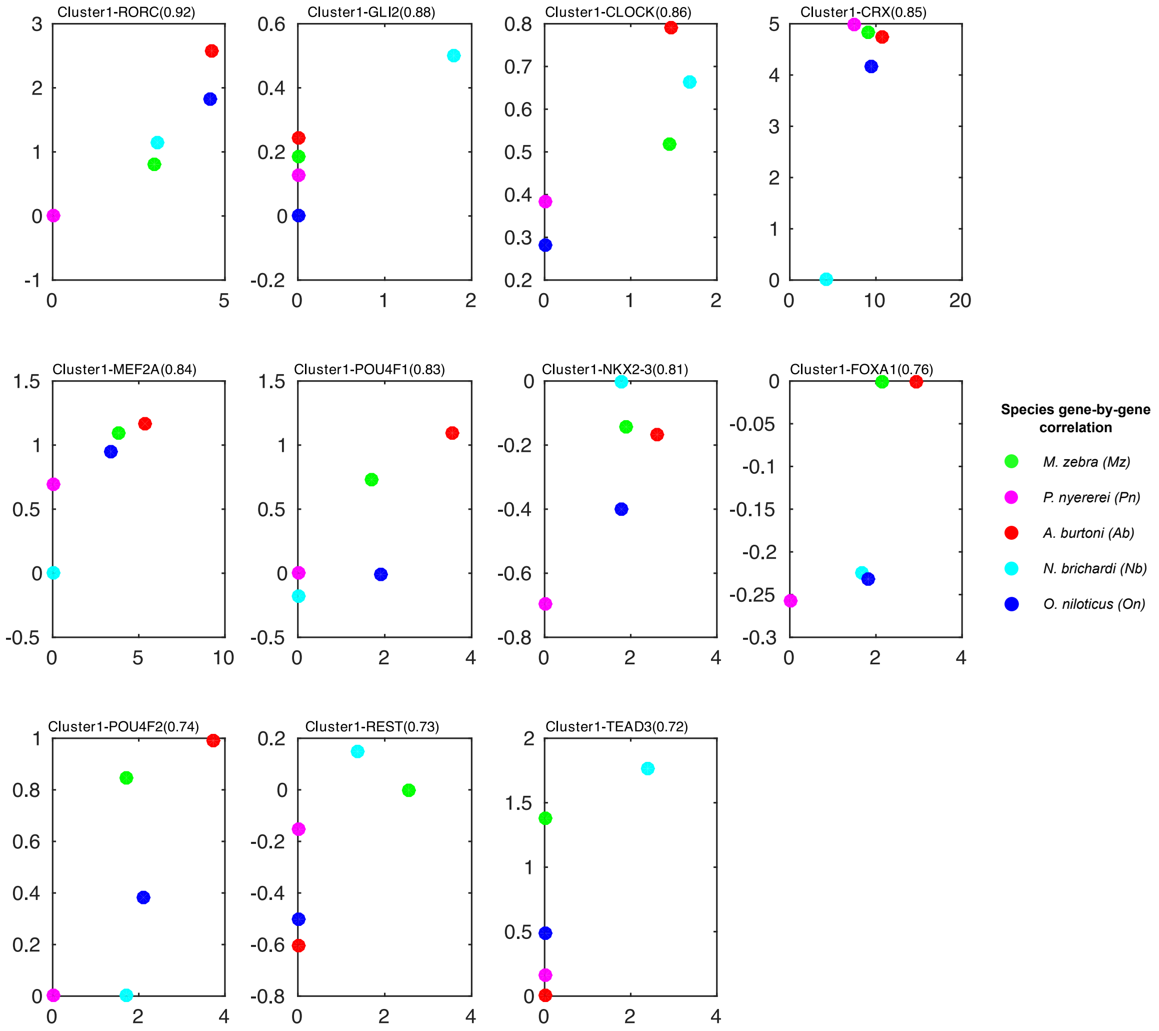

Fig. S6 – TF motif enrichment in module 1 genes and eye expression correlations >0.7 across the five cichlid species. Scatter plots relating to the -log(*q*-value) of fold enrichment (x-axis) of all module 1 gene promoters and the expression of the transcription factor in eye tissue (y-axis). The expression is log zero-mean where the mean of the gene is computed for each species. The title of each scatter plot indicates the module of enrichment (Cluster1), TF symbol, and Pearson correlation coefficient (r) for all points on the plot. Each dot colour corresponds to a particular species as per legend.

Fine scale nucleotide variation at TF binding sites drives functional regulatory divergence in cichlids through GRN rewiring

The impact of noncoding sequence variation on gene expression was tested based on the evolutionary rate of 4622 1:1 orthologous gene promoter sequences against synonymous (fourfold degenerate) sites of protein coding regions, used as a proxy for neutral evolution. In the five cichlid genomes, there is no significant increase in evolutionary rate at promoter regions compared to fourfold-degenerate sites (Additional file 1: Fig. S7a, c, e, and f), with also no difference in promoter evolutionary rate between state-changed and non-state changed genes. We identify very few outlier genes with significantly higher evolutionary rate at promoter regions than corresponding fourfold sites at ancestral nodes (12-351 genes, Additional file 1: Fig. S7b) and within species (29-352 genes, Additional file 1: Fig. S7d), indicative of small-scale changes in promoter regions. Given the lack of significant evolutionary rate in the majority of gene promoter regions, we hypothesize that discrete changes that could otherwise alter *cis*-regulatory binding sites, could drive gene expression variation in the five cichlids.

Owing to the discrete nucleotide variations observed in various regulatory regions, including selected promoter regions (Additional file 1: Fig. S7), we expect that some of the variation may occur at TFBSs (Additional file 1: Fig. S3). We identified several pairwise variants between the five cichlids that overlap various genomic regions (Table S-R2a), including state-changed and non-state changed gene promoters and 3’ UTRs (Additional file 1: Fig. S8). A large proportion of pairwise species variants (12 to 25 million) overlap predicted TFBSs in promoter regions, constituting 14-22% of all pairwise variants in the five species (Table S-R2a, Additional file 1: Fig. S8). GO enrichment analysis of cichlid pairwise variants overlapping gene regulatory regions highlight associations with key molecular processes e.g. signal transduction - non-state changed promoter TFBSs (Additional file 1: Fig. S9).

These findings imply that discrete nucleotide variation at regulatory binding sites could drive functional gene co-expression variation in cichlids through GRN rewiring events.

We focused on regulatory interactions with DNA, most prominently the identification and analysis across species of TF binding to gene promoters (Table S-R3a) as 1) a large proportion of all pairwise species variation (14-22%) overlap TFBSs (Table S-R2a, Additional file 1: Fig. S8) and hence, disrupted binding sites will offer insights into GRN rewiring between species; 2) gene orthology is well characterized (as opposed to other regulators, like miRNAs); and 3) direct correlation to tissue co-expression patterns can be made (Fig. 1a). We used a few metrics to study large-scale network rewiring between species, including the analyses of 1) state changes in module assignment; and 2) rewired network edges based on DyNet[12] network rewiring scores (see *Methods*). We first focused on 6,844 1-to-1 orthologous genes in 215,810 TF-TG interactions, termed 'TF-TG 1-to-1 edges', along the five cichlid tree. In total, we identify 4,060-9,423/215,810 TF-TG 1-to-1 edges that are rewired (in a focal vs other species) along the cichlid tree (FDR<0.05, Fig. 2a), and linked to module assignment state changes of 50-70 out of 379 TFs. Given that the level of statistical significance applied (FDR<0.05) could include all 4,060-9,423 (2-4%) rewired and TF state-changed edges, we further analysed the drop-out over more stringent thresholds. Analyses at more stringent thresholds (than FDR<0.05) maintain a similar number of rewired edges ranging from around 1% retained (FDR<0.01) to 2.3% retained (FDR<0.04) and thus, very few rewired edges are likely to be false positives. In the 215,810 TF-TG 1-to-1 edges, we identify 31 out of 90 teleost and cichlid trait genes associated with morphogenesis from previous studies (Additional file 3: Table S2) that have rewired GRNs based on their DyNet [12] degree-corrected rewiring (D_n) score (Additional file 3: Table S1). A total of 9 out of 31 morphogenesis genes have a few standard deviations higher degree-corrected rewiring (D_n) score than the mean (0.17±0.03 SD) score of all 1-to-1 orthologs (Fig. 2c – left violin plot, orange dots; Additional file 3: Table S3). Furthermore, the degree-corrected rewiring (D_n) score of these nine genes (Fig. 2c – left violin plot, orange dots) is

significantly higher (Kolmogorov–Smirnov KS-test *p-value* = 0.0006) and thus, exhibit more rewired edges compared to rewired 1-to-1 ortholog edges (Fig. 2c – left violin plot, black dots). Examples of the nine genes include *gdf10b* associated with axonal outgrowth and fast evolving in cichlids [13]; *rh2* – a visual opsin gene[11]; *draxin* – a neural development gene under selection in deepwater cichlid species [14]; and *cntn4,* also associated with neural development and fast evolving in cichlids [13] (Fig. 2c – left violin plot; Additional file 3: Table S3). To also study rewired networks of orthologs not shared along each taxon of the five cichlid tree, we extended our analyses beyond focusing on 6,844 1-to-1 orthologs only, by also including 7,746 many-to-many orthogroups (see *Methods*) in a set of 843,168 'TF-TG all edges' across the five species. In these edges, we identify 89 out of 90 teleost and cichlid trait genes associated with morphogenesis from previous studies (Additional file 3: Table S2) that have rewired GRNs based on their DyNet [12] degree-corrected rewiring (D_n) score (Additional file 3: Table S1). A total of 60 out of the 89 morphogenesis genes have a few standard deviations higher degree-corrected rewiring (D_n) score than the mean (0.23±0.007 SD) score of all orthologs (Fig. 2c – right violin plot, orange dots; Additional file 3: Table S5). Furthermore, the degree-corrected rewiring (D_n) score of these 60 genes (Fig. 2c – right violin plot, orange dots) is significantly different (KS-test *p-value* = 6e-14) and thus, exhibit more rewired edges compared to the rewiring of all ortholog edges (Fig. 2c – right violin plot, black dots). These genes include most visual opsins e.g. *rho, sws2* and *sws1* [11]; genes associated with photoreceptor cell differentiation, *actr1b*[15]; eye development, *pax6a* [1]; and neuro- and retino- genesis, *irx1* [16,17] (Fig. 2c – right violin plot; Additional file 3: Table S5).

Since cichlid adaptive radiations extend far beyond the five species we study here, we extended our analyses to include radiating lake species data. In this analysis, we link to previous studies and resources made available [18] to genotype our variants and study how they segregate in the Lake Malawi phylogeny. We overlapped all identified TFBS variants

between *M. zebra* (a Lake Malawi species) and the other four cichlids, onto corresponding positions of variants identified in a 73 Lake Malawi species (134 individuals) genome alignment [18]. Using *M. zebra* genotypes as a reference, the expectation would be that more variants would exist with different lake species (like *N. brichardi* from Lake Tanganyika) as well as distantly related same lake species (from Lake Malawi), than closely related same lake species. Of the total 5710 identified variants, the mean number of different genotypes at corresponding positions (vs *M. zebra*) is higher at distant (Lake Tanganyika – 4278; Rhamphochromis – 1756; Diplotaxadon – 1968; Shallow benthic – 1758) than at closely related (*A. calliptera* – 1674) or same (Mbuna – 1540) clade species (Additional file 1: Fig. S11). This analysis formed the basis for focusing on particular variants that can be associated with traits under selection e.g. visual systems[11] (*sws1)* and morphogenesis[13] (*cntn4*); to ultimately study variants in TFBSs that segregate according to phylogeny and ecology of radiating lake species.

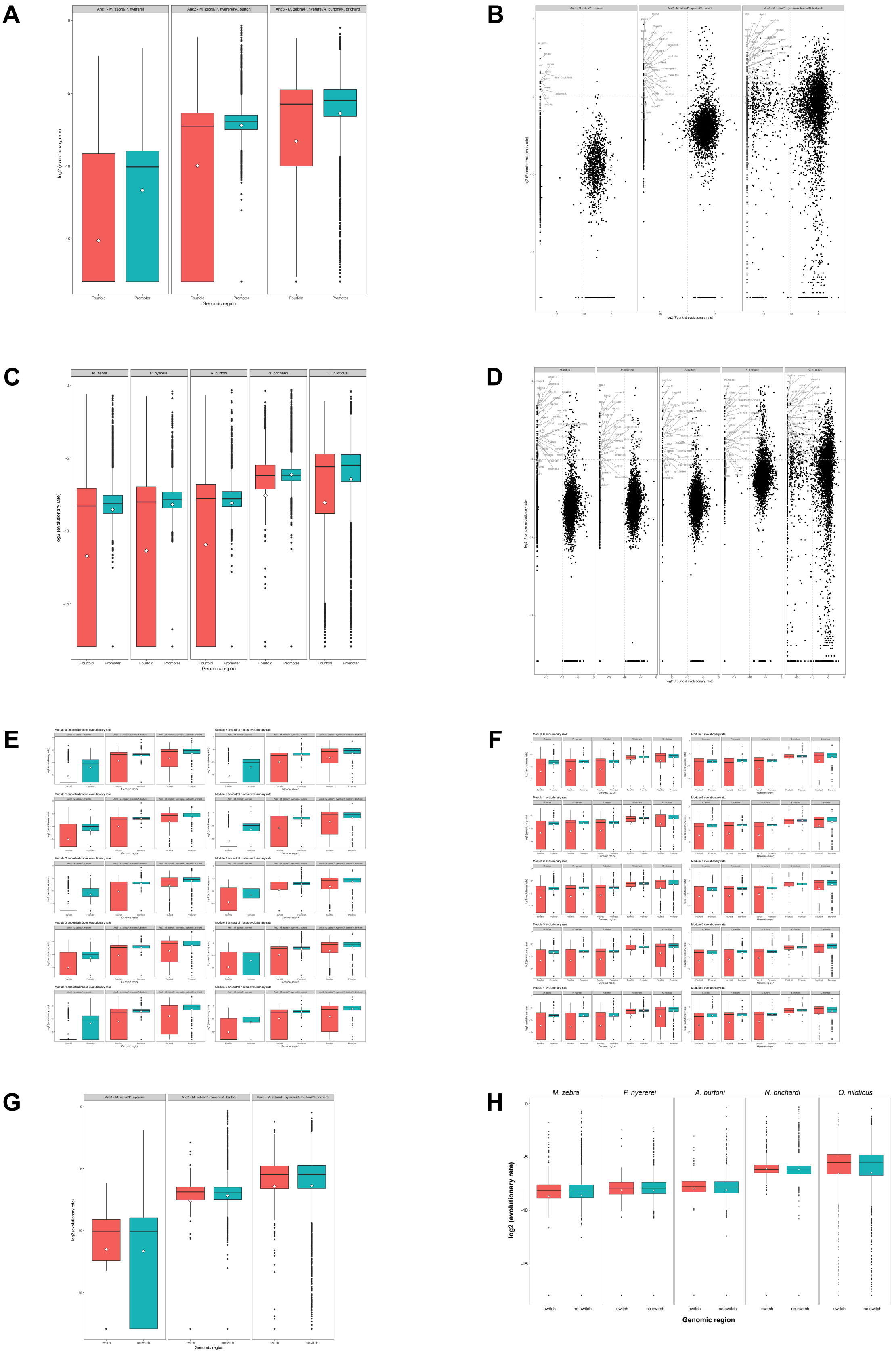

Fig. S7 – Evolutionary rate at cichlid promoter regions. (A) Boxplot of *log2* evolutionary rate in promoters (green bars, *right*) and fourfold degenerate sites (red bars, *left*) of 1:1

orthologous cichlid genes at each ancestral node; **(B)** Dot plot of evolutionary rates at promoter and fourfold degenerate sites of 1:1 orthologous cichlid genes at each ancestral node. Boundaries (grey dotted line) and top 30 outliers genes with high log2 promoter evolutionary (>-5) and low log2 fourfold site rate (<-10) are marked within; **(C)** Boxplot of *log2* evolutionary rate in promoters (green bars, *right*) and fourfold degenerate sites (red bars, *left*) of 1:1 orthologous cichlid genes at each branch; **(D)** Dot plot of evolutionary rates at promoter and fourfold degenerate sites of 1:1 orthologous cichlid genes at each branch. Boundaries (grey dotted line) and top 30 outliers genes with high log2 promoter evolutionary (>-5) and low log2 fourfold site rate (<-10) are marked within; **(E)** Boxplot of *log2* evolutionary rate in promoters (green bars, *right*) and fourfold degenerate sites (red bars, *left*) of co-expressed 1:1 orthologous cichlid genes at each ancestral node; **(F)** Boxplot of *log2* evolutionary rate in promoters (green bars, *right*) and fourfold degenerate sites (red bars, *left*) of co-expressed 1:1 orthologous cichlid genes at each branch; **(G)** Boxplot of *log2* evolutionary rate in promoters regions of state-changed/switching (red bars, *left*) and non-state changed/non-switched (green bars, *right*) 1:1 orthologous cichlid genes at each ancestral node (switches against LCA, as in Fig. R1B); **(H)** Boxplot of *log2* evolutionary rate in promoters regions of state-changed/switching (red bars, *left*) and non-state changed/non-switched (green bars, *right*) 1:1 orthologous cichlid genes at each branch (state changes against all other species, as in Fig. 1B).

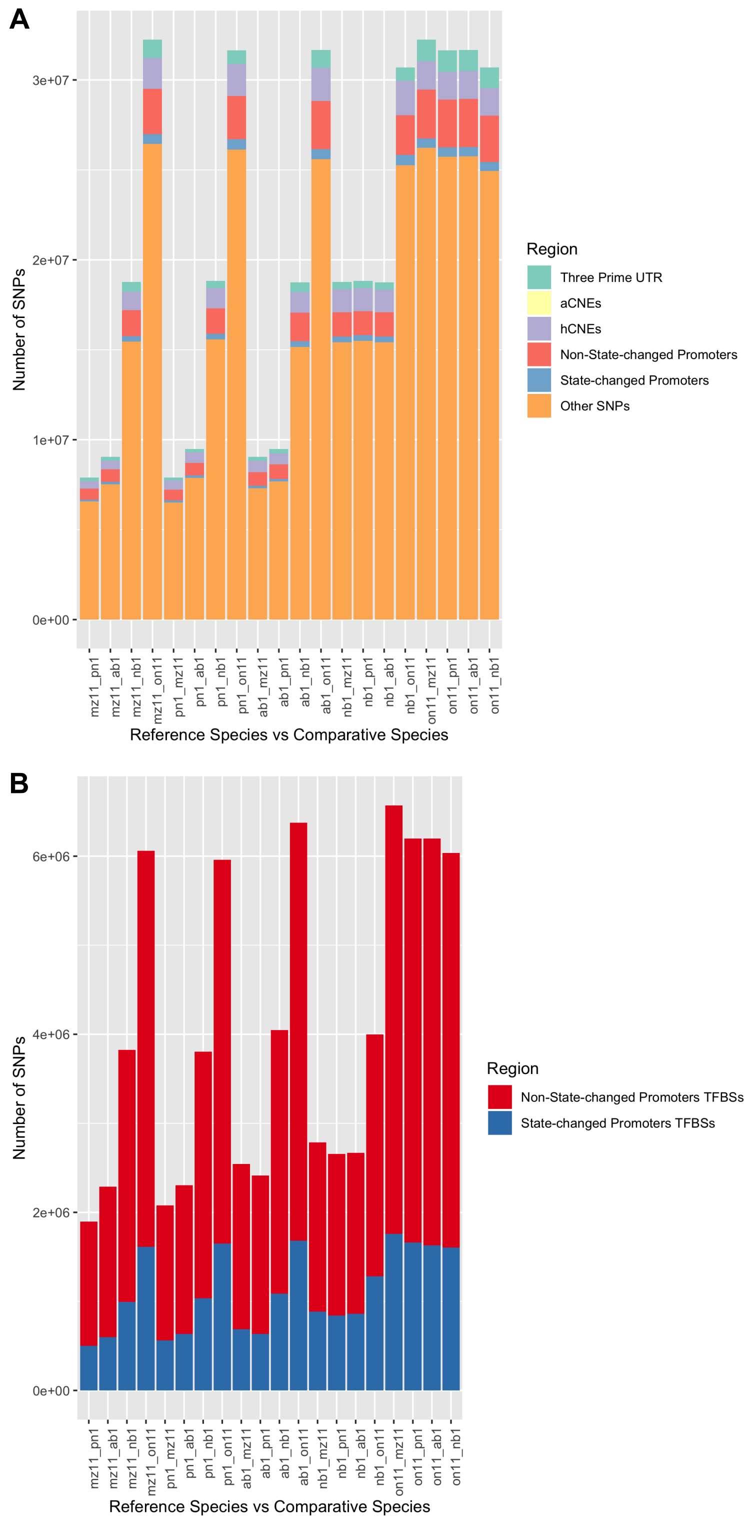

Fig. S8 – Pairwise variants overlapping various regulatory regions between the five cichlids. (a) Number of variants (y-axis) overlapping regulatory regions (colored bars) derived from pairwise comparisons (x-axis). Species are named accordingly: *M. zebra* (mz11); *P. nyererei* (pn1); *A. burtoni* (ab1); *N. brichardi* (nb1); and *O. niloticus* (on11). **(b)** Number of pairwise SNPs overlapping non- and state-changed promoter TFBSs.

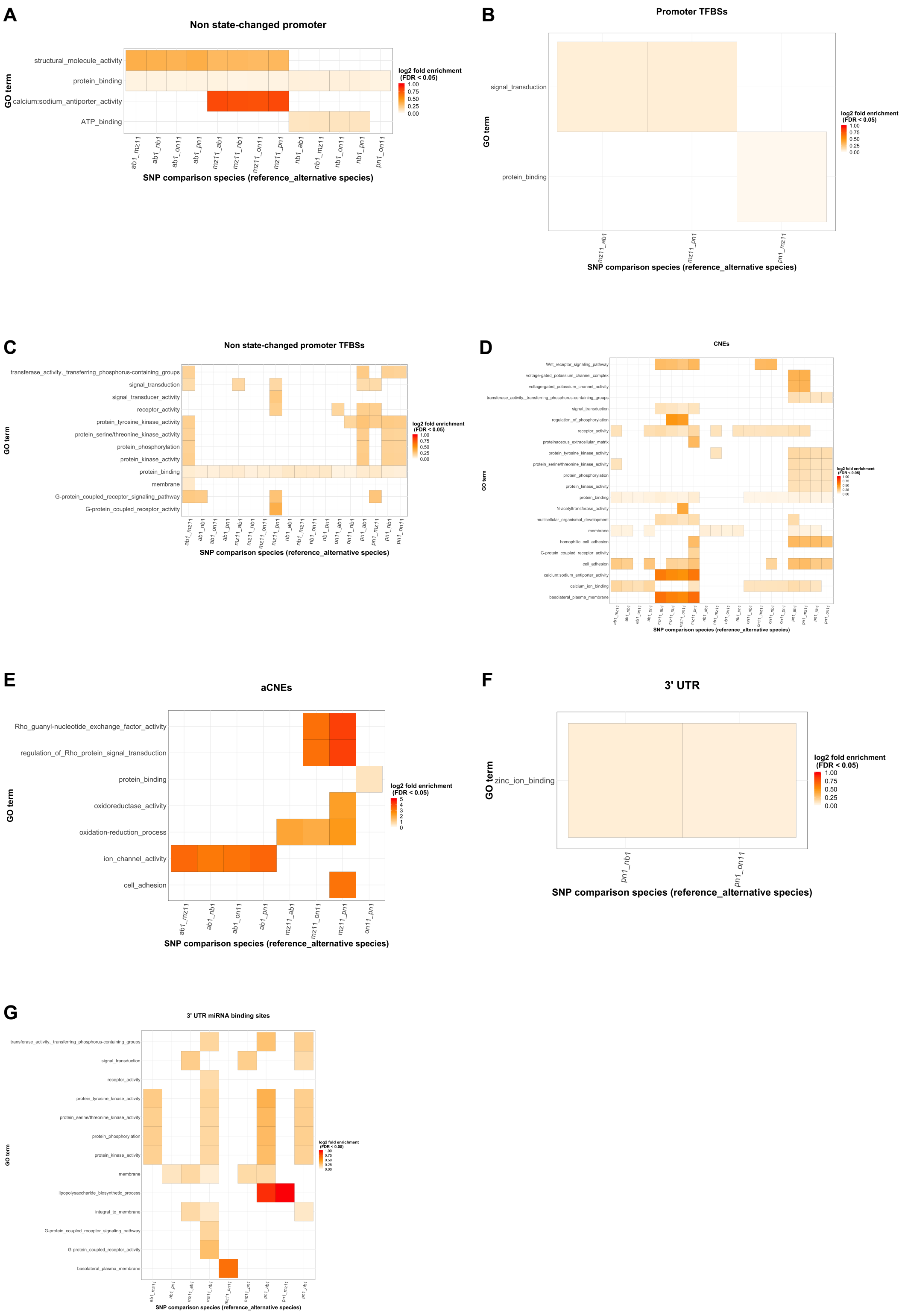

Fig. S9 – Gene Ontology (GO) enrichment of pairwise variants overlapping various regulatory regions between the five cichlids. Enriched terms (y-axis) shown as grid heatmap of *log10* fold enrichment (legend on *right*, FDR <0.05) of pairwise species comparisons (x-axis). Set-based hypergeometric test of enrichment carried out using a

background of all genes in each species genome. No significant (FDR< 0.05) enrichment in ‘state-changed promoters’ and ‘state-changed promoter TFBSs’.

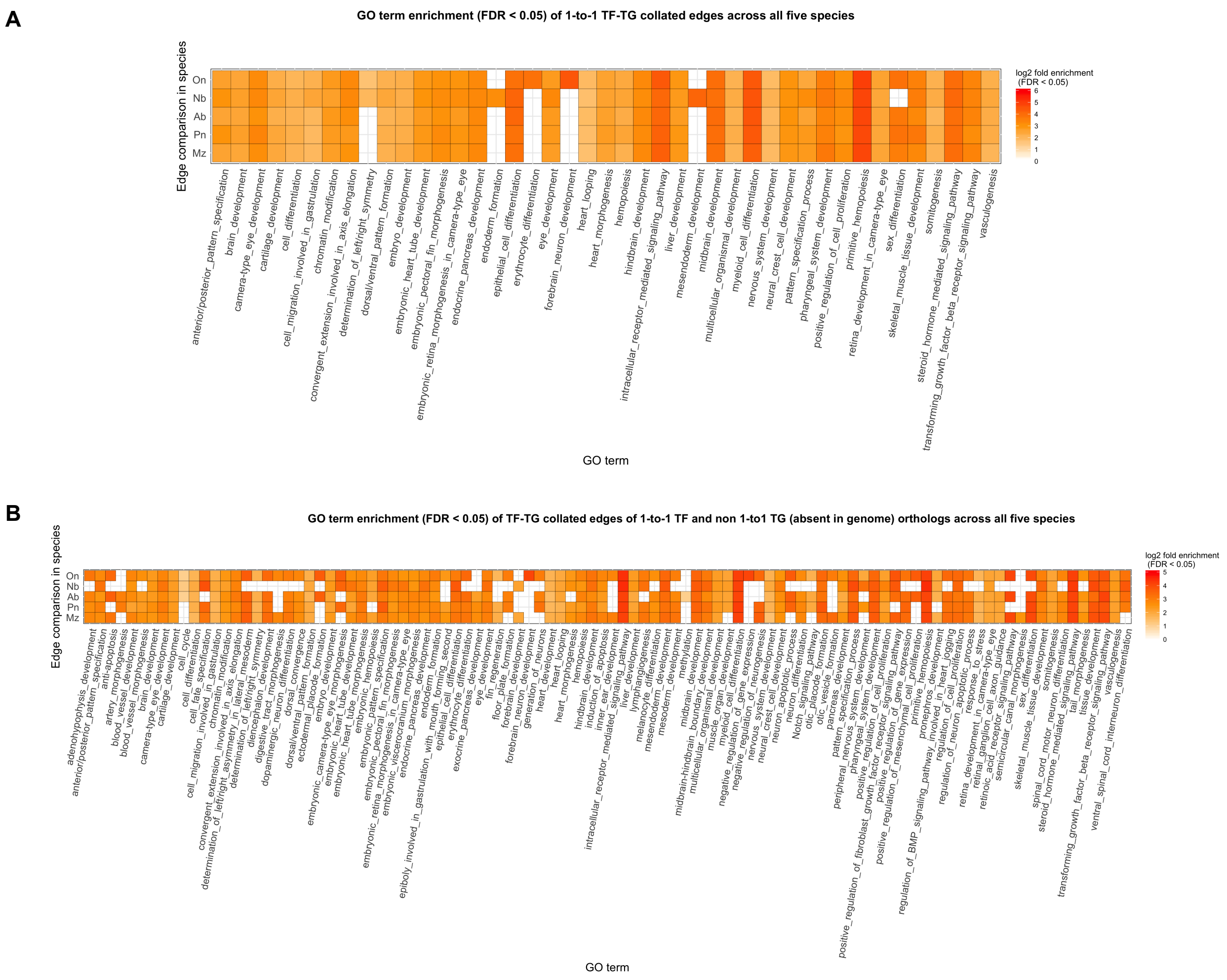

Fig. S10 - Gene Ontology (GO) enrichment of collated network edges. GO enrichment shown for both transcription factors (TF) and target genes (TG) in network edges of **(A)** 1-to-1 TF-TG; and **(B)** 1-to-1 TF and non-1-to-1 TG (absent in genome) orthologs in all five species.

Species (y-axis) and significant FDR-corrected *P*-value (*q*-value <0.05) GO terms (x-axis) where *log10* fold enrichment shown as grid heatmap (legend on *right*). Set-based hypergeometric test of enrichment carried out using a background set of all module genes (18,799 orthogroups).

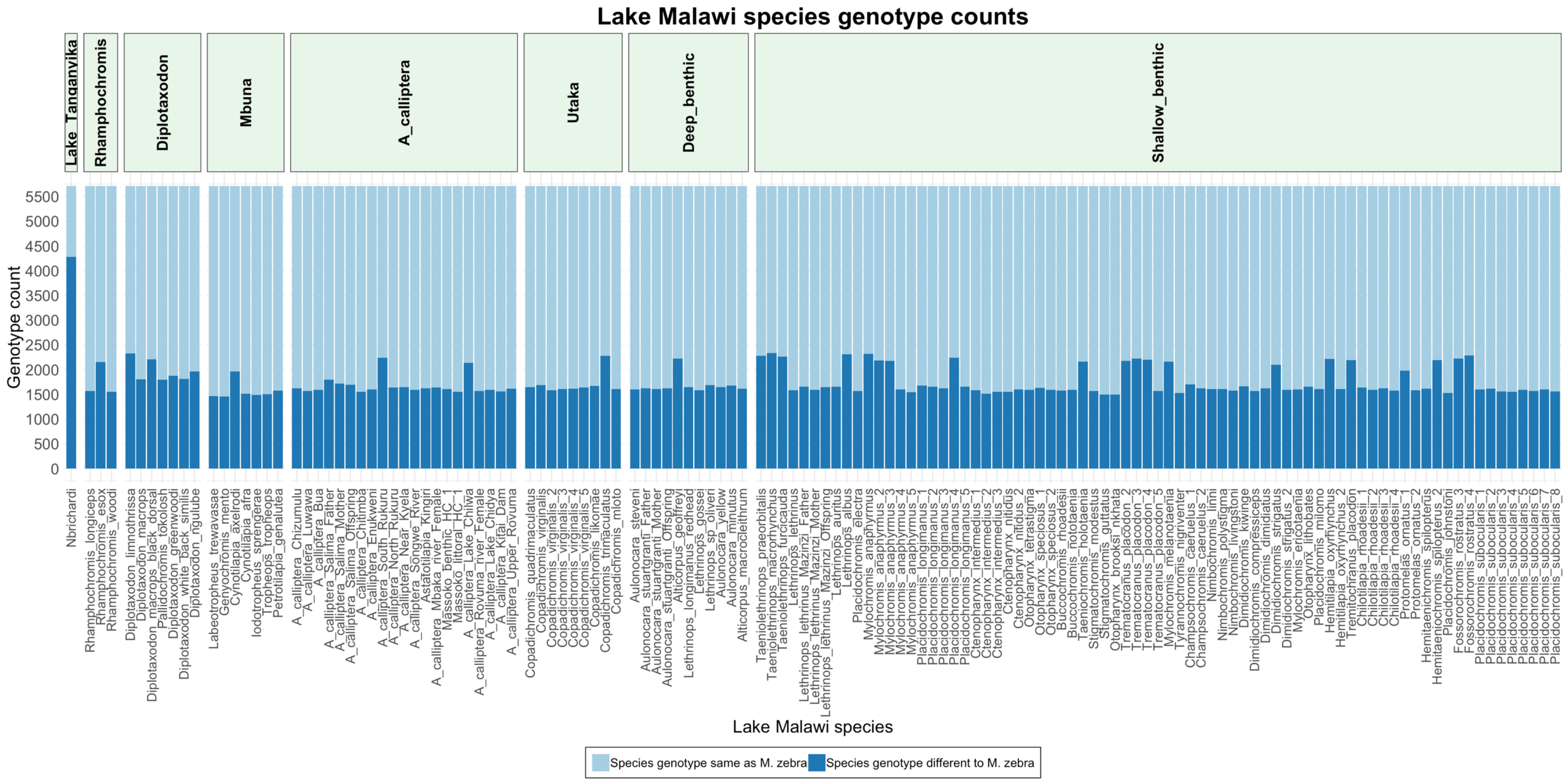

Fig. S11 - Pairwise variants in candidate gene promoter TFBSs that segregate or are conserved in *M. zebra* and Lake Malawi species. Genotype counts (y-axis) of conserved (light blue bars) and diverged (dark blue bars) genotypes compared to *M. zebra* in Lake Malawi species (x-axis). Lake Malawi species are ordered and placed into clades according to the published ASTRAL phylogeny; the least controversial and lowest mean distance phylogeny that included all sampled species[18].

Cis-regulatory changes lead to GRN alterations that control traits linked to phylogeny and ecology of East African cichlid radiations

Sws1 (ultraviolet) opsin is utilized as part of the short-wavelength sensitive palette in *N. brichardi* and *M. zebra*. A comparison of the *sws1* network of both species identifies several common (18 TFs) and unique (*N. brichardi* – 6 TFs; *M. zebra* – 38 TFs) regulators (Fig. 3a). By ranking and plotting the significance (FDR<0.05) of the unique TF-*sws1* edges, we identify that there is a larger proportion of more significant unique regulators of *sws1* in *M. zebra* (18/38 TFs, orange dots less than mean, Fig. 3a *bottom right*) than *N. brichardi* (2/6 TFs, orange dots less than mean, Fig. 3a *bottom right*).

We focus on NR2C2 and RXRB owing to the significance of this unique predicted *sws1* edge interaction in *N. brichardi* (Fig. 3a, *bottom right*), and show that a candidate polymorphic site in *M. zebra sws1* gene promoter has likely disrupted binding of NR2C2 (Fig. 3). The candidate variant that has likely disrupted binding of NR2C2, and possibly regulation of *M. zebra sws1* (Fig. 3) is an outlier homozygous SNP (A|A) when compared to all other four species (*P. nyererei, A. burtoni, N. brichardi* and *O. niloticus*) that have the homozygous G/G genotype (negative orientation). The outlier homozygous SNP in *M. zebra* as well as flanking sequence predicted as the *M. zebra sws1* gene promoter and used for EMSA validation (Fig. 3c-d) is 100% conserved in the recently published chromosome-scale *M. zebra* assembly [19,20].

We accept that the EMSA binding validation (Fig. 3) does not provide evidence for disrupted regulation of *sws1* alone. To address the point of whether NR2C2 are contributing towards regulatory network rewiring, we sought to use our expression data to first predict the regulators for *sws1* by using a regression model. For this, we used expression data from all tissues and species to predict the regulatory relationship based on the co-variation of a TF's

expression level with a TG's expression level across tissues and species (n=30 measurements). Using this we established a "skeleton" network that predicts the potential regulators of *sws1*; among the top regulators of *sws1* were vision-related regulators such as VSX2[21] and NRL[22].

We next compared these predicted expression-based regulators to see the overlap with the motif based (*cis*) regulators however, there was low overlap between the different networks. This is not surprising and we would expect improved overlap upon the inclusion of more tissues (for expression data) and further species-specific motif information derived from epigenetic techniques e.g. ChIP-seq. The regulators that did overlap however, were among the top regulators, namely, TBX4 (confidence 0.9). We next assessed the correlation of the top regulators predicted by expression, including TBX4, NRL, NR2C2 and RXRB to the expression of *sws1* based on three criteria: (a) global correlation (gcc) using all tissues and species (Additional file 1: Fig. S16a *on left*, Additional file 1: Fig. S16b – first column, *gcc*), (b) tissue-specific correlation asking to what extent these dependencies are preserved based on eye-specific correlation (Additional file 1: Fig. S16a *on right*, Additional file 1: Fig. S16b), (c) species-specific correlation (Additional file 1: Fig. S16c). In the tissue-specific expression, we can think of each species as a pseudo knockdown/upregulation experiment of the regulator. If the cross-species variation of expression of the regulator is predictive of the variation in the target, we can conclude that the edge might be rewired because the strength of regulation varies. Based on global correlations, we see that TBX4 and NRL are well-correlated to the expression of *sws1*. Both NR2C2 (and RXRB) have relatively lower correlation, but they rank comparably to the expression-based regulators (Additional file 1: Fig. S16a *on left*, Additional file 1: Fig. S16b – first column, *gcc*), and can therefore explain some of the variation of *sws1*. Focusing on eye-specific correlation only (Additional file 1: Fig. S16a *on right*, Additional file 1: Fig. S16b – second column, *cc*), we see that the expression of TBX4 across species is predictive *of sws1* expression. NRL is not as predictive, largely due to relatively lower expression in N. brichardi, despite a high expression of *sws1*. Whilst both NR2C2 and RXRB are negatively correlated in the eye,

NR2C2's profile is more correlated compared to RXRB – supportive of our functional validations (Fig. 3). Finally, focusing within each species (Additional file 1: Fig. S16c), we find that RXRB's expression is not predictive *of sws1* in N. brichardi (Pearson CC=~0), but is more correlated in *M. zebra* (Pearson CC=0.46, n=6, p-value=0.4). In contrast, NR2C2 is correlated to *sws1* expression in both *M. zebra* (Pearson CC=0.30, n=6, p-value=0.6) and *N. brichardi* (Pearson CC=0.27, n=6, p-value=0.6). This suggests that, in *M. zebra*, based on expression either NR2C2 or RXRB could regulate *sws1* but the correlation is weak, whilst RXRB's correlation is slightly higher. However, in *N. brichardi*, only NR2C2 could to regulate *sws1*, supportive of our functional validations (Fig. 3).

We ran a similar analysis for the *rho* target gene, considering CRX and VSX2 as the top expression regulators, and the duplicated TFs, GATA2A and GATA2 (Additional file 1: Fig. S17) that are predicted to regulate in selected species (see *Main Text*). Based on global correlations, both GATA2A and GATA2 rank among the expression-based regulators with positive correlation (Additional file 1: Fig. S17a) and thus, have the potential to regulate the expression of *rho*. Focusing on the correlations in eye, we see that whilst both GATA2A and GATA2 have a negative correlation, GATA2A ranks better than GATA2 (Additional file 1: Fig. S17a, Additional file 1: Fig. S17b – second column, *cc*). The species-specific correlations are most informative for this regulatory edge (Additional file 1: Fig. S17c). We find that in *O. niloticus* and *A. burtoni,* GATA2A is positively correlated (0.79 and 0.21, respectively), however, in *M. zebra*, where the GATA2A edge is lost (Additional file 1: Fig. S14), GATA2A has a negative correlation. GATA2 is still positively correlated (Additional file 1: Fig. S14), supportive of its predicted regulation of *rho* in *M. zebra, A. burtoni* and *O. niloticus* (Additional file 1: Fig. S14). These species-specific correlations are therefore supportive of GATA2's possible conserved role in all three species, while a more divergent role and binding (Fig. 4) of GATA2A.

To summarise, our expression analysis suggests that the functional binding validations of selected *cis*-regulators, NR2C2 and GATA2A rank comparably among the expression-based regulators and therefore have regulatory potential. NR2C2 is more likely to regulate *sws1 in N. brichardi* compared to RXRB, while both could be plausible in *M. zebra* but still weakly correlated. Given the role of NR2C2 in nuclear receptor signalling [23], important for eye development/function and ability to enhance or repress gene expression in response to environmental cues [24], we suspect an important role in opsin gene expression and cichlid visual system adaptation. GATA2 exhibits a conserved positive correlation with *rho* in all three species with *rho* expression, however, GATA2A's correlation with *rho* in *M. zebra* is significantly lower than in *A. burtoni* and *O. niloticus*, suggesting it is likely not regulating *rho* in *M. zebra*. Taken together these results leverage the natural variation in expression across species and tissues to simulate a perturbation experiment and provide expression-based evidence of the regulatory connections of NR2C2>*sws1,* GATA2A>*rho* and GATA2>*rho*.

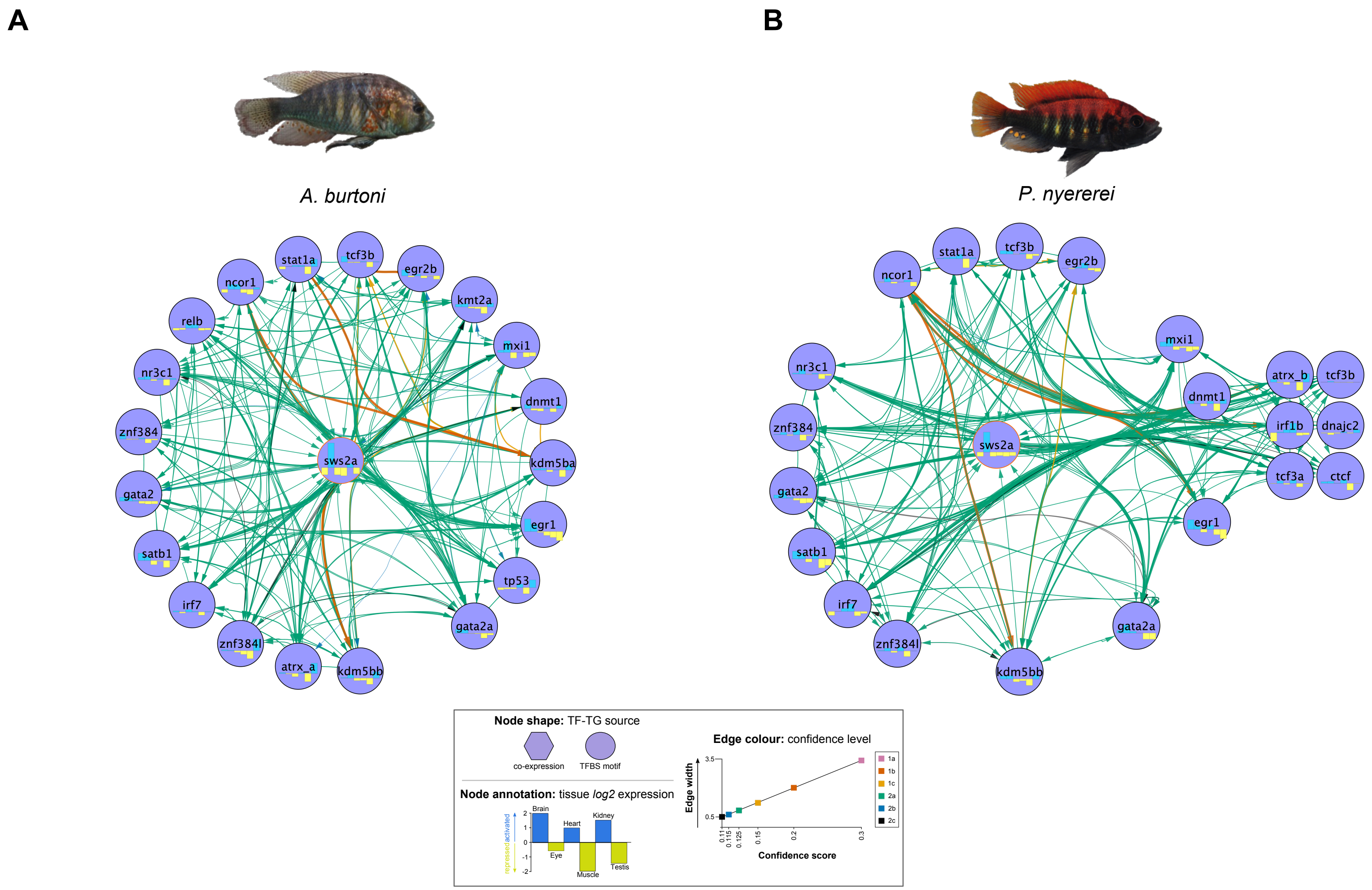

Fig. S12 - Evolution of the *sws2a* opsin regulatory networks in *A. burtoni* and *P. nyererei.* Regulatory networks of *sws2a* opsin shown for (A) *A. burtoni* and (B) *P. nyererei;* circular layout nodes are common regulators, grid layout nodes are unique regulators and node shape, annotation and edge color are denoted in legend.

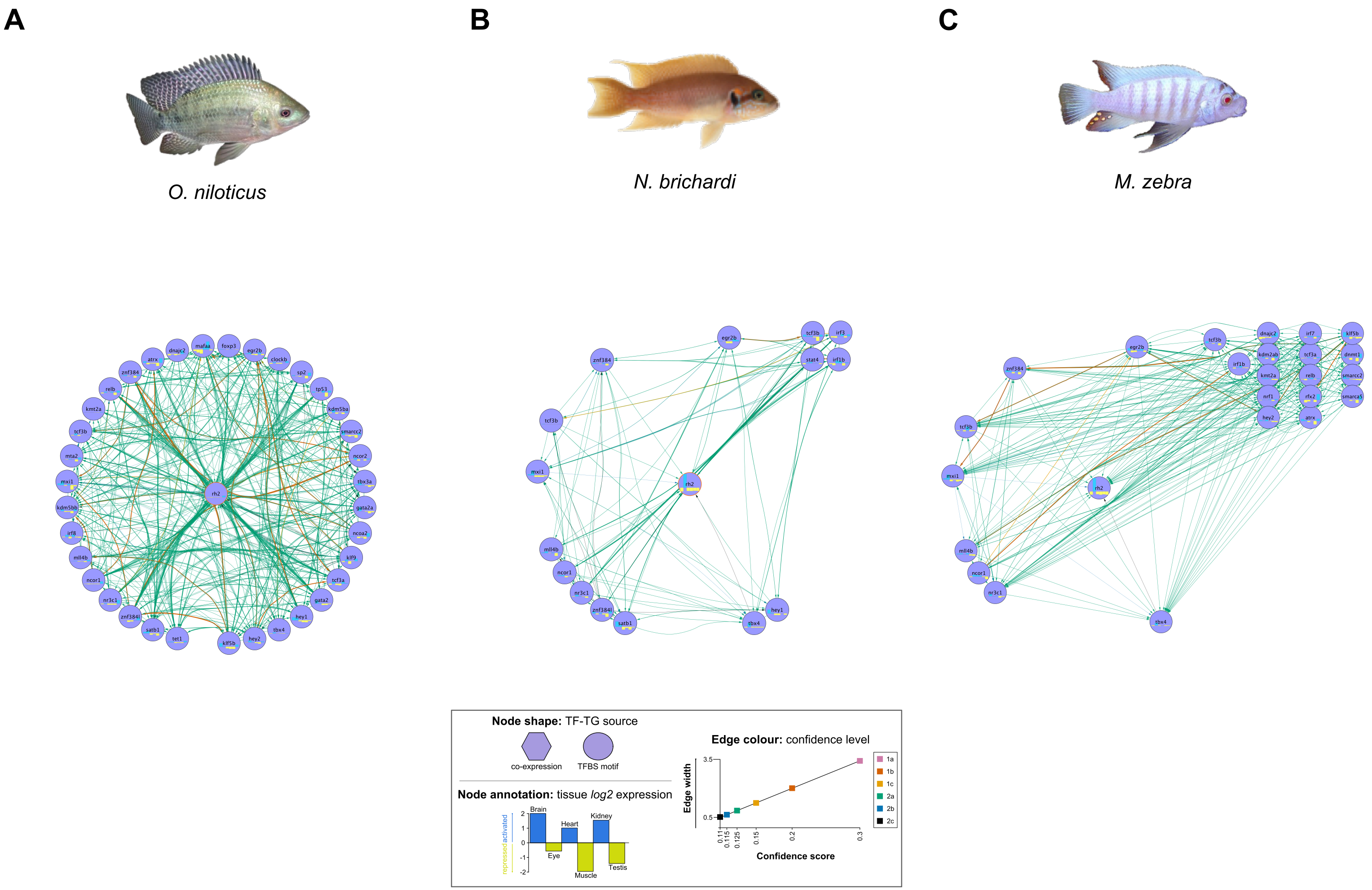

Fig. S13 - Evolution of the *rh2b* opsin regulatory networks in *O. niloticus, N. brichardi* and *M. zebra*. Regulatory networks of *rh2b* opsin shown for (A) *O. niloticus;* (B) *N. brichardi;* and (C) *M. zebra*; circular layout nodes are common regulators, grid layout nodes are unique regulators and node shape, annotation and edge color are denoted in legend.

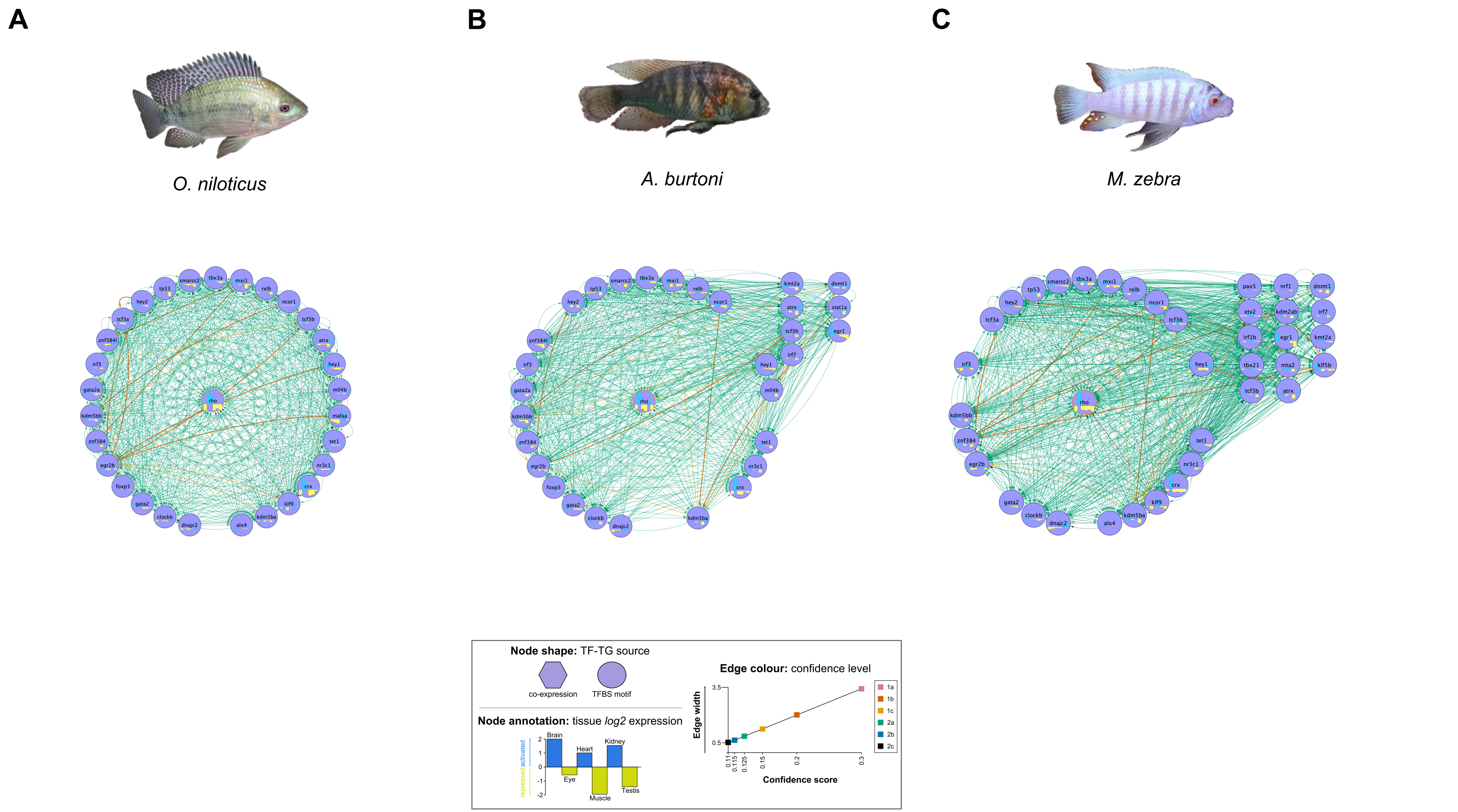

Fig. S14 - Evolution of the rod opsin regulatory networks in *O. niloticus, A. burtoni* and *M. zebra.* Regulatory networks of *rho* (rod) opsin shown for (A) *O. niloticus;* (B) *A. burtoni;* and (C) *M. zebra*; circular layout nodes are common regulators, grid layout nodes are unique regulators and node shape, annotation and edge color are denoted in legend.

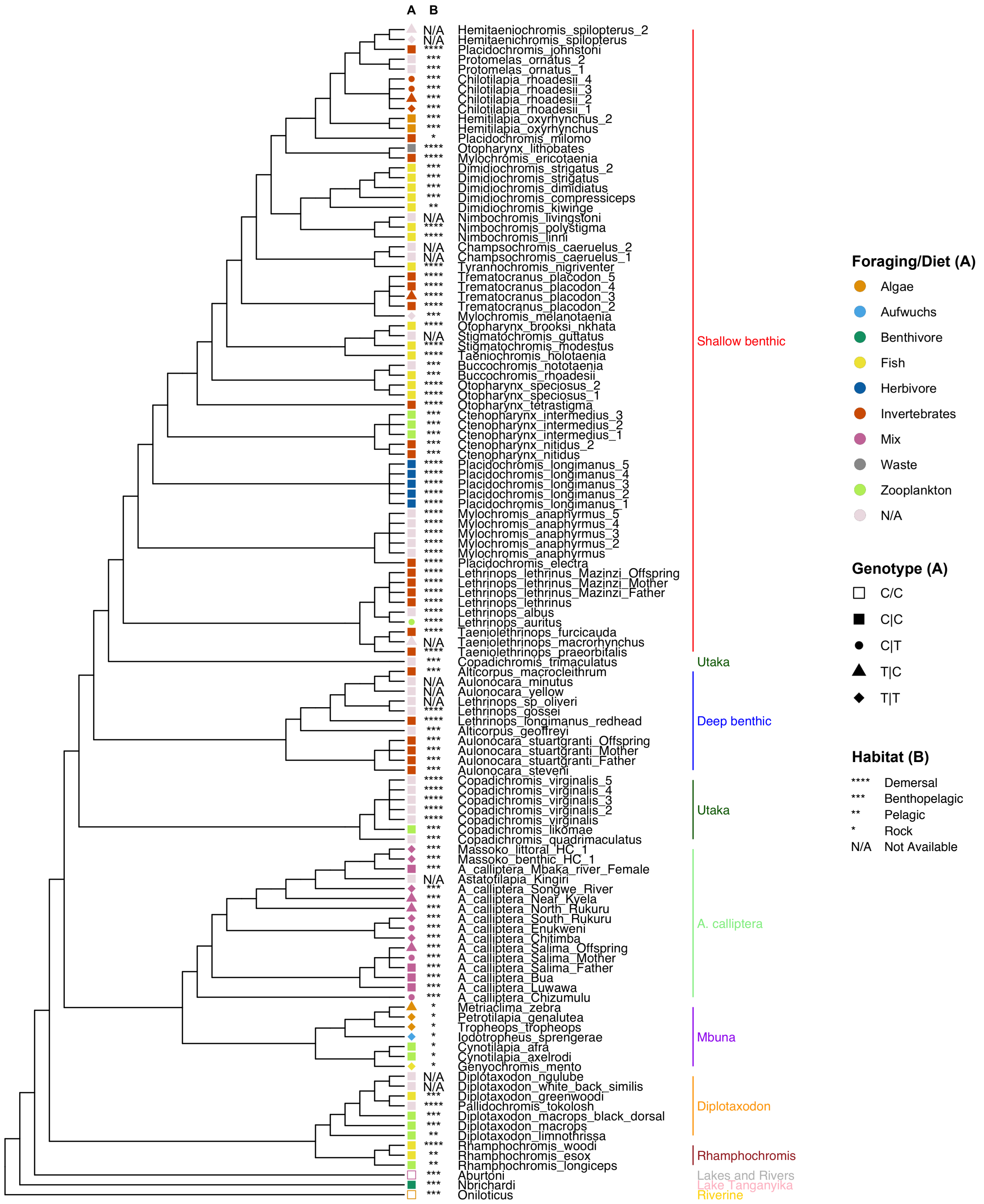

Fig. S15 - Variants overlapping the GATA2A TFBS in *M. zebra rho* promoter, other Lake Malawi species and *O. niloticus, N. brichardi* and *A. burtoni* outgroups. Lake Malawi phylogeny reproduced from published least controversial and all included species ASTRAL phylogeny [18]. Phylogenetic branches are labelled with species sample name and clade and according to legends (*right*): A) Species foraging/diet habit (color) [25] and phased SNP genotype (shape) [18]; B) species habitat [25,26].

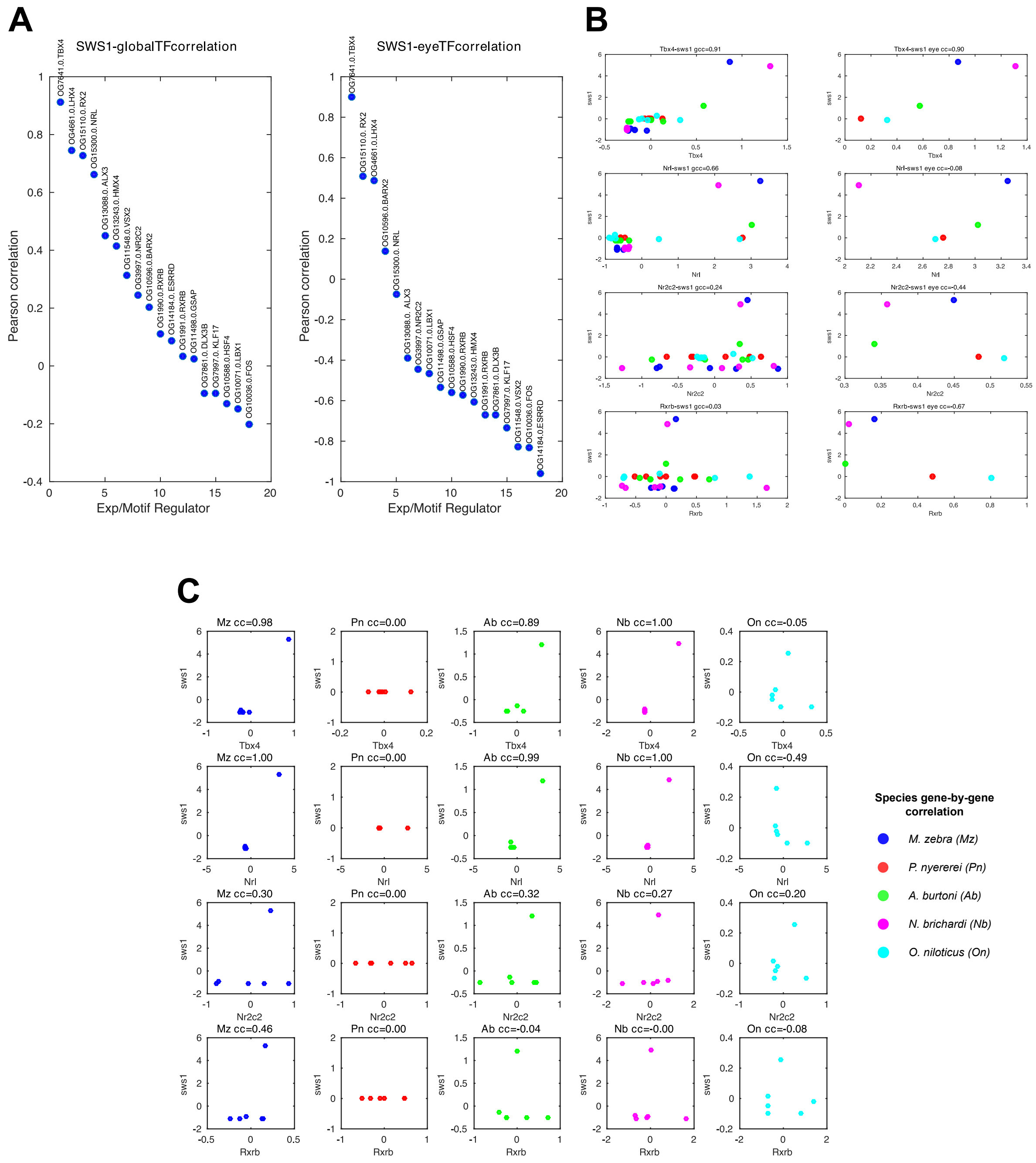

Fig. S16 – Global and eye-specific correlations of *sws1* and primary interacting TFs expression. (A) shows the Pearson correlation coefficient (y-axis) of all regulators expression separated by the expression of all species tissues (left) and eye tissue only (right). Scatter plots showing the expression level of the *sws1* gene (y-axis) and one of four possible regulators (x-axis), separated by **(B)** global tissue expression (left), eye expression (right) in all five species; and **(C)** expression in six tissues separated by each of the five species. Each dot corresponds to the expression of *sws1* and its regulator either in all tissues (Panel B, *left*; Panel C) or eye tissue only (Panel B, *right*). The Pearson's correlation coefficient (cc) between the regulator and *sws1*'s expression are mentioned in the title for panel B and C. For *sws1*, the first two sets of plots for TBX4 and NRL are among the top 2

regulators that were identified using our expression-based network inference approach. The remaining two rows, NR2C2 and RXRB were predicted based on motif scanning on the *sws1* gene promoter.

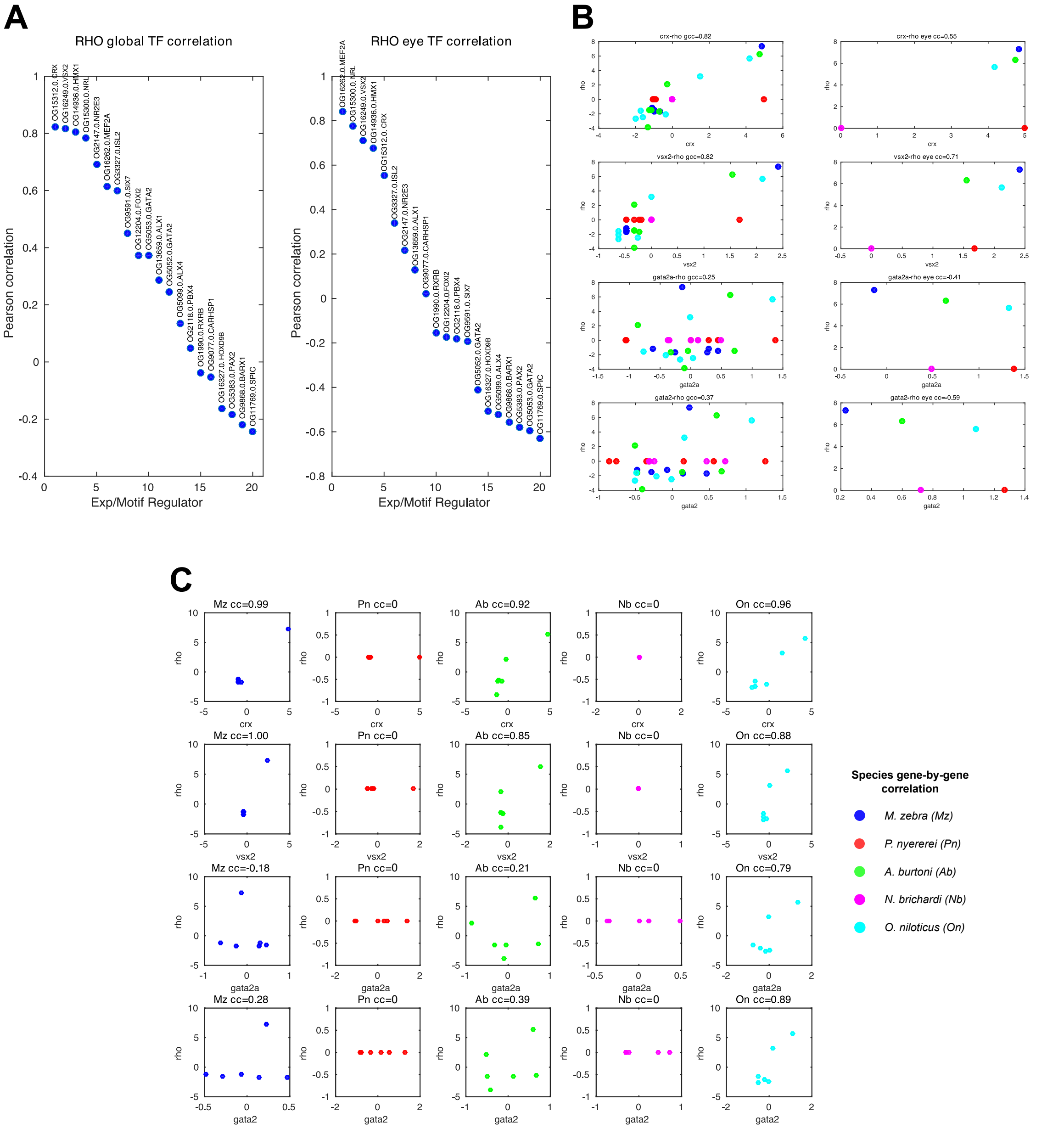

Fig. S17 – Global and eye-specific correlations of *rho* and primary interacting TFs expression. (A) shows the Pearson correlation coefficient (y-axis) of all regulators expression separated by the expression of all species tissues (left) and eye tissue only (right). Scatter plots showing the expression level of the *rho* gene (y-axis) and one of four possible regulators (x-axis), separated by **(B)** global tissue expression (left), eye expression (right) in all five species; and **(C)** expression in six tissues separated by each of the five species. Each dot corresponds to the expression of *rho* and its regulator either in all tissues (Panel B, *left*; Panel C) or eye tissue only (Panel B, *right*). The Pearson's correlation coefficient (cc) between the regulator and *rho*'s expression are mentioned in the title for panel B and C. For *rho*, the first two sets of plots for CRX and VSX2 are among the top 2 regulators that were identified using our expression-based network inference approach. The

remaining two rows, GATA2A and GATA2 were predicted based on motif scanning on the *rho* gene promoter.

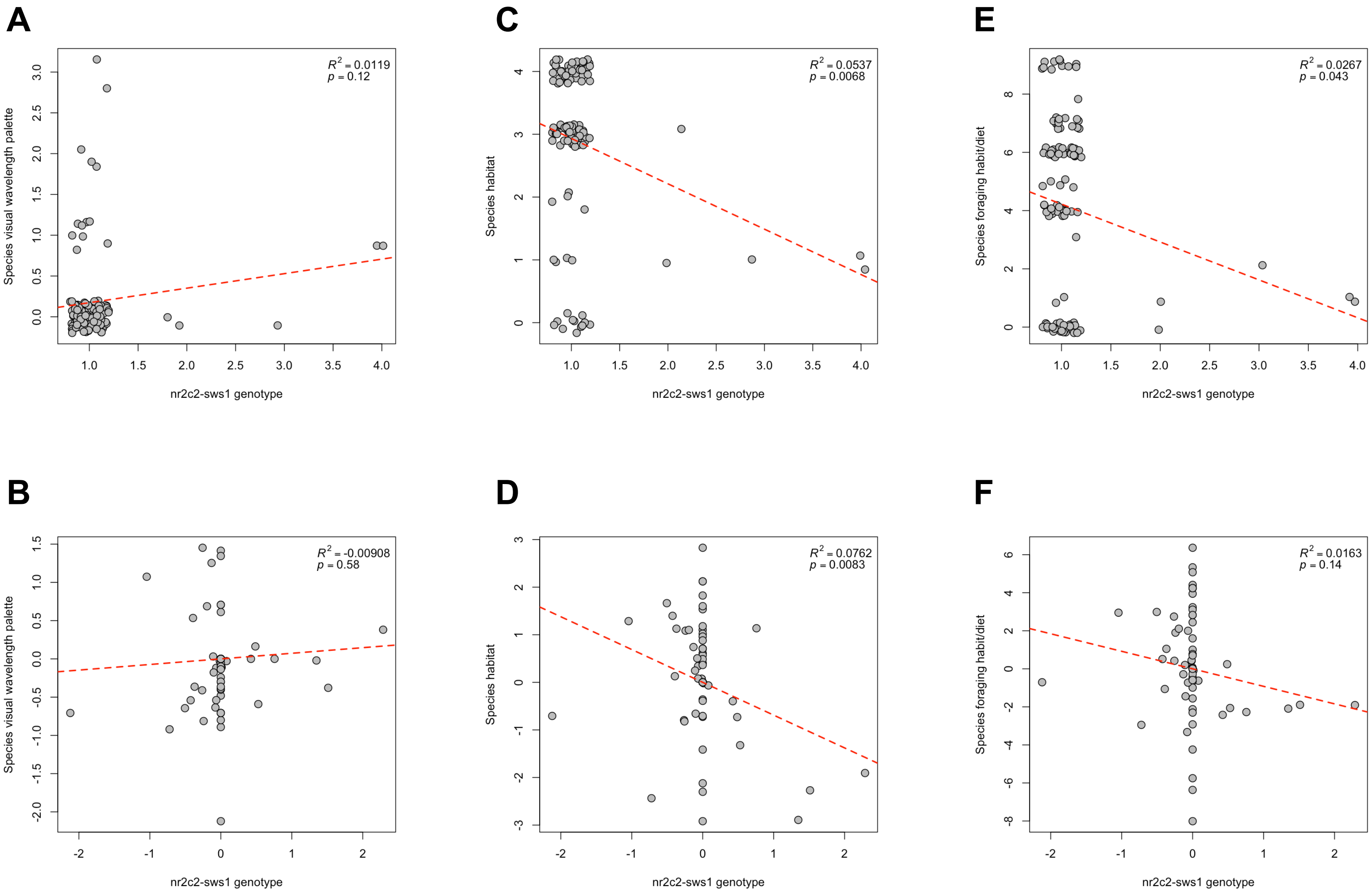

Fig. S18 – Phylogenetic independent contrast analysis of NR2C2-*sws1* TFBS genotypes of Lake Malawi species against their visual traits and ecology. Phylogenetic independent scatterplots of NR2C2-*sws1* TFBS genotypes (1=C|C, 2=C|T, 3=T|C, 4=T|T) in 119 Lake Malawi

individuals (73 species) against their respective **(a) visual wavelength palette** (0=N/A, 1=Short, 2=Medium, 3=Long); **(c) habitat** (0=N/A, 1=Rock, 2=Pelagic, 3=Benthopelagic, 4=Demersal); **(e) foraging habit/diet** (0=N/A, 1=Algae, 2=Aufwuchs, 3=Benthivore, 4=Fish, 5=Herbivore, 6=Invertebrates, 7=Mix, 8=Waste, 9=Zooplankton). Corresponding scatterplots of Lake Malawi ASTRAL phylogeny [18] and regression model fitted to NR2C2-*sws1* TFBS genotypes (1=C|C, 2=C|T, 3=T|C, 4=T|T) of 119 Lake Malawi individuals (73 species) against their respective **(b) visual wavelength palette** (0=N/A, 1=Short, 2=Medium, 3=Long); **(d) habitat** (0=N/A, 1=Rock, 2=Pelagic, 3=Benthopelagic, 4=Demersal); **(f) foraging habit/diet** (0=N/A, 1=Algae, 2=Aufwuchs, 3=Benthivore, 4=Fish, 5=Herbivore, 6=Invertebrates, 7=Mix, 8=Waste, 9=Zooplankton). All data points used as per Fig. 5, with overlapping coordinates 'jittered' around their respective point to highlight density. Adjusted r^2 and *p*-value of each regression line shown in top right of each plot.

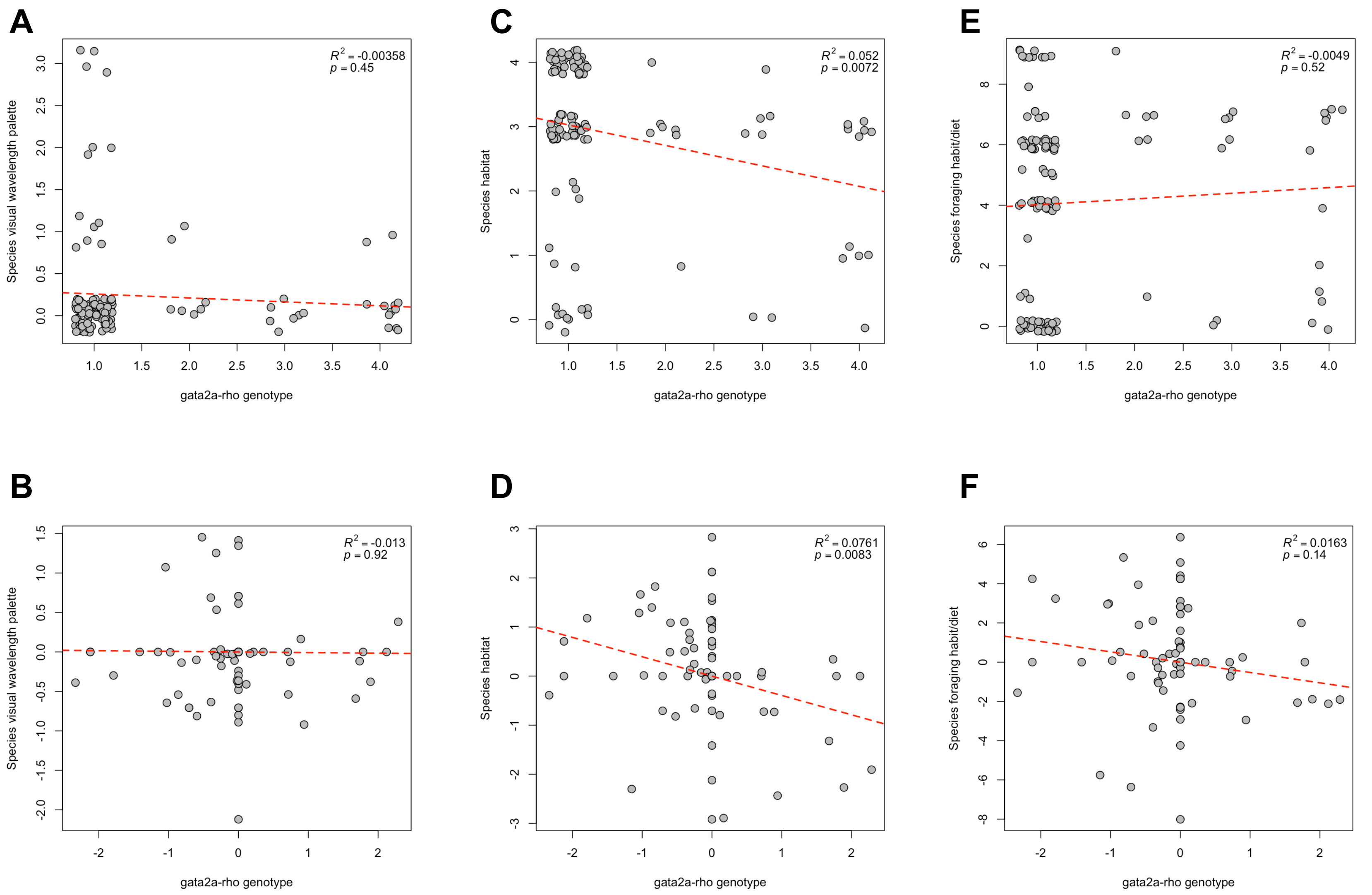

Fig. S19 – Phylogenetic independent contrast analysis of GATA2A-*rho* TFBS genotypes of Lake Malawi species against their visual traits and ecology. Phylogenetic independent scatterplots of GATA2A-*rho* TFBS genotypes (1=C|C, 2=C|T, 3=T|C, 4=T|T) in 119 Lake Malawi

individuals (73 species) against their respective **(a) visual wavelength palette** (0=N/A, 1=Short, 2=Medium, 3=Long); **(c) habitat** (0=N/A, 1=Rock, 2=Pelagic, 3=Benthopelagic, 4=Demersal); **(e) foraging habit/diet** (0=N/A, 1=Algae, 2=Aufwuchs, 3=Benthivore, 4=Fish, 5=Herbivore, 6=Invertebrates, 7=Mix, 8=Waste, 9=Zooplankton). Corresponding scatterplots of Lake Malawi ASTRAL phylogeny [18] and regression model fitted to GATA2A-*rho* TFBS genotypes (1=C|C, 2=C|T, 3=T|C, 4=T|T) of 119 Lake Malawi individuals (73 species) against their respective **(b) visual wavelength palette** (0=N/A, 1=Short, 2=Medium, 3=Long); **(d) habitat** (0=N/A, 1=Rock, 2=Pelagic, 3=Benthopelagic, 4=Demersal); **(f) foraging habit/diet** (0=N/A, 1=Algae, 2=Aufwuchs, 3=Benthivore, 4=Fish, 5=Herbivore, 6=Invertebrates, 7=Mix, 8=Waste, 9=Zooplankton). All data points used as per Supplementary Figure S-R4d, with overlapping coordinates 'jittered' around their respective point to highlight density. Adjusted r^2 and *p*-value of each regression line shown in top right of each plot.

Methods

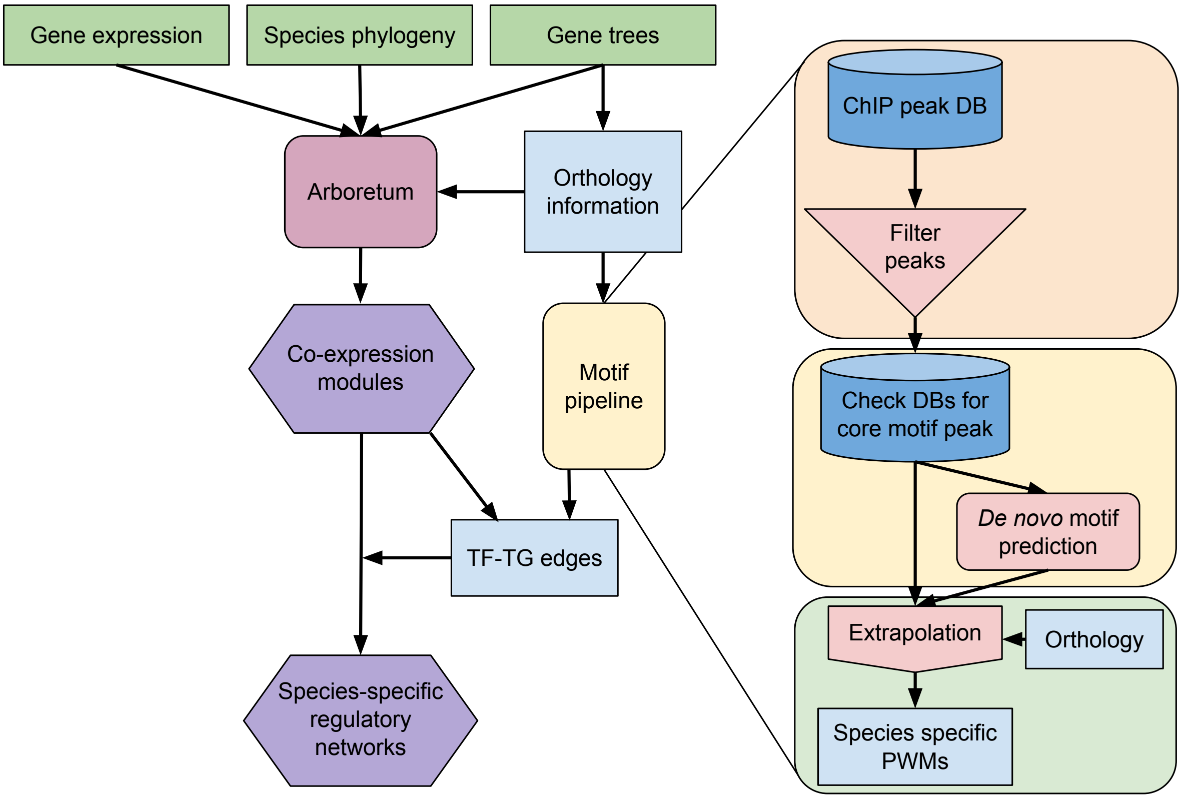

Fig. S20 - Systematic framework for reconstructing and analyzing gene regulatory networks in five cichlids. Our systematic framework comprises: (1) identifying modules of co-expressed genes from multi-tissue/multi-species and single-tissue/multi-species data using Arboretum[27]; (2) Using our developed motif prediction pipeline (*right)*, we integrate several datasets (co-expression, *cis* regulatory elements and transcription factor binding sites (TFBSs)) to; (3) reconstruct species-specific gene regulatory networks (GRNs) refined with gene expression data to find fine-grained tissue-specific network modules.

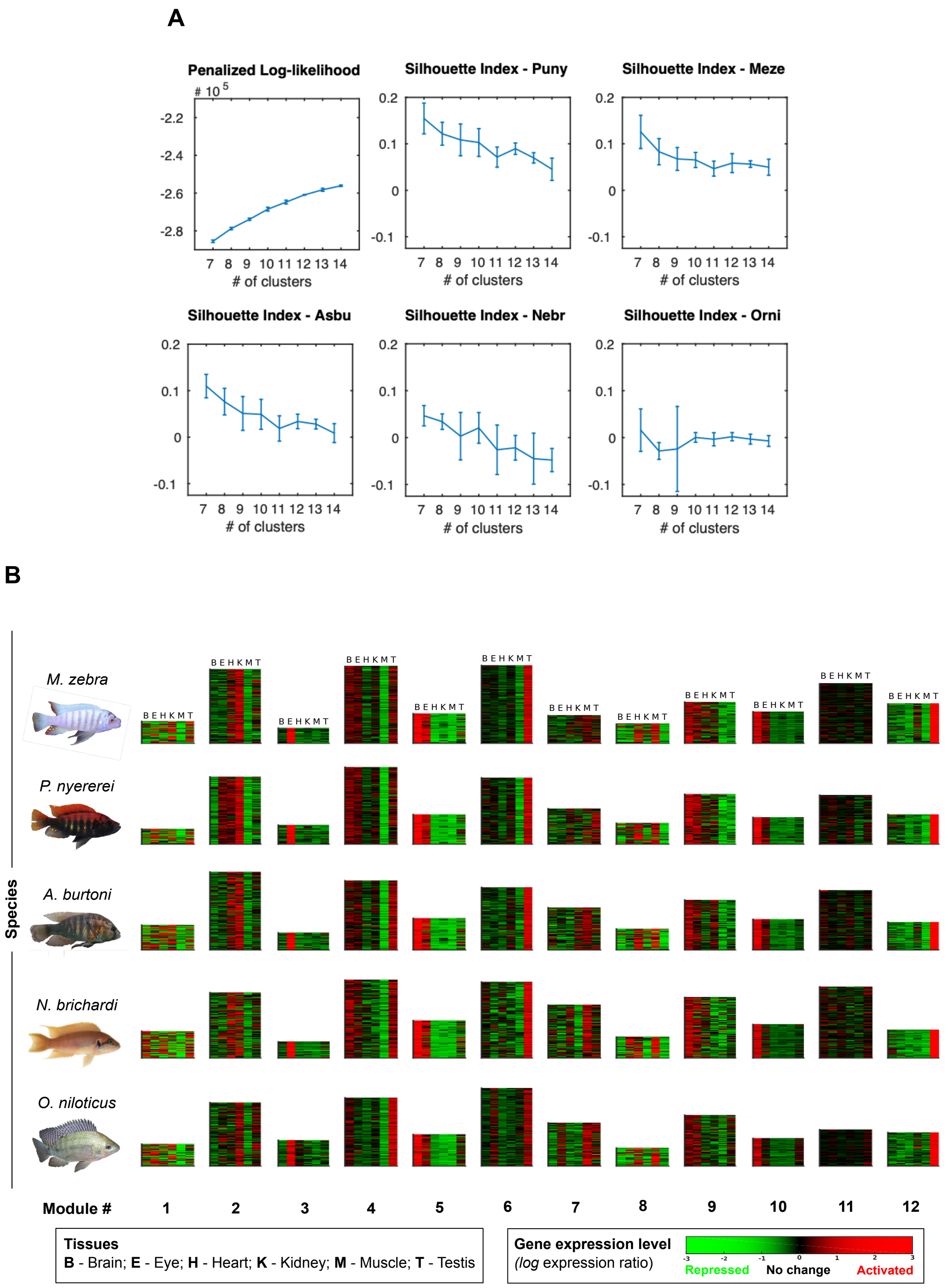

Fig. S21 – Inference of optimal number of multi-tissue transcriptional modules in five cichlids. (a) Penalized log likelihood and silhouette index for *k*=7-14 modules in increments of 1 for the five cichlid species (Puny=*P. nyererei;* Meze=*M. zebra;* Asbu=*A. burtoni;* Nebr=*N. brichardi;* Orni=*O. niloticus*). **(b)** k=12 (1-12, heatmaps) co-expression modules identified by Arboretum [27] in six tissues of five cichlid species. Colour bar denotes *log* expression ratio across each tissue, relative to the mean expression across all tissues - (red)

activated; (green) repressed; and (black) no change. Each heatmap shows the expression profile of genes assigned to that module in a given species and height is proportional to number of genes in the module (on *bottom*).

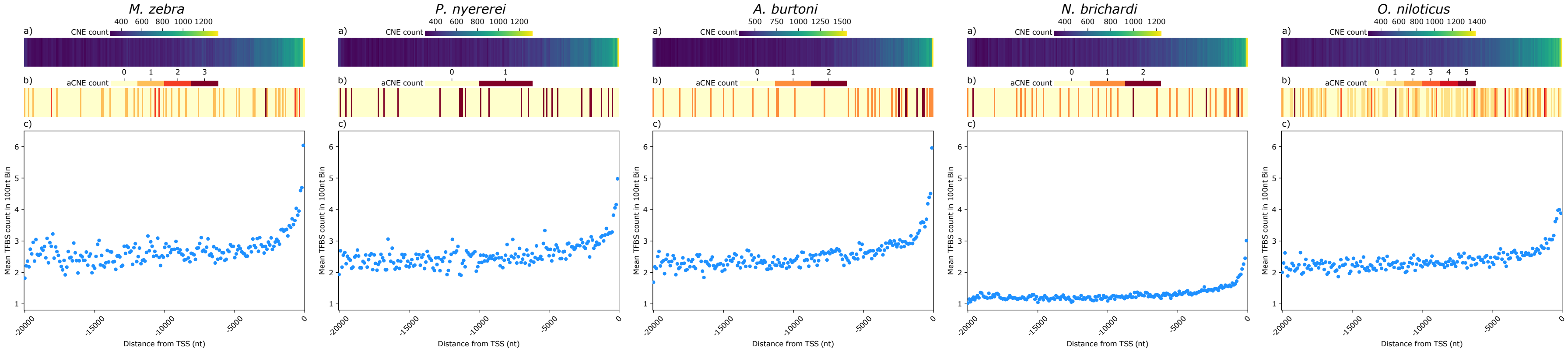

Fig. S22 - Counts of predicted TFBS in 100nt windows over 20kb regions upstream of the TSS in 5 cichlid species shown below counts of conserved non-coding elements (CNEs) and CNEs that significantly diverged from the neutral model (aCNEs) over the same regions (see Brawand, D. *et al.* 2014 *Nature*). Heatmaps represent total counts of a) CNEs and b) aCNEs 0base midpoints intersecting 100nt windows for 20kb upstream of the TSS in each species. CNE and aCNE annotations used in a) and b) are taken from Brawand, D. *et al.* 2014 *Nature*, and were intersected with the 20kb *cis* regions described below before counting. Each data point in c) represents the total count of TFBS predictions within a 100nt window divided by the number of genes with a *cis* region contributing to that bin. *Cis* regions were called as described in the methods, briefly - if a gene was within 20kb to another gene annotation, the *cis* region called from the TSS was truncated to the boundary of the other annotation if on the same strand, or to an equal midpoint between annotations if on opposite strands, otherwise a full 20kb from the TSS was called. TFBSs were counted in a bin if the 0-base midpoint of the prediction fell within the bounds of the bin. To generate TFBS predictions, these 20kb *cis* regions were scanned with the total, nonredundant set of core extrapolations and FIMO *de novo* TFBS predictions for the species, generated as described in the *Methods*.

Additional file 1 - tables

**Nr2c2 DNA-binding Domain (DBD)**		
Primer	*Sequence 5' > 3'*	*Notes*
mz-nb_T7_nr2c2DBD_F1	GGATCCTAATACGACTCACTATAGGGAACAGCCACCATGTCAGGAGATTTGAGCCGACCA	Primer can be used for amplification in both Mz and Nb as it does not cover variable regions. There are only 4 (degenerate) mismatched bases between Nb and Mz; still coding for a 100% conserved DBD.
mz-nb_T7_nr2c2DBD_R1	TTAGGGCACAATGTCGATGGGTTTCCTCTCACTCTGGACAGACTCAGTCTTCATCCCCAT	
**Rxrb DNA-binding Domain (DBD)**		
mz-nb_T7_rxrbDBD_F1	GGATCCTAATACGACTCACTATAGGGAACAGCCACCATGGCTCACAGCCCGGGAATAATG	Primer can be used for amplification in both Mz and Nb as it does not cover variable regions. There are only 3 (degenerate) mismatched bases between Nb and Mz; still coding for a 100% conserved DBD.
mz-nb_T7_rxrbDBD_R1	TTACTGTCGTTCCTCTTGTACCGCTTCCCTCTTCATTCCCATGGCCAGGCACTTCTGGTA	
**DNA probes**		
mz_cy5_nr2c2_rxrb-sws1TG_F1	TCATTAGTCAGAGTCAGAGGTCACAGGA	Since the TFBS is predicted to be shared by both nr2c2 and rxrb, only a single DNA probe can be used to test binding of both nr2c2 and rxrb DBDs.<br><br>Negative control oligos are scrambled motifs of the original sequence so as to maintain nucleotide composition.
mz_nr2c2_rxrb-sws1TG_R1	TCCTGTGACCTCTGACTCTGACTAATGA	
nb_cy5_nr2c2_rxrb-sws1TG_F1	TCATTAGTCAGGGTCAGAGGTCACAGGA	
nb_nr2c2_rxrb-sws1TG_R1	TCCTGTGACCTCTGACCCTGACTAATGA	
nb_cy5_nr2c2_rxrb-sws1TG-ve_F1	TTGGTGAAAAGTGACTGGCTGCAGCAAC	
nb_nr2c2_rxrb-sws1TG-ve_R1	GTTGCTGCAGCCAGTCACTTTTCACCAA	

Table S1. Oligonucleotides used for amplification of DNA-binding domains (DBDs) and EMSA DNA probes. The names and sequences are listed as pairs according to their usage.

Reference Species_ Comparison Species	Total variants	Three Prime UTR	aCNEs	hCNEs	Non-State-changed Promoters	State-changed Promoters	TFBSs	Non-State-changed Promoters TFBSs	State-changed Promoters TFBSs	Promoter flank	Other variants
mz11_pn1	7891416	197764	208	407996	610173	112568	1895509	1393708	501801	673391	6562707
mz11_ab1	9051743	230630	217	467713	696693	130742	2290382	1690816	599566	766937	7525748
mz11_nb1	18774705	537245	346	1026501	1449107	299816	3824346	2828340	996006	1622576	15461690
mz11_on11	32239921	1010838	368	1723742	2514873	550286	6062050	4449729	1612321	2853422	26439814
mz11_oryLat2	56596695	2239770	1175	7026932	3881344	866298	8379459	6164546	2214913	4923529	42581176
mz11_gasAcu1	64804903	3047571	1328	6872705	4506168	1082384	10632874	7735981	2896893	5736773	49294747
mz11_danRer7	24799703	622261	811	2802586	1487540	281167	1873134	1399465	473669	2155558	19605338
pn1_mz11	7891416	148832	652	524481	597081	118337	2076184	1512359	563825	632973	6502033
pn1_ab1	9474922	175499	628	584119	703023	143763	2306157	1671655	634502	751301	7867890
pn1_nb1	18837826	409594	761	1120740	1411056	312237	3803600	2767123	1036477	1556155	15583438
pn1_on11	31642942	754037	782	1773984	2409335	564952	5957661	4307448	1650213	2689308	26139852
pn1_oryLat2	55421413	1824348	1924	7072451	3733927	876813	8087791	5870042	2217749	4724492	41911950
pn1_gasAcu1	63474615	2442763	2323	6858563	4372824	1102629	10169486	7206188	2963298	5488028	48695513
pn1_danRer7	24243979	491588	1613	2915439	1475788	289715	1886208	1397056	489152	2083763	19069836
ab1_mz11	9051743	231059	550	617482	770949	138140	2543224	1856340	686884	788044	7293563
ab1_pn1	9474922	233267	550	615159	805441	144426	2413771	1779798	633973	817266	7676079
ab1_nb1	18755346	533312	667	1160524	1585165	308416	4046368	2957674	1088694	1668510	15167262
ab1_on11	31664404	989531	667	1837439	2692342	553697	6375972	4693714	1682258	2879081	25590728
ab1_oryLat2	55577322	2301485	1783	7315599	4152372	865748	8524348	6282664	2241684	5031746	40940335
ab1_gasAcu1	63706777	3057942	1975	7100149	4805932	1081607	10791519	7845950	2945569	5889798	47659172
ab1_danRer7	24308154	702771	1553	3052445	1575078	289508	2011002	1499144	511858	2210833	18686799
nb1_mz11	18774705	398795	854	1301826	1337025	317229	2787465	1901920	885545	1530873	15418976
nb1_pn1	18837826	399483	853	1287676	1339698	318890	2657360	1816406	840954	1538196	15491226
nb1_ab1	18755346	396301	837	1282981	1335049	318257	2670287	1809999	860288	1530025	15421921
nb1_on11	30695242	728828	813	1925303	2215016	559320	3997080	2714678	1282402	2575948	25265962

nb1_oryLat2	54190417	1793433	2187	7494458	3714716	884265	5205418	3545293	1660125	4629695	40301358
nb1_gasAcu1	62161124	2366297	2657	7315298	4316790	1095410	6590051	4443651	2146400	5396192	47064672
nb1_danRer7	23639358	519443	2019	3263109	1477914	293462	1244362	863477	380885	2068726	18083411
on11_mz11	32239921	1190969	530	1582304	2724962	518287	6569717	4810884	1758833	3069068	26222869
on11_pn1	31642942	1178735	520	1548053	2662480	511247	6196776	4534352	1662424	3011485	25741907
on11_ab1	31664404	1175171	531	1547076	2666456	511971	6197384	4568469	1628915	3012662	25763199
on11_nb1	30695242	1149444	496	1532045	2580351	499713	6036531	4430817	1605714	2924384	24933193
on11_oryLat2	55525414	2572618	1899	6592191	4263518	829077	9196930	6816539	2380391	5207882	41266111
on11_gasAcu1	63636292	3447429	2199	6469516	4991043	1064329	7589059	4443651	3145408	6129422	47661776
on11_danRer7	24264982	827485	1482	2520426	1560561	238361	1843646	1358928	484718	2182041	19116667

Table S2 - Species pairwise variants overlapping various genomic regions. Pairwise variant calling and overlap to various genomic regions defined in *Methods.* Five cichlid species (mz11 – *M. zebra;* pn1 – *P. nyererei;* ab1 – *A. burtoni;* nb1 – *N. brichardi;* and on11 – *O. niloticus*) and three outgroup teleost species (oryLat2 - medaka, gasAcu1 - stickleback and danRer7 - zebrafish) defined within.

Species	No. of gene nodes	Co-expressed TF-TG edges	TF-TG (promoter TFBS) edges
M. zebra	11,075	3,964	4,760,610
P. nyererei	11,070	4,029	4,862,871
A. burtoni	11,638	3,822	5,355,927
N. brichardi	10,015	3,180	3,292,032
O. niloticus	11,790	4,099	5,896,075

Table S3 - Number of statistically significant edges in species reconstructed networks. Network edges derived from several sources (see *Methods*), representing various regulatory interactions/associations in the five cichlids.

Additional file 2 and 3 legends

Additional file 2: Fig. S1 - Gene Ontology (GO) enrichment of modules across all extant and ancestral species. Enriched terms of significance FDR-corrected *P*-value (*q*-value <0.05) in modules (rows and ':n' module number) are shown for extant and ancestral species (columns) and colored according to module and gradient, -log(*q*-value) in each grid position (see legend, *left*). Set-based hypergeometric test of enrichment carried out using a background of all module genes.

Additional file 2: Fig. S2 - Transcription factor motif enrichment of module gene promoters across all extant and ancestral species. All enriched motifs of significance FDR-corrected *P*-value (*q*-value <0.05) in modules (rows and ':n' module number) are shown for extant and ancestral species (columns) and colored according to module and gradient, -log(*q*-value) in each grid position (see legend, *left*). Set-based hypergeometric test of enrichment carried out using a background of all motifs predicted within all module gene promoters.

Additional file 2: Fig. S3-S8 - Heatmap matrices of enrichment, expression and Pearson correlation between the two for each motif, across all six tissues (brain, eye, heart, kidney, muscle and testis). First five columns are gradient coloured (legend to *right*) according to enriched motifs of significance FDR-corrected *P*-value (*q*-value <0.05) in module gene promoters (rows as '_n' module number and '_TF' motifs) shown as *-log*(q-value) in all five extant species (columns). Next five columns are tissue-specific zero-mean log-expression expression ratios (legend to *right)* used as input for Arboretum (see *Methods*) in all five extant species (columns). Final column is Pearson correlation coefficient (r) of motif enrichment and expression values shown in previous columns. r ranges (legend to *right)* from 1 (positive linear correlation, blue), 0 (no linear correlation, white), and −1 (negative linear correlation, red).

Additional file 3: Table S1 - DyNet[12] rewiring scores of TF-TG 1-to-1 edges when all five species networks are compared.

Additional file 3: Table S2 - List of candidate genes from previous molecular evolutionary and developmental studies[1,13,14,25] potentially associated with phenotypic novelty in cichlids that overlap the six tissues we studied.

Additional file 3: Table S3- DyNet[12] rewiring scores of candidate genes in TF-TG 1-to-1 edges when all five species networks are compared.

Additional file 3: Table S4 - DyNet[12] rewiring scores of TF-TG all edges when all five species networks are compared.

Additional file 3: Table S5- DyNet[12] rewiring scores of candidate genes in TF-TG all edges when all five species networks are compared.

Additional file 3: Table S6 – Rate of edge gain and loss in TF-TG all edges across the five cichlid phylogeny. Rates that are >100 (and excluded from analyses) are rank shaded blue (gain) and green (loss) whereas likelihood scores are rank shaded orange.

References

1. Bloomquist RF, Fowler TE, Sylvester JB, Miro RJ, Streelman JT. A compendium of developmental gene expression in Lake Malawi cichlid fishes. BMC Dev Biol. 2017;17:3.

2. Maruska KP, Fernald RD. Social regulation of male reproductive plasticity in an African cichlid fish. Integr Comp Biol. 2013;53:938–50.

3. Browman HI, Hawryshyn CW. Retinoic Acid Modulates Retinal Development in the Juveniles Op a Teleost Fish. J Exp Biol. 1994;193:191–207.

4. Yanai I, Benjamin H, Shmoish M, Chalifa-Caspi V, Shklar M, Ophir R, et al. Genome-wide midrange transcription profiles reveal expression level relationships in human tissue specification. Bioinformatics. 2005;21:650–9.

5. Nguyen T, Di Giovanni S. NFAT signaling in neural development and axon growth. Int J Dev Neurosci. 2008;26:141–5.

6. Witzel HR, Cheedipudi S, Gao R, Stainier DYR, Dobreva GD. Isl2b regulates anterior second heart field development in zebrafish. Sci Rep. 2017;7:41043.

7. Takechi M, Seno S, Kawamura S. Identification of cis-acting elements repressing blue opsin expression in zebrafish UV cones and pineal cells. J Biol Chem. 2008;283:31625–32.

8. Karlstrom RO, Tyurina O V., Kawakami A, Nishioka N, Talbot WS, Sasaki H, et al. Genetic analysis of zebrafish gli1 and gli2 reveals divergent requirements for gli genes in vertebrate development. Development. 2003;130:1549–64.

9. Karlstrom RO, Trowe T, Klostermann S, Baier H, Brand M, Crawford AD, et al. Zebrafish mutations affecting retinotectal axon pathfinding. Development. 1996;123:427–38.

10. O'Quin KE, Smith D, Naseer Z, Schulte J, Engel SD, Loh YHE, et al. Divergence in cis-regulatory sequences surrounding the opsin gene arrays of African cichlid fishes. BMC Evol Biol. 2011;11:120.

11. Carleton K. Cichlid fish visual systems: mechanisms of spectral tuning. Integr Zool. 2009;4:75–86.

12. Goenawan IH, Bryan K, Lynn DJ. DyNet: Visualization and analysis of dynamic

molecular interaction networks. Bioinformatics. 2016;32:2713–5.
13. Brawand D, Wagner CE, Li YI, Malinsky M, Keller I, Fan S, et al. The genomic substrate for adaptive radiation in African cichlid fish. Nature. 2014;93:17–9.
14. Hahn C, Genner MJ, Turner GF, Joyce DA. The genomic basis of cichlid fish adaptation within the deepwater “twilight zone” of Lake Malawi. Evol Lett. 2017;1:184–98.
15. Whited JL. Dynactin is required to maintain nuclear position within postmitotic Drosophila photoreceptor neurons. Development. 2004;131:4677–86.
16. Cohen DR, Cheng CW, Cheng SH, Hui CC. Expression of two novel mouse Iroquois homeobox genes during neurogenesis. Mech Dev. 2000;91:317–21.
17. Choy SW, Cheng CW, Lee ST, Li VWT, Hui MNY, Hui CC, et al. A cascade of irx1a and irx2a controls shh expression during retinogenesis. Dev Dyn. 2010;239:3204–14.
18. Malinsky M, Svardal H, Tyers AM, Miska EA, Genner MJ, Turner GF, et al. Whole-genome sequences of Malawi cichlids reveal multiple radiations interconnected by gene flow. Nat Ecol Evol. 2018;2:1940–55.
19. Conte MA, Kocher TD. An improved genome reference for the African cichlid, Metriaclima zebra. BMC Genomics. 2015;16:724.
20. Conte MA, Joshi R, Moore EC, Nandamuri SP, Gammerdinger WJ, Roberts RB, et al. Chromosome-scale assemblies reveal the structural evolution of African cichlid genomes. Gigascience. 2019;8:giz030.
21. Musser JM, Arendt D. Loss and gain of cone types in vertebrate ciliary photoreceptor evolution. Dev Biol. 2017;431:26–35.
22. Mears AJ, Kondo M, Swain PK, Takada Y, Bush RA, Saunders TL, et al. Nrl is required for rod photoreceptor development. Nat Genet. 2001;29:447–52.
23. Evans RM, Mangelsdorf DJ. Nuclear receptors, RXR, and the big bang. Cell. 2014;157:255–66.
24. Olivares AM, Moreno-Ramos OA, Haider NB. Role of nuclear receptors in central nervous system development and associated diseases. J Exp Neurosci. 2015;9:93–121.
25. Hofmann CM, O’Quin KE, Justin Marshall N, Cronin TW, Seehausen O, Carleton KL.

The eyes have it: Regulatory and structural changes both underlie cichlid visual pigment diversity. PLoS Biol. 2009;7:e1000266.
26. Froese R, Pauly D. Fishbase [Internet]. FishBase. 2017. Available from: www.fishbase.org
27. Roy S, Wapinski I, Pfiffner J, French C, Socha A, Konieczka J, et al. Arboretum: Reconstruction and analysis of the evolutionary history of condition-specific transcriptional modules. Genome Res. 2013;23:1039–50.
