# Supplementary material for: Evolution of regulatory networks associated with traits under selection in cichlids
Source: Genome Biol. 2021 Jan 8;22:25. doi: 10.1186/s13059-020-02208-8 (PMC7791837; doi:10.1186/s13059-020-02208-8)
Supplement: Supplementary file 4 — Additional file 4. Review history. [file 13059_2020_2208_MOESM4_ESM.docx]

**Review history**

**First round of review**

**Reviewer 1**

The manuscript reports a novel computational pipeline to analyze gene regulatory networks in phylogenetic context and applies this to data on cichlid fishes to demonstrate how it works. Evidence if found to suggest several cases network rewiring providing interesting insights on regulatory networks and their phenotypic effects/associations during evolution.

As far as I am concerned, the analyses appear sound and the interpretations follow from the results. What I was left thinking was that the Results section was written in quite long-winded way often mixing results and interpretations, the latter which would be best left to discussion (to some degree of course matter of taste). Also, the conclusion paragraph large re-iterates the abstract, and could shortened

L150 and thereafter. Pearson product moment correlation is universally referred to as "r" - why this new shorthand? Also, when reporting correlations, authors did not provide n- and p-values. Unclear was also whether multiple testing adjustments were made.

L563-565. I think it would be good if authors provide basic information about sample sizes sample origins and preparation rather than referring to a paper published in 2014. Every paper should be self-standing, and readers should no need consult other publications to understand how the research was conducted.

Reference list: there is quite a lot of inconsistencies in reporting (for instance missing pa ge numbers etc.).

**Reviewer 2**

In this interesting manuscript, Tarang Mehta et al. study the evolution of gene regulatory networks in African cichlid fishes using genomic information from the five reference cichlid genomes published under the leadership of Federica Di-Palma. Not least because of this study from 2014, cichlids have become an important model taxon in evolutionary genomic research (in addition to being an important model in adaptive radiation and speciation research). However, while a number of candidate genomic features in cichlid adaptive radiations have been studied in detail, very little is known about the evolution of regulatory networks in these fish. The authors of the present manuscript fill this gap by presenting an impressive integrative analysis of gene regulatory networks in cichlids on the basis of the Arboretum algorithm and subsequent computational analyses. The authors present a first global analysis of regulatory network evolution in cichlids and identify many novel potential candidate regulators and regulatory regions for cichlids as valuable resource for the community. They also provide an in-depth analysis of regulatory variation in the visual system, including validation experiments for a promoter of the sws1 opsin gene.

Overall, I find this is a well-done study that would fit well into the target journal. I nevertheless have a few points that the authors should address/clarify (none of which is substantial):

(1) In the abstract and elsewhere, the authors consider the species flocks of cichlid fishes in the African Great Lakes Victoria, Malawi and Tanganyika as a single adaptive radiation. It would be more precise, however, to consider these as three independent adaptive radiations, as these occurred in different lakes, started out at different time points in the past and from different ancestors. The authors also suggest that these ancestors were all haplochromine cichlids (line 83), which is true for lakes Victoria and Malawi but not for Tanganyika (see e.g. Fig. 4 in my review paper from 2018).

(2) Besides being role models of the phenomenon of adaptive radiation, African lake cichlids are also a textbook example of convergent evolution. I am thus wondering if the authors have looked into convergent state-changes in their network analyses. For example, would it be possible to also map the changes on the branches leading to the species in Fig. 1B and ask the question if some of these changes have occurred on more than one branch (including the internal ones)?

(3) In the last part of the results, the authors intend to test gene regulatory network rewiring in the "context of phylogeny and ecology" using available data for Lake Malawi. However, I did not find any indication if a formal statistical test taking phylogeny into consideration was applied here. This would, in my opinion, be important.

(4) Based on the description on lines 585-597, it remains unclear to me how the authors arrived at the number of modules of 10 as presented in the main manuscript and in Fig. 1. Their choice seems somewhat arbitrary to me. In my opinion, it would be important to show a version of Figure 1 (e.g. in the supplement) with k=19 as determined by Arboretum (l. 588).

(5) Perhaps I have overlooked this, but I do not think that it is made clear which versions of the genomes have been used in this study (the original versions published in the Brawand paper or newer versions including PacBio data for some of the species?). I only found this information for M. zebra (l. 817).


Minor points:
l. 81: should be "adaptive radiation" instead of "adaptive speciation" (but see point (1) above).
l. 184-187: This sentence should be rephrased, I am having a hard time getting it.
l. 273: I would suggest calling these "candidate" trait genes, as very few of those have been functionally confirmed.
l. 311: The notion of "unique cichlid traits" is not really correct here. Especially the visual system, which is in the focus of this paragraph, is not unique to cichlids. I would tone down this section. It is probably enough to say that these traits have been shown to be important in cichlid diversification.
l. 316: "we previously noted", please add a reference then.
l. 375: "this, we" instead of "this ,we".

**Authors’ response to reviewers**

Reviewer #1:

The manuscript reports a novel computational pipeline to analyze gene regulatory networks in phylogenetic context and applies this to data on cichlid fishes to demonstrate how it works. Evidence if found to suggest several cases network rewiring providing interesting insights on regulatory networks and their phenotypic effects/associations during evolution.

As far as I am concerned, the analyses appear sound and the interpretations follow from the results.

Thank you for your positive comments and please allow us to address your points below.

What I was left thinking was that the Results section was written in quite long-winded way often mixing results and interpretations, the latter which would be best left to discussion (to some degree of course matter of taste). Also, the conclusion paragraph large re-iterates the abstract, and could shortened

To rectify the long-winded nature of the results, we have moved some relevant interpretations (whilst avoiding any repetition) to the discussion. This includes:

- Line 116-120 (from original submission) moved to discussion (see Line 419 to 424) and shortened in results (see Line 116)
- Line 177-180 (from original submission) moved to discussion (see Line 434 to 437) and a shortened interpretation is retained in the results (see Line 178-180)
- Line 199-201 (from original submission) has been removed and the start of the next paragraph has been made clearer (see Line 201-204).
- Line 290-292 (from original submission) has been shortened (see Line 287 to 289) and interpretations added to discussion (see Line 444 to 446).
- Line 322-324 (from original submission) has been shortened with the main interpretation removed since it exists in the discussion.
- Line 333-339 (from original submission) has been moved to the discussion (see Line 450-458)
- Line 361-364 (from original submission) has been simplified to become less interpretive.

Furthermore, we have duly shortened the conclusion paragraph so as to not repeat abstract content.

L150 and thereafter. Pearson product moment correlation is universally referred to as "r"

- why this new shorthand? Also, when reporting correlations, authors did not provide n- and p-values. Unclear was also whether multiple testing adjustments were made.

All Pearson correlation coefficient values have now been updated to the standard “r”. The correlations were calculated across species, with n=5. We have updated the text to mention n (see line 150 and thereafter in main text). We have also added the unadjusted *p*-value for the individual TF correlation examples we highlight in the text (see line 162 and thereafter in main text). All n- and p- values have also been added to highlighted TF examples in the supplementary information. We tried to apply an FDR correction to the

*p*-values, however, owing to the small number of samples (n=5), very few survived an FDR<0.05. From the original submission, the scatter plots for selected TF examples we discuss are still retained (see supplementary information Fig. S-R4e-f). Furthemore, we have updated the number of TFs to correctly reflect only unique TFs out of the total 337 TFs (see line 154-159). As a result, no interpretations have changed.

L563-565. I think it would be good if authors provide basic information about sample sizes sample origins and preparation rather than referring to a paper published in 2014. Every paper should be self-standing, and readers should no need consult other publications to understand how the research was conducted.

Details of samples used have been updated in the relevant ‘Inference of multi- and single- tissue transcriptional modules in five cichlids’ section of the methods (see line 576-597).

Reference list: there is quite a lot of inconsistencies in reporting (for instance missing page numbers etc.).

We have duly corrected any referencing inconsistencies in the main and supplementary text.

Reviewer #2

In this interesting manuscript, Tarang Mehta et al. study the evolution of gene regulatory networks in African cichlid fishes using genomic information from the five reference cichlid genomes published under the leadership of Federica Di-Palma. Not least because of this study from 2014, cichlids have become an important model taxon in evolutionary genomic research (in addition to being an important model in adaptive radiation and speciation research). However, while a number of candidate genomic features in cichlid adaptive radiations have been studied in detail, very little is known about the evolution of regulatory networks in these fish. The authors of the present manuscript fill this gap by presenting an impressive integrative analysis of gene regulatory networks in cichlids on the basis of the Arboretum algorithm and subsequent computational analyses. The authors present a first global analysis of regulatory network evolution in cichlids and identify many novel potential candidate regulators and regulatory regions for cichlids as valuable resource for the community. They also provide an in-depth analysis of regulatory variation in the visual system, including validation experiments for a promoter of the sws1 opsin gene.

Overall, I find this is a well-done study that would fit well into the target journal. Thank you for the positive review and please find our comments below.

I nevertheless have a few points that the authors should address/clarify (none of which is substantial):

(1) In the abstract and elsewhere, the authors consider the species flocks of cichlid fishes in the African Great Lakes Victoria, Malawi and Tanganyika as a single adaptive radiation. It would be more precise, however, to consider these as three independent adaptive radiations, as these occurred in different lakes, started out at different time points in the past and from different ancestors. The authors also suggest that these ancestors were all haplochromine cichlids (line 83), which is true for lakes Victoria and Malawi but not for Tanganyika (see e.g. Fig. 4 in my review paper from 2018).

Indeed this is true - we have amended all relevant text so as to not suggest that the Great Lakes Victoria, Malawi and Tanganyika are a single adaptive radiation (see lines

33-34, 80-81, 83 and 406-408). Furthermore, we have removed ‘haplochromine’ as indeed this is not true for the Tanganyika radiation.

(2) Besides being role models of the phenomenon of adaptive radiation, African lake cichlids are also a textbook example of convergent evolution. I am thus wondering if the authors have looked into convergent state-changes in their network analyses. For example, would it be possible to also map the changes on the branches leading to the species in Fig. 1B and ask the question if some of these changes have occurred on more than one branch (including the internal ones)?

This is a valuable point considering the majority of our analysis focuses on differences, as opposed to convergence between species.

By focusing on state changes of 6,844 1-to-1 orthologous genes, we identified convergent state changes of 732 genes along all ancestral nodes versus Anc4. This is made up of 772 genes in Anc3 and Anc2, 734 genes in Anc3 and Anc1, and 996 genes in Anc2 and Anc1 (Fig. S-R1c), including a few TFs (46 TFs - Anc3-2-1; 49 TFs - Anc3- 2; 46 TFs - Anc3-1; 66 TFs - Anc2-1) such as *tbx20, nkx3-1* and *hoxd10.* At each ancestral node, there are generally more unique state changes of genes against the deepest common ancestor (Anc4) (Fig. 1b), than convergent state changes between ancestral nodes when compared to Anc4 (Fig. S-R1c).

When mapping convergent state changes on species branches (focal species vs any other species), we note that there are 463 genes in *M. zebra*, 443 in *P. nyererei*, 394 in

*A. burtoni*, 521 in *N. brichardi*, and 527 in *O. niloticus* (orange numbers, Fig. 1b). In each species (except *P. nyererei*)*,* there are more state changes and expression divergence of genes, than convergent state changes with any of the other species (purple vs orange numbers, Fig. 1b). All convergent state changes between two, three and four species is further summarised in the venn diagram (Fig. S-R1d); convergence of genes is highest in 1) *N. brichardi* and *O. niloticus* (229 genes) when comparing two species versus the rest; 2) the haplochromines (358 genes) when comparing three species versus the rest; and 3) the three haplochromines and *N. brichardi* (983 genes) when comparing four species with the remaining single species. The fewest convergent state changes are generally observed when comparing evolutionary distant species e.g. *M. zebra* and *A. burtoni* (41 genes) versus the rest (Fig. S-R1d). Overall, convergent state changes are generally higher between phylogenetically closer species but in general, lower than unique state changes between species (Fig. 1b; Fig. S-R1c-d).

These details have been included/summarised:

- In Line 127-132 in the main text.
- As additional (orange) numbers on branches in Fig. 1b. These are convergent state changes in each of the ‘focal’ species and any of the other species.
- In Line 16-37 in supplementary information.
- As venn diagrams (Fig. S-R1c and Fig. S-R1d) in supplementary information.

(3) In the last part of the results, the authors intend to test gene regulatory network rewiring in the "context of phylogeny and ecology" using available data for Lake Malawi. However, I did not find any indication if a formal statistical test taking phylogeny into consideration was applied here. This would, in my opinion, be important.

To remind, in this section of the results we focus on variants at visual opsin regulatory sites (gene promoter TFBSs), using *M. zebra* as a reference for mapping to corresponding regions in fellow Lake Malawi species. Here, the ‘context of phylogeny and ecology’ is taken as mapping corresponding sites onto the least controversial and all included Lake Malawi species phylogeny (Malinsky M, *et al.* Nat Ecol Evol. 2018).

If the TFBSs are likely functional, we hypothesise that radiating species with similar foraging habits would share conserved regulatory genotypes, to possibly regulate and tune similar spectral sensitivities; whereas distally related species with dissimilar foraging habits would segregate at the corresponding regulatory site. We started with 157,232 sites that 1) have identified variation between the five cichlid species; and 2) are located in TFBSs of *M. zebra* candidate gene promoters. In total, we identified 5,710/157,232 sites with between-species variation across 73 Lake Malawi species (Fig. S-R3b), that also exhibited flanking sequence conservation, representative of shared ancestral variation.

We believe that the reviewer is asking for a formal statistical test of the data, independent of the phylogeny, to test for any change in the correlation once the phylogeny is fitted. This analysis is indeed important and therefore, we did the following:

1) categorically coded genotypes (of NR2C2>*sws1* and GATA2A>*rho*), visual trait and ecological measurements for each of the 73 Lake Malawi species (119 individuals), and
2) applied a phylogenetic independent contrasts (PICs) test (Felsenstein, J. *Am Nat* 1985) on all correlations with the TFBS genotypes (genotype vs wavelength palette, genotype vs habitat, and genotype vs foraging habit/diet). PICs assumes a linear relationship and process of Brownian motion between traits, and thus, for each combination of data, we firstly generated a scatterplot and tested the regression without any phylogenetic information, and then tested any change in the correlation once the published Lake Malawi phylogeny (Malinsky M, *et al.* Nat Ecol Evol. 2018) and regression model is fitted.

When the Lake Malawi phylogeny is introduced, and a regression model fitted, there is very little change in correlation (for both NR2C2>*sws1* and GATA2A>*rho* genotypes) of all combinations (wavelength palette, habitat and foraging habit/diet) versus genotype (Fig. S-R4g-r). Since the regression slopes are similar, it appears that phylogenetic signal does not have an impact on the association between the tested visual traits, ecology and TFBS genotypes. However, all adjusted r^2^ values are very low, and therefore suggest a weak linear relationship for all associations, that is likely due to 1) missing or incomplete visual trait and ecological data; and 2) testing the influence of few variables on a polygenic trait. This analysis could therefore be developed in future studies with 1) the addition of any missing data (wavelength palette, habitat and/or foraging habit/diet) in the phylogeny; 2) the addition of further variables e.g. average water depth measurements; 3) additional species data from lowly represented clades

e.g. Mbuna; and 4) further experimental testing, particularly in phenotypically divergent species pairs.

We have summarised this additional analysis in the main text (see lines 385-388 of Results; lines 490-498 of Discussion; and lines 871-884 of Methods) and figures are included in the supplementary information (see Fig. S-R4g-r).

(4) Based on the description on lines 585-597, it remains unclear to me how the authors arrived at the number of modules of 10 as presented in the main manuscript and in Fig.

1. Their choice seems somewhat arbitrary to me. In my opinion, it would be important to show a version of Figure 1 (e.g. in the supplement) with k=19 as determined by Arboretum (l. 588).

We apologize for the confusion, the k=19 setting was for an earlier set of results and was left unchanged mistakenly. To determine the number of modules, we ran Arboretum with k=7-14 modules and selected the k that looked best on a combination of Silhouette index, data likelihood and manual inspection of the patterns associated with each cluster. Based on this, k=10 looked best followed by k=12. The k=10 modules are shown in Figure1 and we have added k=12 as a supplementary figure (see supplementary information Fig. S-M2b). We have also included the silhouette index and data likelihood plots as supplementary figures (see supplementary information Fig. S-M2a). The methodology has also been updated accordingly (see lines 619-623 of Methods).

(5) Perhaps I have overlooked this, but I do not think that it is made clear which versions of the genomes have been used in this study (the original versions published in the Brawand paper or newer versions including PacBio data for some of the species?). I only found this information for M. zebra (l. 817).

This was not overlooked - we used all Brawand version genomes and have now duly included this information in the ‘Functional and transcription factor binding site (TFBS) enrichment in modules’ section of the methods (see line 667-673). This is where the genomes are first used for defining gene promoter regions and motif discovery.

Owing to the nature of our comparative approach, and to alleviate any genome-based bias e.g. motif scanning in superior vs inferior genomes, we did not use either of the *M. zebra* or *O. niloticus* PacBio assemblies for our approach. We did however ensure that all functional validations of *M. zebra* and *O. niloticus* targets are cross-checked for 100% sequence identity to the corresponding region in the respective PacBio genome.

Minor points:

l. 81: should be "adaptive radiation" instead of "adaptive speciation" (but see point (1) above). Amended - see line 81.

l. 184-187: This sentence should be rephrased, I am having a hard time getting it. Amended by splitting and rewording - see line 184-188.

l. 273: I would suggest calling these "candidate" trait genes, as very few of those have been functionally confirmed. Amended - see line 271.

l. 311: The notion of "unique cichlid traits" is not really correct here. Especially the visual system, which is in the focus of this paragraph, is not unique to cichlids. I would tone down this section. It is probably enough to say that these traits have been shown to be important in cichlid diversification. Amended to “several genes that are important for cichlid diversification” - see line 308.

l. 316: "we previously noted", please add a reference then. This has been changed to “we note” as it does not refer to a publication, but instead to results in a previous section

- see line 314.

l. 375: "this, we" instead of "this ,we". Amended - see line 364.

**Second round of review**

**Reviewer 2**

I think that the authors have adequately addressed my open points and have provided additional information where necessary. In particular, they are more accurate now on the taxonomy of the cichlid adaptive radiations in East Africa, they report additional information in Fig. 1b (and elsewhere) on convergent state changes and they have clarified my questions related to phylogeny. In my opinion, this manuscript should now be accepted and I would like to congratulate the authors for their work
